# A Catalytic Asymmetric Pictet–Spengler Platform as a Biomimetic Diversification Strategy toward Naturally Occurring Alkaloids

**DOI:** 10.1021/jacs.2c06664

**Published:** 2022-08-17

**Authors:** Manuel J. Scharf, Benjamin List

**Affiliations:** Max-Planck-Institut für Kohlenforschung, Kaiser-Wilhelm-Platz 1, 45470Mülheim an der Ruhr, Germany

## Abstract

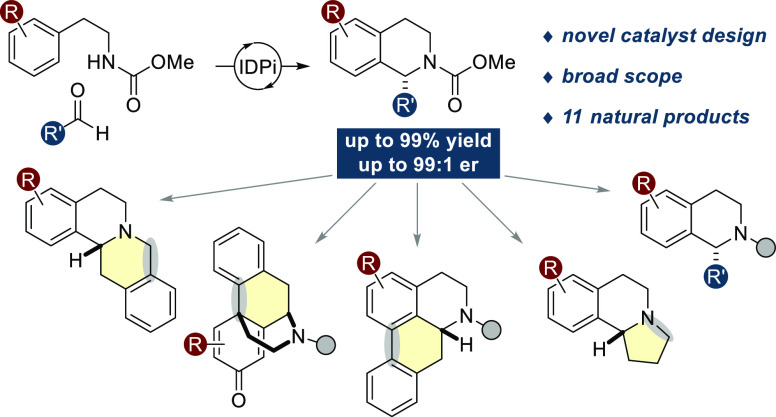

Tetrahydroisoquinoline (THIQ) alkaloids constitute a large and diverse class of bioactive natural products, with the parent compounds and related downstream biosynthetic secondary metabolites spanning thousands of isolated structures. Chemoenzymatic synthetic approaches toward the relevant THIQs rely on Pictet–Spenglerases such as norcoclaurine synthase (NCS), the scope of which is strictly limited to dopamine-related phenolic substrates. To overcome these limitations in the context of chemical synthesis, we herein report asymmetric Pictet–Spengler reactions of *N*-carbamoyl-β-arylethylamines with diverse aldehydes toward enantioenriched THIQs. The obtained products proved to be competent intermediates in the synthesis of THIQ, aporphine, tetrahydroberberine, morphinan, and androcymbine natural products. Novel catalyst design with regard to the stabilization of cationic intermediates was crucial to accomplish high reactivity while simultaneously achieving unprecedented stereoselectivity for the reaction of biologically relevant substrates.

Tetrahydroisoquinoline (THIQ) natural products and related alkaloids constitute one of the oldest known classes of biologically active compounds.^1^ While benzylisoquinoline metabolites are primarily found in members of the Ranunculales plants,^2,3^ potent THIQ antitumor antibiotics have been isolated from bacteria and marine organisms and their pharmaceutical potential remains an active field of investigation.^4−6^ Fascinatingly, strong biological activity within these alkaloids can be observed in simple phenethylamines (mescaline), small-molecule THIQs (salsolinol), and the complex molecular frameworks in the seeds of *Papaver somniferum* (opiates).^7^

Research on THIQs has stimulated innovation in the field of chemical synthesis for over 100 years. Exemplarily, the Pictet–Spengler reaction was developed in 1911 as a novel synthetic strategy toward opium alkaloids.^8^ It was noted in the seminal report that the alternative Bischler–Napieralski strategy^9^ including a successive reduction step can be inefficient and synthetically challenging. Nonetheless, due to the emergence of powerful catalytic asymmetric hydrogenation methods, the synthesis of enantioenriched THIQs via dehydration of amides with subsequent reduction remains a frequently employed approach to date.^10^ The arguably most atom-,^11^ redox-,^12^ and step-economical^13^ catalytic asymmetric Pictet–Spengler reaction, while well explored in the synthesis of tryptoline alkaloids,^14−17^ remains significantly underdeveloped in the context of THIQs.

Remarkably, the vast majority of THIQ-derived natural products originates from a single biosynthetic intermediate, (*S*)-norcoclaurine (Figure 1A).^3,18,19^ It is produced from dopamine by a Pictet–Spenglerase, namely norcoclaurine synthase (NCS), which can furthermore be leveraged to catalyze Pictet–Spengler reactions with an extended scope of aldehydes and ketones.^20−24^ Aided by crystal structure analysis, the underlying reaction mechanism has been investigated computationally to explain the observed reactivity patterns.^25,26^ Specifically, an acidic side chain generates a reactive iminium ion that is nucleophilically intercepted by the electron-rich aromatic ring under the base assistance of a lysine residue in close proximity, generating a neutral dienone intermediate. This catalytic cascade underpins the necessity of dopamine-related phenolic substrates and directly explains the observed loss of reactivity upon removal of this crucial hydroxyl group.^21^

**Figure 1:**
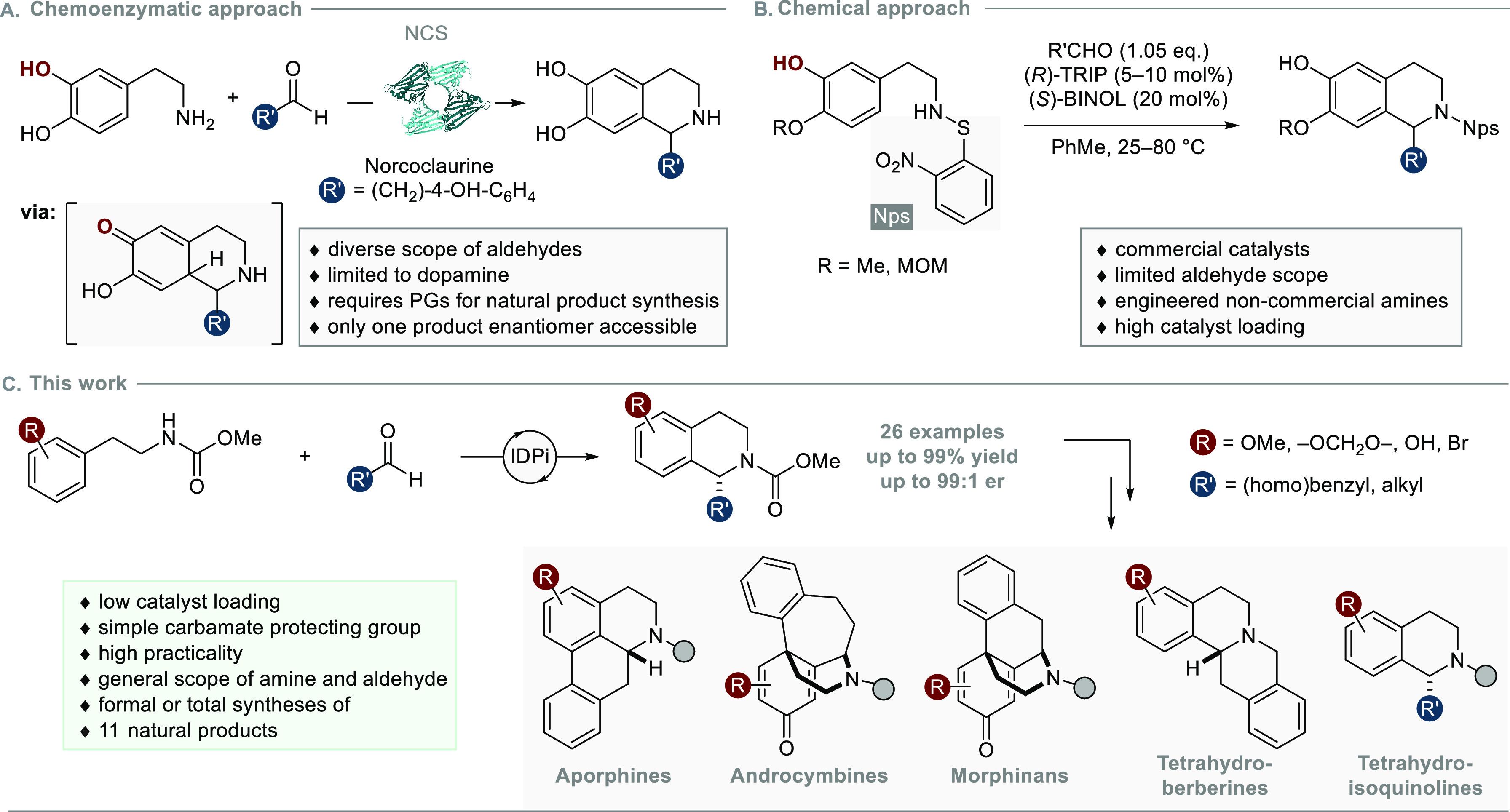
Catalytic asymmetric Pictet–Spengler reactions toward enantioenriched tetrahydroisoquinoline (THIQ) natural products: (A) chemoenzymatic strategy using engineered norcoclaurine synthase (NCS); (B) chemical approach using chiral phosphoric acid (CPA) catalysis; (C) this work: general organocatalytic asymmetric Pictet–Spengler reactions with simple and synthetically relevant *N*-carbamoyl-β-arylethylamines toward diverse members of the THIQ family of alkaloids. IDPi = imidodiphosphorimidate.

Only a few methodologies have been reported with regard to catalytic asymmetric Pictet–Spengler reactions toward THIQs. The work of Hiemstra and co-workers stands out as the only nonenzymatic direct catalytic asymmetric Pictet–Spengler reaction with dopamine-derived substrates (Figure 1B).^27−29^ The developed system was applied to the synthesis of benzylisoquinoline natural products with high selectivity. While this method is strictly limited to phenolic substrates and an uncommon *o*-nitrophenylsulfenyl (Nps) activating group, a variety of alkaloids could be obtained with high enantiopurity after product recrystallization. Nevertheless, high catalyst loadings were necessary and insufficient stereoselectivity was observed in the reaction of many unbiased aliphatic and aromatic aldehydes.

Motivated by our group’s advancements in the field of asymmetric counteranion-directed catalysis (ACDC), we set out to develop an ideal direct catalytic asymmetric Pictet–Spengler reaction for the synthesis of THIQs and related natural products. Key to our design is the independence from phenolic hydrogen bond donors in the substrate, a stipulation that necessitates strongly acidic catalysts capable of dictating stereocontrol via noncovalent interactions such as nonobvious hydrogen bonding from the enantiopure counteranion. Furthermore, while the use of an *N*-protecting group is deemed necessary to achieve reactivity, we chose simple carbamates that can be readily reduced to the corresponding *N*-methyl species present in most THIQ alkaloids, further increasing the step economy of our approach. We herein wish to report the successful realization of the above outlined design (Figure 1C).

To initiate our investigation, we studied the Pictet–Spengler reaction of *N*-carbamoyl-homoveratrylamines **1** and phenylacetaldehyde (Table 1). While relatively nonacidic organocatalysts (p*K*_a_ ≥ 8.4 in CH_3_CN;^30,31^ see the Supporting Information for further details) failed to show any reactivity, we were pleased to observe traces of product formation at ambient temperature with moderately acidic IDPi catalyst **3a** (p*K*_a_ = 4.5 in CH_3_CN; entry 1).^30^ Installment of electron-withdrawing CF_3_ groups in catalyst **4a** expectedly improved the catalytic activity via enhanced acidity to give the product in 46% yield (entry 2). An even stronger reactivity enhancement was accomplished by introducing electron-rich 2-benzofuran substituents in IDPi **5a**, presumably due to their capability to stabilize reactive cationic intermediates via cation−π interactions.^32−34^ Thus, we obtained the THIQ in 60% yield and with promising enantioinduction (entry 3). The selectivity could be significantly improved when the IDPi core was modified from triflyl to perfluorophenylsulfonyl groups in catalyst **5b**; however, the yield was reduced to 17% (entry 4). Gratifyingly, both high selectivity and reactivity were observed when a simple methyl carbamate protecting group was utilized (entries 5–7). Finally, installment of sterically demanding as well as highly dispersive *tert*-butyl and methyl-2-adamantyl substituents in catalysts **6b** and **7b**, respectively, gave satisfactory selectivity in the reaction with phenylacetaldehyde as well as with hexanal (entries 8–10).

**Table 1:**
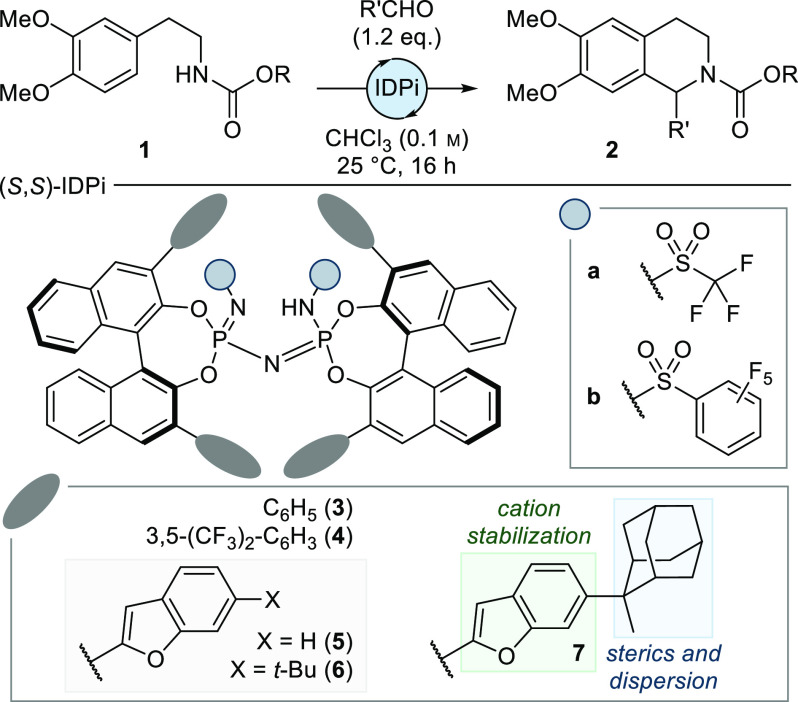
Reaction Development

entry^a^	IDPi	R	R′	yield^b^/%	er^c^
1	**3a**	Ph	Bn	5	55:45
2	**4a**	Ph	Bn	46	59:41
3	**5a**	Ph	Bn	60	70:30
4	**5b**	Ph	Bn	17	83:17
5	**5b**	Bn	Bn	62	87:13
6	**5b**	*t*-Bu	Bn	0	n.d.
7	**5b**	Me	Bn	68	95:5
8	**6b**	Me	Bn	60	96:4
9	**7b**	Me	Bn	69	97:3
10	**7b**	Me	*n*-pent	72	97.5:2.5

a Reactions were conducted with carbamate **1** (0.025 mmol), aldehyde (1.2 equiv), and (*S*,*S*)-IDPi catalyst (2 mol %) in CHCl_3_ (0.25 mL).

b Determined by ^1^H NMR of the crude reaction mixture using triphenylmethane as internal standard.

c Determined by HPLC.

With the optimal reaction conditions in hand, we turned our attention to the exploration of the substrate scope with the main objective being the synthesis of products that allow access to naturally occurring THIQs and other complex natural products (Figure 2). As benzylisoquinolines constitute the biggest class in this family of alkaloids, we tested several substituted phenylacetaldehydes toward THIQs **2a**–**g**. We were pleased to find that highly electron rich aromatic rings were well tolerated in the reaction. In addition to methylated phenols, silyloxy- and dioxolane-substituted substrates did not undergo any side reactions (protodesilylation was observed with catalytic Tf_2_NH). Instead, the products were formed in high yield and with excellent enantioselectivity. Brominated phenylacetaldehydes were also competent reaction partners to give THIQs **2h** and **2i**, the latter of which allowed for unambiguous determination of the absolute configuration by X-ray crystallography. Notably, the halide might serve as a valuable handle for the synthesis of complex bisbenzylisoquinoline natural products via established cross-coupling methodologies. Similarly high reactivity and enantioselectivity was observed in the formation of homobenzyl THIQs, in which the products were formed in up to quantitative yield and 94% enantiomeric excess (**2j**–**m**). Lastly, we were curious to test simple aliphatic aldehydes. In addition to long-chain substrate **2n**, branching in the β- and α-positions of the aldehyde was well tolerated, giving products **2o** and **2p**, respectively, in good yield with excellent enantioselectivity. Even the reaction with acetaldehyde—a notoriously challenging substrate due to its high reactivity paired with little steric demand—could be tamed to give **2q** in quantitative yield with exceptional enantioselectivity. Substrates bearing a benzyl-protected primary alcohol or a methyl ester were similarly well tolerated, yielding **2r** and **2s**. Lastly, after some fine-tuning of the reaction conditions (see the Supporting Information for details), *tert*-butyl-protected α-hydroxyacetaldehyde could be reacted to give **2t** in quantitative yield with good selectivity.

**Figure 2:**
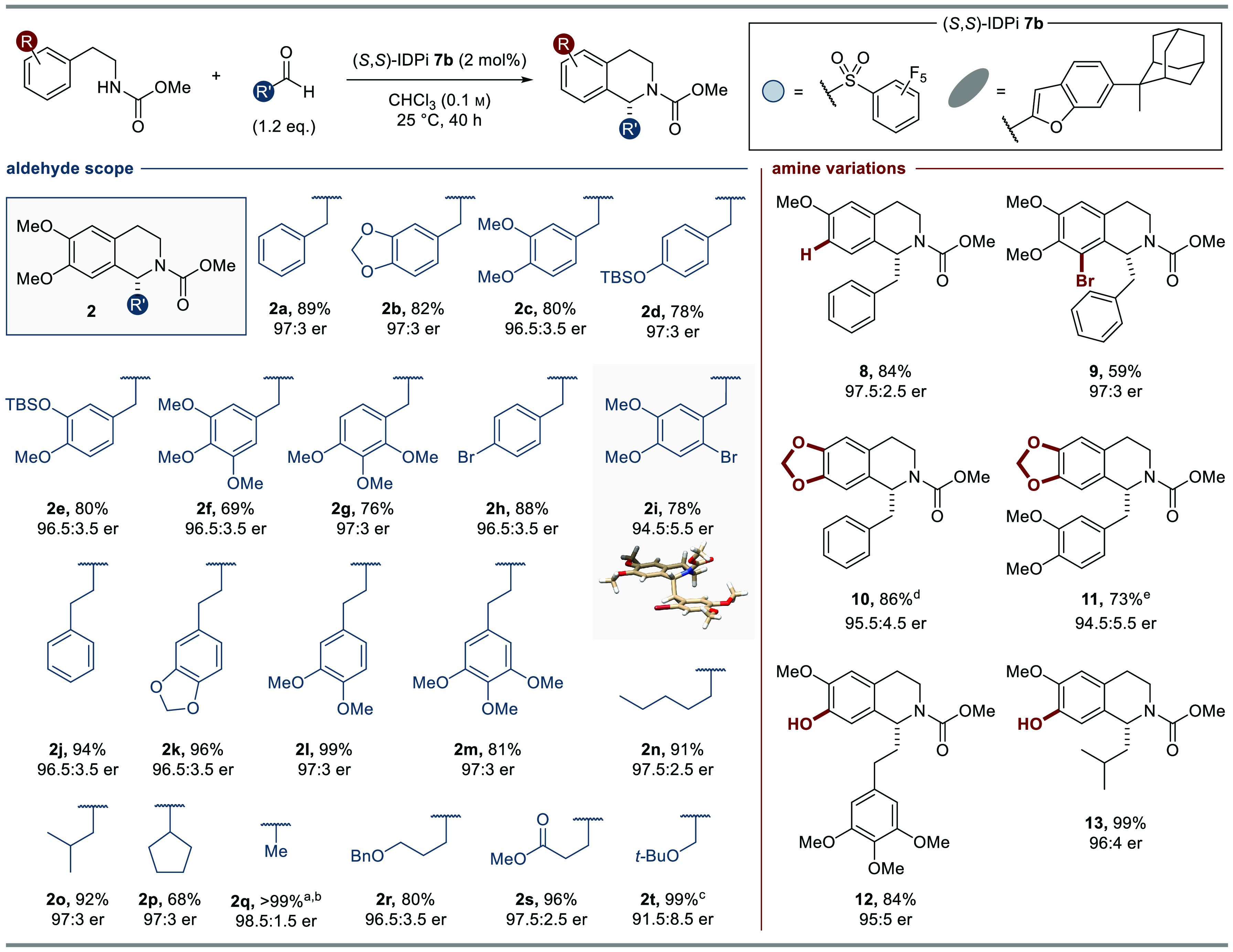
Substrate scope. All reactions were conducted on a 0.10 mmol scale. Yields are reported as isolated yields after column chromatography. ^*a*^Reaction was performed at −40 °C; ^*b*^3.0 equiv of aldehyde was used; ^*c*^Reaction was performed in *n*-pentane (0.025 M) instead of CHCl_3_; *^d^*Reaction was performed in CyH instead of CHCl_3_; ^*e*^Reaction was performed in CyH/CHCl_3_ (10:1, 0.05 M). See the Supporting Information for detailed reaction conditions. TBS = *tert*-butyldimethylsilyl.

Having established a scope of amenable aldehydes, we subsequently explored variations on the aromatic ring of the amine. High reactivity and selectivity were maintained after removal of a single methoxy substituent (**8**). On the other hand, an additional bromine in the 3-position was equally well tolerated by the optimal catalyst (**9**). When the substrate was altered to the dioxole annulation present in many natural products, satisfactory enantioinduction could be achieved by performing the reaction in nonpolar cyclcohexane, presumably due to diminished solvent interactions in the relevant transition states (**10** and **11**). Lastly, removal of a single methyl protecting group led to the efficient formation of products **12** and **13** with comparably high enantioselectivity. These results further demonstrate the importance of noncovalent interactions between the substrate and the catalyst that remain undisturbed by the introduction of a strong hydrogen bond donor.

We subsequently turned our attention to the synthesis of diverse members of the THIQ family of alkaloids (Figure 3A). As was mentioned before, full reduction of the carbamate functionality gives rise to *N*-methylated THIQs. Exemplary, the reaction of **2c**, **11**, and **13** enabled the synthesis of laudanosine, as well as the first asymmetric total syntheses of romneine and lophocerine. Furthermore, deprotection of the carbamate to the secondary amine could be achieved with trimethylsilyl iodide. Accordingly, calycotomine was accessible from **2t** in a single step. Carbamate removal from **2s** and subsequent treatment with base resulted in efficient cyclization toward the respective γ-lactam **14**, which is an established common intermediate in the synthesis of either oleracein E or crispine A. Deprotection of **2c** allowed the implementation of a subsequent Pictet–Spengler reaction with formaldehyde in the total synthesis of the tetrahydroberberine alkaloid xylopinine. The aporphine natural product glaucine is formally accessible either from brominated product **2i** via transition-metal catalysis or from laudanosine via oxidative arene coupling. The famous morphinan skeleton of amurine could be efficiently accessed from THIQ **15** via constant current electrolysis (CCE) conditions developed by Opatz and Waldvogel.^35^ Similarly, *O*-methylandrocymbine, a biosynthetic intermediate to colchicine, is formally accessible in a single step after reduction of Pictet–Spengler product **12**.

**Figure 3:**
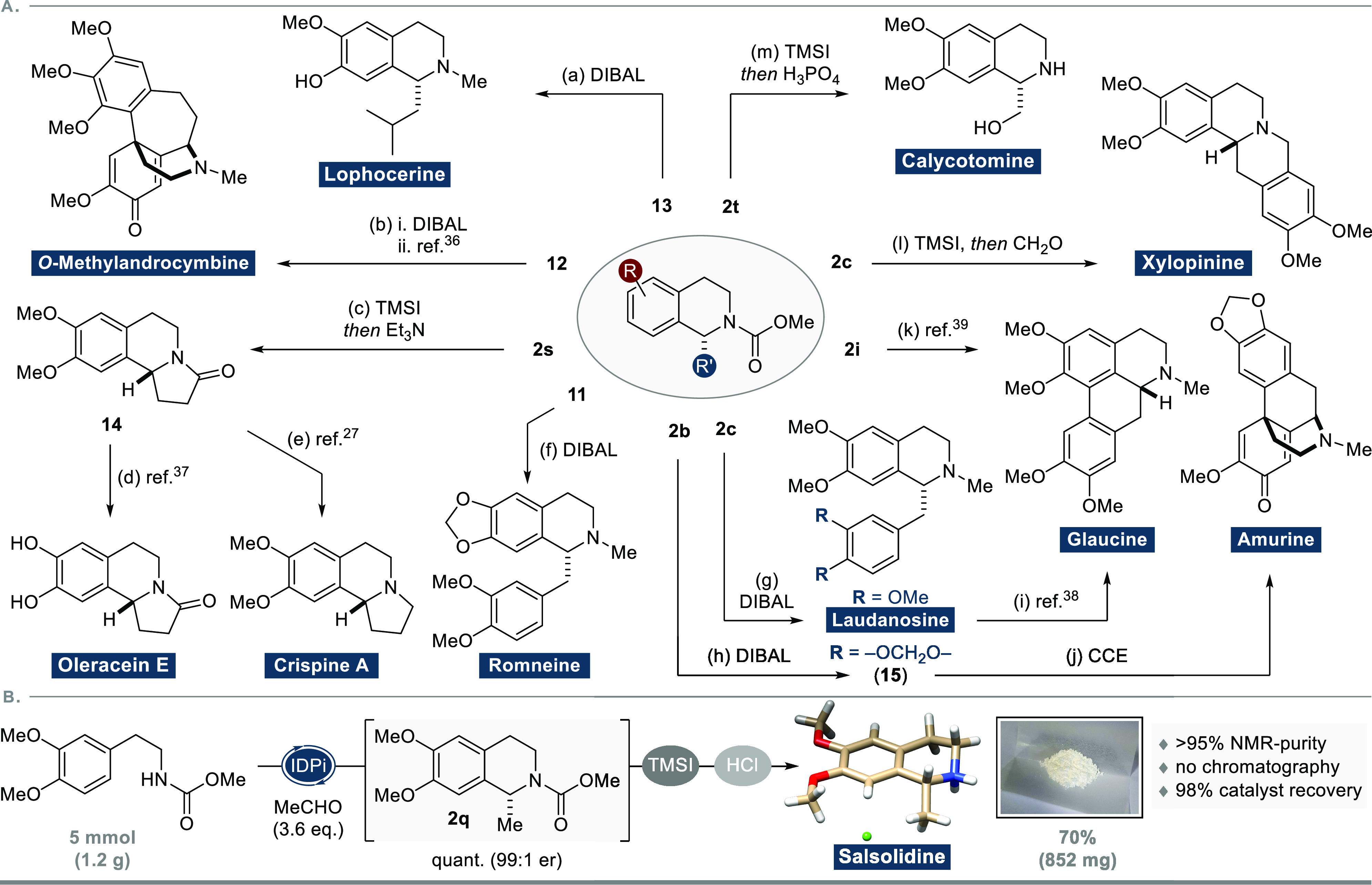
(A) Completed formal and total syntheses of naturally occurring alkaloids. (a) DIBAL, THF, RT, 95%; (b) i. DIBAL, THF, RT, 90%; ii. Nicolaou et al.;^36^ (c) TMSI, DCM, 0 °C to RT, *then* Et_3_N, PhMe, reflux, 52% (74% brsm); (d) Lin et al.;^37^ (e) Mons et al.;^27^ (f) DIBAL, THF, RT, >99%; (g) DIBAL, THF, RT, 94%; (h) DIBAL, THF, RT, 91%; (i) Anakabe et al.;^38^ (j) CCE, HBF_4_, CH_3_CN, 0 °C, 75%; (k) Pieper et al.;^39^ (l) TMSI, DCM, 0 °C to RT, *then* CH_2_O (aq), HCO_2_H, reflux, 76%; (m) TMSI, DCM, 0 °C to RT, *then* H_3_PO_4_ (aq), DCM, RT, 66%. (B) Gram-scale synthesis of salsolidine hydrochloride. TMS = trimethylsilyl. CCE = constant current electrolysis.

As a final test of our methodology, we attempted the direct synthesis of salsolidine from carbamate **1a** on a gram scale (Figure 3B). Gratifyingly, Pictet–Spengler product **2q** was formed quantitatively in near-perfect enantiopurity (99:1 er) with reduced catalyst loading (0.5 mol %). After deprotection of the crude product, simple acid–base extraction and subsequent treatment with HCl (in Et_2_O) was sufficient to precipitate the natural product as the hydrochloride salt without the necessity of any further purification steps, while simultaneously allowing almost quantitative (98%) catalyst recovery.

In conclusion, we have developed a highly enantioselective Pictet–Spengler reaction of *N*-carbamoyl-β-arylethylamines with diverse aldehydes. Our platform allows efficient access to THIQ, aporphine, tetrahydroberberine, morphinan, and androcymbine natural products, utilizing the Pictet–Spengler product as a strategic biomimetic diversification point. Key to the success of our strategy was the discovery of electron-rich IDPi catalysts that show a significantly enhanced reaction rate in comparison to more acidic catalysts while simultaneously furnishing the products with excellent stereoselectivity. The origin of this effect is currently under investigation in our laboratory.

## Supporting Information Available

Experimental details and analytical data for all new compounds (PDF)
