# Supplementary material for: A Catalytic Asymmetric Pictet–Spengler Platform as a Biomimetic Diversification Strategy toward Naturally Occurring Alkaloids
Source: J Am Chem Soc. 2022 Aug 17;144(34):15451–6. doi: 10.1021/jacs.2c06664 (PMC9446894; doi:10.1021/jacs.2c06664)
Supplement: Supplementary file 1 — ja2c06664_si_001.pdf [file ja2c06664_si_001.pdf]

A Catalytic Asymmetric Pictet–Spengler Platform as a Biomimetic Diversification Strategy toward Naturally Occurring Alkaloids

Manuel J. Scharf and Benjamin List*

Contents

1. General Information 2
2. Reaction Optimization 3
3. Asymmetric Pictet-Spengler Reactions 8
4. Synthesis of Natural Products 25
5. Synthesis of Protected *β*-arylethylamines 34
6. Synthesis of Aldehydes 38
7. Synthesis of IDPi Catalysts 46
8. NMR Spectra 55
9. HPLC Traces 145
10. Crystallographic Data 181

References 187

1. General Information

Unless otherwise stated, all reagents were purchased from commercial suppliers and used without further purification. All solvents used in the reactions were distilled from appropriate drying agents prior to use. Reactions were monitored by thin layer chromatography (TLC) on silica gel pre-coated glass (0.2 mm, Macherey-Nagel). Visualization was accomplished by irradiation with UV light at 254 nm and/or cerium ammonium molybdate (CAM) stain and/or $KMnO_4$ stain. Column chromatography was carried out using Merck (60 Å, 230–400 mesh, particle size 0.040–0.063 mm) or VWR (40–63 μm) silica gel, using technical grade solvents. Automated reversed phase column chromatography was conducted on a Biotage Isolera Spektra Four system, using SNAP Ultra C18 HP-Sphere 25 μm reversed phase cartridges. All reported yields refer to chromatographically and spectroscopically pure compounds. ^{1}H and ^{13}C NMR spectra were recorded on a Bruker AV-500 spectrometer in deuterated solvents. ^{1}H chemical shifts (δ) are reported in ppm relative to the protonated solvent resonance employed as the internal standard ($CDCl_3$ δ = 7.26, CD_2Cl_2 δ = 5.32, DMSO δ = 2.50, CD_3OD δ = 3.31 ppm). Data are reported as follows: chemical shift, multiplicity (s = singlet, d = doublet, t = triplet, q = quartet, p = pentet, s = sextet, h = heptet, m = multiplet, b = broad), coupling constants (Hz), and integration. ^{13}C chemical shifts are reported in ppm with the solvent resonance as the internal standard ($CDCl_3$ δ = 77.16, CD_2Cl_2 δ = 54.00, DMSO δ = 39.52, CD_3OD δ = 49.00 ppm). High resolution mass spectra were determined on a Bruker APEX III FTMS (7 T magnet). Optical rotations were determined with an Autopol IV polarimeter (Rudolph Research Analytical) at 589 nm and 25 °C. Data are reported as follows: α_{λ}^{T}, concentration *c* (g/100 mL), and solvent. Enantiomeric ratios (er) were determined by HPLC analysis employing a chiral stationary phase column specified in the individual experiment, by comparing the samples with the corresponding racemic mixtures. Electrochemical transformations were performed using an IKA ElectraSyn 2.0 System in an undivided cell with Electrodes purchased from IKA.

2. Reaction Optimization

An oven-dried GC vial equipped with a magnetic stir bar was charged with the catalyst (2 mol%) and placed under argon. Substrate (0.025 mmol) and aldehyde (1.2 eq.) were dissolved separately in the reaction solvent and sequentially added to the catalyst. The mixture was then stirred at the appropriate temperature for 16 h (for reactions at reduced *T*, the reaction was started at –78 °C and then warmed to the reaction temperature). The reaction was quenched by addition of Et_3N (10 µL) followed by addition of Ph_3CH as internal standard (1.0 M in PhMe, 25 µL, 1.0 eq.). $CDCl_3$ (0.5 mL) was added to the mixture, and 0.5 mL were analyzed by ^{1}H-NMR to determine the product yield. The remaining solution was purified by preparative thin layer chromatography to give the enantiomeric ratio after HPLC analysis.

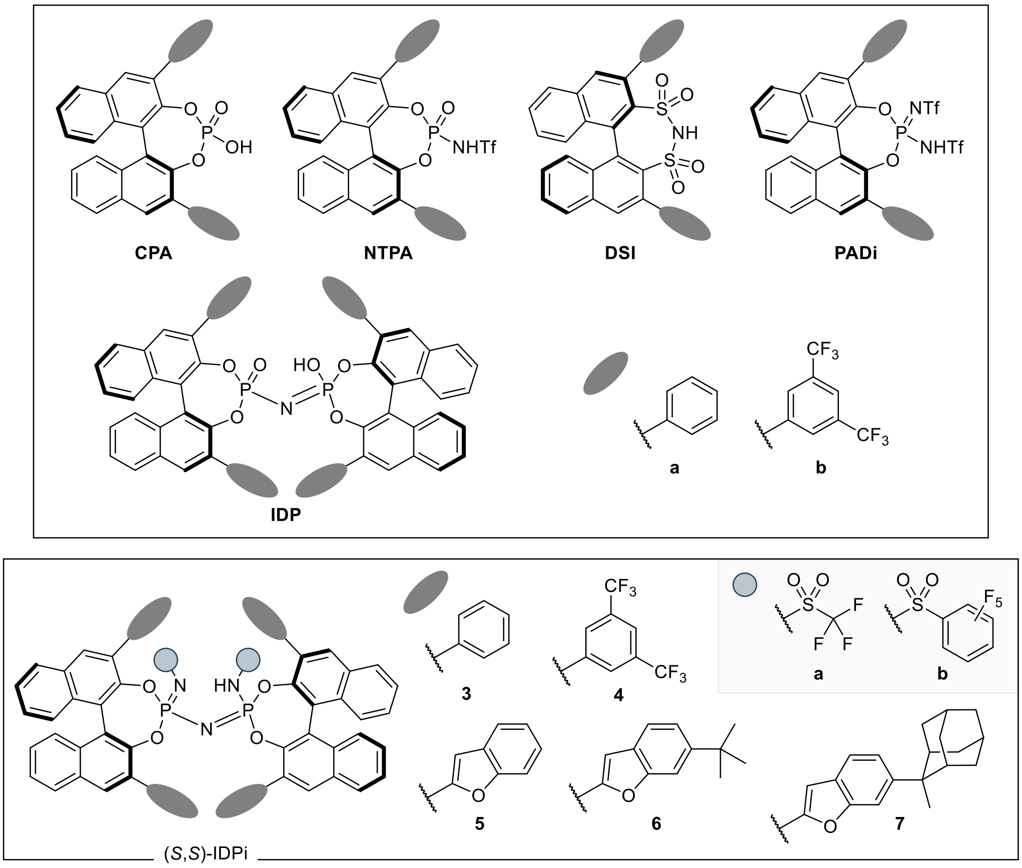

Figure S1: Catalysts used in the reaction optimization.

Table S1: Reaction Optimization.

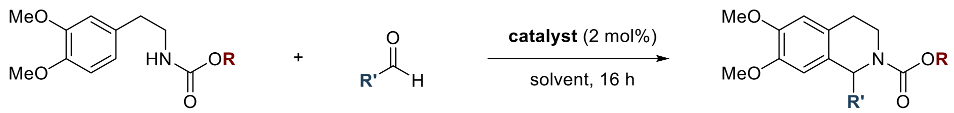

R	R'	catalyst	solvent (conc.)	temperature	yield	e.r.
Ph	Bn	**CPA-a**	$CHCl_3$ (0.1 M)	RT	0%	–
		**CPA-b**	$CHCl_3$ (0.1 M)	RT	0%	–
		**IDP-a**	$CHCl_3$ (0.1 M)	RT	0%	–
		**IDP-b**	$CHCl_3$ (0.1 M)	RT	0%	–
		**DSI-b**	$CHCl_3$ (0.1 M)	RT	0%	–
		**NTPA-a**	$CHCl_3$ (0.1 M)	RT	10%	50:50
		**PADi-b**	$CHCl_3$ (0.1 M)	RT	69%	53:47
		**3a**	$CHCl_3$ (0.1 M)	RT	5%	55:45
		**3a**	$CHCl_3$ (0.2 M)	RT	15%	57:43
		**3a**	$CHCl_3$ (0.4 M)	RT	28%	57:43
		**4a**	$CHCl_3$ (0.1 M)	RT	44%	59:41
		**5a**	$CHCl_3$ (0.1 M)	RT	60%	70:30
		**5a**	CH_2Cl_2 (0.1 M)	RT	69%	58:42
		**5a**	PhMe (0.1 M)	RT	53%	69:31
		**5a**	Et_2O (0.1 M)	RT	21%	33:67
		**5a**	MTBE (0.1 M)	RT	12%	71:29
		**5a**	CyH (0.1 M)	RT	11%	79:21
		**5a**	$CHCl_3$ (0.025 M)	RT	32%	72:28
		**5a**	$CHCl_3$ (0.05 M)	RT	53%	71:29
		**5a**	$CHCl_3$ (0.2 M)	RT	65%	69:31
		**5a**	$CHCl_3$ (0.4 M)	RT	64%	67:33
		**5a**	$CHCl_3$ (0.1 M)	10 °C	54%	70:30
		**5a**	$CHCl_3$ (0.1 M)	0 °C	34%	70:30
		**5a**	$CHCl_3$ (0.1 M)	–20 °C	19%	70:30
		**5b**	$CHCl_3$ (0.1 M)	RT	17%	83:17
t-Bu	Bn	**5b**	$CHCl_3$ (0.1 M)	RT	0%	–
Bn	Bn	**3a**	$CHCl_3$ (0.1 M)	RT	15%	51:49
		**4a**	$CHCl_3$ (0.1 M)	RT	77%	54:46
		**5a**	$CHCl_3$ (0.1 M)	RT	86%	67:33
		**5b**	$CHCl_3$ (0.1 M)	RT	62%	87:13
Me	Bn	**3a**	$CHCl_3$ (0.1 M)	RT	16%	51:49
		**4a**	$CHCl_3$ (0.1 M)	RT	74%	58:42
		**5a**	$CHCl_3$ (0.1 M)	RT	72%	65:35
		**5b**	$CHCl_3$ (0.1 M)	RT	68%	95:5
		**6b**	$CHCl_3$ (0.1 M)	RT	60%	96:4
		**7b**	$CHCl_3$ (0.1 M)	RT	69%	97:3
	n-pent	**6b**	$CHCl_3$ (0.1 M)	RT	76%	93.5:6.5
		**7b**	$CHCl_3$ (0.1 M)	RT	72%	97.5:2.5

Table S2: Reoptimization for 1,3-benzodioxole substrates.

	catalyst	solvent (conc.)	temperature	yield	e.r.
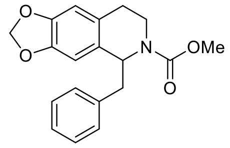	**5b**	$CHCl_3$ (0.1 M)	RT	43%	75:25
	**6b**	$CHCl_3$ (0.1 M)	RT	29%	83.5:16.5
	**7b**	$CHCl_3$ (0.1 M)	RT	53%	90.5:9.5
	**7b**	CH_2Cl_2 (0.1 M)	RT	51%	80:20
	**7b**	PhMe (0.1 M)	RT	44%	93.5:6.5
	**7b**	CyH (0.1 M)	RT	49%	95:5
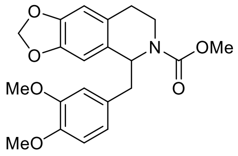	**7b**	$CHCl_3$ (0.1 M)	RT	48%	91.5:8.5
	**7b**	CyH (0.1 M)	RT	16%	94.5:5.5
	**7b**	CyH (0.05 M)	RT	31%	95:5
	**7b**	CyH/$CHCl_3$ (90:10, 0.05 M)	RT	49%	94.5:5.5
	**7b**	CyH/$CHCl_3$ (75:25, 0.05 M)	RT	48%	94:6
	**7b**	CyH/$CHCl_3$ (50:50, 0.05 M)	RT	22%	93:7

Table S3: Reoptimization for protected α-hydroxy-acetaldehydes.

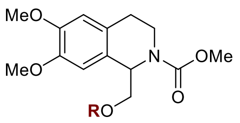

R	catalyst	solvent (conc.)	temperature	yield	e.r.
**TBS**	**5b**	$CHCl_3$ (0.1 M)	RT	43%	75:25
**Bn**	**5b**	$CHCl_3$ (0.1 M)	RT	95%	79:21
	**6b**	$CHCl_3$ (0.1 M)	RT	88%	82:18
	**5b**	$CHCl_3$ (0.1 M)	50 °C	88%	80:20
	**5b**	$CHCl_3$ (0.1 M)	0 °C	78%	79:21
	**5b**	$CHCl_3$ (0.1 M)	–20 °C	57%	76:24
	**5b**	$CHCl_3$ (0.1 M)	–40 °C	27%	69:31
	**5b**	$CHCl_3$ (0.05 M)	RT	75%	79:21
	**5b**	CyH (0.1 M)	RT	58%	61:39
	**5b**	PhMe (0.1 M)	RT	97%	77:23
**Ac**	**5b**	$CHCl_3$ (0.1 M)	RT	49%	87:13
	**6b**	$CHCl_3$ (0.1 M)	RT	30%	88.5:11.5
	**7b**	$CHCl_3$ (0.1 M)	RT	43%	87:13
	**6b**	CH_2Cl_2 (0.1 M)	RT	34%	84:16
	**6b**	PhMe (0.1 M)	RT	43%	87:13
	**6b**	CyH (0.1 M)	RT	46%	87:13
**Bz**	**5b**	$CHCl_3$ (0.1 M)	RT	69%	84.5:15.5
	**6b**	$CHCl_3$ (0.1 M)	RT	60%	85.5:14.5
	**7b**	$CHCl_3$ (0.1 M)	RT	59%	84.5:15.5
**Troc**	**5b**	$CHCl_3$ (0.1 M)	RT	35%	50:50
	**6b**	$CHCl_3$ (0.1 M)	RT	17%	50:50
	**7b**	$CHCl_3$ (0.1 M)	RT	20%	50:50
**TFA**[a]	**5b**	$CHCl_3$ (0.1 M)	RT	22%	55:45
	**6b**	$CHCl_3$ (0.1 M)	RT	13%	55:45
	**7b**	$CHCl_3$ (0.1 M)	RT	18%	56:44
***t*-Bu**	**5b**	$CHCl_3$ (0.1 M)	RT	76%	84.5:15.5
	**6b**	$CHCl_3$ (0.1 M)	RT	82%	86:14
	**7b**	$CHCl_3$ (0.1 M)	RT	82%	86.5:13.5
	**7b**	$CHCl_3$ (0.1 M)	0 °C	86%	85.5:14.5
	**7b**	CyH (0.1 M)	RT	90%	88:12
	**7b**	*n*-pent (0.1 M)	RT	62%	89:11
	**7b**	*n*-pent (0.05 M)	RT	75%	90.5:9.5
	**7b**	*n*-pent (0.025 M)	RT	98%	91.5:8.5
	**7b**	*n*-hex (0.025 M)	RT	63%	90.5:9.5
	**7b**	*n*-hept (0.025 M)	RT	64%	90:10
	**7b**	CyH (0.025 M)	RT	85%	89:11

[a]Isolated as free OH after preparative TLC.

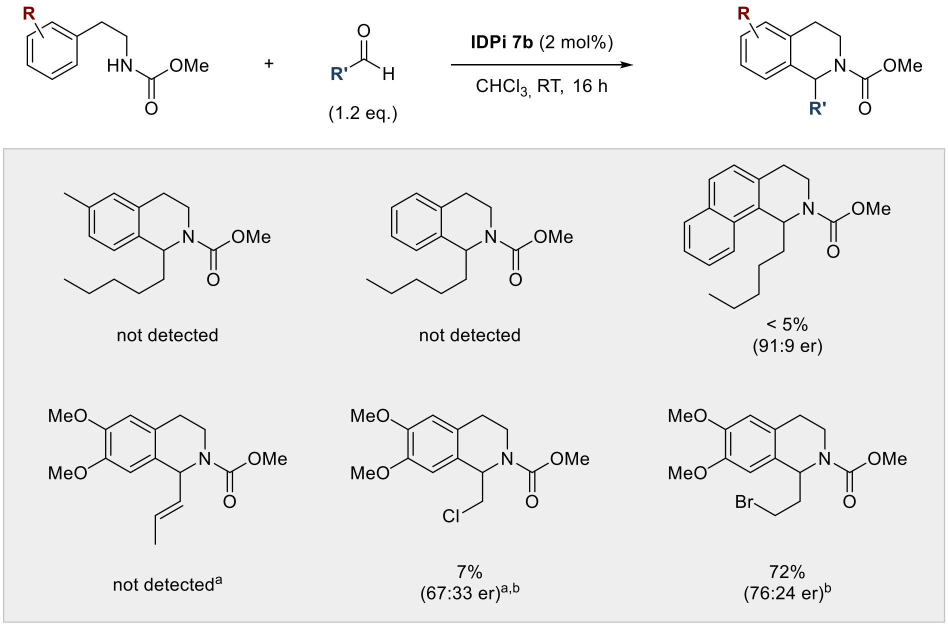

Scheme S1: Current scope limitations. [a]IDPi **5b** was used. [b]The dimethoxy acetal of the corresponding aldehyde was used.

3. Asymmetric Pictet-Spengler Reactions

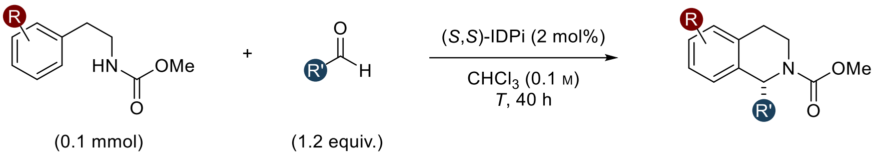

General procedure a

Carbamate (0.10 mmol) and (*S*,*S*)-IDPi catalyst **7b** (4.39 mg, 0.002 mmol, 2 mol%) were weighed into a septum-capped 4 mL vial equipped with a magnetic stir bar and placed under argon. The corresponding aldehyde (0.132 mmol, 1.32 eq.) was placed in an oven-dried GC-vial, argonated, and dissolved in dry $CHCl_3$ (1.1 mL). Of the thus prepared aldehyde stock solution, 1.0 mL (0.12 mmol, 1.2 eq.) were added to the substrate and catalyst under argon, the vial was sealed with Parafilm©, and the mixture was stirred at RT for 40 h. The reaction was quenched by addition of 5 drops of Et_3N and concentrated on silica. The product was isolated by silica gel flash column chromatography. **for deviations from the general procedure, see the corresponding entries.*

General procedure b

Carbamate (0.10 mmol) and (*S*,*S*)-IDPi catalyst **7b** (4.39 mg, 0.002 mmol, 2 mol%) were weighed into a septum-capped 4 mL vial equipped with a magnetic stir bar, placed under argon, and dissolved in dry $CHCl_3$ (1.0 mL). The corresponding aldehyde (0.12 mmol, 1.2 eq.) was subsequently added *via* Hamilton syringe, the vial was sealed with Parafilm©, and the mixture was stirred at RT for 40 h. The reaction was quenched by addition of 5 drops of Et_3N and concentrated on silica. The product was isolated by silica gel flash column chromatography. **for deviations from the general procedure, see the corresponding entries.*

Racemate synthesis

The corresponding racemates for determination of the enantiomeric access by HPLC-analysis were synthesized by reacting carbamate (0.1 mmol) with aldehyde (1.2 mmol) and catalytic amounts of Tf_2NH (7.5 mol%) in dry CH_3CN (1.0 mL) for 16 h. For acid sensitive TBS-protected products **2d** and **2e**, $CHCl_3$ was used instead of CH_3CN.

methyl (*R*)-1-benzyl-6,7-dimethoxy-3,4-dihydroisoquinoline-2(1*H*)-carboxylate (2a):

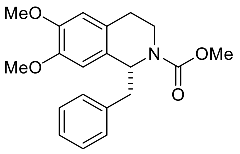

The reaction was performed according to **general procedure b** with carbamate **1a** (23.9 mg, 0.10 mmol, 1.00 eq.) and phenylacetaldehyde (14 µL, 0.12 mmol, 1.2 eq.). Purification by silica gel flash column chromatography (hex/EtOAc 2:1) afforded the product as a colorless oil (30.4 mg, 89 µmol, 89%).

$\boldsymbol{R_F}$ (hex/EtOAc 2:1) = 0.38.

^{1}H-NMR (501 MHz, $CDCl_3$): Mixture of two rotamers with a ratio ≈ 55:45. δ = 7.30–7.16 (m, $3H_{all}$), 7.13–7.06 (m, $2H_{all}$), 6.60 (s, $1H_{maj}$), 6.57 (s, $1H_{min}$), 6.26 (s, $1H_{maj}$), 6.09 (s, $1H_{min}$), 5.26 (dd, J = 8.1, 5.7 Hz, $1H_{min}$), 5.15 (t, J = 7.0 Hz, $1H_{maj}$), 4.17 (ddd, J = 13.1, 5.9, 3.6 Hz, $1H_{maj}$), 3.88–3.80 (m, $3H_{all}$ + $1H_{min}$), 3.71 (s, $3H_{min}$), 3.67 (s, $3H_{maj}$), 3.56 (s, $3H_{min}$), 3.49 (s, $3H_{maj}$), 3.42–3.29 (m, $1H_{all}$), 3.18 (dd, J = 13.1, 5.6 Hz, H_{min}), 3.09 (dd, J = 13.4, 7.4 Hz, $1H_{maj}$), 2.97 (ddd, J = 13.0, 9.9, 7.3 Hz, $1H_{all}$), 2.86

(ddd, J = 16.3, 10.5, 5.9 Hz, $1H_{maj}$), 2.77 (ddd, J = 15.2, 9.2, 5.6 Hz, $1H_{min}$), 2.61 (ddt, J = 26.4, 15.8, 4.5 Hz, $1H_{all}$).

^{13}C-NMR (126 MHz, $CDCl_3$): δ = 156.12, 156.07, 147.91, 147.73, 147.09, 146.86, 138.39, 130.03, 129.81, 128.35, 128.30, 128.23, 126.54, 126.47, 126.40, 126.15, 111.48, 111.14, 110.73, 110.35, 56.40, 56.37, 55.97, 55.95, 55.87, 55.71, 52.69, 52.49, 43.22, 42.82, 39.34, 38.18, 28.23, 28.13.

CI-HRMS: calculated for $C_{20}H_{24}N_1O_4$ ($[M+H]^+$): 342.169983, found: 342.170590.

HPLC (OD-3, *n*-heptane/*i*-PrOH 95:5, 298 K, 283 nm): t_R (minor) = 9.4 min, t_R (major) = 10.7 min, er = 97:3 (94% ee).

$[\alpha]_D^{25}$ = −69.6 (*c* = 0.23, $CHCl_3$).

methyl (*R*)-1-(benzo[*d*][1,3]dioxol-5-ylmethyl)-6,7-dimethoxy-3,4-dihydroisoquinoline-2(1*H*)-carboxylate (2b):

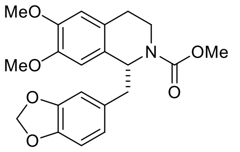

The reaction was performed according to **general procedure a** with carbamate **1a** (23.9 mg, 0.10 mmol, 1.00 eq.) and 2-(benzo[*d*][1,3]dioxol-5-yl)acetaldehyde (**S9**, 21.7 mg, 0.132 mmol, 1.32 eq.). Purification by silica gel flash column chromatography (DCM/EtOAc 19:1 to 9:1) afforded the product as a white foam (31.5 mg, 82 μmol, 82%).

***R*$_F$** (DCM/EtOAc 9:1) = 0.62.

^{1}H-NMR (501 MHz, $CDCl_3$): Mixture of two rotamers with a ratio ≈ 55:45. δ = 6.71 (d, J = 7.9 Hz, $1H_{maj}$), 6.68 (d, J = 7.9 Hz, $1H_{min}$), 6.63–6.56 (m, $2H_{all}$), 6.55–6.48 (m, $1H_{all}$), 6.31 (s, $1H_{maj}$), 6.22 (s, $1H_{min}$), 5.94–5.86 (m, $2H_{all}$), 5.20 (t, J = 6.7 Hz, $1H_{min}$), 5.10 (t, J = 6.9 Hz, $1H_{maj}$), 4.14 (ddd, J = 13.2, 6.0, 3.7 Hz, $1H_{maj}$), 3.85 (s, $3H_{maj}$), 3.86–3.79 (m, $3H_{min}$ + $1H_{min}$), 3.72 (s, $3H_{maj}$), 3.71 (s, $3H_{min}$), 3.66 (s, $3H_{min}$), 3.57 (s, $3H_{maj}$), 3.34 (ddd, J = 13.4, 9.2, 4.6 Hz, $1H_{min}$), 3.27 (ddd, J = 13.2, 10.5, 4.4 Hz, $1H_{maj}$), 3.07 (dd, J = 13.4, 5.8 Hz, $1H_{min}$), 3.00 (dd, J = 13.6, 7.3 Hz, $1H_{maj}$), 2.94–2.81 (m, $1H_{all}$ + $1H_{maj}$), 2.76 (ddd, J = 15.2, 9.2, 5.6 Hz, $1H_{min}$), 2.60 (ddt, J = 25.7, 15.8, 4.5 Hz, $1H_{all}$).

^{13}C-NMR (126 MHz, $CDCl_3$): δ = 156.14, 156.08, 147.96, 147.81, 147.58, 147.52, 147.15, 147.02, 146.22, 146.21, 132.14, 128.37, 128.18, 126.47, 126.26, 122.92, 122.83, 111.54, 111.22, 110.73, 110.36, 110.12, 108.16, 108.11, 100.93, 100.88, 56.43, 56.34, 55.98, 55.88, 52.72, 52.60, 42.94, 42.48, 39.33, 38.27, 28.27, 28.10.

ESI-HRMS: calculated for $C_{21}H_{23}N_1O_6Na_1$ ($[M+Na]^+$): 408.141758, found: 408.141780.

HPLC (OD-3, *n*-heptane/*i*-PrOH 90:10, 298 K, 287 nm): t_R (minor) = 9.0 min, t_R (major) = 11.0 min, er = 97:3 (94% ee).

$[\alpha]_D^{25}$ = −72.9 (*c* = 0.13, $CHCl_3$).

methyl (*R*)-1-(3,4-dimethoxybenzyl)-6,7-dimethoxy-3,4-dihydroisoquinoline-2(1*H*)-carboxylate (2c):

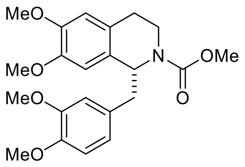

The reaction was performed according to **general procedure a** with carbamate **1a** (23.9 mg, 0.10 mmol, 1.00 eq.) and 2-(3,4-dimethoxyphenyl)acetaldehyde (**S10**, 23.8 mg, 0.132 mmol, 1.32 eq.). Purification by silica gel flash column chromatography (hex/EtOAc 1:1) afforded the product as a colorless oil (32.0 mg, 80 µmol, 80%).

R_F (hex/EtOAc 1:1) = 0.30.

^{1}H-NMR (501 MHz, $CDCl_3$): Mixture of two rotamers with a ratio ≈ 1:1. δ = 6.75 (dd, J = 19.0, 8.1 Hz, $1H_{all}$), 6.66–6.53 (m, $3H_{all}$), 6.34 (s, 1H), 6.18 (s, 1H), 5.23 (dd, J = 7.9, 5.4 Hz, 1H), 5.12 (t, J = 6.9 Hz, 1H), 4.13 (ddd, J = 13.3, 6.0, 3.8 Hz, 1H), 3.84 (s, $3H_{all}$), 3.83 (s, $3H_{all}$), 3.80 (s, 3H), 3.77 (s, 3H), 3.72 (s, $3H_{all}$), 3.62 (s, 3H), 3.54 (s, 3H), 3.32 (ddd, J = 13.3, 9.1, 4.7 Hz, 1H), 3.24 (ddd, J = 13.1, 10.5, 4.4 Hz, 1H), 3.10 (dd, J = 13.3, 5.4 Hz, 1H), 3.02 (dd, J = 13.6, 7.1 Hz, 1H), 2.94 (dd, J = 13.5, 7.2 Hz, $1H_{all}$), 2.83 (ddd, J = 16.2, 10.4, 5.9 Hz, 1H), 2.74 (ddd, J = 15.3, 9.0, 5.6 Hz, 1H), 2.55 (ddt, J = 25.7, 15.8, 4.6 Hz, $1H_{all}$).

^{13}C-NMR (126 MHz, $CDCl_3$): δ = 156.17, 156.11, 148.78, 148.72, 147.93, 147.84, 147.76, 147.74, 147.17, 146.94, 130.86, 128.41, 128.26, 126.57, 126.33, 122.12, 121.98, 112.97, 111.51, 111.17, 111.02, 110.80, 110.39, 56.27, 56.05, 55.98, 55.90, 55.85, 52.70, 52.60, 42.76, 42.31, 39.41, 38.27, 28.21, 28.09.

ESI-HRMS: calculated for $C_{22}H_{27}N_1O_6Na_1$ ($[M+Na]^+$): 424.173058, found: 424.173050.

HPLC (OD-3, *n*-heptane/*i*-PrOH 90:10, 298 K, 282 nm): t_R (minor) = 13.9 min, t_R (major) = 15.8 min, er = 96.5:3.5 (93% ee).

$[\alpha]_D^{25}$ = −51.7 (*c* = 0.14, $CHCl_3$).

methyl (*R*)-1-(4-((tert-butyldimethylsilyl)oxy)benzyl)-6,7-dimethoxy-3,4-dihydroisoquinoline-2(1*H*)-carboxylate (2d):

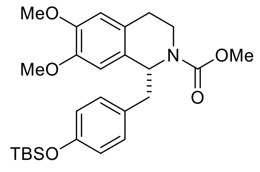

The reaction was performed according to **general procedure a** with carbamate **1a** (23.9 mg, 0.10 mmol, 1.00 eq.) and 2-(4-((*tert*-butyldimethylsilyl)oxy)phenyl)acetaldehyde (**S13**, 33.1 mg, 0.132 mmol, 1.32 eq.). Purification by silica gel flash column chromatography (hex/EtOAc 2:1) afforded the product as a colorless oil (36.7 mg, 78 µmol, 78%).

R_F (hex/EtOAc 2:1) = 0.64.

^{1}H-NMR (501 MHz, $CDCl_3$): Mixture of two rotamers with a ratio ≈ 55:45. δ = 6.93 (dd, J = 12.1, 8.0 Hz, $2H_{all}$), 6.72 (dd, J = 16.0, 8.0 Hz, $2H_{all}$), 6.59 (s, $1H_{maj}$), 6.57 (s, $1H_{min}$), 6.32 (s, $1H_{maj}$), 6.19 (s, $1H_{min}$), 5.21 (dd, J = 7.9, 5.4 Hz, $1H_{min}$), 5.10 (t, J = 6.8 Hz, $1H_{maj}$), 4.14 (ddd, J = 13.2, 5.9, 3.7 Hz, $1H_{maj}$), 3.87–3.76 (m, $3H_{all}$ + $1H_{min}$), 3.71 (d, J = 2.1 Hz, $3H_{all}$), 3.63 (s, $3H_{min}$), 3.53 (s, $3H_{maj}$), 3.38–3.25 (m, $1H_{all}$), 3.08 (dd, J = 13.3, 5.4 Hz, $1H_{min}$), 3.01 (dd, J = 13.6, 7.3 Hz, $1H_{maj}$), 2.96–2.88 (m, $1H_{all}$), 2.84 (ddd, J = 16.3, 10.5, 5.9 Hz, $1H_{maj}$), 2.74 (ddd, J = 15.1, 9.1, 5.6 Hz, $1H_{min}$), 2.61 (dt, J =

16.0, 4.1 Hz, $1H_{maj}$), 2.54 (dt, J = 15.8, 5.0 Hz, $1H_{min}$), 0.97 (s, $9H_{maj}$), 0.97 (s, $9H_{min}$), 0.17 (s, $6H_{maj}$), 0.16 (s, $6H_{min}$).

^{13}C-NMR (126 MHz, $CDCl_3$): δ = 156.11, 154.42, 154.37, 147.89, 147.71, 147.13, 146.93, 131.09, 131.06, 130.93, 130.72, 128.54, 128.41, 126.47, 126.25, 119.94, 119.87, 111.49, 111.15, 110.76, 110.38, 56.45, 55.98, 55.95, 55.82, 52.67, 52.55, 42.47, 42.08, 39.40, 38.24, 28.25, 28.14, 25.82, 18.34, -4.30.

ESI-HRMS: calculated for $C_{26}H_{37}N_1Na_1O_5Si_1$ ($[M+Na]^+$): 494.23332, found: 494.23359.

HPLC (OD-3, *n*-heptane/*i*-PrOH 90:10, 298 K, 282 nm): t_R (minor) = 5.6 min, t_R (major) = 6.8 min, er = 97:3 (94% ee).

$[\alpha]_D^{25}$ = −59.4 (*c* = 0.36, $CHCl_3$).

methyl (*R*)-1-(3-((tert-butyldimethylsilyl)oxy)-4-methoxybenzyl)-6,7-dimethoxy-3,4-dihydroisoquinoline-2(1*H*)-carboxylate (2e):

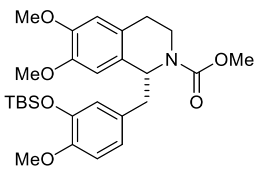

The reaction was performed according to **general procedure a** with carbamate **1a** (23.9 mg, 0.10 mmol, 1.00 eq.) and 2-(4-((*tert*-butyldimethylsilyl)oxy)-3-methoxyphenyl)acetaldehyde (**S15**, 37.0 mg, 0.132 mmol, 1.32 eq.). Purification by silica gel flash column chromatography (hex/EtOAc 2:1) afforded the product as a colorless oil (40.1 mg, 80 µmol, 80%).

***R*$_F$** (hex/EtOAc 2:1) = 0.42.

^{1}H-NMR (501 MHz, $CDCl_3$): Mixture of two rotamers with a ratio ≈ 55:45. δ = 6.73 (d, J = 8.2 Hz, $1H_{maj}$), 6.70 (d, J = 8.6 Hz, $1H_{min}$), 6.63 (d, J = 2.2 Hz, $1H_{maj}$), 6.60–6.53 (m, $2H_{all}$ + $1H_{min}$), 6.29 (s, $1H_{maj}$), 6.26 (s, $1H_{min}$), 5.22 (dd, J = 7.6, 5.4 Hz, $1H_{min}$), 5.10 (t, J = 6.7 Hz, $1H_{maj}$), 4.11 (ddd, J = 13.3, 5.8, 3.5 Hz, $1H_{maj}$), 3.89–3.78 (m, $3H_{all}$ + $1H_{min}$), 3.76 (s, $3H_{maj}$), 3.75 (s, $3H_{min}$), 3.71 (s, $3H_{min}$), 3.70 (s, $3H_{maj}$), 3.66 (s, $3H_{min}$), 3.59 (s, $3H_{maj}$), 3.22 (ddt, J = 12.9, 10.2, 3.2 Hz, $1H_{all}$), 3.08–2.80 (m, $2H_{all}$ + $1H_{maj}$), 2.72 (ddd, J = 15.3, 9.3, 5.6 Hz, $1H_{min}$), 2.60 (dt, J = 15.9, 4.1 Hz, $1H_{maj}$), 2.50 (dt, J = 15.8, 4.8 Hz, $1H_{min}$), 0.98 (s, $9H_{maj}$), 0.96 (s, $9H_{min}$), 0.13 (s, $3H_{maj}$), 0.11 (s, $3H_{maj}$), 0.09 (s, $3H_{min}$), 0.09 (s, $3H_{min}$).

^{13}C-NMR (126 MHz, $CDCl_3$): δ = 156.10, 149.83, 149.78, 147.88, 147.73, 147.12, 147.06, 144.97, 144.78, 130.95, 130.82, 128.46, 128.36, 126.46, 126.37, 123.20, 123.10, 122.66, 122.34, 111.99, 111.49, 111.16, 110.70, 110.37, 56.32, 56.20, 55.94, 55.90, 55.72, 55.68, 52.63, 42.49, 41.70, 39.40, 38.41, 28.26, 28.12, 25.84, 18.55, 18.53, -4.55, -4.61, -4.64.

ESI-HRMS: calculated for $C_{27}H_{39}N_1Na_1O_6Si_1$ ($[M+Na]^+$): 524.24389, found: 524.24435.

HPLC (OD-3, *n*-heptane/*i*-PrOH 97:3, 298 K, 282 nm): t_R (minor) = 8.2 min, t_R (major) = 9.6 min, er = 96.5:3.5 (93% ee).

$[\alpha]_D^{25}$ = −44.8 (*c* = 0.22, $CHCl_3$).

methyl (*R*)-6,7-dimethoxy-1-(3,4,5-trimethoxybenzyl)-3,4-dihydroisoquinoline-2(1*H*)-carboxylate (2f):

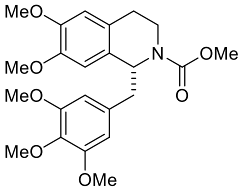

The reaction was performed according to **general procedure a** with carbamate **1a** (23.9 mg, 0.10 mmol, 1.00 eq.) and 2-(3,4,5-trimethoxyphenyl)acetaldehyde (**S17**, 27.7 mg, 0.132 mmol, 1.32 eq.). Purification by silica gel flash column chromatography (hex/EtOAc 1:1) afforded the product as a colorless oil (29.8 mg, 69 µmol, 69%).

$\boldsymbol{R}_{\mathbf{F}}$ (hex/EtOAc 1:1) = 0.21.

^{1}H-NMR (501 MHz, $CDCl_3$): Mixture of two rotamers with a ratio ≈ 55:45. δ = 6.60 (s, $1H_{min}$), 6.59 (s, $1H_{maj}$), 6.33 (s, $1H_{min}$), 6.29 (s, $2H_{min}$), 6.27 (s, $2H_{maj}$), 6.14 (s, $1H_{maj}$), 5.24 (dd, J = 8.3, 5.2 Hz, $1H_{maj}$), 5.13 (t, J = 6.8 Hz, $1H_{min}$), 4.19–4.13 (m, $1H_{min}$), 3.88–3.82 (m, $3H_{all}$ + $1H_{maj}$), 3.83–3.77 (m, $6H_{all}$), 3.75 (s, $3H_{all}$), 3.73 (s, $3H_{min}$), 3.72 (s, $3H_{maj}$), 3.61 (s, $3H_{maj}$), 3.56 (s, $3H_{min}$), 3.36 (ddd, J = 13.2, 8.9, 4.6 Hz, $1H_{maj}$), 3.27 (ddd, J = 13.4, 10.7, 4.4 Hz, $1H_{min}$), 3.12 (dd, J = 13.2, 5.2 Hz, $1H_{maj}$), 3.02 (dd, J = 13.5, 7.1 Hz, $1H_{min}$), 2.95–2.82 (m, $1H_{all}$ + $1H_{min}$), 2.77 (ddd, J = 15.1, 8.9, 5.5 Hz, $1H_{maj}$), 2.60 (ddd, J = 19.6, 11.8, 7.4 Hz, $1H_{all}$).

^{13}C-NMR (126 MHz, $CDCl_3$): δ = 156.19, 156.07, 153.12, 153.04, 148.01, 147.84, 147.19, 146.93, 136.85, 136.70, 134.08, 134.00, 128.27, 128.17, 126.55, 126.32, 111.56, 111.22, 110.91, 110.44, 106.89, 106.82, 60.97, 56.28, 56.25, 56.20, 56.03, 56.01, 55.83, 52.74, 52.64, 43.54, 43.10, 39.50, 38.26, 28.23, 28.10.

ESI-HRMS: calculated for $C_{23}H_{29}N_1O_7Na_1$ ($[M+Na]^+$): 454.183623, found: 454.183800.

HPLC (OD-3, *n*-heptane/*i*-PrOH 90:10, 298 K, 282 nm): t_R (minor) = 12.7 min, t_R (major) = 15.2 min, er = 96.5:3.5 (93% ee).

$[\alpha]_D^{25}$ = −54.4 (*c* = 0.21, $CHCl_3$).

methyl (*R*)-6,7-dimethoxy-1-(2,3,4-trimethoxybenzyl)-3,4-dihydroisoquinoline-2(1*H*)-carboxylate (2g):

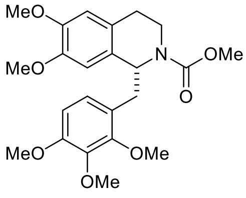

The reaction was performed according to **general procedure a** with carbamate **1a** (23.9 mg, 0.10 mmol, 1.00 eq.) and 2-(2,3,4-trimethoxyphenyl)acetaldehyde (**S19**, 22.7 mg, 0.132 mmol, 1.32 eq.). Purification by silica gel flash column chromatography (DCM/EtOAc 19:1 to 4:1) afforded the product as a colorless oil (33.0 mg, 76 µmol, 76%).

$\boldsymbol{R}_{\mathbf{F}}$ (DCM/EtOAc 9:1) = 0.31.

^{1}H-NMR (501 MHz, $CDCl_3$): Mixture of two rotamers with a ratio ≈ 65:35. δ = 6.76 (d, J = 8.5 Hz, $1H_{min}$), 6.65 (d, J = 8.5 Hz, $1H_{maj}$), 6.60 (s, $1H_{maj}$), 6.58–6.54 (m, $1H_{all}$ + $1H_{min}$), 6.46 (s, $1H_{maj}$), 6.25 (s, $1H_{min}$), 5.31 (t, J = 7.3 Hz, $1H_{min}$), 5.19 (dd, J = 9.1, 5.1 Hz, $1H_{maj}$), 4.26 (ddd, J = 13.2, 6.1, 2.8 Hz, $1H_{maj}$), 3.99–3.89 (m, $3H_{maj}$ + $1H_{min}$), 3.87 (s, $3H_{maj}$), 3.84 (s, $3H_{maj}$), 3.84–3.81 (m, 5.10H), 3.77 (s, $3H_{min}$), 3.76 (s, $3H_{maj}$), 3.65 (s, $3H_{min}$), 3.64 (s, $3H_{min}$), 3.45 (ddd, J = 13.7, 9.9, 4.6 Hz, $1H_{min}$), 3.41–3.31 (m, $3H_{maj}$ + $1H_{maj}$), 3.09–2.98 (m, $1H_{all}$ + $1H_{min}$), 2.93–2.76 (m, $1H_{all}$ + $1H_{maj}$), 2.66 (ddt, J = 15.9, 8.8, 4.1 Hz, $1H_{all}$).

^{13}**C-NMR** (126 MHz, $CDCl_3$): δ = 156.12, 156.04, 152.75, 152.59, 152.27, 147.88, 147.71, 147.26, 146.98, 142.17, 142.14, 129.08, 128.89, 126.31, 126.15, 125.31, 124.60, 124.41, 111.49, 111.26, 110.67, 110.30, 106.99, 106.93, 60.87, 60.84, 60.74, 56.15, 56.12, 55.99, 55.93, 55.79, 55.46, 55.27, 52.60, 52.33, 38.77, 37.52, 37.15, 35.94, 28.24, 28.20.

ESI-HRMS: calculated for $C_{23}H_{29}N_1Na_1O_7$ ($[M+Na]^+$): 454.18362, found: 454.18378.

HPLC (OD-3, *n*-heptane/*i*-PrOH 95:5, 298 K, 282 nm): t_R (minor) = 16.6 min, t_R (major) = 20.0 min, er = 97:3 (94% ee).

$[\alpha]_D^{25}$ = −69.7 (*c* = 0.37, $CHCl_3$).

methyl (*R*)-1-(4-bromobenzyl)-6,7-dimethoxy-3,4-dihydroisoquinoline-2(1*H*)-carboxylate (2h):

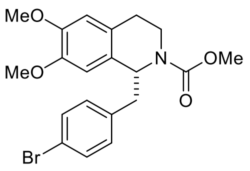

The reaction was performed according to **general procedure a** with carbamate **1a** (23.9 mg, 0.10 mmol, 1.00 eq.) and 2-(4-bromophenyl)acetaldehyde (26.3 mg, 0.132 mmol, 1.32 eq.). Purification by silica gel flash column chromatography (hex/EtOAc 2:1) afforded the product as a colorless oil (36.9 mg, 88 µmol, 88%).

R_F (hex/EtOAc 2:1) = 0.36.

^{1}**H-NMR** (501 MHz, $CDCl_3$): Mixture of two rotamers with a ratio ≈ 1:1. δ = 7.37 (dd, J = 17.0, 7.9 Hz, $2H_{all}$), 6.95 (dd, J = 10.2, 7.9 Hz, $2H_{all}$), 6.60 (s, 1H), 6.57 (s, 1H), 6.30 (s, 1H), 6.15 (s, 1H), 5.22 (t, J = 6.7 Hz, 1H), 5.12 (t, J = 6.9 Hz, 1H), 4.14 (ddd, J = 13.2, 5.9, 3.7 Hz, 1H), 3.85 (s, 3H), 3.84 (s, 3H), 3.79 (dt, J = 12.8, 5.5 Hz, 1H), 3.72 (s, 3H), 3.70 (s, 3H), 3.64 (s, 3H), 3.50 (s, 3H), 3.34 (ddd, J = 13.2, 8.9, 4.7 Hz, 1H), 3.27 (ddd, J = 13.0, 10.4, 4.4 Hz, 1H), 3.11 (dd, J = 13.3, 5.9 Hz, 1H), 3.03 (dd, J = 13.5, 7.7 Hz, 1H), 2.95 (dt, J = 12.6, 6.4 Hz, $1H_{all}$), 2.84 (ddd, J = 16.2, 10.4, 5.9 Hz, 1H), 2.75 (ddd, J = 15.0, 8.9, 5.5 Hz, 1H), 2.58 (ddt, J = 29.0, 15.8, 4.7 Hz, $1H_{all}$).

^{13}**C-NMR** (126 MHz, $CDCl_3$): δ = 156.18, 155.98, 148.05, 147.88, 147.25, 147.06, 137.34, 131.71, 131.53, 131.38, 131.33, 128.00, 127.85, 126.54, 126.32, 120.47, 120.43, 111.56, 111.23, 110.54, 110.20, 56.20, 56.12, 55.98, 55.96, 55.82, 52.77, 52.55, 42.55, 42.19, 39.44, 38.26, 28.18, 28.06.

ESI-HRMS: calculated for $C_{20}H_{22}Br_1N_1Na_1O_4$ ($[M+Na]^+$): 442.06244, found: 442.06243.

HPLC (OD-3, *n*-heptane/*i*-PrOH 90:10, 298 K, 283 nm): t_R (minor) = 6.5 min, t_R (major) = 8.1 min, er = 96.5:3.5 (93% ee).

$[\alpha]_D^{25}$ = −59.7 (*c* = 0.21, $CHCl_3$).

methyl (*R*)-1-(2-bromo-4,5-dimethoxybenzyl)-6,7-dimethoxy-3,4-dihydroisoquinoline-2(1*H*)-carboxylate (2i):

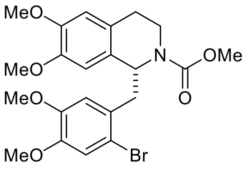

The reaction was performed according to **general procedure a** with carbamate **1a** (23.9 mg, 0.10 mmol, 1.00 eq.) and 2-(2-bromo-4,5-dimethoxyphenyl)acetaldehyde (**S21**, 34.2 mg, 0.132 mmol, 1.32 eq.). Purification by silica gel flash column chromatography (hex/MTBE 1:3) and

another silica gel flash column chromatography (hex/EtOAc 1:1) afforded the product as a white solid (37.4 mg, 78 µmol, 78%).

A crystalline sample for x-ray single crystal structure analysis was obtained by dissolving an aliquot of the product in a small amount of DCM and layering with *n*-pentane.

***R*F** (hex/EtOAc 1:1) = 0.25.

^{1}H-NMR (501 MHz, $CDCl_3$): Mixture of two rotamers with a ratio ≈ 55:45. δ = 7.02 (s, $1H_{maj}$), 6.96 (s, $1H_{min}$), 6.62 (s, $1H_{min}$), 6.60 (s, $1H_{maj}$), 6.58 (s, $1H_{maj}$), 6.57 (s, $1H_{min}$), 6.48 (s, $1H_{maj}$), 6.35 (s, $1H_{min}$), 5.36 (t, J = 7.0 Hz, $1H_{min}$), 5.28 (dd, J = 9.0, 4.8 Hz, $1H_{maj}$), 4.25 (ddd, J = 13.3, 6.0, 3.1 Hz, $1H_{maj}$), 3.90 (dt, J = 13.3, 5.4 Hz, $1H_{min}$), 3.87–3.81 (m, $6H_{all}$), 3.79 (s, $3H_{maj}$), 3.78–3.75 (m, $3H_{all}$), 3.69 (s, $3H_{min}$), 3.66 (s, $3H_{min}$), 3.46–3.29 (m, $3H_{maj}$ + $1H_{all}$), 3.21 (dt, J = 13.8, 5.5 Hz, $1H_{all}$), 3.09 (dd, J = 13.8, 7.5 Hz, $1H_{min}$), 2.99 (dd, J = 13.8, 9.1 Hz, $1H_{maj}$), 2.87 (ddd, J = 16.5, 10.9, 5.9 Hz, $1H_{maj}$), 2.79 (ddd, J = 15.5, 9.5, 5.7 Hz, $1H_{min}$), 2.63 (tt, J = 16.4, 4.3 Hz, $1H_{all}$).

^{13}C-NMR (126 MHz, $CDCl_3$): δ = 156.12, 156.03, 148.42, 148.28, 148.25, 148.19, 148.07, 147.99, 147.47, 147.31, 129.93, 129.77, 128.26, 126.54, 126.45, 115.46, 115.31, 115.04, 114.33, 113.99, 111.52, 111.26, 110.48, 110.22, 56.35, 56.23, 56.17, 56.14, 56.06, 56.01, 55.97, 55.00, 54.50, 52.72, 52.42, 42.29, 41.40, 39.03, 37.84, 28.19, 28.17.

ESI-HRMS: calculated for $C_{22}H_{26}Br_1N_1Na_1O_6$ ($[M+Na]^+$): 502.08357, found: 502.08375.

HPLC (OD-3, *n*-heptane/*i*-PrOH 80:20, 298 K, 286 nm): t_R (minor) = 7.9 min, t_R (major) = 9.9 min, er = 94.5:5.5 (89% ee).

$[\alpha]_D^{25}$ = −62.5 (*c* = 0.22, $CHCl_3$).

methyl (*R*)-6,7-dimethoxy-1-phenethyl-3,4-dihydroisoquinoline-2(1*H*)-carboxylate (2j):

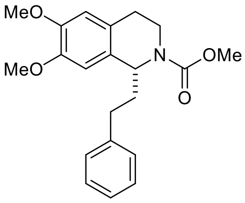

The reaction was performed according to **general procedure b** with carbamate **1a** (23.9 mg, 0.10 mmol, 1.00 eq.) and 3-phenylpropanal (16 µL, 0.12 mmol, 1.2 eq.). Purification by silica gel flash column chromatography (hex/EtOAc 70:30) afforded the product as a colorless oil (33.4 mg, 94 µmol, 94%).

***R*F** (hex/EtOAc 2:1) = 0.39.

^{1}H-NMR (501 MHz, $CDCl_3$): Mixture of two rotamers with a ratio ≈ 1:1. δ = 7.28 (d, J = 6.8 Hz, $2H_{all}$), 7.24–7.12 (m, $3H_{all}$), 6.63–6.56 (m, $2H_{all}$), 6.55 (s, 1H), 6.52 (s, 1H), 5.22 (dd, J = 10.1, 4.8 Hz, 1H), 5.05 (dd, J = 9.6, 4.5 Hz, 1H), 4.34–4.20 (m, 1H), 4.03 (d, J = 13.4 Hz, 1H), 3.84 (s, $3H_{all}$), 3.83 (s, $3H_{all}$), 3.75 (s, 3H), 3.73 (s, 3H), 3.29 (dt, J = 25.6, 11.8 Hz, $1H_{all}$), 3.00–2.68 (m, $3H_{all}$), 2.65 (ddd, J = 16.0, 4.3, 3.0 Hz, $1H_{all}$), 2.18–1.99 (m, $2H_{all}$).

^{13}C-NMR (126 MHz, $CDCl_3$): δ = 156.48, 156.38, 147.87, 147.79, 147.54, 142.07, 141.80, 129.88, 129.52, 128.47, 128.43, 126.18, 126.02, 125.91, 125.85, 111.72, 111.54, 110.20, 109.92, 56.13, 56.00, 54.55, 54.32, 52.77, 52.69, 38.59, 38.42, 38.20, 37.64, 32.87, 32.82, 28.16, 27.80.

CI-HRMS: calculated for $C_{21}H_{26}N_1O_4$ ($[M+H]^+$): 356.185634, found: 356.186280.

HPLC (OD-3, *n*-heptane/*i*-PrOH 90:10, 298 K, 282 nm): t_R (minor) = 6.7 min, t_R (major) = 9.5 min, er = 97:3 (94% ee).

$[\alpha]_D^{25}$ = −88.3 (*c* = 0.36, $CHCl_3$).

methyl (*R*)-1-(2-(benzo[*d*][1,3]dioxol-5-yl)ethyl)-6,7-dimethoxy-3,4-dihydroisoquinoline-2(1*H*)-carboxylate (2k):

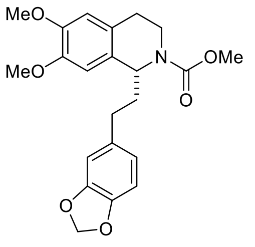

The reaction was performed according to **general procedure a** with carbamate **1a** (23.9 mg, 0.10 mmol, 1.00 eq.) and 3-(benzo[*d*][1,3]dioxol-5-yl)propanal (**S23**, 23.5 mg, 0.132 mmol, 1.32 eq.). Purification by silica gel flash column chromatography (hex/EtOAc 70:30) afforded the product as a colorless oil (38.3 mg, 96 µmol, 96%).

R_F (hex/EtOAc 2:1) = 0.30.

^{1}H-NMR (501 MHz, $CDCl_3$): Mixture of two rotamers with a ratio ≈ 1:1. δ = 6.79–6.61 (m, $3H_{all}$), 6.61–6.56 (m, $1H_{all}$), 6.54 (s, 1H), 6.51 (s, 1H), 5.94–5.85 (m, $2H_{all}$), 5.23–5.12 (m, 1H), 5.02 (dd, J = 9.2, 4.4 Hz, 1H), 4.25 (d, J = 13.3 Hz, 1H), 4.10–3.97 (m, 1H), 3.84 (s, $3H_{all}$), 3.83 (s, $3H_{all}$), 3.74 (s, $3H_{all}$), 3.26 (dd, J = 25.4, 13.3 Hz, $1H_{all}$), 2.98–2.78 (m, $1H_{all}$), 2.75–2.50 (m, $3H_{all}$), 2.12–1.90 (m, $2H_{all}$).

^{13}C-NMR (126 MHz, $CDCl_3$): δ = 156.47, 156.37, 147.88, 147.79, 147.67, 147.55, 145.77, 145.68, 135.93, 135.62, 129.84, 129.48, 126.17, 125.84, 121.13, 111.72, 111.55, 110.18, 109.90, 108.91, 108.24, 100.86, 56.13, 56.00, 54.44, 54.21, 52.76, 52.71, 38.88, 38.75, 38.18, 37.66, 32.62, 32.53, 28.15, 27.78.

ESI-HRMS: calculated for $C_{22}H_{25}N_1O_6Na_1$ ($[M+Na]^+$): 422.157408, found: 422.157530.

HPLC (OD-3, *n*-heptane/*i*-PrOH 70:30, 298 K, 286 nm): t_R (minor) = 4.6 min, t_R (major) = 6.3 min, er = 96.5:3.5 (93% ee).

$[\alpha]_D^{25}$ = −79.7 (*c* = 0.15, $CHCl_3$).

methyl (*R*)-1-(3,4-dimethoxyphenethyl)-6,7-dimethoxy-3,4-dihydroisoquinoline-2(1*H*)-carboxylate (2l):

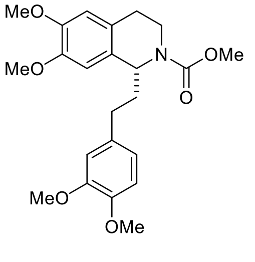

The reaction was performed according to **general procedure a** with carbamate **1a** (23.9 mg, 0.10 mmol, 1.00 eq.) and 3-(3,4-dimethoxyphenyl)propanal (**S22**, 25.6 mg, 0.132 mmol, 1.32 eq.). Purification by silica gel flash column chromatography (hex/EtOAc 1:1) afforded the product as a colorless oil (38.3 mg, 96 µmol, 96%).

R_F (hex/EtOAc 1:1) = 0.31.

^{1}H-NMR (501 MHz, $CDCl_3$): Mixture of two rotamers with a ratio ≈ 1:1. δ = 6.83–6.66 (m, $3H_{all}$), 6.62–6.56 (m, $1H_{all}$), 6.54 (s, 1H), 6.53 (s, 1H), 5.21 (dd, J = 9.9, 4.8 Hz, 1H), 5.05 (dd, J = 9.3, 4.5

Hz, 1H), 4.27 (dd, J = 13.8, 5.9 Hz, 1H), 4.02 (d, J = 12.9 Hz, 1H), 3.86 (s, $3H_{all}$), 3.86–3.83 (m, $6H_{all}$), 3.82 (s, $3H_{all}$), 3.74 (s, $3H_{all}$), 3.35–3.20 (m, $1H_{all}$), 2.99–2.79 (m, $1H_{all}$), 2.79–2.57 (m, $3H_{all}$), 2.19–1.94 (m, $2H_{all}$).

^{13}C-NMR (126 MHz, $CDCl_3$): δ = 156.48, 156.40, 148.95, 147.90, 147.80, 147.53, 147.36, 147.26, 134.68, 134.45, 129.88, 129.51, 126.18, 125.87, 120.16, 111.88, 111.72, 111.54, 111.39, 110.24, 109.94, 56.14, 56.03, 55.99, 55.96, 54.48, 54.33, 52.74, 38.74, 38.42, 38.24, 37.64, 32.37, 28.15, 27.79.

ESI-HRMS: calculated for $C_{23}H_{29}N_1O_6Na_1$ ($[M+Na]^+$): 438.188708, found: 438.188930.

HPLC (OD-3, *n*-heptane/*i*-PrOH 70:30, 298 K, 281 nm): t_R (minor) = 6.5 min, t_R (major) = 8.6 min, er = 97:3 (94% ee).

$[\alpha]_D^{25}$ = −72.1 (*c* = 0.32, $CHCl_3$).

methyl (*R*)-6,7-dimethoxy-1-(3,4,5-trimethoxyphenethyl)-3,4-dihydroisoquinoline-2(1*H*)-carboxylate (2m):

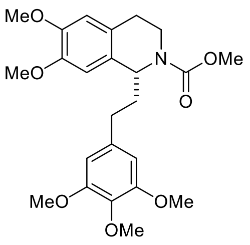

The reaction was performed according to **general procedure a** with carbamate **1a** (23.9 mg, 0.10 mmol, 1.00 eq.) and 3-(3,4,5-trimethoxyphenyl)propanal (**S24**, 29.6 mg, 0.132 mmol, 1.32 eq.). Purification by silica gel flash column chromatography (hex/EtOAc 3:2 to 1:1) afforded the product as a colorless oil (36.3 mg, 81 μmol, 81%).

***R*$_F$** (hex/EtOAc 1:1) = 0.29.

^{1}H-NMR (501 MHz, $CDCl_3$): Mixture of two rotamers with a ratio ≈ 1:1. δ = 6.59 (s, $1H_{all}$), 6.57–6.52 (m, $1H_{all}$), 6.46–6.37 (m, $2H_{all}$), 5.21 (dd, J = 9.9, 4.9 Hz, 1H), 5.10–5.03 (m, 1H), 4.28 (dd, J = 13.6, 5.9 Hz, 1H), 4.02 (d, J = 13.1 Hz, 1H), 3.87–3.82 (m, $12H_{all}$), 3.81 (s, $3H_{all}$), 3.74 (s, $3H_{all}$), 3.36–3.20 (m, $1H_{all}$), 2.99–2.79 (m, $1H_{all}$), 2.79–2.57 (m, $3H_{all}$), 2.21–1.94 (m, $2H_{all}$).

^{13}C-NMR (126 MHz, $CDCl_3$): δ = 156.50, 156.40, 153.28, 153.22, 147.97, 147.86, 147.57, 137.80, 137.61, 136.36, 136.21, 129.78, 129.40, 126.21, 125.92, 111.75, 111.57, 110.28, 109.97, 105.36, 60.95, 56.22, 56.18, 56.01, 54.51, 54.37, 52.78, 38.56, 38.30, 38.16, 37.67, 33.20, 28.16, 27.80.

ESI-HRMS: calculated for $C_{24}H_{31}N_1O_7Na_1$ ($[M+Na]^+$): 468.199273, found: 468.198940.

HPLC (OD-3, *n*-heptane/*i*-PrOH 70:30, 298 K, 282 nm): t_R (minor) = 6.2 min, t_R (major) = 8.6 min, er = 97:3 (94% ee).

$[\alpha]_D^{25}$ = −66.1 (*c* = 0.35, $CHCl_3$).

methyl (*R*)-6,7-dimethoxy-1-pentyl-3,4-dihydroisoquinoline-2(1*H*)-carboxylate (2n):

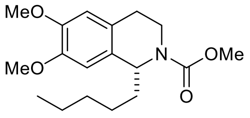

The reaction was performed according to **general procedure b** with carbamate **1a** (23.9 mg, 0.10 mmol, 1.00 eq.) and hexanal (15 μL, 0.12 mmol, 1.2 eq.). Purification by silica gel flash column chromatography (hex/EtOAc 70:30) afforded the product as a colorless oil (29.2 mg, 91 μmol, 91%).

$\boldsymbol{R}_F$ (hex/EtOAc 2:1) = 0.64.

^{1}H-NMR (501 MHz, $CDCl_3$): Mixture of two rotamers with a ratio ≈ 1:1. δ = 6.61–6.53 (m, $2H_{all}$), 5.09 (dd, J = 9.7, 5.1 Hz, 1H), 4.97 (dd, J = 10.1, 4.4 Hz, 1H), 4.28–4.19 (m, 1H), 4.04–3.96 (m, 1H), 3.85 (s, $3H_{all}$), 3.84 (s, $3H_{all}$), 3.71 (s, $3H_{all}$), 3.33–3.24 (m, 1H), 3.19 (ddd, J = 15.5, 11.9, 4.2 Hz, 1H), 2.86 (dddd, J = 32.5, 16.3, 11.0, 6.1 Hz, $1H_{all}$), 2.62 (dt, J = 16.2, 3.7 Hz, $1H_{all}$), 1.84–1.61 (m, $2H_{all}$), 1.50–1.21 (m, $6H_{all}$), 0.93–0.84 (m, $3H_{all}$).

^{13}C-NMR (126 MHz, $CDCl_3$): δ = 156.43, 147.80, 147.70, 147.49, 130.45, 130.07, 126.14, 125.82, 111.72, 111.54, 110.31, 110.01, 56.16, 56.02, 54.65, 54.52, 52.69, 52.63, 38.09, 37.42, 36.98, 36.81, 31.91, 31.78, 28.20, 27.89, 26.17, 26.11, 22.74, 14.19.

EI-HRMS: calculated for $C_{18}H_{27}N_1O_4$ ($[M]^+$): 321.193459, found: 321.193110.

HPLC (OD-3, *n*-heptane/*i*-PrOH 97:3, 298 K, 282 nm): t_R (minor) = 7.0 min, t_R (major) = 8.4 min, er = 97.5:2.5 (95% ee).

$[\alpha]_D^{25}$ = −89.2 (*c* = 0.21, $CHCl_3$).

methyl (*R*)-1-isobutyl-6,7-dimethoxy-3,4-dihydroisoquinoline-2(1*H*)-carboxylate (2o):

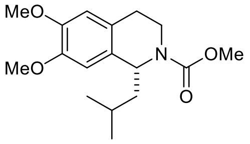

The reaction was performed according to **general procedure b** with carbamate **1a** (23.9 mg, 0.10 mmol, 1.00 eq.) and isovaleraldehyde (13 µL, 0.12 mmol, 1.2 eq.). Purification by silica gel flash column chromatography (hex/EtOAc 4:1) afforded the product as a colorless oil (28.3 mg, 92 µmol, 92%).

$\boldsymbol{R}_F$ (hex/EtOAc 2:1) = 0.59.

^{1}H-NMR (501 MHz, $CDCl_3$): Mixture of two rotamers with a ratio ≈ 1:1. δ = 6.58 (s, 1H), 6.55 (s, 1H), 6.54 (s, 1H), 6.50 (s, 1H), 5.20 (dd, J = 10.7, 4.2 Hz, 1H), 5.05 (dd, J = 10.8, 3.9 Hz, 1H), 4.23 (ddd, J = 13.5, 6.3, 2.2 Hz, 1H), 4.02 (ddd, J = 13.5, 6.1, 2.7 Hz, 1H), 3.85 (d, J = 2.7 Hz, $3H_{all}$), 3.83 (d, J = 2.4 Hz, $3H_{all}$), 3.71 (d, J = 2.7 Hz, $3H_{all}$), 3.26 (ddd, J = 13.5, 11.3, 4.4 Hz, 1H), 3.19 (td, J = 12.6, 4.3 Hz, 1H), 2.88 (dddd, J = 33.7, 16.8, 11.4, 6.2 Hz, $1H_{all}$), 2.60 (ddd, J = 16.1, 4.4, 2.5 Hz, $1H_{all}$), 1.78 (ddt, J = 14.3, 10.6, 3.6 Hz, $1H_{all}$), 1.72–1.58 (m, $1H_{all}$), 1.48–1.35 (m, $1H_{all}$), 1.08 (d, J = 6.4 Hz, 3H), 1.04 (d, J = 6.5 Hz, 3H), 0.96–0.91 (m, $3H_{all}$).

^{13}C-NMR (126 MHz, $CDCl_3$): δ = 156.44, 156.37, 147.82, 147.69, 147.51, 130.81, 130.38, 126.13, 125.76, 111.80, 111.62, 110.24, 109.97, 56.20, 56.18, 56.02, 52.88, 52.85, 52.72, 52.61, 46.44, 46.14, 37.72, 37.15, 28.07, 27.71, 25.21, 25.06, 23.72, 23.64, 22.33, 21.91.

ESI-HRMS: calculated for $C_{17}H_{25}N_1O_4Na_1$ ($[M+Na]^+$): 330.167578, found: 330.167580.

HPLC (OD-3, *n*-heptane/*i*-PrOH 97:3, 298 K, 282 nm): t_R (minor) = 6.4 min, t_R (major) = 8.2 min, er = 97:3 (94% ee).

$[\alpha]_D^{25}$ = −82.2 (*c* = 0.24, $CHCl_3$).

methyl (*R*)-1-cyclopentyl-6,7-dimethoxy-3,4-dihydroisoquinoline-2(1*H*)-carboxylate (2p):

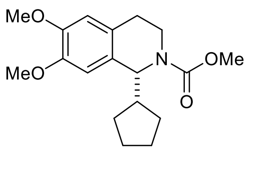

The reaction was performed according to **general procedure b** with carbamate **1a** (23.9 mg, 0.10 mmol, 1.00 eq.) and cyclopentanecarbaldehyde (13 µL, 0.12 mmol, 1.2 eq.). Purification by silica gel flash column chromatography (hex/EtOAc 4:1) afforded the product as a colorless oil (21.7 mg, 68 µmol, 68%).

R_F (hex/EtOAc 2:1) = 0.48.

^{1}H-NMR (501 MHz, $CDCl_3$): Mixture of two rotamers with a ratio ≈ 1:1. δ = 6.66 (s, 1H), 6.63 (s, 1H), 6.60 (s, 1H), 6.59 (s, 1H), 4.90 (d, J = 9.8 Hz, 1H), 4.75 (d, J = 9.7 Hz, 1H), 4.18 (ddd, J = 13.4, 7.0, 3.4 Hz, 1H), 3.94 (ddd, J = 13.2, 6.6, 4.7 Hz, 1H), 3.87 – 3.81 (m, $6H_{all}$), 3.70 (s, $3H_{all}$), 3.43 (ddd, J = 13.4, 9.2, 5.6 Hz, 1H), 3.34 (ddd, J = 13.3, 10.2, 5.4 Hz, 1H), 2.92 (ddd, J = 16.9, 10.2, 7.0 Hz, 1H), 2.84 (ddd, J = 15.9, 9.2, 6.6 Hz, 1H), 2.78–2.68 (m, $1H_{all}$), 2.15 (dtd, J = 10.1, 6.8, 2.9 Hz, $1H_{all}$), 1.83–1.61 (m, $4H_{all}$), 1.59–1.33 (m, $4H_{all}$).

^{13}C-NMR (126 MHz, $CDCl_3$): δ = 156.59, 156.20, 147.97, 147.84, 146.93, 146.87, 130.39, 129.92, 126.10, 125.82, 111.81, 111.57, 111.05, 110.77, 58.97, 58.94, 56.17, 56.13, 52.70, 52.62, 47.02, 46.89, 38.89, 38.21, 31.42, 31.33, 30.01, 29.96, 27.81, 27.51, 25.41, 25.32, 24.38, 24.25.

ESI-HRMS: calculated for $C_{18}H_{25}N_1O_4Na_1$ ($[M+Na]^+$): 342.167578, found: 342.167800.

HPLC (OJ-3R, MeOH/H_2O 90:10, 298 K, 283 nm): t_R (minor) = 4.5 min, t_R (major) = 6.6 min, er = 97:3 (94% ee).

$[\alpha]_D^{25}$ = −60.2 (*c* = 0.22, $CHCl_3$).

methyl (*R*)-6,7-dimethoxy-1-methyl-3,4-dihydroisoquinoline-2(1*H*)-carboxylate (2q):

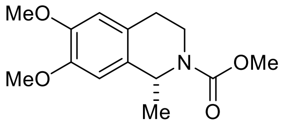

The reaction was performed according to **general procedure b** with carbamate **1a** (23.9 mg, 0.10 mmol, 1.00 eq.) and acetaldehyde (17 µL, 0.30 mmol, 3.0 eq.) at −40 °C. Purification by silica gel flash column chromatography (hex/EtOAc 4:1) afforded the product as a colorless oil (26.5 mg, 100 µmol, >99%).

R_F (hex/EtOAc 2:1) = 0.32.

^{1}H-NMR (501 MHz, $CDCl_3$): Mixture of two rotamers with a ratio ≈ 1:1. δ = 6.62–6.51 (m, $2H_{all}$), 5.27–5.01 (m, $1H_{all}$), 4.31–4.16 (m, 1H), 4.11–3.99 (m, 1H), 3.85 (s, $3H_{all}$), 3.84 (s, $3H_{all}$), 3.73 (s, $3H_{all}$), 3.33–3.13 (m, $1H_{all}$), 2.94–2.77 (m, $1H_{all}$), 2.63 (dt, J = 15.9, 3.4 Hz, $1H_{all}$), 1.43 (d, J = 6.8 Hz, $3H_{all}$).

^{13}C-NMR (126 MHz, $CDCl_3$): δ = 147.74, 130.72, 130.26, 126.12, 125.76, 111.54, 109.84, 56.14, 56.03, 52.69, 50.20, 37.85, 37.43, 28.63, 28.49, 22.26, 21.83.

EI-HRMS: calculated for $C_{14}H_{19}N_1O_4$ ($[M]^+$): 265.130858, found: 265.130920.

HPLC (IC-3R, MeOH/H_2O 90:10, 298 K, 284 nm): t_R (minor) = 6.3 min, t_R (major) = 7.9 min, er = 98.5:1.5 (97% ee).

$[\alpha]_D^{25}$ = −119.4 (*c* = 0.28, $CHCl_3$).

methyl (*R*)-1-(3-(benzyloxy)propyl)-6,7-dimethoxy-3,4-dihydroisoquinoline-2(1*H*)-carboxylate (2r):

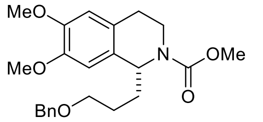

The reaction was performed according to **general procedure a** with carbamate **1a** (23.9 mg, 0.10 mmol, 1.00 eq.) and 4-(benzyloxy)butanal (23.5 mg, 0.132 mmol, 1.32 eq.). Purification by silica gel flash column chromatography (DCM/acetone 19:1) and another silica gel flash column chromatography (hex/acetone 4:1) afforded the product as a colorless oil (31.9 mg, 80 µmol, 80%) as well as unreacted starting material **1a** (4.8 mg, 20 µmol, 20%).

***R*$_F$** (hex/EtOAc 2:1) = 0.33.

^{1}H-NMR (501 MHz, $CDCl_3$): Mixture of two rotamers with a ratio ≈ 1:1. δ = 7.38–7.23 (m, 5H$_{all}$), 6.63–6.52 (m, 2H$_{all}$), 5.19–5.08 (m, 1H), 5.05–4.94 (m, 1H), 4.50 (s, 2H$_{all}$), 4.23 (dd, J = 13.9, 5.9 Hz, 1H), 4.01 (d, J = 13.6 Hz, 1H), 3.84 (s, 3H$_{all}$), 3.83–3.80 (m, 3H$_{all}$), 3.71 (s, 3H), 3.69 (s, 3H), 3.60–3.45 (m, 2H$_{all}$), 3.27 (t, J = 12.1 Hz, 1H), 3.19 (td, J = 12.7, 4.1 Hz, 1H), 2.94–2.79 (m, 1H$_{all}$), 2.65–2.57 (m, 1H$_{all}$), 1.95–1.66 (m, 4H$_{all}$).

^{13}C-NMR (126 MHz, $CDCl_3$): δ = 156.47, 156.38, 147.85, 147.75, 147.55, 138.74, 138.64, 130.15, 129.78, 128.50, 127.76, 127.71, 127.64, 126.14, 125.83, 111.71, 111.53, 110.26, 109.99, 73.12, 73.06, 70.12, 70.05, 56.14, 56.03, 54.30, 54.27, 52.72, 38.06, 37.45, 33.48, 33.29, 28.20, 27.83, 26.76.

ESI-HRMS: calculated for $C_{23}H_{29}N_1O_5Na_1$ ([M+Na]$^+$): 422.193793, found: 422.194220.

HPLC (OD-3, *n*-heptane/*i*-PrOH 80:20, 298 K, 282 nm): t_R (minor) = 4.9 min, t_R (major) = 6.7 min, er = 96.5:3.5 (93% ee).

$[\alpha]_D^{25}$ = −62.4 (*c* = 0.26, $CHCl_3$).

methyl (*R*)-6,7-dimethoxy-1-(3-methoxy-3-oxopropyl)-3,4-dihydroisoquinoline-2(1*H*)-carboxylate (2s):

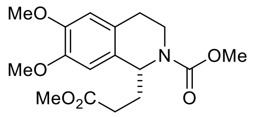

The reaction was performed according to **general procedure a** with carbamate **1a** (23.9 mg, 0.10 mmol, 1.00 eq.) and methyl 4-oxobutanoate (15.3 mg, 0.132 mmol, 1.32 eq.). Purification by silica gel flash column chromatography (DCM/Et_2O 9:1) afforded the product as a colorless oil (32.4 mg, 96 µmol, 96%).

***R*$_F$** (DCM/Et_2O 9:1) = 0.22.

^{1}H-NMR (501 MHz, $CDCl_3$): Mixture of two rotamers with a ratio ≈ 55:45. δ = 6.63 (s, 1H$_{min}$), 6.62 (s, 1H$_{maj}$), 6.58 (s, 1H$_{maj}$), 6.56 (s, 1H$_{min}$), 5.14 (dd, J = 10.5, 4.5 Hz, 1H$_{min}$), 5.04 (dd, J = 11.0, 3.6 Hz, 1H$_{maj}$), 4.23 (ddd, J = 13.4, 6.1, 2.2 Hz, 1H$_{maj}$), 4.00 (ddd, J = 13.6, 5.9, 2.9 Hz, 1H$_{min}$), 3.86 (s, 3H$_{maj}$), 3.86 (s, 3H$_{min}$), 3.85 (s, 3H$_{min}$), 3.84 (s, 3H$_{maj}$), 3.71 (s, 3H$_{min}$), 3.70 (s, 3H$_{maj}$), 3.68 (s, 3H$_{maj}$), 3.67 (s, 3H$_{min}$), 3.23 (ddd, J = 13.5, 11.2, 4.2 Hz, 1H$_{min}$), 3.13 (ddd, J = 13.3, 11.7, 4.1 Hz, 1H$_{maj}$), 2.85 (dddd, J = 32.4, 16.6, 11.5, 5.9 Hz, 1H$_{all}$), 2.61 (ddt, J = 16.3, 4.5, 2.4 Hz, 1H$_{all}$), 2.56–2.36 (m, 2H$_{all}$), 2.20–1.95 (m, 2H$_{all}$).

^{13}C-NMR (126 MHz, $CDCl_3$): δ = 173.95, 173.78, 156.58, 156.25, 147.99, 147.92, 147.69, 129.30, 128.96, 126.19, 125.93, 111.66, 111.54, 110.13, 109.89, 56.15, 56.03, 54.04, 53.76, 52.84, 52.71, 51.77, 38.16, 37.46, 31.51, 31.41, 31.19, 30.86, 28.15, 27.83.

EI-HRMS: calculated for $C_{17}H_{23}N_1O_6$ ($[M]^+$): 337.151989, found: 337.152020.

HPLC (OJ-3, *n*-heptane/*i*-PrOH 80:20, 298 K, 300 nm): t_R (minor) = 9.0 min, t_R (major) = 11.9 min, er = 97.5:2.5 (95% ee).

$[\alpha]_D^{25}$ = −78.1 (*c* = 0.21, $CHCl_3$).

methyl (*R*)-1-(*tert*-butoxymethyl)-6,7-dimethoxy-3,4-dihydroisoquinoline-2(1*H*)-carboxylate (2t):

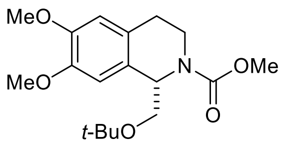

The reaction was performed according to **general procedure a** with carbamate **1a** (23.9 mg, 0.10 mmol, 1.00 eq.) and 2-(*tert*-butoxy)acetaldehyde (**S25**, 58 wt% in DCM, 26.5 mg, 0.132 mmol, 1.32 eq.) in *n*-pentane (4.4 mL). Purification by silica gel flash column chromatography (hex/EtOAc 40%) afforded the product as a colorless oil (33.5 mg, 99 µmol, 99%).

***R*$_F$** (hex/EtOAc 2:1) = 0.37.

^{1}H-NMR (501 MHz, $CDCl_3$): Mixture of two rotamers with a ratio ≈ 60:40. δ = δ 6.79 (s, $1H_{min}$), 6.74 (s, $1H_{maj}$), 6.62–6.53 (m, $1H_{all}$), 5.13–5.01 (m, $1H_{all}$), 4.27–4.16 (m, $1H_{maj}$), 3.97–3.90 (m, $1H_{min}$), 3.84 (s, $6H_{all}$), 3.72 (s, $3H_{all}$), 3.66–3.60 (m, $1H_{min}$), 3.57 (t, J = 8.2 Hz, $1H_{all}$), 3.54–3.48 (m, $1H_{maj}$), 3.48–3.39 (m, $1H_{min}$), 3.26 (td, J = 12.2, 4.1 Hz, $1H_{maj}$), 2.93–2.73 (m, $1H_{all}$), 2.73–2.59 (m, $1H_{all}$), 1.12 (s, $9H_{maj}$), 1.10 (s, $9H_{min}$).

^{13}C-NMR (126 MHz, $CDCl_3$): δ = 156.64, 156.23, 148.03, 147.80, 147.22, 127.21, 126.94, 126.87, 126.36, 111.46, 111.14, 111.05, 110.80, 73.36, 73.25, 64.97, 64.93, 56.04, 55.96, 54.82, 54.68, 52.64, 39.74, 38.35, 28.56, 28.25, 27.54.

ESI-HRMS: calculated for $C_{18}H_{27}N_1O_5Na_1$ ($[M+Na]^+$): 360.178143, found: 360.178030.

HPLC (OD-3, *n*-heptane/*i*-PrOH 98:2, 298 K, 282 nm): t_R (minor) = 9.4 min, t_R (major) = 10.2 min, er = 91.5:8.5 (83% ee).

$[\alpha]_D^{25}$ = −70.0 (*c* = 0.22, $CHCl_3$).

methyl (*R*)-1-benzyl-6-methoxy-3,4-dihydroisoquinoline-2(1*H*)-carboxylate (8):

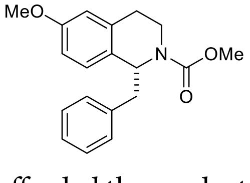

The reaction was performed according to **general procedure b** with carbamate **S3** (20.9 mg, 0.10 mmol, 1.00 eq.) and phenylacetaldehyde (14 µL, 0.12 mmol, 1.2 eq.). Purification by silica gel flash column chromatography (hex/EtOAc 4:1) and another silica gel flash column chromatography (DCM/EtOAc 3%) afforded the product as a colorless oil (26.3 mg, 84 µmol, 84%).

***R*$_F$** (hex/EtOAc 2:1) = 0.54.

^{1}H-NMR (501 MHz, $CDCl_3$): Mixture of two rotamers with a ratio ≈ 60:40. δ = 7.29–7.17 (m, $3H_{all}$), 7.09 (d, J = 7.0 Hz, $2H_{maj}$), 7.05 (d, J = 7.1 Hz, $2H_{min}$), 6.90 (d, J = 8.5 Hz, $1H_{maj}$), 6.76–6.70 (m, $1H_{all}$), 6.68–6.61 (m, $1H_{all}$ + $1H_{min}$), 5.32 (t, J = 6.7 Hz, $1H_{min}$), 5.20 (dd, J = 8.1, 5.7 Hz, $1H_{maj}$), 4.14 (ddt, J = 11.6, 5.5, 2.8 Hz, $1H_{maj}$), 3.79 (s, $3H_{maj}$), 3.78 (s, $3H_{min}$), 3.77–3.72 (m, $1H_{min}$), 3.69 (s, $3H_{min}$), 3.41 (s, $3H_{maj}$), 3.39–3.28 (m, $1H_{all}$), 3.13 (dd, J = 13.3, 6.1 Hz, $1H_{min}$), 3.09–2.97 (m, $1H_{all}$ + $1H_{maj}$), 2.89

(ddd, J = 16.3, 10.4, 5.9 Hz, $1H_{maj}$), 2.76 (ddd, J = 14.8, 8.5, 5.5 Hz, $1H_{min}$), 2.67 (dt, J = 16.2, 4.2 Hz, $1H_{maj}$), 2.57 (dt, J = 15.9, 5.3 Hz, $1H_{min}$).

^{13}C-NMR (126 MHz, $CDCl_3$): δ = 158.33, 158.28, 156.18, 138.38, 138.25, 135.83, 135.75, 129.90, 129.69, 128.86, 128.67, 128.32, 128.29, 128.20, 126.50, 126.44, 113.49, 113.31, 112.47, 111.99, 56.30, 56.27, 55.37, 55.33, 52.68, 52.38, 43.40, 43.00, 39.40, 38.00, 28.92, 28.89.

ESI-HRMS: calculated for $C_{19}H_{21}N_1O_3Na_1$ ($[M+Na]^+$): 334.141363, found: 334.141270.

HPLC (OJ-3R, CH_3CN/H_2O 70:30, 298 K, 278 nm): t_R (minor) = 3.5 min, t_R (major) = 4.9 min, er = 97.5:2.5 (95% ee).

$[\alpha]_D^{25}$ = −48.6 (*c* = 0.22, $CHCl_3$).

methyl (*R*)-1-benzyl-8-bromo-6,7-dimethoxy-3,4-dihydroisoquinoline-2(1*H*)-carboxylate (9):

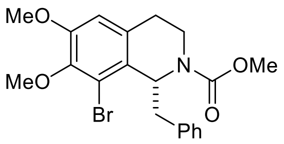

The reaction was performed according to **general procedure b** with carbamate **S5** (31.8 mg, 0.10 mmol, 1.00 eq.) and phenylacetaldehyde (14 µL, 0.12 mmol, 1.2 eq.). Purification by silica gel flash column chromatography (DCM/EtOAc 19:1) afforded the product as a colorless oil (24.9 mg, 59 µmol, 59%).

***R*$_F$** (hex/EtOAc 2:1) = 0.29.

^{1}H-NMR (501 MHz, $CDCl_3$): Mixture of two rotamers with a ratio ≈ 70:30. δ = 7.32–7.10 (m, $5H_{all}$), 6.66 (s, $1H_{maj}$), 6.60 (s, $1H_{min}$), 5.69 (dd, J = 8.9, 4.4 Hz, $1H_{min}$), 5.46 (dd, J = 10.3, 3.3 Hz, $1H_{maj}$), 4.17 (ddd, J = 13.3, 6.7, 3.7 Hz, $1H_{maj}$), 3.87 (s, $3H_{maj}$), 3.86 (s, $3H_{maj}$), 3.85 (s, $3H_{min}$), 3.83 (s, $3H_{min}$), 3.76 (dt, J = 12.3, 5.8 Hz, $1H_{min}$), 3.60 (s, $3H_{min}$), 3.57–3.46 (m, $1H_{all}$), 3.37 (dd, J = 14.1, 4.4 Hz, $1H_{min}$), 3.30 (dd, J = 14.0, 3.4 Hz, $1H_{maj}$), 3.26 (s, $3H_{maj}$), 2.94–2.84 (m, $1H_{all}$), 2.80 (dd, J = 13.9, 10.3 Hz, $1H_{maj}$), 2.76–2.65 (m, $1H_{all}$), 2.41 (dt, J = 16.2, 5.4 Hz, $1H_{min}$).

^{13}C-NMR (126 MHz, $CDCl_3$): δ = 156.13, 152.29, 152.19, 145.20, 138.51, 138.19, 131.91, 131.77, 129.60, 129.47, 129.39, 129.09, 128.27, 128.20, 126.58, 126.54, 118.77, 118.41, 112.15, 111.79, 60.70, 60.66, 56.47, 56.21, 55.93, 52.74, 52.37, 39.51, 39.44, 38.51, 37.22, 28.37.

ESI-HRMS: calculated for $C_{20}H_{22}N_1O_4Br_1Na_1$ ($[M+Na]^+$): 442.062453, found: 442.062560.

HPLC (OD-3, *n*-heptane/*i*-PrOH 97:3, 298 K, 287 nm): t_R (minor) = 9.4 min, t_R (major) = 10.4 min, er = 97:3 (94% ee).

$[\alpha]_D^{25}$ = −33.2 (*c* = 0.25, $CHCl_3$).

methyl (*R*)-5-benzyl-7,8-dihydro-[1,3]dioxolo[4,5-*g*]isoquinoline-6(5*H*)-carboxylate (10):

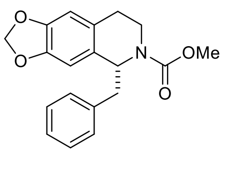

The reaction was performed according to **general procedure b** with carbamate **S6** (22.3 mg, 0.10 mmol, 1.00 eq.) and phenylacetaldehyde (14 µL, 0.12 mmol, 1.2 eq.) in CyH. Purification by silica gel flash column chromatography (hex/EtOAc 3:1) afforded the product as a colorless oil (27.9 mg, 86 µmol, 86%).

R_F (hex/EtOAc 2:1) = 0.61.

^{1}H-NMR (501 MHz, $CDCl_3$): Mixture of two rotamers with a ratio ≈ 55:45. δ = 7.30–7.17 (m, $3H_{all}$), 7.12–7.03 (m, $2H_{all}$), 6.58 (s, $1H_{maj}$), 6.55 (s, $1H_{min}$), 6.47 (s, $1H_{maj}$), 6.32 (s, $1H_{min}$), 5.95–5.85 (m, $2H_{all}$), 5.26 (t, J = 6.6 Hz, $1H_{min}$), 5.13 (t, J = 6.8 Hz, $1H_{maj}$), 4.12 (ddd, J = 13.1, 6.0, 4.0 Hz, $1H_{maj}$), 3.73 (dt, J = 12.1, 5.6 Hz, $1H_{min}$), 3.68 (s, $3H_{min}$), 3.39 (s, $3H_{maj}$), 3.35–3.23 (m, $1H_{all}$), 3.13–2.96 (m, $2H_{all}$), 2.81 (ddd, J = 16.2, 10.4, 5.9 Hz, $1H_{maj}$), 2.69 (ddd, J = 14.9, 8.7, 5.5 Hz, $1H_{min}$), 2.58 (dt, J = 16.1, 4.2 Hz, $1H_{maj}$), 2.46 (dt, J = 15.9, 5.3 Hz, $1H_{min}$).

^{13}C-NMR (126 MHz, $CDCl_3$): δ = 156.14, 156.04, 146.54, 146.40, 146.06, 145.88, 138.23, 138.08, 129.82, 129.78, 129.64, 128.33, 128.26, 127.76, 127.73, 126.59, 126.56, 108.72, 108.36, 107.67, 107.20, 101.01, 100.92, 56.70, 52.70, 52.39, 43.24, 42.84, 39.42, 38.06, 28.57, 28.54.

CI-HRMS: calculated for $C_{19}H_{20}N_1O_4$ ($[M+H]^+$): 326.138684, found: 326.139160.

HPLC (OD-3, *n*-heptane/*i*-PrOH 98:2, 298 K, 290 nm): t_R (minor) = 11.0 min, t_R (major) = 12.1 min, er = 95.5:4.5 (91% ee).

$[\alpha]_D^{25}$ = −48.0 (*c* = 0.17, $CHCl_3$).

methyl (*R*)-5-(3,4-dimethoxybenzyl)-7,8-dihydro-[1,3]dioxolo[4,5-*g*]isoquinoline-6(5*H*)-carboxylate (11):

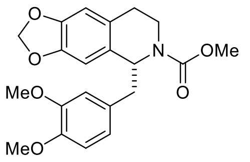

The reaction was performed according to **general procedure a** with carbamate **S6** (22.3 mg, 0.10 mmol, 1.00 eq.) and 2-(3,4-dimethoxyphenyl)acetaldehyde (**S10**, 22.3 mg, 0.132 mmol, 1.32 eq.) in CyH/$CHCl_3$ 10:1 (2.2 mL). Purification by silica gel flash column chromatography (hex/EtOAc 3:1) afforded the product as a white solid (27.9 mg, 86 µmol, 86%).

R_F (DCM/EtOAc 9:1) = 0.47.

^{1}H-NMR (501 MHz, $CDCl_3$): Mixture of two rotamers with a ratio ≈ 50:50. δ = 6.77 (d, J = 8.1 Hz, 1H), 6.73 (d, J = 8.1 Hz, 1H), 6.62 (dd, J = 8.1, 1.9 Hz, 1H), 6.60–6.52 (m, $2H_{all}$ + 1H), 6.48 (s, 1H), 6.34 (s, 1H), 5.90 (d, J = 4.8 Hz, 2H), 5.88 (d, J = 8.3 Hz, 2H), 5.23 (t, J = 6.5 Hz, 1H), 5.10 (t, J = 6.6 Hz, 1H), 4.07 (ddd, J = 13.1, 5.9, 4.0 Hz, 1H), 3.86–3.84 (m, $3H_{all}$), 3.81 (s, 3H), 3.77 (s, 3H), 3.73–3.61 (m, 3H + 1H), 3.48 (s, 3H), 3.25 (dddd, J = 27.6, 13.6, 9.3, 4.6 Hz, $1H_{all}$), 3.00 (dtd, J = 31.7, 13.3, 6.9 Hz, $2H_{all}$), 2.79 (ddd, J = 16.1, 10.2, 5.9 Hz, 1H), 2.68 (ddd, J = 14.6, 8.4, 5.4 Hz, 1H), 2.54 (dt, J = 16.1, 4.3 Hz, 1H), 2.45 (dt, J = 15.9, 5.4 Hz, 1H).

^{13}C-NMR (126 MHz, $CDCl_3$): δ = 156.18, 156.10, 148.77, 148.65, 147.85, 147.77, 146.51, 146.38, 146.04, 145.86, 130.73, 130.57, 129.79, 129.63, 127.85, 127.82, 121.91, 121.82, 112.91, 112.83, 111.15, 111.01, 108.70, 108.35, 107.80, 107.27, 101.00, 100.91, 56.64, 56.62, 56.03, 55.98, 55.94, 55.88, 52.71, 52.55, 42.80, 42.34, 39.56, 38.24, 28.58, 28.51.

ESI-HRMS: calculated for $C_{21}H_{23}N_1O_6Na_1$ ([M+Na]$^+$): 408.141758, found: 408.141880.

HPLC (OJ-3, *n*-heptane/*i*-PrOH 70:30, 298 K, 287 nm): t_R (minor) = 8.8 min, t_R (major) = 12.7 min, er = 94.5:5.5 (89% ee).

$[\alpha]_D^{25}$ = −43.4 (*c* = 0.27, $CHCl_3$).

methyl (*R*)-7-hydroxy-6-methoxy-1-(3,4,5-trimethoxyphenethyl)-3,4-dihydroisoquinoline-2(1*H*)-carboxylate (12):

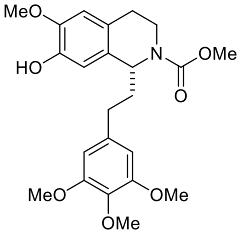

The reaction was performed according to **general procedure a** with carbamate **S8** (22.5 mg, 0.10 mmol, 1.00 eq.) and 3-(3,4,5-trimethoxyphenyl)propanal (**S24**, 29.6 mg, 0.132 mmol, 1.32 eq.). Purification by silica gel flash column chromatography (hex/EtOAc 40–50%) afforded the product as a white foam (36.1 mg, 84 µmol, 84%).

***R*F** (hex/EtOAc 1:1) = 0.26.

^{1}H-NMR (501 MHz, $CDCl_3$): Mixture of two rotamers with a ratio ≈ 1:1. δ = 6.66 (s, $1H_{all}$), 6.57 (s, $1H_{all}$), 6.43–6.38 (m, $2H_{all}$), 5.55 (s, $1H_{all}$), 5.17 (s, 1H), 5.03 (s, 1H), 4.25 (d, J = 13.0 Hz, 1H), 4.00 (t, J = 8.4 Hz, 1H), 3.86–3.83 (m, $9H_{all}$), 3.81 (s, $3H_{all}$), 3.73 (s, $3H_{all}$), 3.36–3.17 (m, $1H_{all}$), 2.99–2.78 (m, $1H_{all}$), 2.64 (dt, J = 16.0, 3.8 Hz, $1H_{all}$), 2.05 (s, $2H_{all}$).

^{13}C-NMR (126 MHz, $CDCl_3$): δ = 156.49, 153.22, 145.50, 144.08, 137.86, 137.62, 136.28, 136.17, 130.50, 130.18, 125.53, 125.35, 113.14, 112.71, 111.02, 110.84, 105.33, 60.97, 56.22, 56.08, 54.50, 54.33, 52.77, 38.47, 38.22, 37.81, 33.18, 28.33, 27.94.

CI-HRMS: calculated for $C_{23}H_{29}N_1O_7Na_1$ ([M+Na]$^+$): 454.183623, found: 454.183480.

HPLC (IC-3R, MeOH/H_2O 90:10, 298 K, 284 nm): t_R (minor) = 10.6 min, t_R (major) = 13.2 min, er = 95:5 (90% ee).

$[\alpha]_D^{25}$ = −48.9 (*c* = 0.38, $CHCl_3$).

methyl (*R*)-7-hydroxy-1-isobutyl-6-methoxy-3,4-dihydroisoquinoline-2(1*H*)-carboxylate (13):

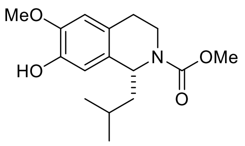

The reaction was performed according to **general procedure b** with carbamate **S8** (22.5 mg, 0.10 mmol, 1.00 eq.) and isovaleraldehyde (13 µL, 0.12 mmol, 1.2 eq.). Purification by silica gel flash column chromatography (hex/EtOAc 2:1) afforded the product as a colorless oil (29.0 mg, 89 µmol, 84%).

***R*F** (hex/EtOAc 2:1) = 0.49.

^{1}H-NMR (501 MHz, $CDCl_3$): Mixture of two rotamers with a ratio ≈ 55:45. δ = 6.64 (s, $1H_{min}$), 6.61 (s, $1H_{maj}$), 6.56 (s, $1H_{maj}$), 6.54 (s, $1H_{min}$), 5.53 (s, $1H_{all}$), 5.16 (dd, J = 10.4, 4.4 Hz, $1H_{min}$), 5.02 (dd, J = 10.5, 4.0 Hz, $1H_{maj}$), 4.20 (ddd, J = 13.4, 6.2, 2.4 Hz, $1H_{maj}$), 3.99 (ddd, J = 13.5, 6.0, 3.0 Hz, $1H_{min}$), 3.86–3.83 (m, $3H_{all}$), 3.71 (s, $3H_{min}$), 3.70 (s, $3H_{maj}$), 3.26 (ddd, J = 13.3, 11.1, 4.3 Hz, $1H_{min}$), 3.19 (ddd, J = 13.3, 11.5, 4.4 Hz, $1H_{maj}$), 2.86 (dddd, J = 34.5, 16.6, 11.3, 6.1 Hz, $1H_{all}$), 2.60 (ddd, J = 16.0, 4.3, 2.7 Hz, $1H_{all}$), 1.75 (ddt, J = 13.3, 10.4, 2.8 Hz, $1H_{all}$), 1.71–1.57 (m, $1H_{all}$), 1.40 (tdd, J = 14.0, 9.1,

4.2 Hz, $1H_{all}$), 1.05 (d, J = 6.5 Hz, $3H_{min}$), 1.01 (d, J = 6.5 Hz, $3H_{maj}$), 0.93 (d, J = 6.7 Hz, $3H_{min}$), 0.92 (d, J = 6.6 Hz, $3H_{maj}$).

^{13}C-NMR (126 MHz, $CDCl_3$): δ = 156.44, 156.41, 145.38, 145.29, 143.99, 131.52, 131.15, 125.45, 125.17, 113.09, 112.73, 111.05, 110.86, 56.08, 52.80, 52.75, 52.71, 52.61, 46.53, 46.29, 37.91, 37.33, 28.25, 27.84, 25.17, 25.03, 23.65, 23.56, 22.35, 21.90.

CI-HRMS: calculated for $C_{16}H_{23}N_1O_4Na_1$ ($[M+Na]^+$): 316.151928, found: 316.151620.

HPLC (OD-3, *n*-heptane/*i*-PrOH 95:5, 298 K, 285 nm): t_R (minor) = 7.5 min, t_R (major) = 9.0 min, er = 96:4 (92% ee).

$[\boldsymbol{\alpha}]_{\boldsymbol{D}}^{\mathbf{25}}$ = −55.3 (*c* = 0.25, $CHCl_3$).

4. Synthesis of Natural Products

(*R*)-Lophocerine

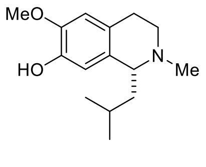

A flame-dried 25 mL flask under argon was charged with Pictet-Spengler product **13** (24.4 mg, 0.0832 mmol, 1.00 eq.) and dry THF (5.0 mL). The solution was cooled to 0 °C and diisobutylaluminum hydride (1.0 M in PhMe, 1.3 mL, 1.3 mmol, 16 eq.) was added slowly. The mixture was then warmed to RT and stirred for 1 h, when full conversion of the starting material was observed by TLC. The reaction was quenched by addition of sat. aq. sodium potassium tartrate (15 mL) and stirred for another 30 min. The phases were separated and the aqueous layer was extracted with DCM (3x). The combined organic layers were dried over anhydrous Na_2SO_4 and concentrated under reduced pressure. Purification by silica gel flash column chromatography (DCM/MeOH 8–10%) gave Lophocerine (19.7 mg, 0.079 mmol, 95%) as a yellow oil.

R_F (DCM/MeOH 9:1) = 0.26.

^{1}H-NMR (501 MHz, $CDCl_3$): δ = 6.61 (s, 1H), 6.54 (s, 1H), 3.85 (s, 3H), 3.46 (dd, J = 8.1, 5.2 Hz, 1H), 3.20 (ddd, J = 12.9, 8.9, 5.3 Hz, 1H), 2.90–2.76 (m, 2H), 2.57–2.47 (m, 1H), 2.45 (s, 3H), 1.85 (ddt, J = 15.0, 13.1, 6.6 Hz, 1H), 1.69 (ddd, J = 13.9, 8.1, 5.7 Hz, 1H), 1.39 (ddd, J = 13.9, 8.3, 5.2 Hz, 1H), 0.97 (d, J = 6.6 Hz, 3H), 0.92 (d, J = 6.7 Hz, 3H).

^{13}C-NMR (126 MHz, $CDCl_3$): δ = 145.17, 143.89, 131.34, 124.80, 113.52, 110.87, 60.83, 56.01, 45.79, 45.49, 41.86, 25.29, 23.43, 23.37, 22.57.

ESI-HRMS: calculated for $C_{15}H_{24}N_1O_2$ ($[M+H]^+$): 250.180154, found: 250.180370.

HPLC (IC-3, (*n*-heptane + 0.1% Et_3N)/*i*-PrOH 70:30, 298 K, 286 nm): t_R (minor) = 3.0 min, t_R (major) = 7.5 min, er = 96:4 (92% ee).

$[\alpha]_D^{25}$ = −12.0 (*c* = 0.12, $CHCl_3$).

(*R*)-6-methoxy-2-methyl-1-(3,4,5-trimethoxyphenethyl)-1,2,3,4-tetrahydroisoquinolin-7-ol (S1)

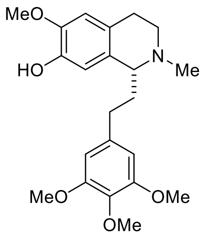

A flame-dried 25 mL flask under argon was charged with Pictet-Spengler product **12** (40.3 mg, 0.0934 mmol, 1.00 eq.) and dry THF (5.0 mL). The solution was cooled to 0 °C and diisobutylaluminum hydride (1.0 M in PhMe, 1.4 mL, 1.4 mmol, 15 eq.) was added slowly. The mixture was then warmed to RT and stirred for 1 h, when full conversion of the starting material was observed by TLC. The reaction was quenched by addition of sat. aq. sodium potassium tartrate (15 mL) and stirred for another 30 min. The phases were separated and the aqueous layer was extracted with DCM (3x). The combined organic layers were dried over anhydrous Na_2SO_4 and concentrated under reduced pressure. Purification by silica gel flash column chromatography (DCM/MeOH 9:1) gave the desired product (31.2 mg, 0.087 mmol, 94%) as a colorless oil. The NMR-spectroscopic data was in agreement with the literature.[1]

R_F (DCM/MeOH 9:1) = 0.34.

^{1}**H-NMR** (501 MHz, $CDCl_3$): δ = 6.67 (s, 1H), 6.55 (s, 1H), 6.40 (s, 2H), 3.85 (s, 3H), 3.83 (s, 6H), 3.81 (s, 3H), 3.41 (t, J = 5.4 Hz, 1H), 3.20–3.09 (m, 1H), 2.80–2.63 (m, 4H), 2.55–2.48 (m, 1H), 2.47 (s, 3H), 2.10–1.98 (m, 2H).

^{13}**C-NMR** (126 MHz, $CDCl_3$): δ = 153.16, 145.12, 143.99, 138.75, 136.05, 130.43, 126.07, 112.97, 110.74, 105.47, 62.78, 60.95, 56.19, 55.99, 48.44, 42.80, 36.72, 32.02, 25.67.

ESI-HRMS: calculated for $C_{22}H_{30}N_1O_5$ ($[M+H]^+$): 388.21185, found: 388.21188.

HPLC (OJ-3, (*n*-heptane + 0.1% Et_3N)/*i*-PrOH 70:30, 298 K, 286 nm): t_R (minor) = 6.9 min, t_R (major) = 9.0 min, er = 94:6 (88% ee).

$[\alpha]_D^{25}$ = −10.9 (*c* = 0.11, $CHCl_3$).

(*R*)-8,9-dimethoxy-1,5,6,10b-tetrahydropyrrolo[2,1-*a*]isoquinolin-3(2*H*)-one (14)

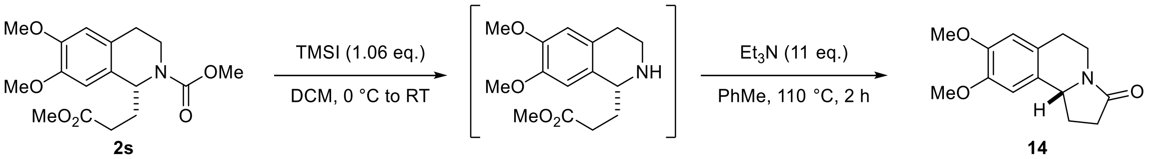

A flame-dried Schlenk under argon was charged with Pictet–Spengler product **2s** (78 mg, 0.23 mmol, 1.0 eq.) and dry DCM (2.5 mL). The solution was cooled to 0 °C and TMSI (35 µL, 0.25 mmol, 1.06 eq.) was added. The reaction vessel was covered in Al-foil and the mixture was stirred overnight while slowly warming to RT. The reaction was quenched by addition of sat. aq. $NaHCO_3$, the aqueous layer was extracted with EtOAc (3x), the combined organic layers were dried over anhydrous Na_2SO_4, and concentrated under reduced pressure.

The crude material was redissolved in dry PhMe (5 mL) in a 25 mL flask equipped with a reflux condenser, Et_3N (0.35mL) was added, and the mixture was heated to reflux for 2 h. After cooling to RT, the reaction was quenched by addition of sat. aq. $NaHCO_3$, the aqueous layer was extracted with EtOAc (3x), the combined organic layers were dried over anhydrous Na_2SO_4, and concentrated under reduced pressure. Purification by silica gel flash column chromatography (DCM/acetone 10–40%) gave unreacted starting material **2s** (23.9 mg, 0.071 mmol, 31%) as well as 8,9-dimethoxy-1,5,6,10b-tetrahydropyrrolo[2,1-a]isoquinolin-3(2H)-one (**14**, 29.5 mg, 0.119 mmol, 52%, 74% brsm) as a yellow oil. The NMR-spectroscopic data was in agreement with the literature.[2]

***R*$_F$** (DCM/acetone 1:1) = 0.47.

^{1}**H-NMR** (501 MHz, $CDCl_3$): δ = 6.61 (s, 1H), 6.56 (s, 1H), 4.71 (t, J = 7.9 Hz, 1H), 4.30 (ddd, J = 12.8, 6.1, 2.0 Hz, 1H), 3.86 (s, 4H), 3.85 (s, 3H), 3.06–2.94 (m, 1H), 2.87 (ddd, J = 17.2, 11.4, 6.1 Hz, 1H), 2.70–2.65 (m, 1H), 2.65–2.60 (m, 1H), 2.60–2.51 (m, 1H), 2.50–2.40 (m, 1H), 1.89–1.76 (m, 1H).

^{13}**C-NMR** (126 MHz, $CDCl_3$): δ = 173.25, 148.26, 148.07, 129.49, 125.68, 111.83, 107.80, 56.68, 56.19, 56.04, 37.18, 31.91, 28.22, 27.89.

EI-HRMS: calculated for $C_{14}H_{17}N_1O_3$ ($[M]^+$): 247.120294, found: 247.120490.

HPLC (OJ-3, *n*-heptane/*i*-PrOH 60:40, 298 K, 283 nm): t_R (minor) = 5.8 min, t_R (major) = 7.1 min, er = 96:4 (92% ee).

$[\alpha]_D^{25}$ = +124.6 (c = 0.18, $CHCl_3$); Lit.:[3] $[\alpha]_D^{25}$ = +175.8 (c = 3.09, $CHCl_3$).

(*R*)-Romneine

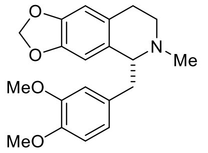

A flame-dried 25 mL flask under argon was charged with Pictet-Spengler product **11** (27.5 mg, 0.0714 mmol, 1.00 eq.) and dry THF (5.0 mL). The solution was cooled to 0 °C and diisobutylaluminum hydride (1.0 M in PhMe, 1.1 mL, 1.1 mmol, 15 eq.) was added slowly. The mixture was then warmed to RT and stirred for 1 h, when full conversion of the starting material was observed by TLC. The reaction was quenched by addition of sat. aq. sodium potassium tartrate (15 mL) and stirred for another 30 min. The phases were separated and the aqueous layer was extracted with DCM (3x). The combined organic layers were dried over anhydrous Na_2SO_4 and concentrated under reduced pressure. Purification by silica gel flash column chromatography (DCM/MeOH 9:1) gave romneine (24.3 mg, 0.071 mmol, >99%) as a colorless oil. The NMR-spectroscopic data was in agreement with the literature.[4]

$\boldsymbol{R_F}$ (DCM/MeOH 9:1) = 0.42.

^{1}H-NMR (501 MHz, $CDCl_3$): δ = 6.76 (d, J = 8.2 Hz, 1H), 6.68 (dd, J = 8.2, 2.0 Hz, 1H), 6.61 (d, J = 2.0 Hz, 1H), 6.53 (s, 1H), 6.26 (s, 1H), 5.88–5.83 (m, 2H), 3.85 (s, 3H), 3.79 (s, 3H), 3.68 (t, J = 6.0 Hz, 1H), 3.17–3.10 (m, 1H), 3.06 (dd, J = 14.0, 5.7 Hz, 1H), 2.86–2.68 (m, 3H), 2.53 (dt, J = 15.4, 4.7 Hz, 1H), 2.49 (s, 3H).

^{13}C-NMR (126 MHz, $CDCl_3$): δ = 148.64, 147.48, 146.01, 145.50, 132.31, 130.49, 127.39, 121.72, 113.01, 111.10, 108.48, 108.04, 100.67, 65.28, 55.97, 55.91, 46.96, 42.69, 41.24, 25.86.

ESI-HRMS: calculated for $C_{20}H_{24}N_1O_4$ ($[M+H]^+$): 342.16998, found: 342.16992.

HPLC (OJ-3, (*n*-heptane + 0.1% Et_3N)/*i*-PrOH 70:30, 298 K, 287 nm): t_R (minor) = 7.0 min, t_R (major) = 8.2 min, er = 96:4 (92% ee).

$[\alpha]_D^{25}$ = −17.9 (c = 0.12, $CHCl_3$).

(*R*)-Laudanosine

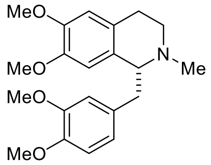

A flame-dried 25 mL flask under argon was charged with Pictet-Spengler product **2c** (37.3 mg, 0.0929 mmol, 1.00 eq.) and dry THF (5.0 mL). The solution was cooled to 0 °C and diisobutylaluminum hydride (1.0 M in PhMe, 1.4 mL, 1.4 mmol, 15 eq.) was added slowly. The mixture was then warmed to RT and stirred for 1 h, when full conversion of the starting material was observed by TLC. The reaction was quenched by addition of sat. aq. sodium potassium tartrate (15 mL) and stirred for another 30 min. The phases were separated and the aqueous layer was extracted with DCM (3x). The combined organic layers were dried over anhydrous Na_2SO_4 and concentrated under reduced pressure. Purification by silica gel flash column chromatography (DCM/MeOH 9:1) gave laudanosine (31.2 mg, 0.087 mmol, 94%) as a white solid. The NMR-spectroscopic data was in agreement with the literature.[5]

R**F** (DCM/MeOH 9:1) = 0.37.

^{1}H-NMR (501 MHz, $CDCl_3$): δ = 6.76 (d, J = 8.1 Hz, 1H), 6.63 (dd, J = 8.1, 2.0 Hz, 1H), 6.59 (d, J = 2.0 Hz, 1H), 6.55 (s, 1H), 6.05 (s, 1H), 3.83 (s, 3H), 3.83 (s, 3H), 3.78 (s, 3H), 3.70 (dd, J = 7.9, 4.8 Hz, 1H), 3.56 (s, 3H), 3.21–3.11 (m, 2H), 2.87–2.73 (m, 3H), 2.58 (dt, J = 15.6, 4.7 Hz, 1H), 2.54 (s, 3H).

^{13}C-NMR (126 MHz, $CDCl_3$): δ = 148.69, 147.47, 147.43, 146.47, 132.53, 129.20, 126.04, 121.99, 113.16, 111.32, 111.23, 111.15, 64.97, 56.03, 55.93, 55.88, 55.67, 47.05, 42.73, 40.97, 25.58.

ESI-HRMS: calculated for $C_{21}H_{28}N_1O_4$ ($[M+H]^+$): 358.20128, found: 358.20111.

HPLC (AD-3, (*n*-heptane + 0.1% Et_3N)/*i*-PrOH 80:20, 298 K, 282 nm): t_R (minor) = 5.9 min, t_R (major) = 6.5 min, er = 97.5:2.5 (95% ee).

$[\alpha]_D^{25}$ = −46.5 (*c* = 0.20, $CHCl_3$).

(*R*)-1-(benzo[*d*][1,3]dioxol-5-ylmethyl)-6,7-dimethoxy-2-methyl-1,2,3,4-tetrahydroisoquinoline (15)

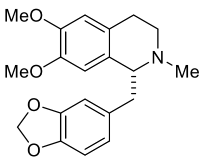

A flame-dried 50 mL flask under argon was charged with Pictet-Spengler product **2b** (99.0 mg, 0.257 mmol, 1.00 eq.) and dry THF (15 mL). The solution was cooled to 0 °C and diisobutylaluminum hydride (1.0 M in PhMe, 3.8 mL, 3.8 mmol, 15 eq.) was added slowly. The mixture was then warmed to RT and stirred for 1 h, when full conversion of the starting material was observed by TLC. The reaction was quenched by addition of sat. aq. sodium potassium tartrate (20 mL) and stirred for another 30 min. The phases were separated and the aqueous layer was extracted with DCM (3x). The combined organic layers were dried over anhydrous Na_2SO_4 and concentrated under reduced pressure. Purification by silica gel flash column chromatography (DCM/MeOH 9:1) gave the desired product (80.2 mg, 0.235 mmol, 91%) as a white solid. The NMR-spectroscopic data was in agreement with the literature.[6]

R**F** (DCM/MeOH 9:1) = 0.49.

^{1}H-NMR (501 MHz, $CDCl_3$): δ = 6.70 (d, J = 7.9 Hz, 1H), 6.64 (d, J = 1.7 Hz, 1H), 6.57–6.53 (m, 2H), 6.11 (s, 1H), 5.92–5.88 (m, 2H), 3.84 (s, 3H), 3.66 (dd, J = 7.4, 5.4 Hz, 1H), 3.62 (s, 3H), 3.17 (ddd, J = 12.5, 8.9, 5.1 Hz, 1H), 3.09 (dd, J = 13.8, 5.4 Hz, 1H), 2.83 (ddd, J = 15.1, 8.9, 5.7 Hz, 1H), 2.79–2.71 (m, 2H), 2.59 (dt, J = 15.8, 4.6 Hz, 1H), 2.51 (s, 3H).

^{13}C-NMR (126 MHz, $CDCl_3$): δ = 147.49, 147.45, 146.57, 145.85, 133.93, 129.30, 126.08, 122.75, 111.36, 111.14, 110.26, 108.07, 100.85, 65.02, 55.89, 55.73, 46.93, 42.74, 41.17, 25.44.

ESI-HRMS: calculated for $C_{20}H_{24}N_1O_4$ ($[M+H]^+$): 342.16998, found: 342.17006.

HPLC (AD-3, (*n*-heptane + 0.1% Et_3N)/*i*-PrOH 80:20, 298 K, 282 nm): t_R (minor) = 6.1 min, t_R (major) = 5.4 min, er = 97:3 (94% ee).

$[\alpha]_D^{25}$ = −67.1 (*c* = 0.29, $CHCl_3$).

(*R*)-Amurine

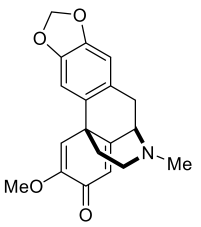

A 10 mL IKA ElectraSyn vial was charged with 1-(benzo[d][1,3]dioxol-5-ylmethyl)-6,7-dimethoxy-2-methyl-1,2,3,4-tetrahydroisoquinoline (**15**, 34.1 mg, 0.10 mmol, 1.0 eq.), HPLC grade CH_3CN (10.0 mL), and HBF_4 (48 wt% in H_2O, 52 µL, 0.40 mmol, 4.0 eq.). The mixture was mounted on an ElectraSyn GOGO module equipped with a BDD anode (approx. 4.2 cm^2 submerged area) and a Pt cathode. The reaction was cooled to 0 °C and constant current electrolysis was performed (J = 0.75 mA/cm^2, I = 3.1 mA, z = 2.2 F). After complete reaction, the mixture was diluted with EtOAc (50 mL) and quenched with sat. aq. $NaHCO_3$. The aqueous layer was extracted with EtOAc (3x), and the combined organic layers were dried over anhydrous Na_2SO_4 and concentrated under reduced pressure. Purification by silica gel flash column chromatography (EtOAc/Et_3N 4:1) gave amurine (24.5 mg, 0.075 mmol, 75%) as a yellow foam. The NMR-spectroscopic data was in agreement with the literature.[7]

***R*F** (EtOAc/Et_3N 4:1) = 0.35.

^{1}H-NMR (501 MHz, $CDCl_3$): δ = 6.82 (s, 1H), 6.59 (s, 1H), 6.30 (s, 1H), 6.28 (s, 1H), 5.93 (d, J = 1.4 Hz, 1H), 5.90 (d, J = 1.4 Hz, 1H), 3.78 (s, 3H), 3.65 (d, J = 6.1 Hz, 1H), 3.29 (d, J = 17.9 Hz, 1H), 2.98 (dd, J = 17.9, 6.2 Hz, 1H), 2.61–2.52 (m, 2H), 2.44 (s, 3H), 1.91 (ddd, J = 12.7, 10.8, 6.5 Hz, 1H), 1.80 (dt, J = 12.5, 2.6 Hz, 1H).

^{13}C-NMR (126 MHz, $CDCl_3$): δ = 181.03, 161.44, 151.49, 147.01, 146.89, 131.11, 129.69, 122.31, 118.87, 107.63, 105.24, 101.31, 60.83, 55.17, 45.77, 42.55, 41.82, 41.35, 33.04.

ESI-HRMS: calculated for $C_{19}H_{20}N_1O_4$ ($[M+H]^+$): 326.13868, found: 326.13901.

HPLC (IE-3, (*n*-heptane + 0.1% Et_3N)/*i*-PrOH 50:50, 298 K, 290 nm): t_R (minor) = 19.1 min, t_R (major) = 11.9 min, er = 96.5:3.5 (93% ee).

$[\alpha]_D^{25}$ = +5.5 (c = 0.18, $CHCl_3$).

(*R*)-Xylopinine

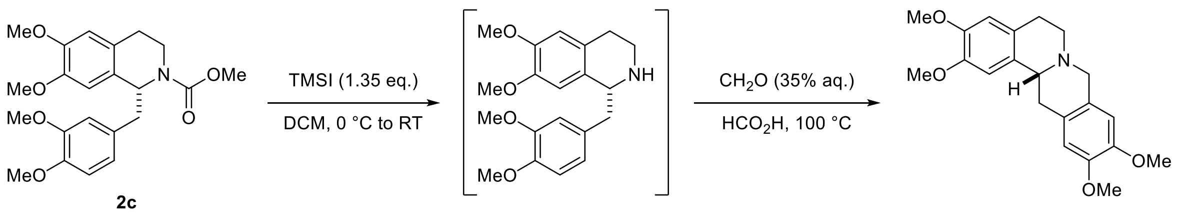

2c

A flame-dried Schlenk under argon was charged with Pictet–Spengler product **2c** (67.0 mg, 0.167 mmol, 1.00 eq.) and dry DCM (3.5 mL). The mixture was cooled to 0 °C and TMSI (32 µL, 0.22 mmol, 1.4 eq.) was added. The reaction vessel was covered in Al-foil and the mixture was stirred overnight while slowly warming to RT. The reaction was quenched by addition of sat. aq. $NaHCO_3$, the aqueous layer was extracted with EtOAc (3x), the combined organic layers were dried over anhydrous Na_2SO_4, and concentrated under reduced pressure.

The crude material was redissolved in formic acid (98%, 0.75 mL) in a 5 mL vial, aq. CH_2O (35 wt%, 0.42 mL, 5.3 mmol, 32 eq.) was added, and the mixture was heated to 100 °C for 1 h. After cooling to

RT, the reaction was quenched by addition of sat. aq. $NaHCO_3$ and the aqueous layer was extracted with EtOAc (3x). The combined organic layers were dried over anhydrous Na_2SO_4 and concentrated under reduced pressure. Purification by silica gel flash column chromatography (EtOAc/Et_3N 99:1) gave the desired product (45.1 mg, 0.127 mmol, 76%) as a white solid. The NMR-spectroscopic data was in agreement with the literature.[8]

***R*F** (EtOAc/Et_3N 99:1) = 0.43.

^{1}H-NMR (501 MHz, $CDCl_3$): δ = 6.74 (s, 1H), 6.66 (s, 1H), 6.61 (s, 1H), 6.57 (s, 1H), 3.94 (d, J = 14.5 Hz, 1H), 3.88 (s, 3H), 3.86 (s, 3H), 3.85 (s, 4H), 3.84 (s, 3H), 3.67 (d, J = 14.5 Hz, 1H), 3.58 (dd, J = 11.3, 3.9 Hz, 1H), 3.24 (dd, J = 15.9, 4.0 Hz, 1H), 3.18–3.09 (m, 2H), 2.83 (dd, J = 15.5, 11.5 Hz, 1H), 2.69–2.57 (m, 2H).

^{13}C-NMR (126 MHz, $CDCl_3$): δ = 147.73, 147.59, 147.54, 147.50, 129.89, 126.85, 126.46, 126.40, 111.49, 111.46, 109.13, 108.65, 59.70, 58.35, 56.15, 56.05, 56.01, 55.94, 51.47, 36.52, 29.16.

EI-HRMS: calculated for $C_{21}H_{25}N_1O_4$ ($[M]^+$): 355.177809, found: 355.178410.

HPLC (AD-3, (*n*-heptane + 0.1% Et_3N)/*i*-PrOH 60:40, 298 K, 283 nm): t_R (minor) = 19.8 min, t_R (major) = 6.3 min, er = 90:10 (80% ee).

$[\alpha]_D^{25}$ = +159.4 (*c* = 0.35, $CHCl_3$); Lit. (for the opposite enantiomer):[9] $[\alpha]_D^{25}$ = −280 (*c* = 0.1, $CHCl_3$).

(*S*)-Calycotomine

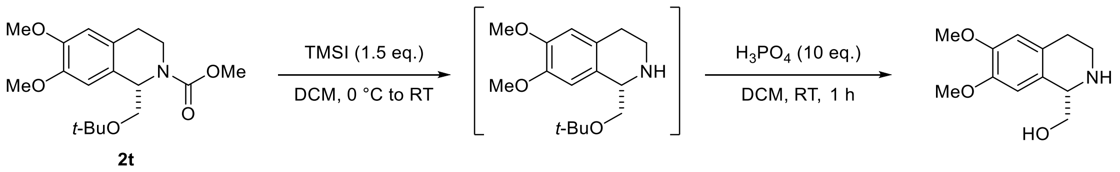

A flame-dried Schlenk under argon was charged with Pictet–Spengler product **2t** (95.1 mg, 0.282 mmol, 1.00 eq.) and dry DCM (5.5 mL). The mixture was cooled to 0 °C and TMSI (60 µL, 0.42 mmol, 1.5 eq.) was added. The reaction vessel was covered in Al-foil and the mixture was stirred overnight while slowly warming to RT. The reaction was quenched by addition of sat. aq. $NaHCO_3$, the aqueous layer was extracted with EtOAc (3x), the combined organic layers were dried over anhydrous Na_2SO_4, and concentrated under reduced pressure.

The crude material was redissolved in DCM (5.5 mL) in a 10 mL vial, aq. H_3PO_4 (85 wt%, 0.2 mL, 3.0 mmol, 10 eq.) was added, and the mixture was stirred vigorously at RT for 1 h. The reaction was quenched by addition of sat. aq. Na_2CO_3 and the aqueous layer was extracted with EtOAc (3x). The combined organic layers were washed with brine, dried over anhydrous Na_2SO_4, and concentrated under reduced pressure. The crude material was redissolved in DCM (25 mL) and extracted with aq. HCl (10 w%, 3x). The combined HCl-layers were washed with DCM, basified with solid NaOH, and reextracted with DCM (3x). The combined organic extracts were dried over anhydrous Na_2SO_4 and concentrated under reduced pressure to give calycotomine (41.7 mg, 0.187 mmol, 66%) as a white solid.

^{1}**H-NMR** (501 MHz, CD_3OD): δ = 6.75 (s, 1H), 6.68 (s, 1H), 3.92 (dd, J = 8.4, 4.0 Hz, 1H), 3.80 (dd, J = 11.2, 4.1 Hz, 1H), 3.78 (s, 2H), 3.78 (s, 3H), 3.72 (dd, J = 11.1, 8.4 Hz, 1H), 3.17 (ddd, J = 12.3, 7.2, 5.2 Hz, 1H), 2.92 (dt, J = 11.8, 5.6 Hz, 1H), 2.81–2.67 (m, 2H).

^{13}**C-NMR** (126 MHz, CD_3OD): δ = 149.35, 148.79, 129.06, 128.16, 113.51, 111.42, 65.16, 57.99, 56.58, 56.40, 40.35, 29.49.

ESI-HRMS: calculated for $C_{12}H_{18}N_1O_3$ ($[M+H]^+$): 224.12812, found: 224.12816.

HPLC was measured after derivatization to the *N*-Boc protected amine according to a literature procedure[10] (IA-3, *n*-heptane/*i*-PrOH 70:30, 298 K, 282 nm): t_R (minor) = 6.2 min, t_R (major) = 5.2 min, er = 91.5:8.5 (83% ee).

$[\alpha]_D^{25}$ = −9.8 (*c* = 0.16, $CHCl_3$); Lit.:[11] $[\alpha]_D^{25}$ = −15.0 (*c* = 0.18, $CHCl_3$).

(*R*)-Salsolidine hydrochloride

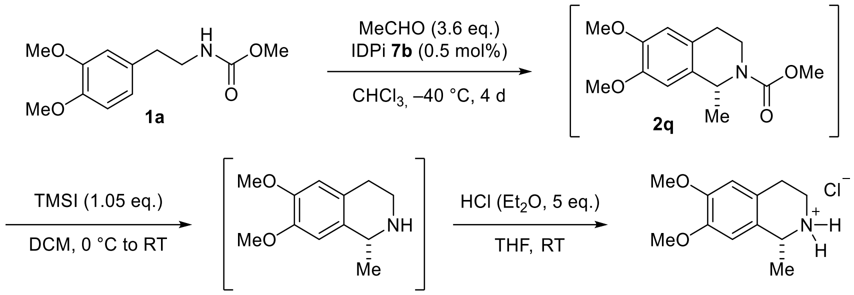

A flame-dried 100 mL Young Schlenk under argon was charged with carbamate **1a** (1.20 g, 5.00 mmol, 1.00 eq.) and IDPi catalyst **7b** (54.8 mg, 0.025 mmol, 0.5 mol%). Dry $CHCl_3$ (50 mL) was added and the solution was cooled to –78 °C. Acetaldehyde (1.0 mL, 18 mmol, 3.6 eq.) was added to the reaction, the Schlenk was closed, and the mixture was stirred in a Dewar filled with EtOH maintained at –40 °C by the aid of a cryostat. After 4 d reaction time, the mixture was warmed to RT and stirred for another 2 h. The reaction was then quenched by addition of sat. aq. $NaHCO_3$ (100 mL), the aqueous layer was extracted with DCM (5x), the combined organic layers were dried over anhydrous Na_2SO_4, concentrated under reduced pressure, and azeotroped from PhMe (3x). An aliquot of the crude material was analyzed by ^{1}H-NMR to confirm quantitative formation of the Pictet-Spengler product. A small sample was taken for determination of the enantiomeric excess by HPLC.

TLC of the crude reaction mixture (hex/EtOAc 1:1). UV (left), CAM-stain (right)	HPLC-trace of the crude reaction mixture (IC-3R, MeOH/H_2O 90:10, 298 K, 284 nm): t_R (minor) = 6.3 min, t_R (major) = 7.9 min, er = 99:1 (98% ee).

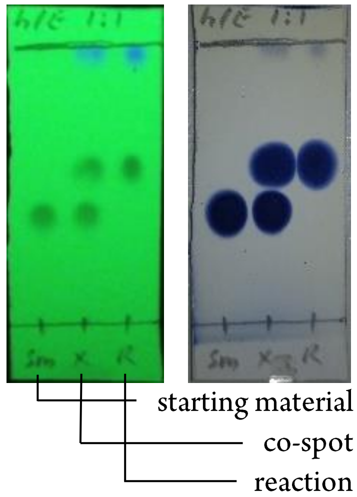

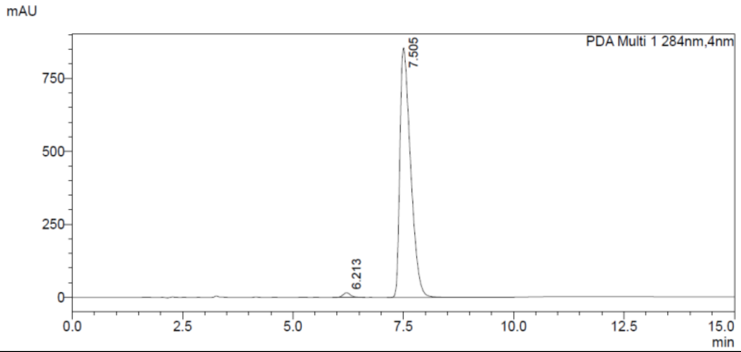

IC-3R, MeOH/H_2O 90:10, 298 K, 284 nm		
**peak #**	**t_R / min**	**area / %**
1	6.213	1.247
2	7.505	98.753

The crude material was dissolved in dry DCM (50 mL) in a flame-dried Schlenk under argon and cooled to 0 °C. TMSI (0.75 mL, 5.3 mmol, 1.05 eq.) was added slowly and the mixture was allowed to warm to RT over the course of 16 h. The reaction was quenched by addition sat. aq. $NaHCO_3$ (50 mL) and the aqueous layer was extracted with DCM (5x). The combined organic layers were extracted with aqueous HCl (1.2 M, 5x), dried over anhydrous Na_2SO_4, and concentrated under reduced pressure. Purification by silica gel flash column chromatography (DCM/EtOAc 100:0 to 19:1) gave remaining Pictet-Spengler product **2q** (216 mg, 0.815 mmol, 16%) as well as, after filtration over DOWEX, recovered IDPi catalyst **7b** (53.7 mg, 0.024 mmol, 98%). The combined HCl-layers were basified by

addition of solid NaOH and extracted with MTBE (5x). The combined MTBE layers were dried over anhydrous Na_2SO_4 and concentrated under reduced pressure, to give 1.17 g of crude material.

The crude material was dissolved in dry THF (25 mL) and HCl (2.0 M in Et_2O, 12.5 mL, 25 mmol, 5 eq.) was added under vigorous stirring. A white precipitate formed immediately and the flask was placed in the freezer at –20 °C overnight. After warming to RT, the solid was collected by filtration (under gentle stream of argon to prevent product solubilizing in condensed water) and washed with Et_2O to give salsolidine hydrochloride (852 mg, 3.49 mmol, 70%) as a white solid. The NMR-spectroscopic data was in agreement with the literature.[12]

A crystalline sample for x-ray single crystal structure analysis was obtained by dissolving an aliquot of the product in a small amount of water and layering with THF.

^{1}H-NMR (501 MHz, DMSO-d_6): δ = 9.80 (s, 1H), 9.31 (s, 1H), 6.83 (s, 1H), 6.77 (s, 1H), 4.42 (q, J = 7.0 Hz, 1H), 3.74 (s, 3H), 3.73 (s, 3H), 3.41–3.34 (m, 1H), 3.27–3.17 (m, 1H), 2.97 (dt, J = 17.0, 6.4 Hz, 1H), 2.87 (dt, J = 16.9, 5.9 Hz, 1H), 1.58 (d, J = 6.8 Hz, 3H).

^{13}C-NMR (126 MHz, DMSO-d_6): δ = 148.14, 147.70, 125.82, 123.64, 111.68, 109.56, 55.72, 55.52, 49.87, 38.19, 24.58, 19.18.

ESI-HRMS: calculated for $C_{12}H_{18}N_1O_2$ ($[M\text{-}Cl]^+$): 208.133204, found: 208.133230.

HPLC was measured after derivatization to methyl carbamate **2q** (IC-3R, MeOH/H_2O 90:10, 298 K, 284 nm): t_R (minor) = 6.1 min, t_R (major) = 7.6 min, er = 96.5:3.5 (93% ee).

$[\alpha]_D^{25}$ = +22.7 (c = 1.21, H_2O); Lit.:[13] $[\alpha]_D^{25}$ = +24.1 (c = 1.8, H_2O).

5. Synthesis of Protected *β*-arylethylamines

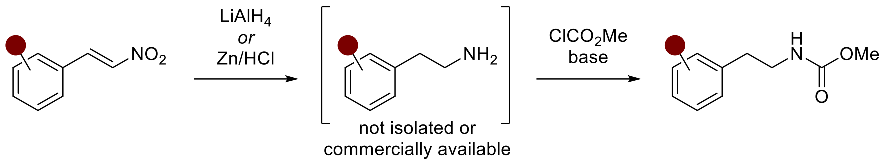

methyl (3,4-dimethoxyphenethyl)carbamate (1a)

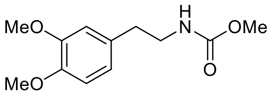

A 500 mL round-bottom flask equipped with a stir bar was charged with 3,4-dimethoxyphenethylamine (8.5 mL, 50 mmol, 1.0 eq.), Na_2CO_3 (13.3 g, 126 mmol, 2.5 eq.), THF (100 mL), and water (100 mL). Methyl chloroformate (4.6 mL, 60 mmol, 1.2 eq.) was added dropwise, and the mixture was vigorously stirred at RT for 16 h. The reaction was quenched by addition of aqueous HCl (1.2 M, 100 mL) and EtOAc (50 mL). The phases were separated and the organic layer was washed with aqueous HCl (1.2 M, 3x) and brine, dried over anhydrous Na_2SO_4, and concentrated. Purification by silica gel flash column chromatography (hex/EtOAc 20–40%) yielded the desired product as a slowly solidifying colorless oil (9.93 g, 41.5 mmol, 82%).

R_F (hex/EtOAc 2:1) = 0.19.

^{1}H-NMR (501 MHz, $CDCl_3$): δ = 6.81 (d, J = 8.0 Hz, 1H), 6.73 (dd, J = 8.2, 2.0 Hz, 1H), 6.71–6.69 (m, 1H), 4.68 (bs, 1H), 3.87 (s, 3H), 3.86 (s, 3H), 3.66 (bs, 3H), 3.42 (q, J = 6.7 Hz, 2H), 2.75 (t, J = 7.0 Hz, 2H).

^{13}C-NMR (126 MHz, $CDCl_3$): δ = 157.12, 149.21, 147.87, 131.40, 120.83, 112.09, 111.55, 56.08, 56.01, 52.19, 42.46, 35.88.

EI-HRMS: calculated for $C_{12}H_{17}N_1O_4$ ($[M]^+$): 239.115209, found: 239.115470.

tert-butyl (3,4-dimethoxyphenethyl)carbamate (1b)

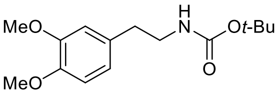

A flame-dried 100 mL two-necked flask under argon equipped with a stir bar was charged with di-*tert*-butyl dicarbonate (7.8 mL, 34 mmol, 1.0 eq.) and THF (50 mL). 3,4-dimethoxyphenethylamine (8.6 mL, 51 mmol, 1.5 eq.) was added dropwise and the mixture was allowed to reach RT while stirring for 6 h. The reaction was quenched by addition of sat. aq. $NaHCO_3$ (100 mL) and the aqueous layer was extracted with EtOAc (3x). The combined organic phases were washed with aqueous HCl (1.2 M, 3x), saturated $NaHCO_3$, and brine, dried over anhydrous Na_2SO_4, and concentrated. Crystallization from hex/EtOAc yielded the desired product as a white solid (6.8 g, 24 mmol, 71%).

^{1}H-NMR (501 MHz, CD_2Cl_2): δ = 6.80 (d, J = 8.5 Hz, 1H), 6.75–6.70 (m, 2H), 4.61 (bs, 1H), 3.82 (s, 3H), 3.80 (s, 3H), 3.31 (q, J = 6.8 Hz, 2H), 2.71 (t, J = 7.1 Hz, 2H), 1.41 (s, 9H).

^{13}C-NMR (126 MHz, CD_2Cl_2): δ = 156.09, 149.61, 148.24, 132.18, 121.06, 112.77, 112.11, 79.16, 56.24, 56.12, 42.30, 36.08, 28.53.

EI-HRMS: calculated for $C_{15}H_{23}N_1O_4$ ($[M]^+$): 281.162159, found: 281.162240.

benzyl (3,4-dimethoxyphenethyl)carbamate (1c)

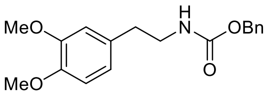

A 250 mL flask equipped with a stir bar was charged with dimethoxyphenethylamine (1.7 mL, 10 mmol, 1.0 eq.), K_2CO_3 (1.66 g, 12 mmol, 1.2 eq.) and THF (50 mL). Benzyl chloroformate (1.6 mL, 11 mmol, 1.1 eq.) was added dropwise and the mixture was stirred at RT for 1 h. The reaction was then filtered and concentrated. Crystallization from hex/THF yielded the desired product as a white solid (1.0 g, 3.2 mmol, 32%).

^{1}H-NMR (501 MHz, $CDCl_3$): δ = 7.39–7.28 (m, 5H), 6.79 (d, J = 8.0 Hz, 1H), 6.74–6.62 (m, 2H), 5.10 (s, 2H), 4.77 (bs, 1H), 3.85 (s, 3H), 3.84 (s, 3H), 3.44 (q, J = 6.8 Hz, 2H), 2.76 (t, J = 7.0 Hz, 2H).

^{13}C-NMR (126 MHz, $CDCl_3$): δ = 156.45, 149.19, 147.86, 136.72, 131.33, 128.66, 128.25, 120.82, 112.08, 111.55, 66.77, 56.07, 55.97, 42.46, 35.80.

EI-HRMS: calculated for $C_{18}H_{21}N_1O_4$ ($[M]^+$): 315.146509, found: 315.146420.

phenyl (3,4-dimethoxyphenethyl)carbamate (1d)

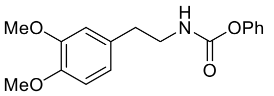

A 100 mL flask equipped with a stir bar was charged with diphenyl carbonate (4.3 g, 20 mmol, 1.0 eq.), H_2O (36 mL) and THF (5 mL). Dimethoxyphenethylamine (3.4 mL, 20 mmol, 1.0 eq.) was added dropwise and the mixture was stirred at RT for 16 h. The aqueous layer was extracted with EtOAc (3x50 mL). The combined organic layers were washed with NaOH (2 M) and brine, dried over anhydrous Na_2SO_4, and concentrated. Purification by silica gel flash column chromatography (hex/EtOAc 2:1 to 1:1) and crystallization from hex/EtOAc yielded the desired product as a white crystalline solid (5.0 g, 17 mmol, 82%).

^{1}H-NMR (501 MHz, $CDCl_3$): δ = 7.35 (t, J = 8.0 Hz, 2H), 7.19 (t, J = 6.9 Hz, 1H), 7.10 (d, J = 7.4 Hz, 2H), 6.84 (d, J = 8.1 Hz, 1H), 6.79–6.73 (m, 2H), 5.04 (bs, 1H), 3.89 (s, 3H), 3.88 (s, 3H), 3.51 (q, J = 6.7 Hz, 2H), 2.84 (t, J = 7.0 Hz, 2H).

^{13}C-NMR (126 MHz, $CDCl_3$): δ = 154.70, 151.15, 149.28, 147.97, 131.18, 129.42, 125.43, 121.69, 120.86, 112.12, 111.62, 56.10, 56.04, 42.61, 35.65.

EI-HRMS: calculated for $C_{17}H_{19}N_1O_4$ ($[M]^+$): 301.130859, found: 301.130390.

methyl (3-methoxyphenethyl)carbamate (S2)

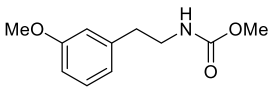

A 250 mL round-bottom flask equipped with a stir bar was charged with 3-methoxyphenethylamine (3.0 mL, 21 mmol, 1.0 eq.), THF (100 mL), and Et_3N (8.6 mL, 62 mmol, 3.0 eq.). Methyl chloroformate (1.8 mL, 23 mmol, 1.1 eq.) was added dropwise, and the mixture was stirred at RT for 16 h. The reaction was quenched by addition of aqueous HCl (1.2 M, 100 mL) and EtOAc (50 mL). The phases were separated and the organic layer was washed with aqueous HCl (1.2 M, 3x) and brine, dried over anhydrous Na_2SO_4, and concentrated. Purification by silica gel flash column chromatography (hex/EtOAc 4:1) yielded the desired product as a colorless oil (3.51 g, 16.8 mmol, 82%).

R_F (hex/EtOAc 4:1) = 0.20.

^{1}H-NMR (501 MHz, CD_2Cl_2): δ = 7.21 (t, J = 7.8 Hz, 1H), 6.81–6.71 (m, 3H), 4.79 (bs, 1H), 3.78 (s, 3H), 3.61 (s, 3H), 3.40 (q, J = 6.8 Hz, 2H), 2.77 (t, J = 7.1 Hz, 2H).

^{13}C-NMR (126 MHz, CD_2Cl_2): δ = 160.28, 157.19, 141.04, 129.88, 121.42, 114.84, 112.10, 55.50, 52.21, 42.51, 36.53.

EI-HRMS: calculated for $C_{11}H_{15}N_1O_3$ ($[M]^+$): 209.104643, found: 209.104900.

2-(3-bromo-4,5-dimethoxyphenyl)ethan-1-amine (S3)

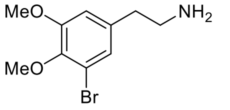

The reaction was performed according to a literature procedure.[14] A 500 mL round-bottom flask equipped with a stir bar was charged with MeOH (42 mL), and cooled to −10 °C (acetone/ice). (*E*)-1-bromo-2,3-dimethoxy-5-(2-nitrovinyl)benzene[14] (5.7 g, 20 mmol, 1.0 eq.), zinc powder (22.8 g, 349 mmol, 17.6 eq.), and conc. aq. HCl (58 mL) were added in alternating small portions over the course of 30 min. The reaction was warmed to 0 °C after 15 min and then stirred for a total of 8 h. The mixture was subsequently filtered through filter paper while cooling the receiving flask to 0 °C. The liquid was basified with solid NaOH, dried over anhydrous Na_2SO_4, and concentrated to yield the desired product as a yellow oil (4.66 g). The crude material was used for the next step without further purification.

methyl (3-bromo-4,5-dimethoxyphenethyl)carbamate (S4)

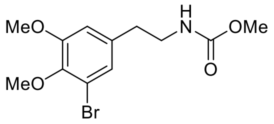

A 250 mL round-bottom flask equipped with a stir bar was charged with crude 2-(3-bromo-4,5-dimethoxyphenyl)ethan-1-amine (**S3**, 4.66 g, 17.9 mmol, 1.0 eq.), THF (100 mL), and Et_3N (11 mL, 79 mmol, 4.4 eq.). Methyl chloroformate (2.2 mL, 29 mmol, 1.6 eq.) was added dropwise, and the mixture was stirred at RT for 16 h. The reaction was quenched by addition of aqueous HCl (1.2 M, 50 mL) and EtOAc (50 mL). The phases were separated and the organic layer was washed with aqueous HCl (1.2 M, 3x) and brine, dried over anhydrous Na_2SO_4, and concentrated. Purification by silica gel flash column chromatography (hex/EtOAc 3:1 to 2:1) and another silica gel flash column chromatography (DCM/EtOAc 2.5-10%) yielded the desired product as a yellow solid (3.44 g, 10.8 mmol, 55% over two steps). The NMR-spectroscopic data was in agreement with the literature.[15]

^{1}H-NMR (501 MHz, $CDCl_3$): δ = 6.95 (d, J = 1.9 Hz, 1H), 6.69–6.65 (m, 1H), 4.74 (s, 1H), 3.84 (s, 3H), 3.82 (s, 3H), 3.66 (s, 3H), 3.40 (q, J = 6.8 Hz, 2H), 2.73 (t, J = 7.0 Hz, 2H).

^{13}C-NMR (126 MHz, $CDCl_3$): δ = 157.08, 153.80, 145.21, 136.12, 124.78, 117.76, 112.40, 60.68, 56.23, 52.23, 42.17, 35.87.

ESI-HRMS: calculated for $C_{12}H_{17}N_1O_4Br_1$ ($[M+H]^+$): 318.033559, found: 318.033500.

methyl (2-(benzo[*d*][1,3]dioxol-5-yl)ethyl)carbamate (S5)

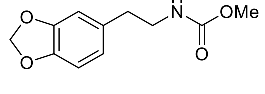

A 100 mL round-bottom flask equipped with a stir bar was charged with 3,4-methylenedioxyphenethylamine hydrochloride (2.0 g, 9.9 mmol, 1.0 eq.), THF (50 mL), and Et_3N (5.5 mL, 39 mmol, 4.0 eq.). Methyl chloroformate (0.85 mL, 11 mmol, 1.1 eq.) was added dropwise, and the mixture was stirred at RT for 2 h. The reaction was quenched by addition of aqueous HCl (1.2 M, 50 mL) and EtOAc (50 mL). The phases were separated and the organic layer was washed with aqueous HCl (1.2 M, 3x) and brine, dried over

anhydrous Na_2SO_4, and concentrated. Purification by silica gel flash column chromatography (pentane/EtOAc 30–40%) yielded the desired product as a white solid (1.0 g, 4.7 mmol, 47%).

R**F** (hex/EtOAc 2:1) = 0.39.

^{1}H-NMR (501 MHz, CD_2Cl_2): δ = 6.74 (d, J = 7.9 Hz, 1H), 6.69 (d, J = 1.7 Hz, 1H), 6.64 (dd, J = 7.9, 1.7 Hz, 1H), 5.92 (s, 2H), 4.73 (bs, 1H), 3.61 (s, 3H), 3.34 (q, J = 6.7 Hz, 2H), 2.70 (t, J = 7.0 Hz, 2H).

^{13}C-NMR (126 MHz, CD_2Cl_2): δ = 157.17, 148.22, 146.59, 133.19, 122.06, 109.41, 108.57, 101.48, 52.23, 42.79, 36.21.

EI-HRMS: calculated for $C_{11}H_{13}N_1O_4$ ($[M]^+$): 223.083909, found: 223.083930.

4-(2-aminoethyl)-2-methoxyphenol (S6)

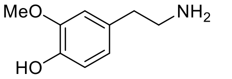

A flame-dried 250 mL round-bottom flask under argon equipped with a stir bar and a reflux condenser was charged with $LiAlH_4$ (1.0 M in THF, 55 mL, 55 mmol, 4.0 eq.) and cooled to 0 °C. (*E*)-2-methoxy-4-(2-nitrovinyl)phenol[16] (2.7 g, 14 mmol, 1.0 eq.) was dissolved in dry THF (25 mL) and added slowly to the reaction mixture. After complete addition, the mixture was heated to reflux for 3 h. The reaction was cooled to 0 °C and quenched by careful addition of water (25 mL). Sat. aq. potassium sodium tartrate (100 mL) was added and the aqueous layer was extracted with EtOAc (5x). The combined organic layers were dried over anhydrous Na_2SO_4 and concentrated to yield a brown oil that was used for the next step without further purification.

methyl (4-hydroxy-3-methoxyphenethyl)carbamate (S7)

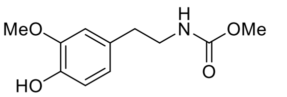

A 250 mL round-bottom flask equipped with a stir bar was charged with crude 4-(2-aminoethyl)-2-methoxyphenol (**S6**, 14 mmol, 1.0 eq.), Et_2O (25 mL), water (25 mL), and Na_2CO_3 (4.5 g, 42 mmol, 3.1 eq.). Methyl chloroformate (1.1 mL, 14 mmol, 1.0 eq.) was added dropwise, and the mixture was stirred at RT for 3 h. The reaction was diluted with EtOAc (100 mL) and quenched by addition of aqueous HCl (1.2 M, 100 mL). The phases were separated and the organic layer was washed with aqueous HCl (1.2 M, 3x) and brine, dried over anhydrous Na_2SO_4, and concentrated. Purification by silica gel flash column chromatography (hex/EtOAc 30–50%) and automated reversed phase column chromatography ($MeOH/H_2O$ 60:40 to 100:0) yielded the desired product as a white solid (598 mg, 2.65 mmol, 19% over two steps). The NMR-spectroscopic data was in agreement with the literature.[17]

^{1}H-NMR (501 MHz, $CDCl_3$): δ = 6.85 (d, J = 8.1 Hz, 1H), 6.71–6.66 (m, 2H), 5.51 (s, 1H), 4.67 (s, 1H), 3.88 (s, 3H), 3.66 (s, 3H), 3.41 (q, J = 6.7 Hz, 2H), 2.74 (t, J = 7.0 Hz, 2H).

^{13}C-NMR (126 MHz, CD_2Cl_2): δ = 157.22, 147.09, 144.68, 131.23, 121.69, 114.57, 111.80, 56.33, 52.21, 42.79, 36.15.

EI-HRMS: calculated for $C_{11}H_{15}N_1O_4$ ($[M]^+$): 225.099559, found: 225.099420.

6. Synthesis of Aldehydes

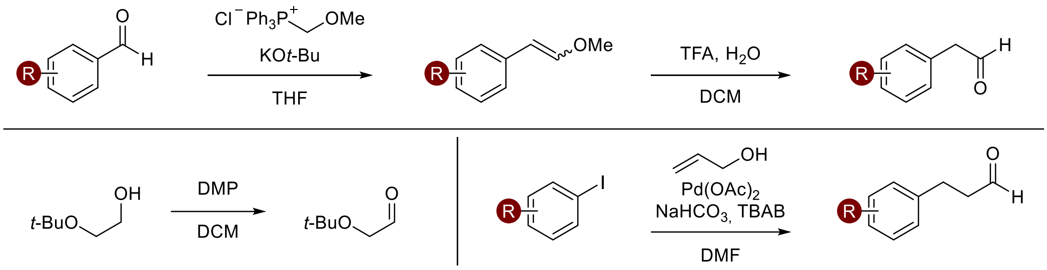

5-(2-methoxyvinyl)benzo[*d*][1,3]dioxole (S8)

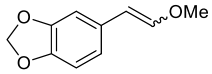

A flame-dried 250 mL three-necked flask under argon was charged with piperonal (1.50 g, 10 mmol, 1.0 eq.), (methoxymethyl)triphenylphosphonium chloride (4.1 g, 12 mmol, 1.2 eq.) and dry THF (50 mL). The suspension was cooled to −10 °C (acetone/ice) and KO*t*-Bu (1.35 g, 12.0 mmol, 1.20 eq.) was added in one portion. The mixture was warmed to RT and stirred for 16 h. The reaction was quenched by addition of sat. aq. NH_4Cl and the aqueous layer was extracted with MTBE (3x). The combined organic layers were washed with brine, dried over anhydrous Na_2SO_4, and concentrated under reduced pressure. Purification by silica gel flash column chromatography (hex/MTBE 19:1 to 9:1) yielded the product (1.33 g, 7.47 mmol, 75%, $E/Z \approx 55{:}45$) as a yellow oil. The NMR-spectroscopic data was in agreement with the literature.[18]

^{1}H-NMR (501 MHz, CD_2Cl_2): δ = 7.25 (d, J = 1.6 Hz, $1H_{min}$), 6.92 (d, J = 13.0 Hz, $1H_{maj}$), 6.91 (dd, J = 8.1, 1.7 Hz, $1H_{min}$), 6.76 (d, J = 1.7 Hz, $1H_{maj}$), 6.73 (d, J = 4.6 Hz, $1H_{min}$), 6.71 (d, J = 4.5 Hz, $1H_{maj}$), 6.66 (dd, J = 8.0, 1.7 Hz, $1H_{maj}$), 6.07 (d, J = 7.0 Hz, $1H_{min}$), 5.91 (s, $2H_{min}$), 5.91 (s, $2H_{maj}$), 5.75 (d, J = 12.9 Hz, $1H_{maj}$), 5.14 (d, J = 7.0 Hz, $1H_{min}$), 3.75 (s, $3H_{min}$), 3.64 (s, $3H_{maj}$).

^{13}C-NMR (126 MHz, CD_2Cl_2): δ = 148.42, 148.32, 147.84, 147.13, 146.15, 145.84, 131.07, 130.80, 122.08, 119.24, 108.81, 108.71, 108.28, 105.51, 105.22, 105.12, 101.44, 101.34, 60.92, 56.93.

EI-HRMS: calculated for $C_{10}H_{10}O_3$ ($[M]^+$): 178.062445, found: 178.062710.

2-(benzo[*d*][1,3]dioxol-5-yl)acetaldehyde (S9)

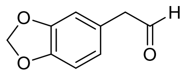

A 250 mL flask was charged with 5-(2-methoxyvinyl)benzo[*d*][1,3]dioxole (**S8**, 1.33 g, 7.47 mmol, 1.00 eq.), DCM (110 mL) and water (2.3 mL, 130 mmol, 17 eq.). The mixture was cooled to 0 °C and trifluoroacetic acid (2.3 mL, 30 mmol, 4.0 eq.) was added. The mixture was subsequently warmed to RT and stirred for 16 h. The reaction was quenched by addition of sat. aq. Na_2CO_3 and the aqueous layer was extracted with DCM (3x). The combined organic layers were dried over anhydrous Na_2SO_4 and concentrated under reduced pressure. Purification by silica gel flash column chromatography (hex/MTBE 19:1 to 9:1) yielded the product (510 mg, 2.95 mmol, 40%) as a colorless oil. The NMR-spectroscopic data was in agreement with the literature.[18]

^{1}H-NMR (501 MHz, CD_2Cl_2): δ = 9.69 (t, J = 2.2 Hz, 1H), 6.80 (d, J = 7.9 Hz, 1H), 6.69 (d, J = 1.7 Hz, 1H), 6.67 (dd, J = 7.9, 1.8 Hz, 1H), 5.96 (s, 2H), 3.59 (d, J = 2.2 Hz, 2H).

^{13}C-NMR (126 MHz, CD_2Cl_2): δ = 199.59, 148.54, 147.36, 126.10, 123.17, 110.24, 108.89, 101.74, 50.46.

EI-HRMS: calculated for $C_9H_8O_3$ ($[M]^+$): 164.046795, found: 164.046910.

1,2-dimethoxy-4-(2-methoxyvinyl)benzene (S10)

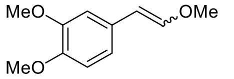

A flame-dried 500 mL three-necked flask under argon was charged with 3,4-dimethoxybenzaldehyde (5.82 g, 35 mmol, 1.0 eq.), (methoxymethyl)-triphenylphosphonium chloride (14 g, 42 mmol, 1.2 eq.) and dry THF (175 mL). The suspension was cooled to −10 °C (acetone/ice) and KO*t*-Bu (4.7 g, 42 mmol, 1.2 eq.) was added in one portion. The mixture was warmed to RT and stirred for 16 h. The reaction was quenched by addition of sat. aq. NH_4Cl and the aqueous layer was extracted with MTBE (3x). The combined organic layers were washed with brine, dried over anhydrous Na_2SO_4, and concentrated under reduced pressure. Purification by silica gel flash column chromatography (hex/EtOAc 15–20%) yielded the product (4.64 g, 23.9 mmol, 68%, $E/Z \approx 55:45$) as a colorless oil. The NMR-spectroscopic data was in agreement with the literature.[18]

^{1}H-NMR (501 MHz, $CDCl_3$): δ = 7.24 (d, J = 2.0 Hz, $1H_{min}$), 7.07 (dd, J = 8.3, 2.0 Hz, $1H_{min}$), 6.94 (d, J = 12.9 Hz, $1H_{maj}$), 6.80 (d, J = 8.4 Hz, $1H_{min}$), 6.79–6.76 (m, $3H_{maj}$), 6.07 (d, J = 7.0 Hz, $1H_{min}$), 5.78 (d, J = 12.9 Hz, $1H_{maj}$), 5.17 (d, J = 7.0 Hz, $1H_{min}$), 3.88 (s, $3H_{maj}$ + $3H_{min}$), 3.87 (s, $3H_{min}$), 3.86 (s, $3H_{maj}$), 3.77 (s, $3H_{min}$), 3.67 (s, $3H_{maj}$).

^{13}C-NMR (126 MHz, CD_2Cl_2): δ = 149.75, 149.13, 148.15, 148.00, 147.80, 147.03, 129.80, 129.62, 121.14, 117.92, 112.33, 112.30, 111.70, 108.90, 105.57, 105.17, 60.92, 56.89, 56.25, 56.13, 56.09, 56.03.

EI-HRMS: calculated for $C_{11}H_{14}O_3$ ($[M]^+$): 194.093745, found: 194.093990.

2-(3,4-dimethoxyphenyl)acetaldehyde (S11)

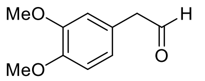

A 500 mL flask was charged with 1,2-dimethoxy-4-(2-methoxyvinyl)benzene (**S10**, 3.56 g, 18.3 mmol, 1.00 eq.), DCM (270 mL) and water (6.0 mL, 330 mmol, 18 eq.). The mixture was cooled to 0 °C and trifluoroacetic acid (6.0 mL, 78 mmol, 4.3 eq.) was added. The mixture was subsequently warmed to RT and stirred for 16 h. The reaction was quenched by addition of sat. aq. Na_2CO_3 and the aqueous layer was extracted with DCM (3x). The combined organic layers were dried over anhydrous Na_2SO_4 and concentrated under reduced pressure. Purification by silica gel flash column chromatography (hex/MTBE 10–40%) yielded the product (1.19 g, 6.27 mmol, 34%) as a colorless oil. The NMR-spectroscopic data was in agreement with the literature.[18]

^{1}H-NMR (501 MHz, CD_2Cl_2): δ = 9.73 (t, J = 2.4 Hz, 1H), 6.87 (d, J = 8.1 Hz, 1H), 6.77 (dd, J = 8.1, 2.0 Hz, 1H), 6.70 (d, J = 2.0 Hz, 1H), 3.88 (s, 6H), 3.62 (d, J = 2.5 Hz, 2H).

***tert*-butyl(4-(2-methoxyvinyl)phenoxy)dimethylsilane (S12)**

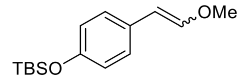

A flame-dried 500 mL three-necked flask under argon was charged with 4-((*tert*-butyldimethylsilyl)oxy)benzaldehyde (5.9 g, 25 mmol, 1.0 eq.), (methoxy-methyl)triphenylphosphonium chloride (14.6 g, 42.6 mmol, 1.70 eq.) and dry THF (175 mL). The suspension was cooled to −10 °C (acetone/ice) and KO*t*-Bu (4.78 g, 42.6 mmol, 1.70 eq.) was added in one portion. The mixture was warmed to RT and stirred for 16 h. The reaction was quenched by addition of sat. aq. NH_4Cl and the aqueous layer was extracted with MTBE (3x). The combined organic layers were washed with brine, dried over anhydrous Na_2SO_4, and concentrated under reduced

pressure. Purification by silica gel flash column chromatography (hex/EtOAc 2.5%) and another silica gel flash column chromatography (hex/MTBE 2.5%) yielded the product (4.64 g, 17.5 mmol, 70%, $E/Z \approx 1{:}1$) as a colorless oil. The NMR-spectroscopic data was in agreement with the literature.[19]

^{1}H-NMR (501 MHz, $CDCl_3$): δ = 7.47–7.42 (m, 2H), 7.11–7.07 (m, 2H), 6.93 (d, J = 13.0 Hz, 1H), 6.78–6.73 (m, 4H), 6.05 (d, J = 7.0 Hz, 1H), 5.77 (d, J = 13.0 Hz, 1H), 5.17 (d, J = 7.0 Hz, 1H), 3.75 (s, 3H), 3.66 (s, 3H), 0.98 (d, J = 0.9 Hz, 18H), 0.18 (d, J = 1.2 Hz, 12H).

EI-HRMS: calculated for $C_{15}H_{24}O_2Si_1$ ($[M]^+$): 264.154008, found: 264.154080.

2-(4-((*tert*-butyldimethylsilyl)oxy)phenyl)acetaldehyde (S13)

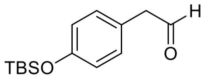

A 500 mL flask was charged with *tert*-butyl(4-(2-methoxyvinyl)phenoxy)-dimethylsilane (**S12**, 3.42 g, 13.0 mmol, 1.00 eq.), DCM (190 mL) and water (4.0 mL, 220 mmol, 17 eq.). The mixture was cooled to 0 °C and trifluoroacetic acid (4.0 mL, 52 mmol, 4.0 eq.) was added. The mixture was subsequently warmed to RT and stirred for 16 h. The reaction was quenched by addition of sat. aq. Na_2CO_3 and the aqueous layer was extracted with DCM (3x). The combined organic layers were dried over anhydrous Na_2SO_4 and concentrated under reduced pressure. Purification by silica gel flash column chromatography (hex/MTBE 2.5%) yielded the product (245 mg, 0.931 mmol, 7%) as a colorless oil. The NMR-spectroscopic data was in agreement with the literature.[19]

^{1}H-NMR (501 MHz, $CDCl_3$): δ = 9.72 (t, J = 2.5 Hz, 1H), 7.09–7.05 (m, 2H), 6.85–6.81 (m, 2H), 3.61 (d, J = 2.5 Hz, 2H), 0.98 (s, 9H), 0.20 (s, 6H).

EI-HRMS: calculated for $C_{14}H_{22}O_2Si_1$ ($[M]^+$): 250.138359, found: 250.138040.

***tert*-butyl(2-methoxy-5-(2-methoxyvinyl)phenoxy)dimethylsilane (S14)**

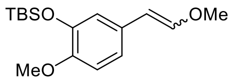

A flame-dried 250 mL three-necked flask under argon was charged with 3-((*tert*-butyldimethylsilyl)oxy)-4-methoxybenzaldehyde (7.2 g, 27 mmol, 1.0 eq.), (methoxy-methyl)triphenylphosphonium chloride (11.1 g, 32.4 mmol, 1.20 eq.) and dry THF (135 mL). The suspension was cooled to 0 °C and KO*t*-Bu (3.65 g, 32.5 mmol, 1.20 eq.) was added in one portion. The mixture was warmed to RT and stirred for 4 h. The reaction was quenched by addition of sat. aq. NH_4Cl and the aqueous layer was extracted with MTBE (3x). The combined organic layers were washed with brine, dried over anhydrous Na_2SO_4, and concentrated under reduced pressure. Purification by silica gel flash column chromatography (hex/EtOAc 19:1 to 9:1) yielded the product (6.05 g, 20.6 mmol, 76%, $E/Z \approx 55{:}45$) as a colorless oil. The NMR-spectroscopic data was in agreement with the literature.[19]

^{1}H-NMR (501 MHz, CD_2Cl_2): δ = 7.17 (d, J = 2.1 Hz, 1H), 7.06 (dd, J = 8.4, 2.1 Hz, 1H), 6.91 (d, J = 12.9 Hz, 1H), 6.79–6.73 (m, 4H), 6.05 (d, J = 7.0 Hz, 1H), 5.71 (d, J = 12.9 Hz, 1H), 5.10 (d, J = 7.0 Hz, 1H), 3.77 (s, 3H), 3.76 (s, 3H), 3.74 (s, 3H), 3.64 (s, 3H), 0.99 (s, 18H), 0.15 (s, 6H), 0.15 (s, 6H).

2-(3-((*tert*-butyldimethylsilyl)oxy)-4-methoxyphenyl)acetaldehyde (S15)

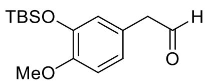

A 500 mL flask was charged with *tert*-butyl(2-methoxy-5-(2-methoxyvinyl)-phenoxy)dimethylsilane (**S14**, 6.05 g, 20.6 mmol, 1.00 eq.), DCM (360 mL) and

water (6.3 mL, 350 mmol, 17 eq.). Trifluoroacetic acid (6.3 mL, 82 mmol, 4.0 eq.) was added and the mixture was stirred at RT for 20 h. The reaction was quenched by addition of sat. aq. Na_2CO_3 and the aqueous layer was extracted with DCM (3x). The combined organic layers were dried over anhydrous Na_2SO_4 and concentrated under reduced pressure. Purification by silica gel flash column chromatography (hex/EtOAc 2–3%), automated reversed phase column chromatography (CH_3CN/H_2O 60:40 to 100:0) and another silica gel flash column chromatography (hex/MTBE 95:5) yielded the product (2.15 g, 7.68 mmol, 37%) as a colorless oil. The NMR-spectroscopic data was in agreement with the literature.[19]

^{1}H-NMR (501 MHz, $CDCl_3$): δ = 9.69 (t, J = 2.5 Hz, 1H), 6.84 (d, J = 8.2 Hz, 1H), 6.75 (dd, J = 8.1, 2.2 Hz, 1H), 6.71 (d, J = 2.2 Hz, 1H), 3.80 (s, 3H), 3.55 (d, J = 2.6 Hz, 2H), 0.99 (s, 9H), 0.15 (s, 6H).

^{13}C-NMR (126 MHz, CD_2Cl_2): δ = 199.90, 150.77, 145.69, 124.93, 123.20, 122.65, 112.80, 55.81, 50.09, 25.88, 18.74, -4.54.

ESI-HRMS: calculated for $C_{15}H_{24}O_3Si_1Na_1$ ($[M+Na]^+$): 303.138693, found: 303.138490.

1,2,3-trimethoxy-5-(2-methoxyvinyl)benzene (S16)

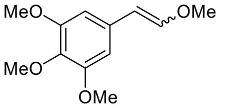

A flame-dried 250 mL three-necked flask under argon was charged with 3,4,5-trimethoxybenzaldehyde (5.9 g, 30 mmol, 1.0 eq.), (methoxymethyl)-triphenylphosphonium chloride (12.0 g, 35.0 mmol, 1.20 eq.) and dry THF (150 mL). The suspension was cooled to 0 °C and KO*t*-Bu (3.93 g, 35.0 mmol, 1.20 eq.) was added in one portion. The mixture was warmed to RT and stirred for 4 h. The reaction was quenched by addition of sat. aq. NH_4Cl and the aqueous layer was extracted with MTBE (3x). The combined organic layers were washed with brine, dried over anhydrous Na_2SO_4, and concentrated under reduced pressure. Purification by silica gel flash column chromatography (hex/EtOAc 17.5–20%) yielded the product (3.66 g, 16.3 mmol, 54%, $E/Z \approx 55{:}45$) as a colorless oil. The NMR-spectroscopic data was in agreement with the literature.[19]

^{1}H-NMR (501 MHz, CD_2Cl_2): δ = 7.00 (d, J = 12.9 Hz, $1H_{maj}$), 6.83 (s, $2H_{min}$), 6.45 (s, $2H_{maj}$), 6.12 (d, J = 7.0 Hz, $1H_{min}$), 5.75 (d, J = 12.9 Hz, $1H_{maj}$), 5.13 (d, J = 7.0 Hz, $1H_{min}$), 3.82 (s, $6H_{maj}$), 3.81 (s, $6H_{min}$), 3.78 (s, $3H_{min}$), 3.74 (s, $3H_{min}$), 3.73 (s, $3H_{maj}$), 3.67 (s, $3H_{maj}$).

EI-HRMS: calculated for $C_{12}H_{16}O_4$ ($[M]^+$): 224.104310, found: 224.104390.

2-(3,4,5-trimethoxyphenyl)acetaldehyde (S17)

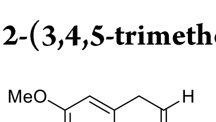

A 500 mL flask was charged with 1,2,3-trimethoxy-5-(2-methoxyvinyl)benzene (**S16**, 3.63 g, 16.2 mmol, 1.00 eq.), DCM (250 mL) and water (5.0 mL, 280 mmol, 17 eq.). Trifluoroacetic acid (5.0 mL, 65 mmol, 4.0 eq.) was added and the mixture was stirred at RT for 20 h. The reaction was quenched by addition of sat. aq. Na_2CO_3 and the aqueous layer was extracted with DCM (3x). The combined organic layers were dried over anhydrous Na_2SO_4 and concentrated under reduced pressure. Purification by silica gel flash column chromatography (hex/EtOAc 4:1 to 2:1), another silica gel flash column chromatography (DCM/acetone 2–3%), and automated reversed phase column chromatography (CH_3CN/H_2O 50:50 to 100:0) yielded the product (1.93 g, 9.17 mmol, 57%) as a yellow oil. The NMR-spectroscopic data was in agreement with the literature.[19]

^{1}**H-NMR** (501 MHz, CD_2Cl_2): δ = 9.71 (t, J = 2.3 Hz, 1H), 6.42 (s, 2H), 3.82 (s, 6H), 3.76 (s, 3H), 3.60 (d, J = 2.3 Hz, 2H).

^{13}**C-NMR** (126 MHz, CD_2Cl_2): δ = 199.50, 154.09, 137.74, 128.07, 107.07, 60.81, 56.44, 51.12.

EI-HRMS: calculated for $C_{11}H_{14}O_4$ ($[M]^+$): 210.088660, found: 210.088800.

1,2,3-trimethoxy-4-(2-methoxyvinyl)benzene (S18)

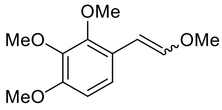

A flame-dried 250 mL three-necked flask under argon was charged with 2,3,4-trimethoxybenzaldehyde (3.0 g, 15 mmol, 1.0 eq.), (methoxymethyl)-triphenylphosphonium chloride (6.1 g, 18 mmol, 1.2 eq.) and dry THF (75 mL). The suspension was cooled to 0 °C and KO*t*-Bu (2.0 g, 18 mmol, 1.2 eq.) was added in one portion. The mixture was warmed to RT and stirred for 16 h. The reaction was quenched by addition of sat. aq. NH_4Cl and the aqueous layer was extracted with MTBE (3x). The combined organic layers were washed with brine, dried over anhydrous Na_2SO_4, and concentrated under reduced pressure. Purification by silica gel flash column chromatography (hex/EtOAc 15–20%) yielded the product (2.23 g, 9.94 mmol, 65%, $E/Z \approx 55:45$) as a colorless oil.

^{1}**H-NMR** (501 MHz, CD_2Cl_2): δ = 7.69 (d, J = 8.8 Hz, $1H_{min}$), 7.02 (d, J = 13.0 Hz, $1H_{maj}$), 6.94 (d, J = 8.7 Hz, $1H_{maj}$), 6.64 (d, J = 8.8 Hz, $1H_{min}$), 6.62 (d, J = 8.8 Hz, $1H_{maj}$), 6.13 (d, J = 7.2 Hz, $1H_{min}$), 5.91 (d, J = 13.0 Hz, $1H_{maj}$), 5.46 (d, J = 7.2 Hz, $1H_{min}$), 3.82 (s, $3H_{maj}$), 3.82 (s, $3H_{maj}$), 3.81 (s, $3H_{maj}$ + $3H_{min}$), 3.81 (s, $3H_{min}$), 3.80 (s, $3H_{min}$), 3.74 (s, $3H_{min}$), 3.67 (s, $3H_{maj}$).

^{13}**C-NMR** (126 MHz, CD_2Cl_2): δ = 152.31, 152.24, 150.90, 149.03, 147.60, 143.16, 142.64, 124.48, 123.61, 123.16, 120.07, 108.40, 107.85, 100.33, 98.80, 61.35, 61.01, 60.99, 60.92, 60.83, 56.82, 56.38, 56.30.

EI-HRMS: calculated for $C_{12}H_{16}O_4$ ($[M]^+$): 224.104310, found: 224.104590.

2-(2,3,4-trimethoxyphenyl)acetaldehyde (S19)

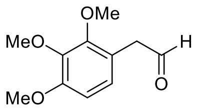

A 500 mL flask was charged with 1,2,3-trimethoxy-4-(2-methoxyvinyl)benzene (**S18**, 2.19 g, 9.78 mmol, 1.00 eq.), DCM (150 mL) and water (3.0 mL, 170 mmol, 17 eq.). Trifluoroacetic acid (3.0 mL, 39 mmol, 4.0 eq.) was added and the mixture was stirred at RT for 20 h. The reaction was quenched by addition of sat. aq. Na_2CO_3 and the aqueous layer was extracted with DCM (3x). The combined organic layers were dried over anhydrous Na_2SO_4 and concentrated under reduced pressure. Purification by silica gel flash column chromatography (hex/EtOAc 9:1 to 4:1) yielded the product (1.42 g, 6.73 mmol, 69%) as a colorless oil.

^{1}**H-NMR** (501 MHz, CD_2Cl_2): δ = 9.67 (t, J = 2.0 Hz, 1H), 6.82 (d, J = 8.4 Hz, 1H), 6.66 (d, J = 8.5 Hz, 1H), 3.84 (s, 6H), 3.83 (s, 3H), 3.58 (d, J = 1.9 Hz, 2H).

^{13}**C-NMR** (126 MHz, CD_2Cl_2): δ = 200.09, 154.00, 152.56, 142.77, 125.47, 119.12, 107.86, 61.11, 60.92, 56.35, 45.45.

EI-HRMS: calculated for $C_{11}H_{14}O_4$ ($[M]^+$): 210.088660, found: 210.088780.

1-bromo-4,5-dimethoxy-2-(2-methoxyvinyl)benzene (S20)

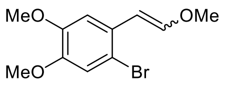

A flame-dried 250 mL three-necked flask under argon was charged with 6-bromoveratraldehyde (2.46 g, 10.0 mmol, 1.00 eq.), (methoxymethyl)triphenylphosphonium chloride (4.1 g, 12 mmol, 1.2 eq.) and dry THF (50 mL). The suspension was cooled to −10 °C (acetone/ice) and KO*t*-Bu (1.35 g, 12.0 mmol, 1.20 eq.) was added in one portion. The mixture was warmed to RT and stirred for 6.5 h. The reaction was quenched by addition of sat. aq. NH_4Cl and the aqueous layer was extracted with MTBE (3x). The combined organic layers were washed with brine, dried over anhydrous Na_2SO_4, and concentrated under reduced pressure. Purification by silica gel flash column chromatography (*n*-pentane/MTBE 9:1 to 4:1) yielded the product (1.94 g, 7.09 mmol, 71%, $E/Z \approx 65:35$) as a colorless oil.

^{1}H-NMR (501 MHz, CD_2Cl_2): δ = 7.68 (s, $1H_{min}$), 7.01 (s, $1H_{maj}$), 7.00 (s, $1H_{min}$), 6.92 (d, J = 12.8 Hz, $1H_{maj}$), 6.84 (s, $1H_{maj}$), 6.20 (d, J = 7.2 Hz, $1H_{min}$), 6.00 (d, J = 12.9 Hz, $1H_{maj}$), 5.48 (d, J = 7.2 Hz, $1H_{min}$), 3.82 (s, $3H_{maj}$), 3.81 (s, $3H_{min}$), 3.80–3.79 (m, $3H_{maj}$ + $3H_{min}$), 3.78 (s, $3H_{min}$), 3.70 (s, $3H_{maj}$).

^{13}C-NMR (126 MHz, CD_2Cl_2): δ = 149.96, 149.25, 148.68, 148.55, 148.43, 148.22, 128.79, 128.11, 116.20, 115.75, 113.42, 113.12, 113.04, 109.00, 104.66, 103.80, 61.18, 57.05, 56.49, 56.37, 56.34, 56.18.

EI-HRMS: calculated for $C_{11}H_{13}O_3Br_1$ ($[M]^+$): 272.004270, found: 272.004750.

2-(2-bromo-4,5-dimethoxyphenyl)acetaldehyde (S21)

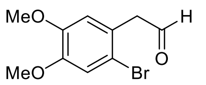

A 250 mL flask was charged with 1-bromo-4,5-dimethoxy-2-(2-methoxyvinyl)benzene (**S20**, 1.94 g, 7.09 mmol, 1.00 eq.), DCM (100 mL) and water (2.2 mL, 120 mmol, 17 eq.). Trifluoroacetic acid (2.2 mL, 29 mmol, 4.1 eq.) was added and the mixture was stirred at RT for 20 h. The reaction was quenched by addition of sat. aq. Na_2CO_3 and the aqueous layer was extracted with DCM (3x). The combined organic layers were dried over anhydrous Na_2SO_4 and concentrated under reduced pressure. Purification by silica gel flash column chromatography (hex/MTBE 4:1 to 2:1) yielded the product (1.36 g, 5.27 mmol, 74%) as a white solid.

^{1}H-NMR (501 MHz, CD_2Cl_2): δ = 9.71 (t, J = 1.7 Hz, 1H), 7.08 (s, 1H), 6.72 (s, 1H), 3.83 (s, 3H), 3.81 (s, 3H), 3.77 (d, J = 1.7 Hz, 2H).

^{13}C-NMR (126 MHz, CD_2Cl_2): δ = 198.78, 149.72, 149.32, 124.90, 116.19, 115.20, 114.79, 56.54, 56.43, 50.56.

EI-HRMS: calculated for $C_{10}H_{11}O_3Br_1$ ($[M]^+$): 257.988620, found: 257.988810.

3-(3,4-dimethoxyphenyl)propanal (S22)

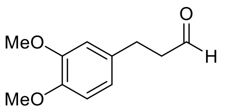

The reaction was performed according to a literature procedure.[20] A flame-dried Schlenk under argon was charged with $NaHCO_3$ (1.68 g, 20.0 mmol, 2.00 eq.), tetrabutylammonium bromide (1.61 g, 5.00 mmol, 0.5 eq.), $Pd(OAc)_2$ (112 mg, 0.500 mmol, 0.05 eq.), and dry DMF (8.5 mL) and the mixture was sparged with argon for 30 seconds. Allyl alcohol (1.0 mL, 15 mmol, 1.5 eq.) and 4-iodo-1,2-dimethoxybenzene (2.64 g, 10.0 mmol, 1.00 eq.) were added, the flask was closed, and the mixture was heated to 80 °C for 4.5 h. After cooling

to RT, the reaction was diluted with MTBE and subsequently washed with H_2O (3x) and brine. The organic layer was dried over anhydrous Na_2SO_4 and concentrated under reduced pressure. Purification by silica gel flash column chromatography (hex/MTBE 9:1 to 4:1) yielded the product (1.20 g, 6.18 mmol, 62%) as a colorless oil. The NMR-spectroscopic data was in agreement with the literature.[21]

^{1}H-NMR (501 MHz, $CDCl_3$): δ = 9.82 (t, J = 1.6 Hz, 1H), 6.79 (d, J = 7.9 Hz, 1H), 6.74–6.70 (m, 2H), 3.87 (s, 3H), 3.85 (s, 3H), 2.91 (t, J = 7.5 Hz, 2H), 2.78–2.74 (m, 2H).

^{13}C-NMR (126 MHz, $CDCl_3$): δ = 201.83, 149.13, 147.69, 133.06, 120.23, 111.83, 111.53, 56.07, 55.98, 45.64, 27.89.

3-(benzo[*d*][1,3]dioxol-5-yl)propanal (S23)

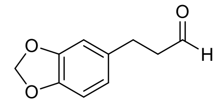

The reaction was performed according to a literature procedure.[20] A flame-dried Schlenk under argon was charged with $NaHCO_3$ (1.68 g, 20.0 mmol, 2.00 eq.), tetrabutylammonium bromide (1.61 g, 5.00 mmol, 0.5 eq.), $Pd(OAc)_2$ (112 mg, 0.500 mmol, 0.05 eq.), and dry DMF (8.5 mL) and the mixture was sparged with argon for 30 seconds. Allyl alcohol (1.0 mL, 15 mmol, 1.5 eq.) and 5-iodobenzo[*d*][1,3]dioxole (2.48 g, 10.0 mmol, 1.00 eq.) were added, the flask was closed, and the mixture was heated to 80 °C for 4.5 h. After cooling to RT, the reaction was diluted with MTBE and subsequently washed with H_2O (3x) and brine. The organic layer was dried over anhydrous Na_2SO_4 and concentrated under reduced pressure. Purification by silica gel flash column chromatography (*n*-pentane/MTBE 19:1 to 9:1) yielded the product (1.34 g, 7.49 mmol, 75%) as a colorless oil. The NMR-spectroscopic data was in agreement with the literature.[20]

^{1}H-NMR (501 MHz, $CDCl_3$): δ = 9.80 (t, J = 1.4 Hz, 1H), 6.73 (d, J = 7.9 Hz, 1H), 6.68 (d, J = 1.7 Hz, 1H), 6.63 (dd, J = 7.9, 1.7 Hz, 1H), 5.92 (s, 2H), 2.88 (t, J = 7.5 Hz, 2H), 2.73 (ddd, J = 8.9, 7.4, 1.3 Hz, 2H).

3-(3,4,5-trimethoxyphenyl)propanal (S24)

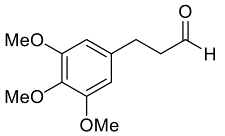

The reaction was performed according to a literature procedure.[20] A flame-dried Schlenk under argon was charged with $NaHCO_3$ (1.68 g, 20.0 mmol, 2.00 eq.), tetrabutylammonium bromide (1.61 g, 5.00 mmol, 0.5 eq.), $Pd(OAc)_2$ (112 mg, 0.500 mmol, 0.05 eq.), and dry DMF (8.5 mL) and the mixture was sparged with argon for 30 seconds. Allyl alcohol (1.0 mL, 15 mmol, 1.5 eq.) and 5-iodo-1,2,3-trimethoxybenzene (2.94 g, 10.0 mmol, 1.00 eq.) were added, the flask was closed, and the mixture was heated to 80 °C for 5 h. After cooling to RT, the reaction was diluted with MTBE and subsequently washed with H_2O (3x) and brine. The organic layer was dried over anhydrous Na_2SO_4 and concentrated under reduced pressure. Purification by silica gel flash column chromatography (hex/MTBE 10–40%) yielded the product (1.28 g, 5.69 mmol, 57%) as a colorless oil. The NMR-spectroscopic data was in agreement with the literature.[22]

^{1}H-NMR (501 MHz, $CDCl_3$): δ = 9.82 (p, J = 1.2 Hz, 1H), 6.40 (s, 2H), 3.84 (q, J = 1.0 Hz, 6H), 3.81 (q, J = 0.9 Hz, 3H), 2.90 (t, J = 7.5 Hz, 2H), 2.79–2.72 (m, 2H).

^{13}C-NMR (126 MHz, $CDCl_3$): δ = 201.56, 153.40, 136.61, 136.25, 105.40, 60.96, 56.21, 45.51, 28.60.

2-(*tert*-butoxy)acetaldehyde (S25)

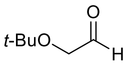

A flame-dried 250 mL two-necked flask under argon was charged with 2-*tert*-butoxyethanol (2.0 g, 17 mmol, 1.0 eq.) and dry DCM (80 mL) and cooled to 0 °C. Dess-Martin periodinane (7.96 g, 18.8 mmol, 1.11 eq.) was added, and the mixture was stirred at RT for 2.5 h. The reaction was quenched by addition of sat. aq. $NaHCO_3$, the organic layer was extracted with DCM (3x), and the combined organic layers were dried over dried over anhydrous Na_2SO_4 and concentrated under reduced pressure. Purification by silica gel flash column chromatography (*n*-pentane/Et_2O 3:1) yielded the product (1.17 g, 5.84 mmol, 35%) as a colorless 58 wt% solution in DCM.

^{1}H-NMR (501 MHz, $CDCl_3$): δ = 9.72 (t, J = 1.2 Hz, 1H), 3.98 (d, J = 1.1 Hz, 2H), 1.24 (s, 9H).

^{13}C-NMR (126 MHz, $CDCl_3$): δ = 202.56, 74.51, 68.77, 27.51.

EI-HRMS: calculated for $C_6H_{13}O_2$ ($[M+H]^+$): 117.091005, found: 117.091200.

7. Synthesis of IDPi Catalysts

2-bromo-6-(*tert*-butyl)benzofuran (S29)

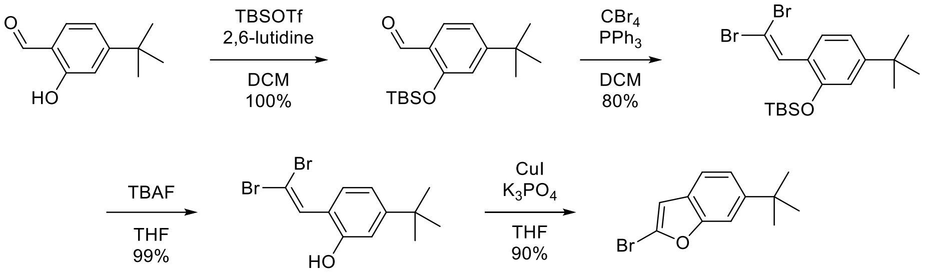

4-(*tert*-butyl)-2-((*tert*-butyldimethylsilyl)oxy)benzaldehyde (S26)

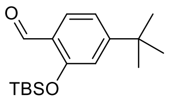

A flame-dried 100 mL Schlenk flask under argon was charged with 4-*tert*-butylsalicylaldehyde (5.24 g, 29.4 mmol, 1.00 eq.), DCM (60 mL), and 2,6-lutidine (10.5 mL, 89.2 mmol, 3.04 eq.). The mixture was cooled to 0 °C and TBSOTf (10.0 mL, 43.5 mmol, 1.48 eq.) was added slowly. The mixture was warmed to RT and stirred for 24 h. The reaction was quenched by addition of sat. aq. $NaHCO_3$ and extracted with DCM (3x). The combined organic layers were washed with brine, dried over anhydrous Na_2SO_4, and concentrated under reduced pressure. Purification by silica gel flash column chromatography (hex/EtOAc 100:0 to 99:1) yielded the product as a yellow oil (8.60 g, 29.4 mmol, 100%).

$\boldsymbol{R}_F$ (hex/EtOAc 9:1) = 0.57.

^{1}H-NMR (501 MHz, $CDCl_3$): δ = 10.40 (d, J = 0.9 Hz, 1H), 7.74 (d, J = 8.2 Hz, 1H), 7.07 (ddd, J = 8.3, 1.8, 0.9 Hz, 1H), 6.88 (d, J = 1.7 Hz, 1H), 1.31 (s, 10H), 1.03 (s, 10H), 0.28 (s, 6H).

^{13}C-NMR (126 MHz, $CDCl_3$): δ = 189.93, 160.29, 158.97, 128.09, 124.93, 119.07, 117.46, 35.38, 31.06, 25.86, 18.53, -4.09.

CI-HRMS: calculated for $C_{17}H_{29}O_2Si_1$ ($[M+H]^+$): 293.193134, found: 293.193110.

tert-butyl(5-(*tert*-butyl)-2-(2,2-dibromovinyl)phenoxy)dimethylsilane (S27)

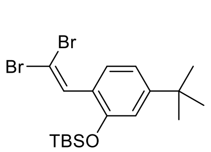

A flame-dried 500 mL three-necked flask under argon was charged with 4-(*tert*-butyl)-2-((*tert*-butyldimethylsilyl)oxy)benzaldehyde (**S26**, 8.60 g, 29.4 mmol, 1.00 eq.) and DCM (140 mL) and cooled to 0 °C. Triphenylphosphine (23.1 g, 88.1 mmol, 3.00 eq.) and tetrabromomethane (14.6 g, 44.0 mmol, 1.50 eq.) were added simultaneously and the mixture was allowed to slowly warm to RT overnight. The reaction was quenched by addition of Na_2SO_3 (75 g in 500 mL H_2O) and the aqueous layer was extracted with DCM (3x). The combined organic phases were washed with sat. aq. $NaHCO_3$, H_2O, and brine, dried over anhydrous Na_2SO_4, and concentrated under reduced pressure. Purification by silica gel flash column chromatography (hex/EtOAc 100:0 to 98:2) yielded the product as a yellow oil (10.5 g, 23.4 mmol, 80%).

$\boldsymbol{R}_F$ (hex/EtOAc 19:1) = 0.76.

^{1}H-NMR (501 MHz, $CDCl_3$): δ = 7.63 (d, J = 8.2 Hz, 1H), 7.56 (s, 1H), 7.00 (dd, J = 8.2, 1.9 Hz, 1H), 6.83 (d, J = 1.9 Hz, 1H), 1.29 (s, 9H), 1.03 (s, 8H), 0.21 (s, 5H).

^{13}**C-NMR** (126 MHz, $CDCl_3$): δ = 153.49, 152.79, 133.69, 128.42, 124.17, 118.07, 116.78, 88.66, 34.68, 31.12, 25.75, 18.26, -4.32.

EI-HRMS: calculated for $C_{18}H_{28}O_1Si_1Br_2$ ($[M]^+$): 446.027094, found: 446.026650.

5-(*tert*-butyl)-2-(2,2-dibromovinyl)phenol (S28)

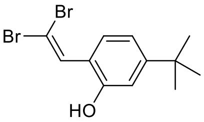

A flame-dried 250 mL three-necked flask under argon was charged with *tert*-butyl(5-(*tert*-butyl)-2-(2,2-dibromovinyl)phenoxy)dimethylsilane (**S27**, 10.5 g, 23.4 mmol, 1.00 eq.) and THF (120 mL) and cooled to 0 °C. TBAF (1.0 M in THF, 30 mL, 30 mmol, 1.28 eq.) was added and the mixture was allowed to slowly warm to RT overnight. The reaction was quenched by addition of H_2O (100 mL) and the aqueous layer was extracted with MTBE (3x). The combined organic phases were washed with sat. aq. NH_4Cl and brine, dried over anhydrous Na_2SO_4, and concentrated under reduced pressure. Purification by silica gel flash column chromatography (hex/EtOAc 100:0 to 95:5) yielded the product as a colorless oil (7.73 g, 23.1 mmol, 99%).

R_F (hex/EtOAc 9:1) = 0.33.

^{1}**H-NMR** (501 MHz, $CDCl_3$): δ = 7.54 (s, 1H), 7.51 (dd, J = 8.2, 0.7 Hz, 1H), 6.98 (dd, J = 8.2, 1.9 Hz, 1H), 6.84 (d, J = 1.9 Hz, 1H), 4.93 (s, 1H), 1.30 (s, 9H).

^{13}**C-NMR** (126 MHz, $CDCl_3$): δ = 154.14, 152.38, 132.35, 128.75, 120.01, 118.00, 113.06, 91.18, 34.90, 31.24.

EI-HRMS: calculated for $C_{12}H_{14}O_1Br_2$ ($[M]^+$): 331.940616, found: 331.940700.

2-bromo-6-(*tert*-butyl)benzofuran (S29)

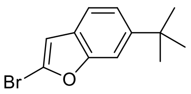

The reaction was conducted according to a literature procedure for similar substrates.[23] A flame-dried 250 mL Young Schlenk under argon was charged with K_3PO_4 (9.6 g, 45 mmol, 2.0 eq.) and CuI (216 mg, 1.13 mmol, 5 mol%). 5-(*tert*-butyl)-2-(2,2-dibromovinyl)phenol (**S28**, 7.57 g, 22.7 mmol, 1.00 eq.) was added in THF (110 mL), the flask was closed and covered in aluminum foil, and the mixture was heated to 80 °C for 20 h. After cooling to RT, the mixture was filtered over Celite® (eluted with MTBE), and concentrated under reduced pressure. Purification by silica gel flash column chromatography (100% hex) yielded the product as a colorless oil (5.15 g, 20.4 mmol, 90%).

R_F (100% hex) = 0.68.

^{1}**H-NMR** (501 MHz, $CDCl_3$): δ = 7.47 (dt, J = 1.6, 0.8 Hz, 1H), 7.42 (dd, J = 8.2, 0.6 Hz, 1H), 7.30 (dd, J = 8.2, 1.6 Hz, 1H), 6.67 (d, J = 0.9 Hz, 1H), 1.36 (s, 9H).

^{13}**C-NMR** (126 MHz, $CDCl_3$): δ = 148.41, 127.63, 126.19, 121.29, 119.43, 108.06, 107.76, 35.10, 31.74.

EI-HRMS: calculated for $C_{12}H_{13}O_1Br_1$ ($[M]^+$): 252.014440, found: 252.014680.

2-iodo-6-(2-methyladamantan-2-yl)benzofuran (S31)

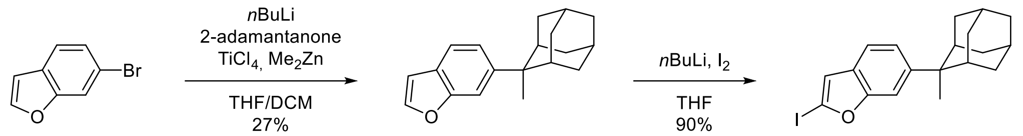

6-(2-methyladamantan-2-yl)benzofuran (S30)

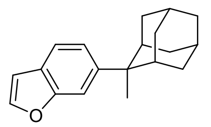

A flame-dried 100 mL Schlenk flask under argon was charged with 6-bromobenzofuran (2.48 g, 12.6 mmol, 1.00 eq.) and THF (50 mL). The mixture was cooled to –78 °C, *n*BuLi (2.40 M in *n*-hexane, 5.50 mL, 13.2 mmol, 1.05 eq.) was added slowly, and the reaction was stirred for 2 h at –78 °C (formation of a white precipitate was observed). 2-adamantanone (2.08 g, 13.9 mmol, 1.10 eq.) was added in one portion, and the reaction was allowed to slowly warm to RT over 16 h. The solvent was removed under reduced pressure and a white powder was obtained that was further dried on high vacuum for 30 min. The solid was redissolved in DCM (60 mL) and cooled to –40 °C. $TiCl_4$ (5.5 mL, 50 mmol, 4.0 eq.) and Me_2Zn (5.92 M in DCM, 8.5 mL, 50 mmol, 4.0 eq.) were added simultaneously and the reaction was stirred at –40 °C for 1h, and at –10 °C for another 2 h. The mixture was poured onto ice water and the aqueous layer was extracted with DCM (3x). The combined organic layers were dried over anhydrous Na_2SO_4 and concentrated under reduced pressure. The crude mixture was purified by silica gel flash column chromatography (100% pentane) to give the product as a colorless oil (907 mg, 3.40 mmol, 27%).

$\boldsymbol{R_F}$ (pentane) = 0.51.

^{1}H-NMR (501 MHz, CD_2Cl_2): δ = 7.60 (d, J = 2.2 Hz, 1H), 7.54 (d, J = 8.3 Hz, 1H), 7.51–7.50 (m, 1H), 7.28 (dd, J = 8.3, 1.7 Hz, 1H), 6.74 (dd, J = 2.3, 1.0 Hz, 1H), 2.40 (t, J = 3.2 Hz, 2H), 2.29 (dd, J = 12.8, 3.2 Hz, 3H), 1.97–1.87 (m, 4H), 1.80 (dtt, J = 12.0, 2.7, 1.6 Hz, 2H), 1.76–1.66 (m, 4H), 1.63–1.55 (m, 2H), 1.26 (s, 3H).

^{13}C-NMR (126 MHz, CD_2Cl_2): δ = 156.26, 148.47, 145.05, 124.72, 121.00, 120.89, 108.86, 106.59, 44.09, 39.18, 35.18, 34.40, 33.52, 30.99, 28.53, 28.20.

EI-HRMS: calculated for $C_{19}H_{22}O_1$ ([M]$^+$): 266.166515, found: 266.166970.

2-iodo-6-(2-methyladamantan-2-yl)benzofuran (S31)

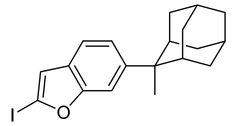

A flame-dried 50 mL Schlenk flask under argon was charged with 6-(2-methyladamantan-2-yl)benzofuran (**S30**, 868 mg, 3.26 mmol, 1.00 eq.) and THF (10 mL). The mixture was cooled to –78 °C and *n*BuLi (2.50 M in *n*-hexane, 1.70 mL, 4.25 mmol, 1.30 eq.) was added slowly. The reaction was warmed to 0 °C, stirred for 2 h, and cooled to –78 °C again. Iodine (1.34 g, 5.28 mmol, 1.62 eq.) was dissolved in THF (2 mL) and slowly added to the reaction mixture, which was allowed to slowly warm to RT overnight. The reaction was quenched by addition of H_2O (100 mL) and the aqueous layer was extracted with MTBE (3x). The combined organic phases were washed with sat. aq. Na_2SO_3 and brine, dried over anhydrous Na_2SO_4, and concentrated under reduced pressure. The crude mixture was purified by silica gel flash column chromatography (100% pentane) to yield the product as a white foam (1.15 g, 2.93 mmol, 90%).

$\boldsymbol{R_F}$ (pentane) = 0.56.

^{1}H-NMR (501 MHz, $CDCl_3$): δ = 7.48–7.46 (m, 1H), 7.43 (d, J = 8.2 Hz, 1H), 7.23 (dd, J = 8.3, 1.6 Hz, 1H), 6.90 (d, J = 0.9 Hz, 1H), 2.36 (d, J = 4.0 Hz, 2H), 2.26 (dd, J = 13.1, 3.3 Hz, 3H), 1.93 (s, 1H), 1.87 (d, J = 12.7 Hz, 2H), 1.82–1.74 (m, 2H), 1.70 (s, 3H), 1.57 (dd, J = 12.4, 2.9 Hz, 2H), 1.25 (s, 3H).

^{13}C-NMR (126 MHz, $CDCl_3$): δ = 159.24, 148.23, 126.36, 120.89, 119.32, 117.05, 108.16, 94.85, 43.82, 38.88, 34.82, 34.08, 33.27, 30.89, 28.05, 27.71.

EI-HRMS: calculated for $C_{19}H_{21}O_1I_1$ ($[M]^+$): 392.063162, found: 392.063900.

Synthesis of substituted BINOLs

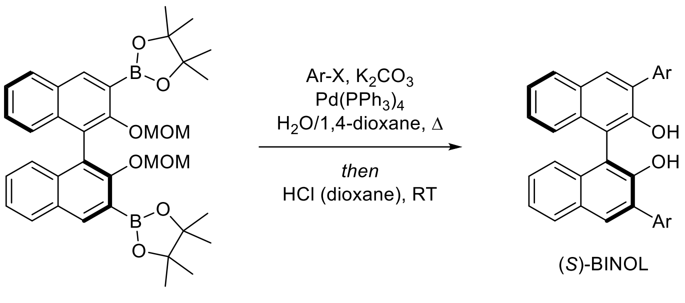

(*S*)-3,3'-bis(benzofuran-2-yl)-BINOL (S32)

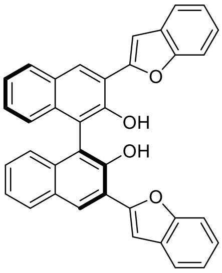

A 100 mL three-necked flask equipped with a reflux condenser was charged with (*S*)-MOM-BINOL Bpin ester (2.24 g, 3.58 mmol, 1.00 eq.) and 2-bromobenzofuran (1.90 g, 9.64 mmol, 2.70 eq.) and argonated (3x). 1,4-dioxane (30 mL) and K_2CO_3 (2.0 M in H2O, 10 mL, 20 mmol, 5.6 eq.) were added and the solution was sparged with argon for 20 min. Subsequently, $Pd(PPh_3)_4$ (185 mg, 0.160 mmol, 0.045 eq.) was added and the reaction was heated to reflux for 5 h. After cooling to RT, the reaction was quenched by addition of sat. aq. NH_4Cl (50 mL) and the aqueous layer was extracted with EtOAc (3x). The combined organic phases were washed with brine, dried over anhydrous Na_2SO_4, and concentrated under reduced pressure. The crude material was dissolved in THF (25 mL) in a 100 mL round bottom flask under air. HCl (4.0 M in 1,4-dioxane, 16 mL, 64 mmol, 18 eq.) was added and the reaction was stirred at RT for 16 h, after which full conversion was confirmed by TLC (hex/EtOAc 9:1). The mixture was diluted with EtOAc (50 mL) and quenched by addition of HCl (1.2 M, 100 mL). The aqueous layer was extracted with EtOAc (3x), the combined organic phases were washed with sat. aq. $NaHCO_3$ and brine, dried over anhydrous Na_2SO_4, and concentrated under reduced pressure. The crude material was purified by silica gel flash column chromatography (hex/EtOAc 19:1 to 9:1) to give the product as an off-white solid (1.85 g, 3.57 mmol, 99%).

R_F (hex/EtOAc 9:1) = 0.28.

^{1}H-NMR (501 MHz, CDCl3): δ = 8.78 (s, 2H), 8.05 (d, J = 8.1 Hz, 2H), 7.61 (td, J = 7.8, 1.1 Hz, 4H), 7.49 (d, J = 1.0 Hz, 2H), 7.44 (ddd, J = 8.0, 6.7, 1.2 Hz, 2H), 7.34 (dddd, J = 15.2, 8.2, 7.0, 1.3 Hz, 4H), 7.25 (td, J = 7.4, 1.0 Hz, 2H), 7.18 (dd, J = 8.5, 1.1 Hz, 2H), 5.88 (s, 2H).

^{13}**C-NMR** (126 MHz, CDCl3): δ = 154.41, 151.78, 149.98, 133.00, 129.69, 129.42, 129.19, 128.51, 128.23, 124.97, 124.22, 123.13, 121.61, 119.53, 111.95, 111.07, 107.86.

ESI-HRMS: calculated for C36H21O4 ([M-H]$^-$): 517.144535, found: 517.144550.

$[\alpha]_D^{25}$ = +91.9 (*c* = 0.21, $CHCl_3$).

(*S*)-3,3'-bis(6-(*tert*-butyl)benzofuran-2-yl)-BINOL (S33)

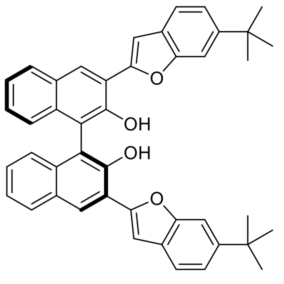

A 100 mL three-necked flask equipped with a reflux condenser under argon was charged with (*S*)-MOM-BINOL Bpin ester (1.70 g, 2.70 mmol, 1.00 eq.), 2-bromo-6-(*tert*-butyl)benzofuran (**S29**, 1.68 g, 2.50 mmol, 2.50 eq.), 1,4-dioxane (25 mL), and K_2CO_3 (2.0 M in H_2O, 8.5 mL, 17 mmol, 6.3 eq.), and the solution as sparged with argon for 15 min. $Pd(PPh_3)_4$ (157 mg, 0.14 mmol, 5 mol%) was added lastly and the mixture was heated to reflux for 16 h. After cooling to RT, the reaction was quenched by addition of sat. aq. NH_4Cl and the aqueous layer was extracted with EtOAc (3x). The combined organic phases were washed with brine, dried over anhydrous Na_2SO_4, and concentrated under reduced pressure. The crude material was dissolved in THF (20 mL) in a 100 mL round bottom flask under air. HCl (4.0 M in 1,4-dioxane, 14 mL, 56 mmol, 21 eq.) was added and the reaction was stirred at RT for 16 h. The mixture was diluted with EtOAc and quenched by addition of HCl (1.2 M). The aqueous layer was extracted with EtOAc (3x), the combined organic phases were washed sat. aq. $NaHCO_3$ and brine, dried over anhydrous Na_2SO_4, and concentrated under reduced pressure. The crude material was purified by silica gel flash column chromatography (hex/EtOAc 19:1 to 9:1) and another silica gel flash column chromatography (hex/DCM 20-30%) to yield the product as a white solid (1.59 g, 2.53 mmol, 79%).

R_F (hex/EtOAc 9:1) = 0.50.

^{1}**H-NMR** (501 MHz, $CDCl_3$): δ = 8.75 (s, 2H), 8.03 (d, J = 8.1 Hz, 2H), 7.65 (d, J = 1.6 Hz, 2H), 7.53 (d, J = 8.2 Hz, 2H), 7.47 (d, J = 1.0 Hz, 2H), 7.43 (ddd, J = 8.1, 6.8, 1.2 Hz, 2H), 7.36–7.29 (m, 4H), 7.17 (dd, J = 8.4, 1.1 Hz, 2H), 5.89 (s, 2H), 1.43 (s, 18H).

^{13}**C-NMR** (126 MHz, $CDCl_3$): δ = 154.85, 151.60, 149.96, 149.10, 132.92, 129.47, 129.11, 128.17, 128.08, 127.05, 124.92, 124.28, 121.03, 120.86, 119.78, 111.98, 107.78, 107.65, 35.25, 31.85.

ESI-HRMS: calculated for $C_{44}H_{37}O_4$ ([M-H]$^-$): 629.269735, found: 629.270020.

$[\alpha]_D^{25}$ = +90.1 (*c* = 0.30, $CHCl_3$).

(S)-3,3'-bis(6-(2-methyladamantan-2-yl)benzofuran-2-yl)-BINOL (S34)

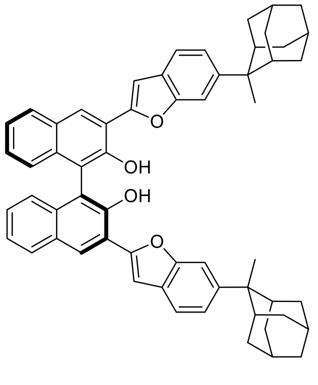

A 50 mL three-necked flask equipped with a reflux condenser under argon was charged with (S)-MOM-BINOL Bpin ester (1.27 g, 2.02 mmol, 1.00 eq.), 2-iodo-6-(2-methyladamantan-2-yl)benzofuran (**S31**, 1.13 g, 4.56 mmol, 2.26 eq.), 1,4-dioxane (20 mL), and K_2CO_3 (2.0 M in H_2O, 6.0 mL, 12 mmol, 5.9 eq.), and the solution was sparged with argon for 15 min. $Pd(PPh_3)_4$ (117 mg, 0.10 mmol, 5 mol%) was added lastly and the mixture was heated to reflux for 16 h. After cooling to RT, the reaction was quenched by addition of sat. aq. NH_4Cl and the aqueous layer was extracted with EtOAc (3x). The combined organic phases were washed with brine, dried over anhydrous Na_2SO_4, and concentrated under reduced pressure. The crude material was dissolved in THF (20 mL) in a 100 mL round bottom flask under air. HCl (4.0 M in 1,4-dioxane, 10 mL, 40 mmol, 20 eq.) was added and the reaction was stirred at RT for 16 h. The mixture was diluted with EtOAc and quenched by addition of HCl (1.2 M). The aqueous layer was extracted with EtOAc (3x), the combined organic phases were washed sat. aq. $NaHCO_3$ and brine, dried over anhydrous Na_2SO_4, and concentrated under reduced pressure. The crude material was purified by silica gel flash column chromatography (hex/DCM 15-20%) to yield the product as a white solid (790 mg, 0.969 mmol, 48%).

R_F (hex/EtOAc 19:1) = 0.27.

^{1}H-NMR (501 MHz, $CDCl_3$): δ = 8.74 (s, 2H), 8.02 (d, J = 8.2 Hz, 2H), 7.61 (s, 2H), 7.54 (d, J = 8.2 Hz, 2H), 7.47 (d, J = 0.9 Hz, 2H), 7.43 (ddd, J = 8.1, 6.7, 1.2 Hz, 2H), 7.32 (ddd, J = 8.2, 6.8, 1.3 Hz, 2H), 7.29 (dd, J = 8.3, 1.5 Hz, 2H), 7.17 (dd, J = 8.4, 1.1 Hz, 2H), 5.91 (s, 2H), 2.44 (d, J = 3.7 Hz, 4H), 2.30 (dd, J = 12.6, 3.3 Hz, 4H), 2.02–1.93 (m, 6H), 1.87–1.78 (m, 4H), 1.78–1.70 (m, 6H), 1.66–1.58 (m, 4H), 1.32 (s, 6H).

^{13}C-NMR (126 MHz, $CDCl_3$): δ = 155.34, 151.50, 149.97, 148.97, 132.90, 129.48, 129.09, 128.11, 128.04, 126.66, 124.90, 124.29, 121.13, 120.87, 119.83, 112.03, 108.24, 107.70, 44.02, 38.96, 34.93, 34.22, 33.36, 30.91, 28.12, 27.79.

ESI-HRMS: calculated for $C_{58}H_{53}O_4^-$ ([M-H]$^-$): 813.394935, found: 813.394950.

$[\alpha]_D^{25}$ = +53.1 (c = 0.38, $CHCl_3$).

Synthesis of IDPi catalysts

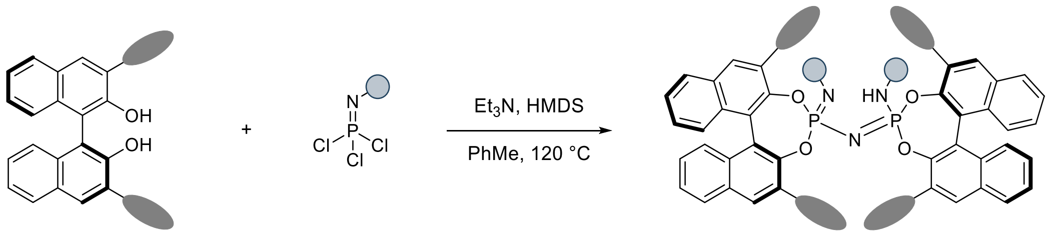

(*S*,*S*)-(benzofuran-2-yl)-CF_3-IDPi (5a)

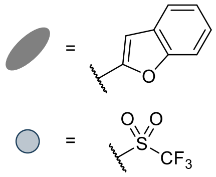

(*S*)-BINOL **S32** (75.0 mg, 0.145 mmol, 2.01 eq.) was placed in a flame-dried Young-Schlenk under Argon and dissolved in toluene (1.5 mL). P(NTf)Cl_3 (23.0 µL, 0.144 mmol, 2.00 eq.) and subsequently Et_3N (80.0 µL, 0.574 mmol, 7.99 eq.) were added to the reaction and the mixture was stirred at RT for 30 min. HMDS (15.0 µL, 0.072 mmol, 1.00 eq.) was added, the mixture was stirred at RT for 15 min, and subsequently heated to 120 °C for 3 d. After cooling to RT, the mixture was diluted with DCM and quenched by addition of HCl (1.2 M). The aqueous layer was extracted with DCM (3x), the combined organic phases were dried over anhydrous Na_2SO_4 and concentrated under reduced pressure. The crude material was purified by silica gel flash column chromatography (hex/EtOAc 2:1) and another silica gel flash column chromatography (DCM/EtOAc 19:1). The product was acidified by filtration over a short plug of DOWEX 50WX8 (H-form, eluted with DCM) and obtained as a beige solid (85.0 mg, 0.061 mmol, 84%).

$\boldsymbol{R_F}$ (hex/EtOAc 2:1) = 0.28.

^{1}H-NMR (501 MHz, CD_2Cl_2): complexed with *n*-pentane. δ = 8.64 (s, 2H), 8.12–8.06 (m, 2H), 8.06–8.00 (m, 2H), 7.88 (ddd, J = 8.0, 6.7, 1.1 Hz, 2H), 7.70–7.62 (m, 6H), 7.57 (ddd, J = 8.1, 6.7, 1.1 Hz, 2H), 7.48–7.40 (m, 4H), 7.39–7.29 (m, 4H), 7.29–7.16 (m, 6H), 7.16–7.11 (m, 2H), 6.91 (d, J = 15.1 Hz, 4H), 6.84 (ddd, J = 8.4, 7.1, 1.2 Hz, 2H), 6.80 (s, 2H), 6.63 (td, J = 7.4, 1.1 Hz, 2H).

^{19}F-NMR (471 MHz, CD_2Cl_2): δ = -78.30.

^{31}P-NMR (203 MHz, CD_2Cl_2): δ = -16.89.

^{13}C-NMR (126 MHz, CD_2Cl_2): δ = 154.79, 154.57, 149.98, 149.65, 142.71 (t, J = 5.2 Hz), 141.58(t, J = 5.3 Hz), 132.21, 132.03, 131.96, 131.93, 129.92, 129.70, 129.50, 129.08, 129.03, 128.94, 128.15, 127.88, 127.88, 127.45, 127.27, 127.25, 125.65, 125.08, 123.65, 123.30, 122.97, 122.05, 122.03, 121.94, 121.86, 121.68, 119.86 (qt, J = 320.4, 2.6 Hz), 111.35, 110.82, 108.36, 107.67.

ESI-HRMS: calculated for $C_{74}H_{40}N_3O_{12}P_2S_2F_6^-$ ($[M-H]^-$): 1402.143846, found: 1402.145400.

$[\alpha]_D^{25}$ = +573.4 (*c* = 0.22, $CHCl_3$).

(*S*,*S*)-(benzofuran-2-yl)-C_6F_5-IDPi (5b)

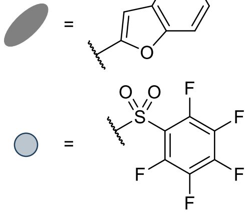

(*S*)-BINOL **S32** (1.06 g, 2.05 mmol, 2.03 eq.) was added to a flame-dried Young-Schlenk charged with $P(NSO_2C_6F_5)Cl_3$ (782 mg, 2.04 mmol, 2.03 eq.) and the solids were gently heated to 50 °C with a heat gun under high vacuum for 10 min. The mixture was placed under argon and suspended in dry toluene (20 mL). Et_3N (1.2 mL, 8.6 mmol, 8.6 eq.) was added and the mixture was stirred at RT for 1 h. HMDS (210 µL, 1.01 mmol, 1.00 eq.) was added, the mixture was stirred at RT for 10 min and subsequently heated to 120 °C for 3 d. After cooling to RT, the mixture was diluted with DCM and quenched by addition of HCl (1.2 M). The aqueous layer was extracted with DCM (3x), the combined organic phases were dried over anhydrous Na_2SO_4 and concentrated under reduced pressure. The crude material was purified by silica gel flash column chromatography (100% DCM). The product was acidified by filtration over a short plug of DOWEX 50WX8 (H-form, eluted with DCM) and obtained as an off-white solid (825 mg, 0.516 mmol, 51%).

$\boldsymbol{R}_F$ (hex/EtOAc 2:1) = 0.17.

^{1}H-NMR (501 MHz, CD_2Cl_2): δ = 8.55 (s, 2H), 8.12 (d, J = 8.1 Hz, 2H), 8.07 (d, J = 8.3 Hz, 2H), 7.87 (ddd, J = 8.1, 6.7, 1.1 Hz, 2H), 7.73 (d, J = 7.4 Hz, 2H), 7.63–7.55 (m, 6H), 7.52 (s, 2H), 7.40 (ddd, J = 8.4, 7.1, 1.3 Hz, 2H), 7.31 (dddd, J = 15.9, 8.2, 7.1, 1.1 Hz, 4H), 7.23 (d, J = 8.6 Hz, 2H), 7.19 (d, J = 8.5 Hz, 2H), 7.12–7.06 (m, 6H), 6.83–6.77 (m, 4H), 6.61–6.54 (m, 2H).

^{19}F-NMR (471 MHz, CD_2Cl_2): δ = -136.71 (d, J = 21.3 Hz, 4F), -145.73 (t, J = 21.5 Hz, 2F), -159.61 (t, J = 20.7 Hz, 4F).

^{31}P-NMR (203 MHz, CD_2Cl_2): δ = -16.65.

^{13}C-NMR (126 MHz, CD_2Cl_2): δ = 154.49, 154.32, 149.80, 149.43, 142.45 (t, J = 5.4 Hz), 141.72 (t, J = 5.0 Hz), 132.02, 132.01, 131.82, 131.80, 129.67, 129.58, 129.31, 129.18, 128.78, 128.03, 128.00, 127.89, 127.73, 127.43, 127.36, 127.09, 125.77, 124.87, 123.65, 123.16 (t, J = 1.6 Hz), 122.73, 122.35, 122.07 (t, J = 1.5 Hz), 121.77 (t, J = 1.8 Hz), 121.72, 111.03, 110.80, 108.90, 107.99.

ESI-HRMS: calculated for $C_{84}H_{40}N_3O_{12}S_2P_2F_{10}$ ([M-H]$^-$): 1598.137460, found: 1598.136910.

$[\alpha]_D^{25}$ = +492.8 (*c* = 0.39, $CHCl_3$).

(*S*,*S*)-(6-(*tert*-butyl)benzofuran-2-yl)-C_6F_5-IDPi (6b)

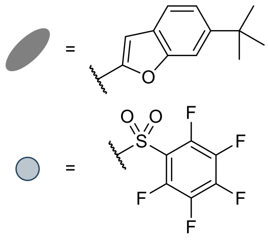

(*S*)-BINOL **S33** (1.02 g, 1.62 mmol, 2.05 eq.) was added to a flame-dried Young-Schlenk charged with $P(NSO_2C_6F_5)Cl_3$ (620 mg, 1.62 mmol, 2.05 eq.) and the solids were gently heated to 50 °C with a heat gun under high vacuum for 10 min. The mixture was placed under argon and dissolved in dry toluene (15 mL). Et_3N (0.89 mL, 6.39 mmol, 8.08 eq.) was added to the reaction and the mixture was stirred at RT for 15 min (formation of a white precipitate was observed). HMDS (165 µL, 0.79 mmol, 1.00 eq.) was added, the mixture was stirred at RT for 15 min and subsequently heated to 120 °C for 3 d. After cooling to RT, the mixture was diluted with DCM and quenched by addition of HCl (1.2 M). The aqueous layer was extracted with DCM (3x), the combined organic phases were dried over anhydrous Na_2SO_4 and concentrated under reduced pressure. The crude material was purified by silica gel flash column chromatography (100% DCM). The product was acidified by filtration over a short plug of DOWEX 50WX8 (H-form, eluted with DCM) and obtained as an off-white solid (1.32 g, 0.724 mmol, 92%).

$\boldsymbol{R}_F$ (hex/EtOAc 4:1) = 0.22.

^{1}H-NMR (501 MHz, CD_2Cl_2): δ = 8.48 (s, 2H), 8.13 (d, J = 8.1 Hz, 2H), 8.04 (d, J = 7.9 Hz, 2H), 7.89 (ddd, J = 8.0, 6.8, 1.1 Hz, 2H), 7.68–7.53 (m, 8H), 7.42 (s, 2H), 7.38 (dd, J = 8.3, 1.6 Hz, 2H), 7.33 (ddd, J = 8.2, 6.7, 1.2 Hz, 4H), 7.23 (d, J = 8.5 Hz, 2H), 7.13 (s, 2H), 7.03 (d, J = 8.2 Hz, 2H), 6.93 (s, 2H), 6.79–6.75 (m, 2H), 6.63 (dd, J = 8.2, 1.7 Hz, 2H), 1.47 (s, 18H), 0.99 (s, 18H).

^{19}F-NMR (471 MHz, CD_2Cl_2): δ = -136.71 (d, J = 21.2 Hz, 4F), -145.82 (t, J = 22.0 Hz, 2F), -159.70 (t, J = 19.7 Hz, 4F).

^{31}P-NMR (203 MHz, CD_2Cl_2): δ = -17.44.

^{13}C-NMR (126 MHz, CD_2Cl_2): δ = 154.85, 154.66, 150.09, 149.41, 149.00, 148.83, 142.43 (t, J = 5.3 Hz), 141.74 (t, J = 5.3 Hz), 132.17, 131.89, 131.78, 131.64, 129.71, 129.14, 128.55, 127.92, 127.68, 127.63, 127.60, 127.39, 127.32, 127.12, 126.82, 126.59, 123.12, 122.44, 122.24, 121.93, 121.66, 121.49, 121.29, 120.72, 108.84, 107.93, 107.63, 107.25, 35.48, 34.89, 31.80, 31.41.

ESI-HRMS: calculated for $C_{100}H_{72}N_3O_{12}S_2F_{10}P_2^-$ ([M-H]$^-$): 1822.387860, found: 1822.388580.

$[\alpha]_D^{25}$ = +450.9 (*c* = 0.50, $CHCl_3$).

(*S*,*S*)-(6-(2-methyladamantan-2-yl)benzofuran-2-yl)-C_6F_5-IDPi (7b)

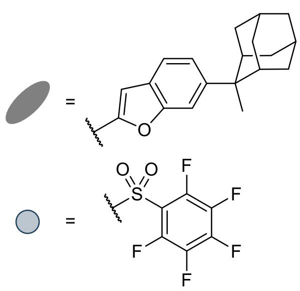

(*S*)-BINOL **S34** (700 mg, 0.859 mmol, 2.01 eq.) was added to a flame-dried Young-Schlenk charged with $P(NSO_2C_6F_5)Cl_3$ (328 mg, 0.858 mmol, 2.01 eq.) and the solids were gently heated to 50 °C with a heat gun under high vacuum for 10 min. The mixture was placed under argon and suspended in dry toluene (20 mL). Et_3N (0.50 mL, 3.59 mmol, 8.44 eq.) was added to the reaction and the mixture was stirred at RT for 1 h. HMDS (89 µL, 0.43 mmol, 1.0 eq.) was added, the mixture was stirred at RT for 10 min and subsequently heated to 120 °C for 4 d. After cooling to RT, the mixture was diluted with DCM and quenched by addition of HCl (1.2 M). The aqueous layer was extracted with DCM (3x), the combined organic phases were dried over anhydrous Na_2SO_4 and concentrated under reduced pressure. The crude material was purified by silica gel flash column chromatography (100% DCM) and a second silica gel flash column chromatography (hex/EtOAc 9:1 to 4:1). The product was acidified by filtration over a plug of DOWEX 50WX8 (H-form, eluted with DCM) and obtained as a white solid (696 mg, 0.317 mmol, 75%).

$\boldsymbol{R_F}$ (hex/EtOAc 4:1) = 0.40.

^{1}H-NMR (501 MHz, CD_2Cl_2): δ = 8.41 (s, 2H), 8.17 (d, J = 8.2 Hz, 2H), 7.96 (d, J = 8.3 Hz, 2H), 7.90 (t, J = 7.5 Hz, 2H), 7.69 (dd, J = 8.4, 2.1 Hz, 2H), 7.62 (ddd, J = 8.4, 6.9, 1.3 Hz, 2H), 7.59 (s, 2H), 7.53 (ddd, J = 8.1, 6.7, 1.1 Hz, 2H), 7.46 (s, 2H), 7.36 (dd, J = 8.4, 1.7 Hz, 2H), 7.33–7.25 (m, 4H), 7.17 (d, J = 8.6 Hz, 2H), 7.14 (s, 2H), 7.07 (d, J = 8.0 Hz, 2H), 6.81 (s, 2H), 6.62 (dd, J = 8.3, 1.6 Hz, 2H), 2.49 (s, 4H), 2.38–2.32 (m, 4H), 2.17–1.94 (m, 14H), 1.91–1.81 (m, 6H), 1.77 (s, 6H), 1.72–1.48 (m, 14H), 1.404–1.36 (m, 10H), 0.99–0.84 (m, 10H).

^{19}F-NMR (471 MHz, CD_2Cl_2) δ = -136.48 (d, J = 21.9 Hz, 4F), -146.02 (s, 2F), -159.61 (t, J = 20.4 Hz, 4F).

^{31}P-NMR (203 MHz, CD_2Cl_2) δ = -17.14.

^{13}C-NMR (126 MHz, CD_2Cl_2) δ = 155.51, 154.98, 149.82, 149.24, 148.89, 148.82, 142.19 (t, J = 5.1 Hz), 141.60 (t, J = 4.9 Hz), 132.21, 131.84, 131.76, 131.48, 129.56, 129.02, 128.11, 127.88, 127.58, 127.54, 127.52, 127.48, 127.22, 126.95, 126.64, 126.43, 123.12, 122.21, 121.82, 121.60, 121.45, 120.36, 108.93, 108.27, 108.14, 107.65, 44.36, 43.92, 39.27, 39.02, 35.32, 35.27, 35.08, 34.80, 34.46, 34.41, 34.06, 33.84, 33.61, 33.37, 33.24, 30.78, 30.74, 28.64, 28.29, 28.28, 28.02.

ESI-HRMS: calculated for $C_{128}H_{104}F_{10}N_3O_{12}P_2S_2^-$ ([M-H]$^-$): 2190.638260, found: 2190.639340.

$[\alpha]_D^{25}$ = +350.5 (*c* = 0.33, $CHCl_3$).

8. NMR Spectra

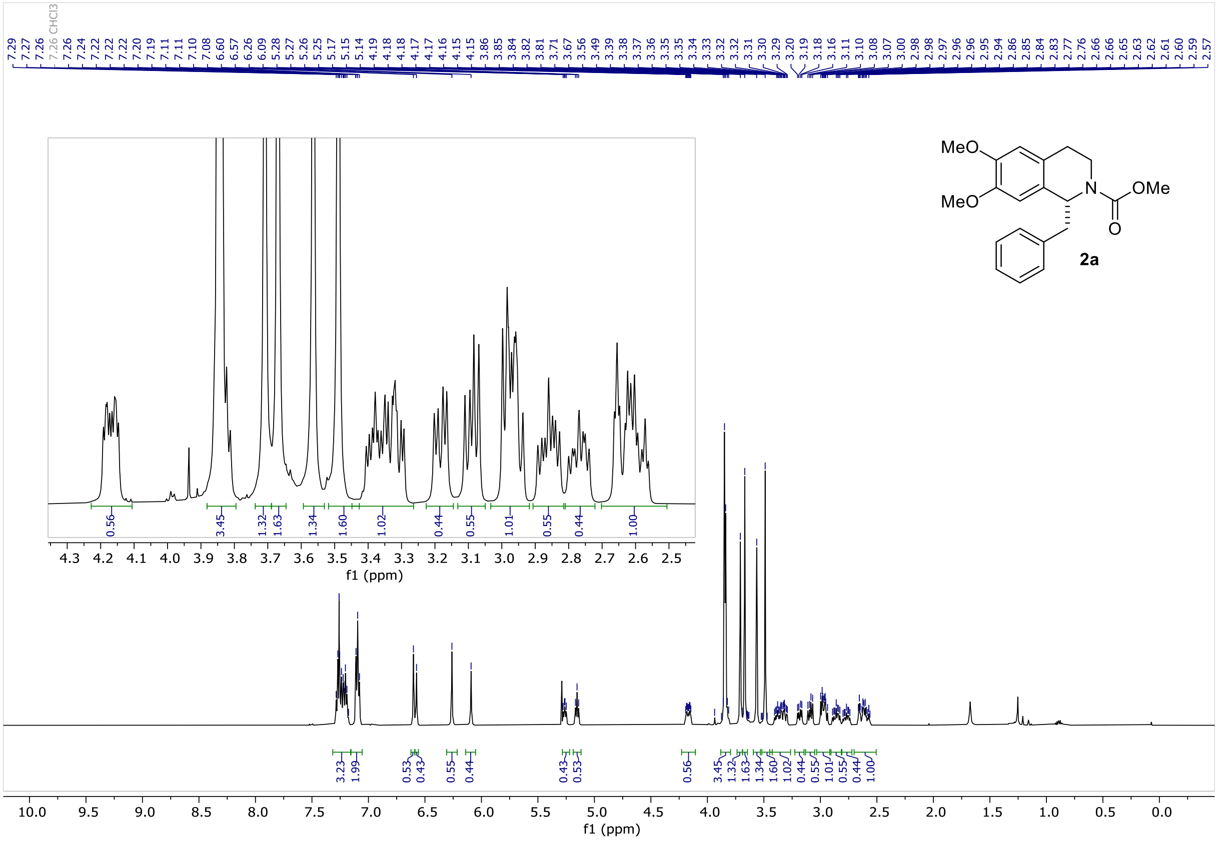

^{1}**H-NMR** spectrum of compound **2a**.

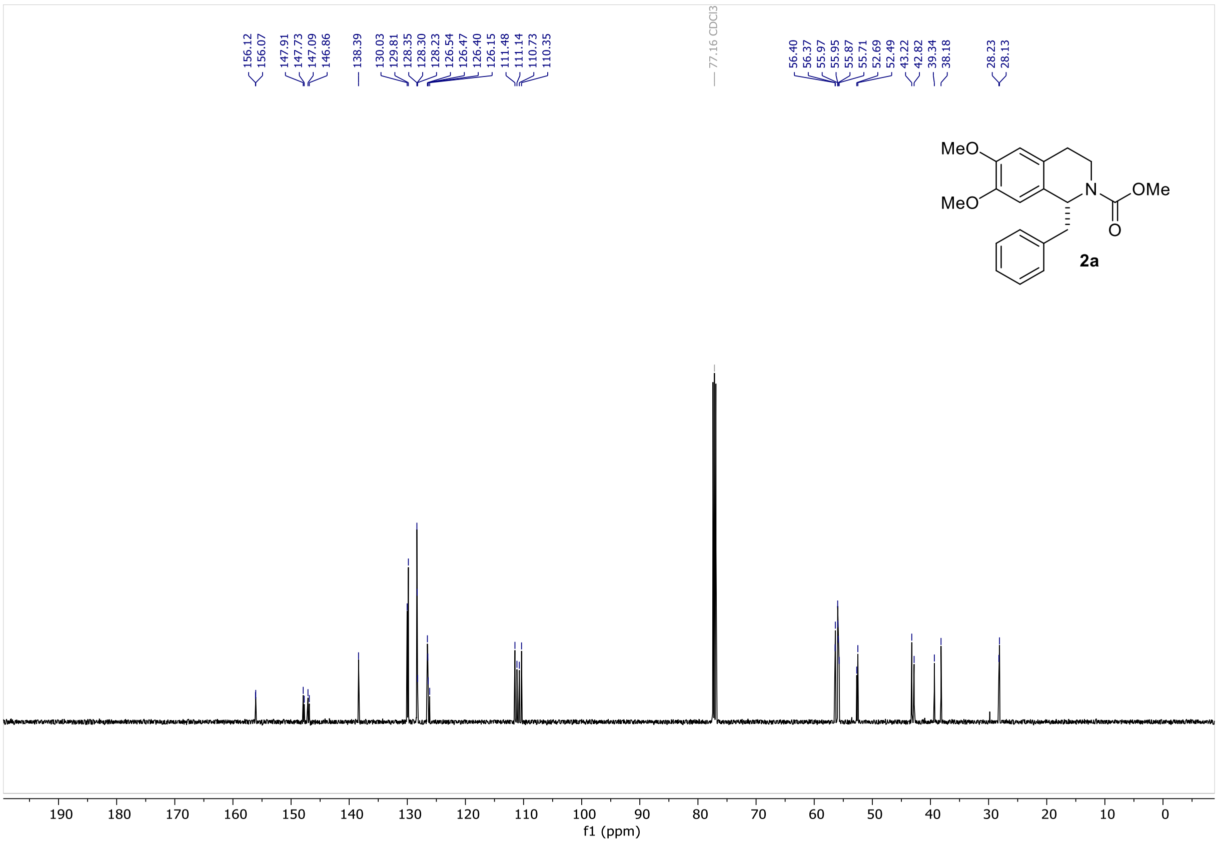

^{13}**C-NMR** spectrum of compound **2a**.

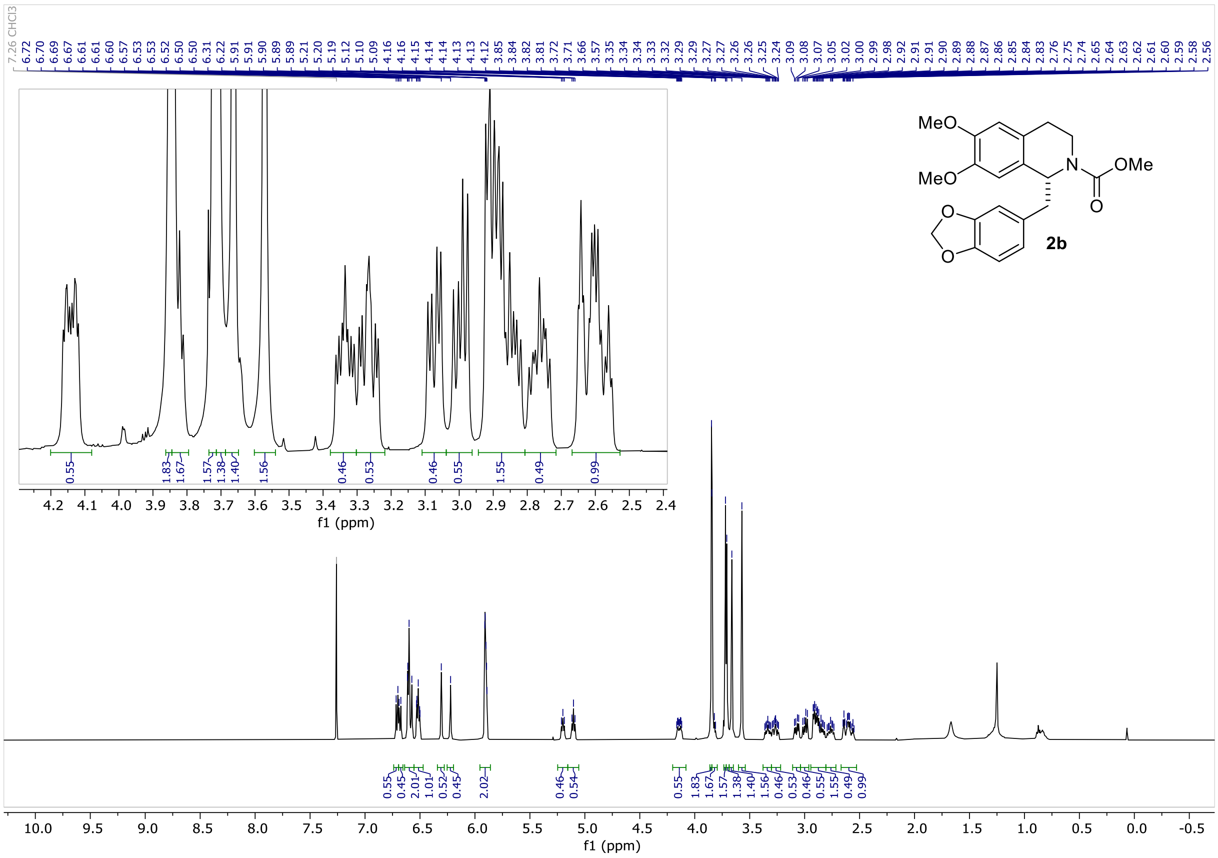

^{1}H-NMR spectrum of compound **2b**.

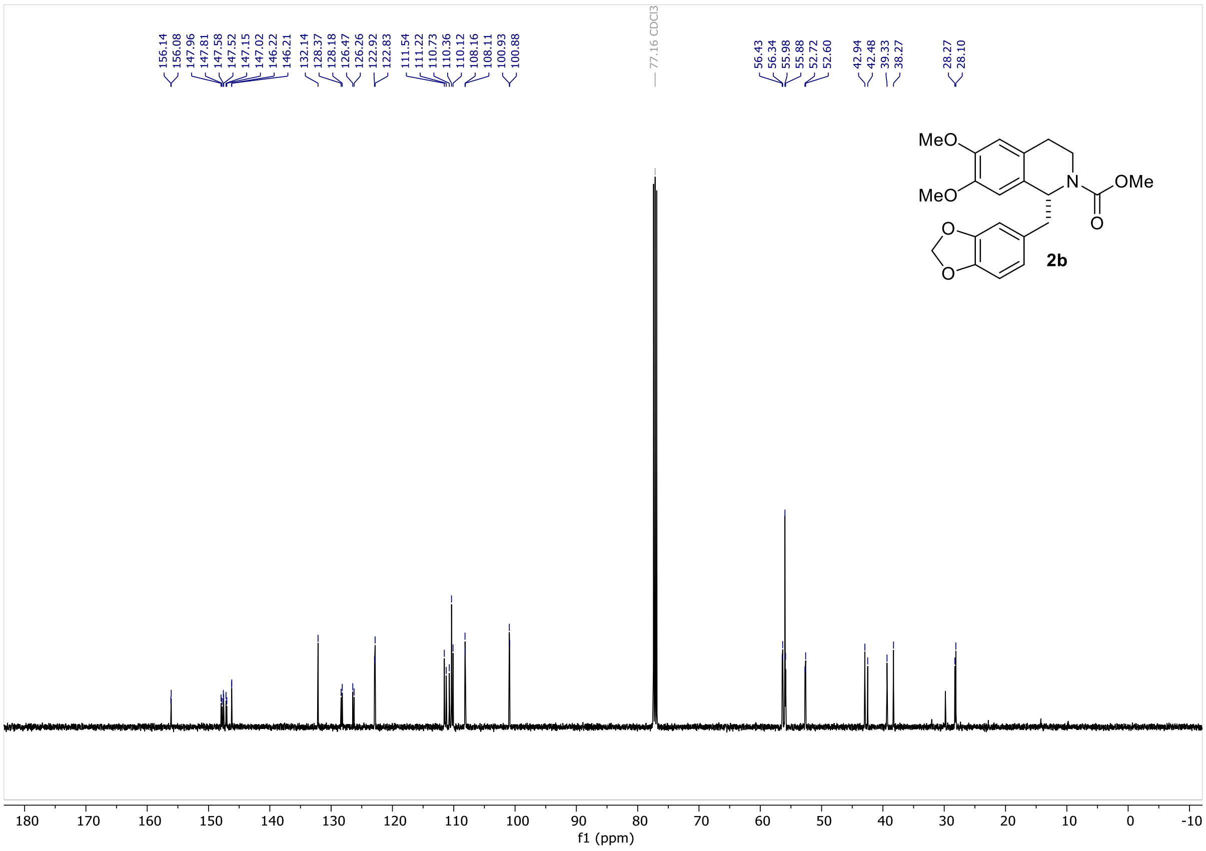

^{13}C-NMR spectrum of compound **2b**.

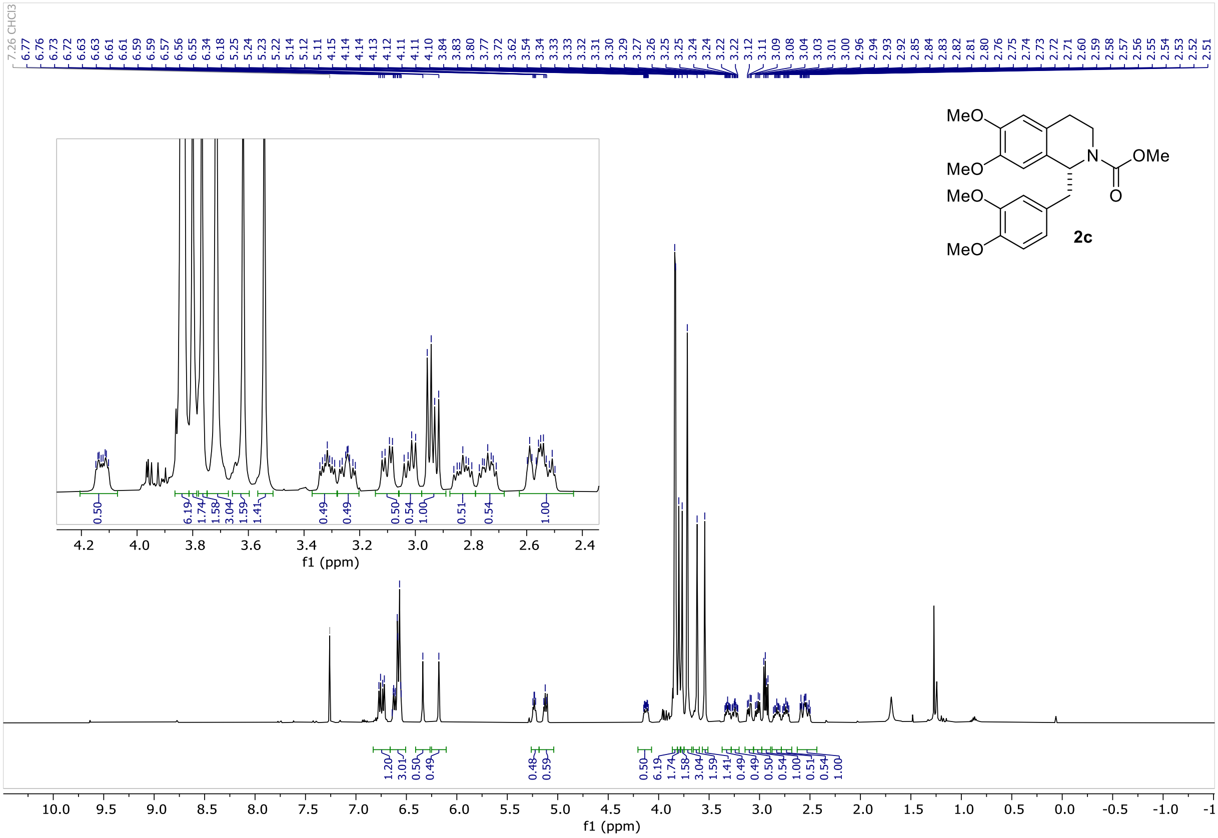

^{1}H-NMR spectrum of compound **2c**.

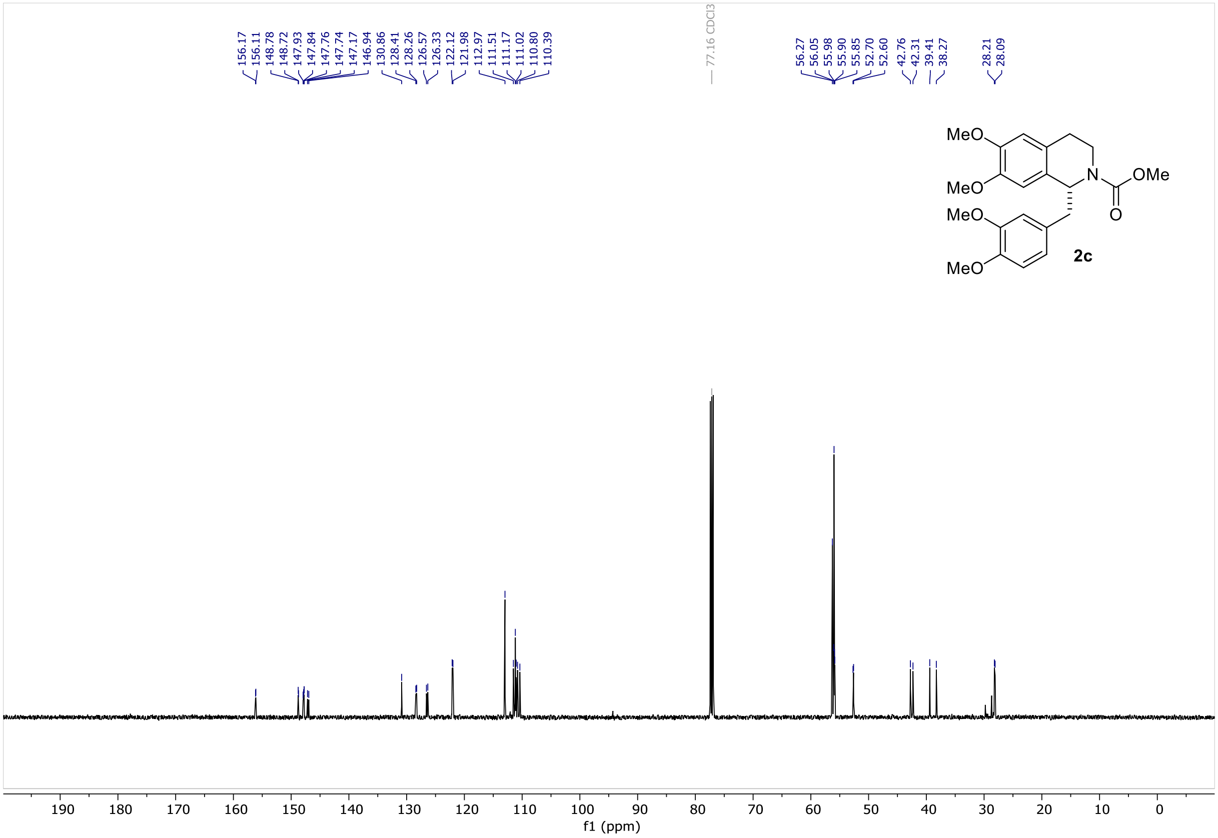

^{13}C-NMR spectrum of compound **2c**.

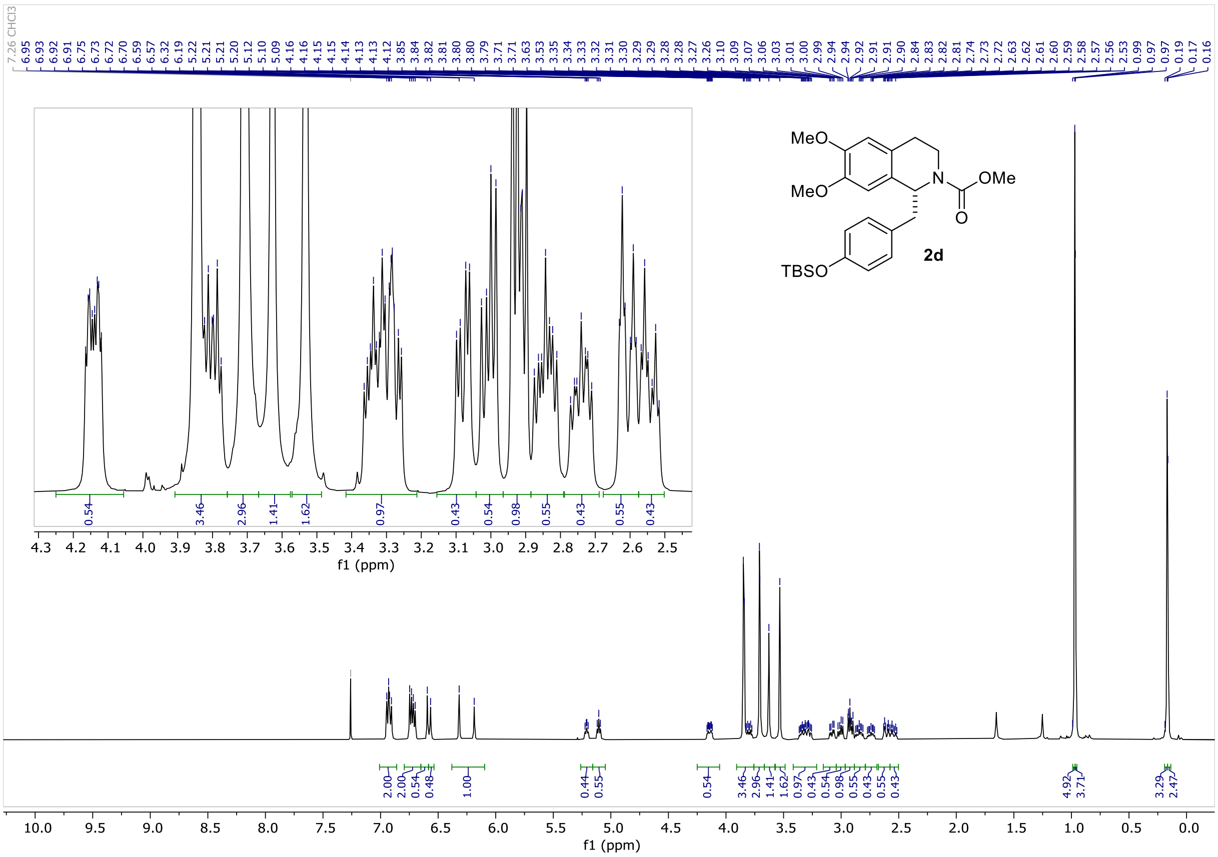

¹H-NMR spectrum of compound **2d**.

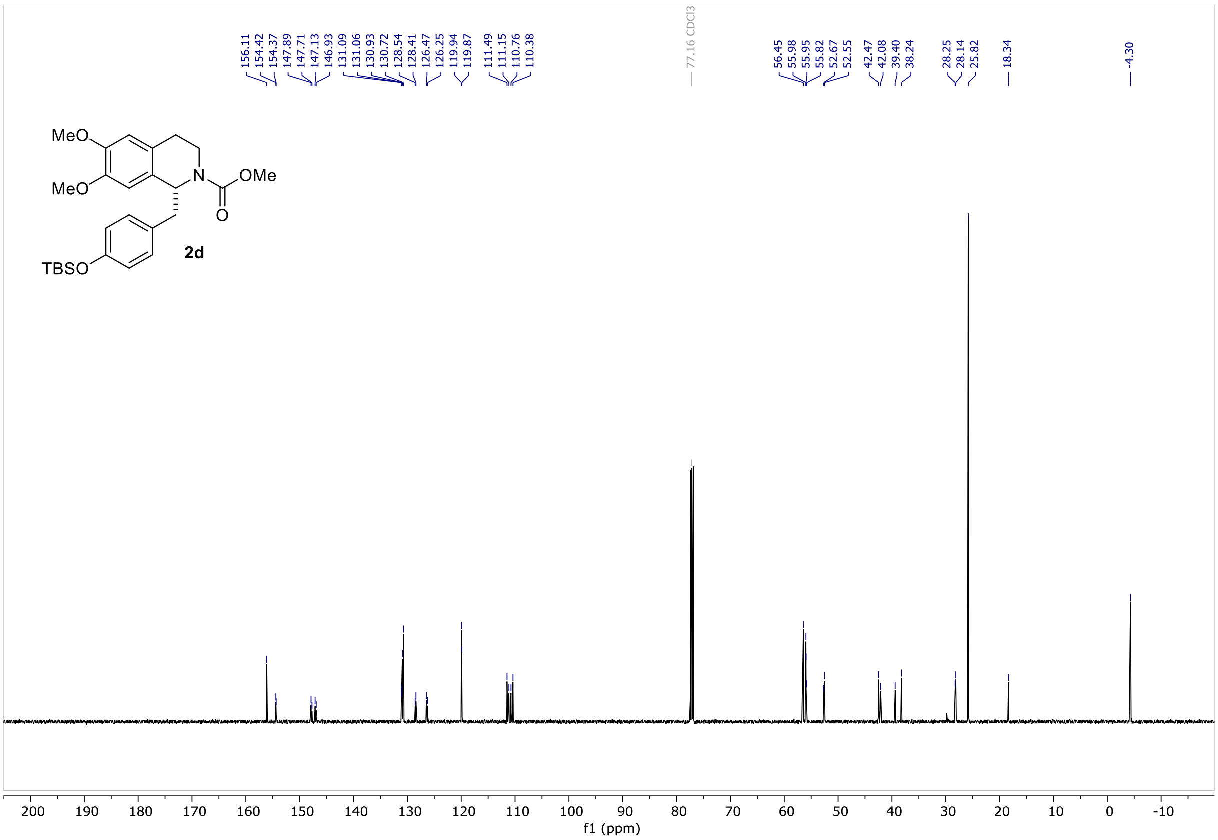

¹³C-NMR spectrum of compound **2d**.

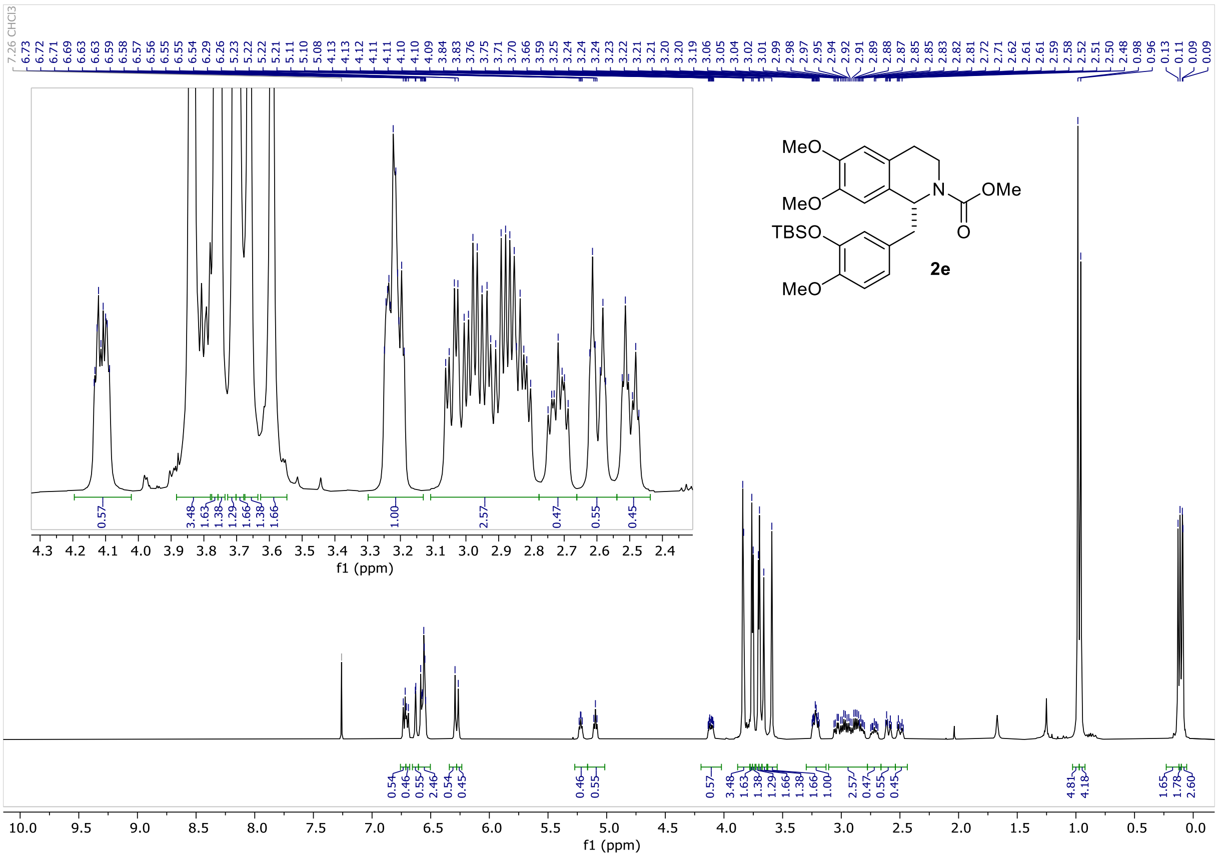

^{1}H-NMR spectrum of compound **2e**.

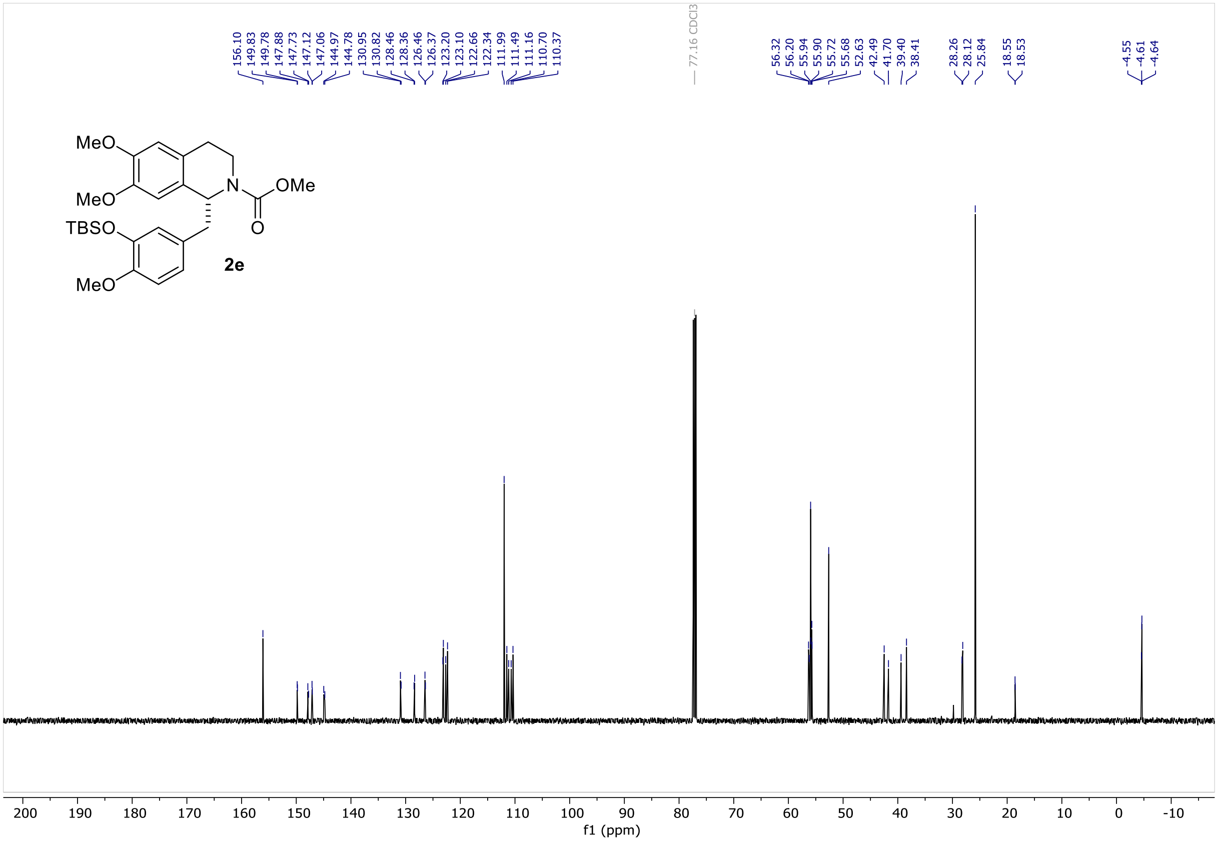

^{13}C-NMR spectrum of compound **2e**.

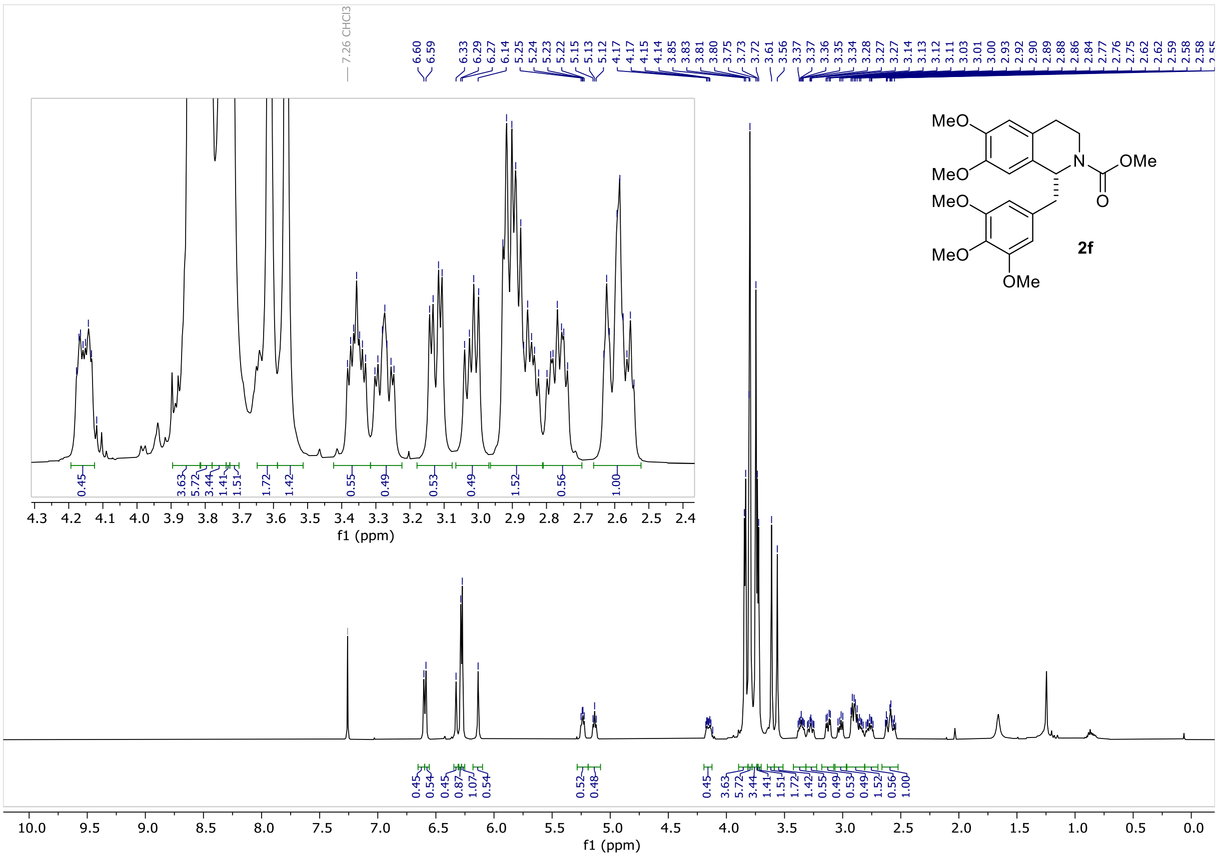

^{1}H-NMR spectrum of compound **2f**.

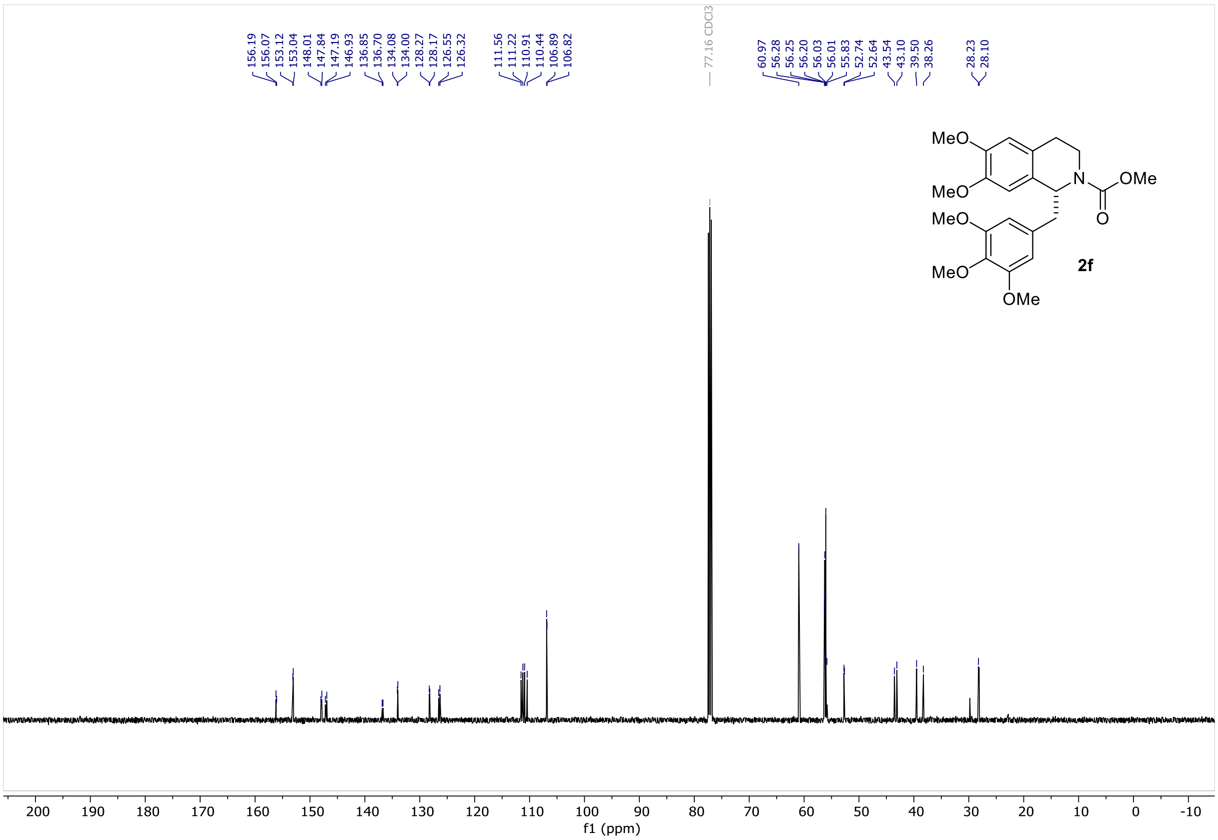

^{13}C-NMR spectrum of compound **2f**.

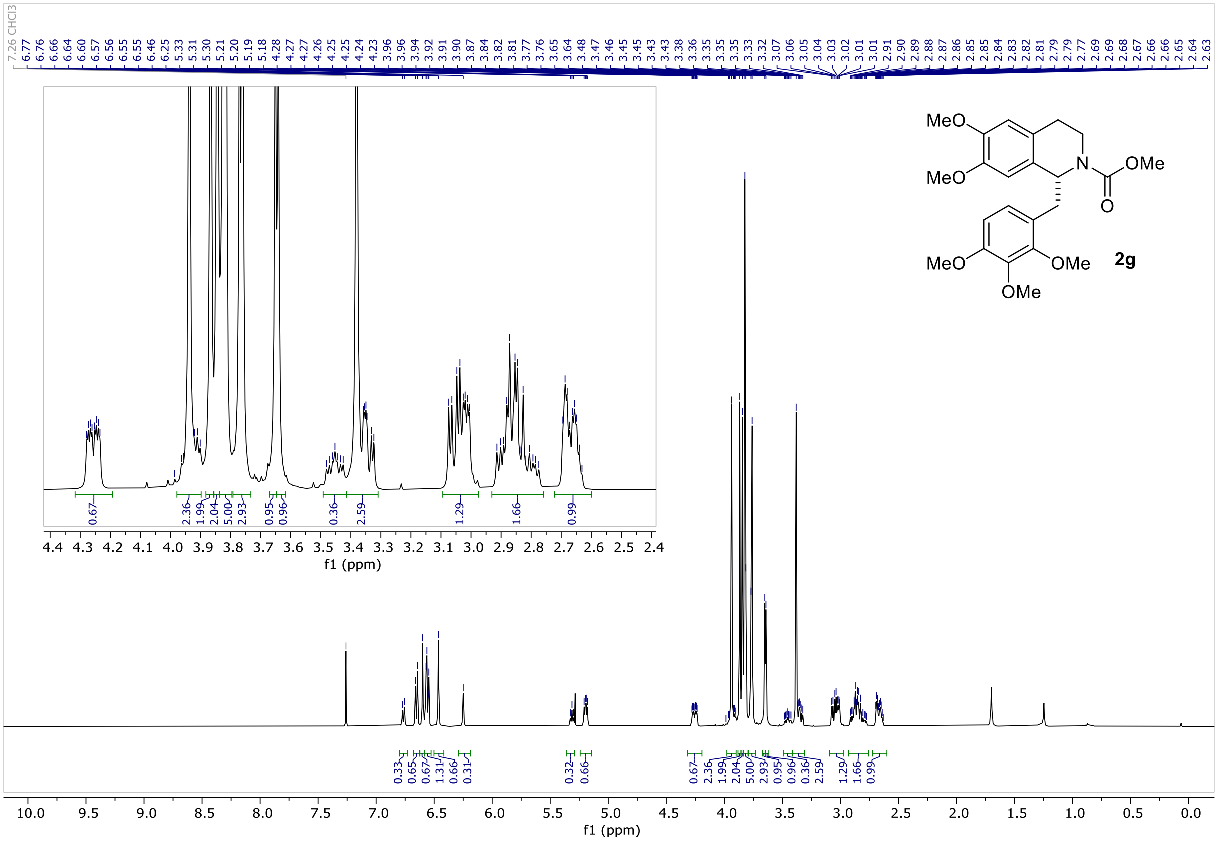

^{1}H-NMR spectrum of compound **2g**.

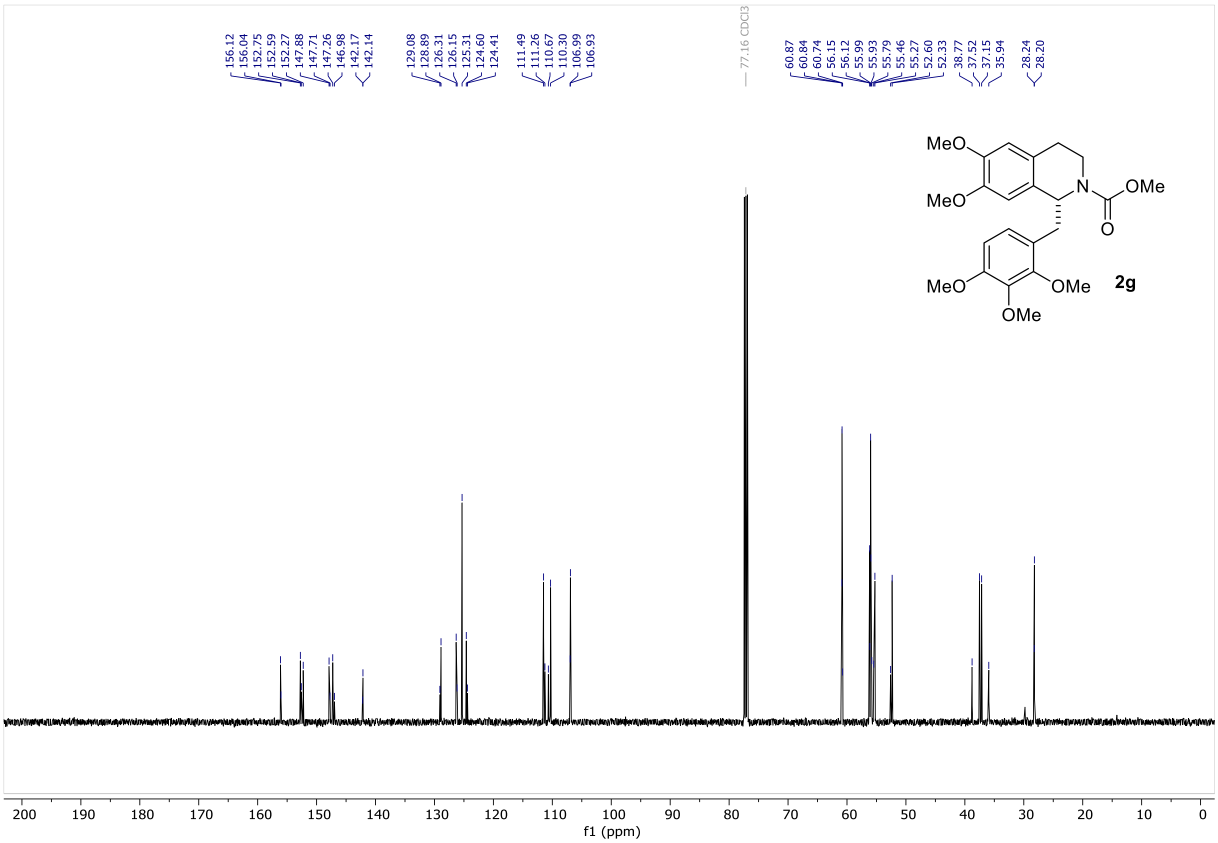

^{13}C-NMR spectrum of compound **2g**.

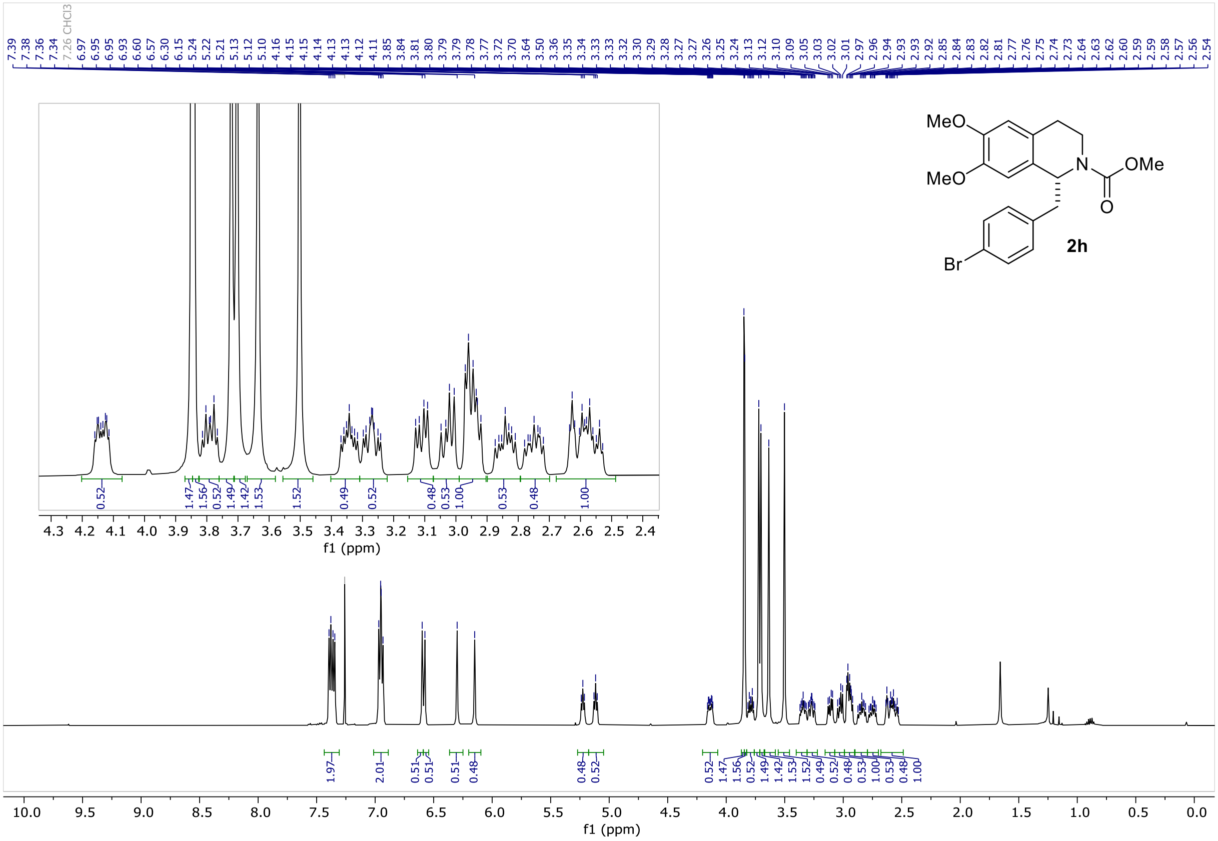

^{1}H-NMR spectrum of compound **2h**.

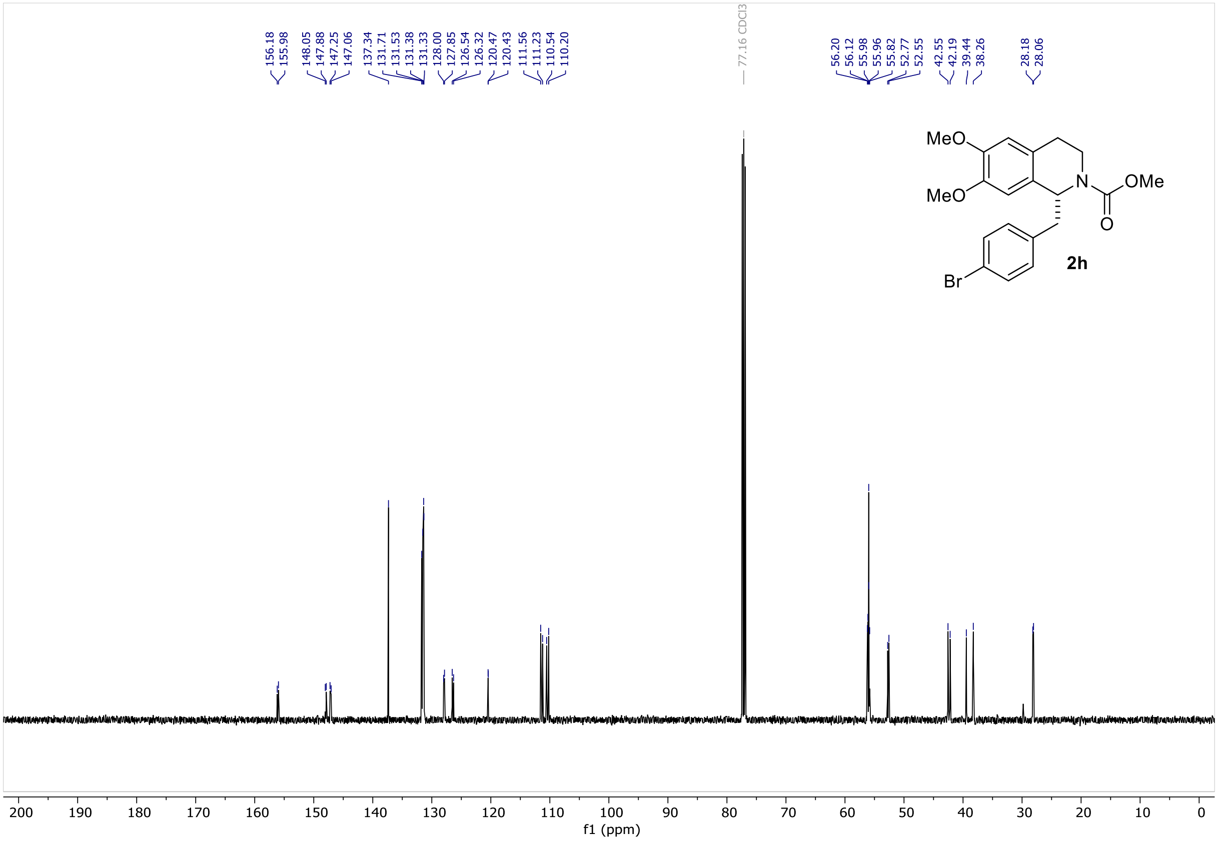

^{13}C-NMR spectrum of compound **2h**.

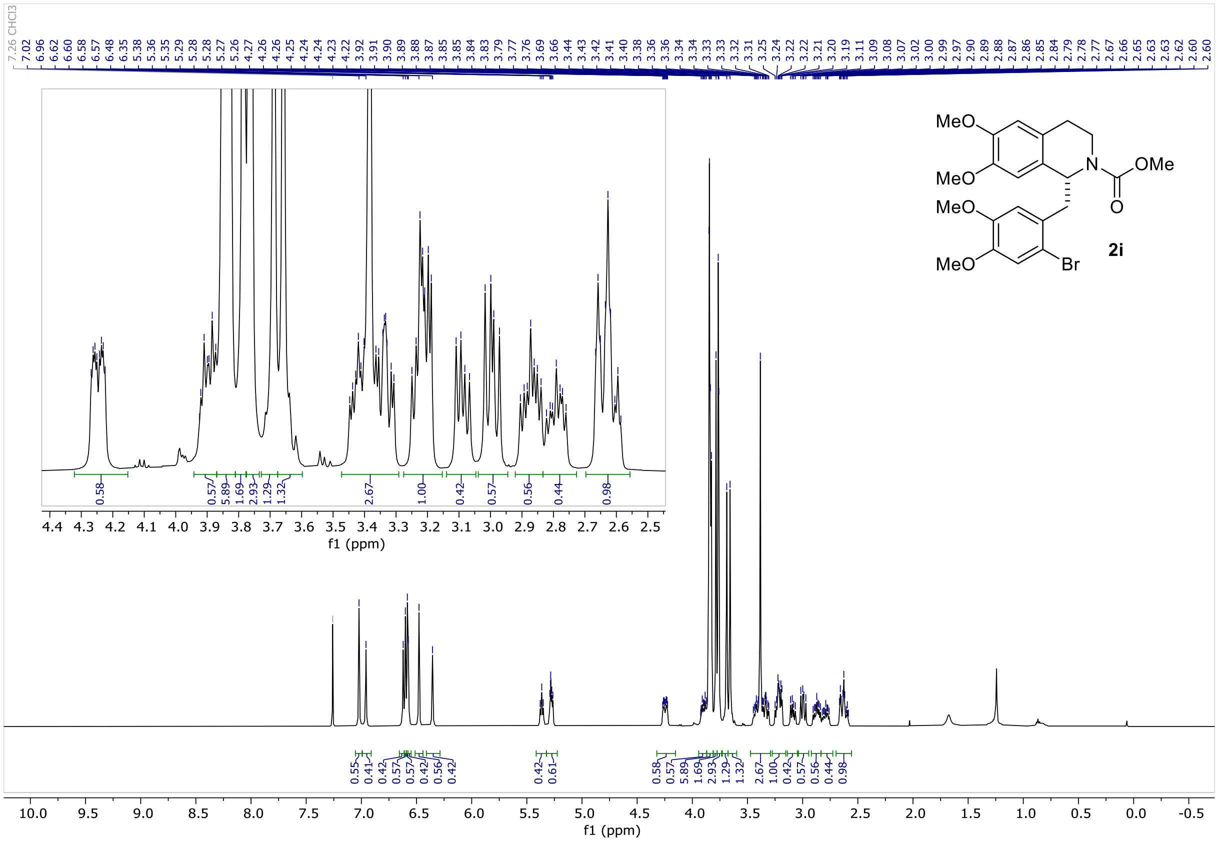

[1]**H-NMR** spectrum of compound **2i**.

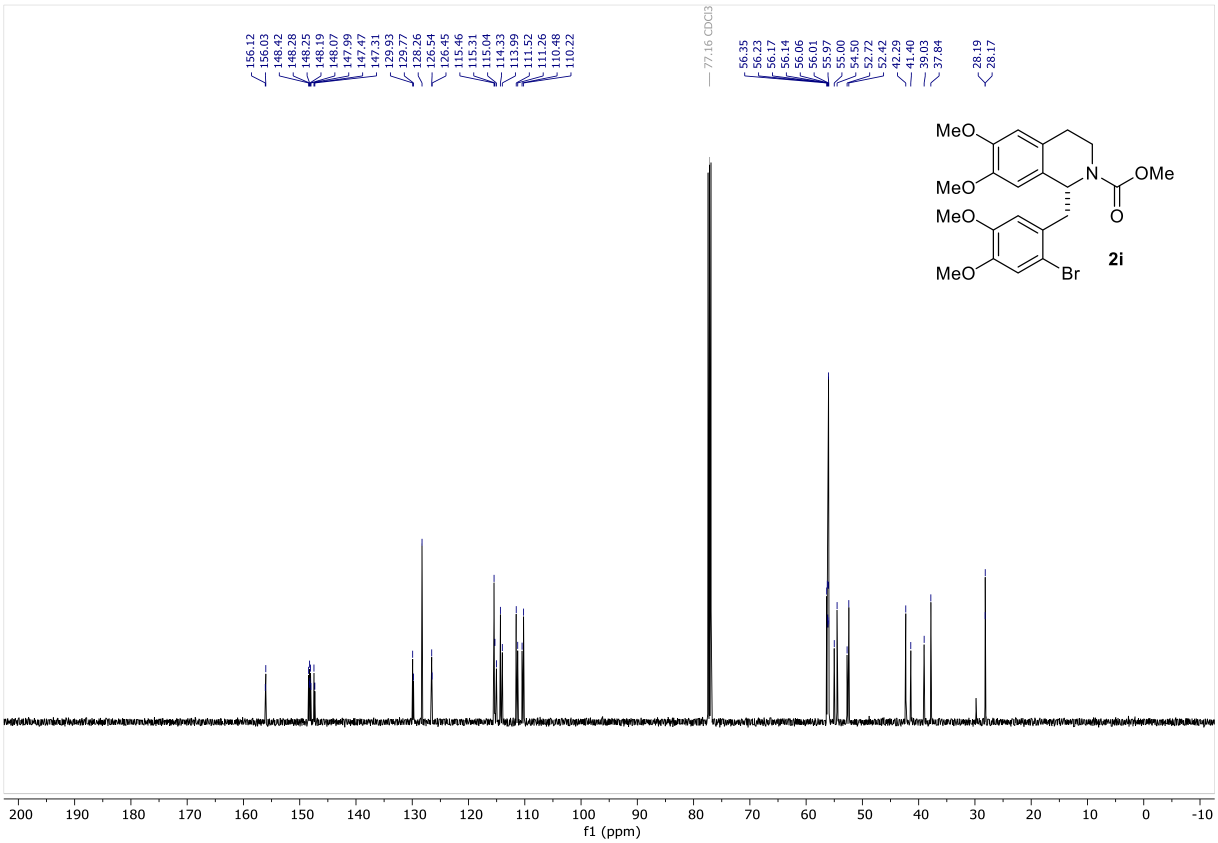

[13]**C-NMR** spectrum of compound **2i**.

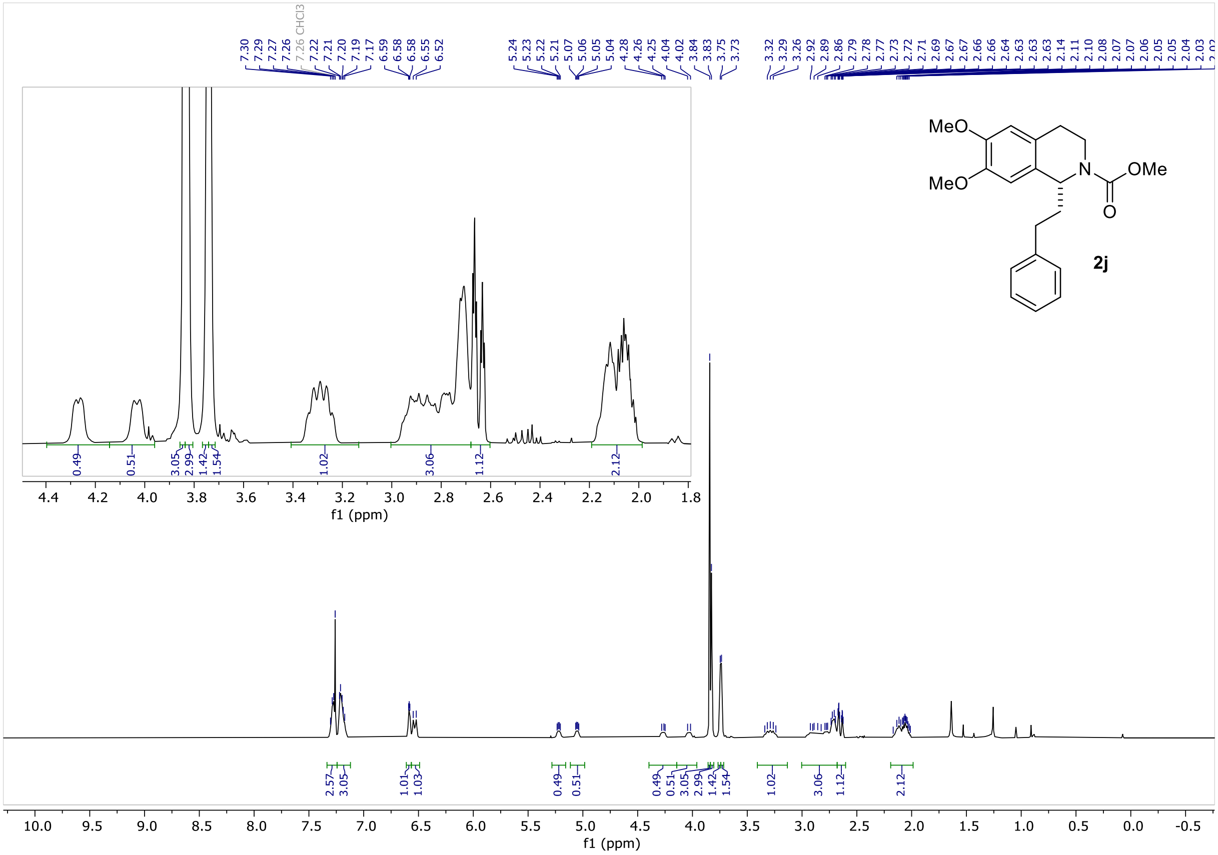

^{1}H-NMR spectrum of compound **2j**.

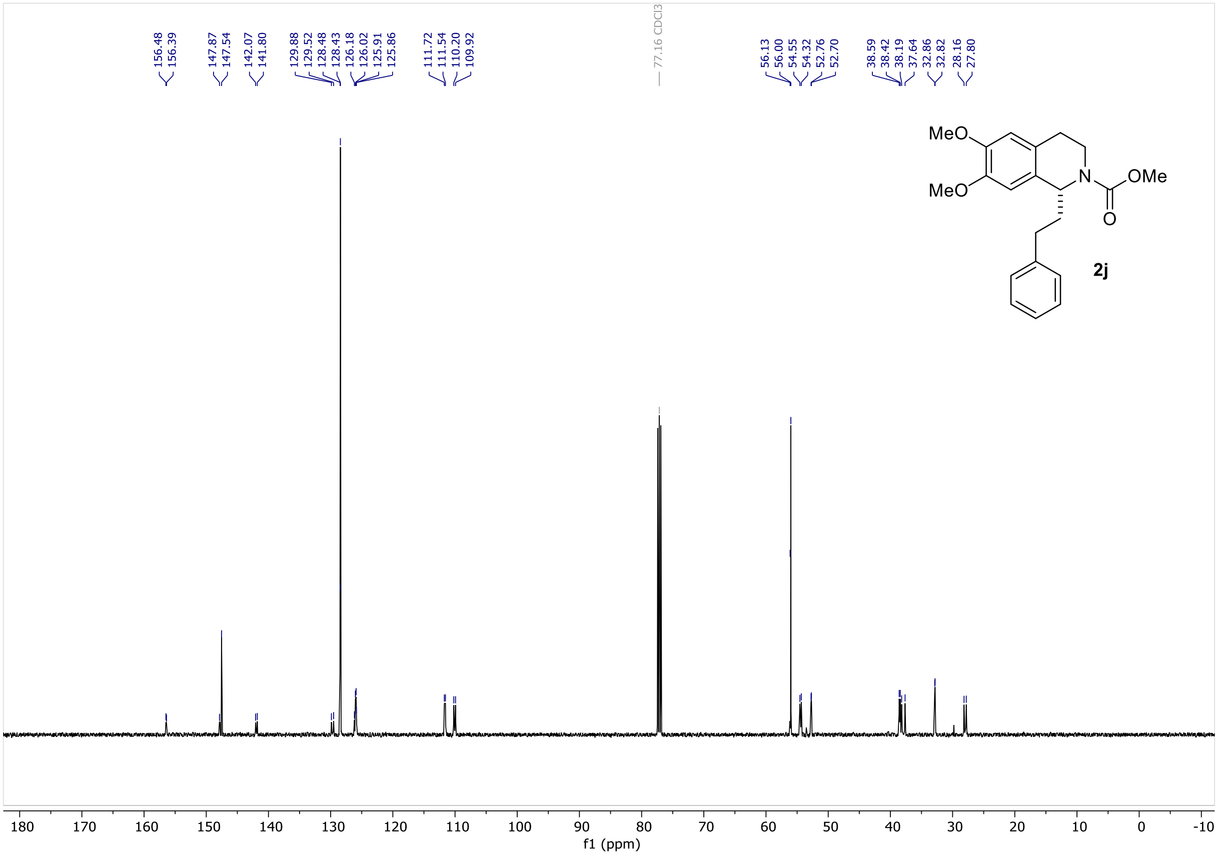

^{13}C-NMR spectrum of compound **2j**.

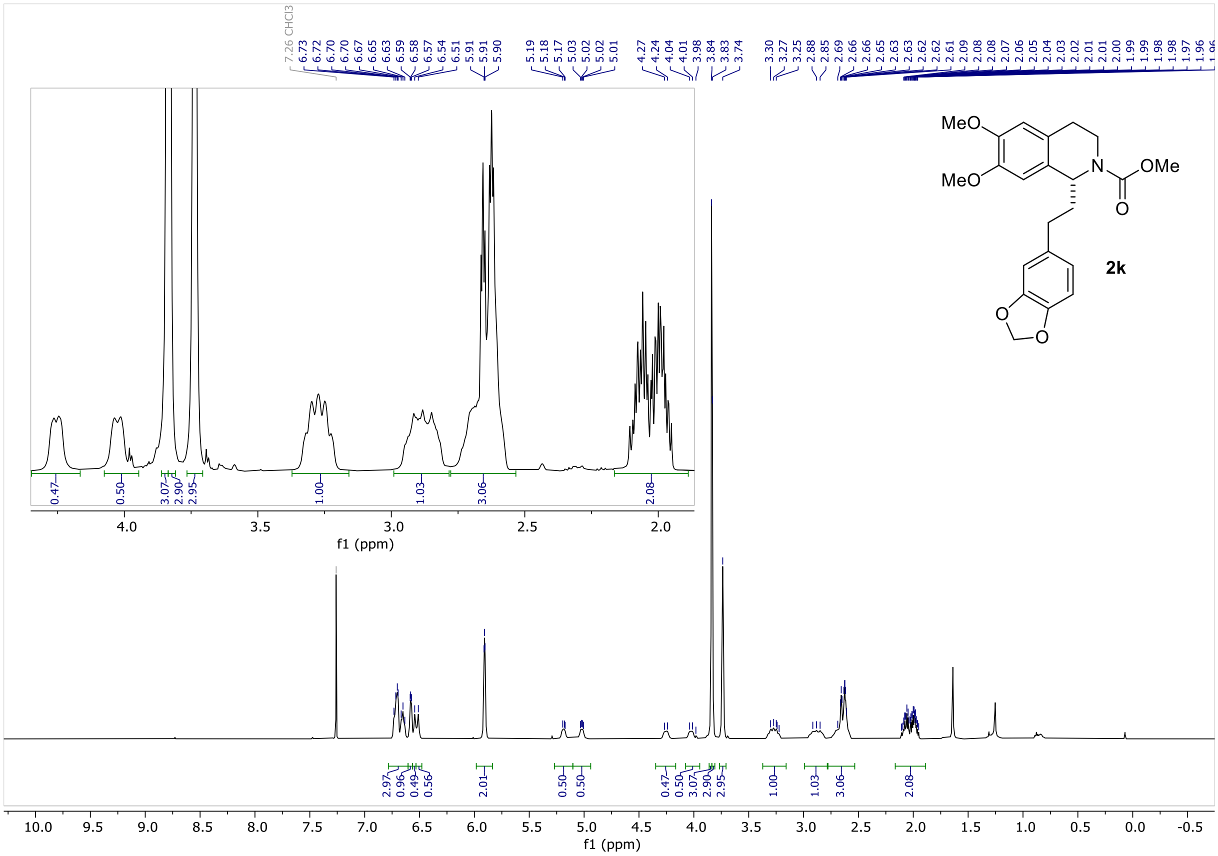

¹H-NMR spectrum of compound **2k**.

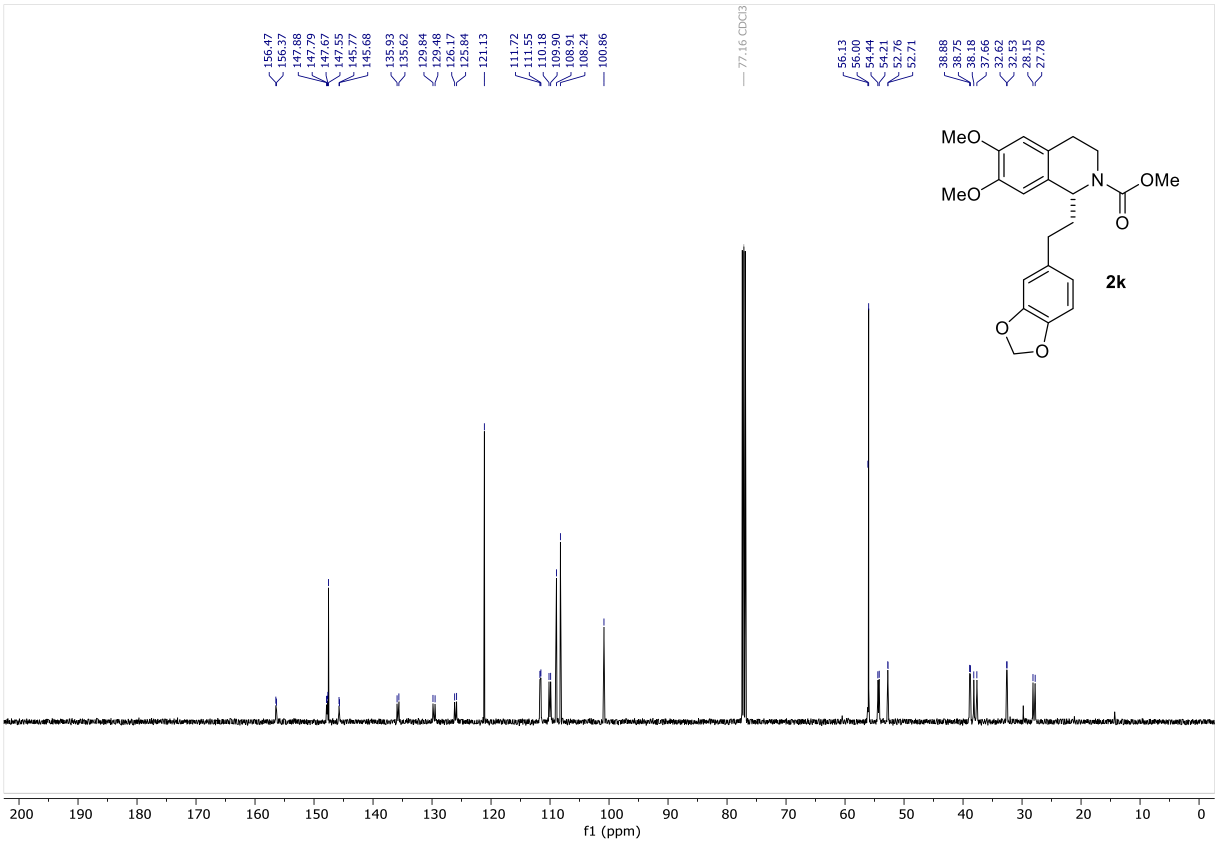

¹³C-NMR spectrum of compound **2k**.

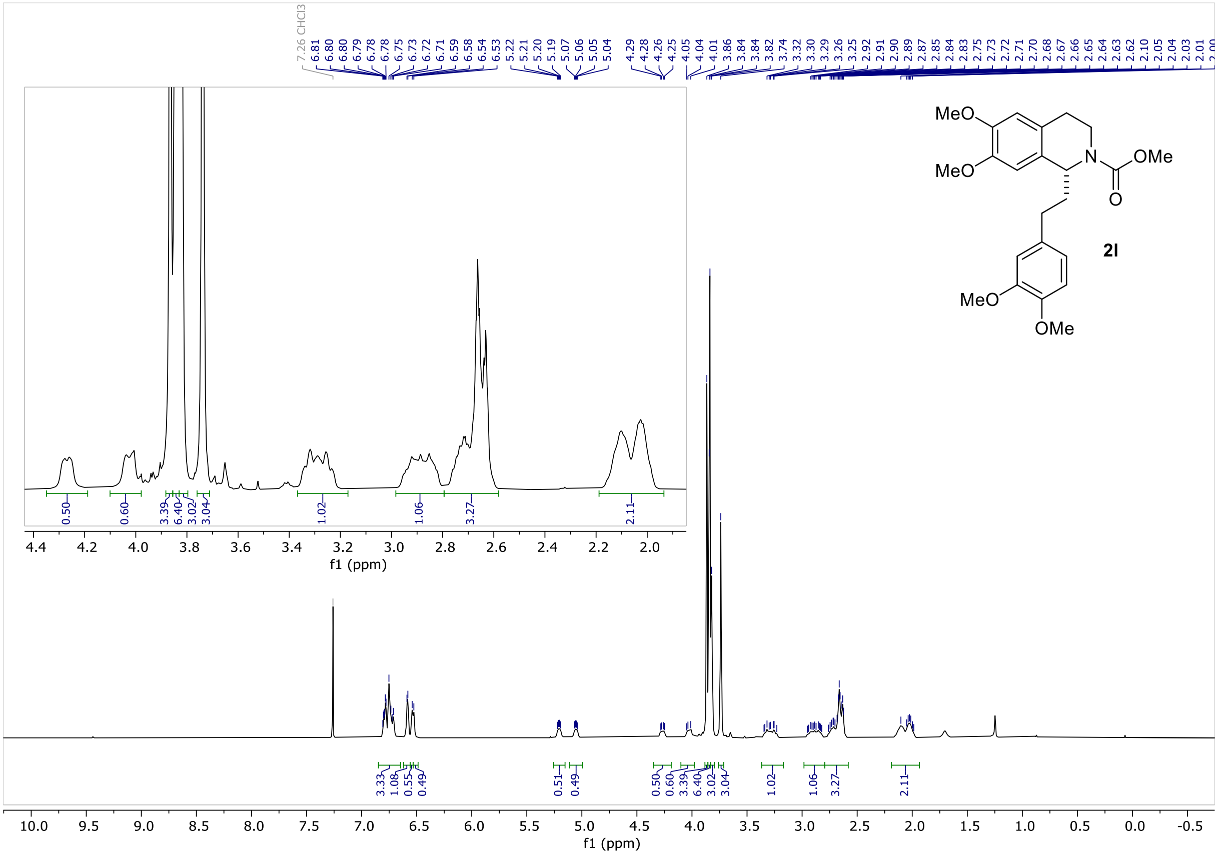

[1]**H-NMR** spectrum of compound **2l**.

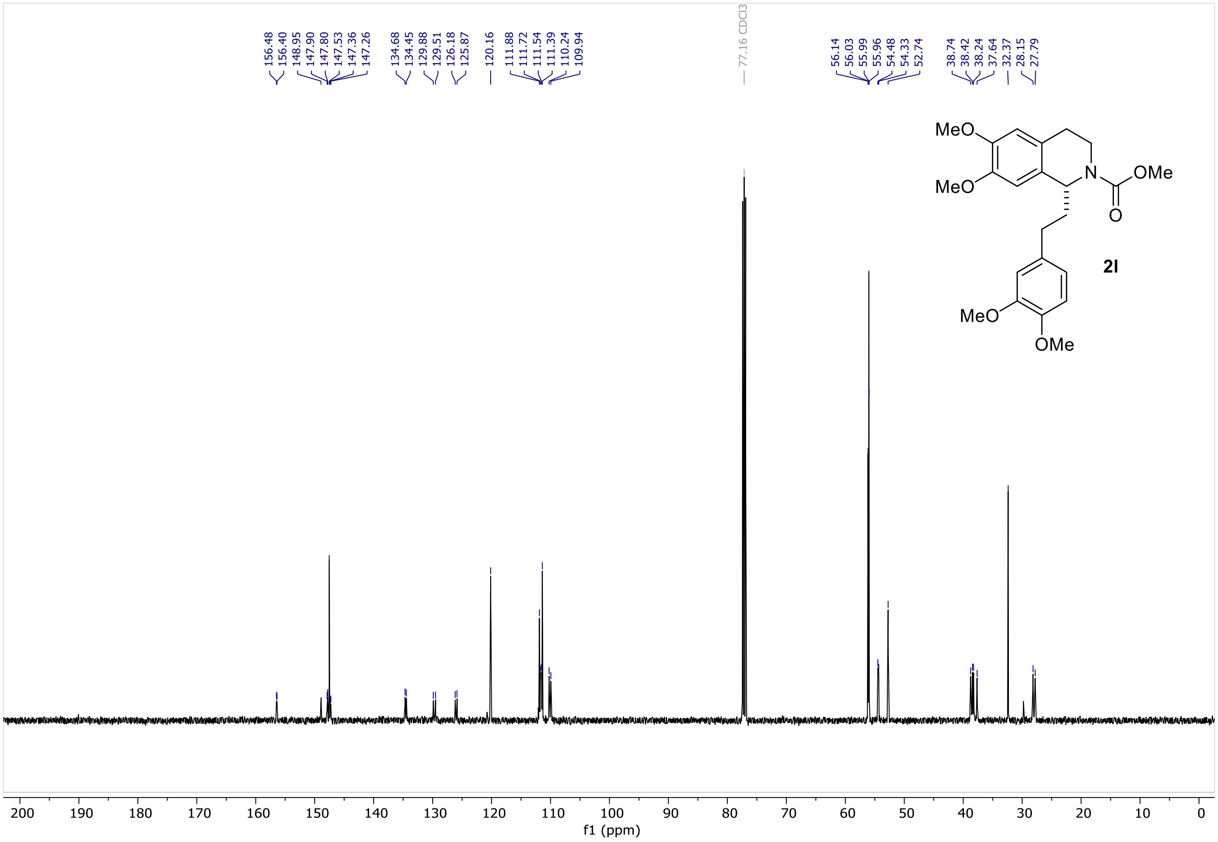

[13]**C-NMR** spectrum of compound **2l**.

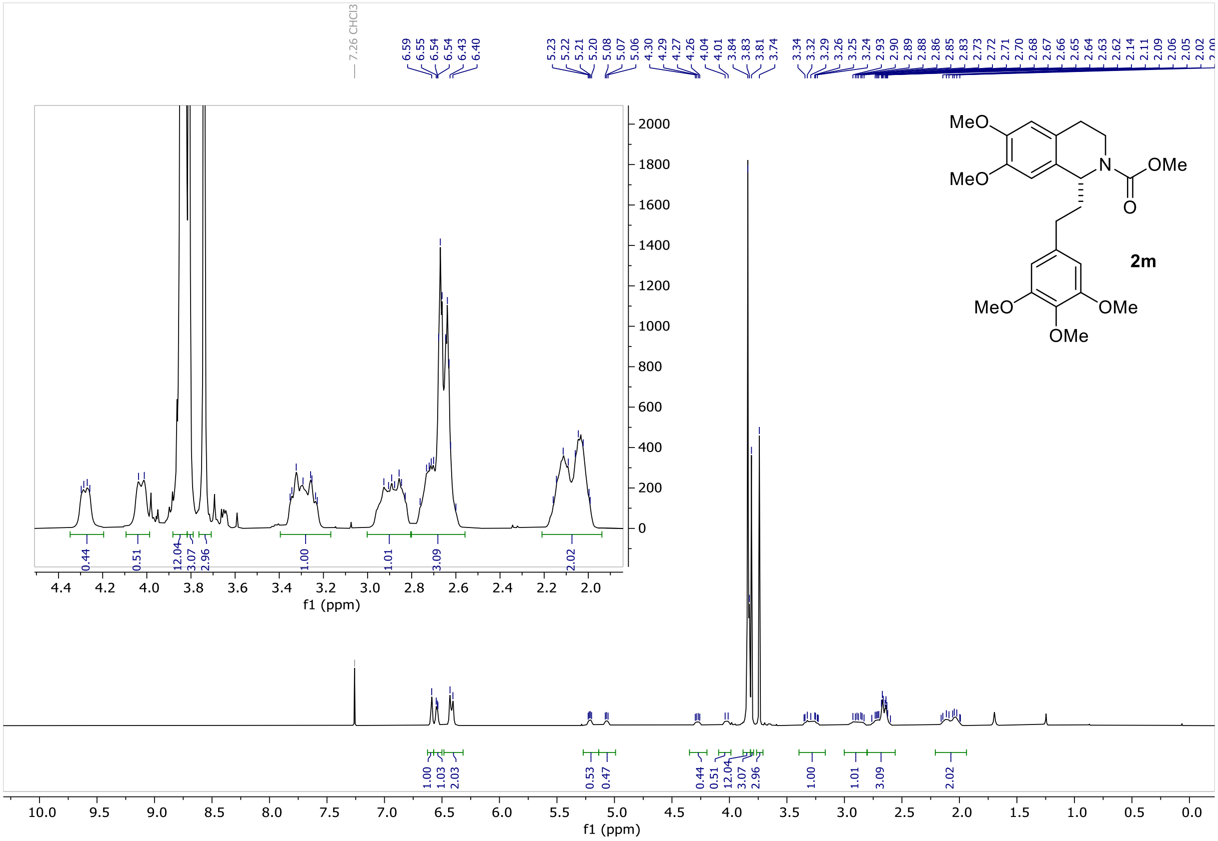

^{1}H-NMR spectrum of compound **2m**.

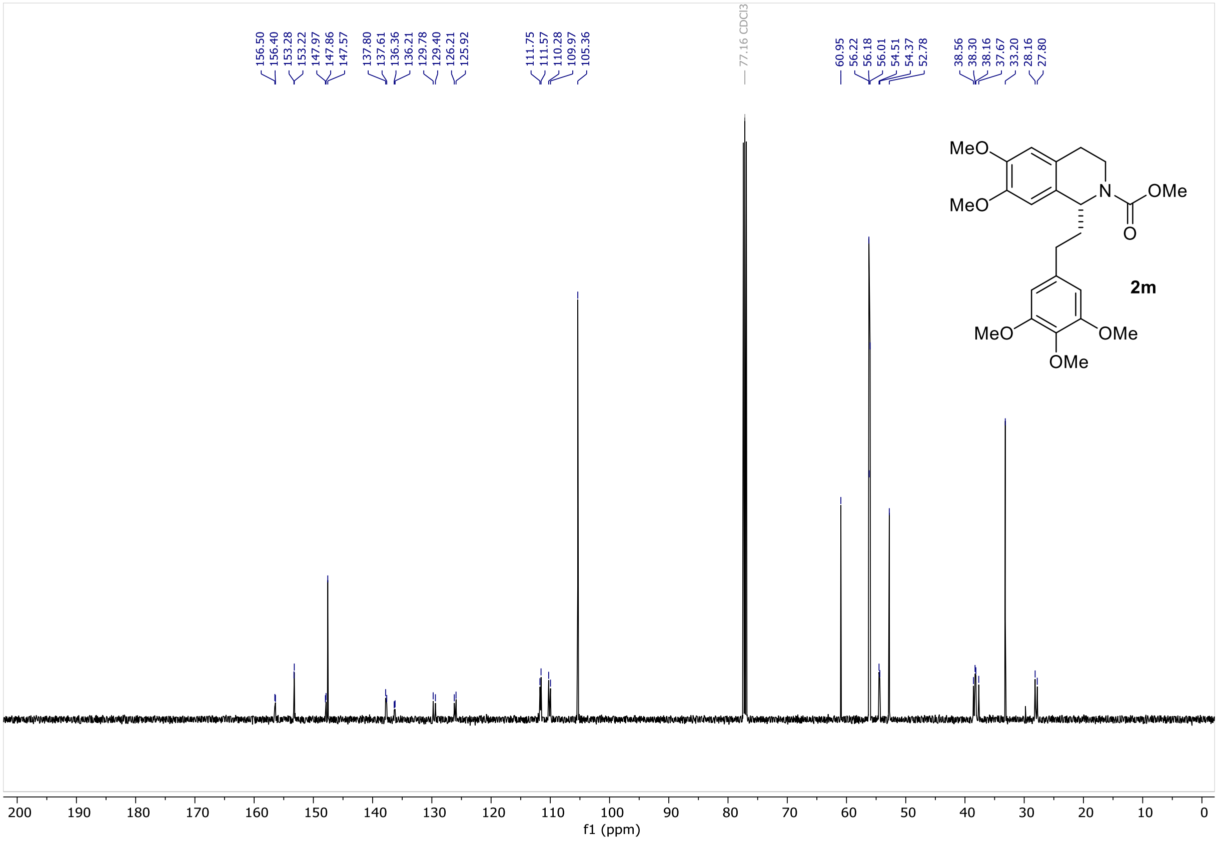

^{13}C-NMR spectrum of compound **2m**.

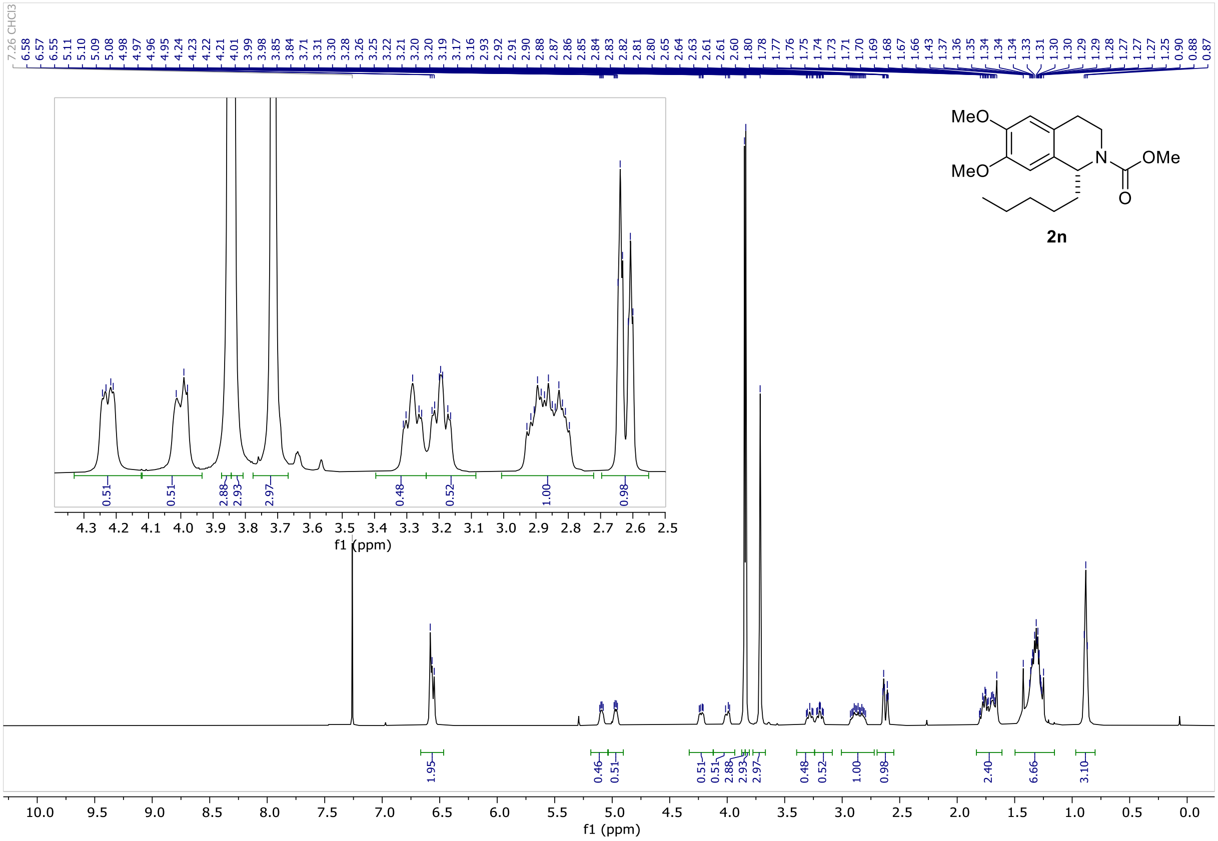

^{1}H-NMR spectrum of compound **2n**.

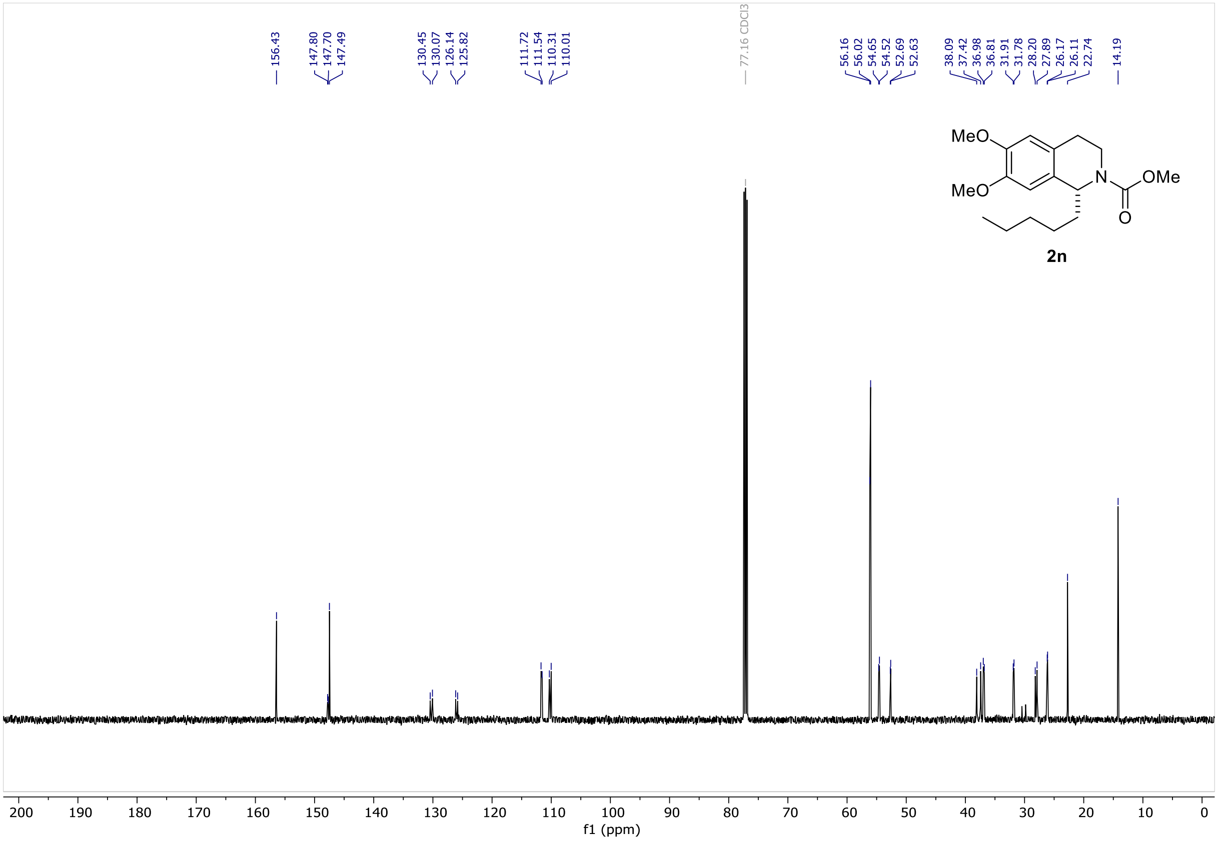

^{13}C-NMR spectrum of compound **2n**.

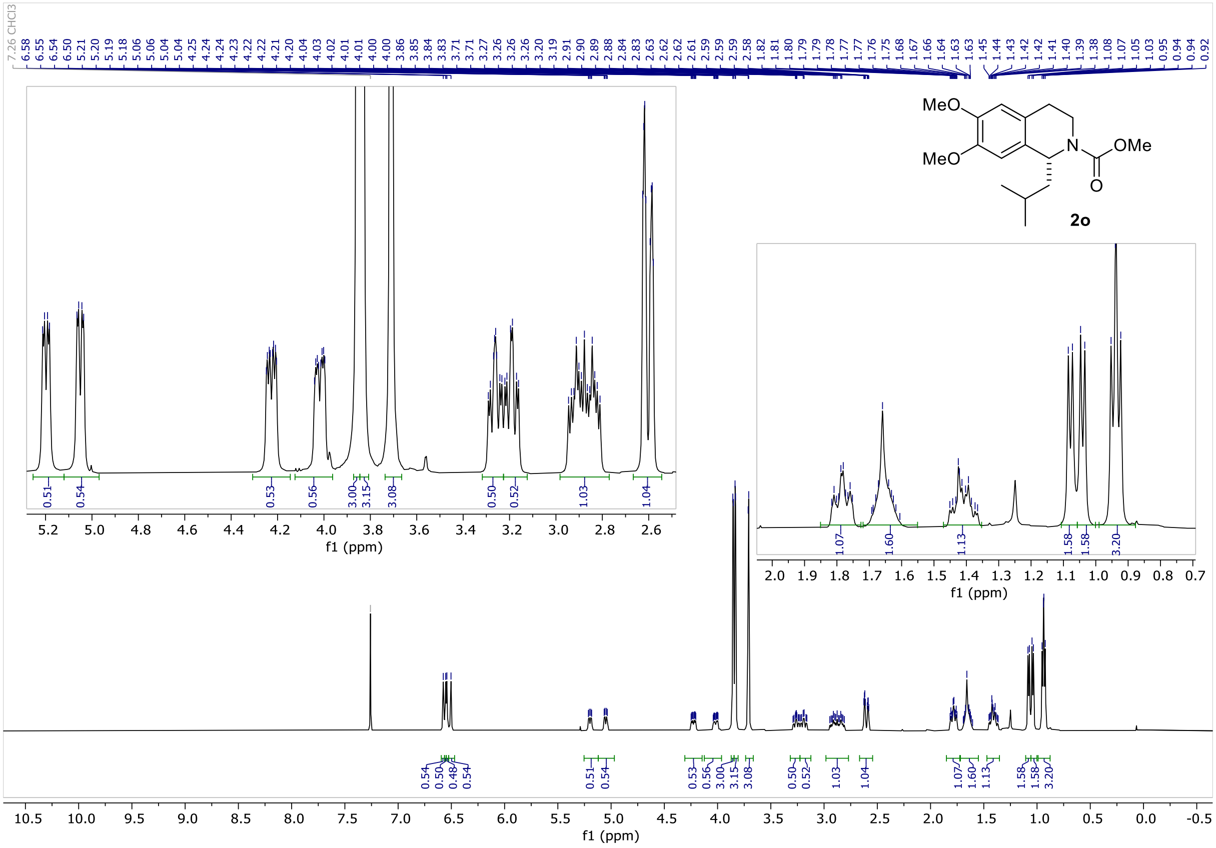

[1]**H-NMR** spectrum of compound **2o**.

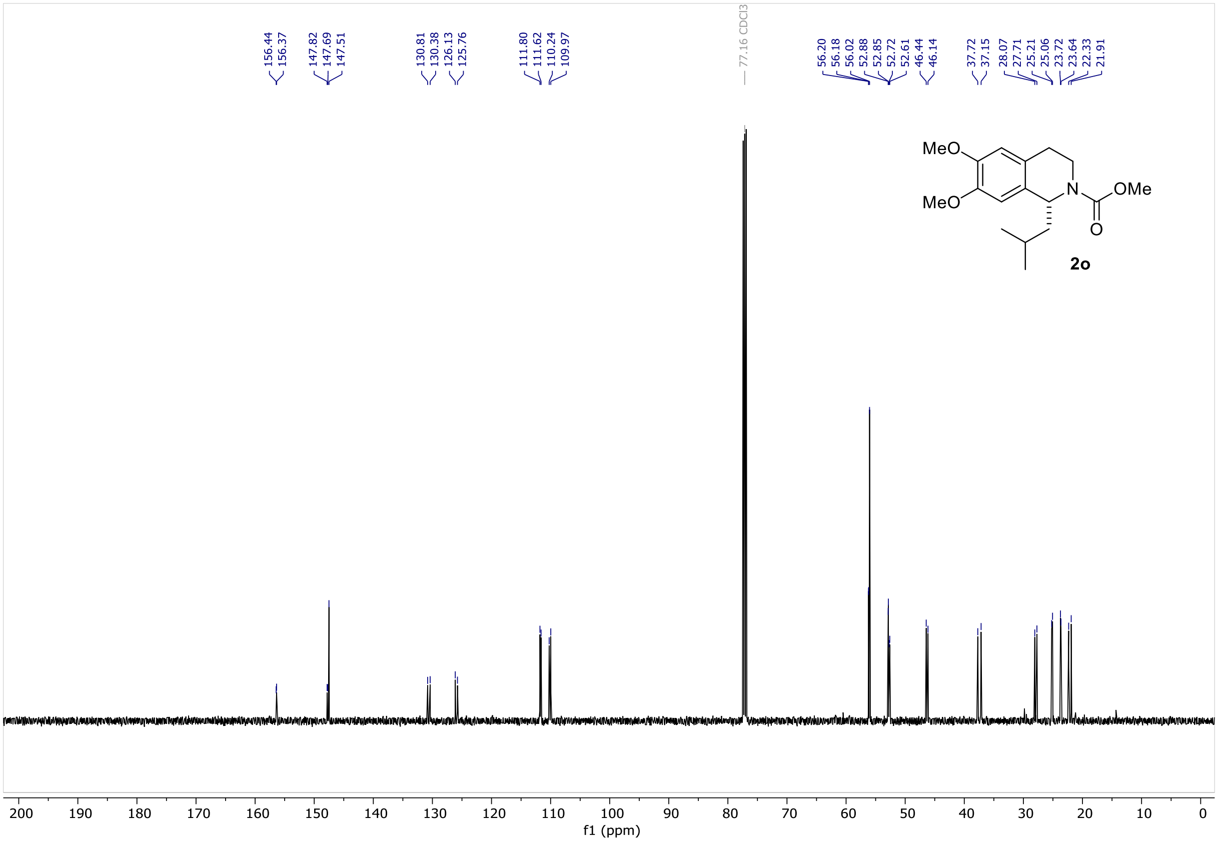

[13]**C-NMR** spectrum of compound **2o**.

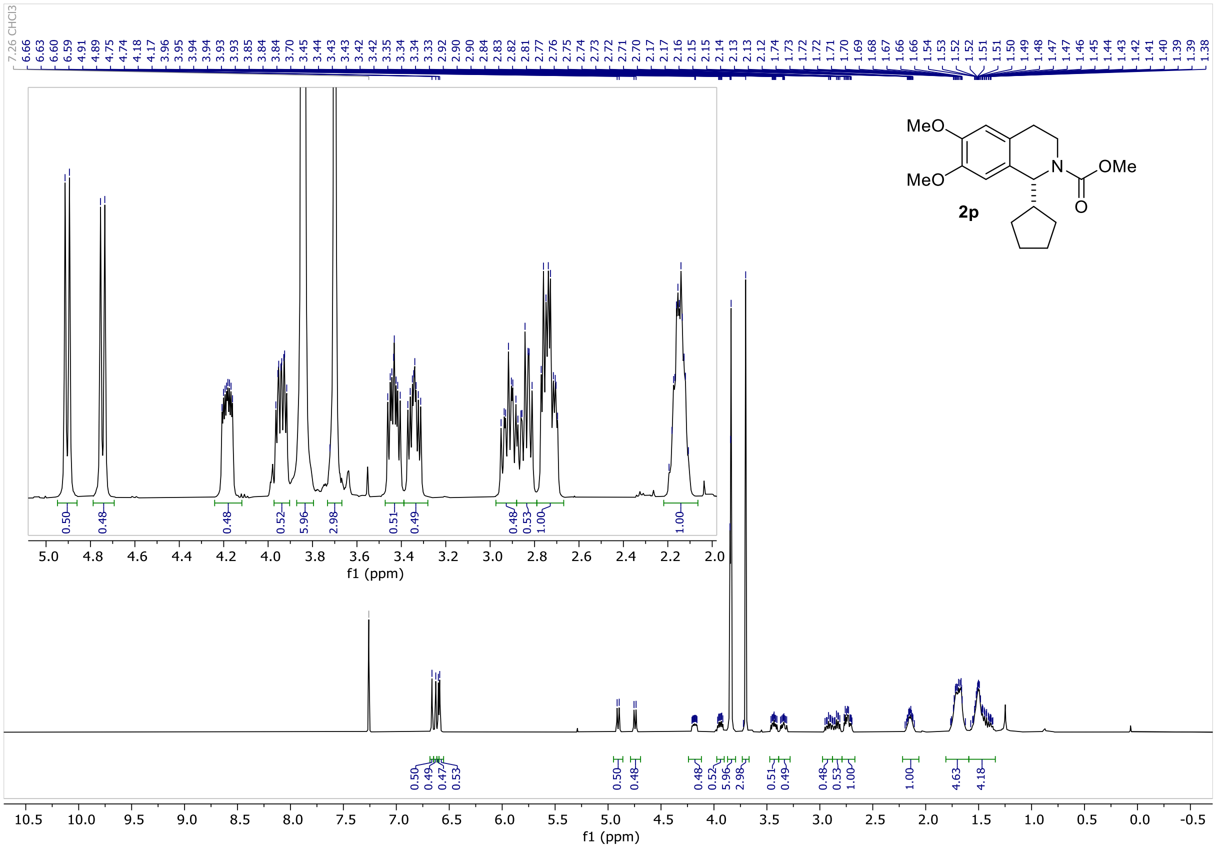

^{1}H-NMR spectrum of compound **2p**.

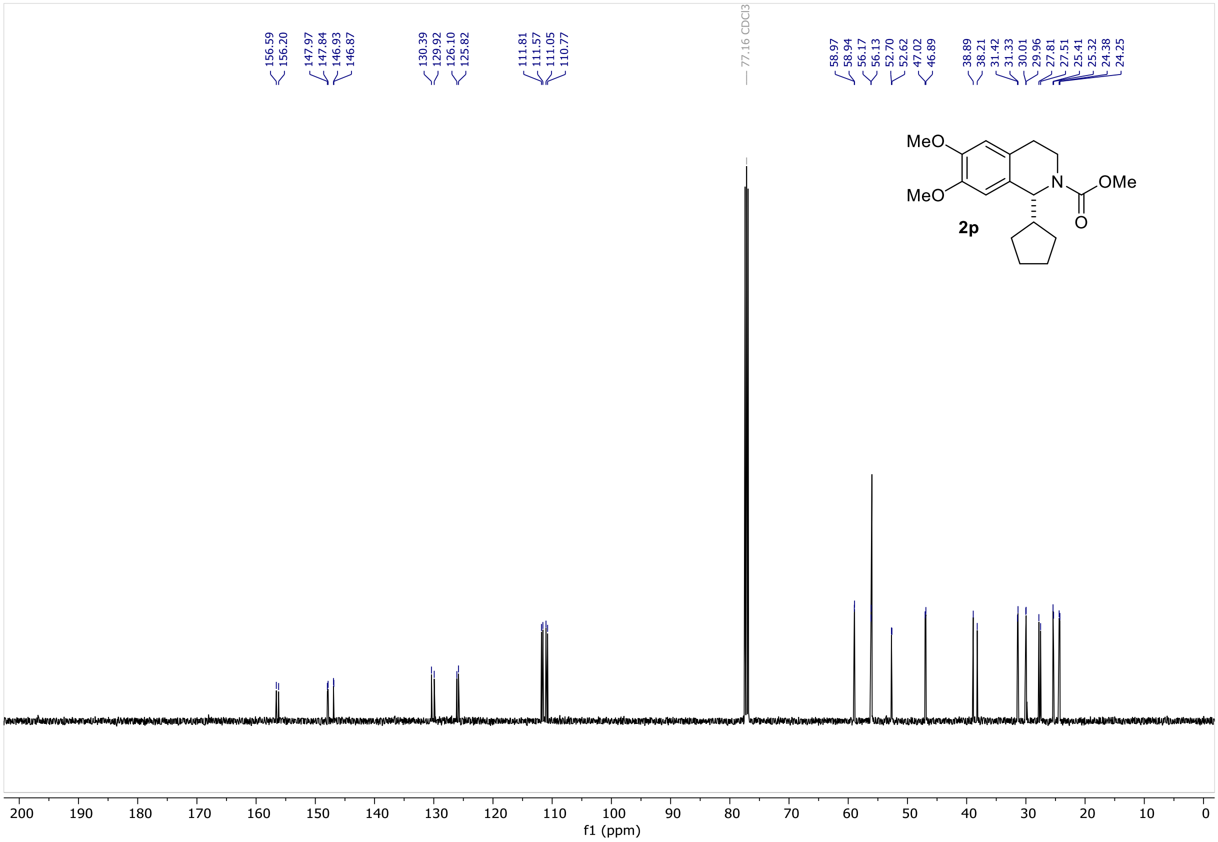

^{13}C-NMR spectrum of compound **2p**.

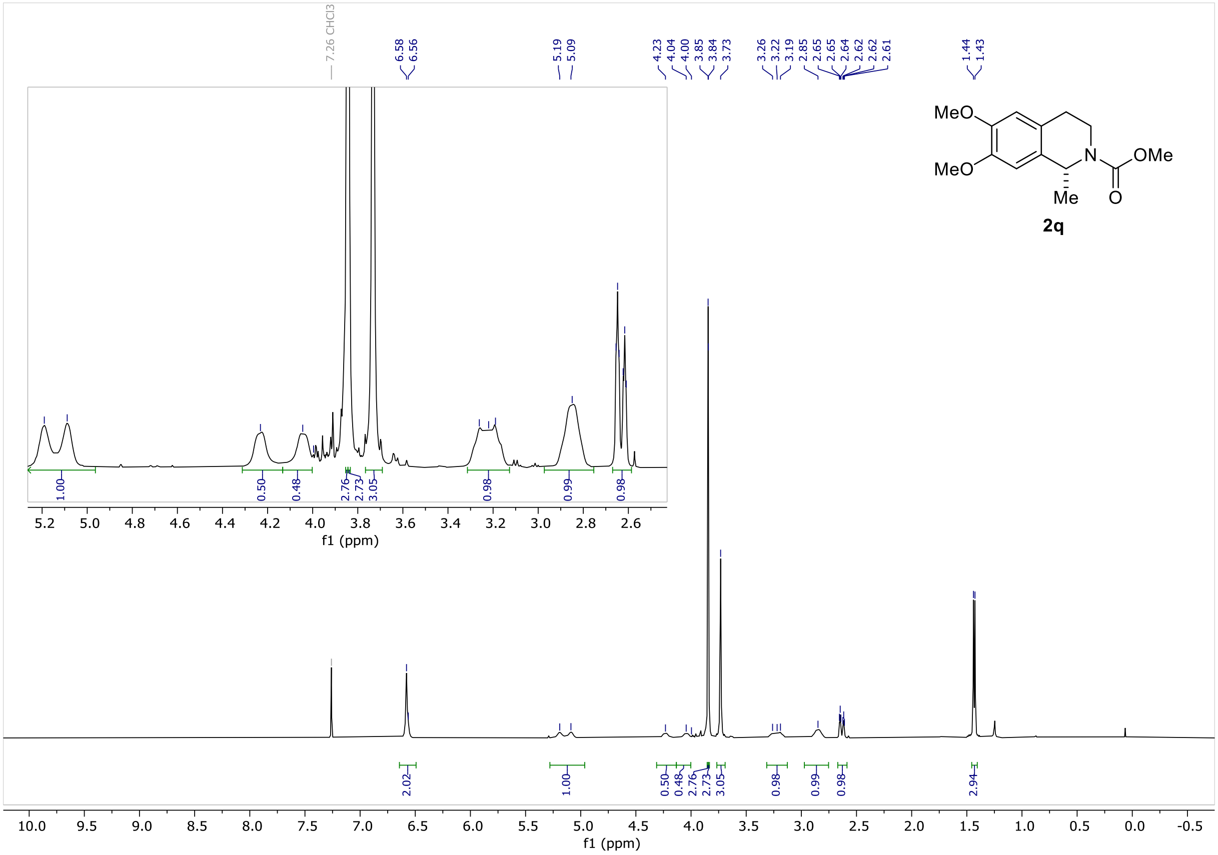

^{1}H-NMR spectrum of compound **2q**.

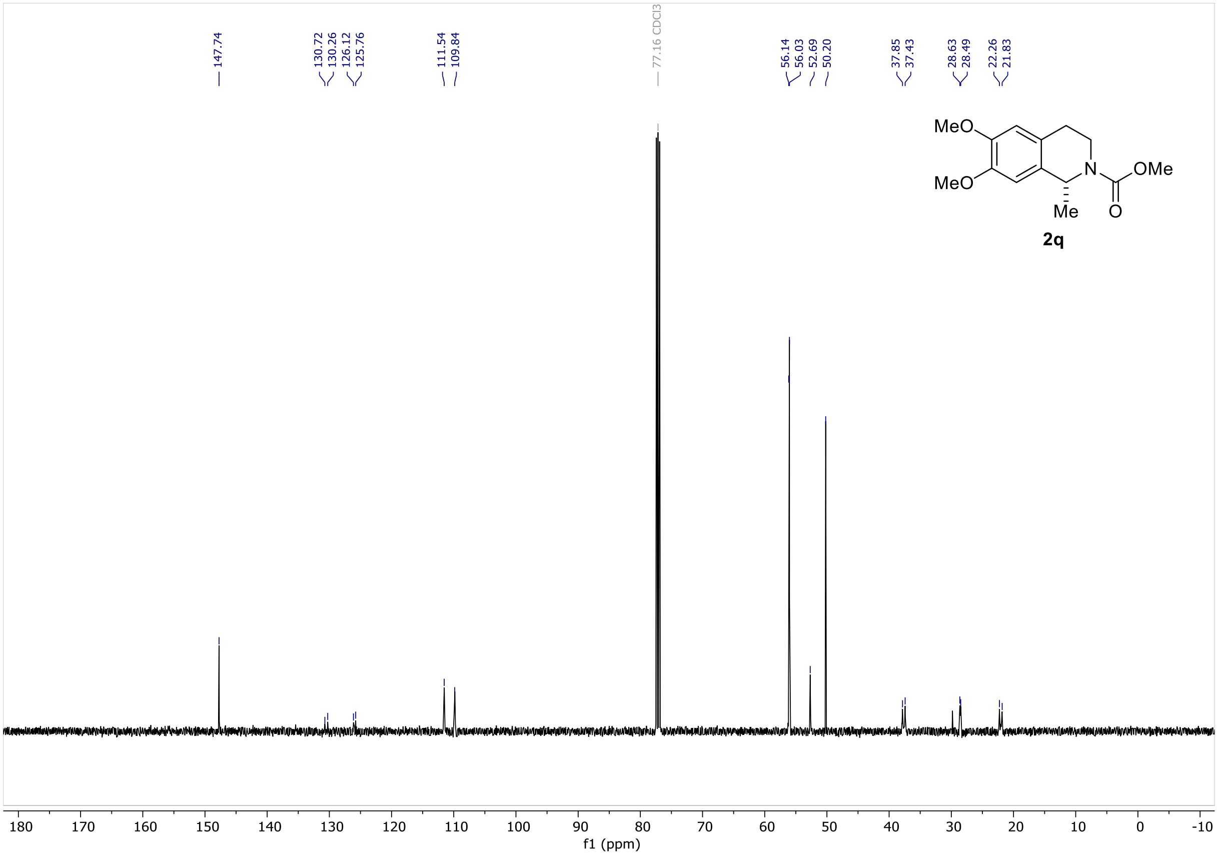

^{13}C-NMR spectrum of compound **2q**.

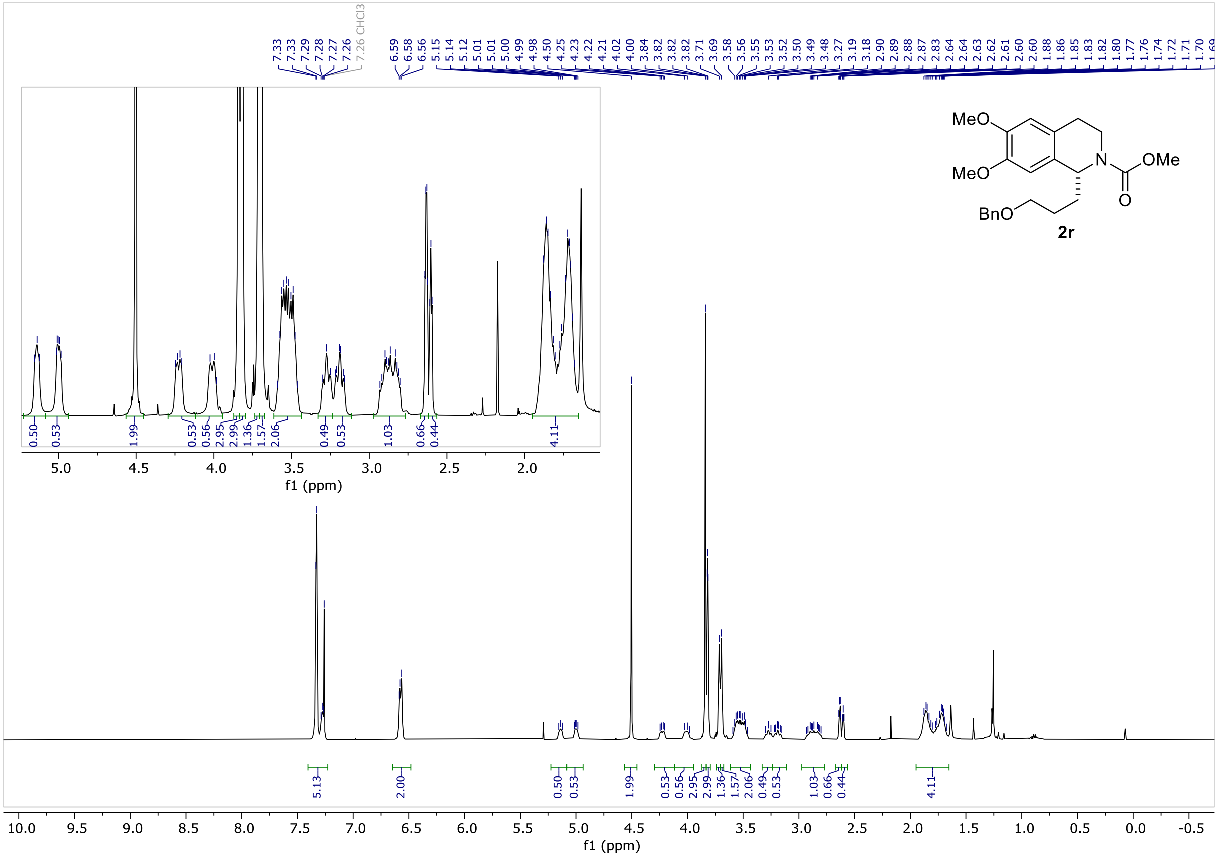

^{1}H-NMR spectrum of compound **2r**.

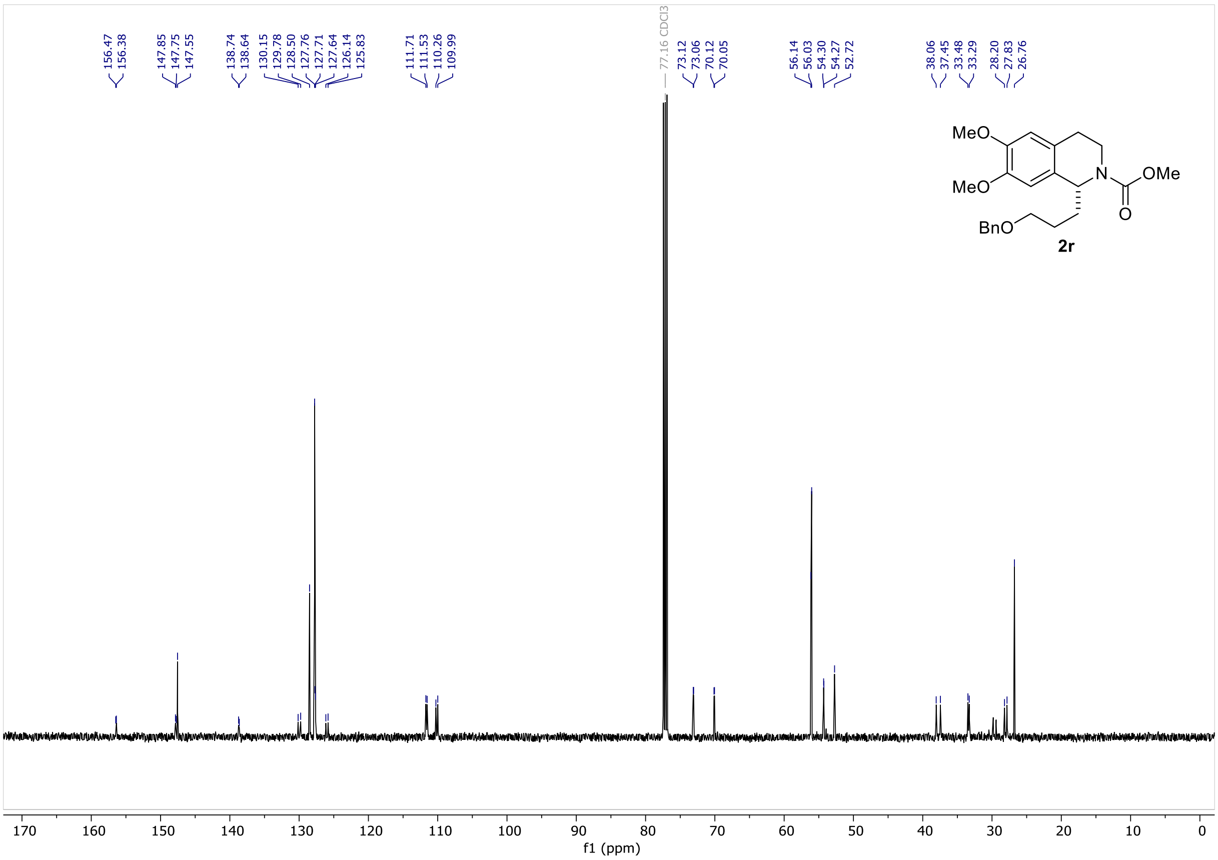

^{13}C-NMR spectrum of compound **2r**.

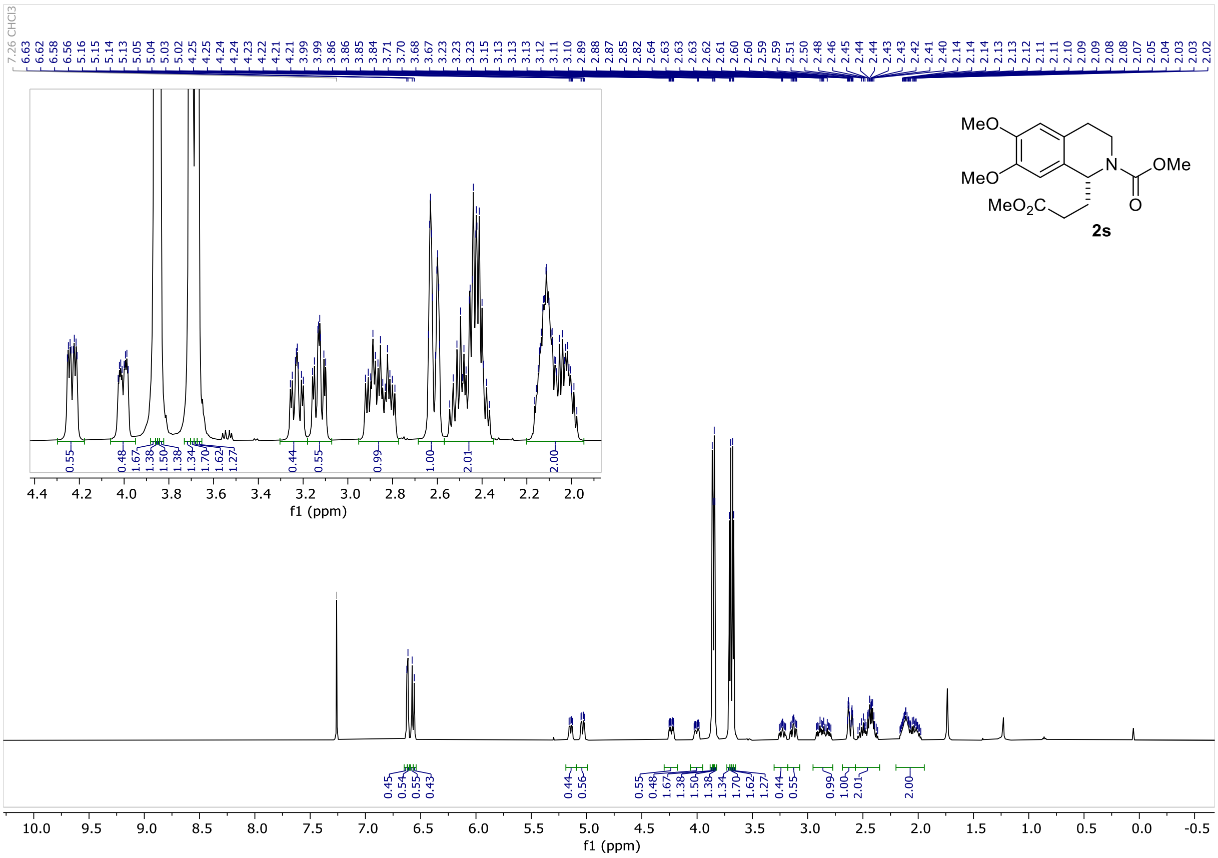

^{1}H-NMR spectrum of compound **2s**.

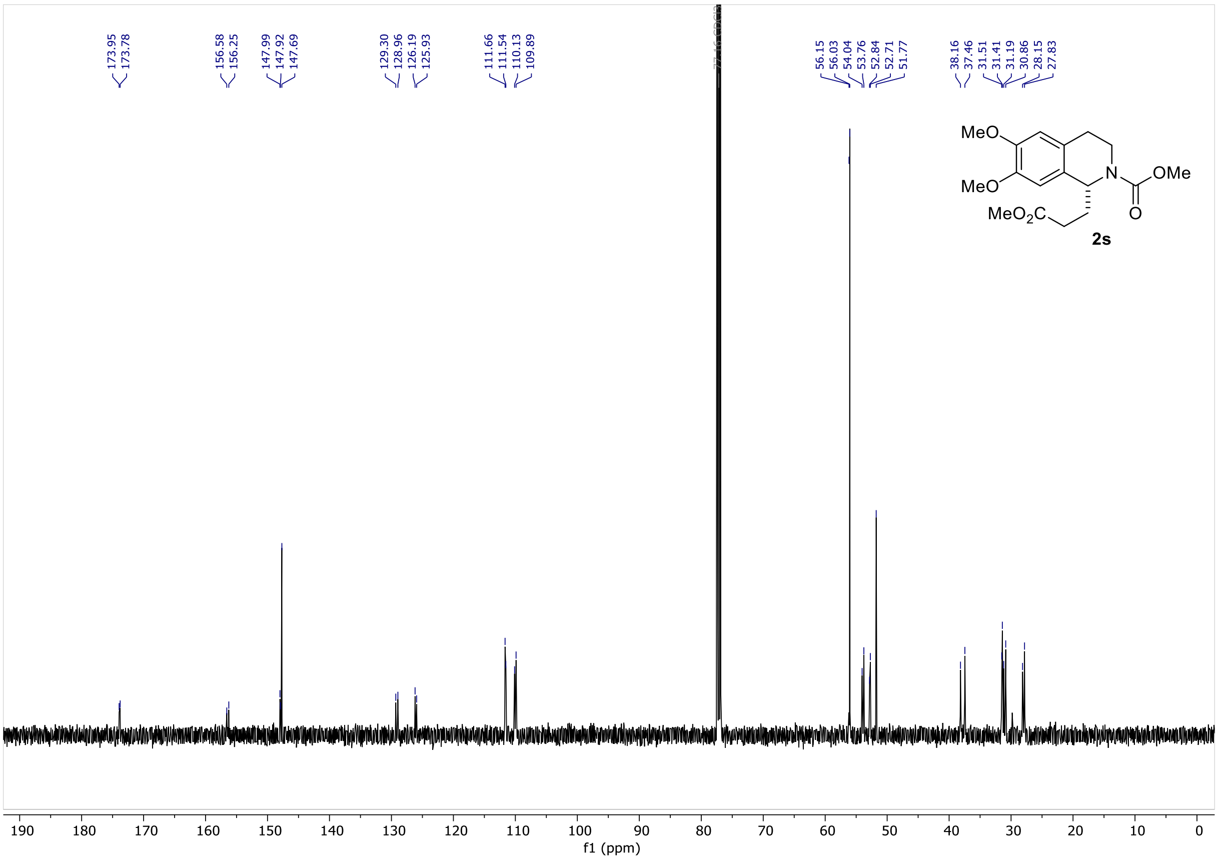

^{13}C-NMR spectrum of compound **2s**.

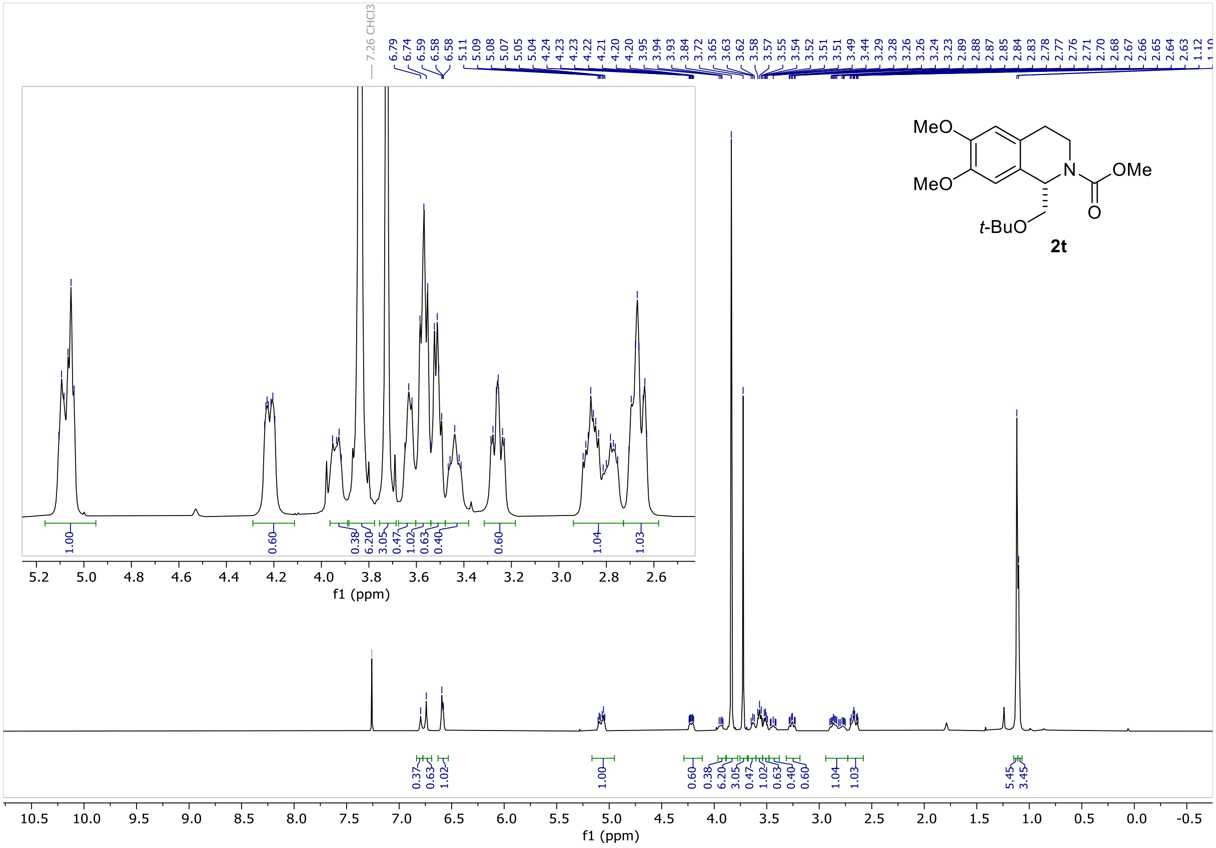

^{1}H-NMR spectrum of compound **2t**.

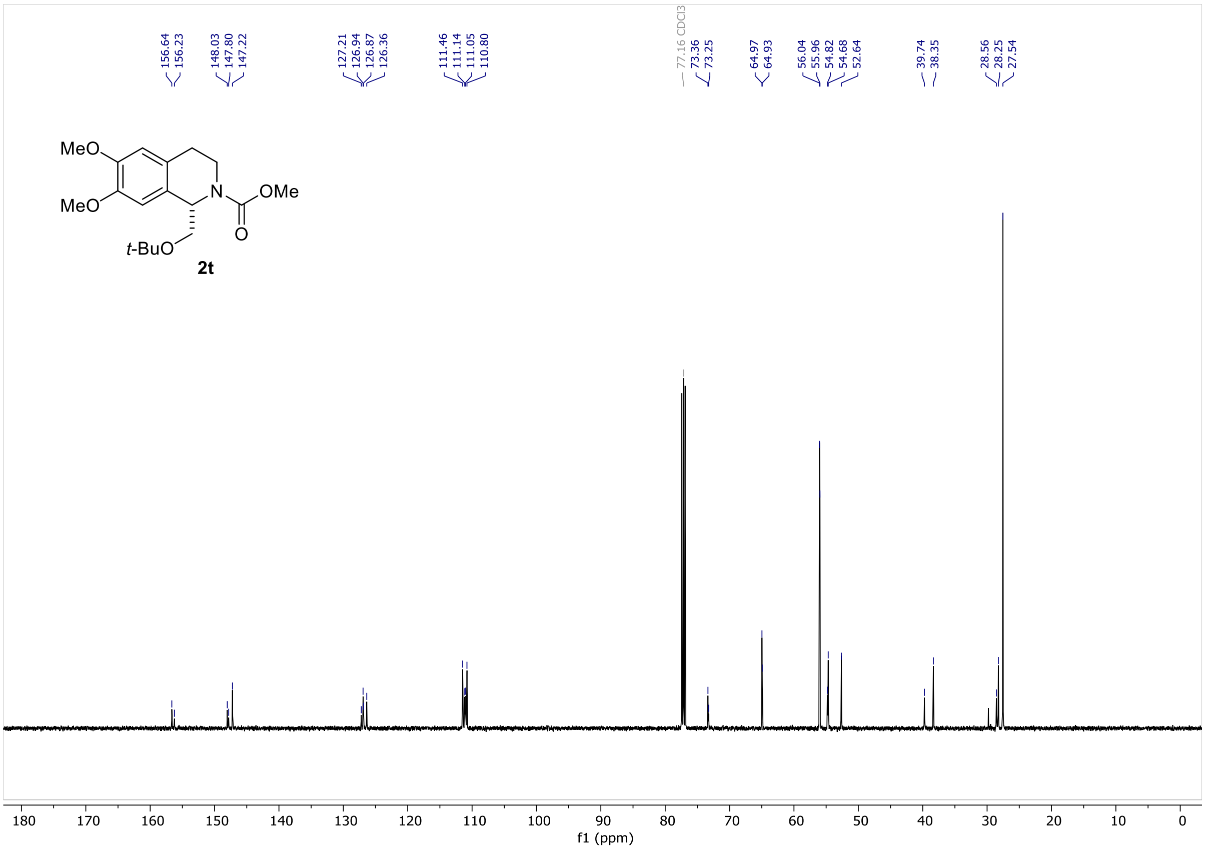

^{13}C-NMR spectrum of compound **2t**.

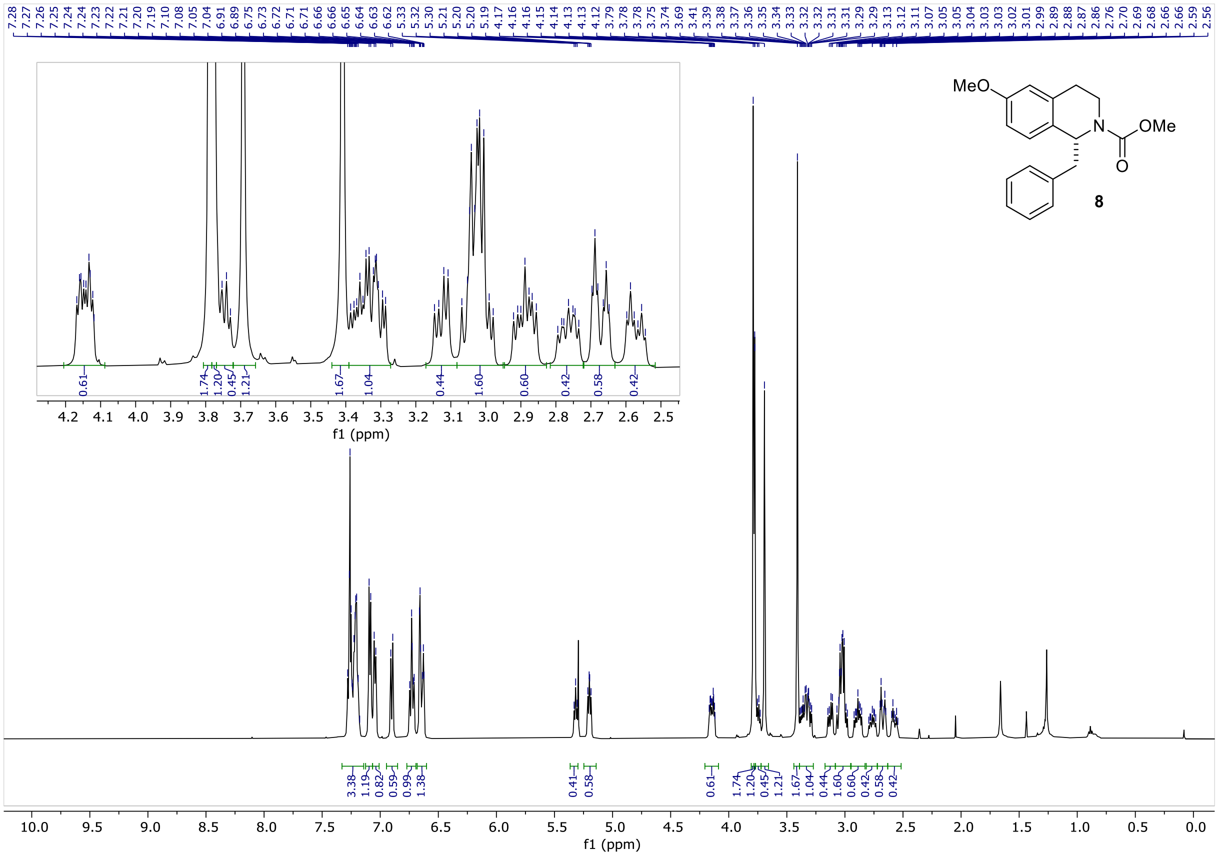

[1]**H-NMR** spectrum of compound **8**.

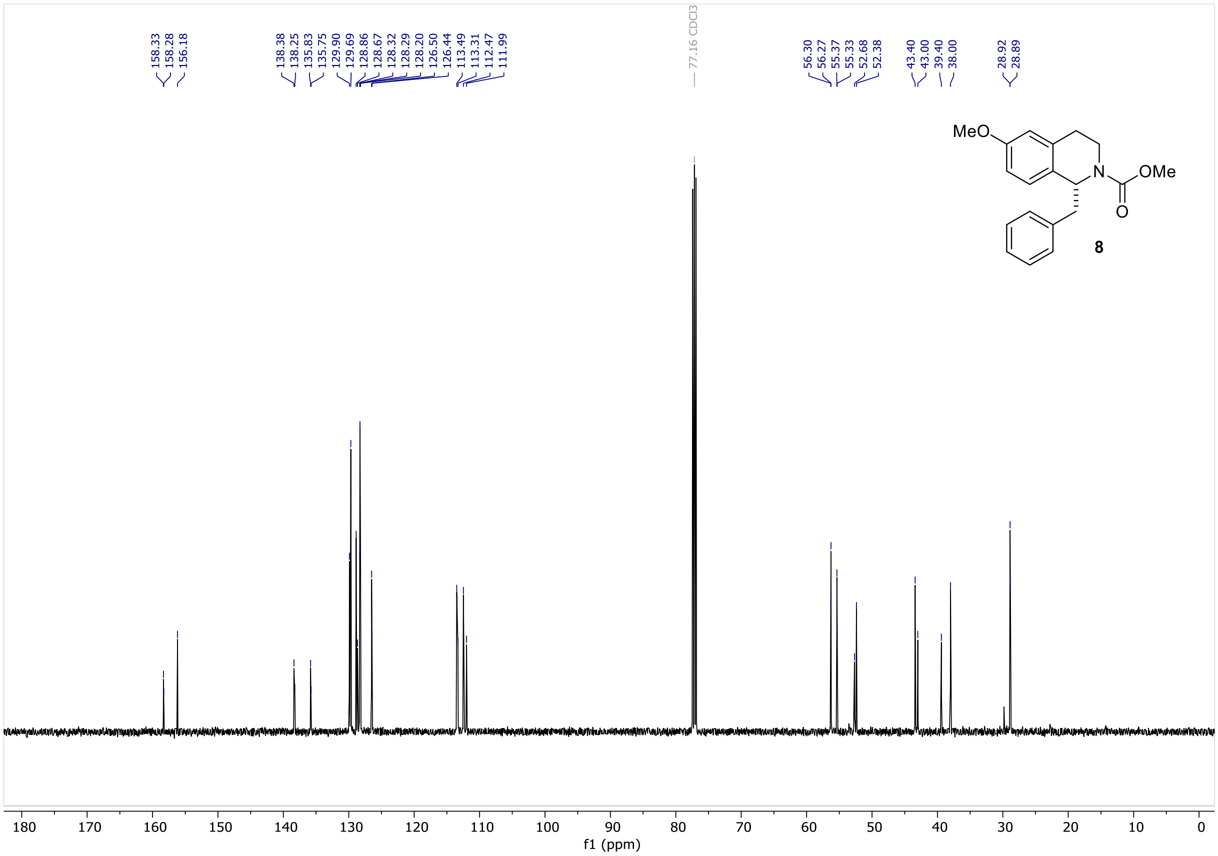

[13]**C-NMR** spectrum of compound **8**.

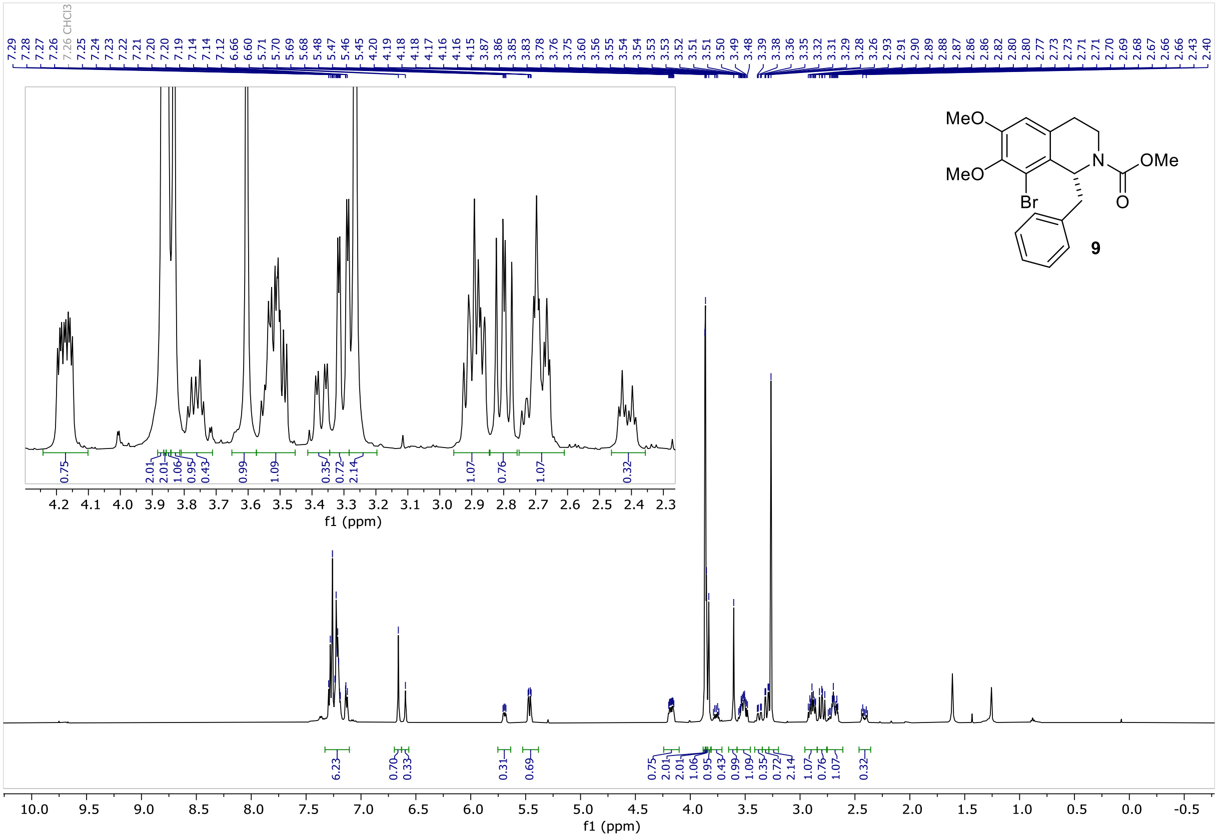

^{1}H-NMR spectrum of compound **9**.

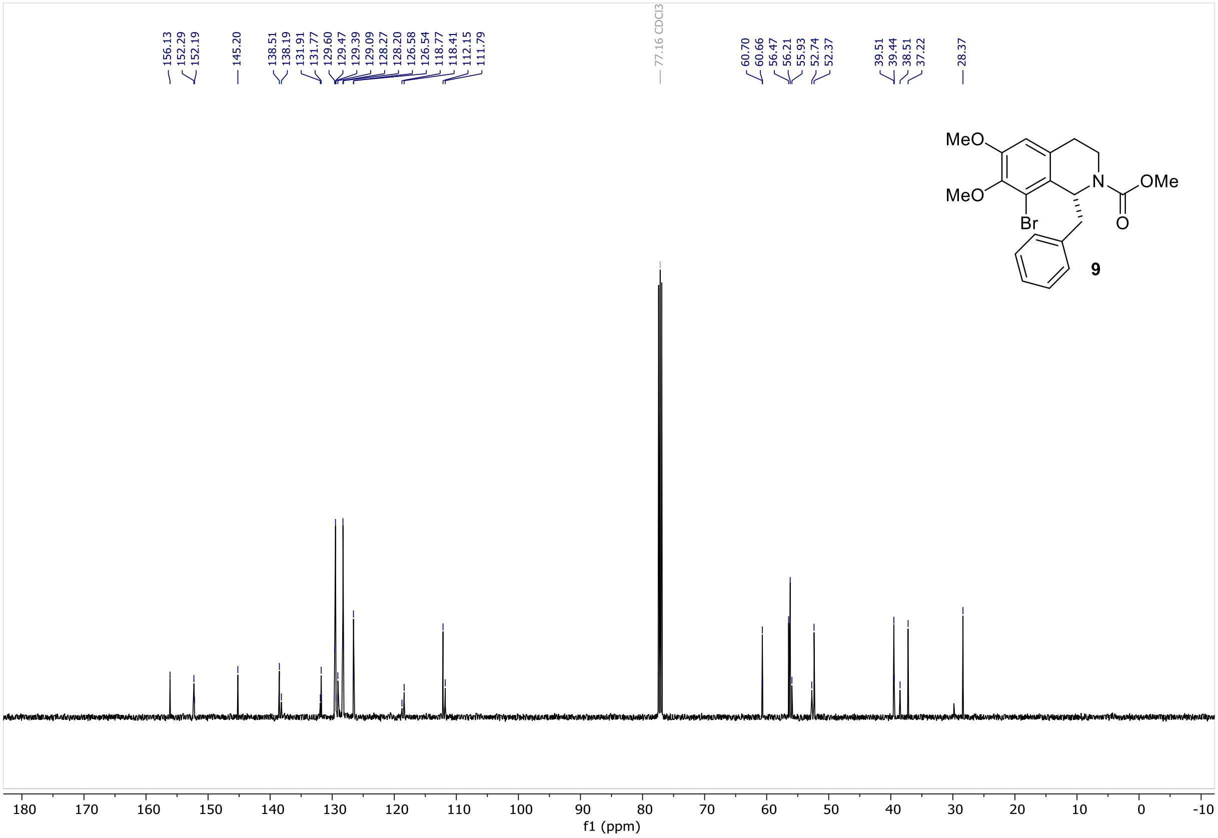

[12]C-NMR spectrum of compound **9**.

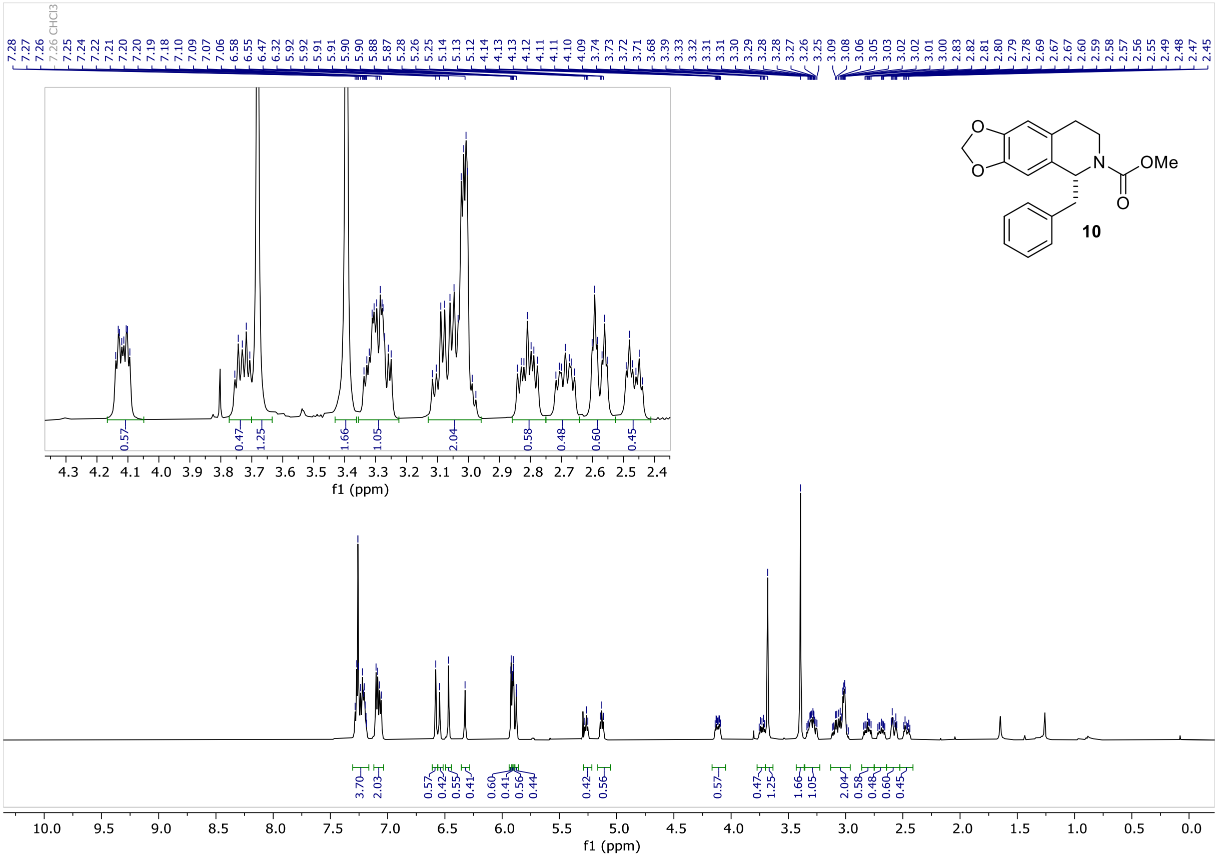

^{1}**H-NMR** spectrum of compound **10**.

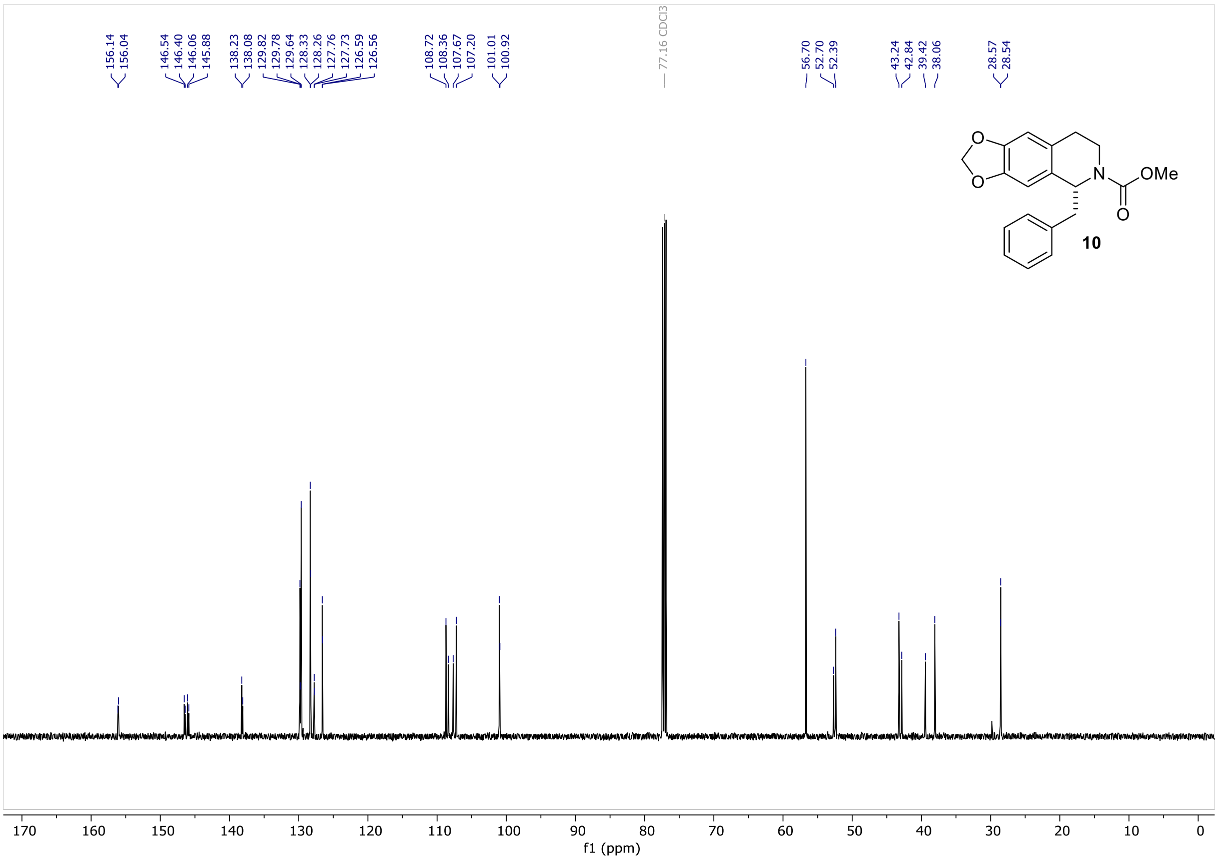

^{12}**C-NMR** spectrum of compound **10**.

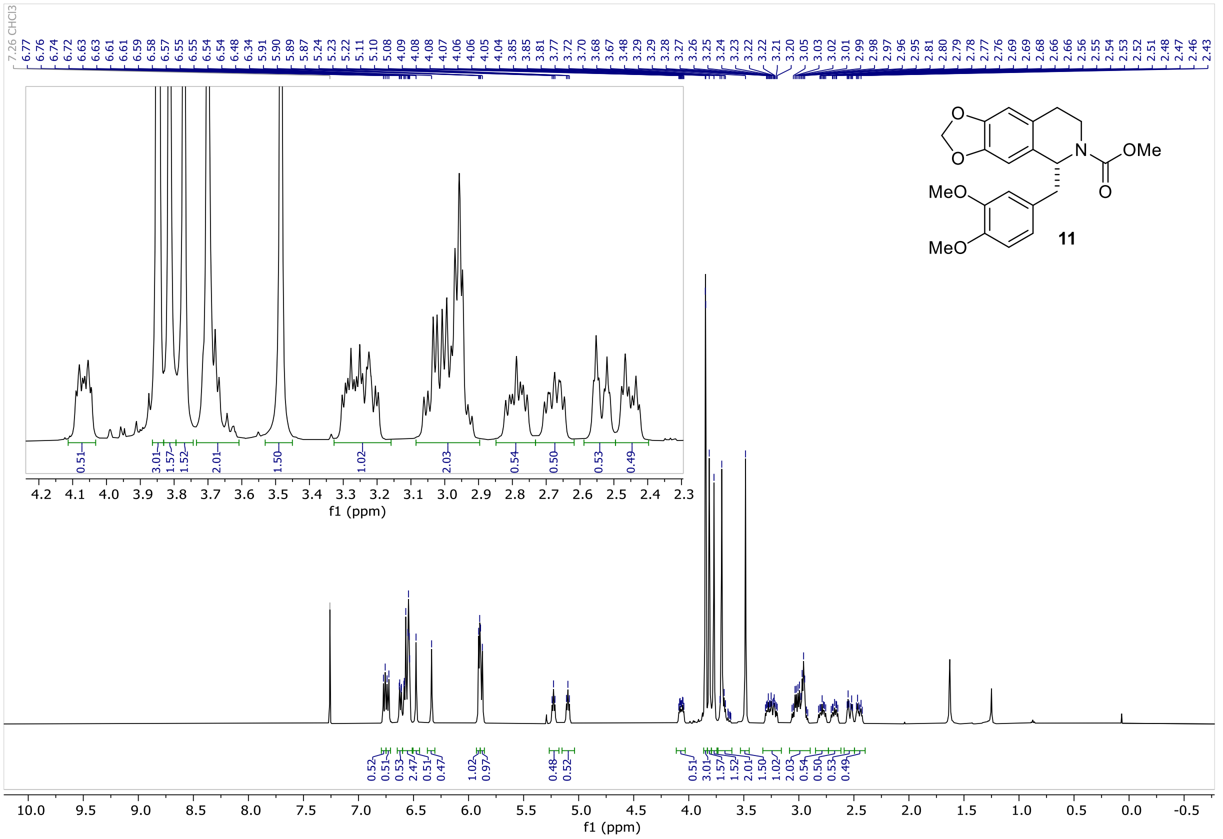

^{1}H-NMR spectrum of compound **11**.

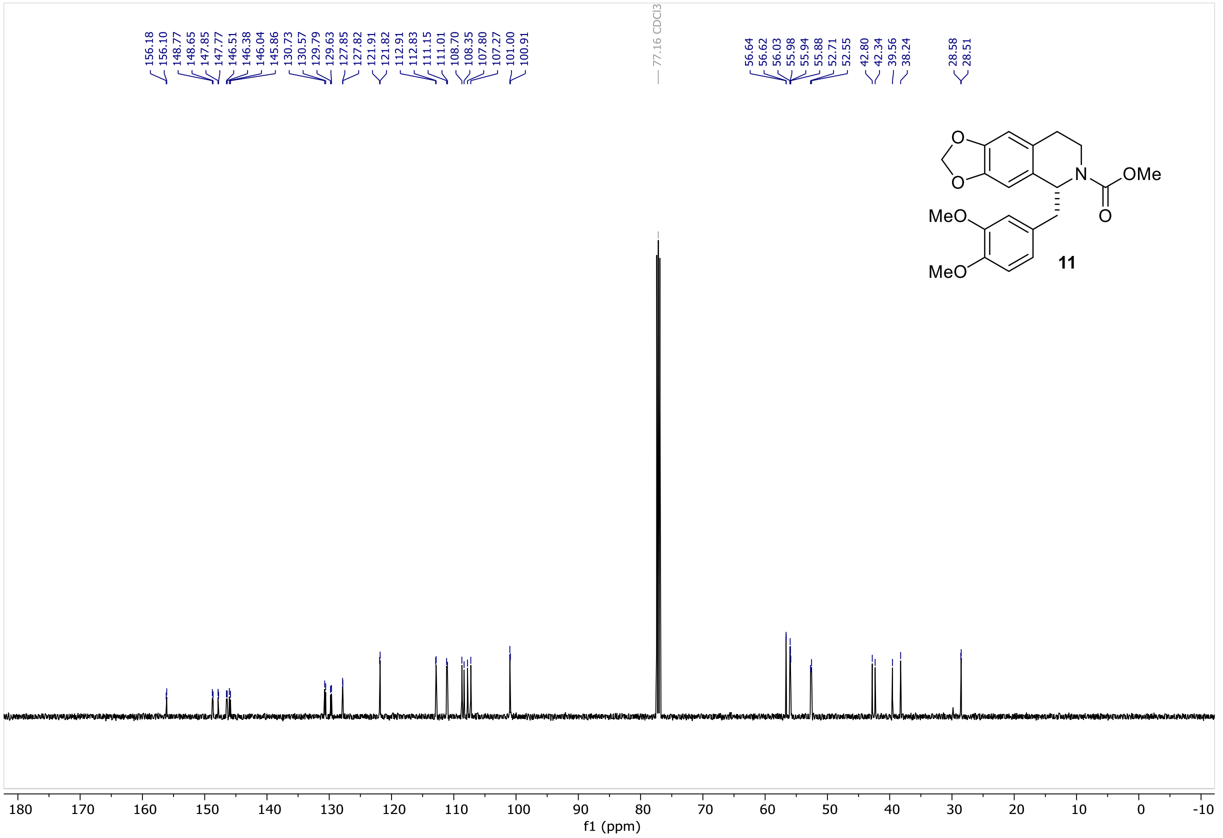

^{13}C-NMR spectrum of compound **11.**

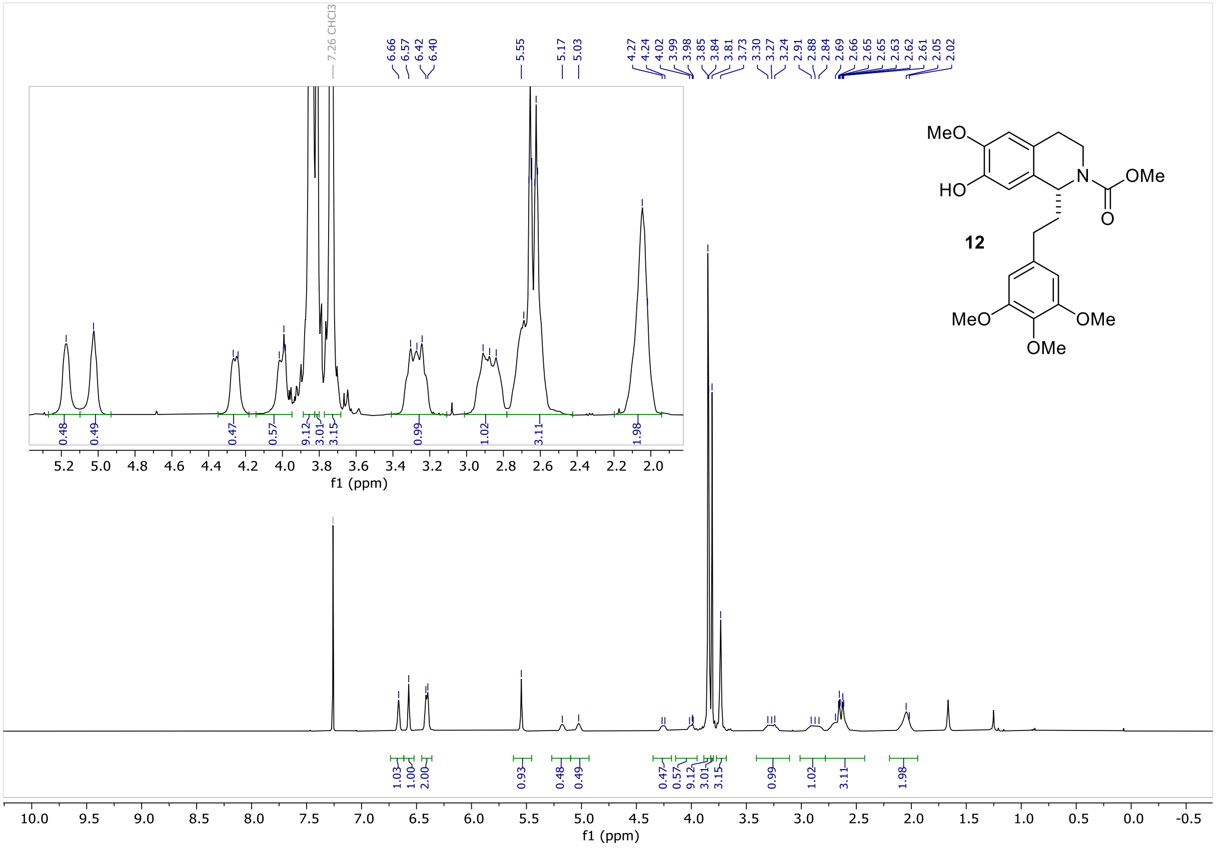

^{1}H-NMR spectrum of compound **12.**

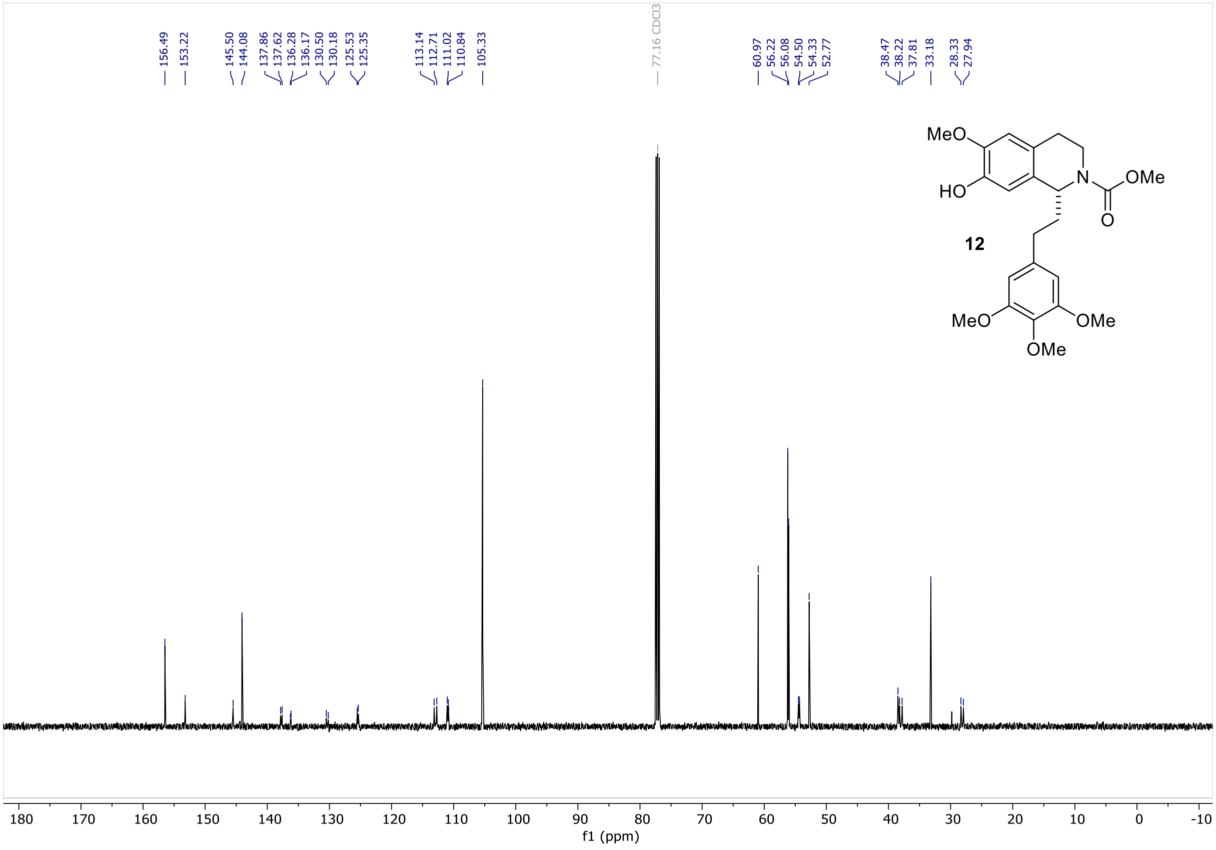

^{13}C-NMR spectrum of compound **12.**

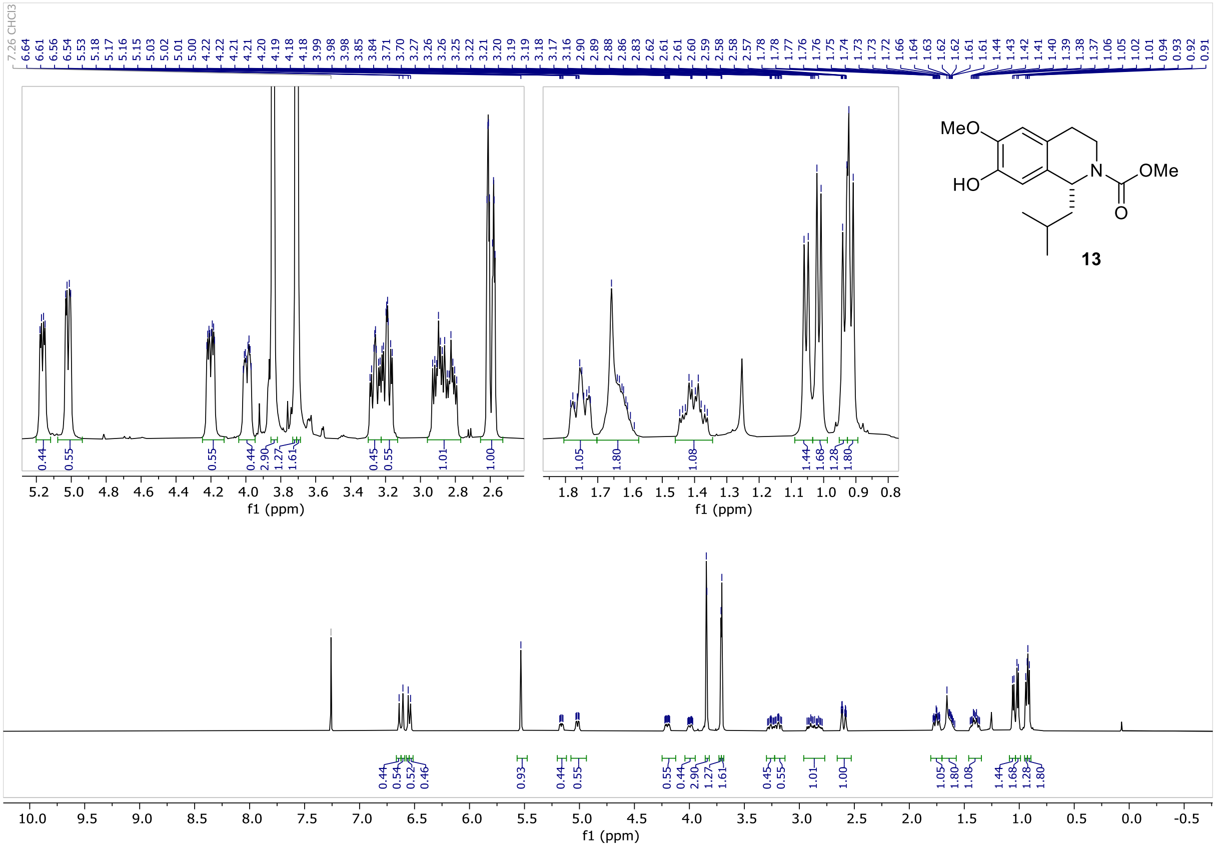

^{1}H-NMR spectrum of compound **13.**

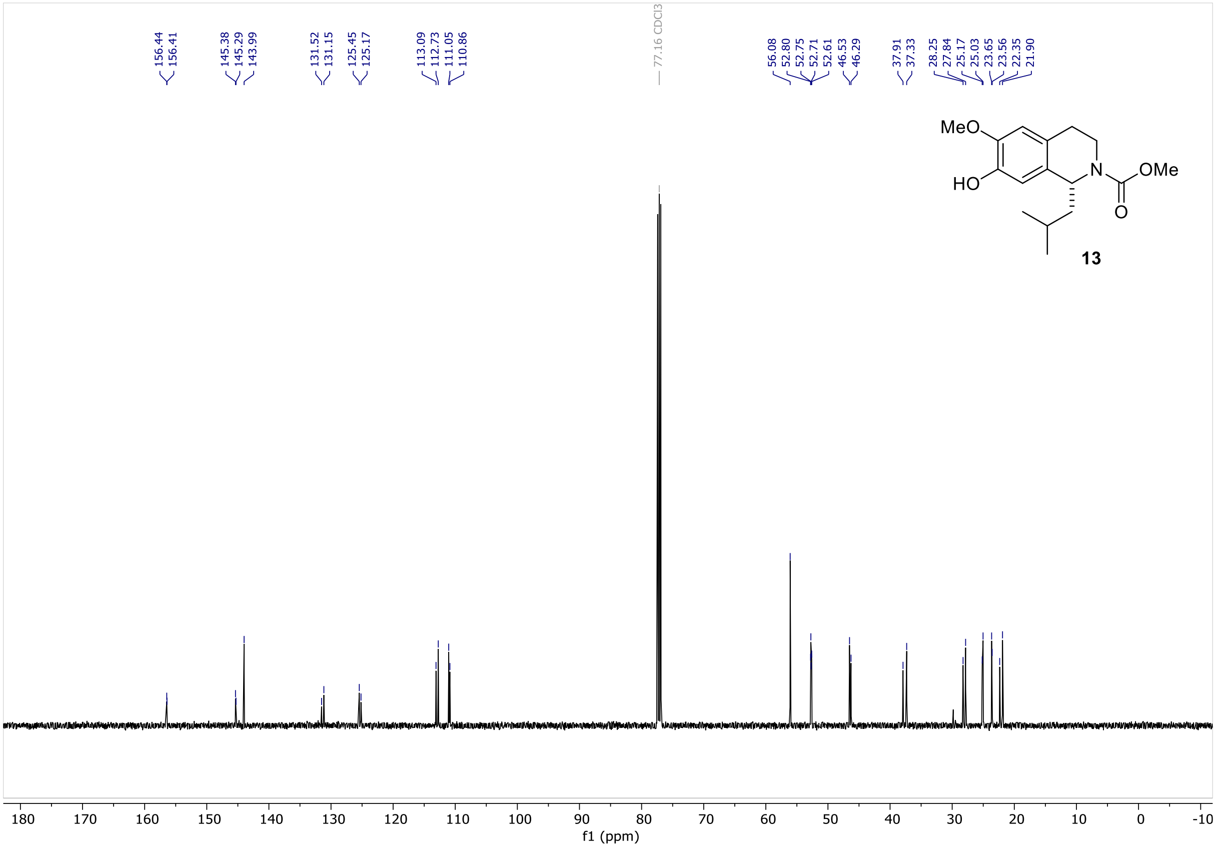

^{13}C-NMR spectrum of compound **13.**

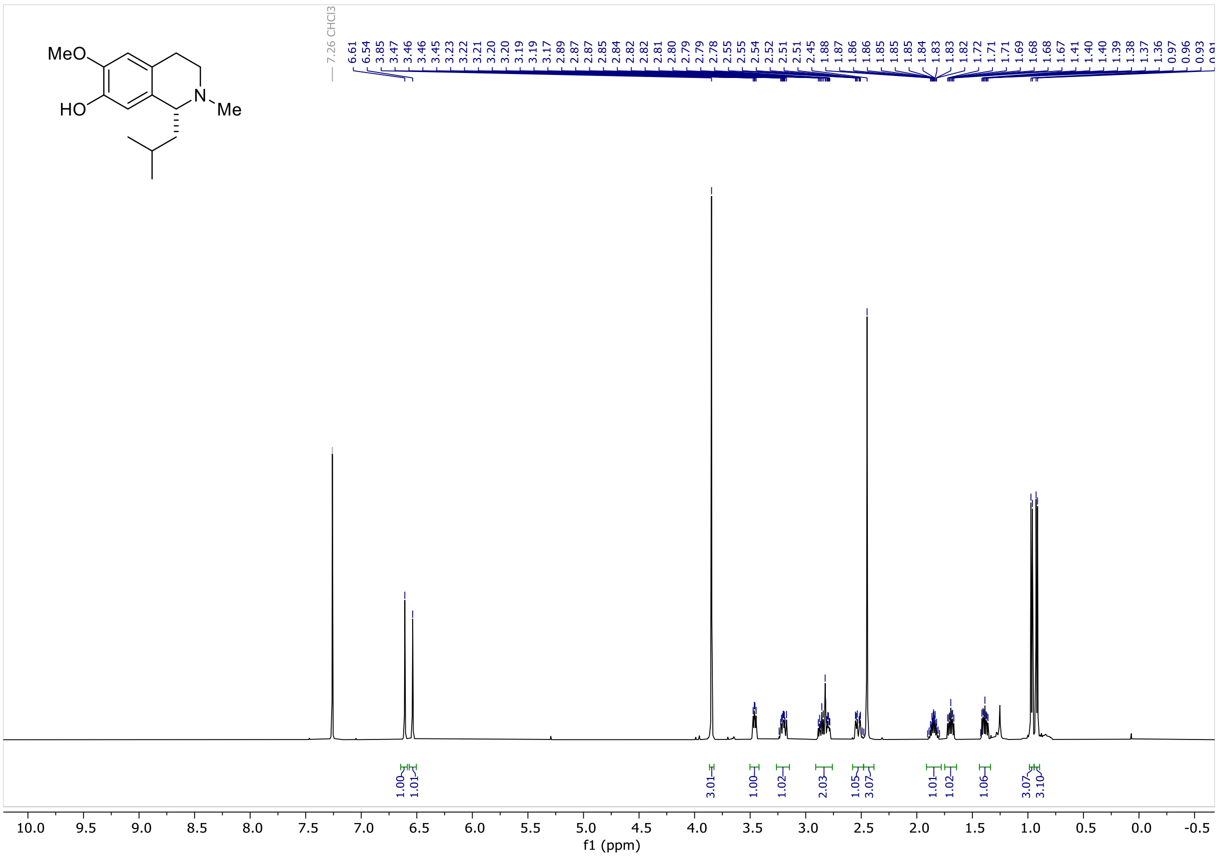

^{1}H-NMR spectrum of Lophocerine**.**

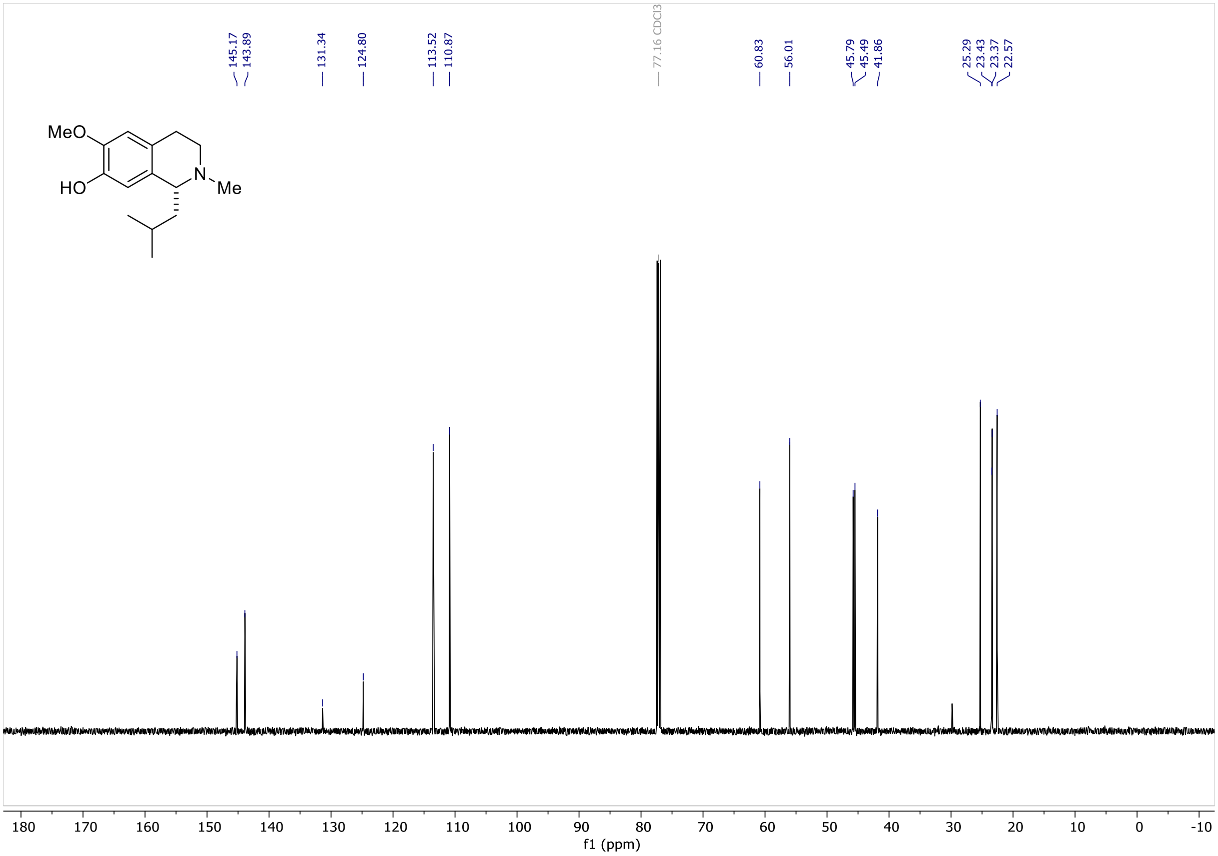

^{13}C-NMR spectrum of Lophocerine**.**

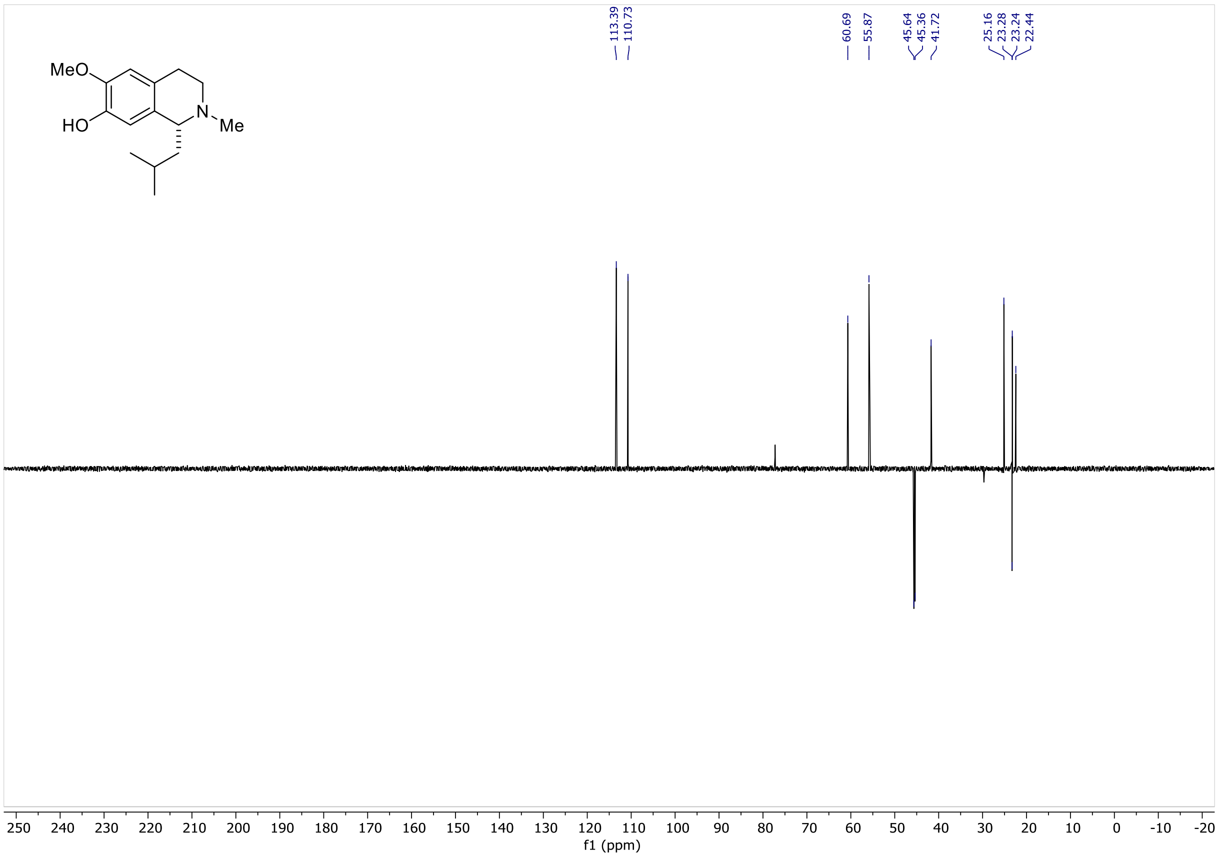

DEPT 135 NMR spectrum of Lophocerine.

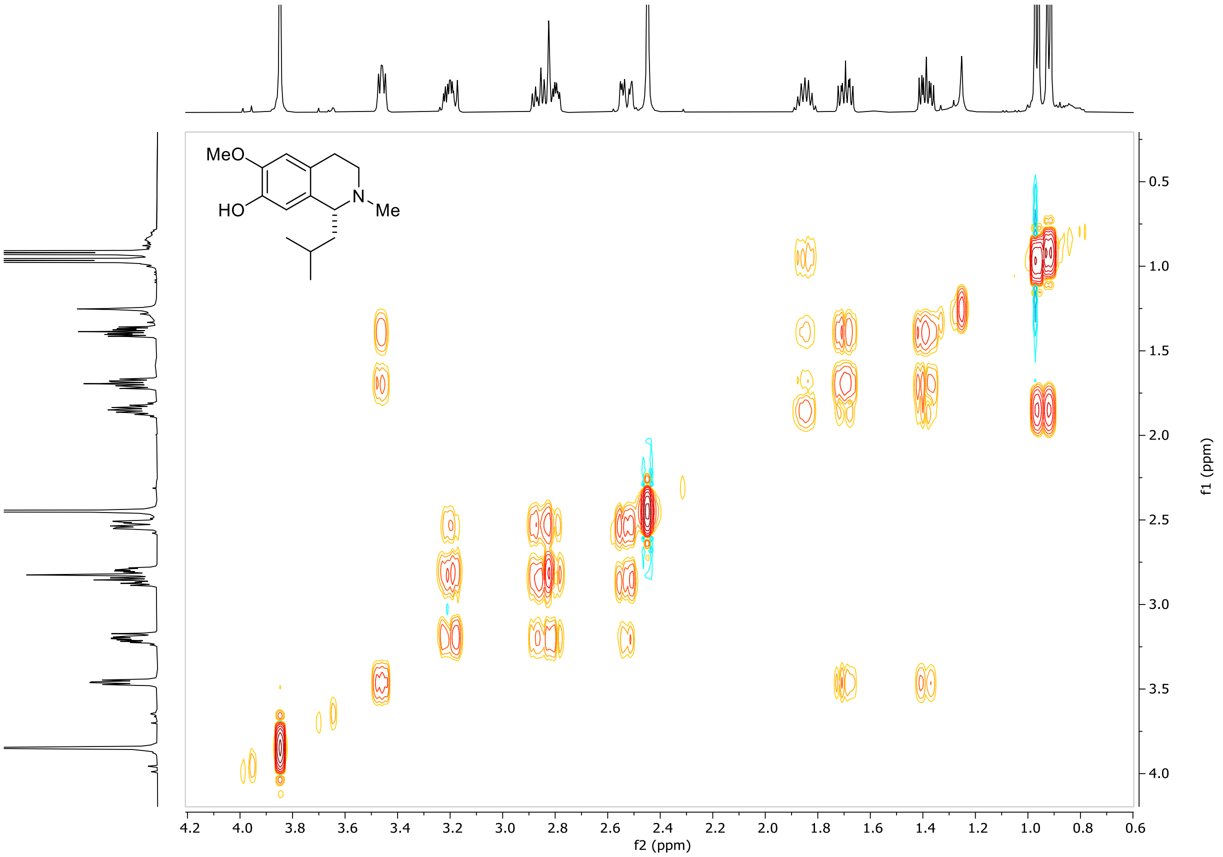

^{1}H-COSY NMR spectrum of Lophocerine.

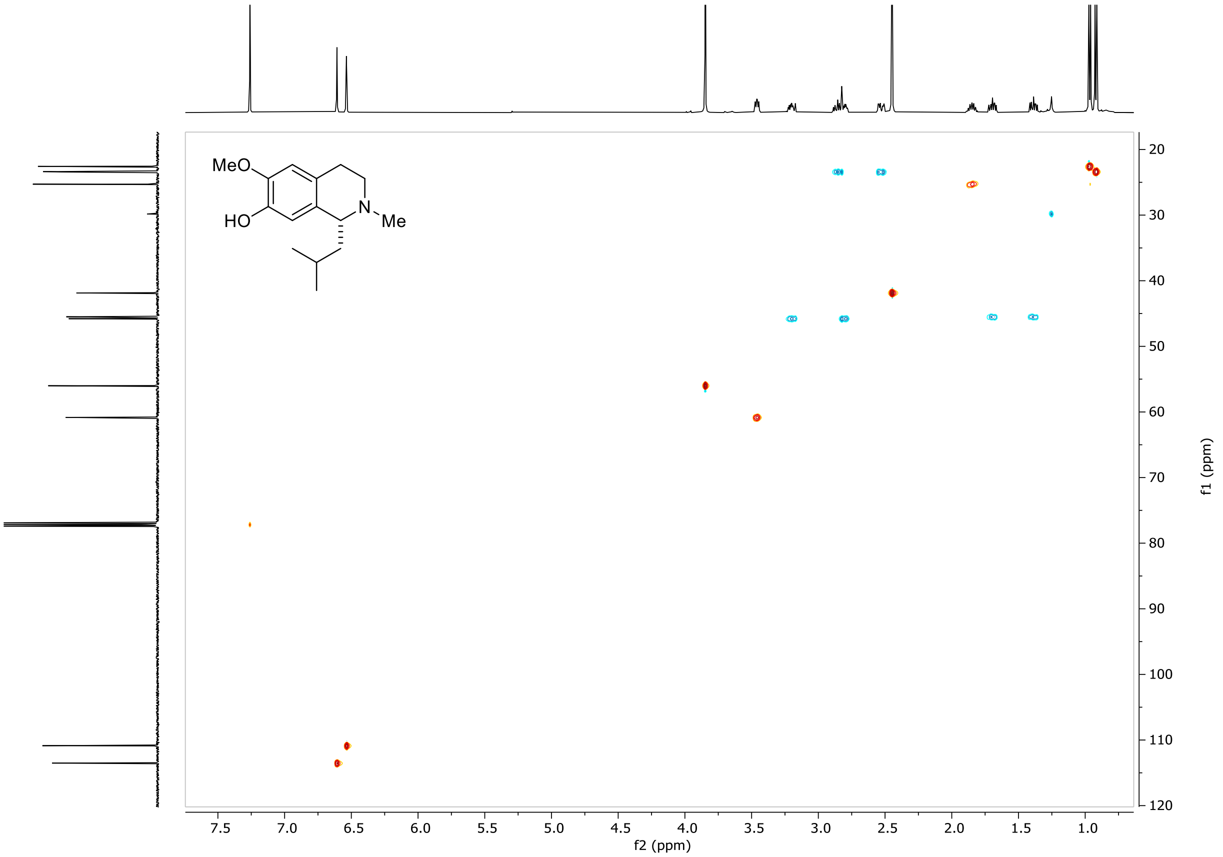

^{1}H-^{13}C-HSQC NMR spectrum of Lophocerine.

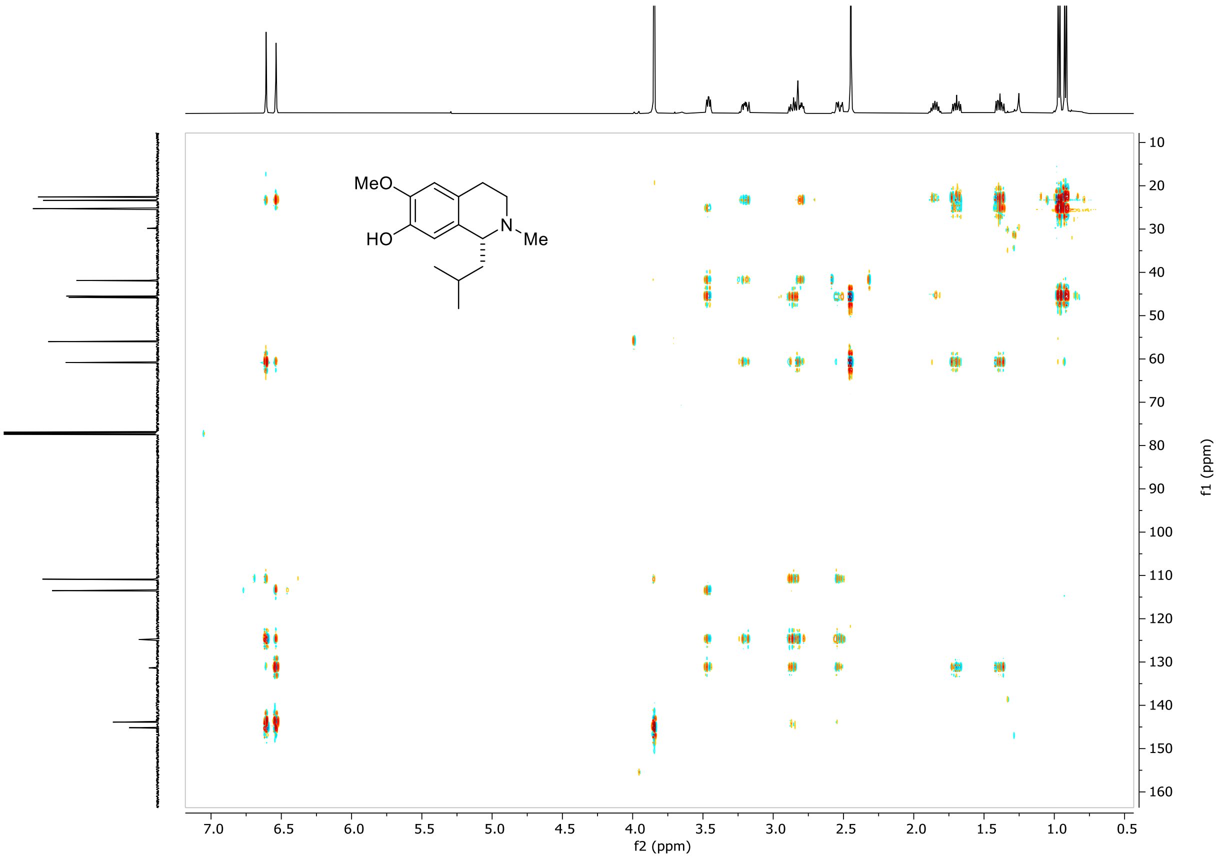

^{1}H-^{13}C-HMBC NMR spectrum of Lophocerine.

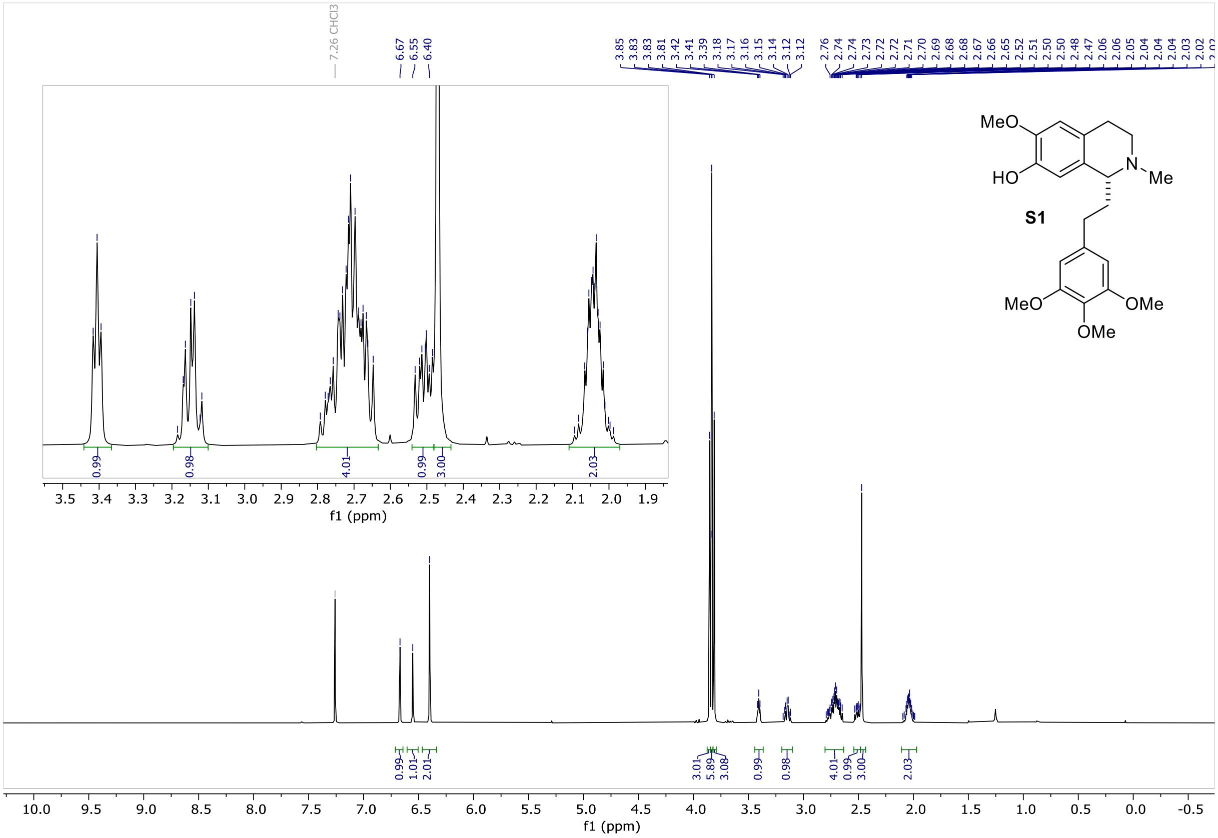

^{1}H-NMR spectrum of compound **S1.**

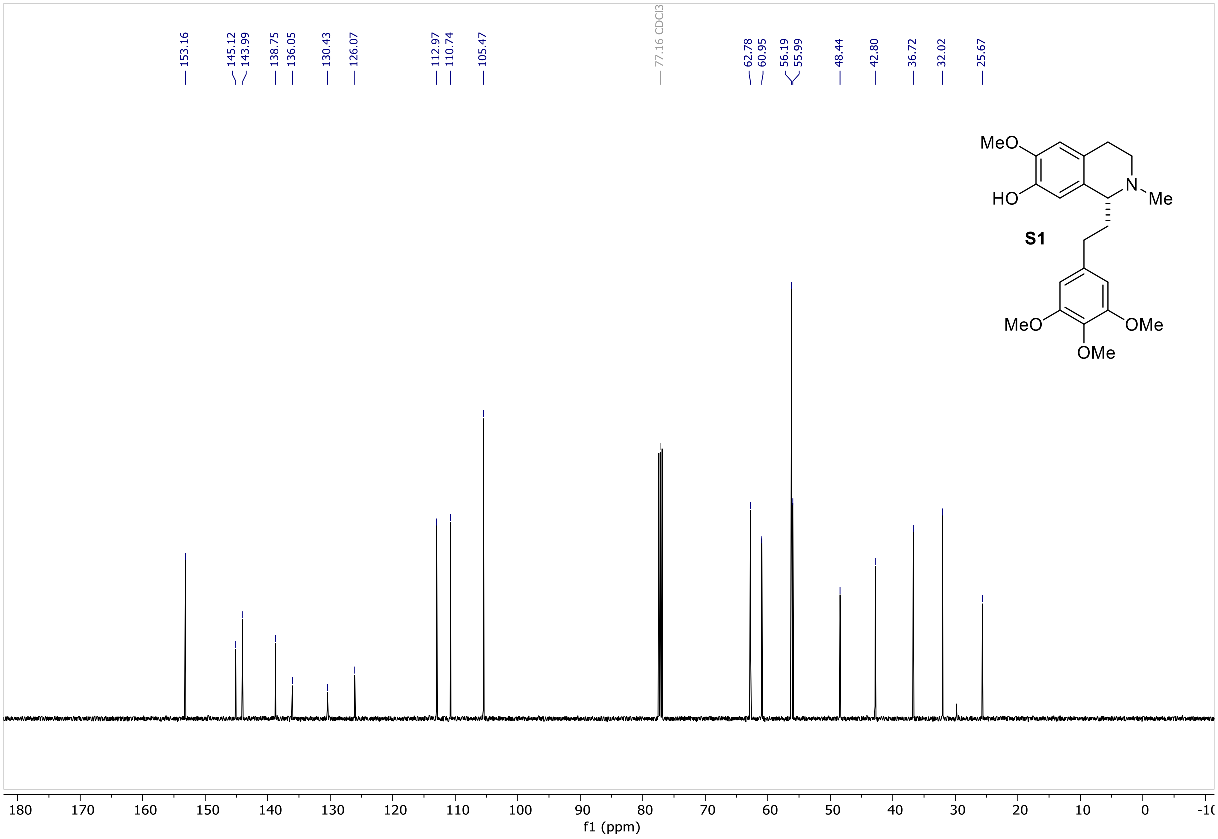

^{13}C-NMR spectrum of compound **S1.**

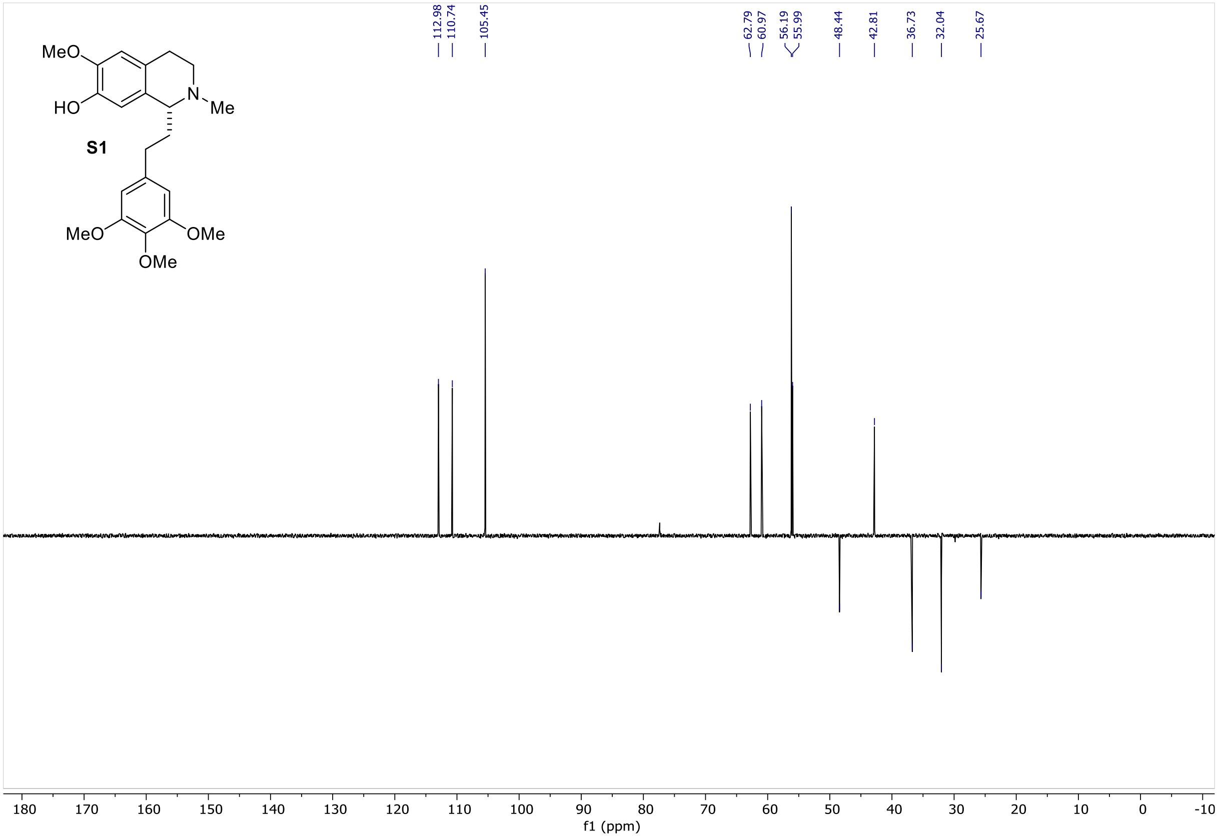

DEPT135-NMR spectrum of compound **S1.**

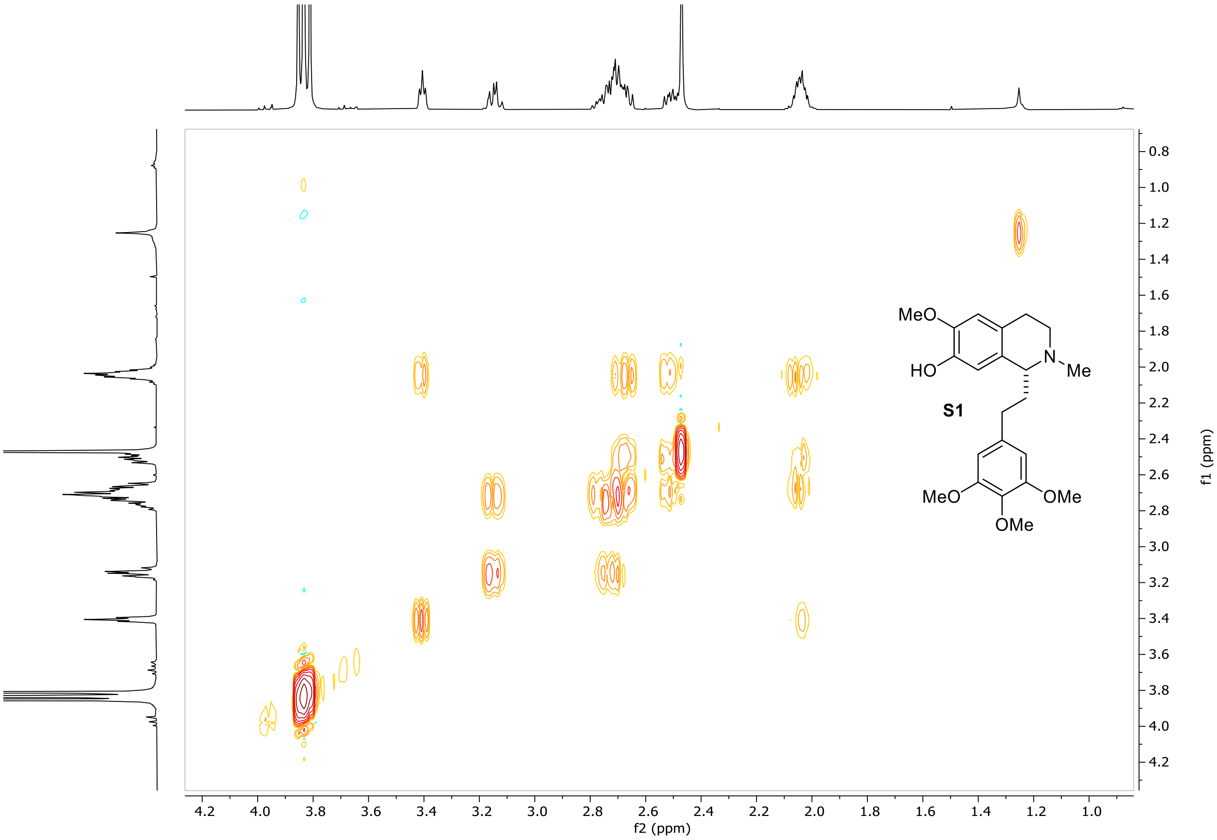

^{1}H-COSY NMR spectrum of compound **S1.**

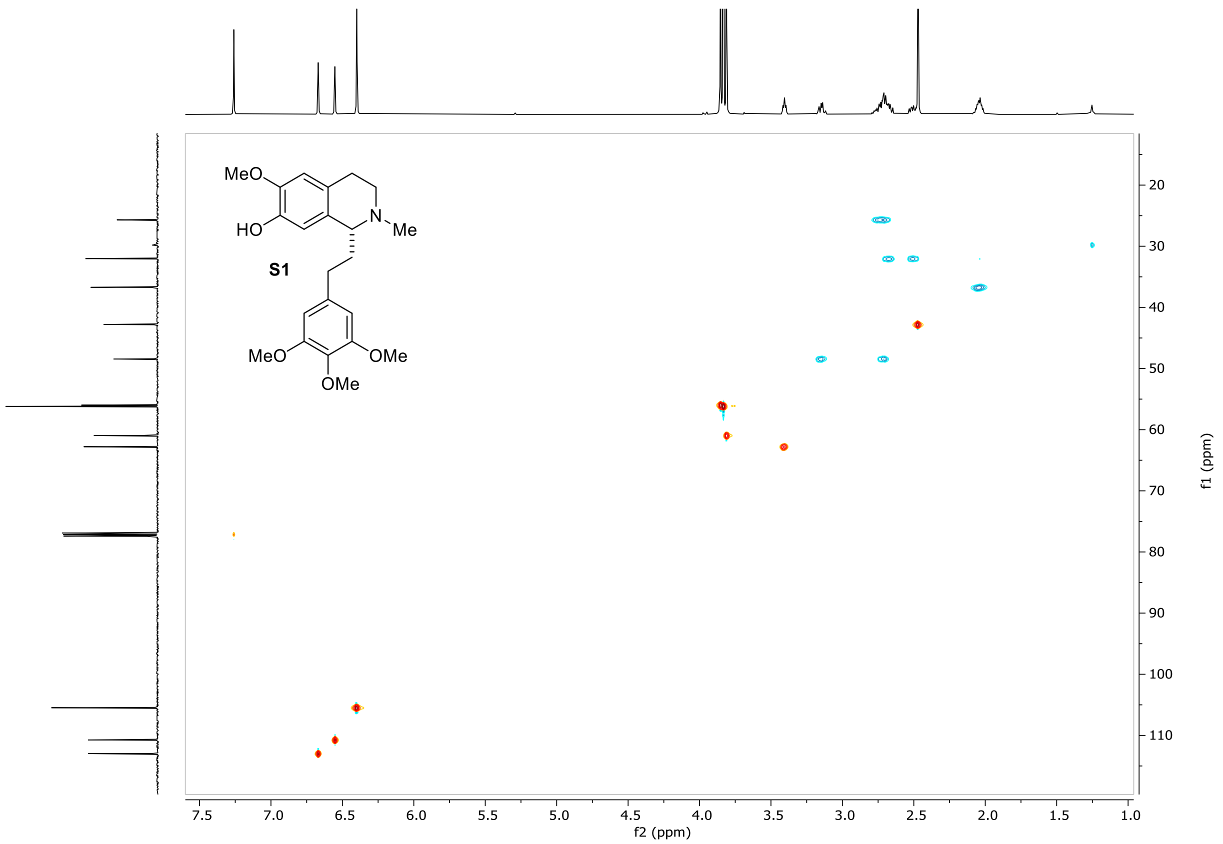

^{1}H-^{13}C-HSQC NMR spectrum of compound **S1.**

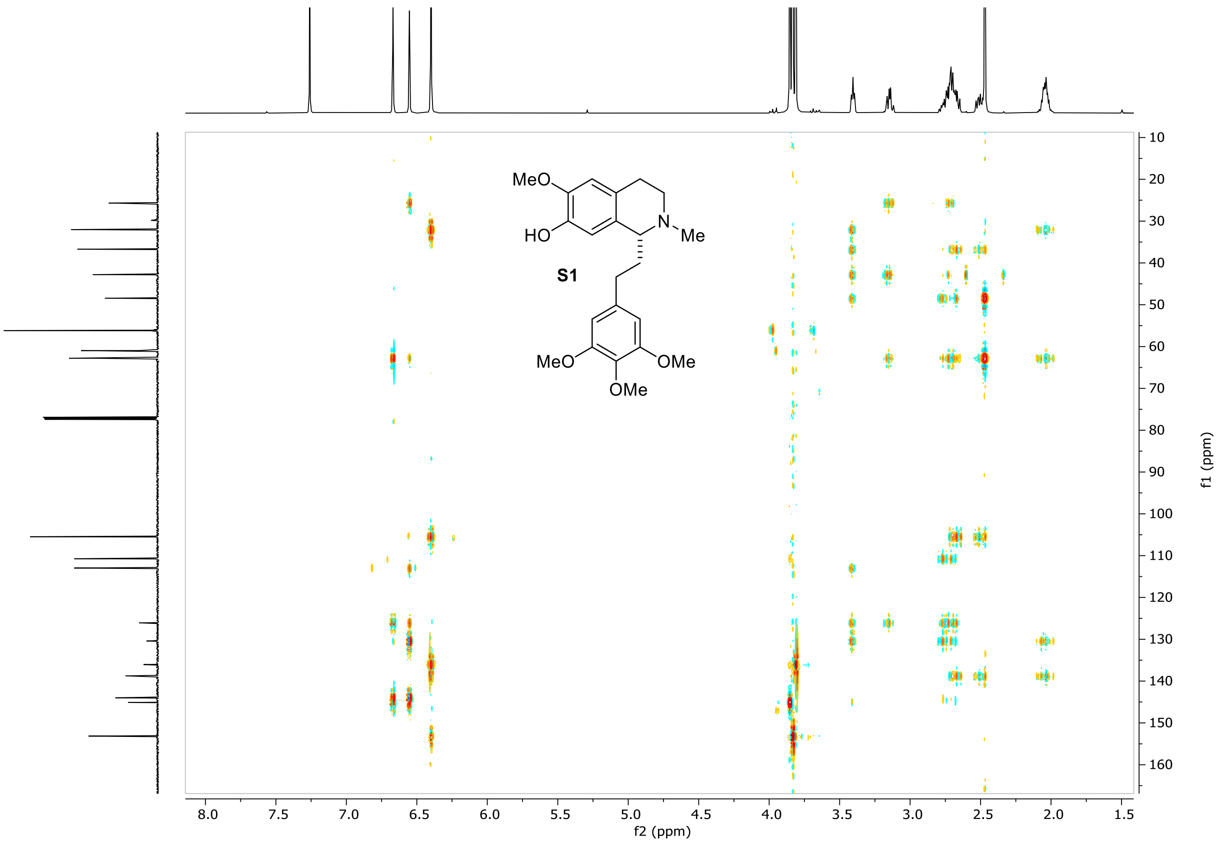

^{1}H-^{13}C-HMBC NMR spectrum of compound **S1.**

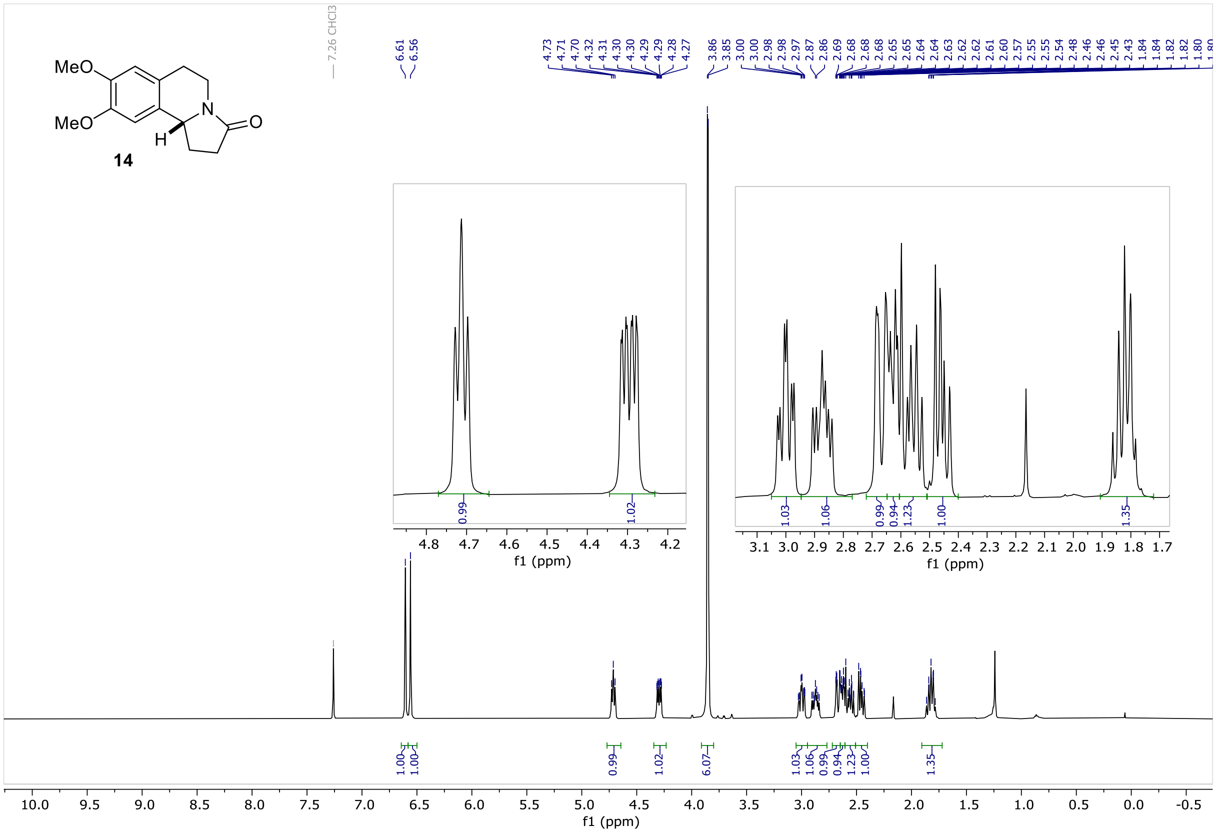

[1]**H-NMR** spectrum of compound **14**.

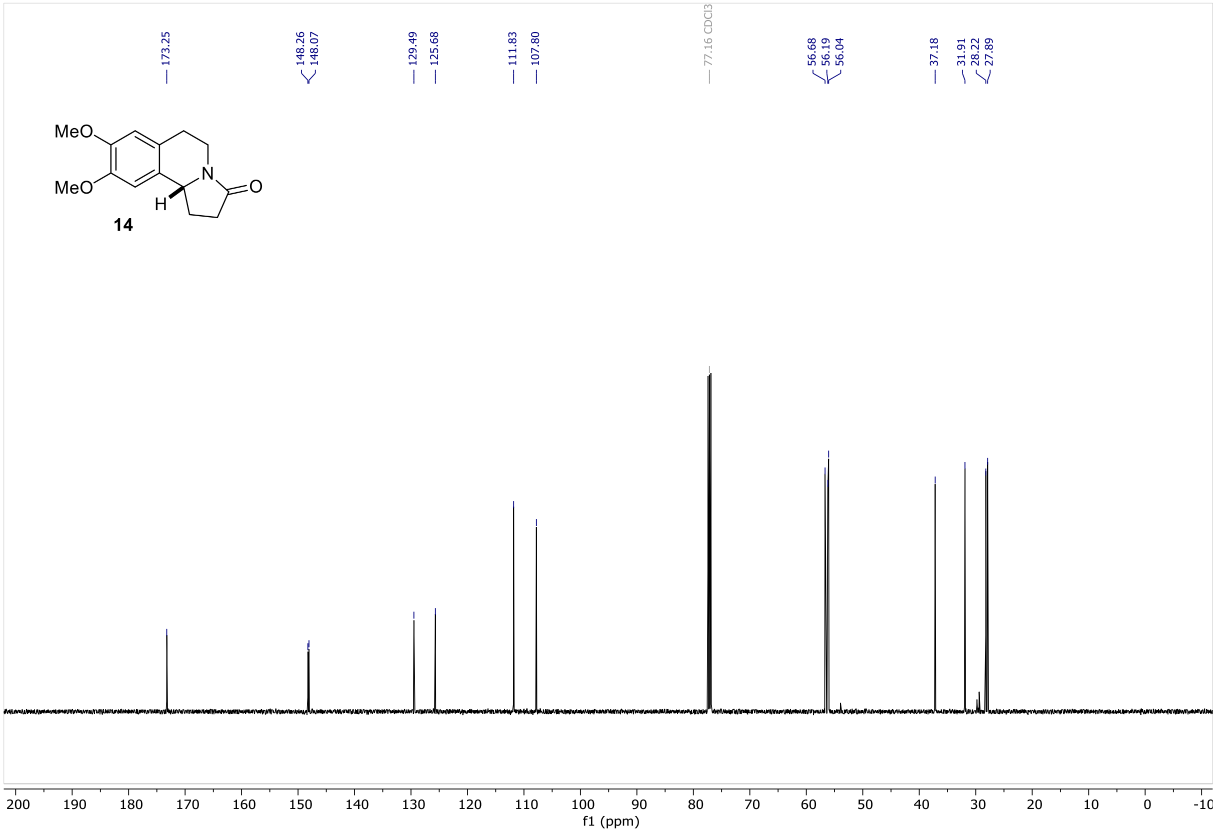

[13]**C-NMR** spectrum of compound **14.**

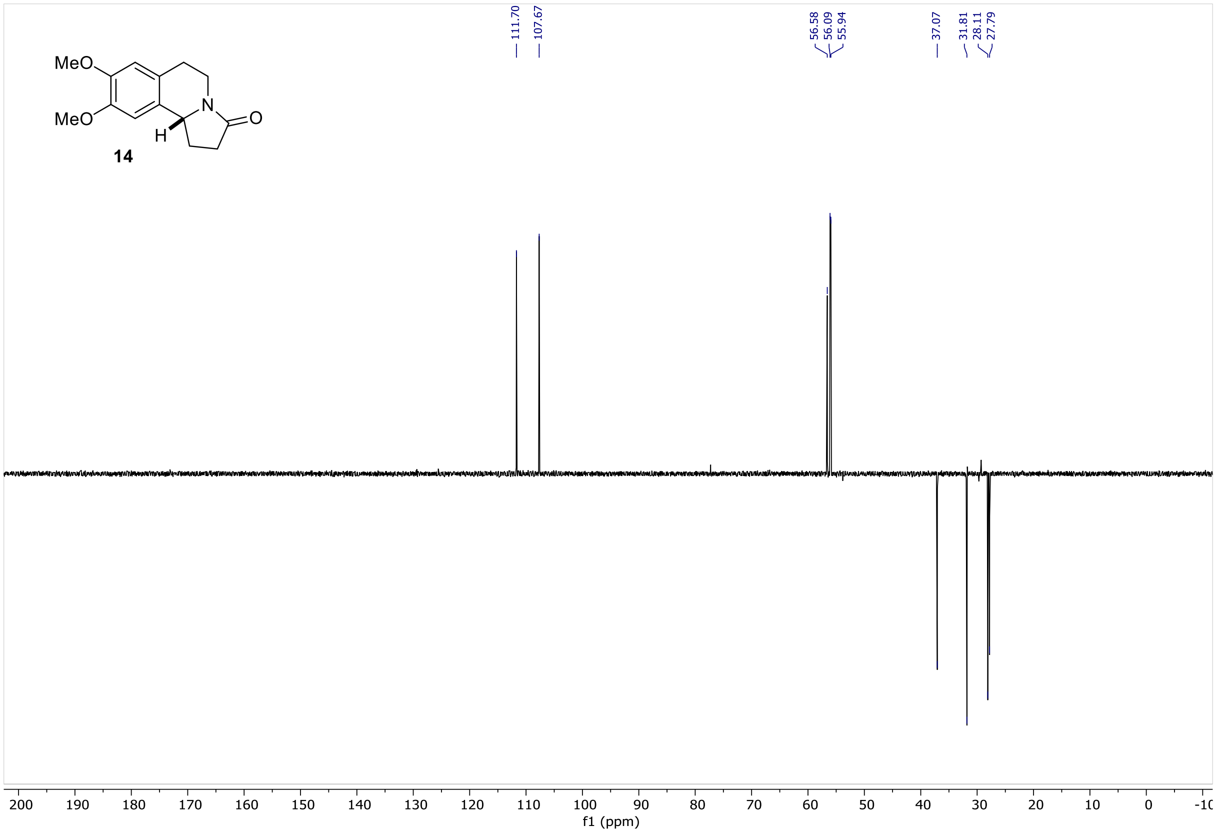

DEPT-135 NMR-spectrum of compound **14.**

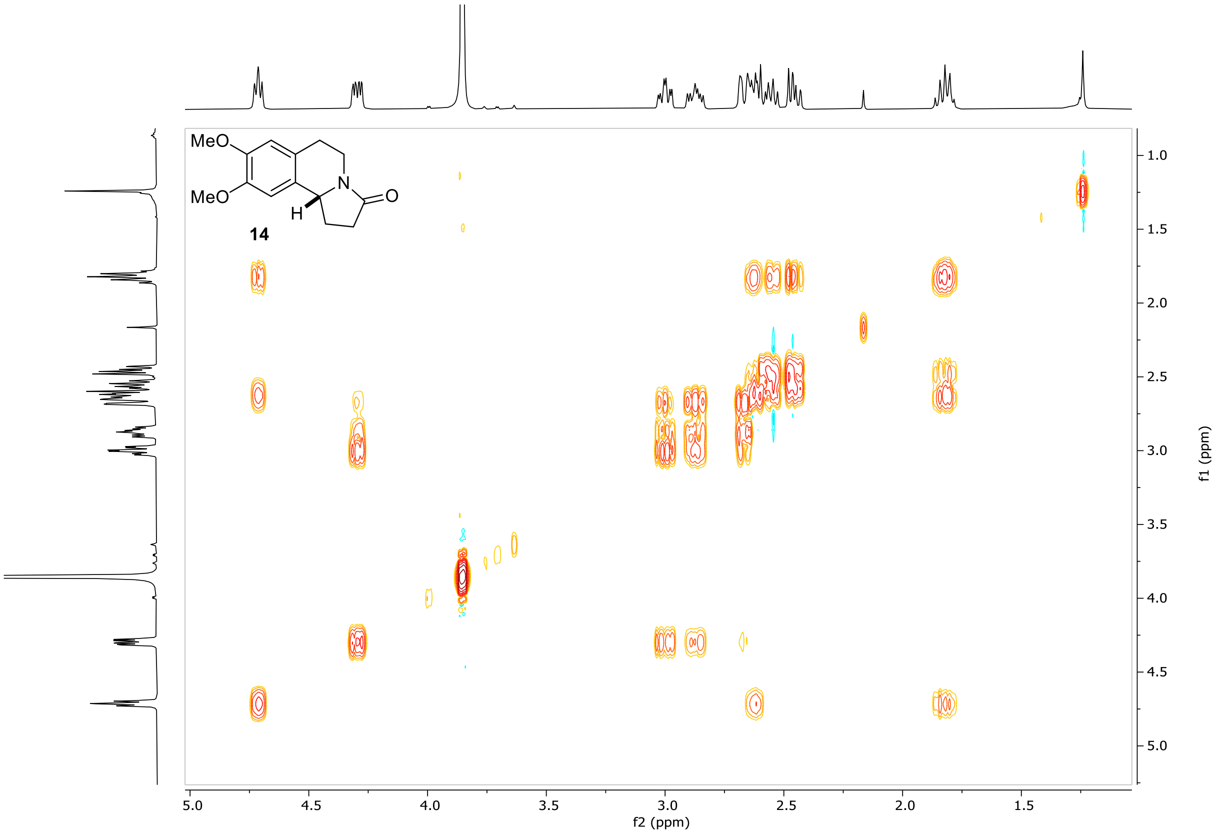

^{1}H-COSY NMR-spectrum of compound **14.**

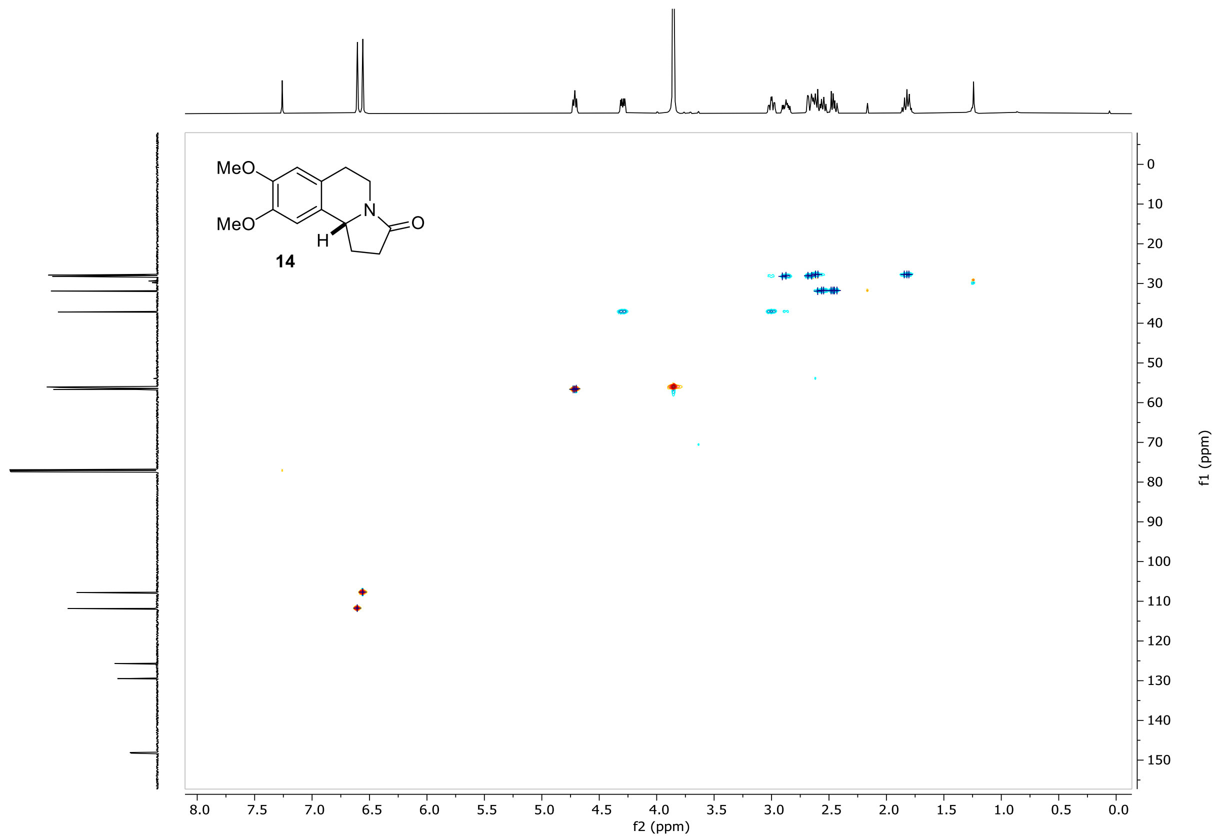

^{1}H-^{13}C-HSQC NMR-spectrum of compound **14.**

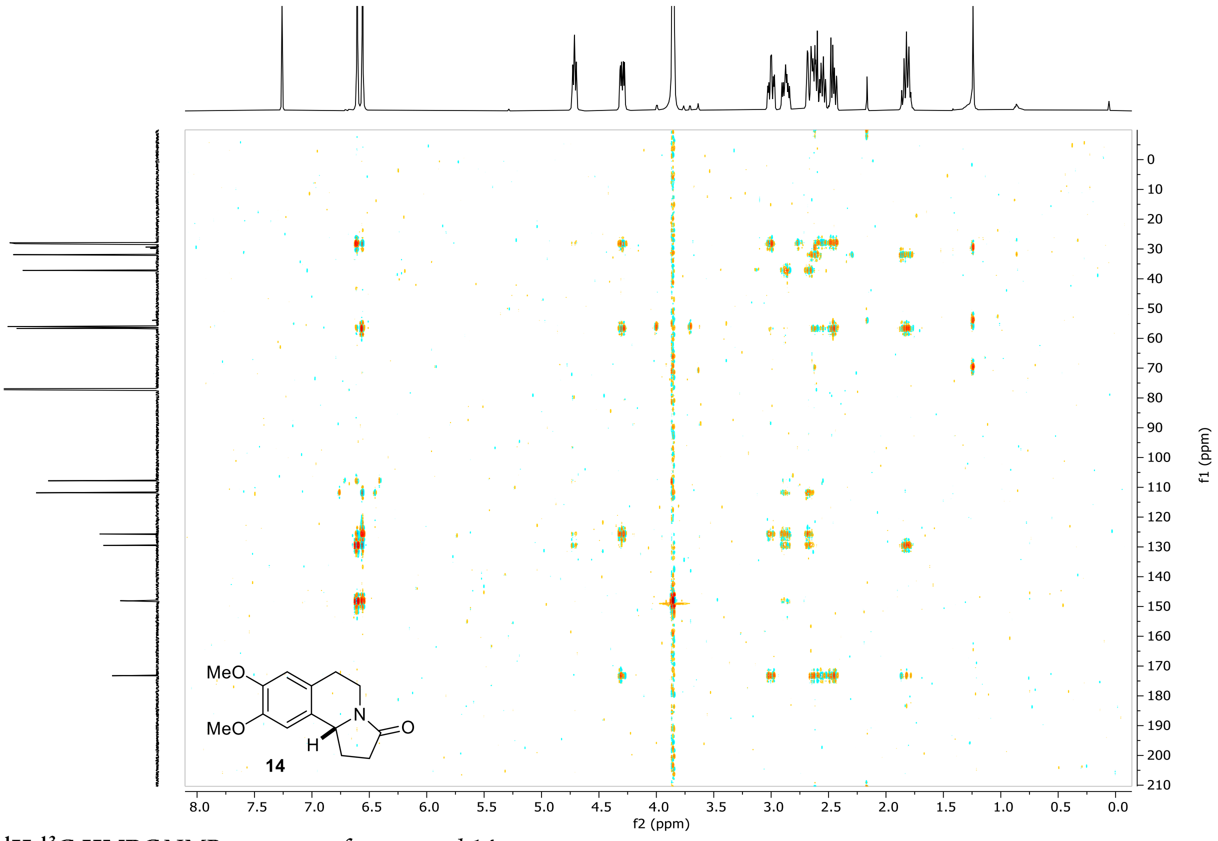

^{1}H-^{13}C-HMBC NMR-spectrum of compound **14.**

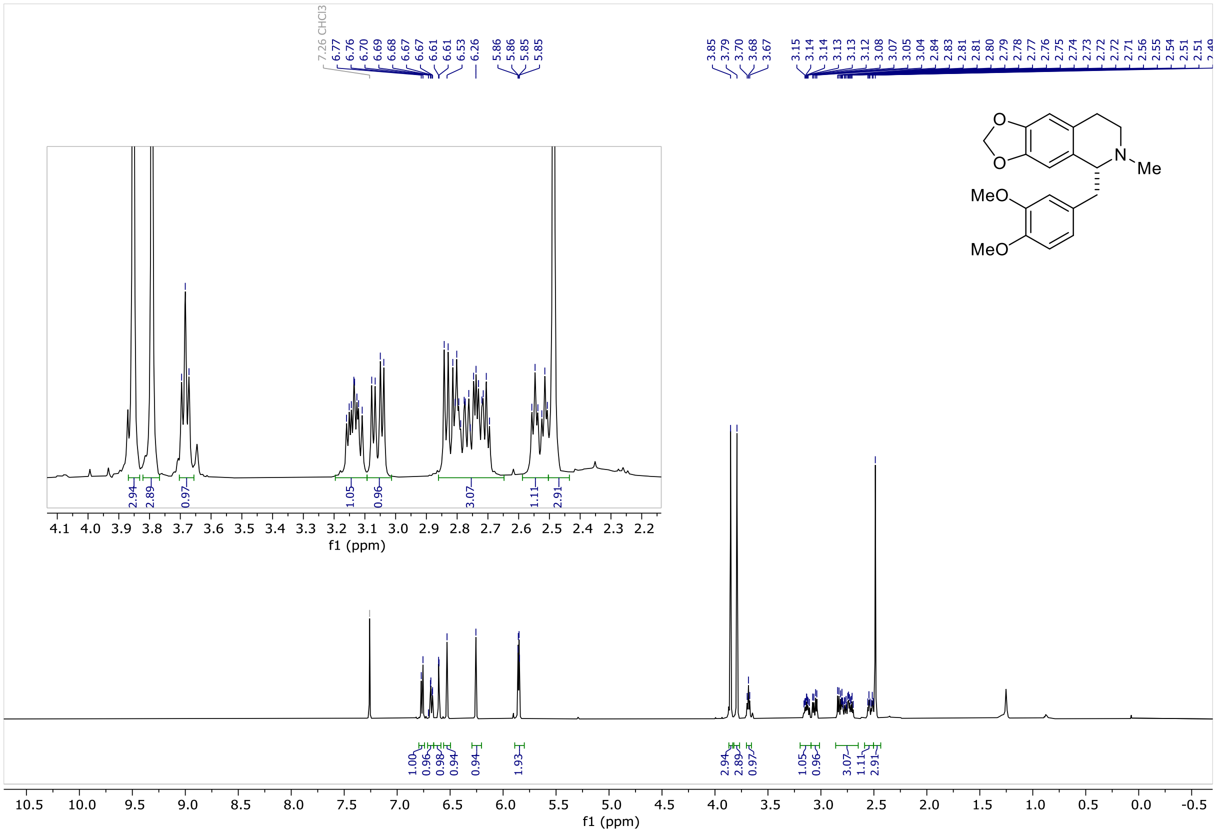

^{1}H-NMR-spectrum of Romneine.

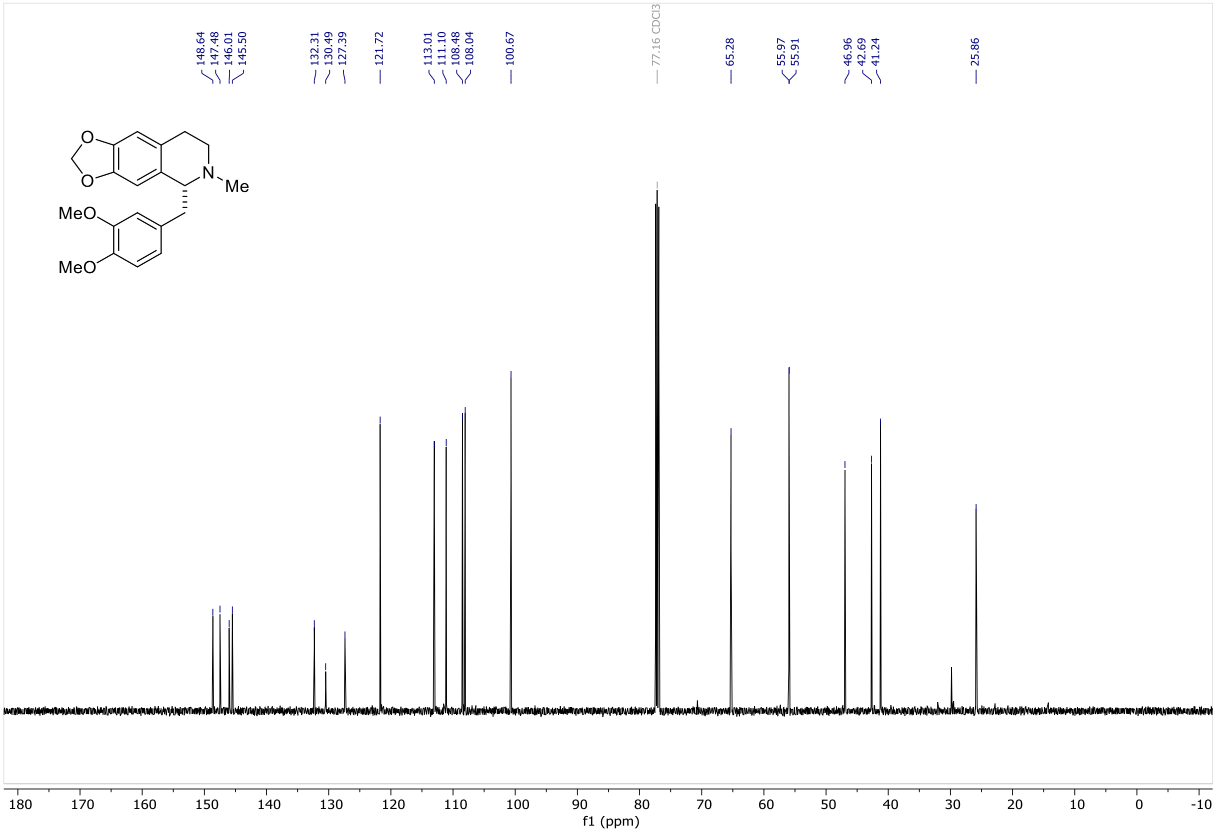

^{13}C-NMR-spectrum of Romneine.

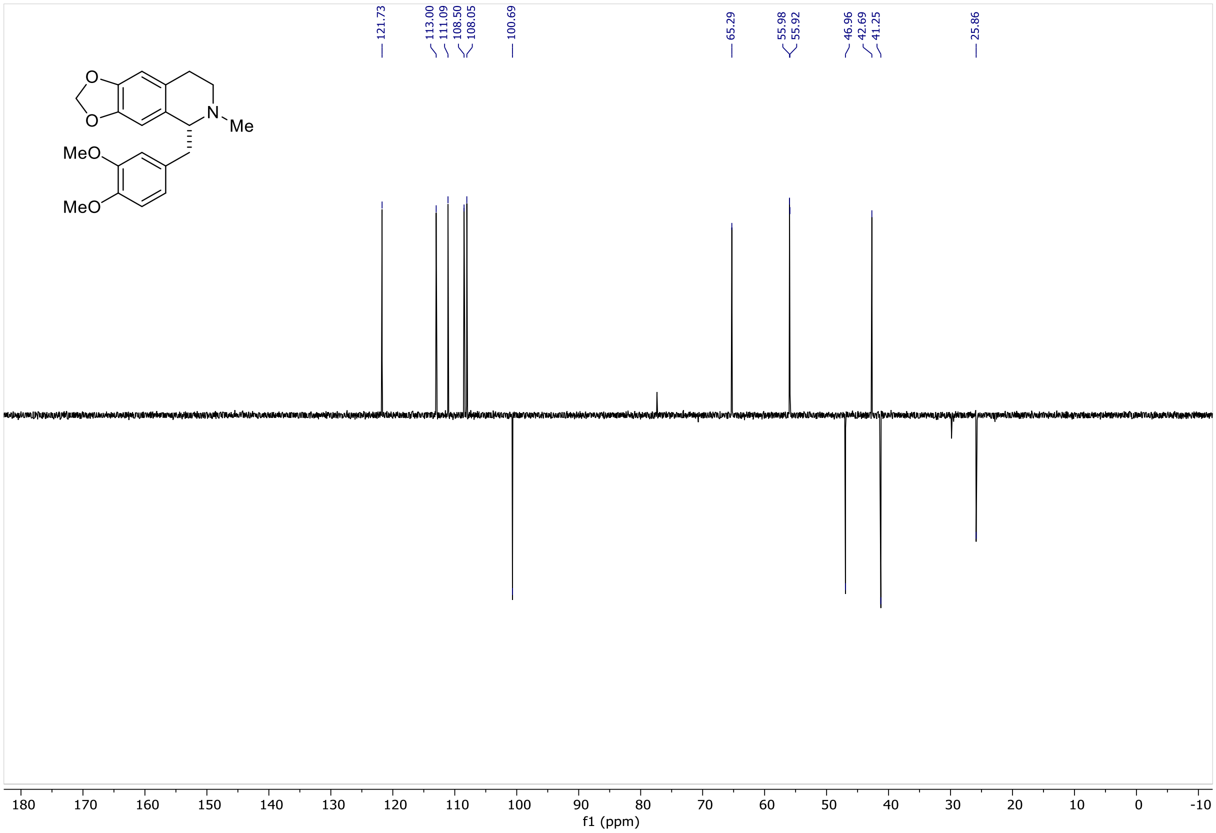

DEPT-135-NMR-spectrum of Romneine.

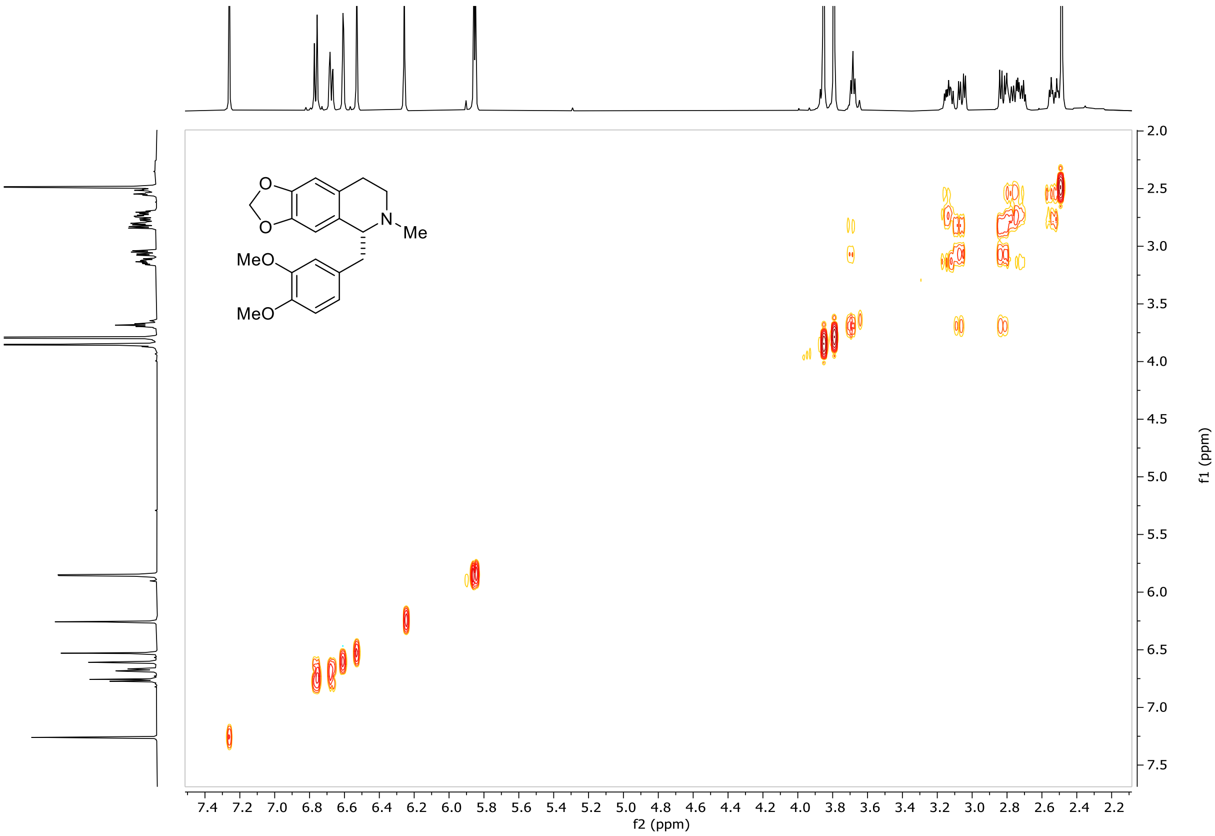

[1]**H-COSY-NMR**-spectrum of Romneine.

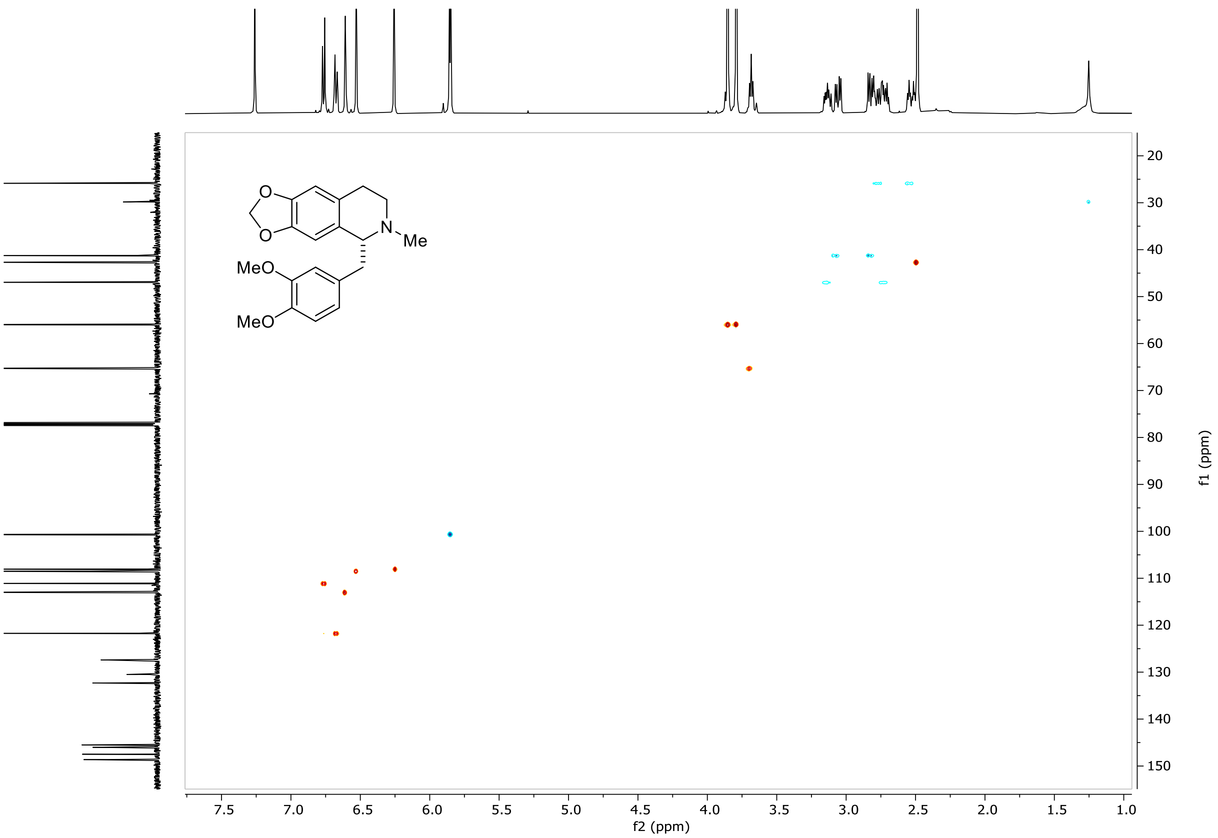

^{1}H-^{13}C-HSQC NMR-spectrum of Romneine.

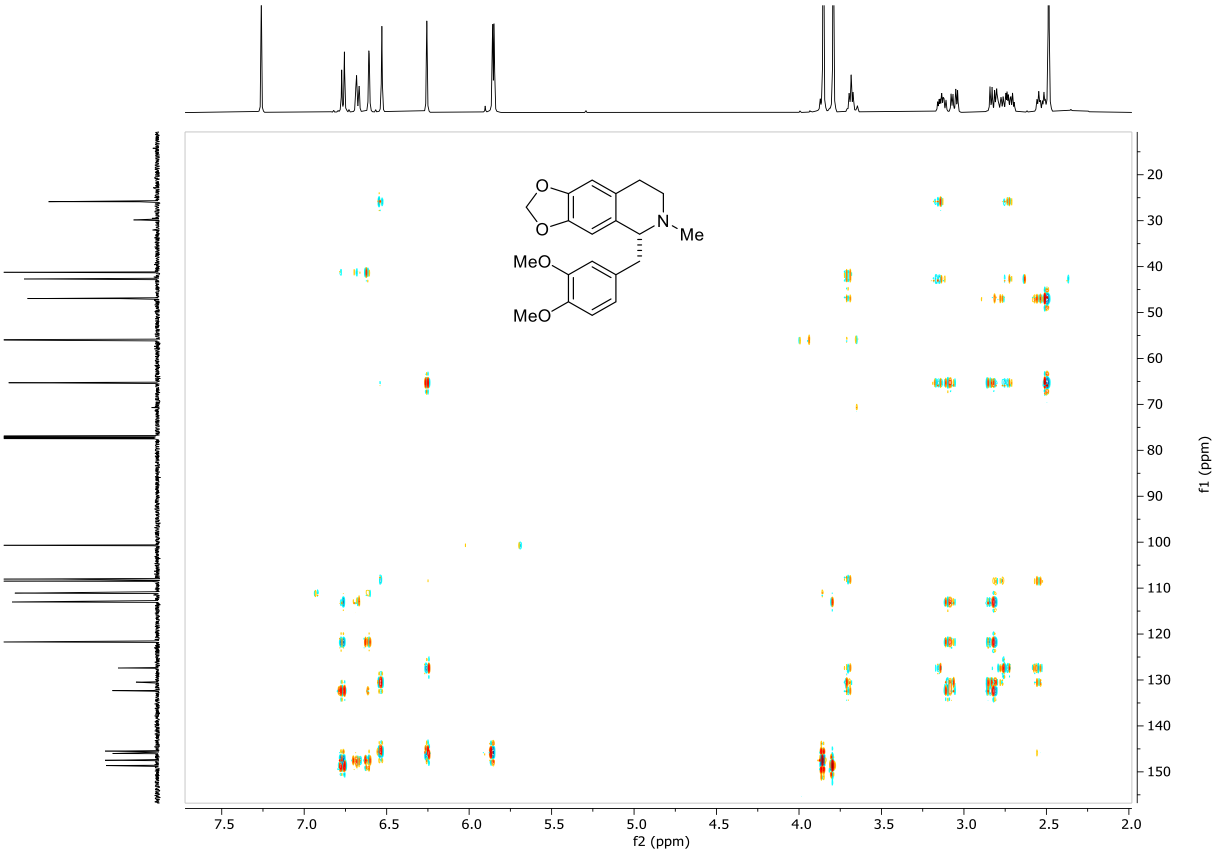

^{1}H-^{13}C-HMBC NMR-spectrum of Romneine.

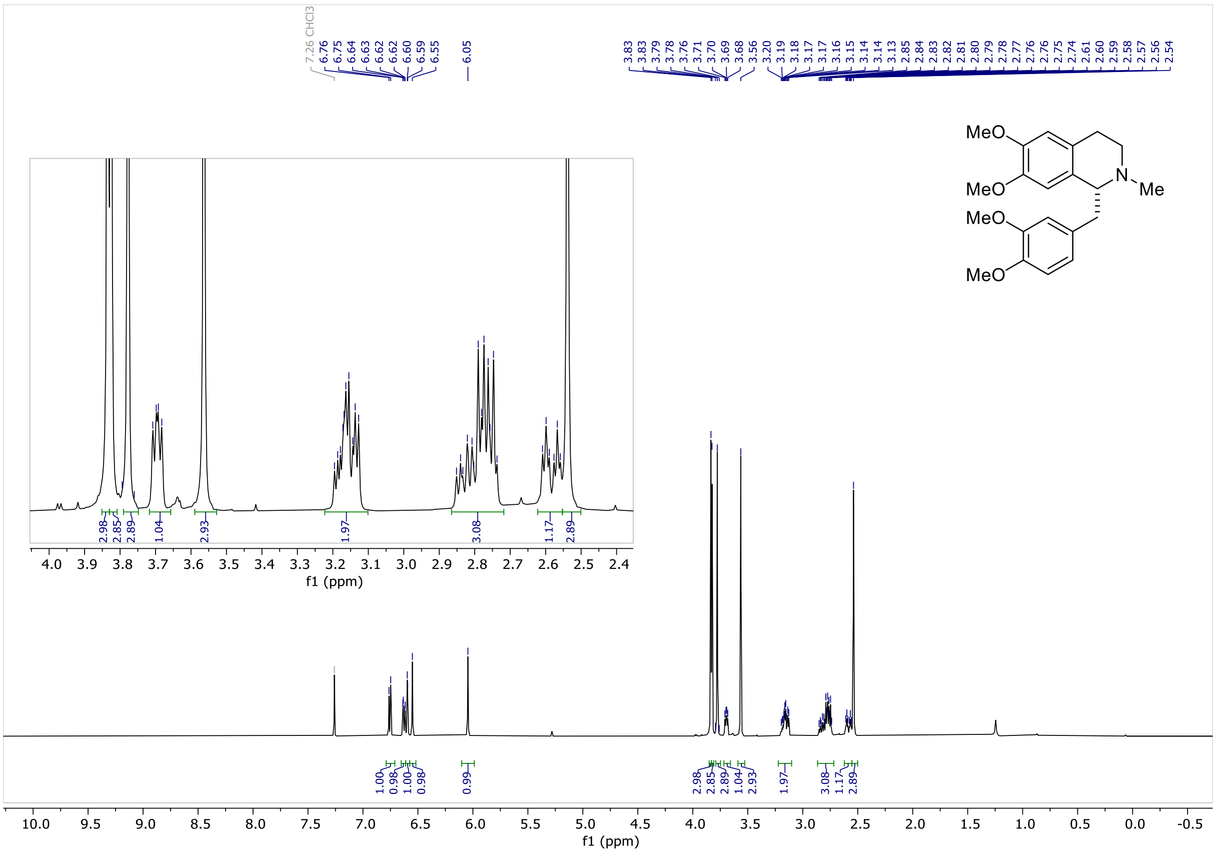

^{1}H-NMR spectrum of compound Laudanosine.

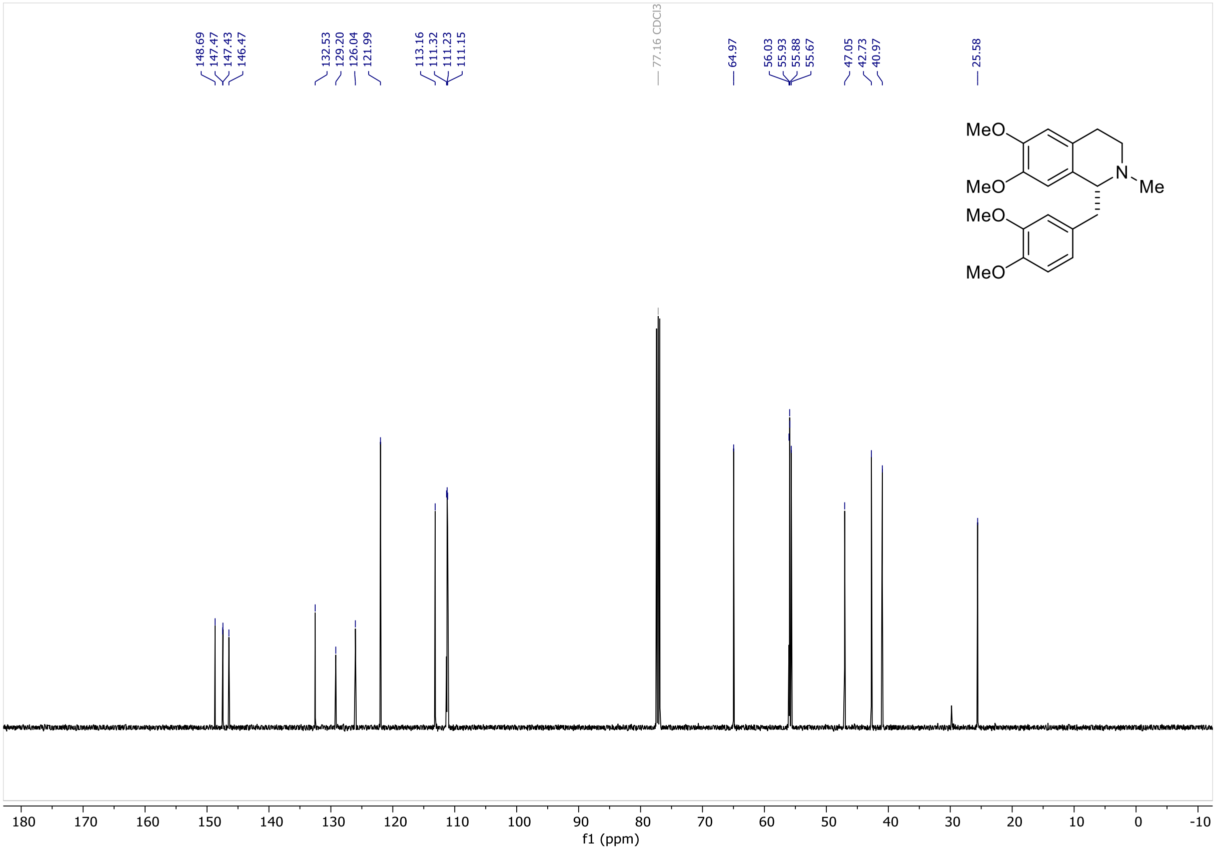

^{13}C-NMR spectrum of compound Laudanosine.

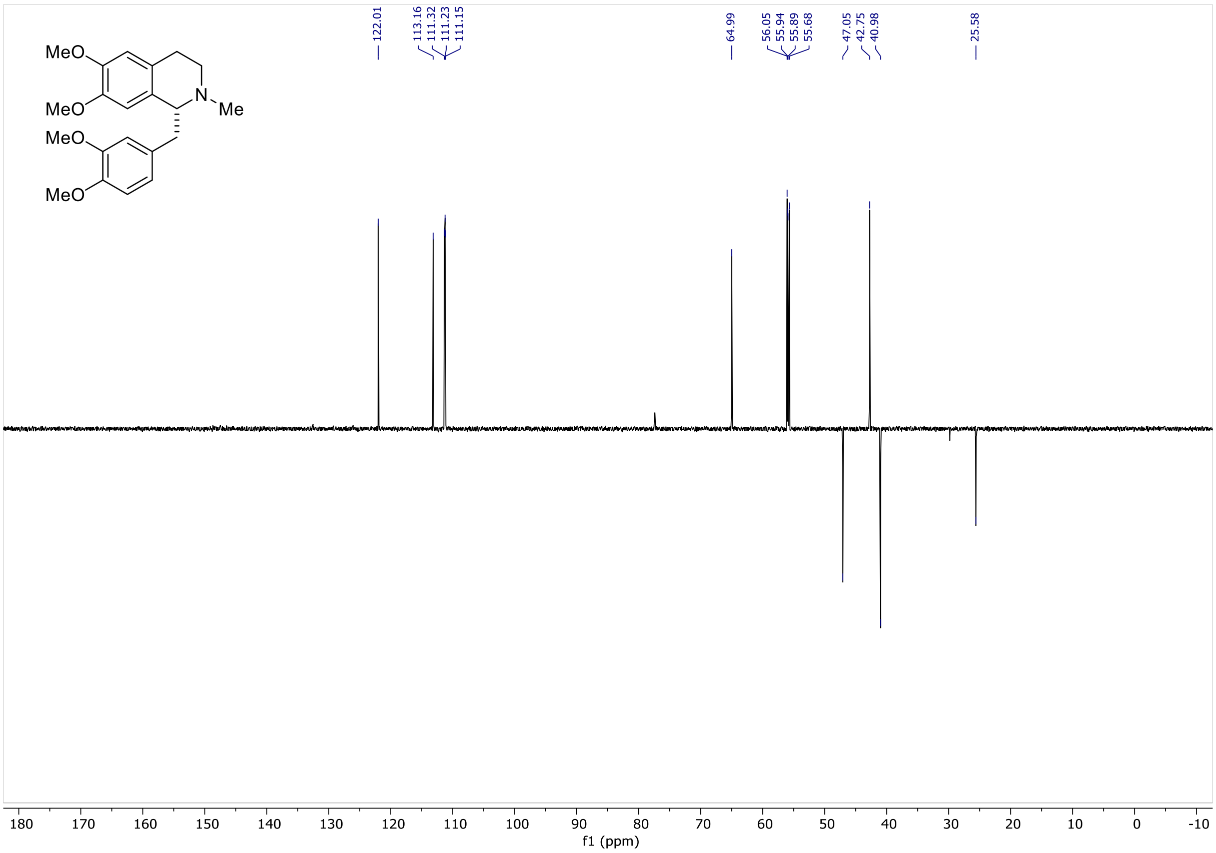

DEPT135-NMR spectrum of compound Laudanosine.

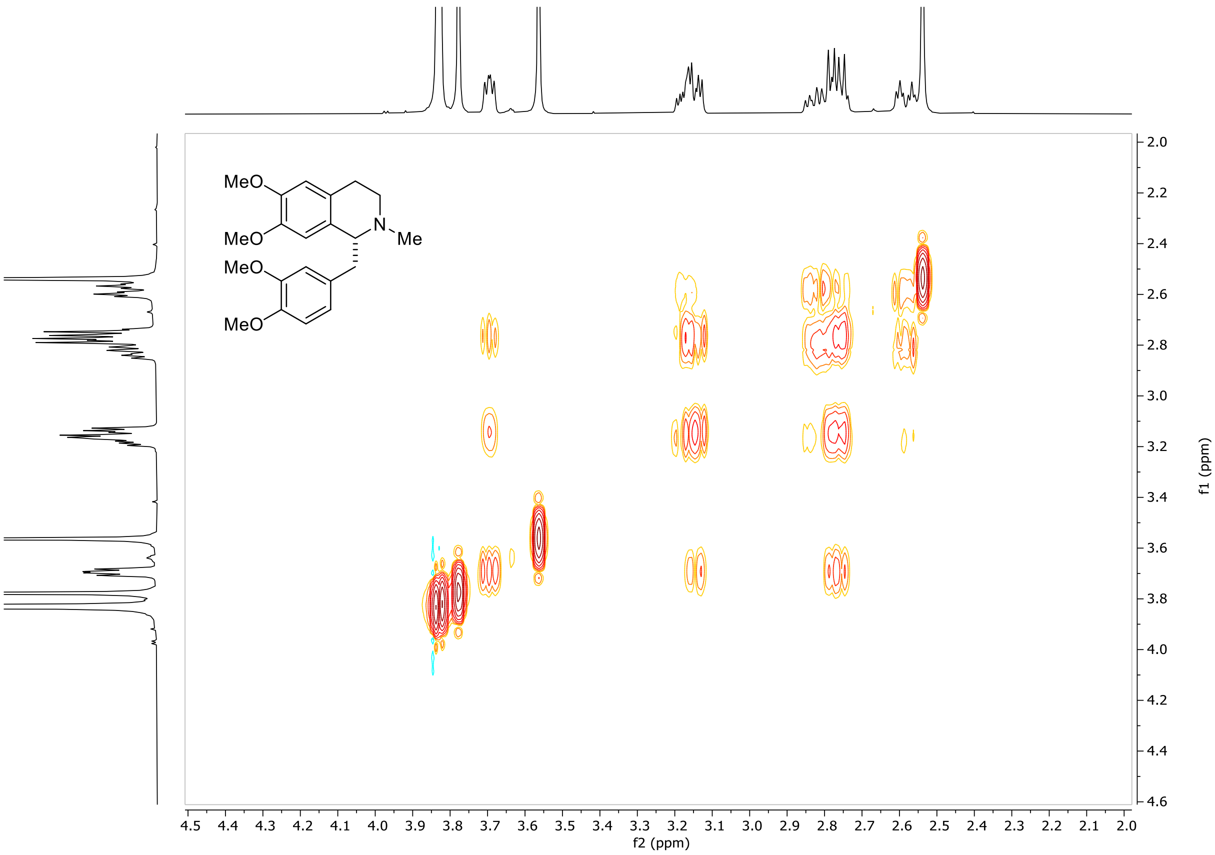

^{1}H-COSY-NMR spectrum of compound Laudanosine.

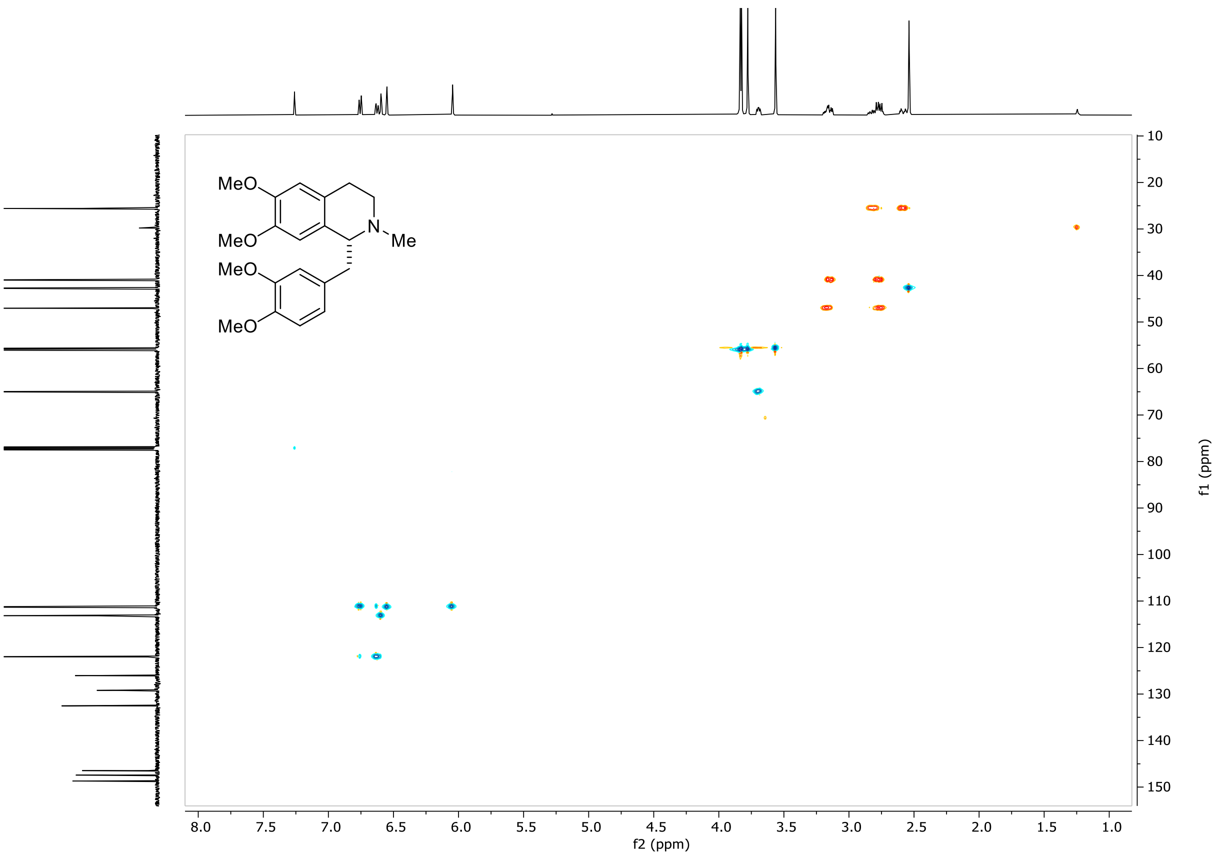

¹H-¹³C-HSQC NMR spectrum of Laudanosine.

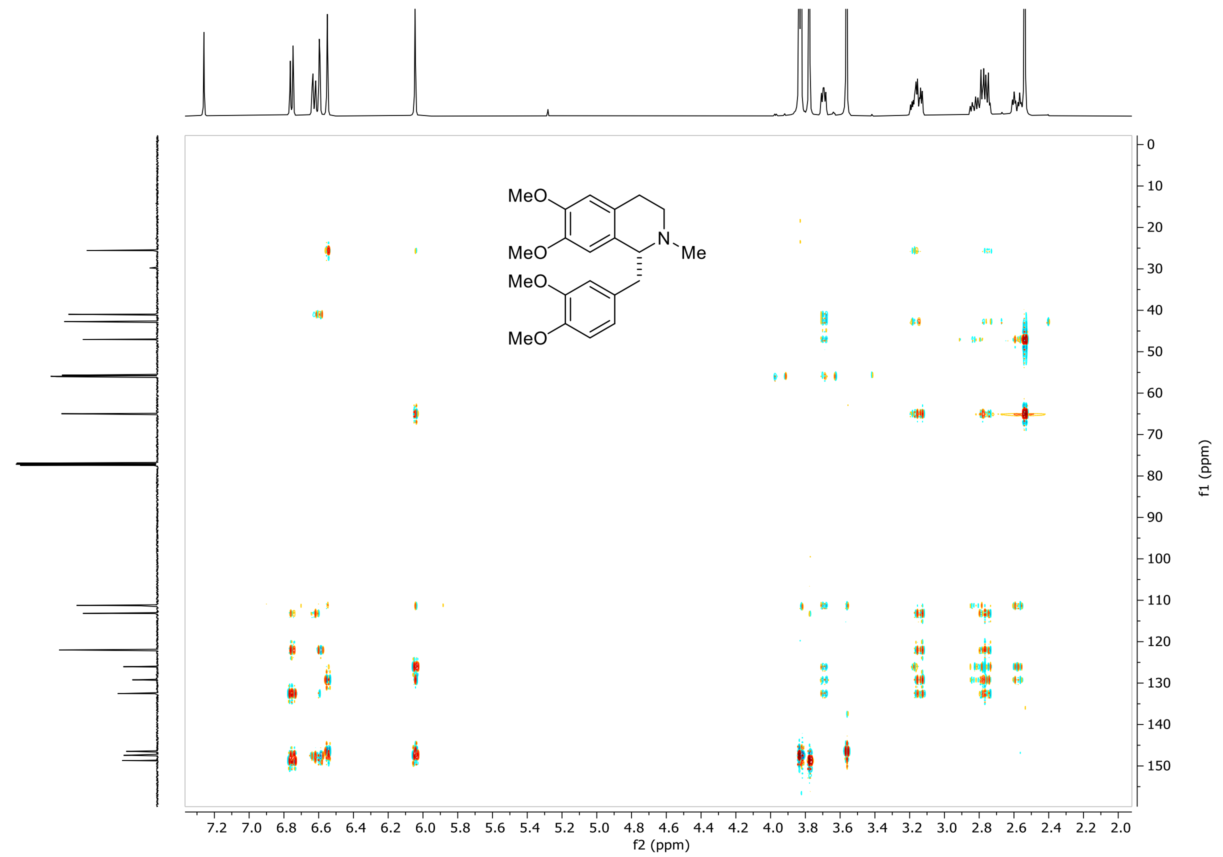

¹H-¹³C-HMBC NMR spectrum of compound Laudanosine.

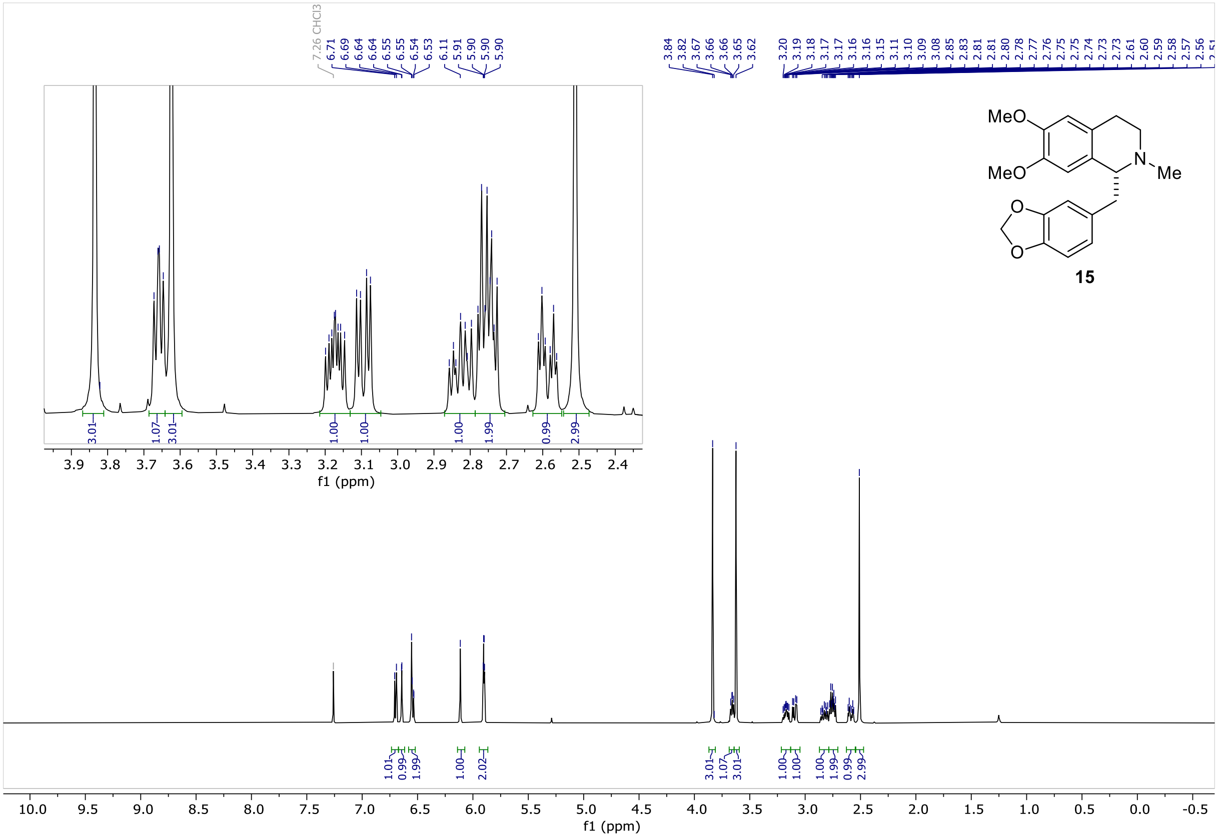

[1]**H-NMR** spectrum of compound **15**.

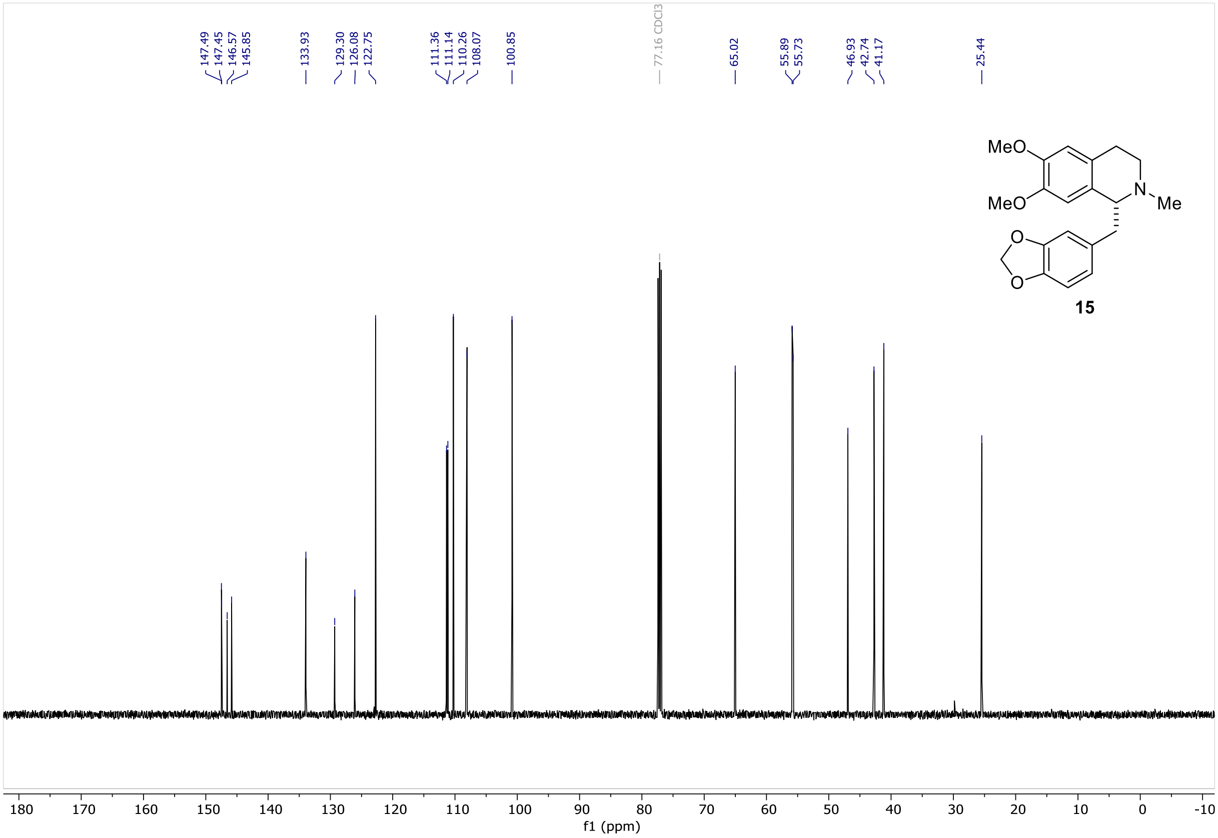

[13]**C-NMR** spectrum of compound **15**.

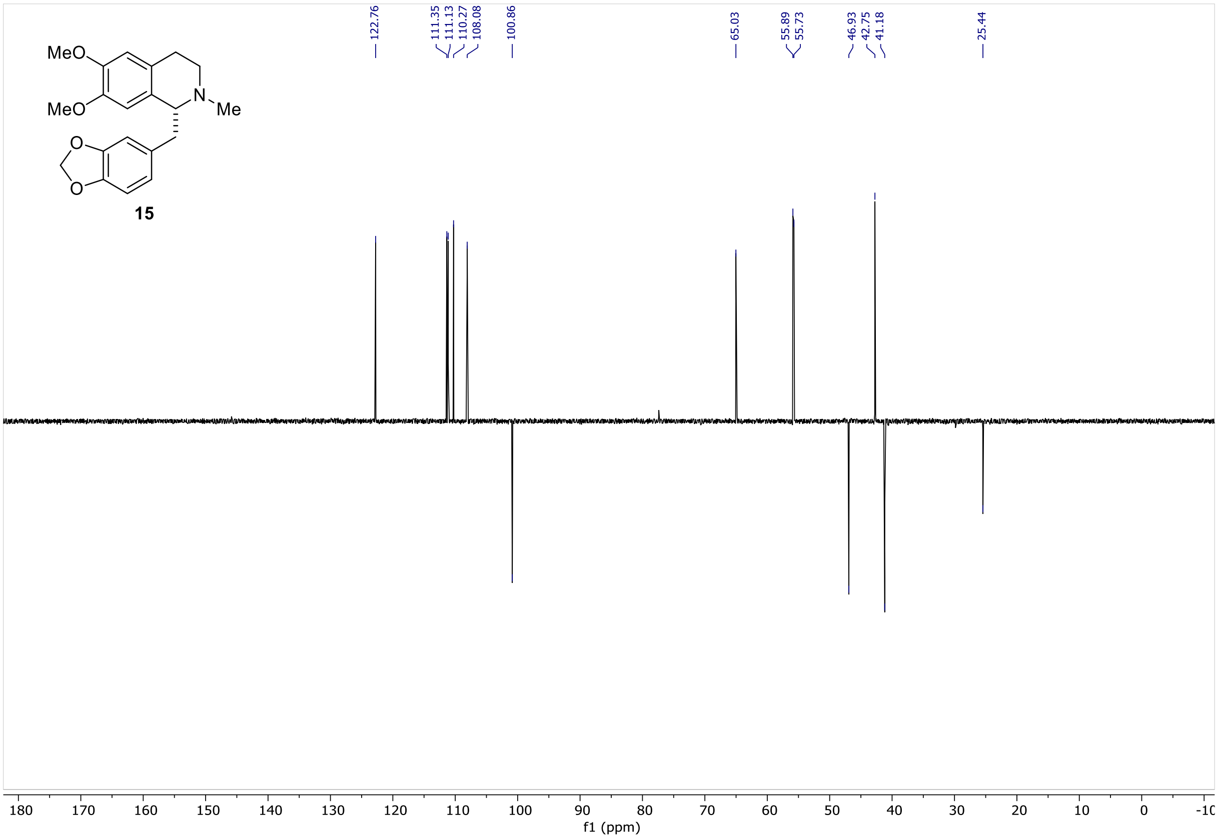

DEPT-135 NMR spectrum of compound **15**.

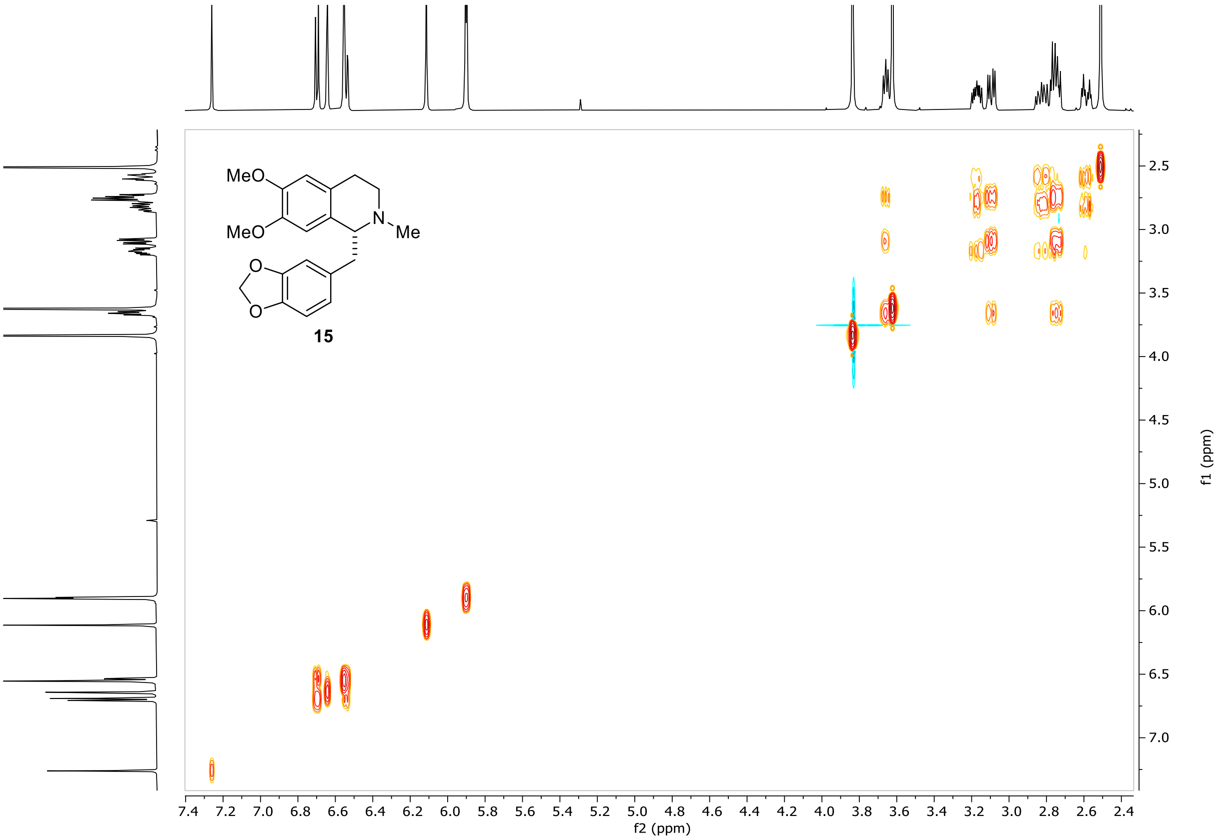

^{1}H-COSY NMR spectrum of compound **15**.

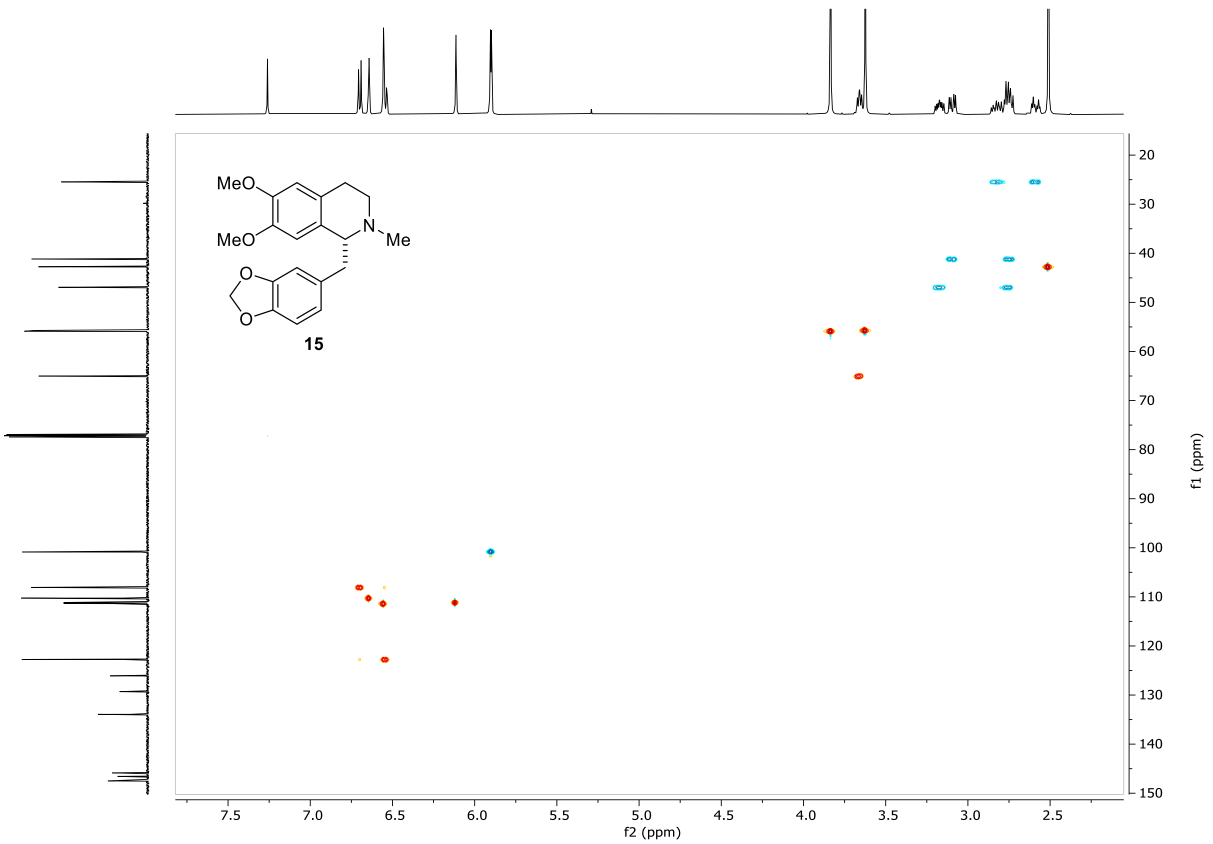

^{1}H-^{13}C-HSQC NMR spectrum of compound **15**.

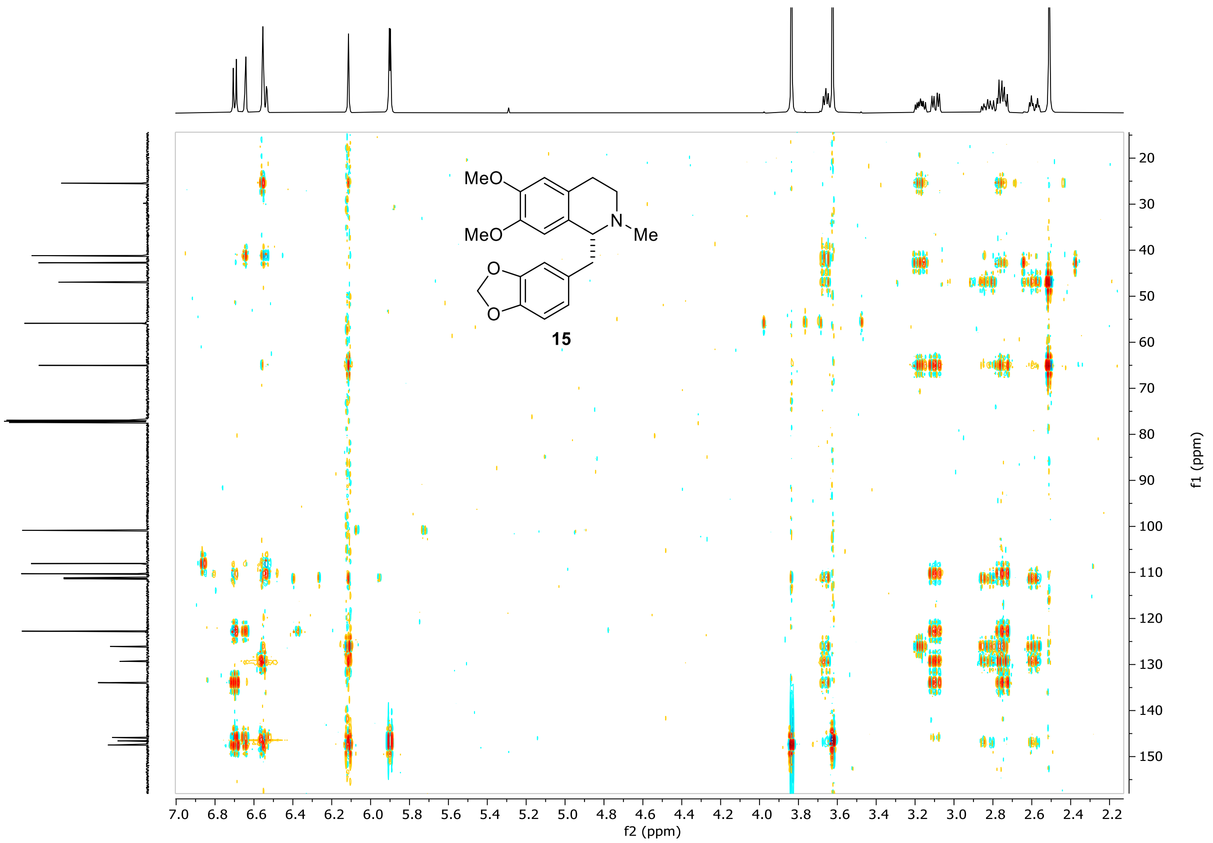

^{1}H-^{13}C-HMBC NMR spectrum of compound **15**.

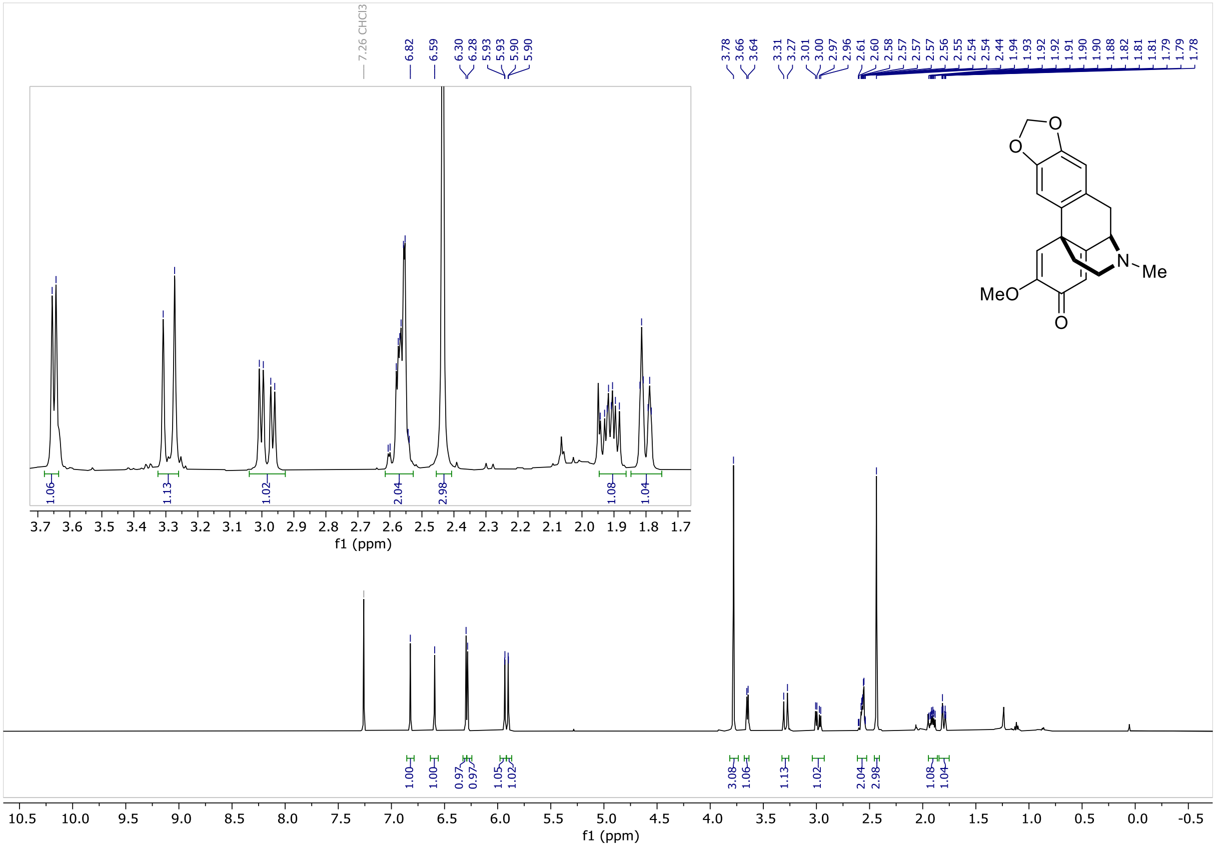

^{1}H-NMR spectrum of Amurine**.**

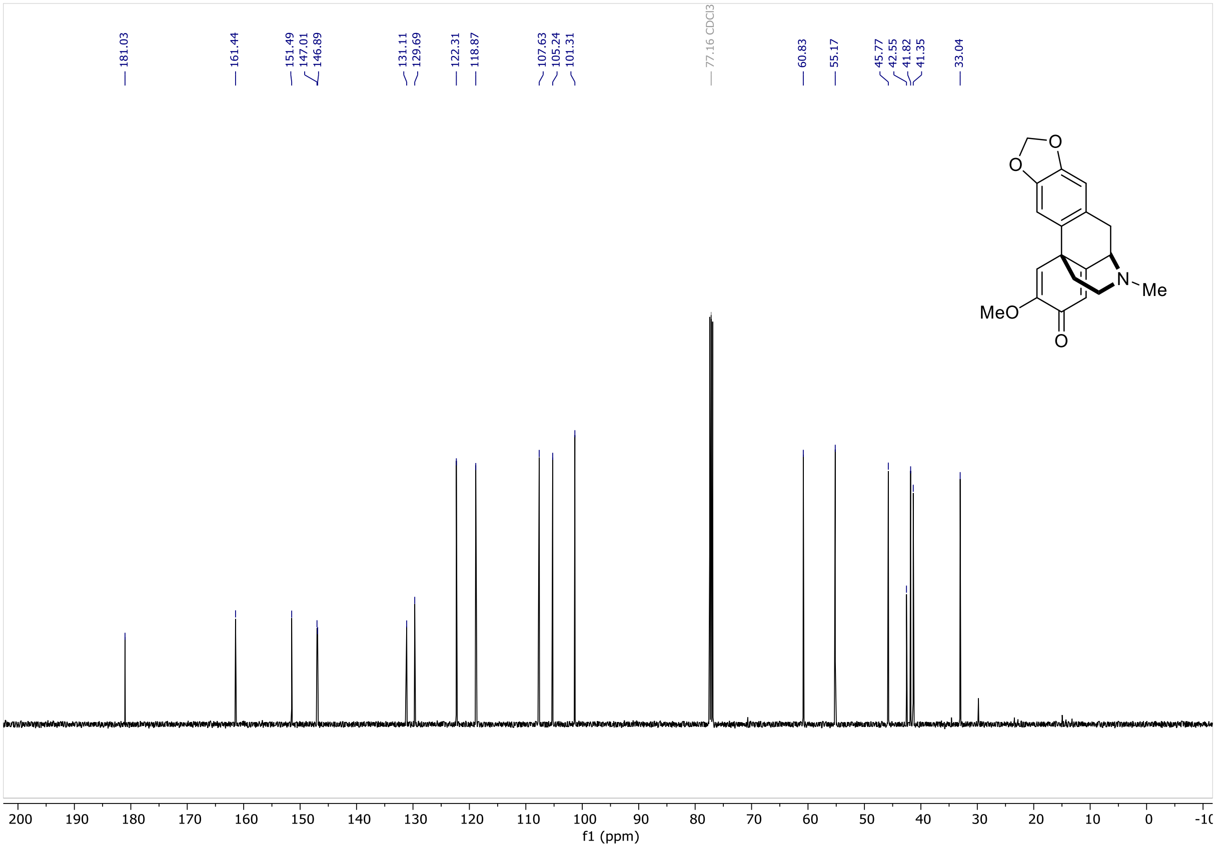

^{13}C-NMR spectrum of Amurine**.**

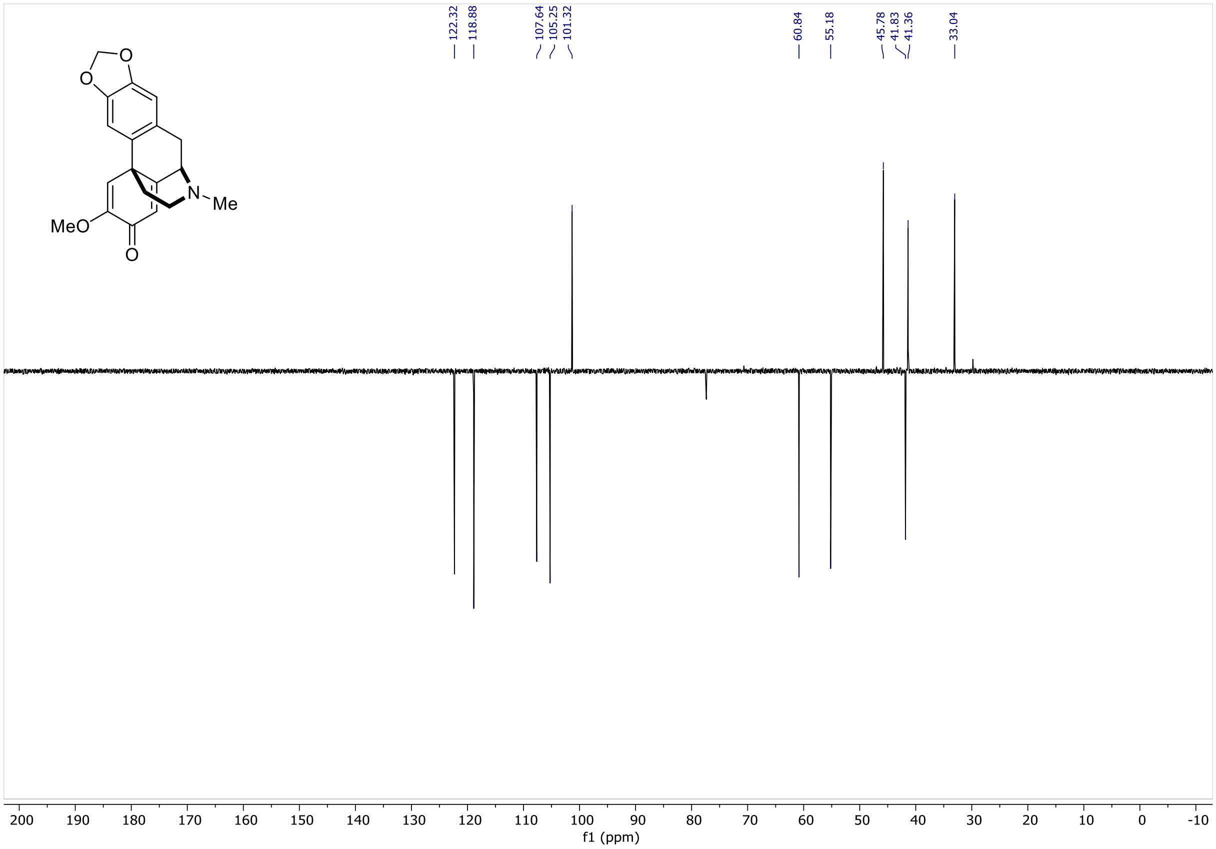

DEPT-135 NMR spectrum of Amurine.

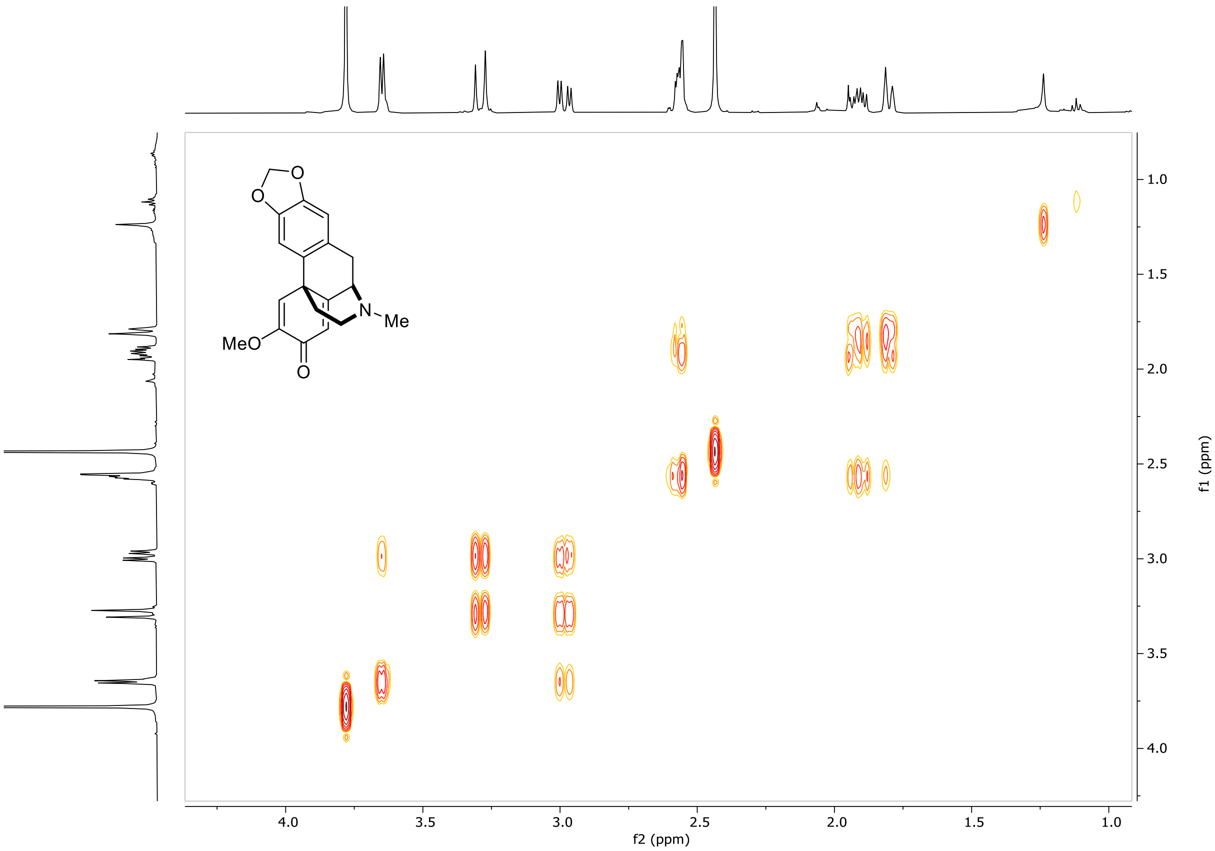

^{1}H-COSY NMR spectrum of Amurine.

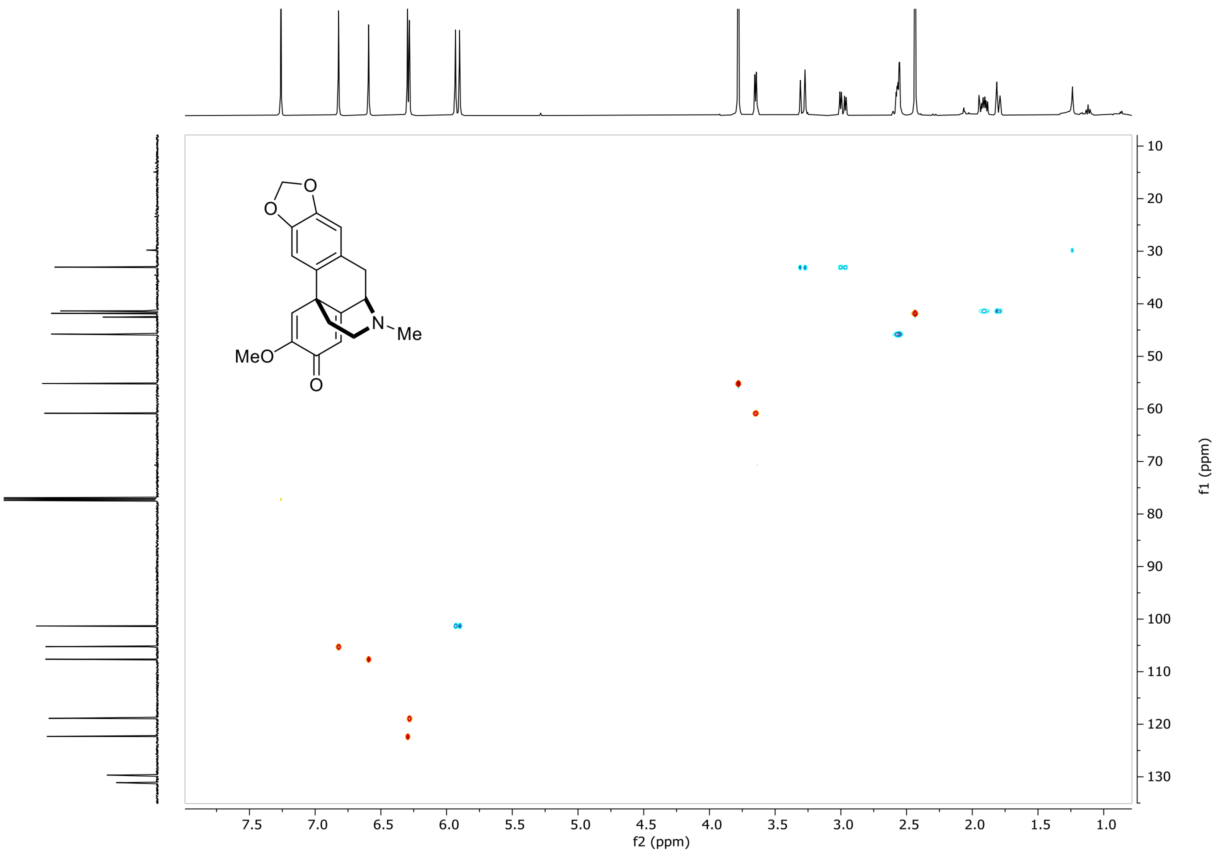

^{1}H-^{13}C-HSQC NMR spectrum of Amurine.

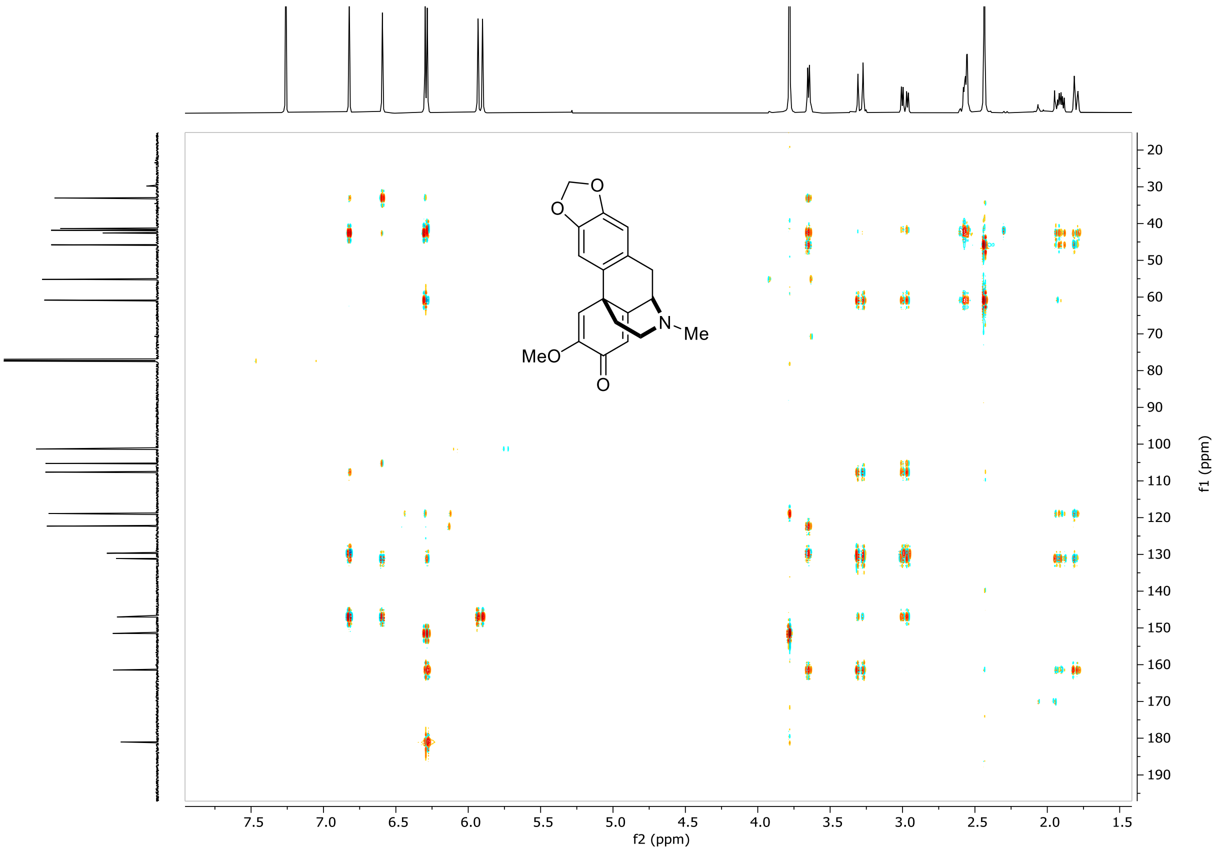

^{1}H-^{13}C-HMBC NMR spectrum of Amurine.

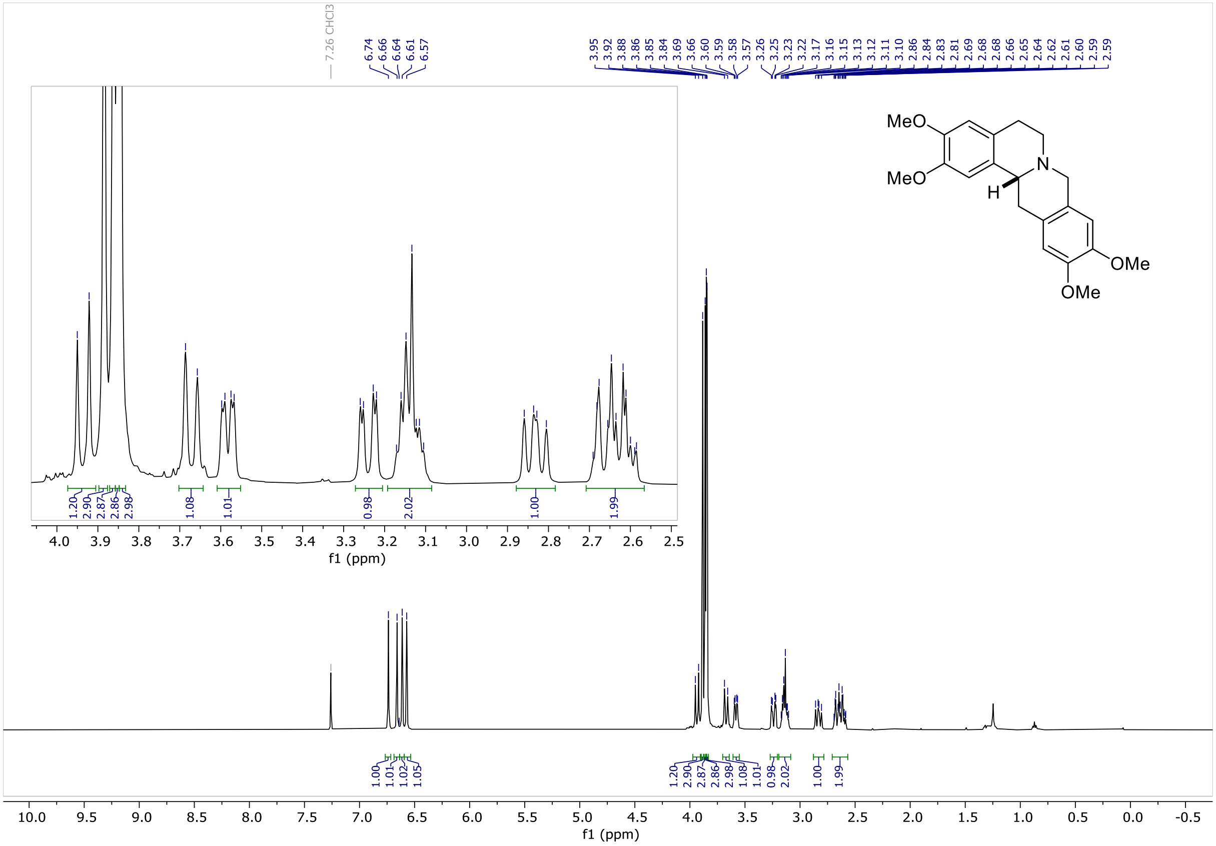

^{1}H-NMR spectrum of Xylopinine.

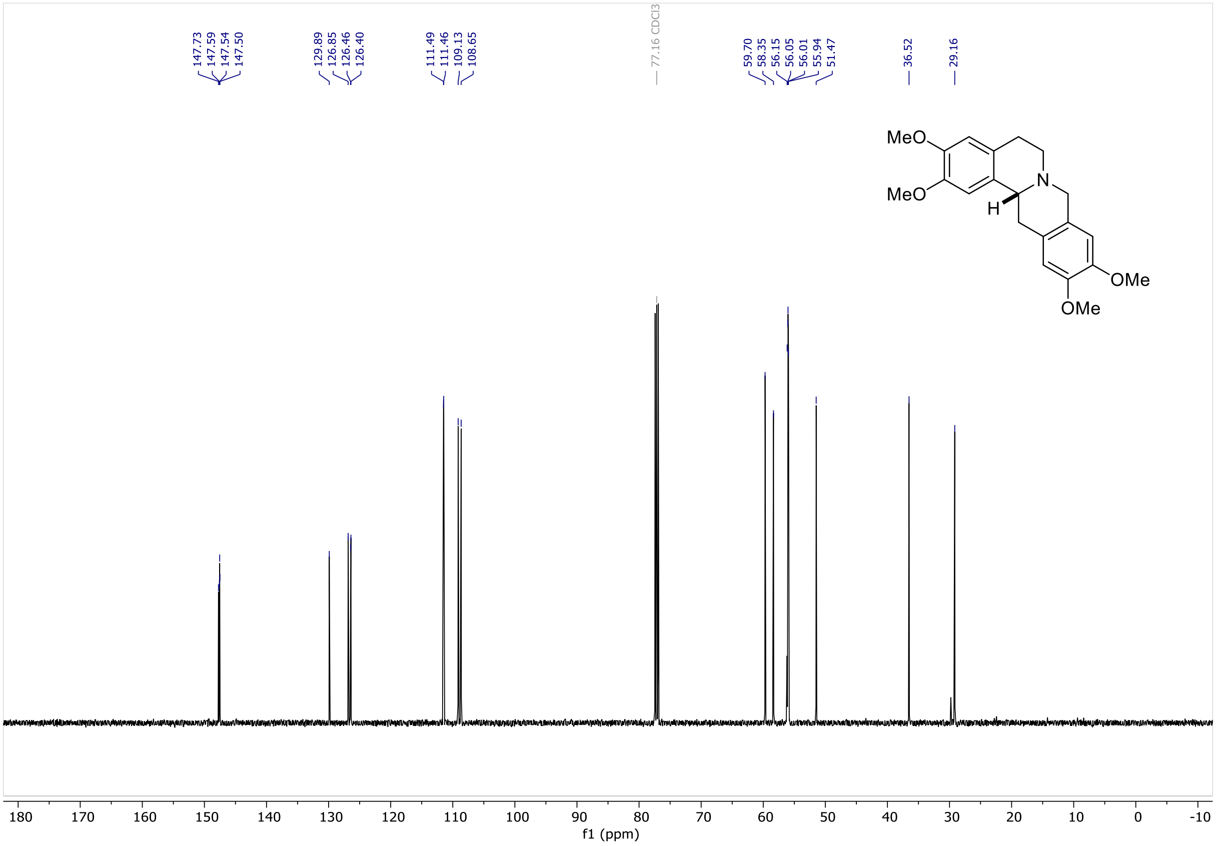

^{13}C-NMR spectrum of Xylopinine.

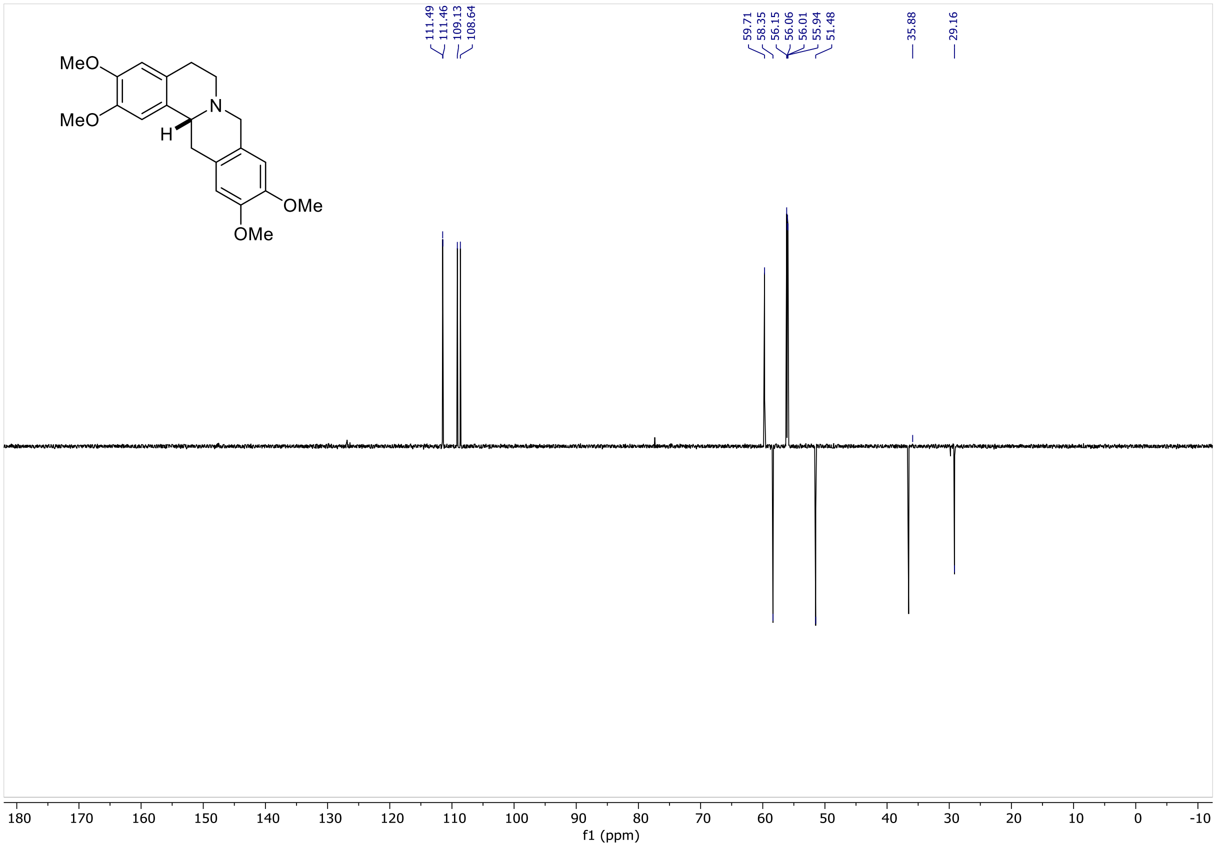

DEPT135-NMR spectrum of Xylopinine.

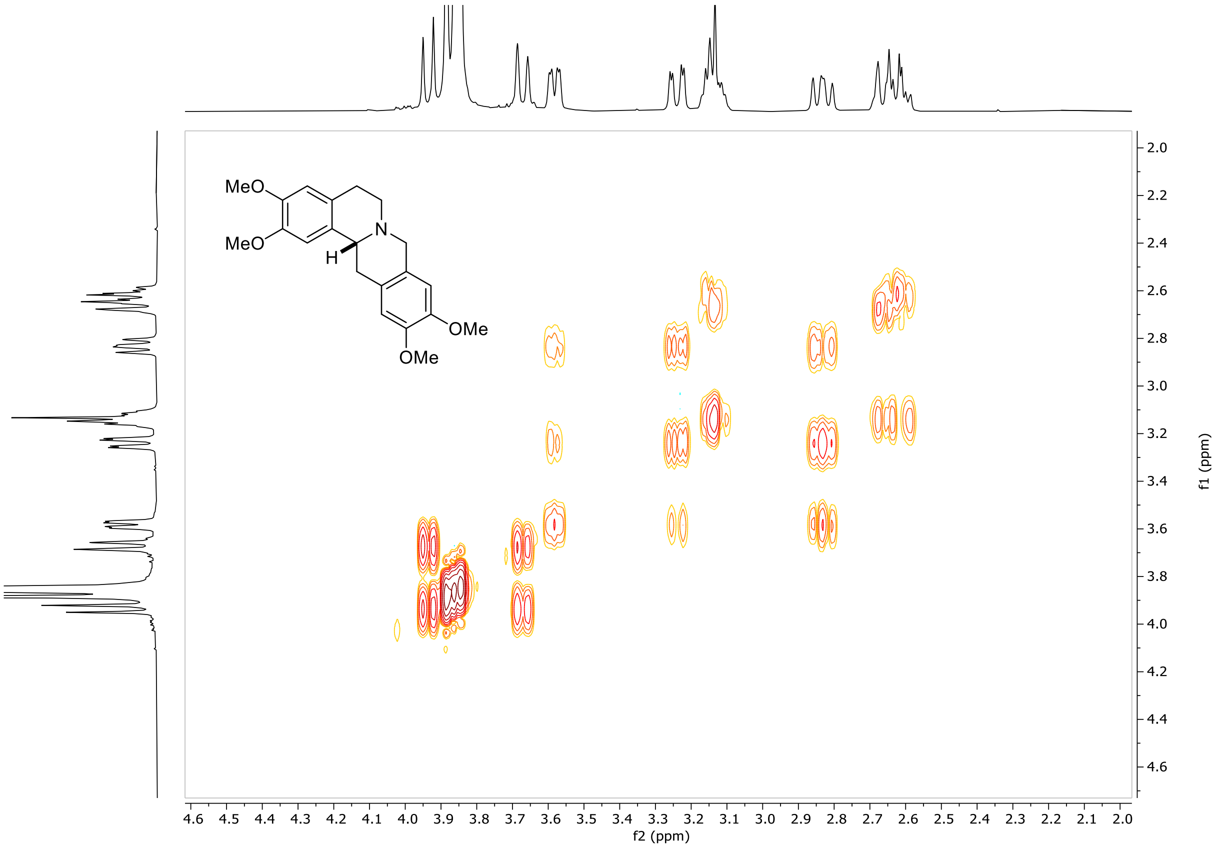

[1]**H-COSY NMR** spectrum of Xylopinine.

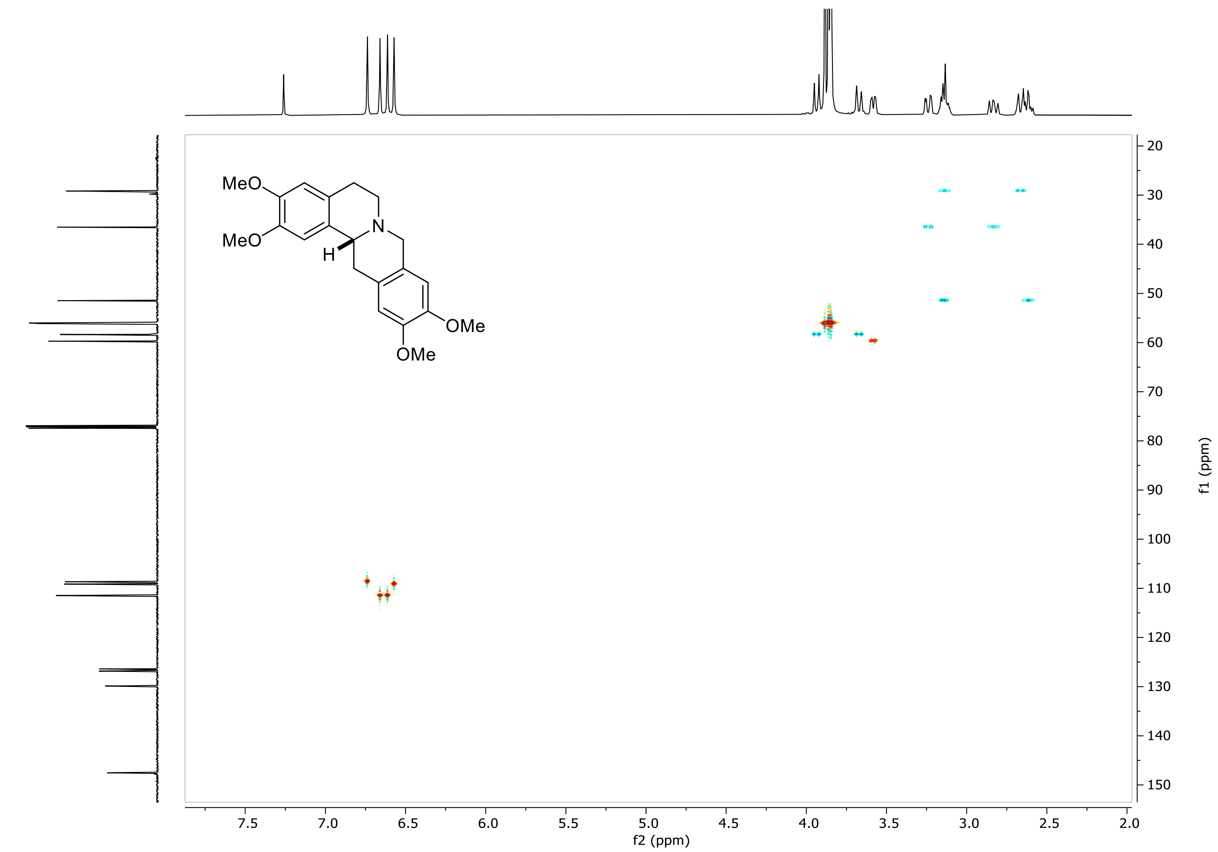

¹H-¹³C-HSQC NMR spectrum of Xylopinine.

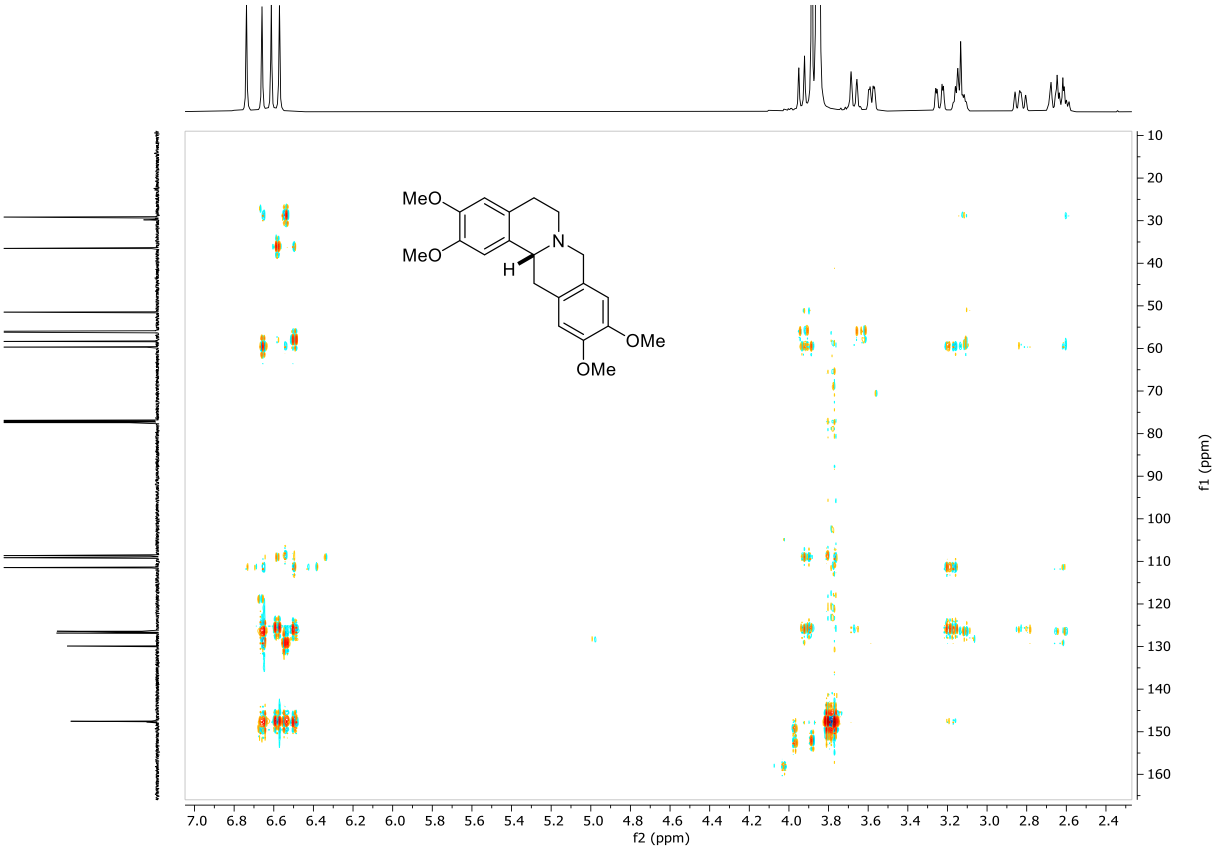

¹H-¹³C-HMBC NMR spectrum of Xylopinine.

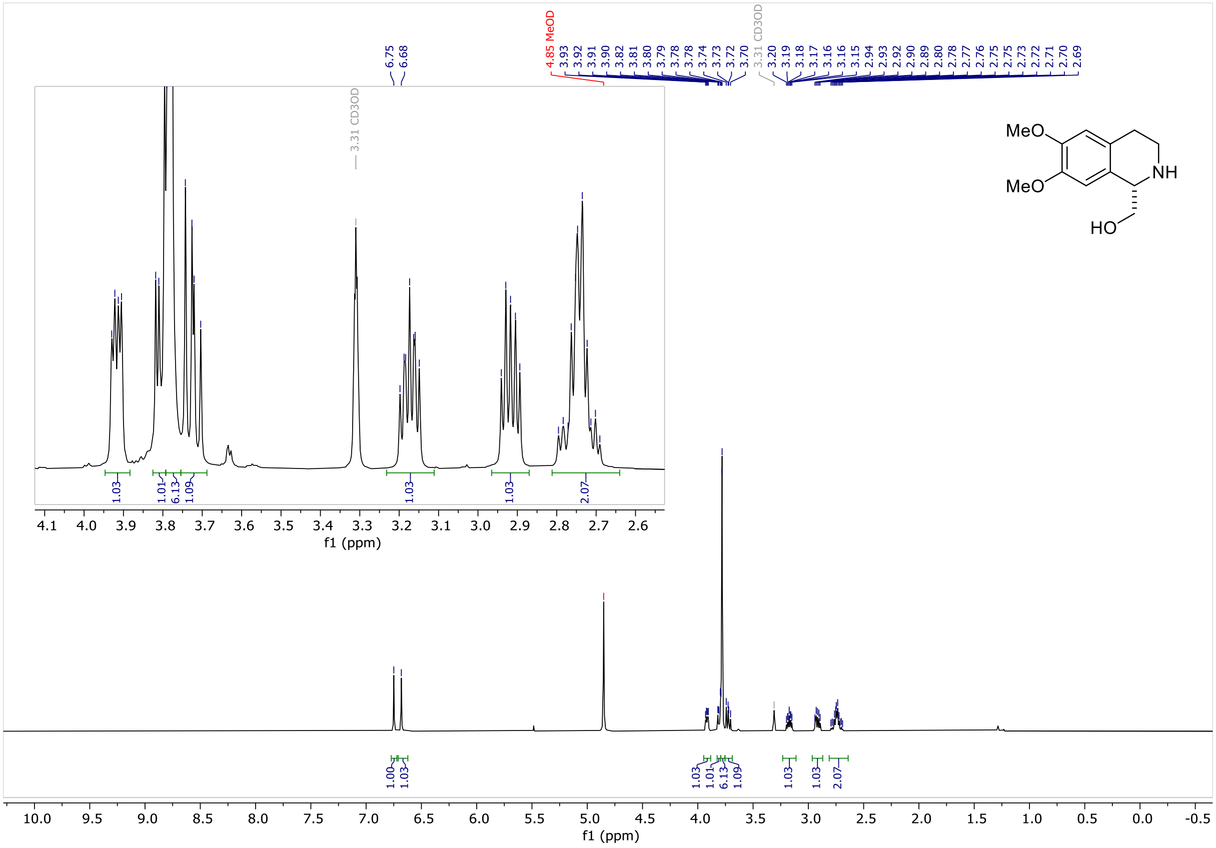

^{1}H-NMR spectrum of Calycotomine**.**

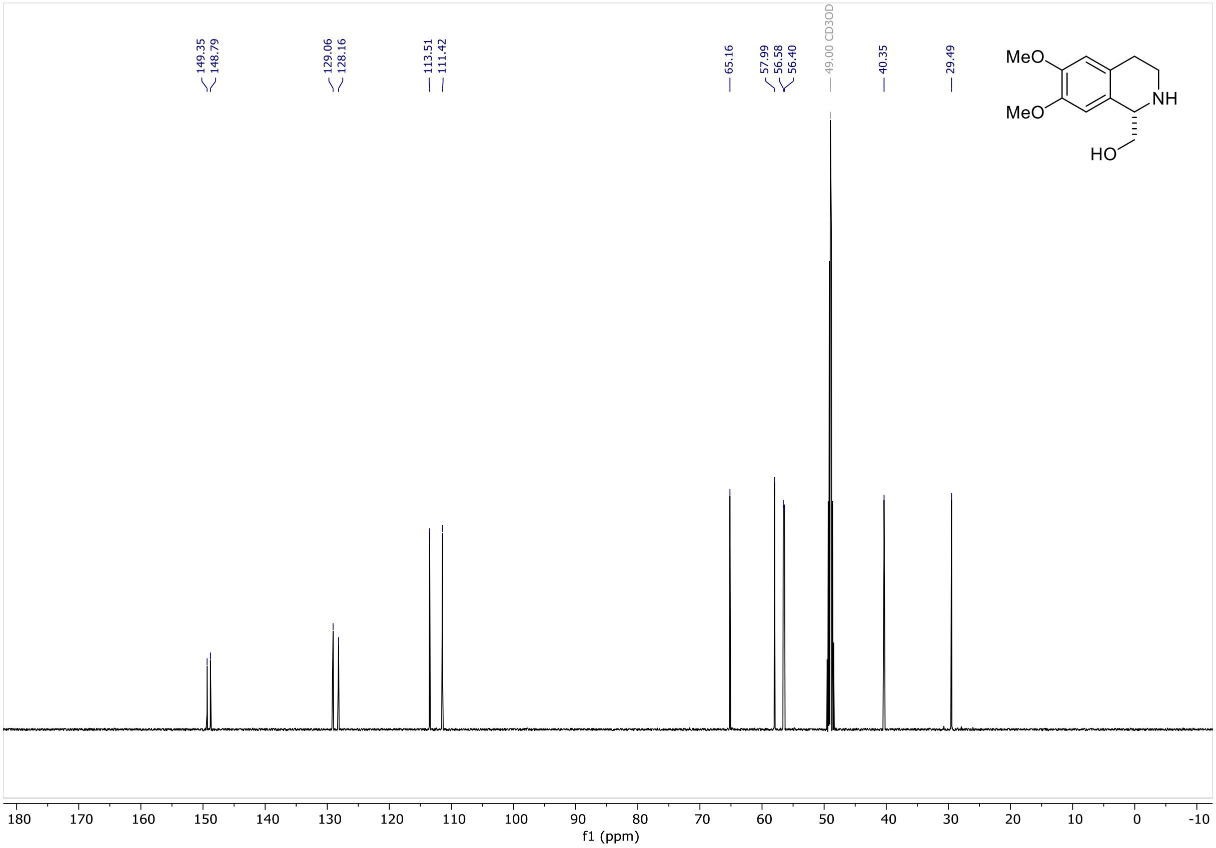

^{13}C-NMR spectrum of Calycotomine**.**

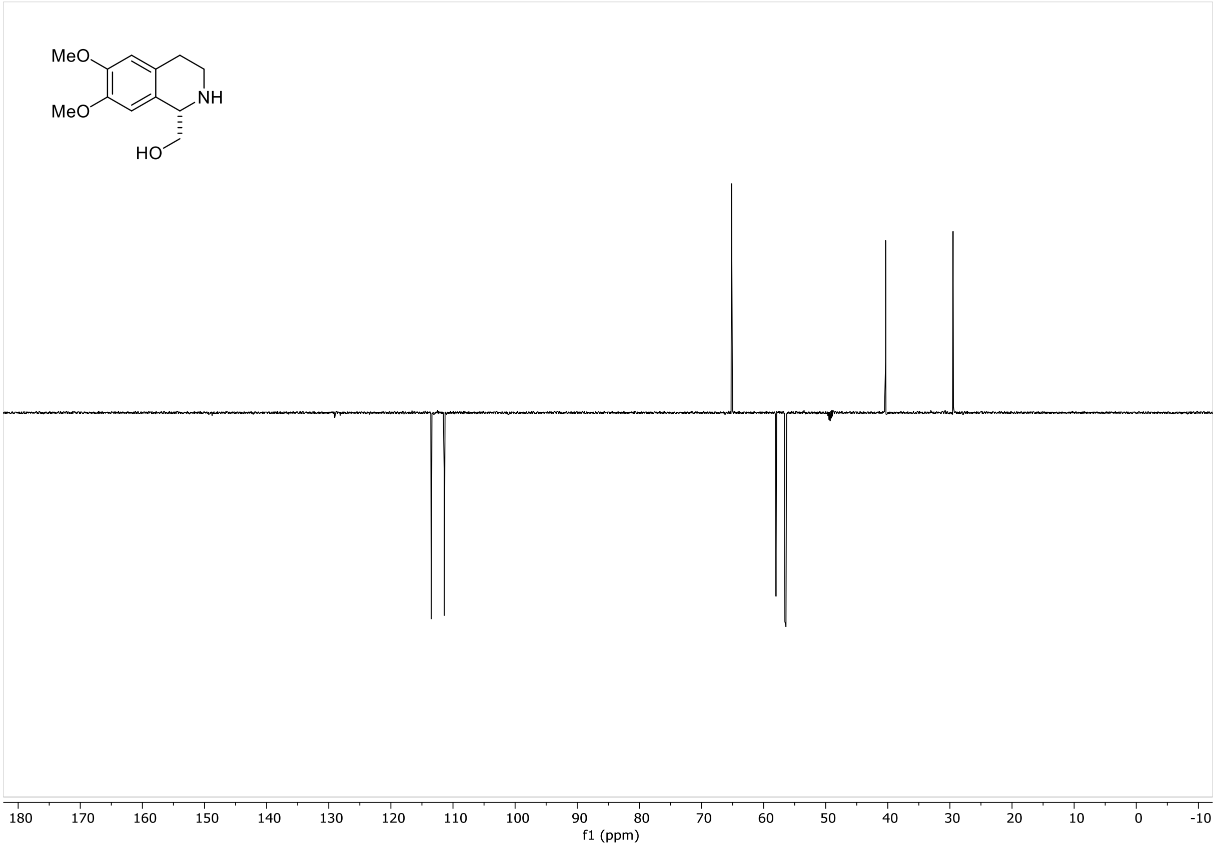

DEP-135 NMR spectrum of Calycotomine.

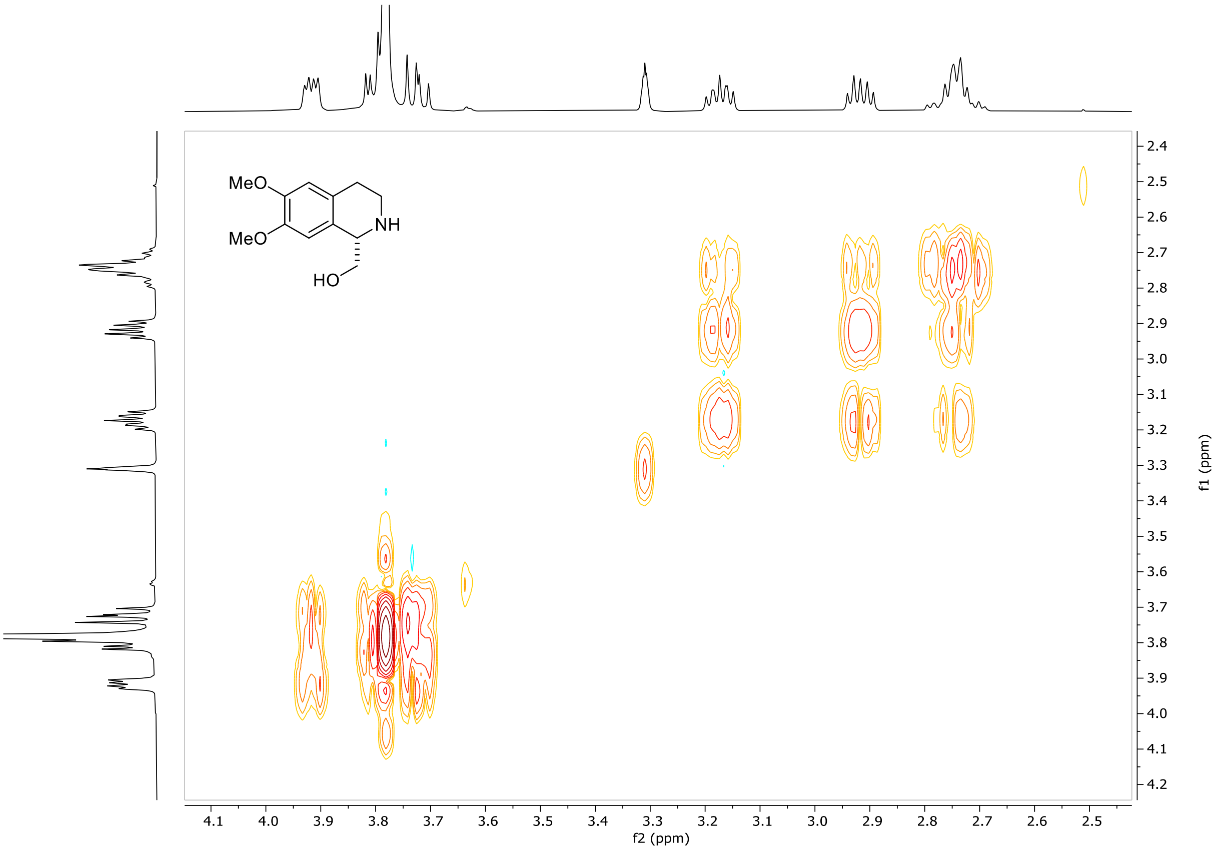

^{1}H-COSY NMR spectrum of Calycotomine.

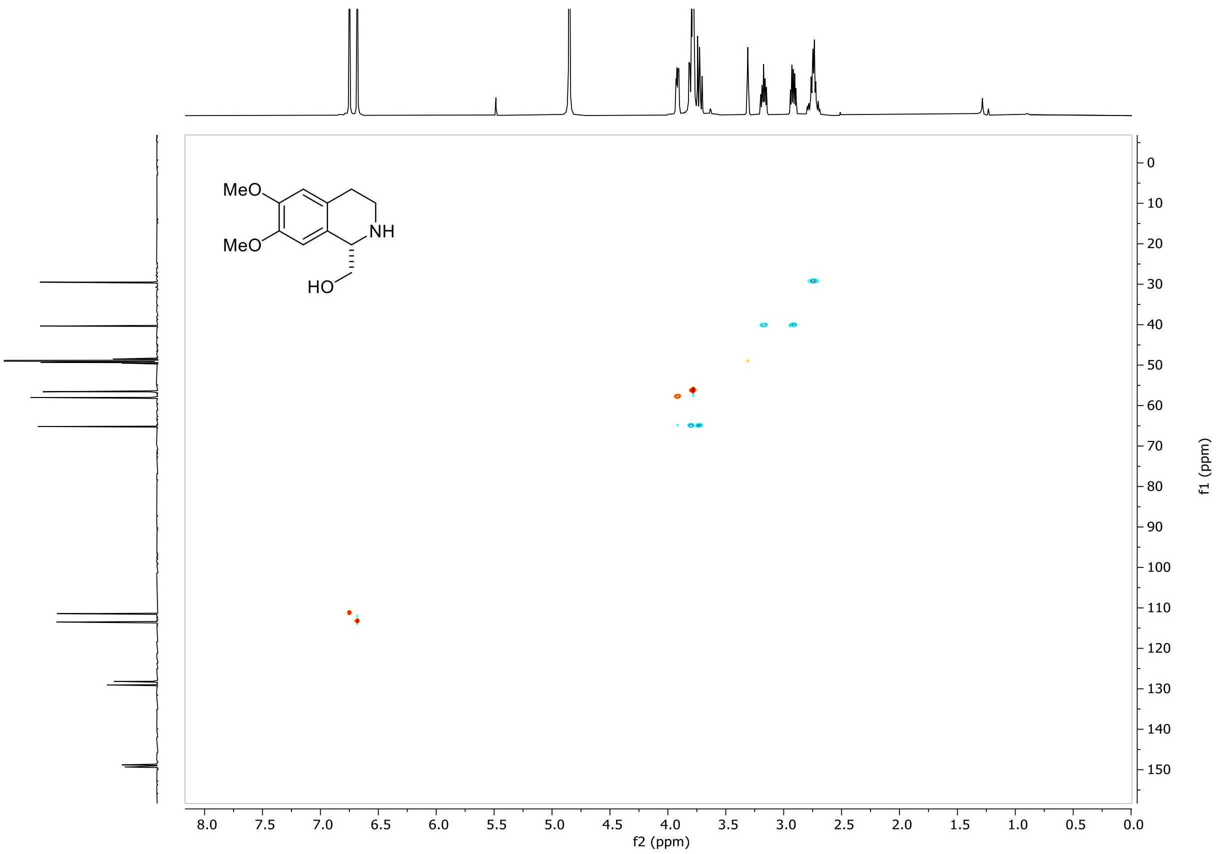

^{1}H-^{13}C-HSQC NMR spectrum of Calycotomine.

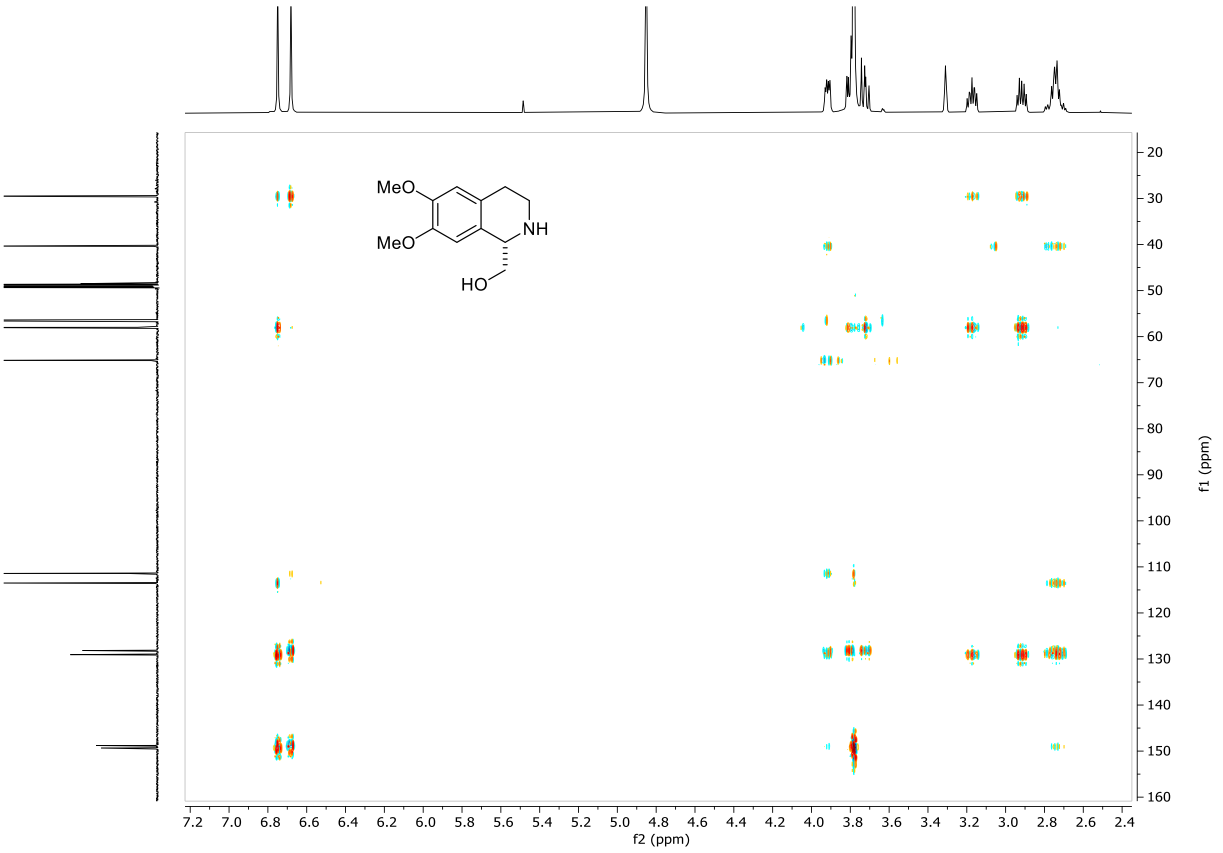

^{1}H-^{13}C-HMBC NMR spectrum of Calycotomine.

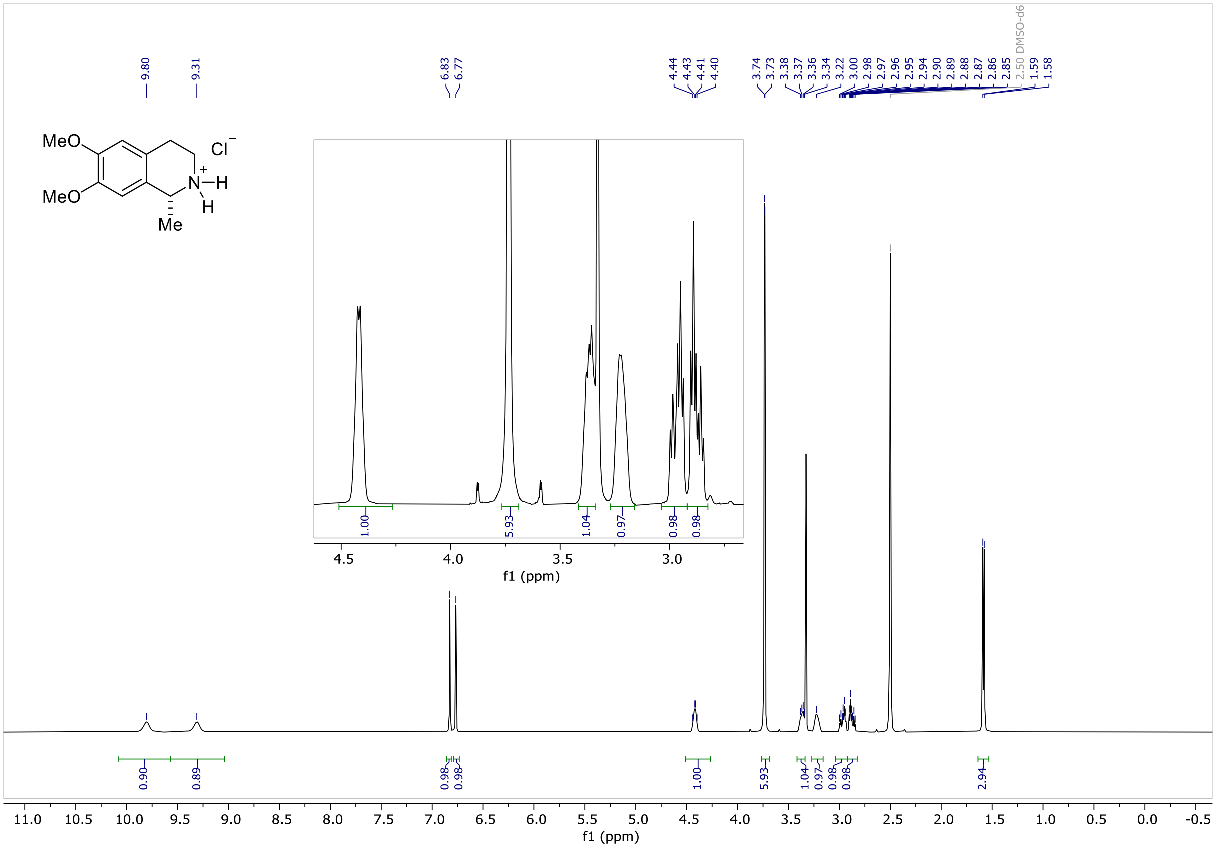

^{1}H-NMR spectrum of salsolidine hydrochloride.

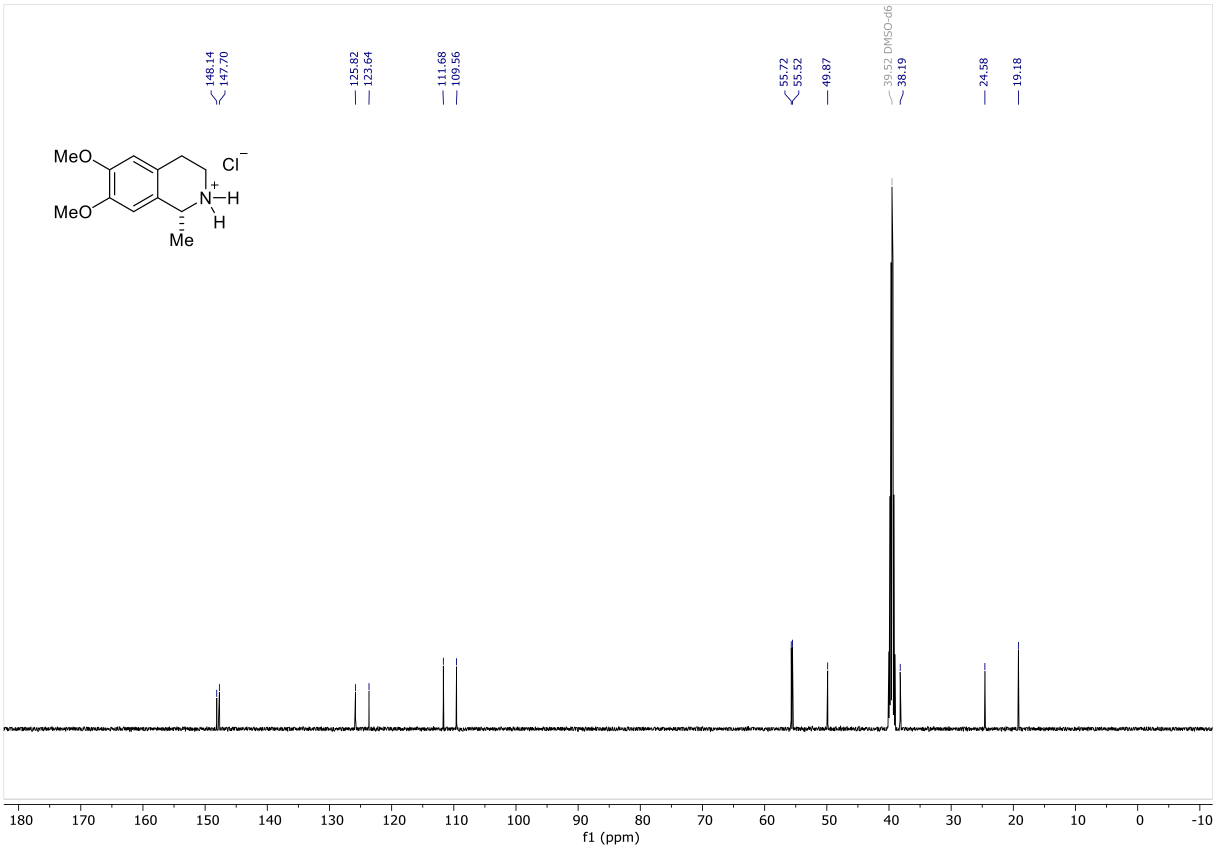

^{13}C-NMR spectrum of salsolidine hydrochloride.

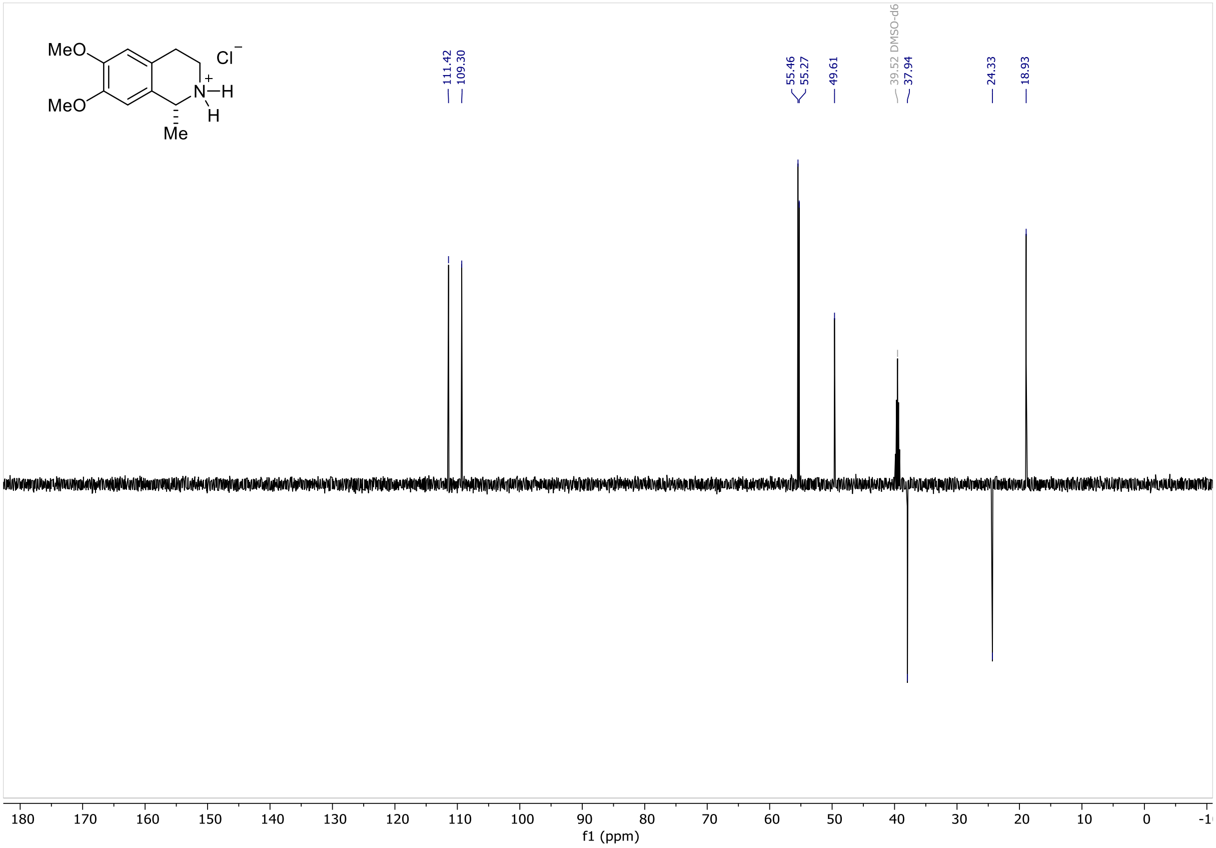

DEPT-135 NMR-spectrum of salsolidine hydrochloride.

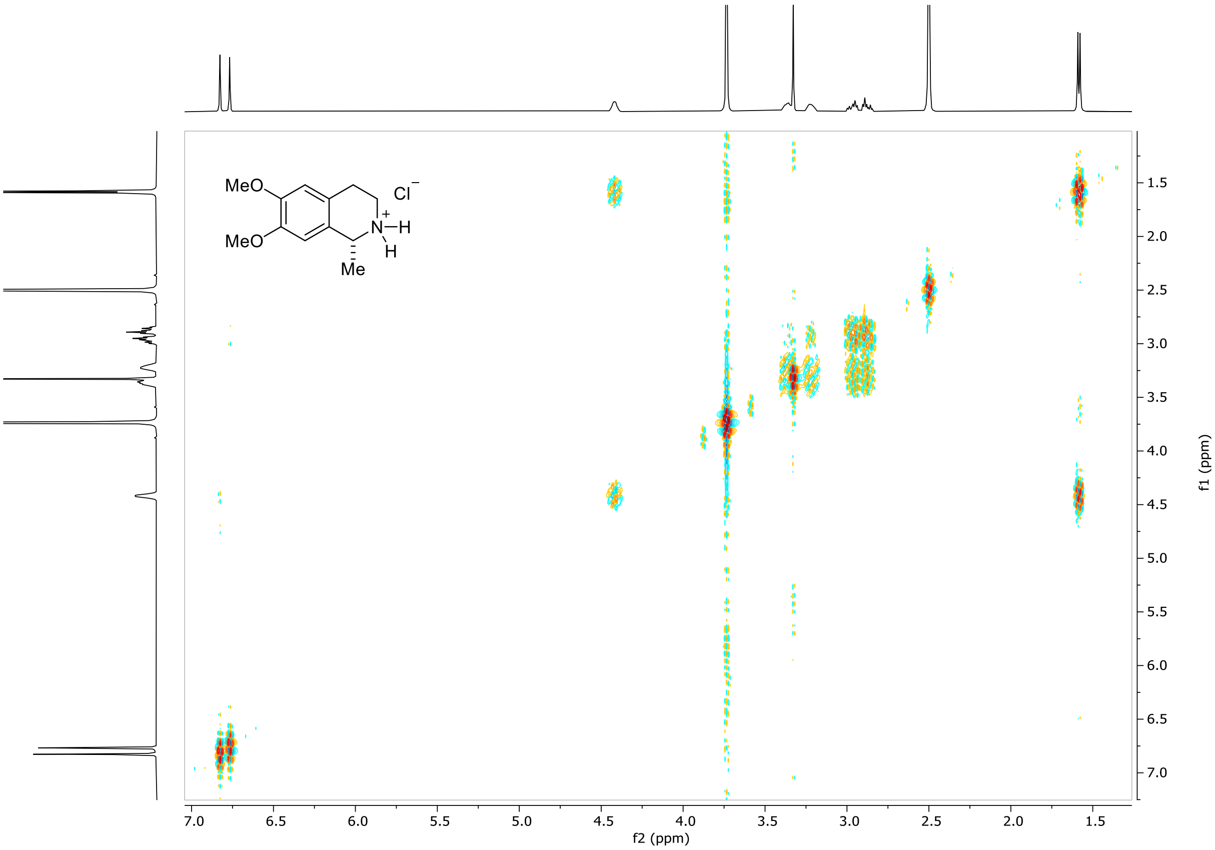

[1]**H-COSY** NMR-spectrum of salsolidine hydrochloride.

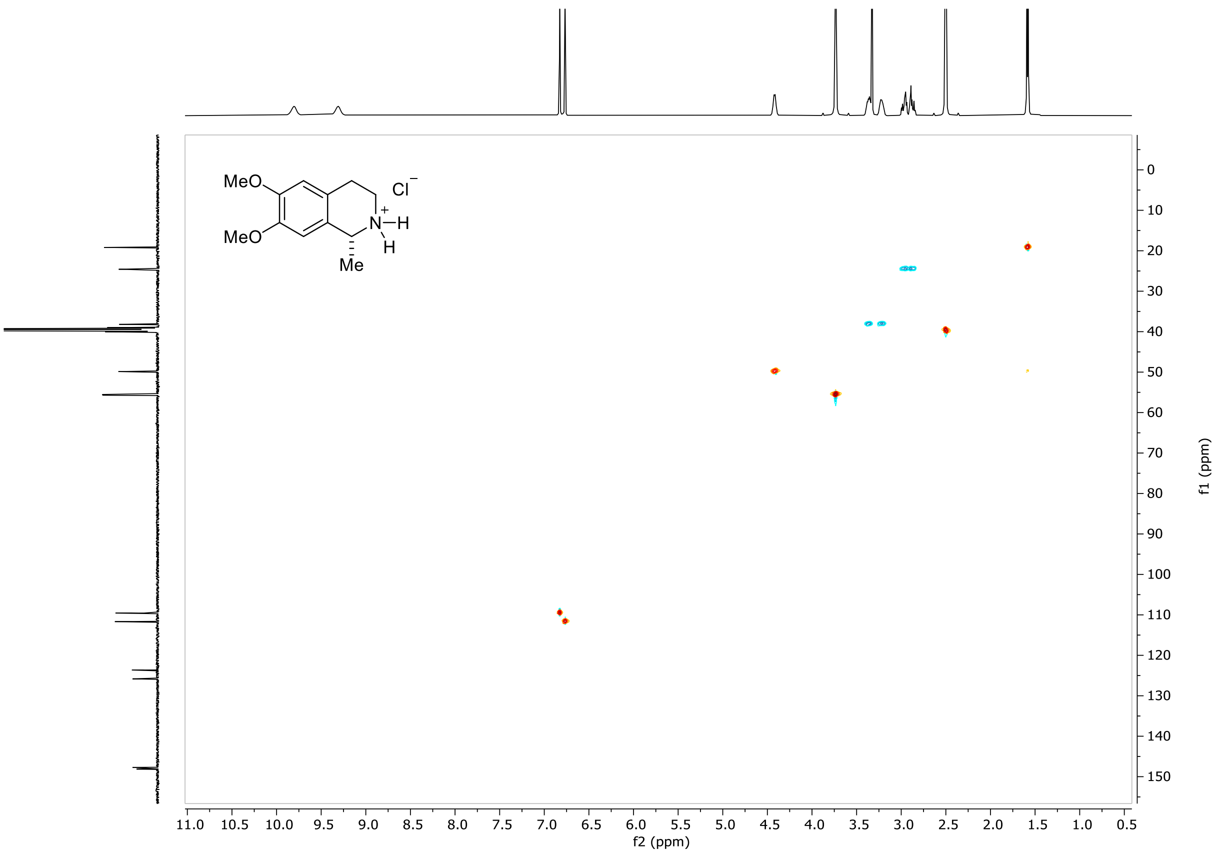

^{1}H-^{13}C-HSQC NMR-spectrum of salsolidine hydrochloride.

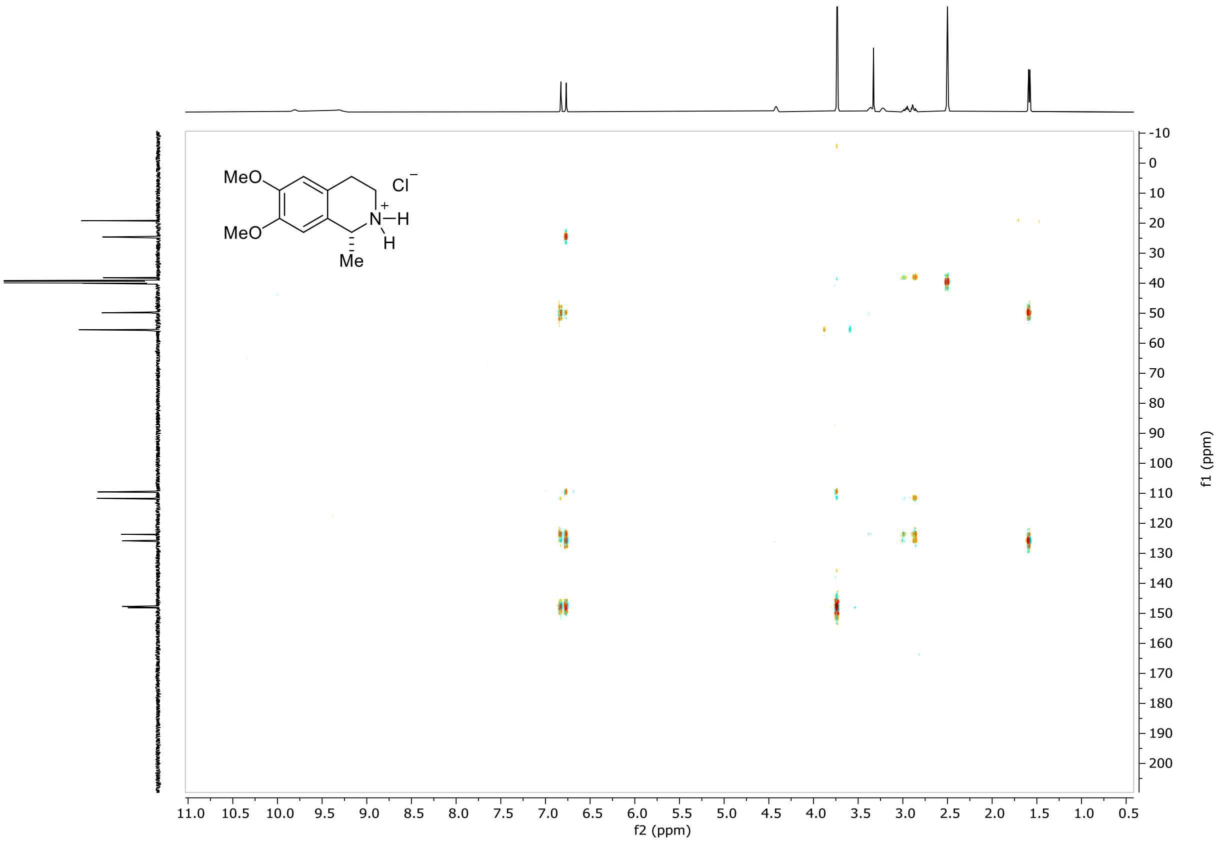

^{1}H-^{13}C-HMBC NMR-spectrum of salsolidine hydrochloride.

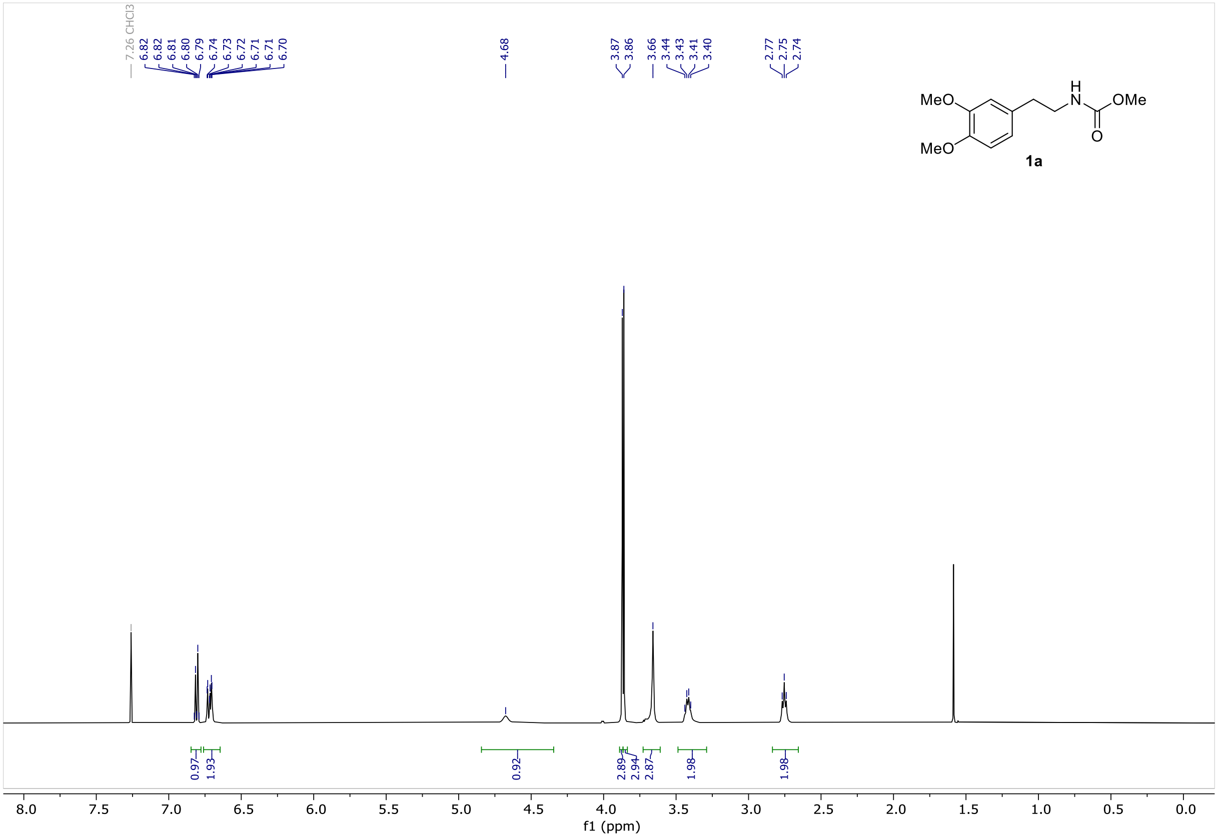

^{1}H-NMR spectrum of compound **1a.**

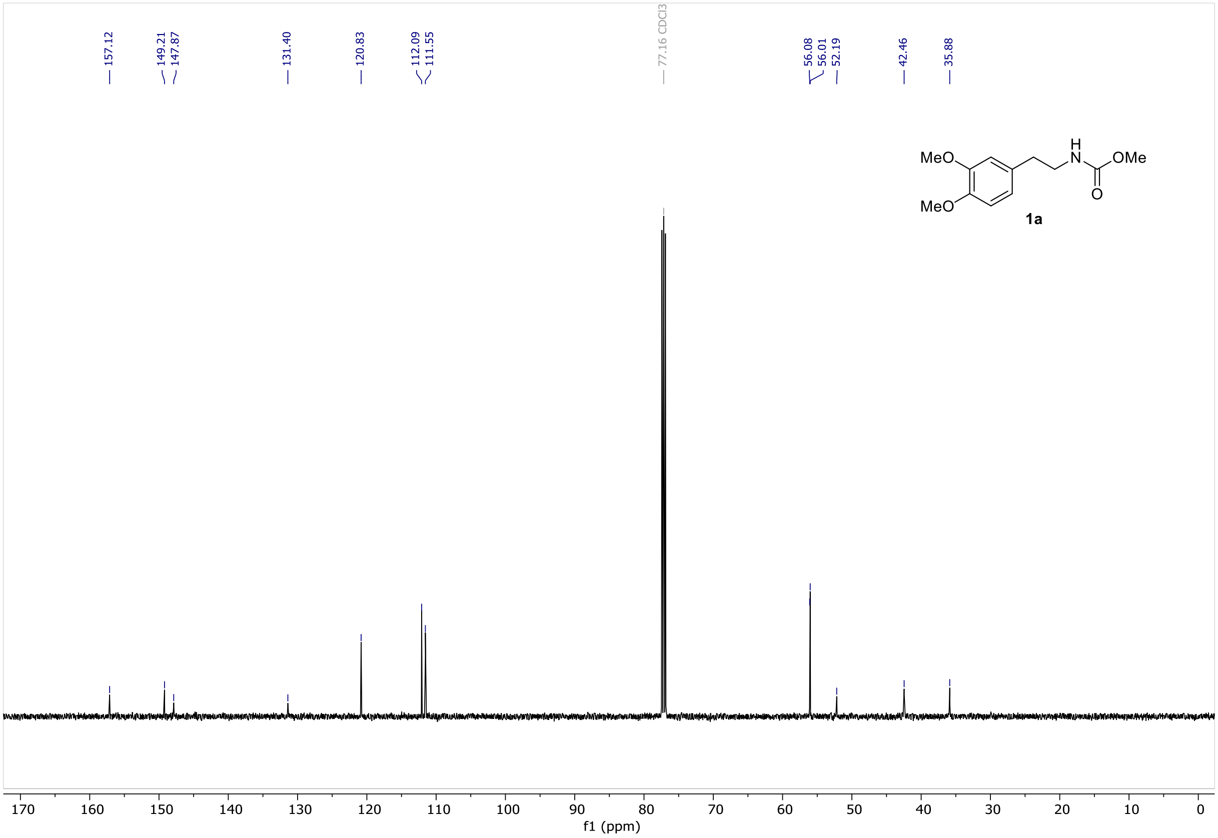

^{13}C-NMR spectrum of compound **1a.**

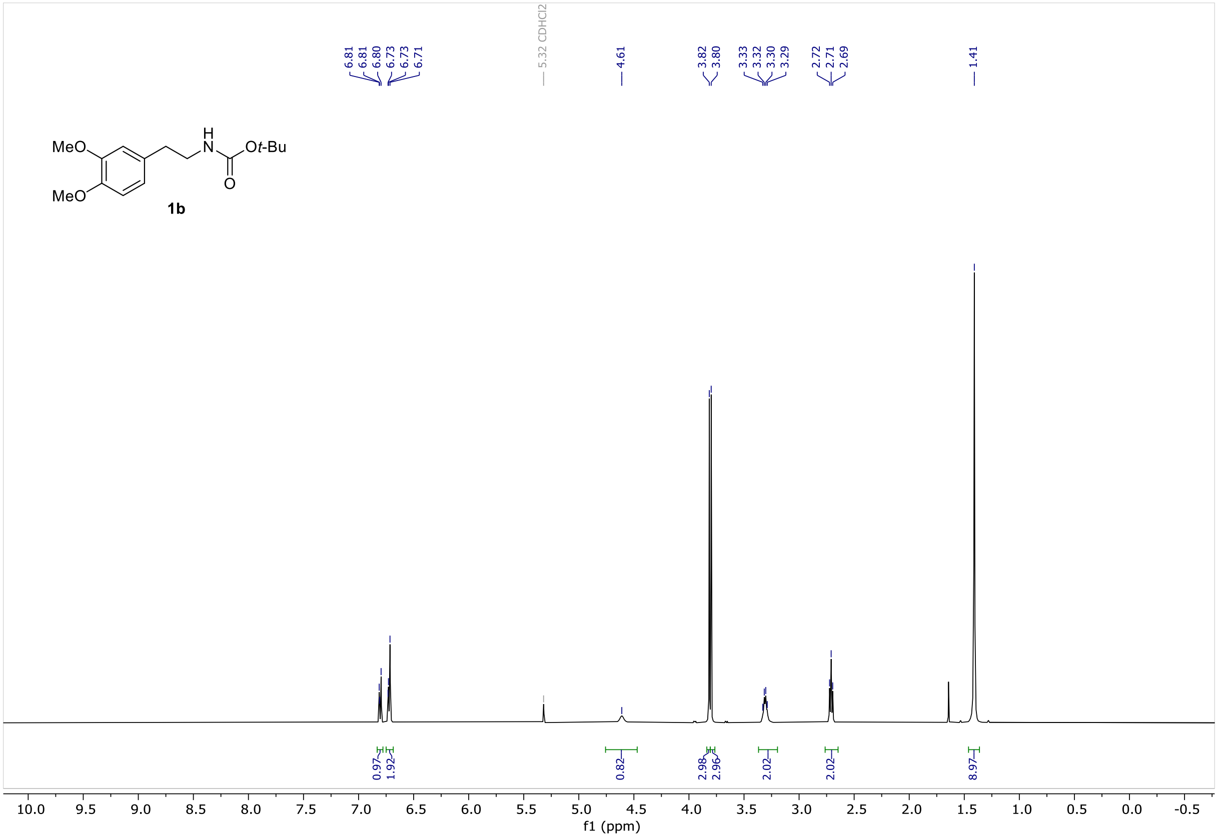

^{1}H-NMR spectrum of compound **1b.**

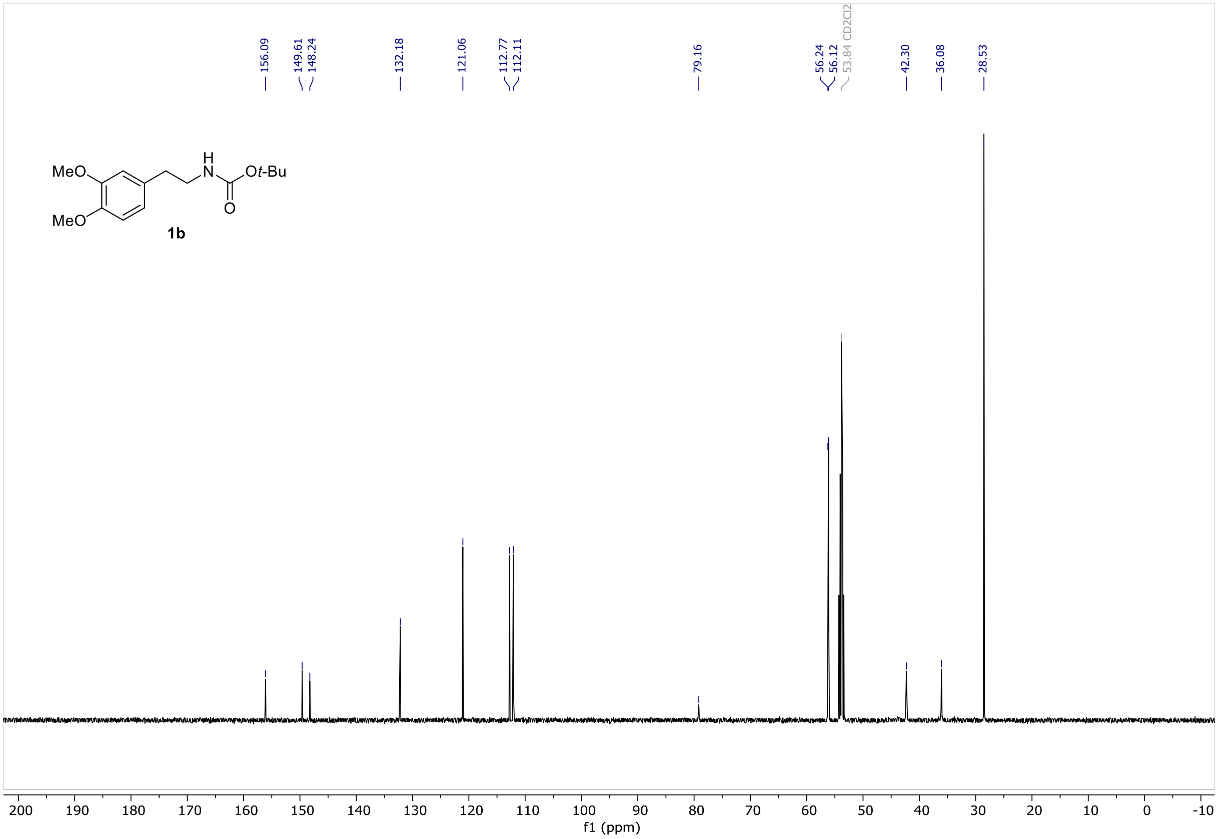

^{13}C-NMR spectrum of compound **1b.**

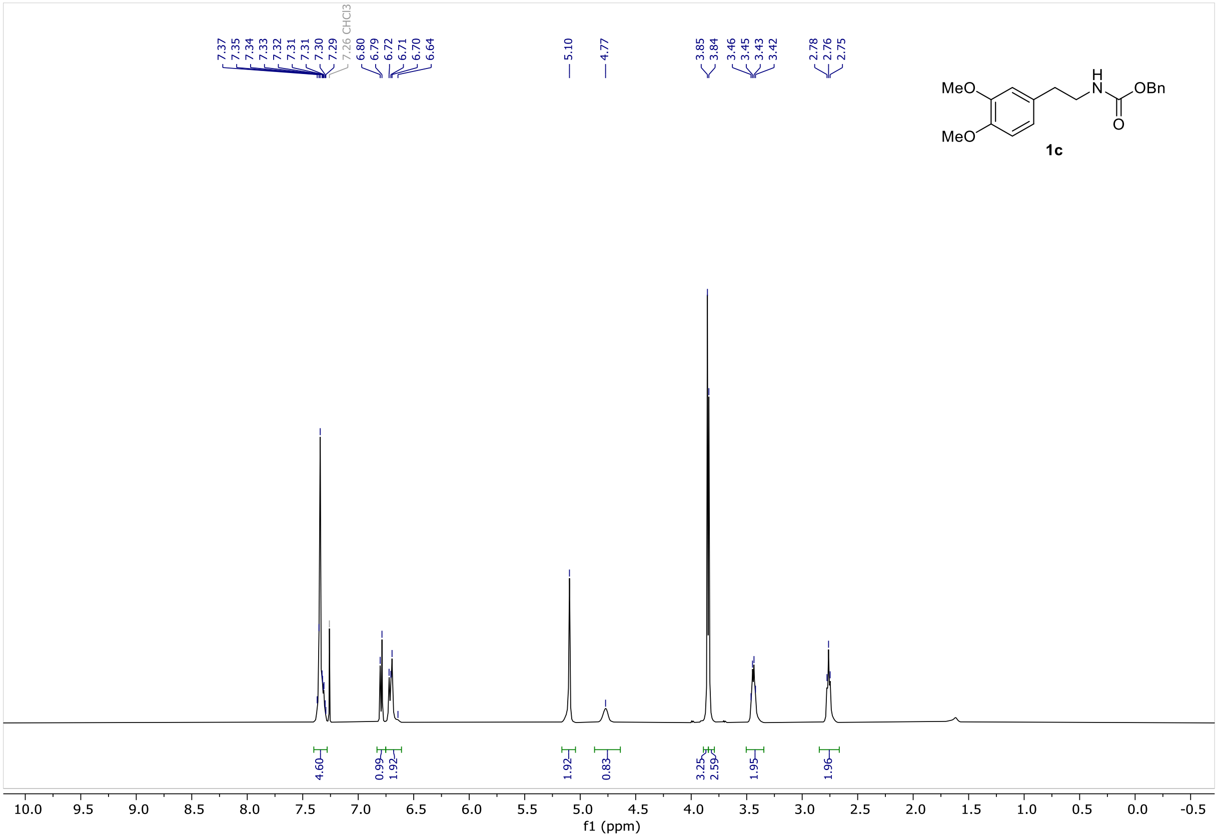

¹H-NMR spectrum of compound **1b.**

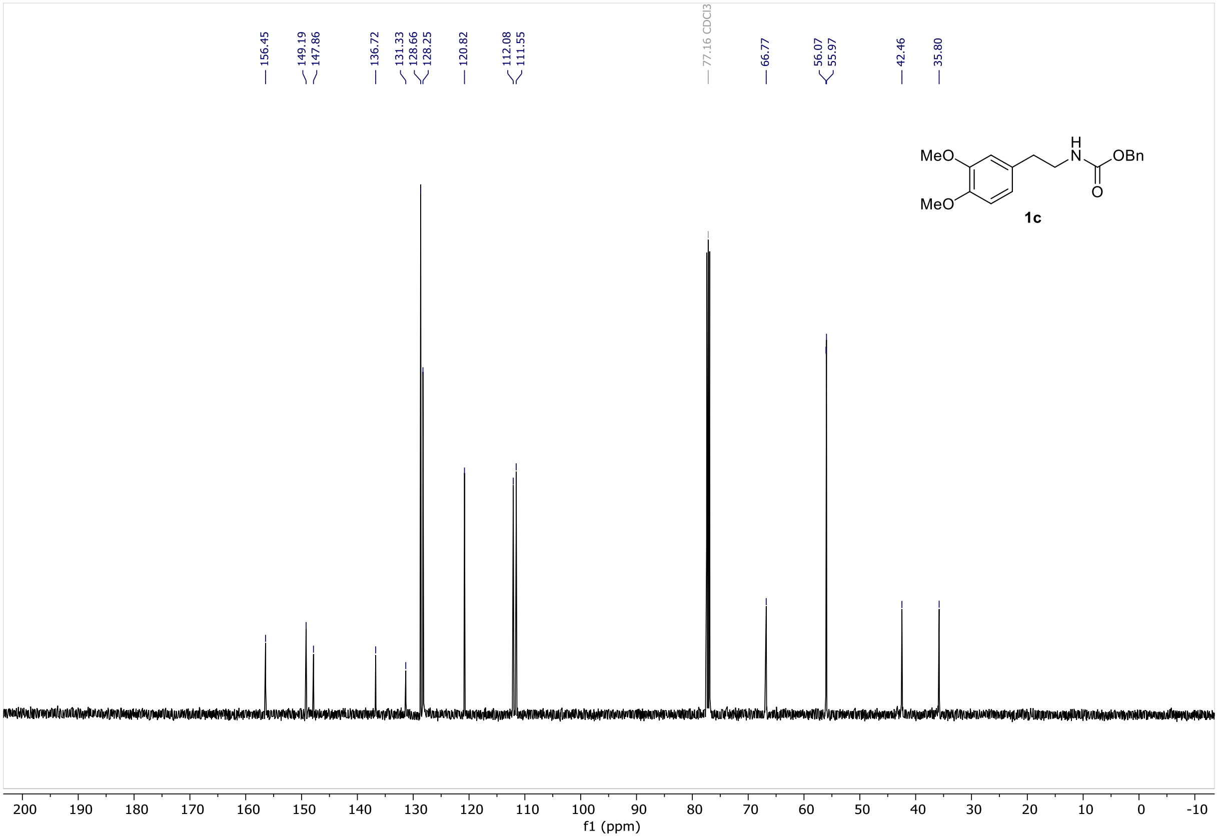

¹³C-NMR spectrum of compound **1b.**

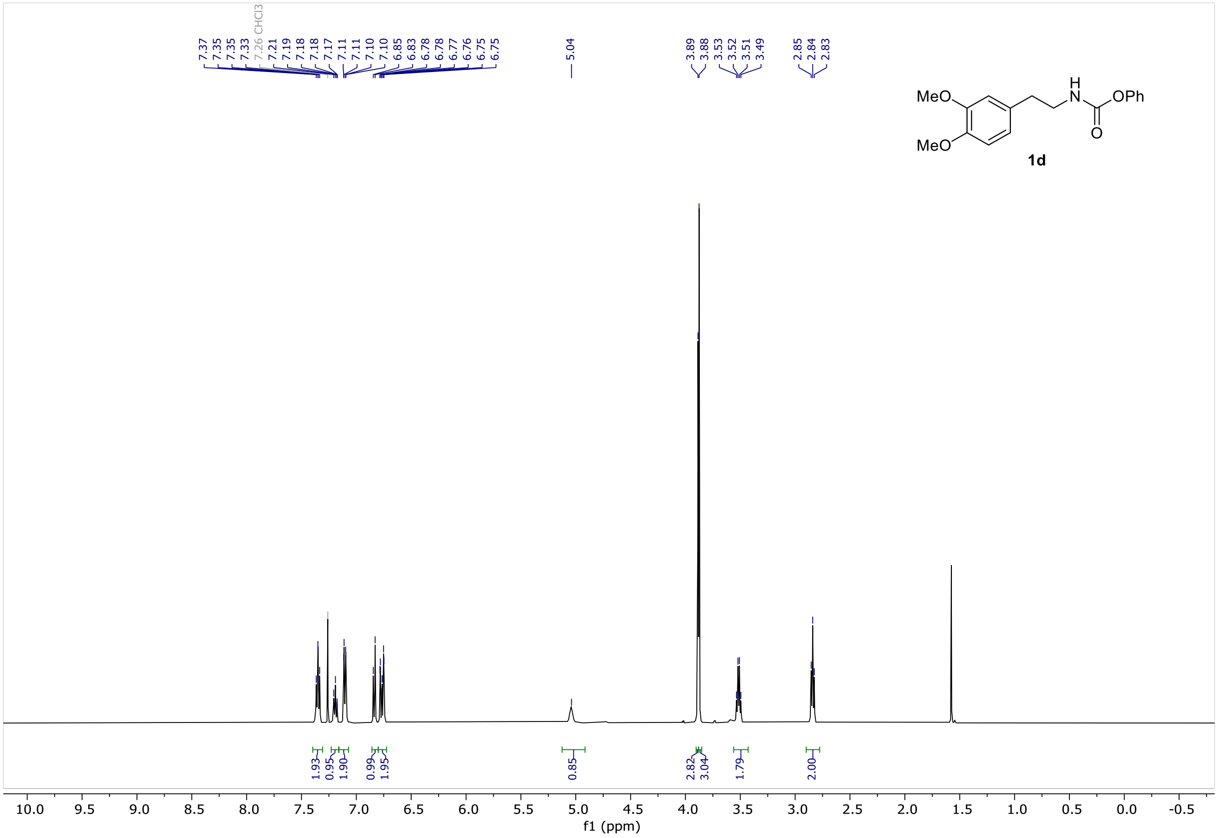

^{1}H-NMR spectrum of compound **1d.**

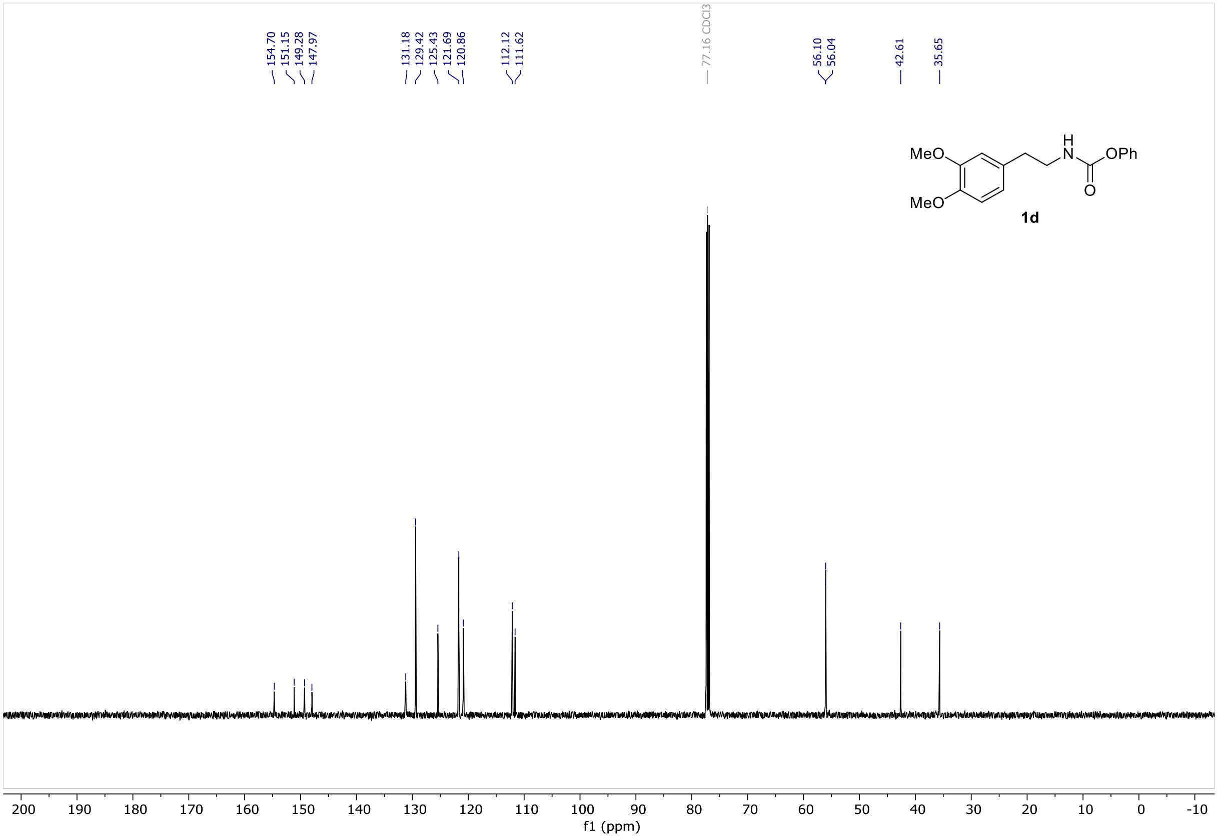

^{13}C-NMR spectrum of compound **1d.**

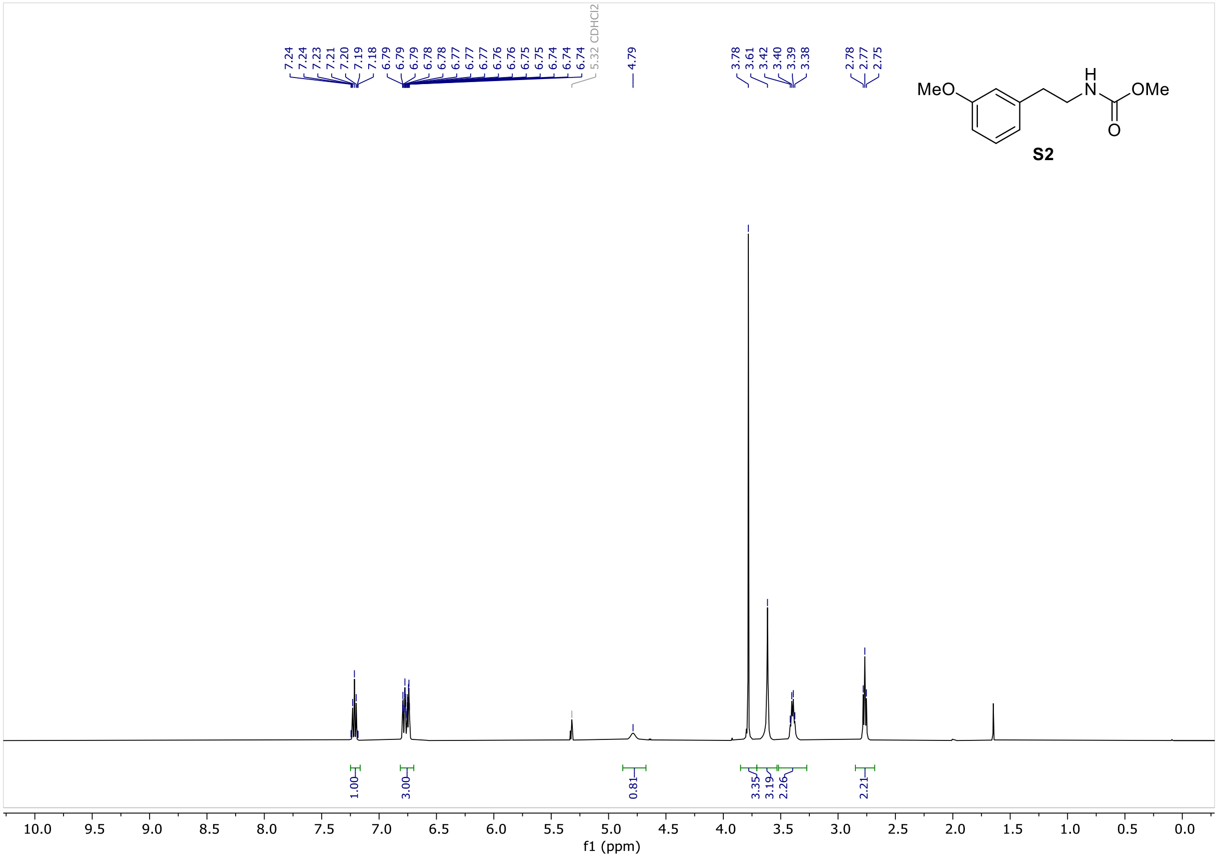

^{1}H-NMR spectrum of compound **S2.**

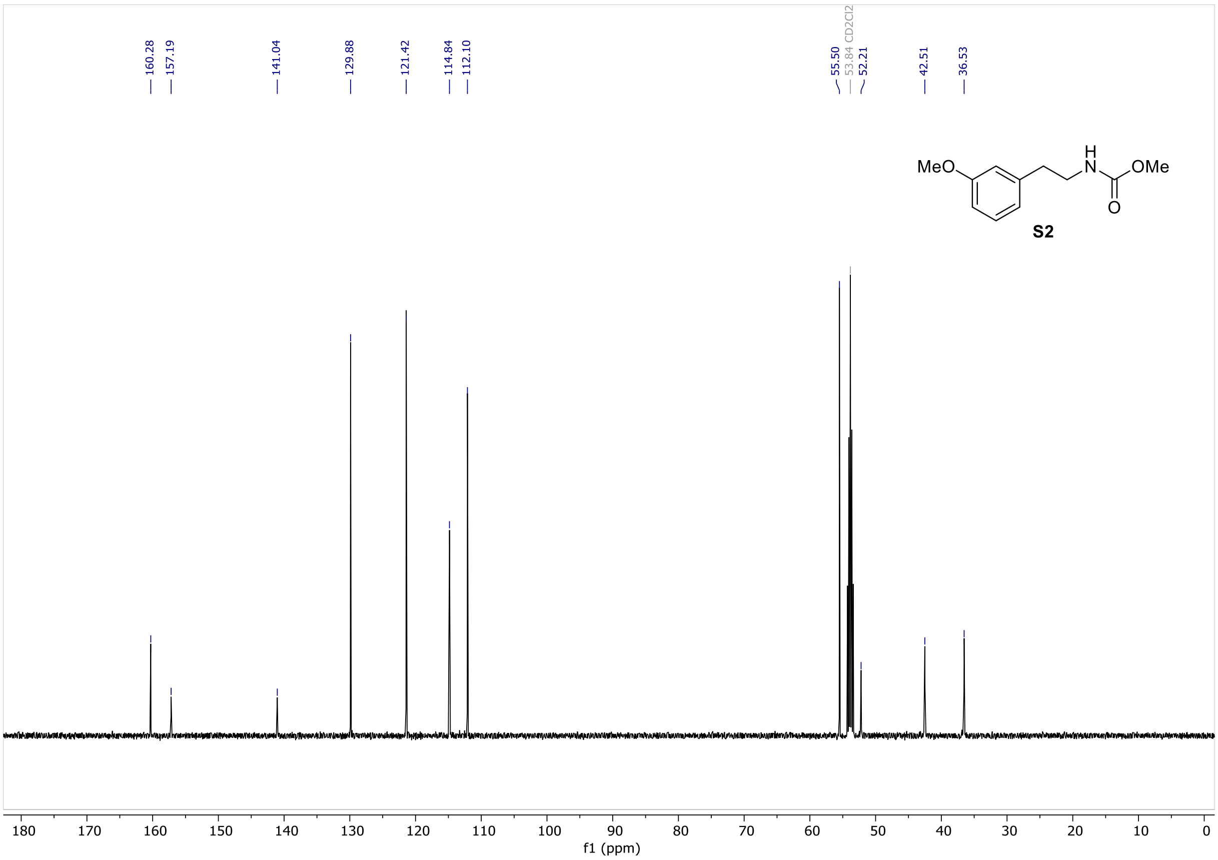

^{13}C-NMR spectrum of compound **S2.**

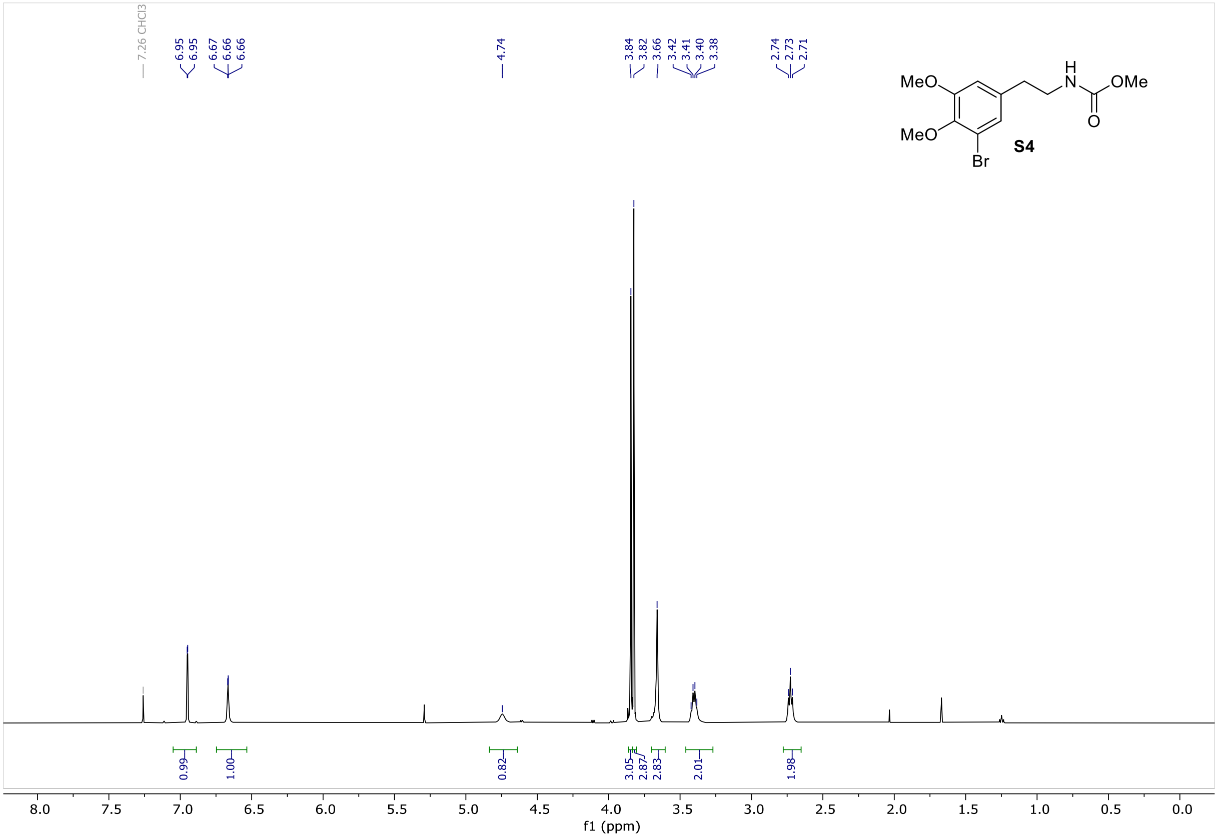

[1]**H-NMR** spectrum of compound **S4.**

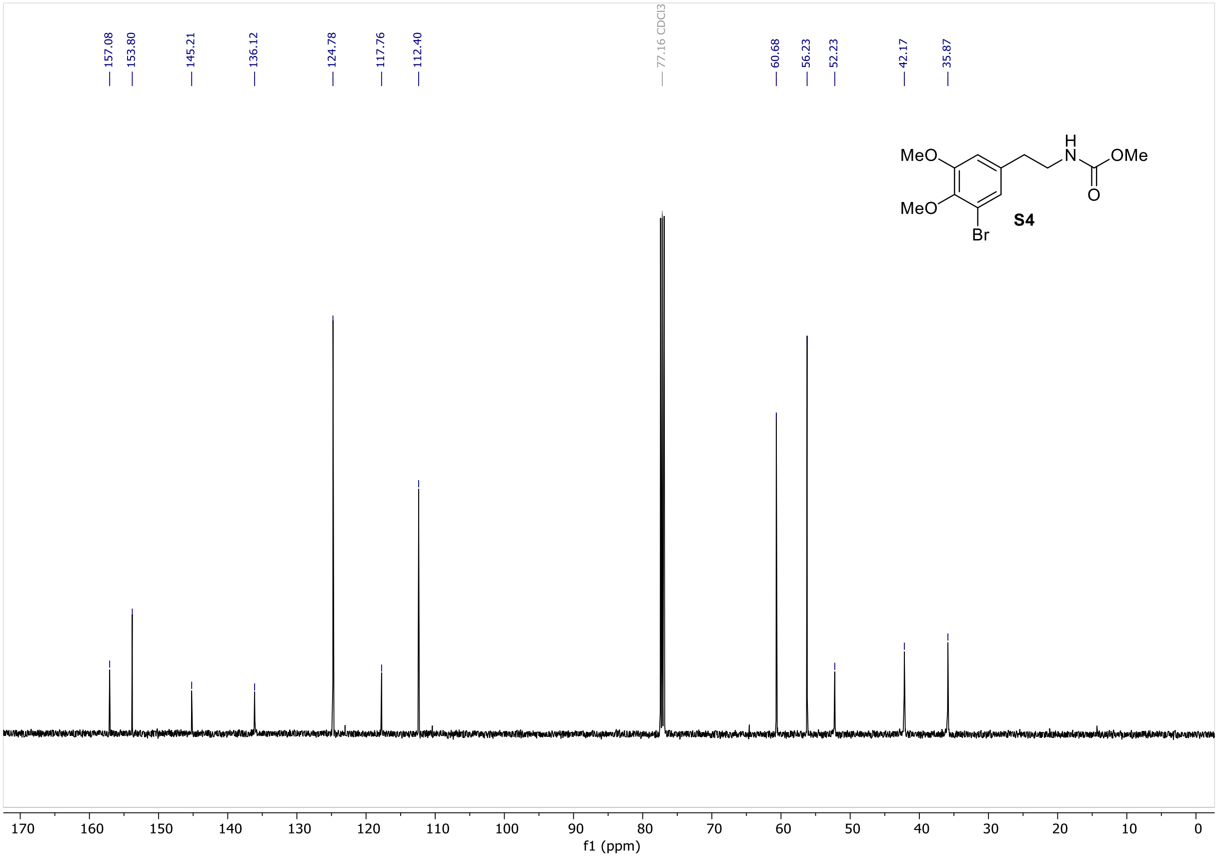

[13]**C-NMR** spectrum of compound **S4.**

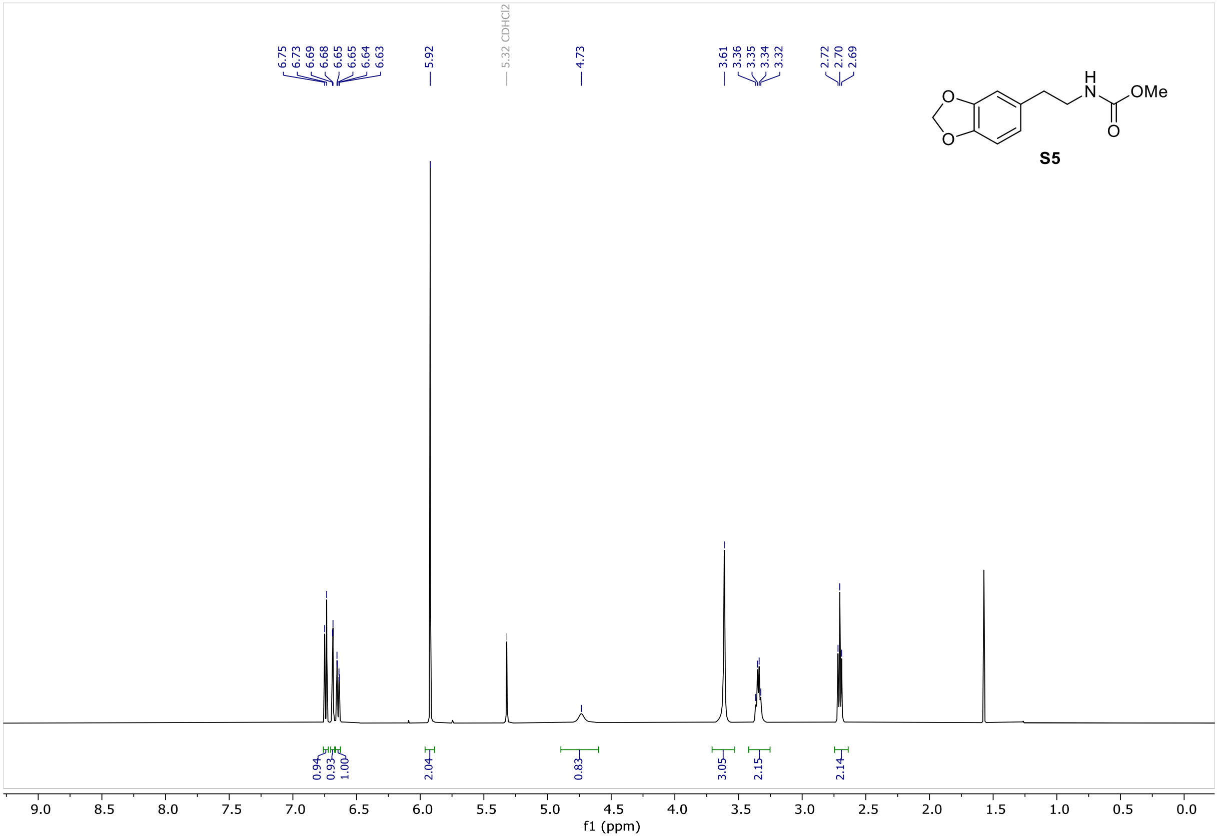

^{1}**H-NMR** spectrum of compound **S5.**

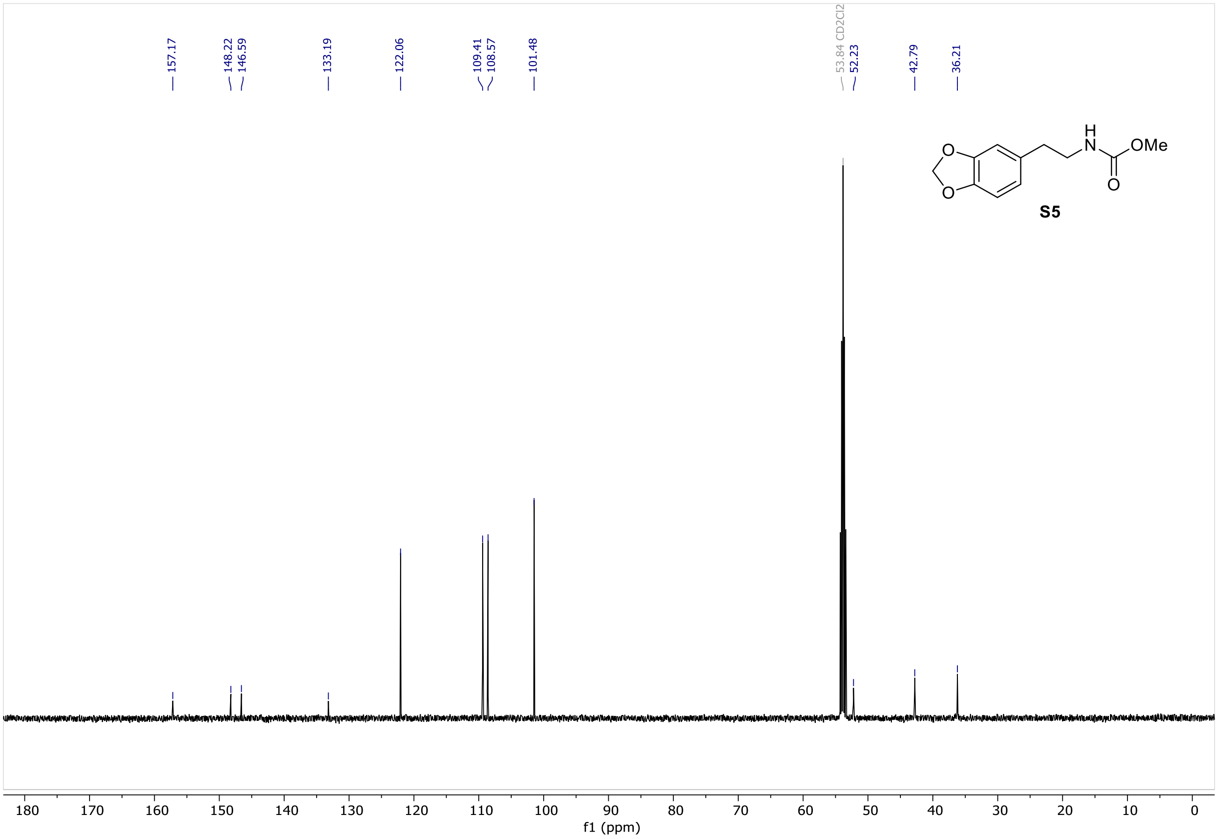

^{13}**C-NMR** spectrum of compound **S5.**

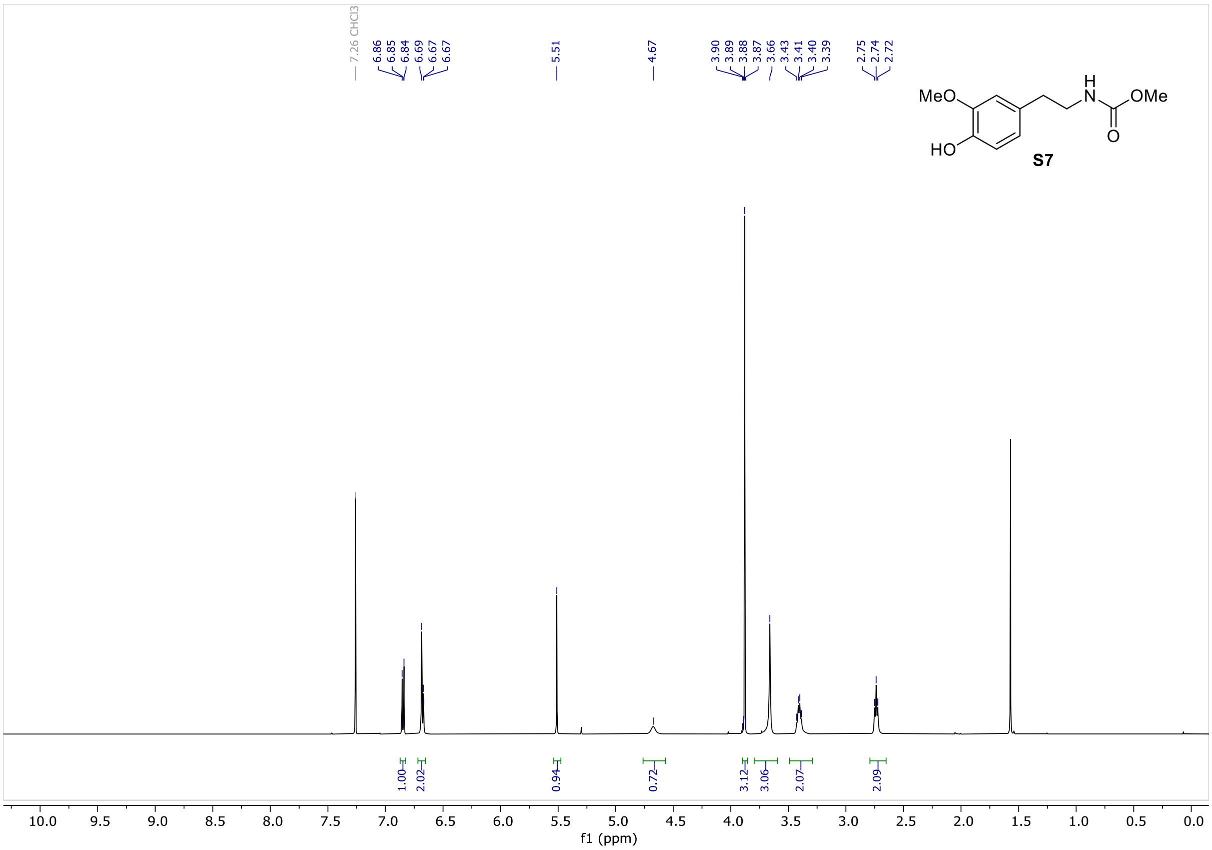

^{1}H-NMR spectrum of compound **S7.**

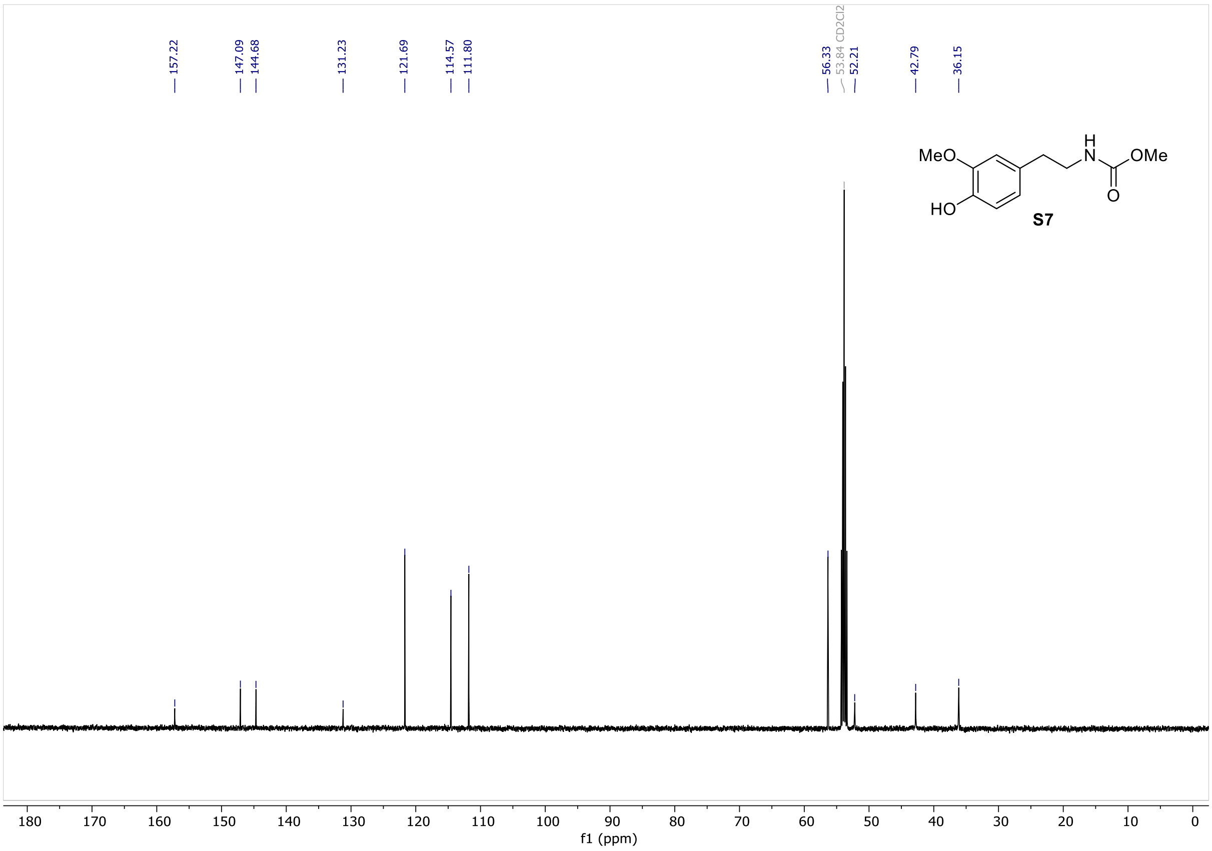

^{13}C-NMR spectrum of compound **S7.**

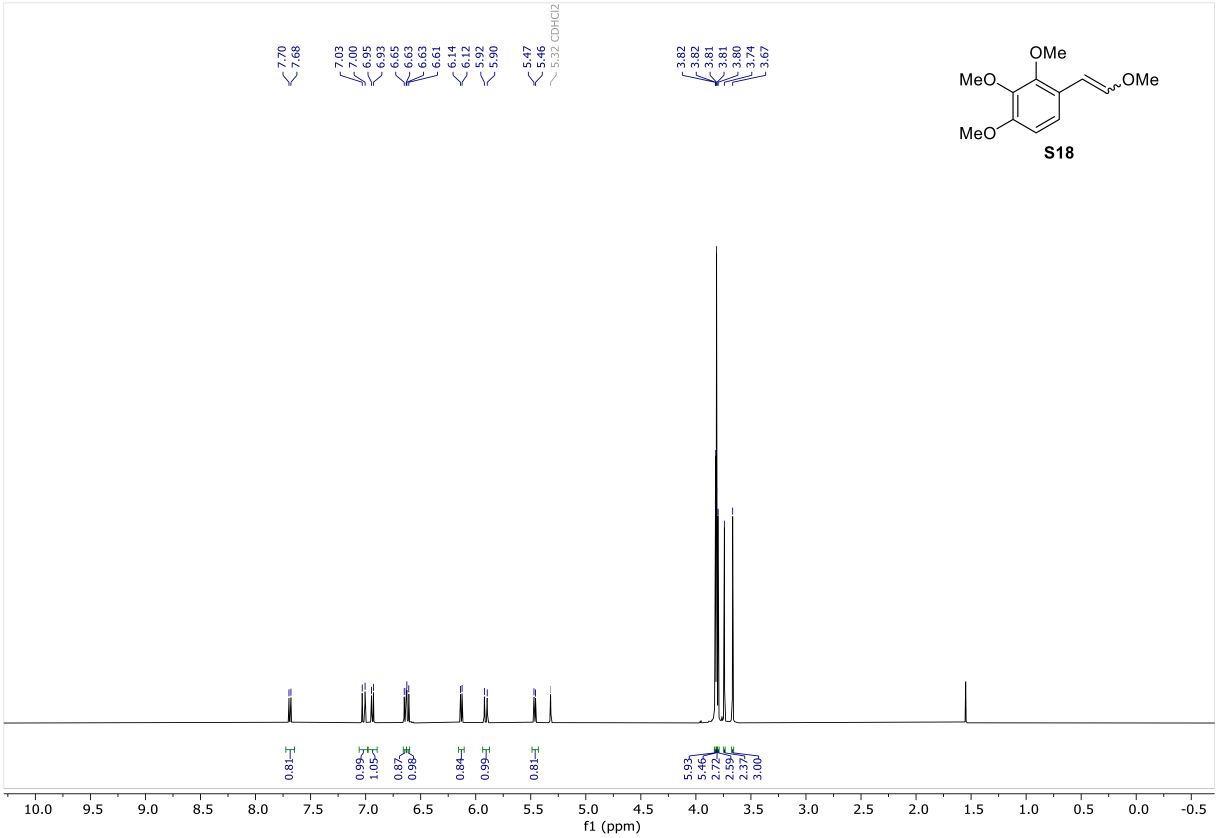

^{1}H-NMR spectrum of compound **S18.**

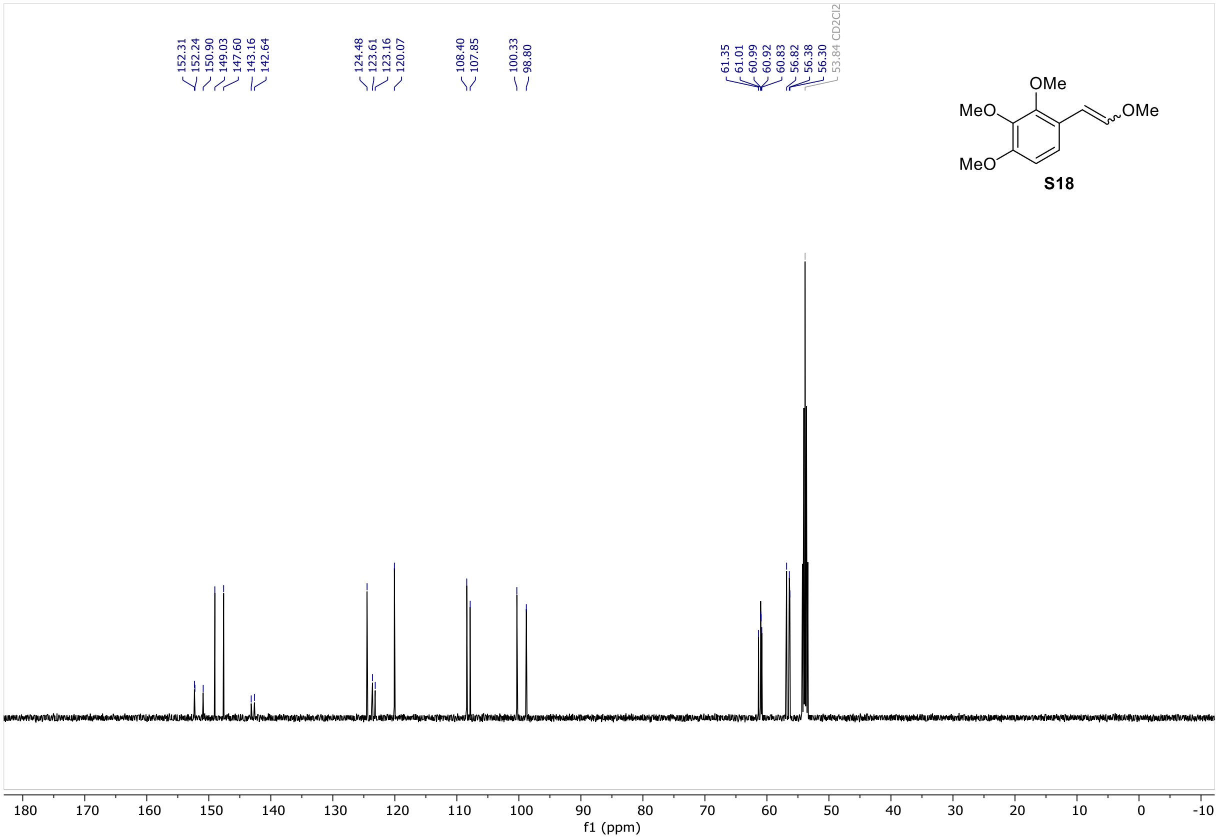

^{13}C-NMR spectrum of compound **S18.**

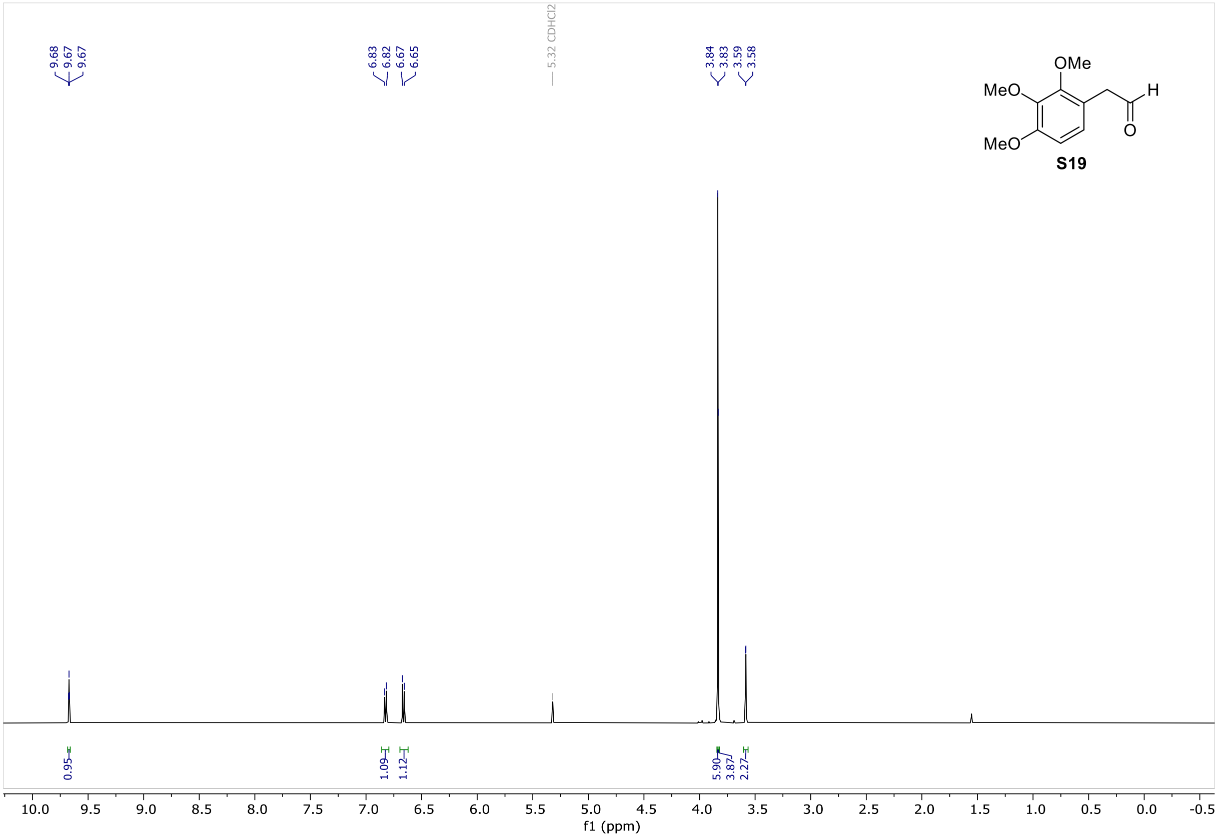

^{1}H-NMR spectrum of compound **S19.**

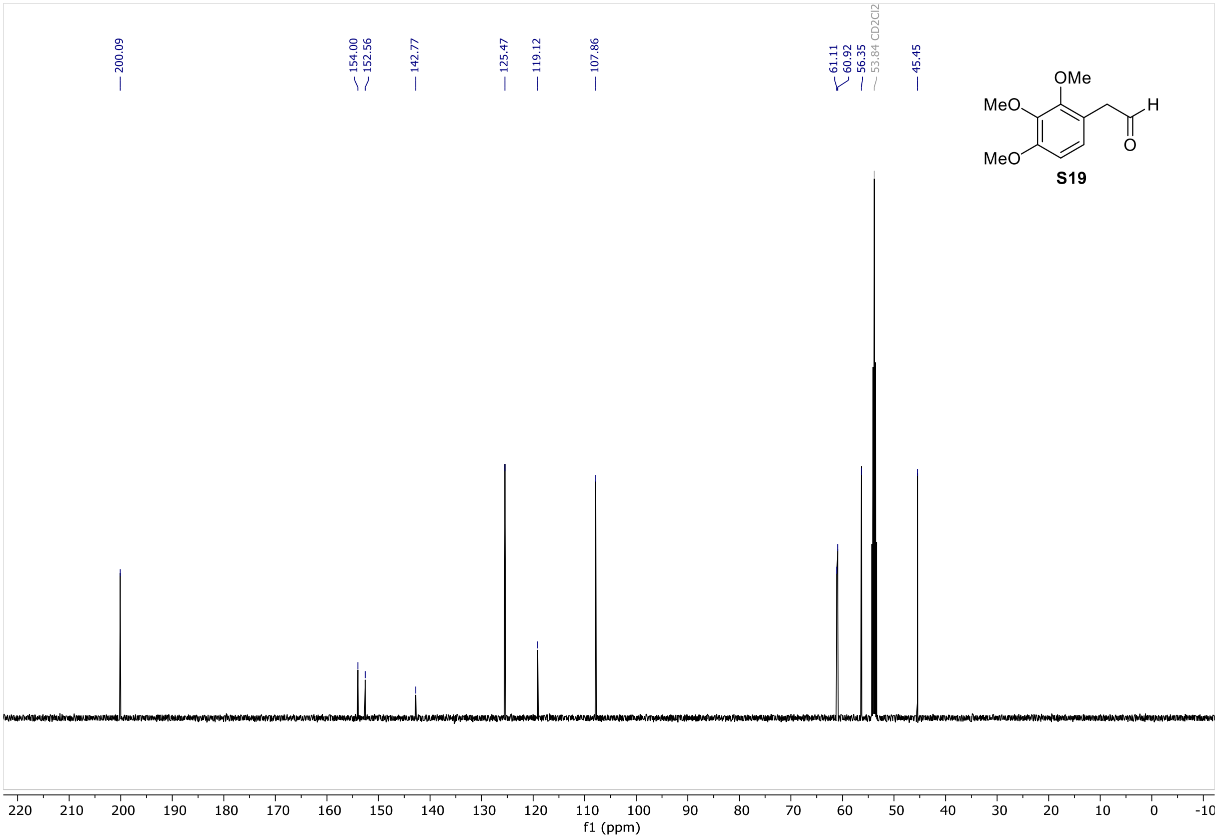

^{13}C-NMR spectrum of compound **S19.**

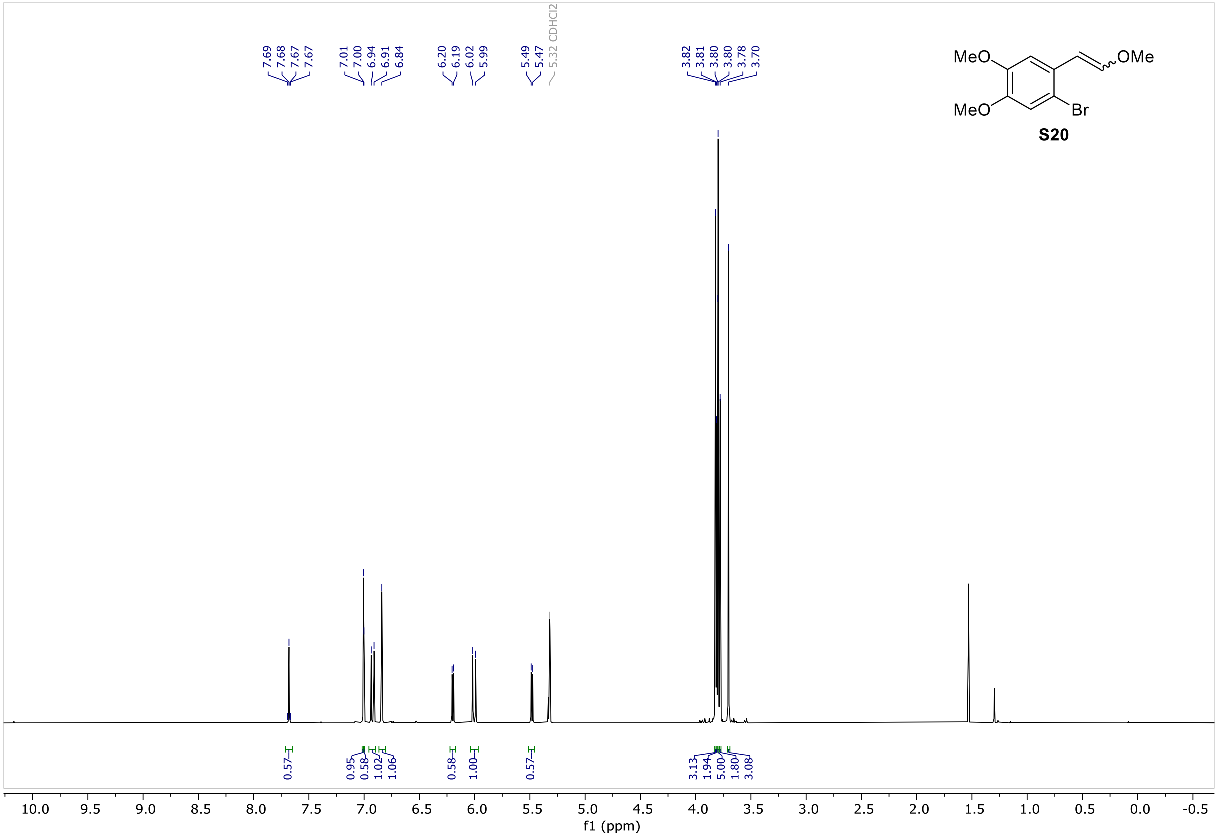

^{1}H-NMR spectrum of compound **S20.**

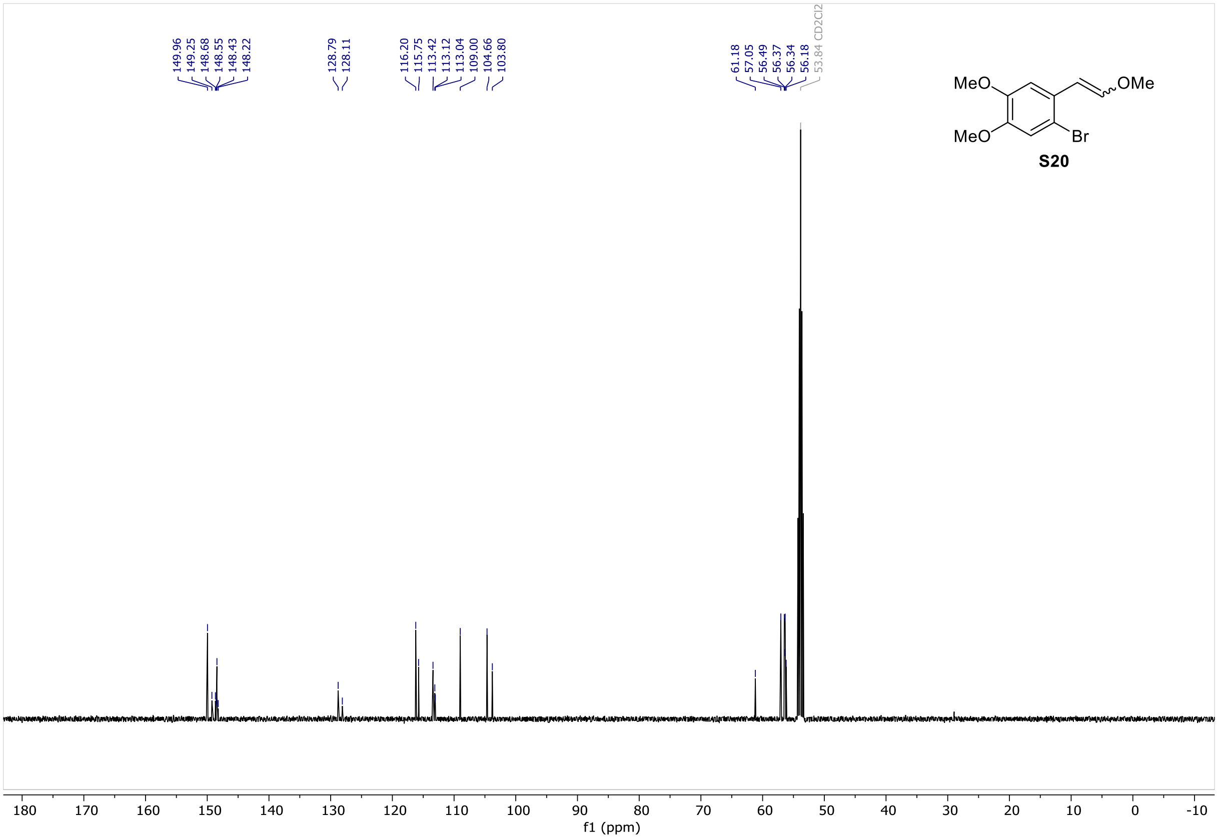

^{13}C-NMR spectrum of compound **S20.**

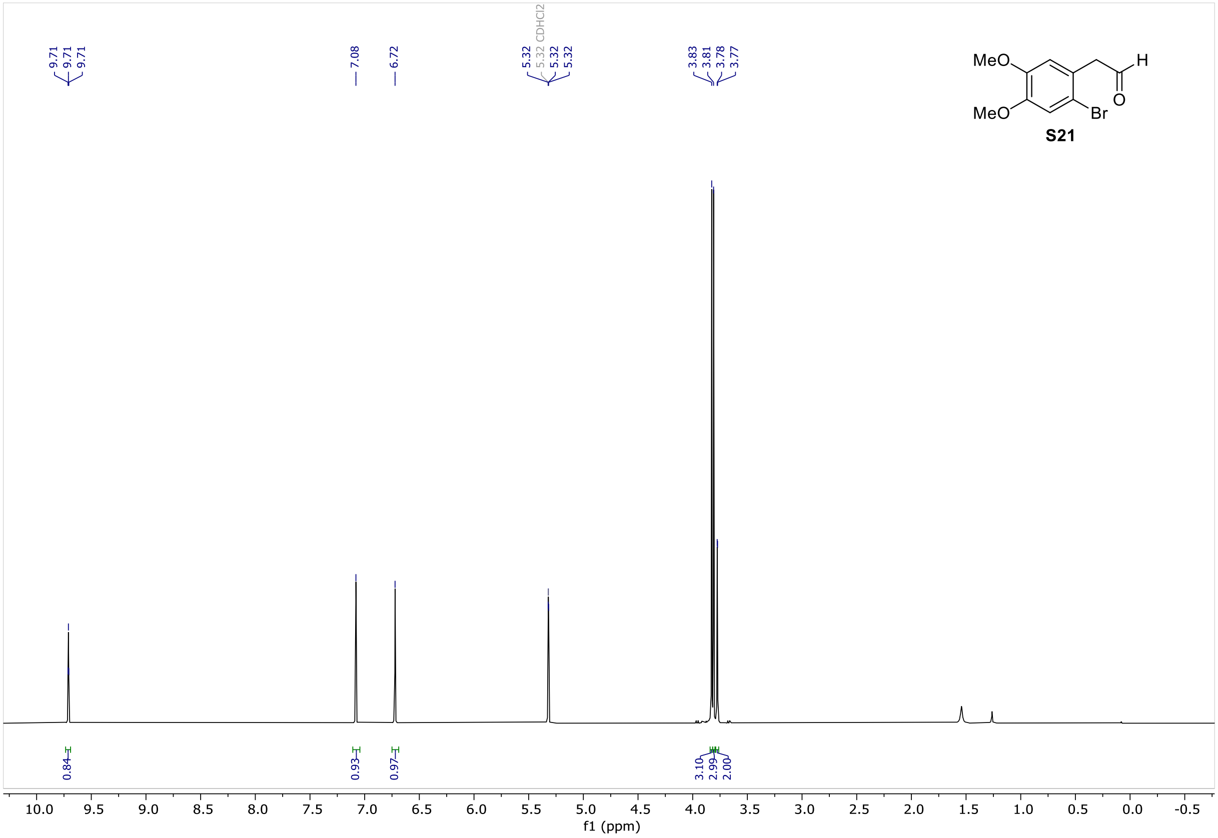

^{1}H-NMR spectrum of compound **S21.**

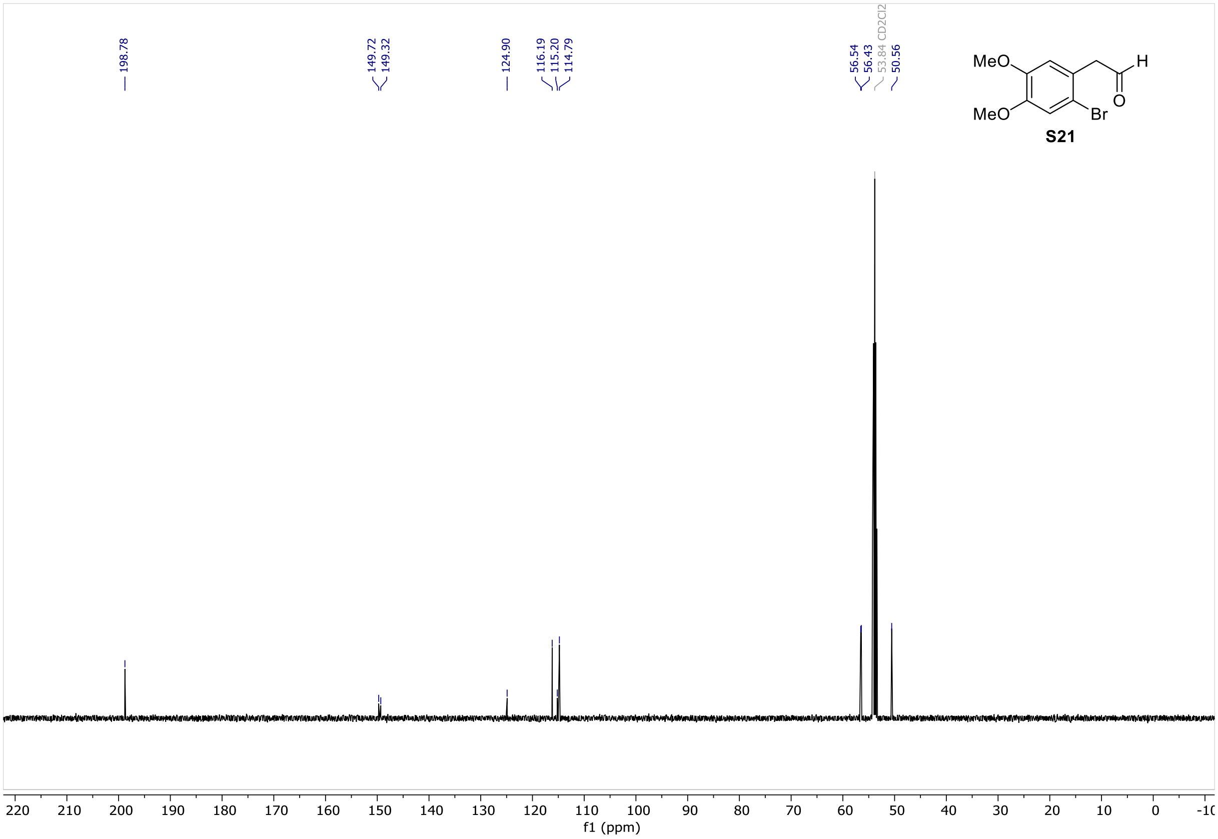

^{13}C-NMR spectrum of compound **S21.**

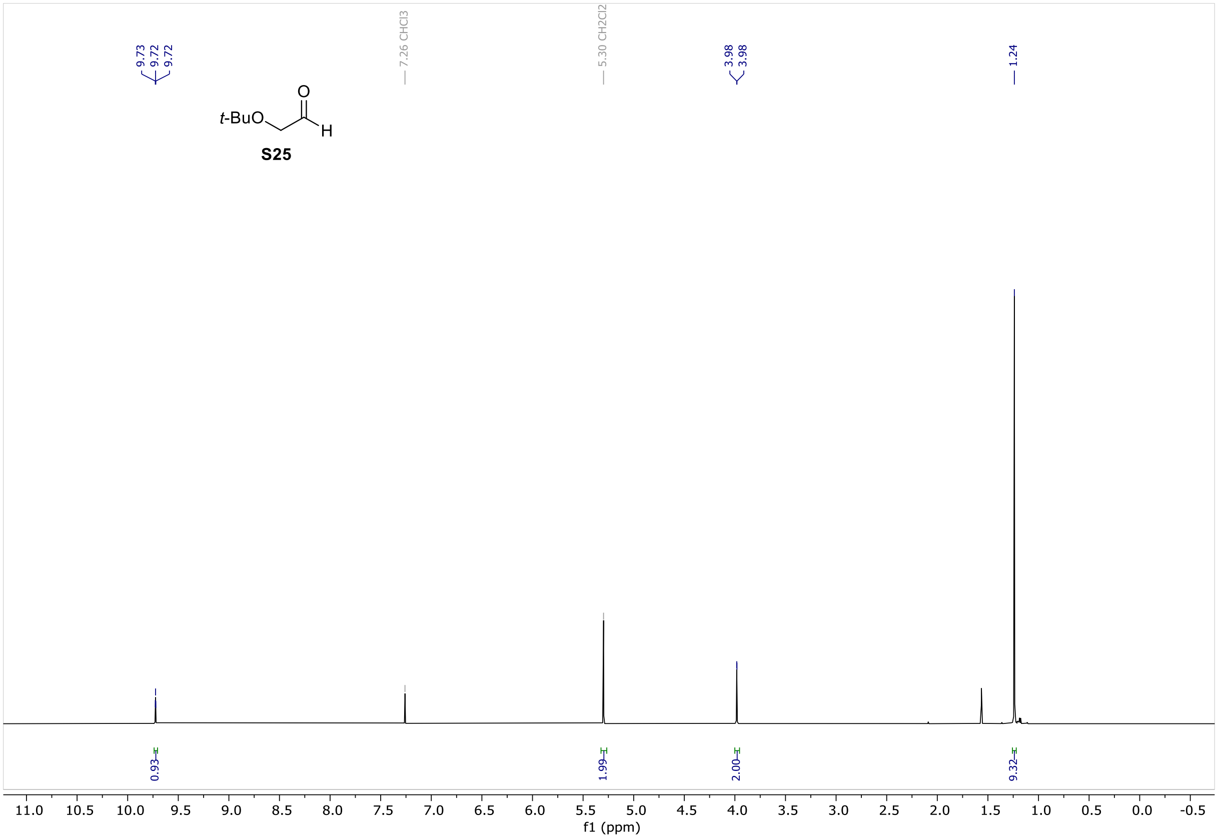

[1]**H-NMR** spectrum of compound **S25.**

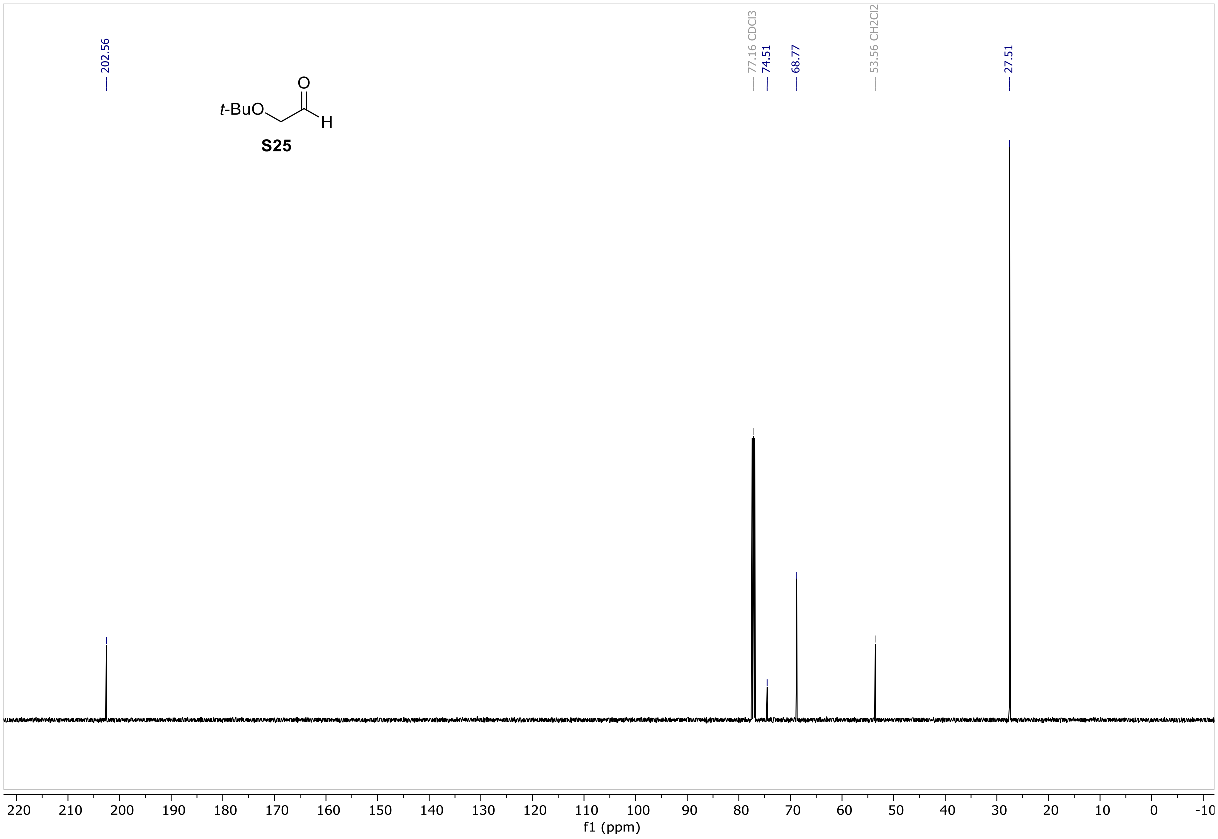

[13]**C-NMR** spectrum of compound **S25.**

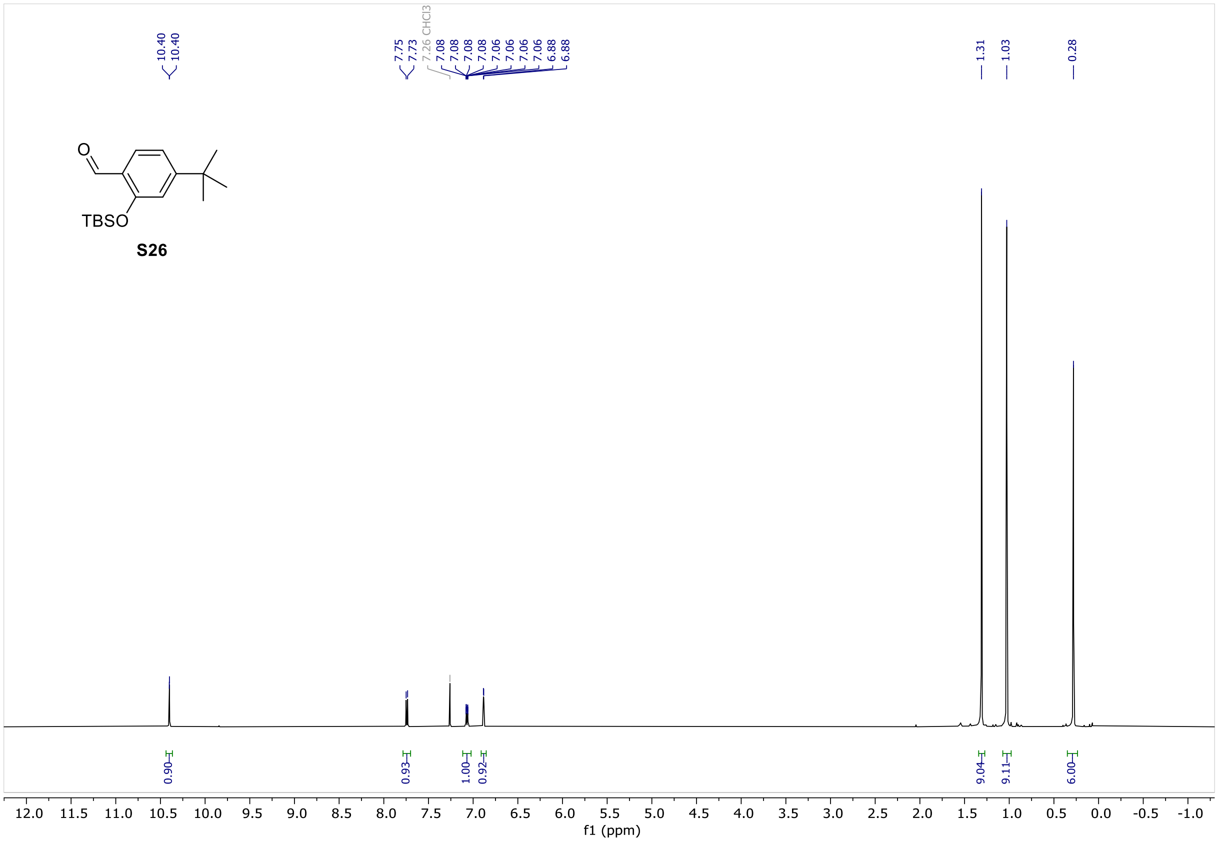

^{1}H-NMR spectrum of compound **S26.**

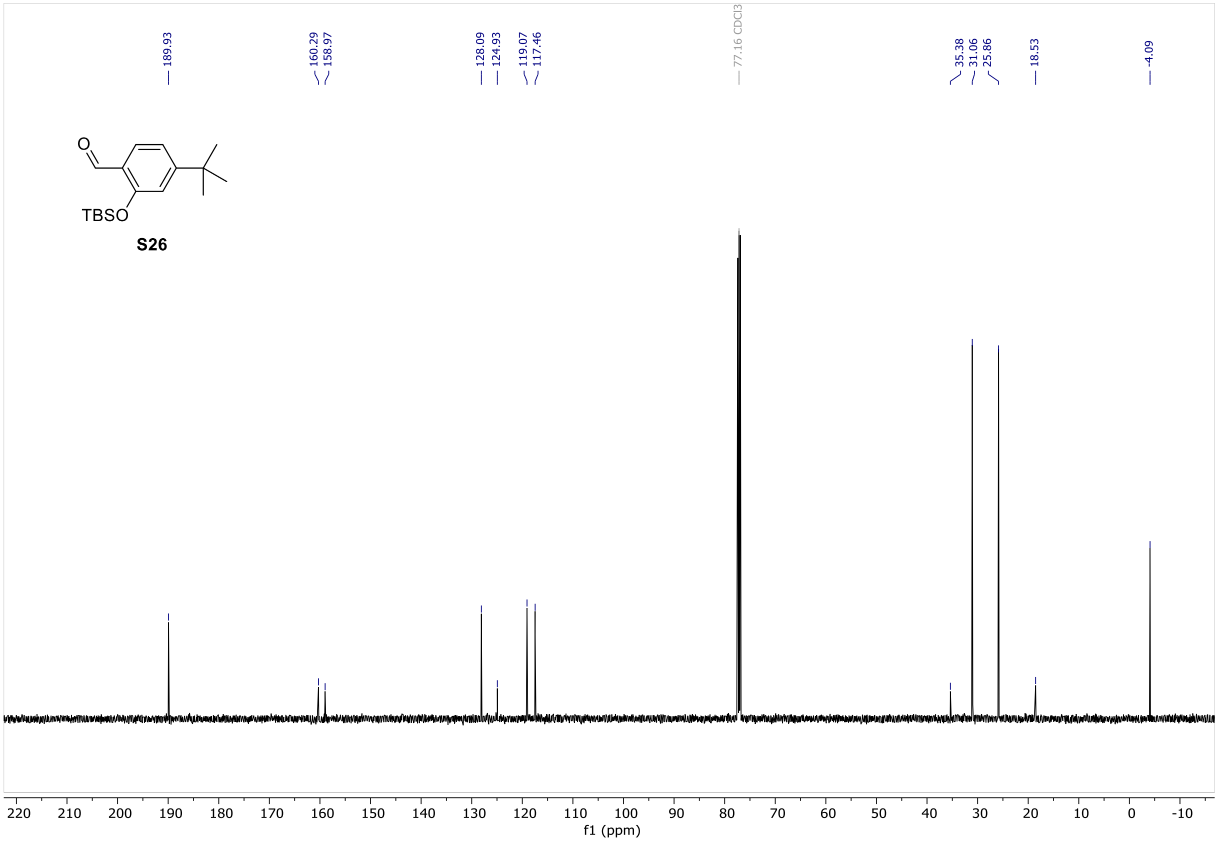

^{13}C-NMR spectrum of compound **S26.**

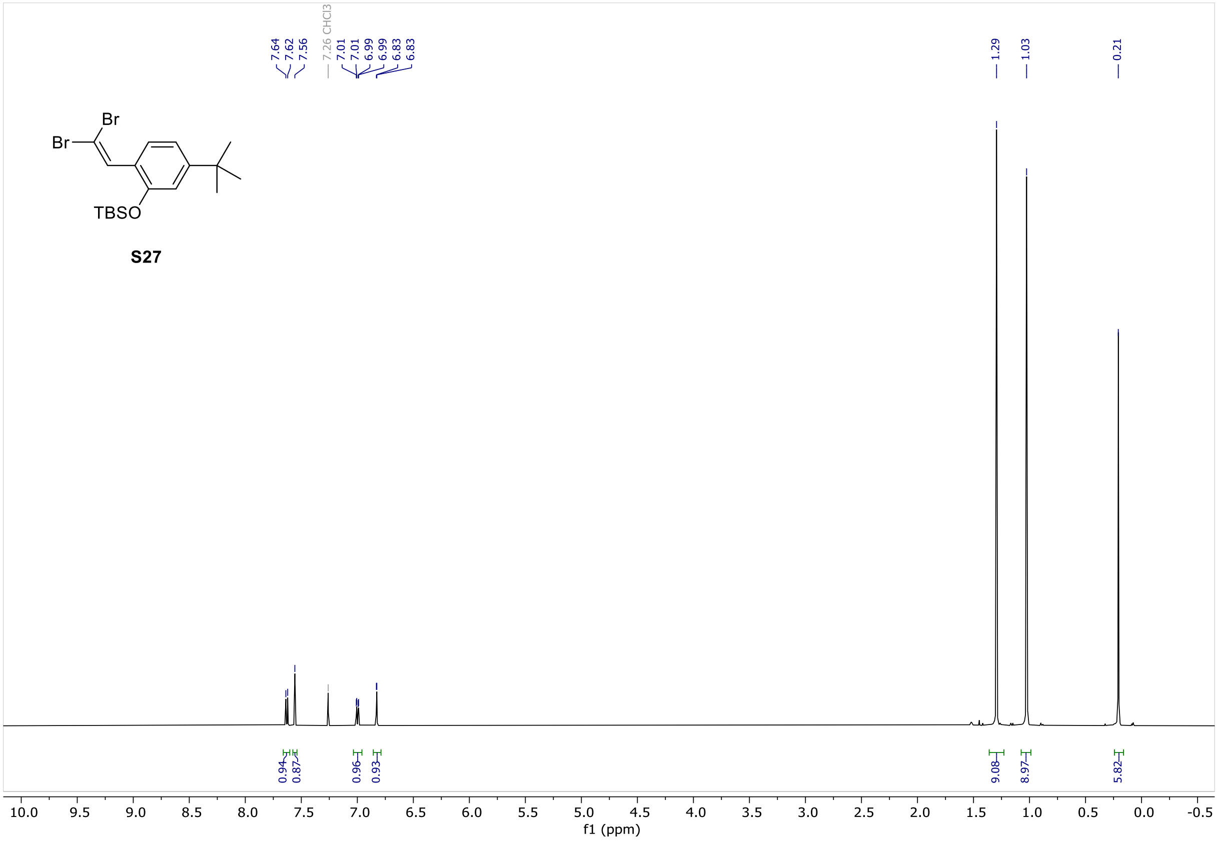

^{1}H-NMR spectrum of compound **S27.**

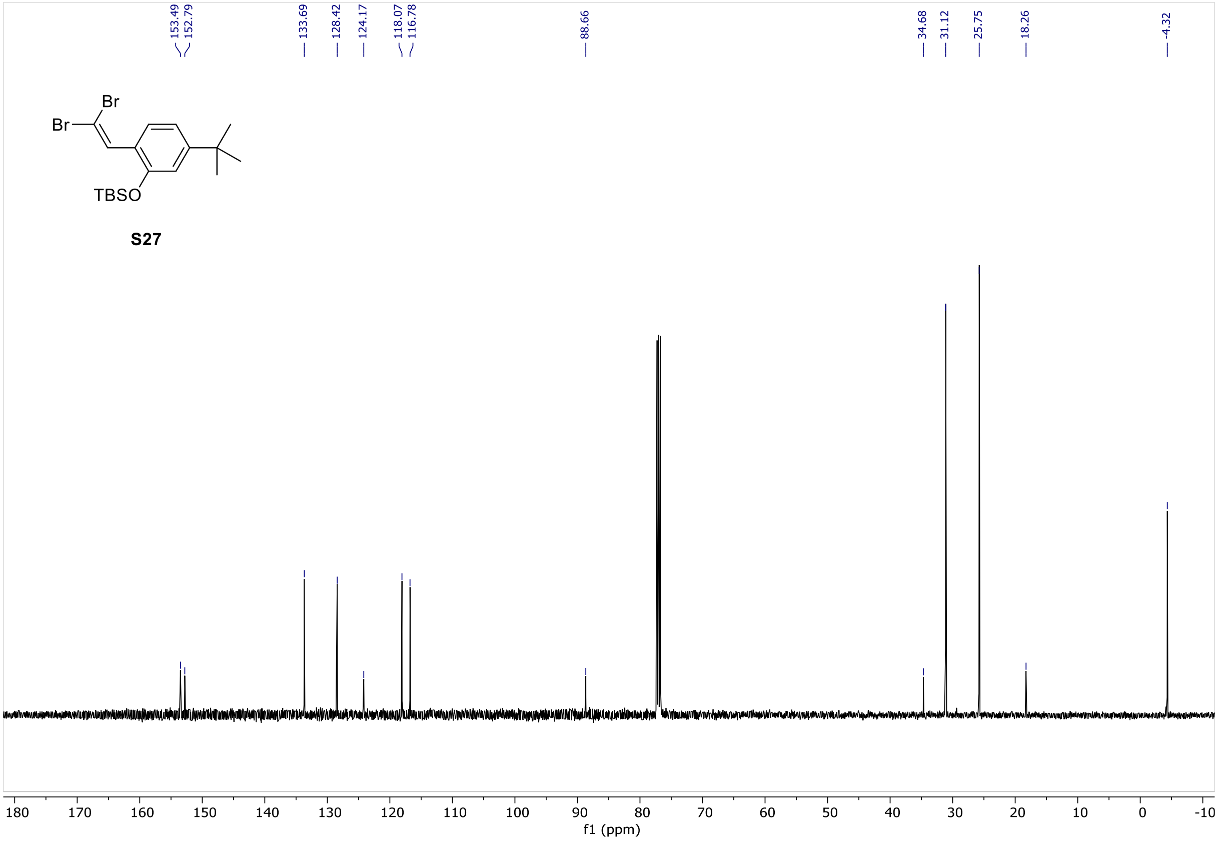

^{13}C-NMR spectrum of compound **S27.**

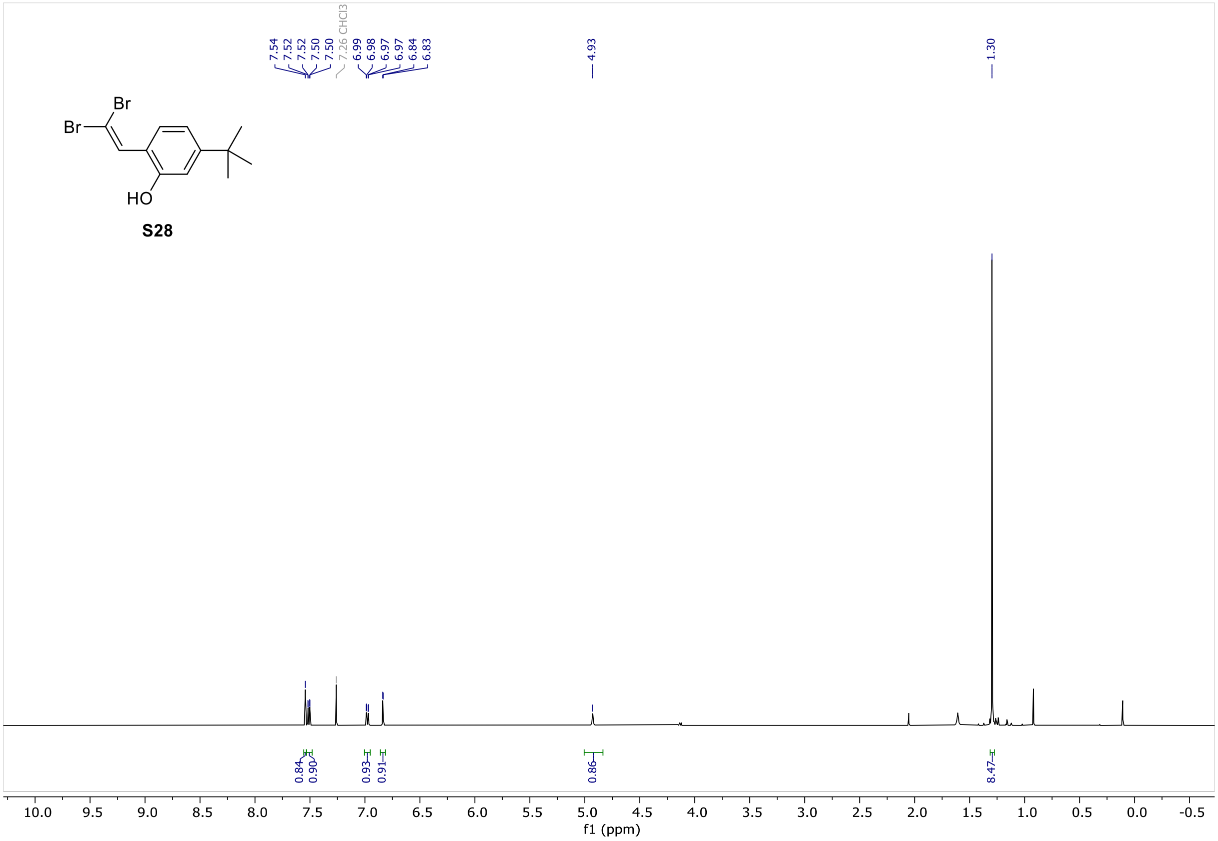

^{1}H-NMR spectrum of compound **S28.**

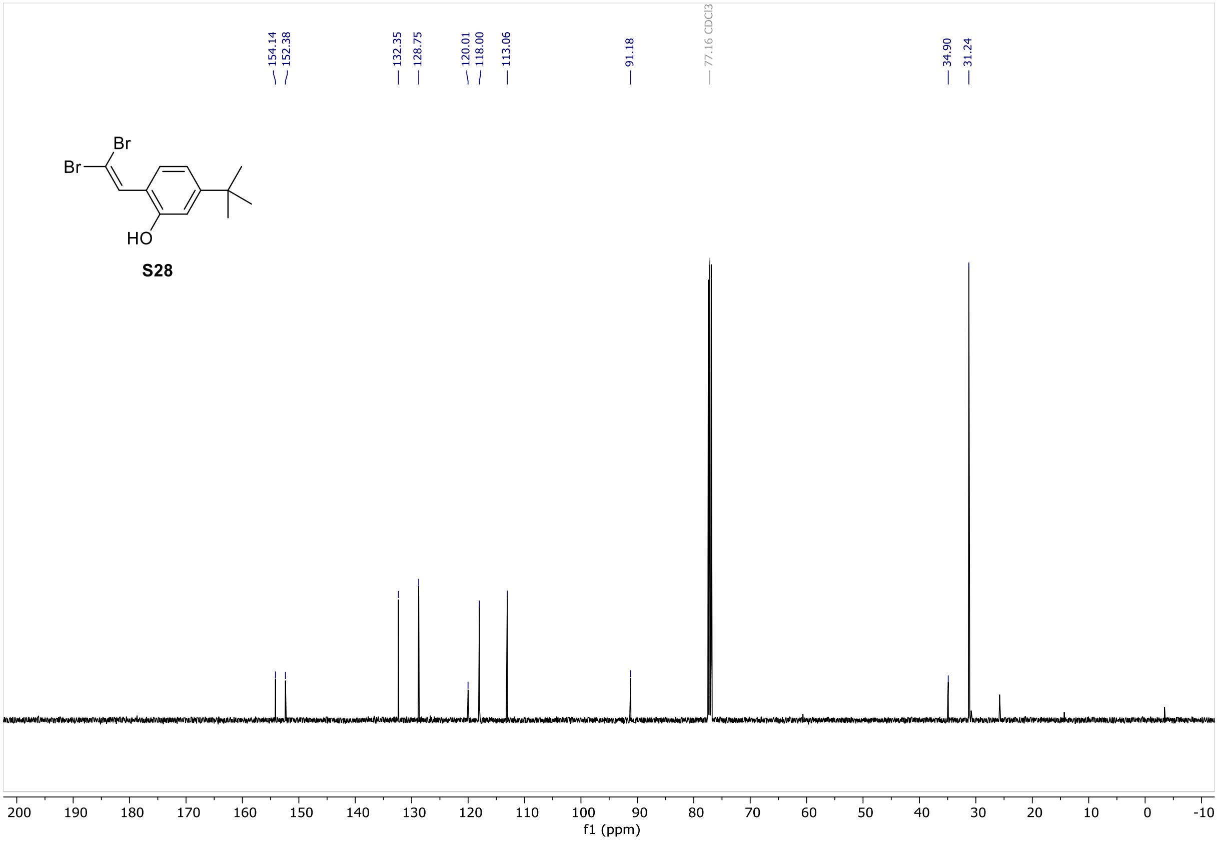

^{13}C-NMR spectrum of compound **S28.**

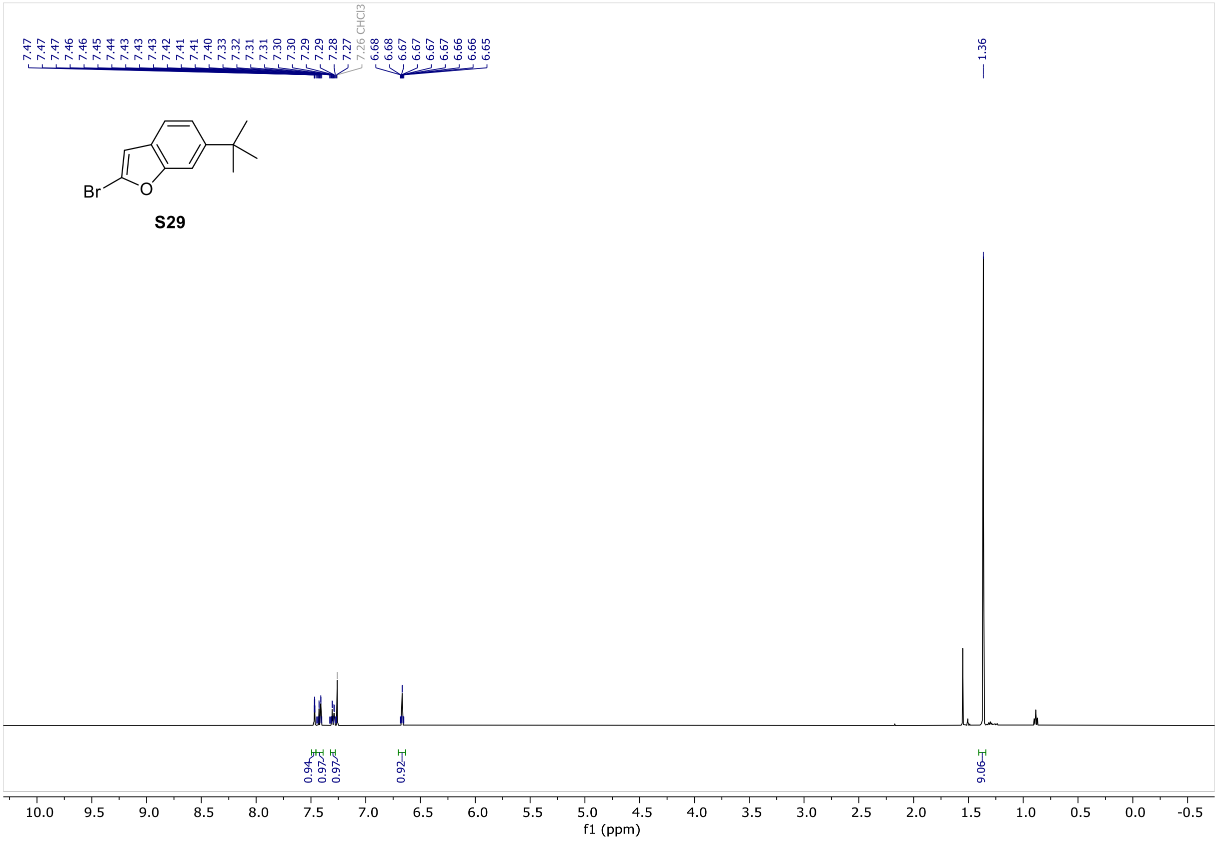

^{1}H-NMR spectrum of compound **S29.**

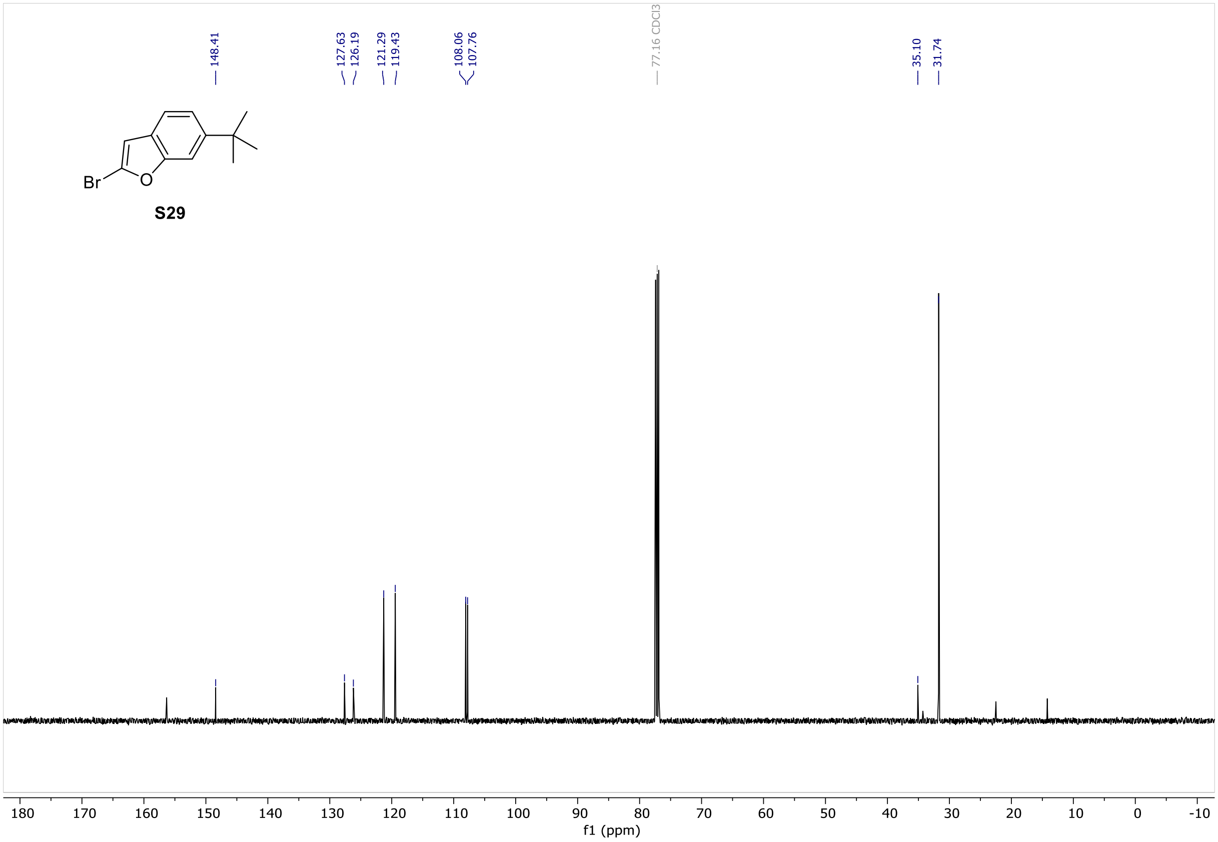

^{13}C-NMR spectrum of compound **S29.**

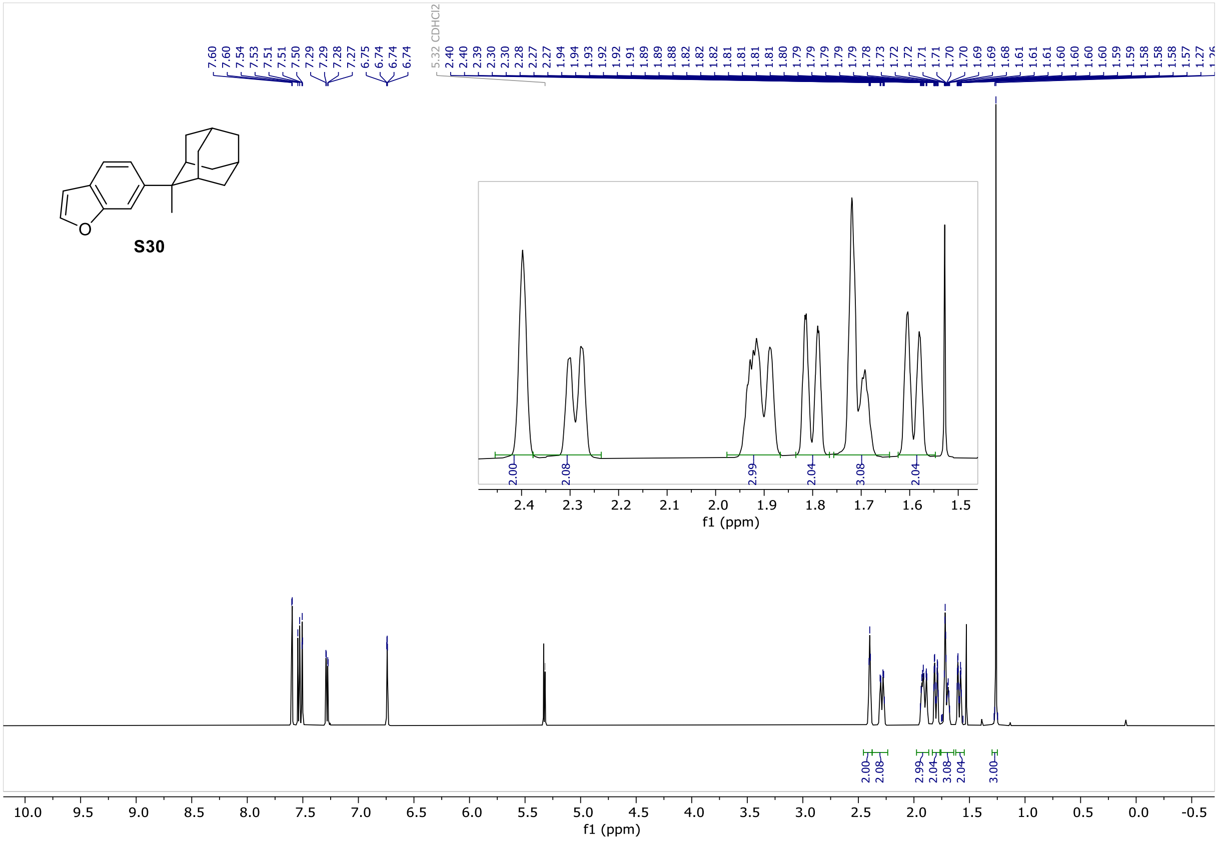

^{1}**H-NMR** spectrum of compound **S30.**

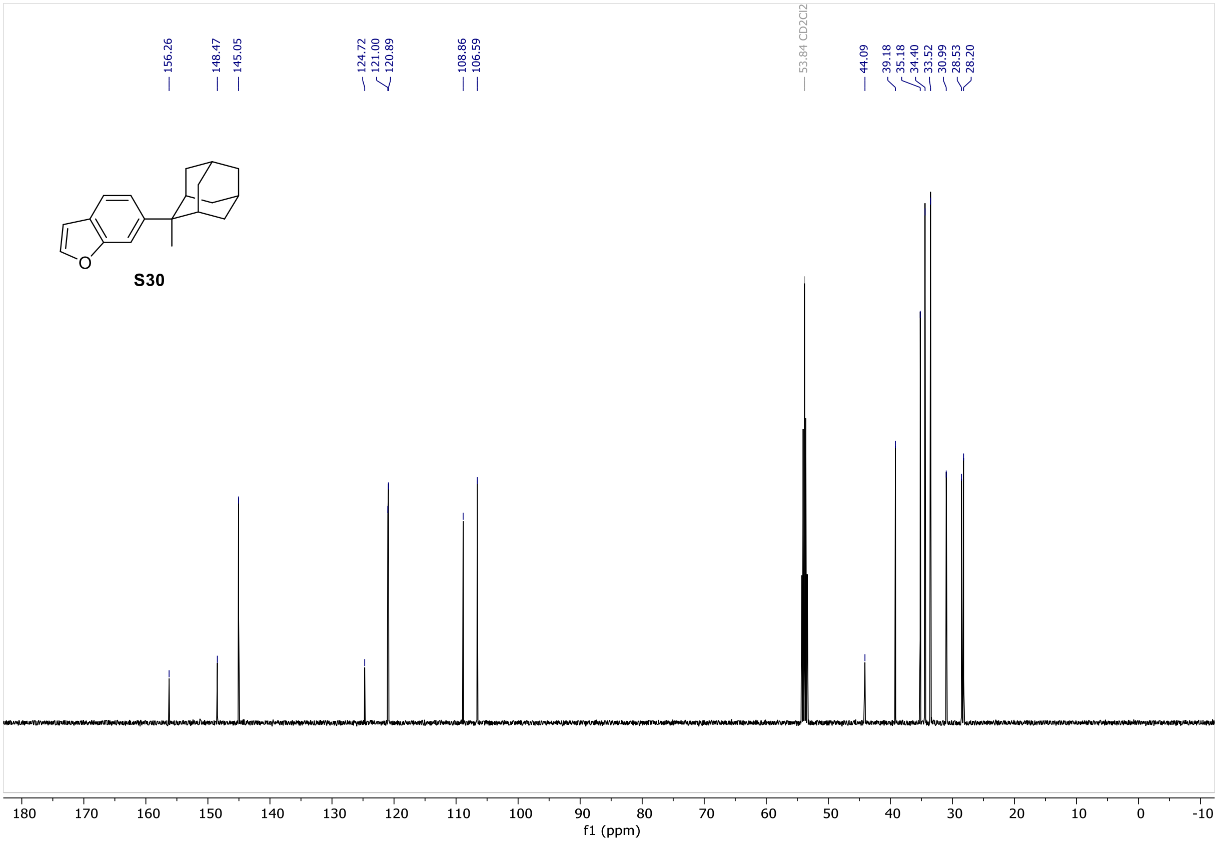

^{13}**C-NMR** spectrum of compound **S30.**

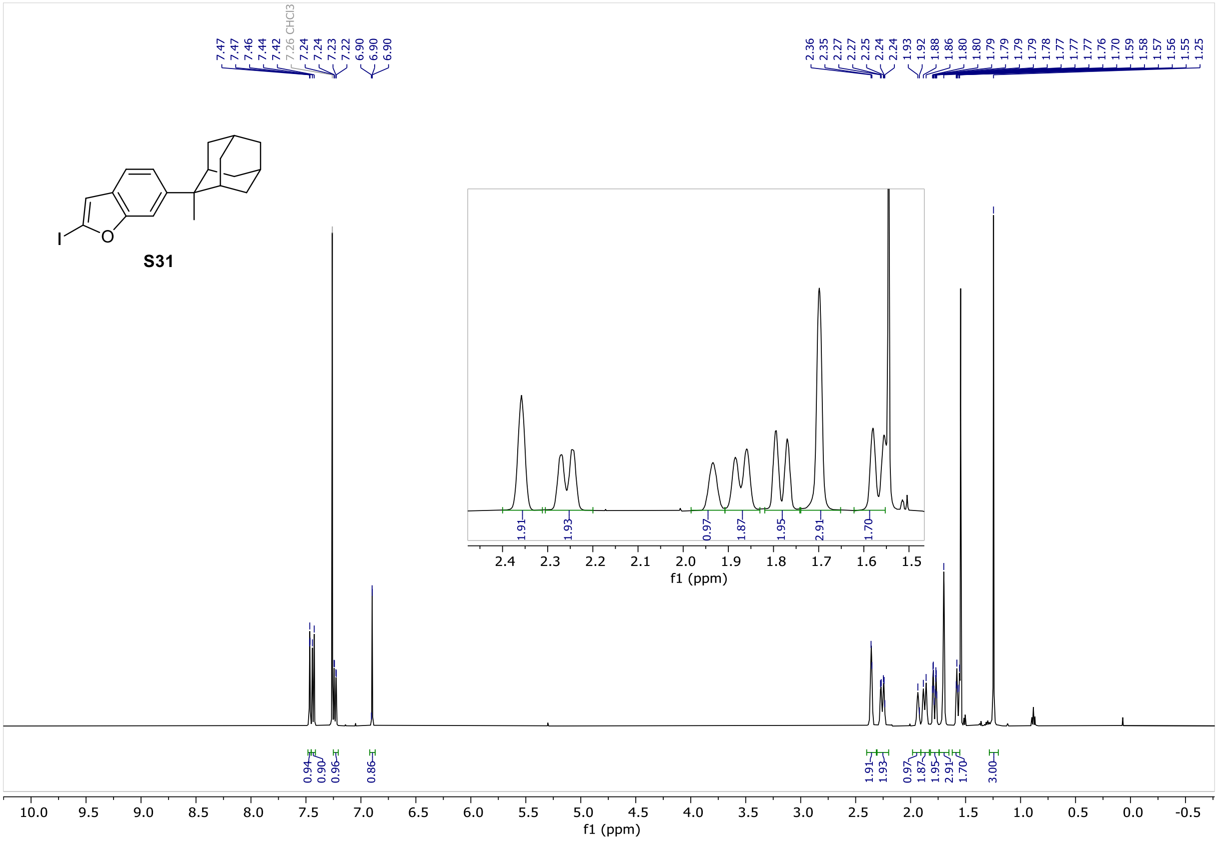

^{1}H-NMR spectrum of compound **S31.**

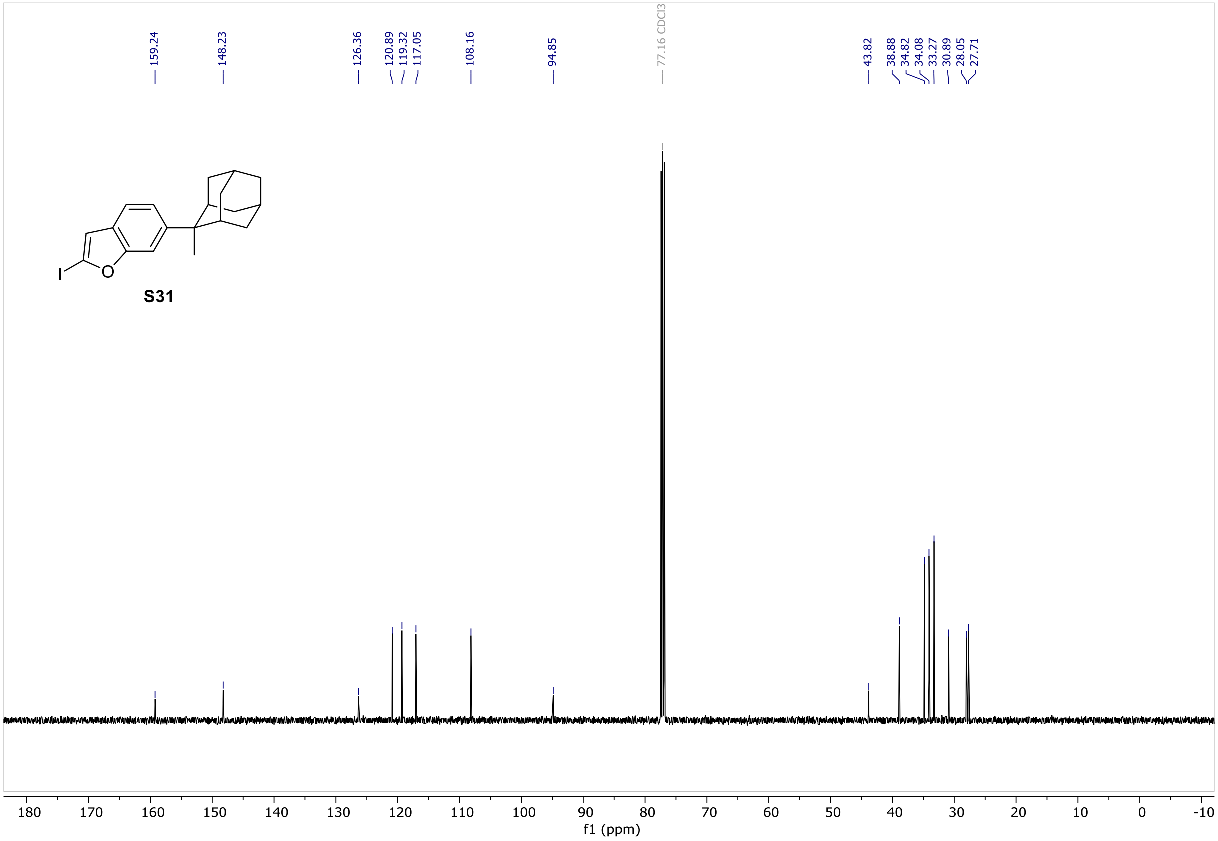

^{13}C-NMR spectrum of compound **S31.**

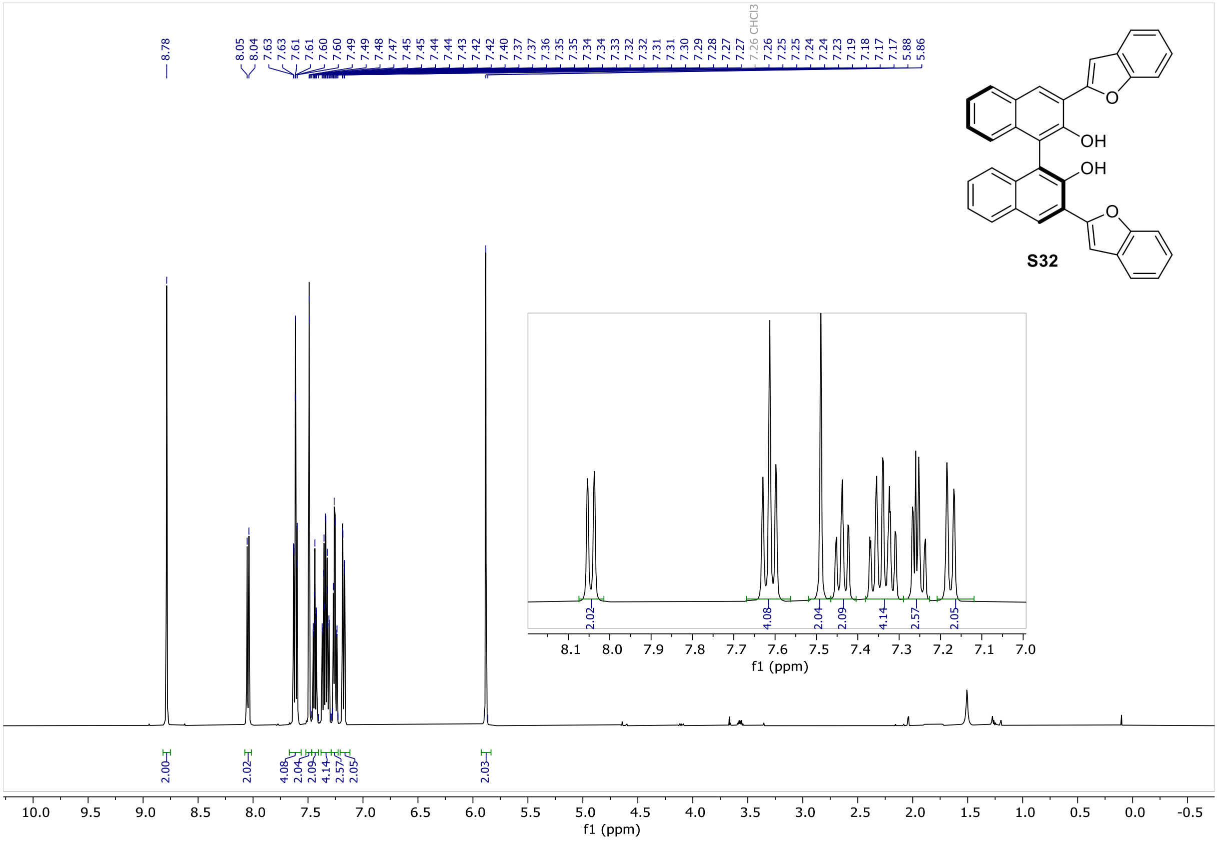

^{1}H-NMR spectrum of compound **S32.**

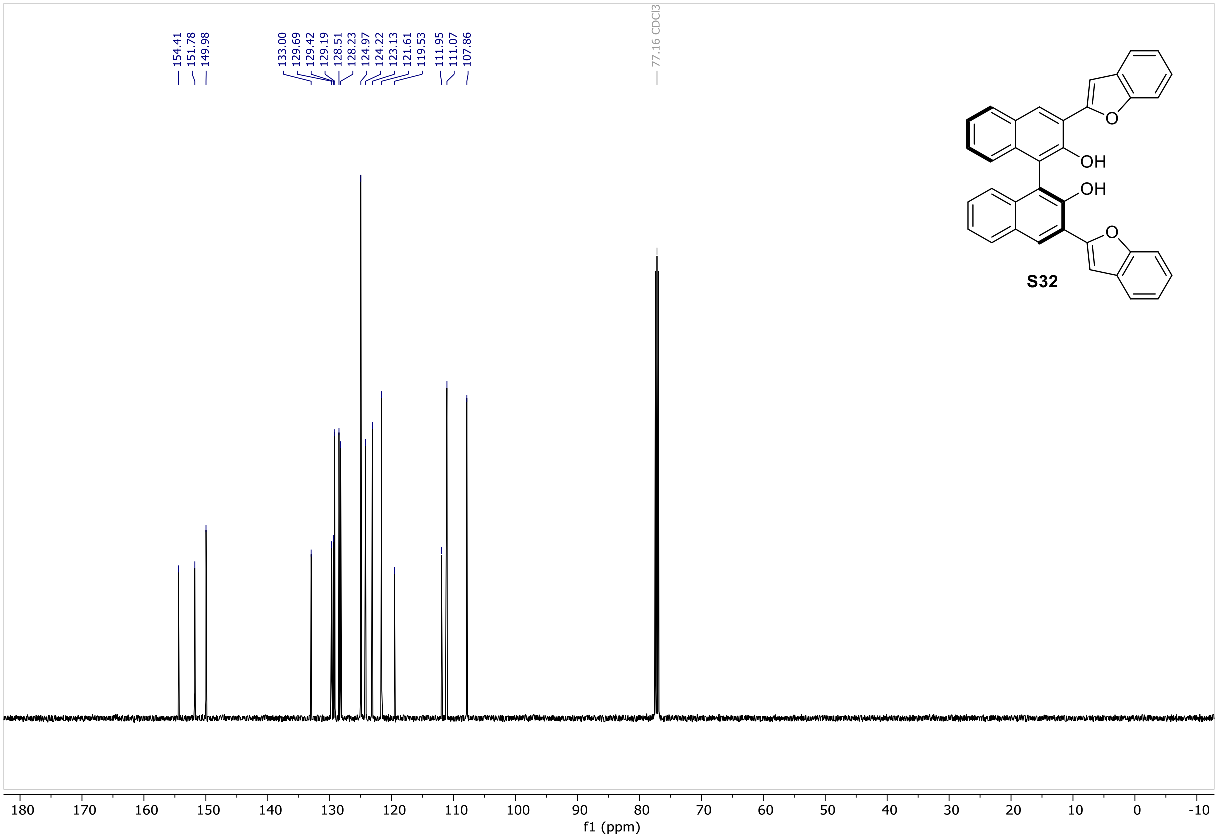

^{13}C-NMR spectrum of compound **S32.**

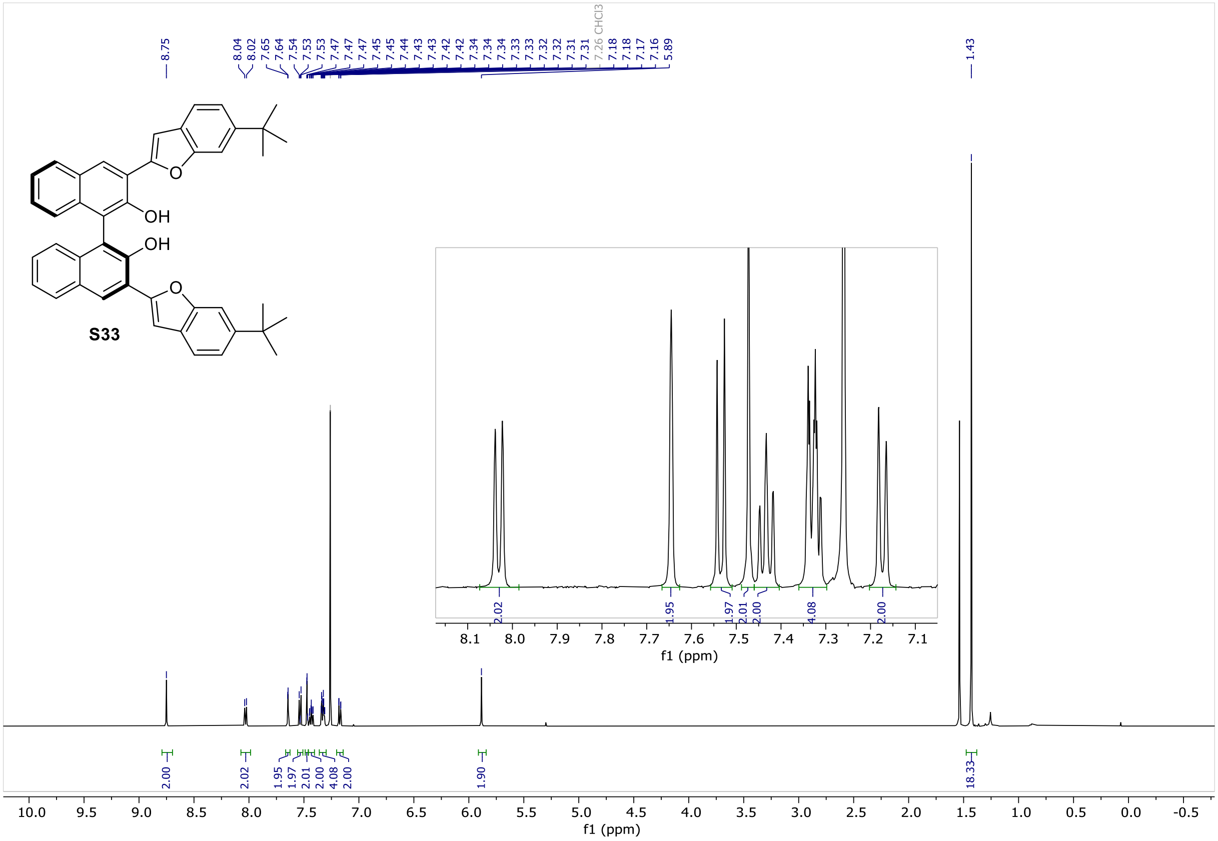

^{1}H-NMR spectrum of compound **S33.**

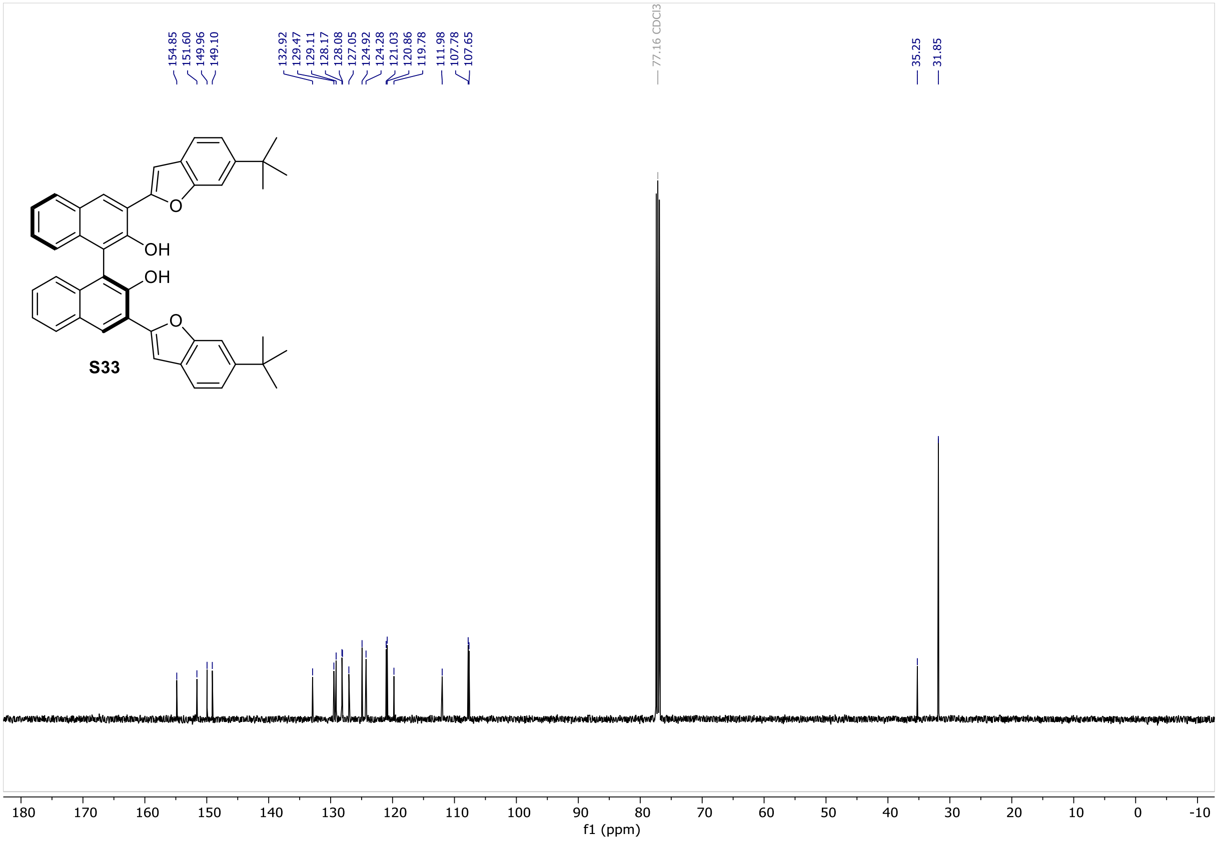

^{13}C-NMR spectrum of compound **S33.**

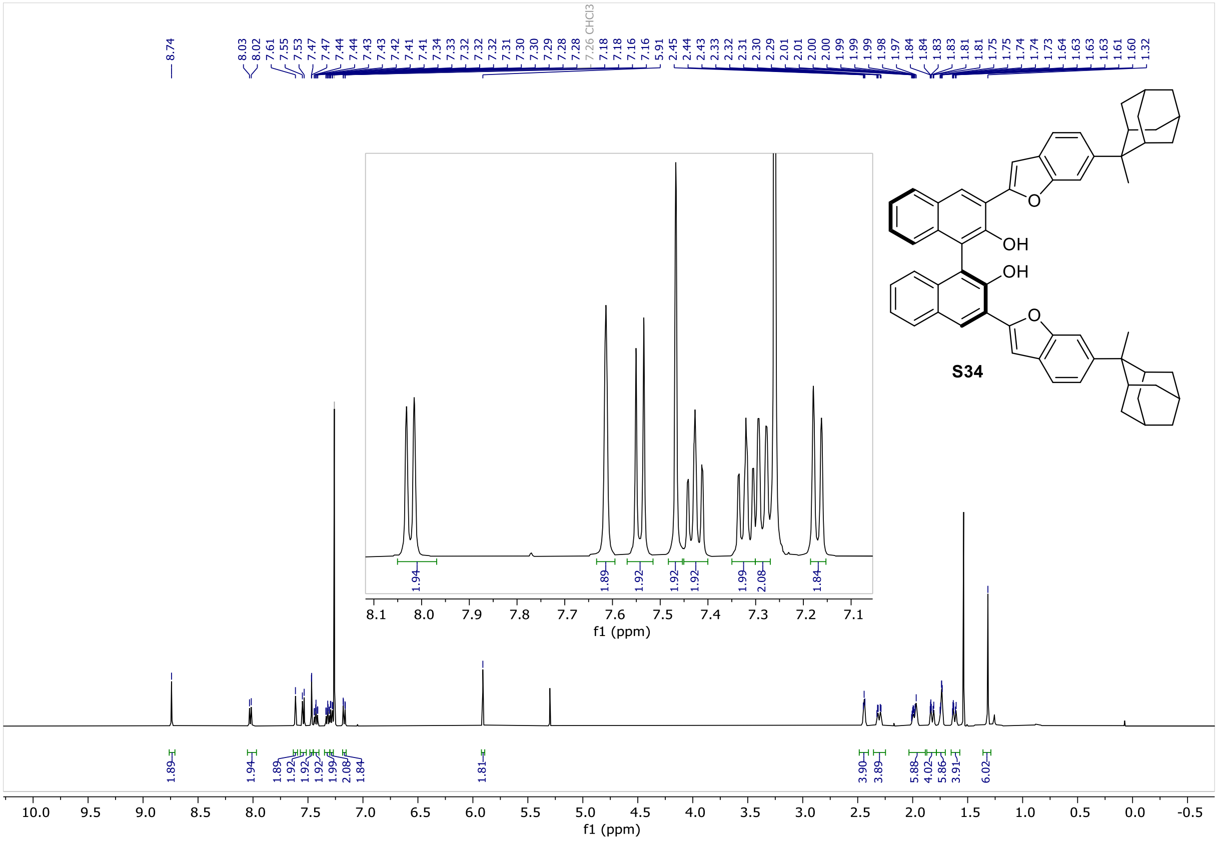

^{1}H-NMR spectrum of compound **S34.**

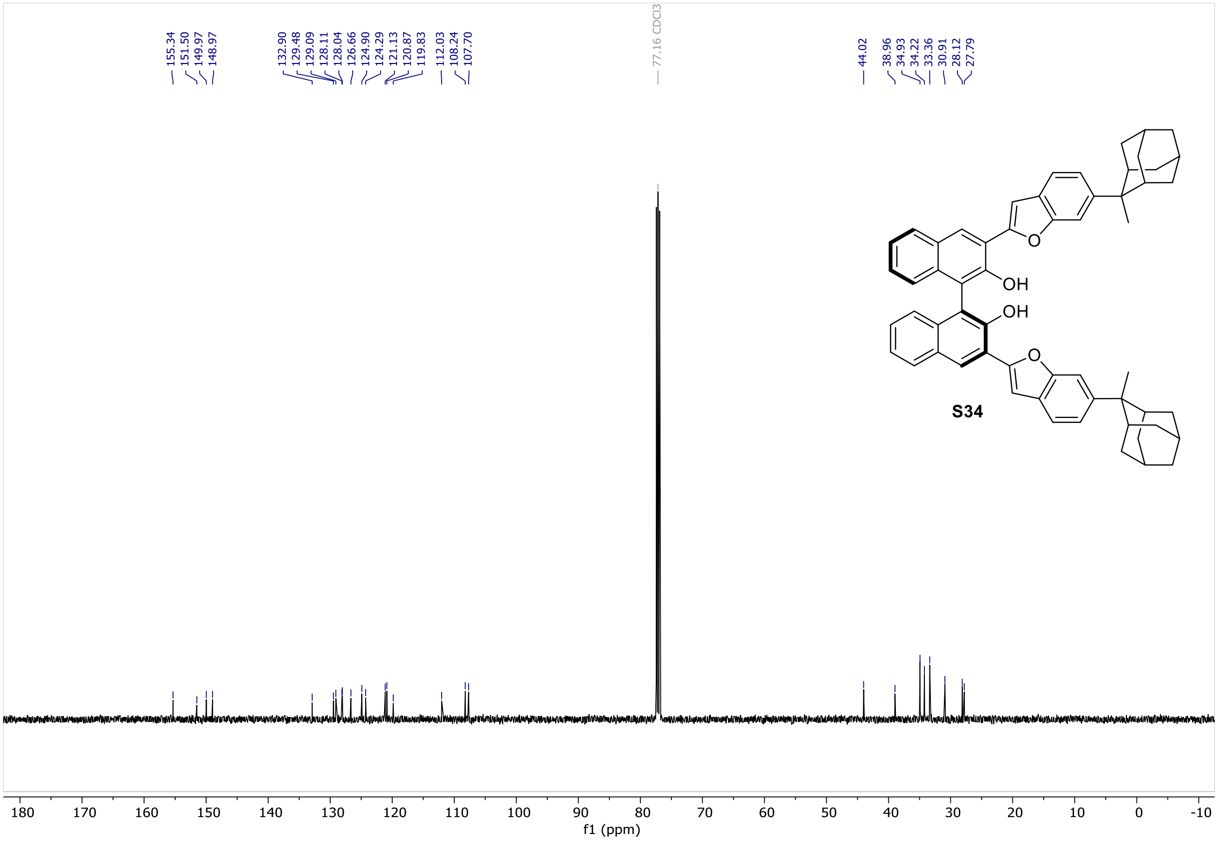

^{13}C-NMR spectrum of compound **S34.**

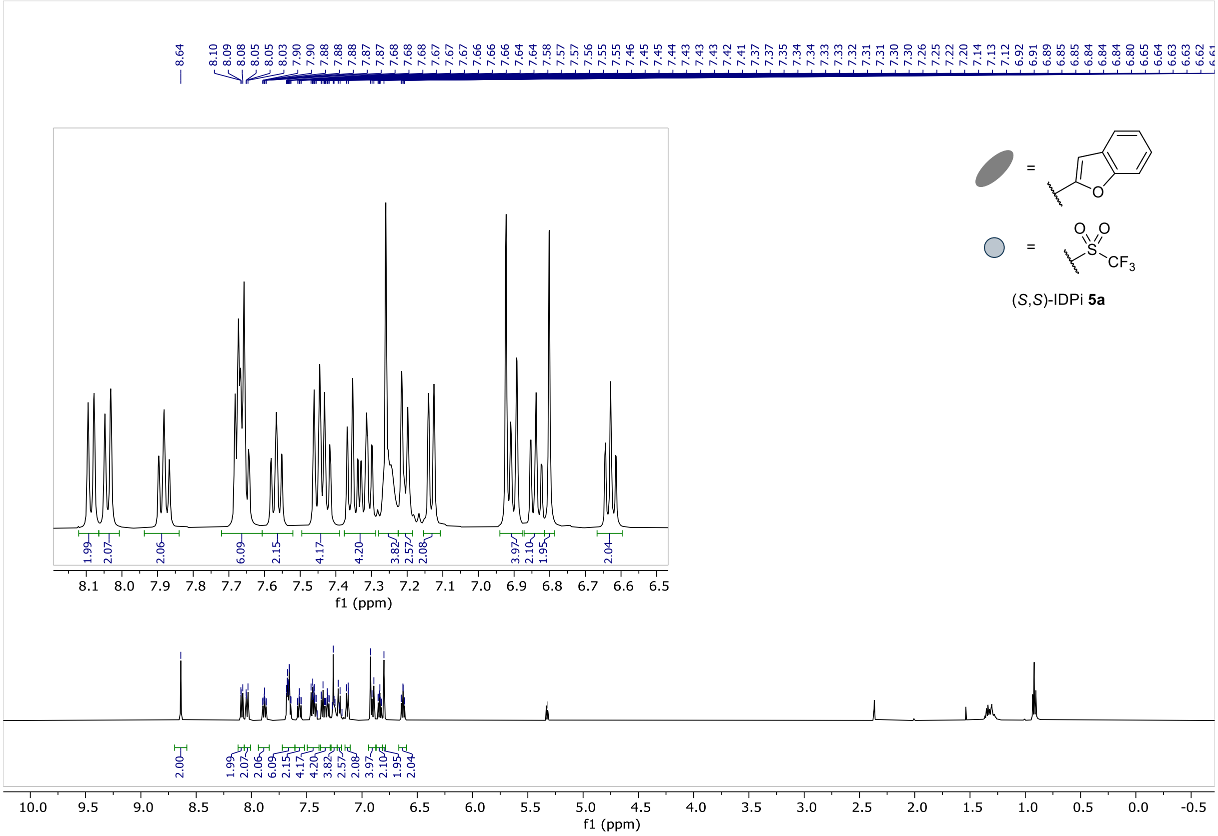

^{1}H-NMR spectrum of IDPi **5a.**

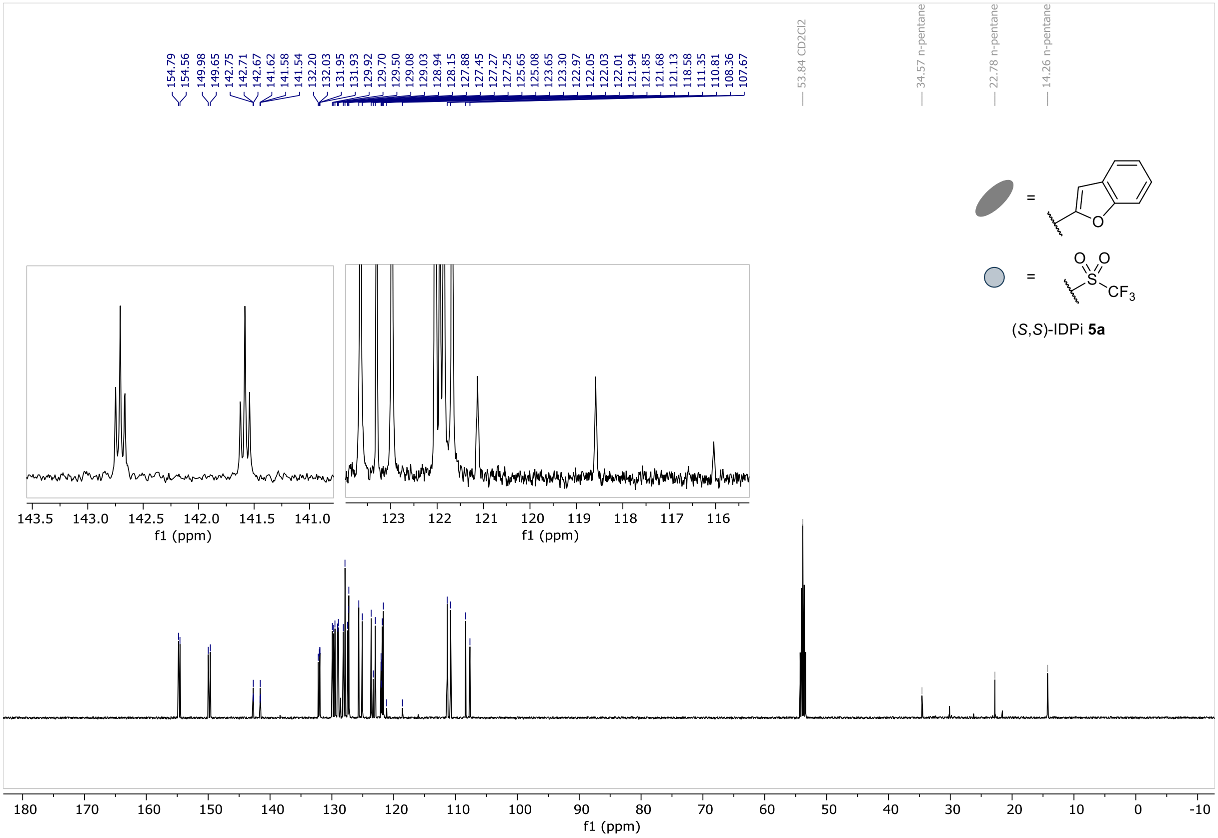

^{13}C-NMR spectrum of IDPi 5**a.**

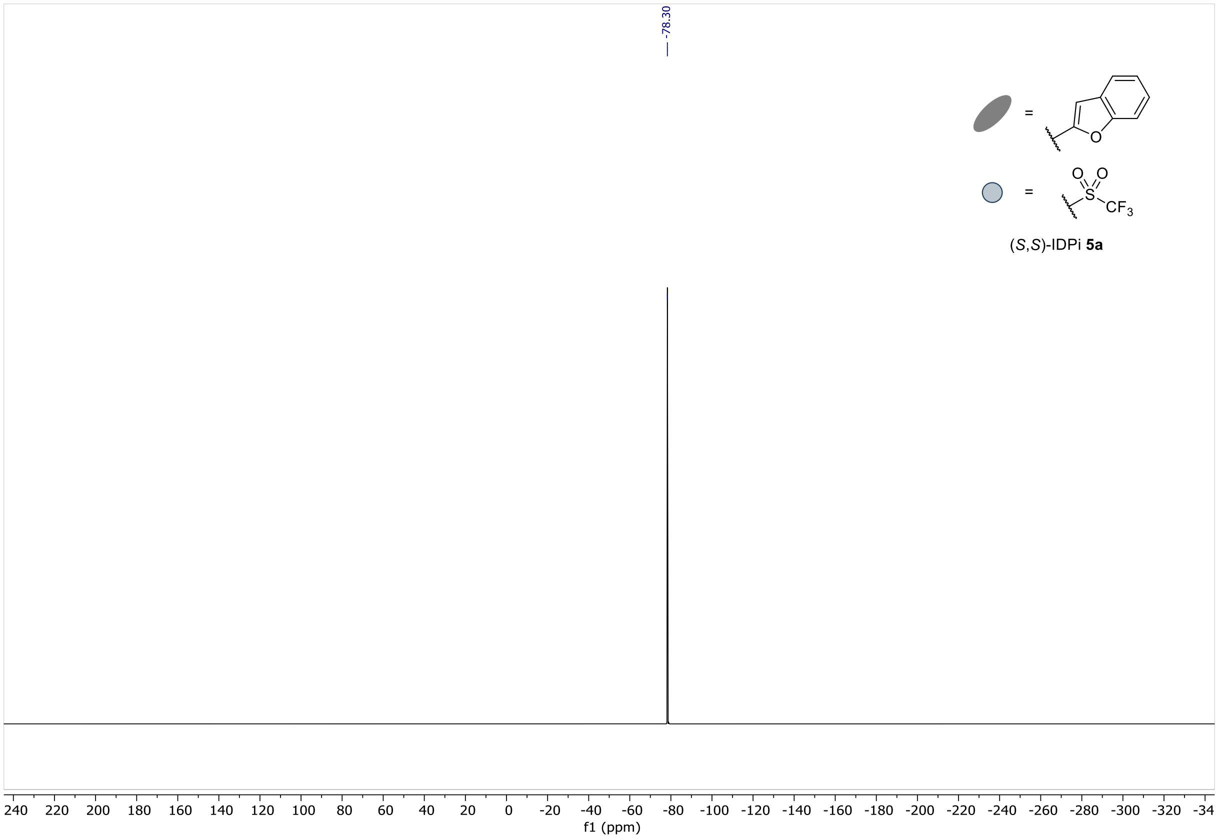

^{19}F-NMR spectrum of IDPi **5a.**

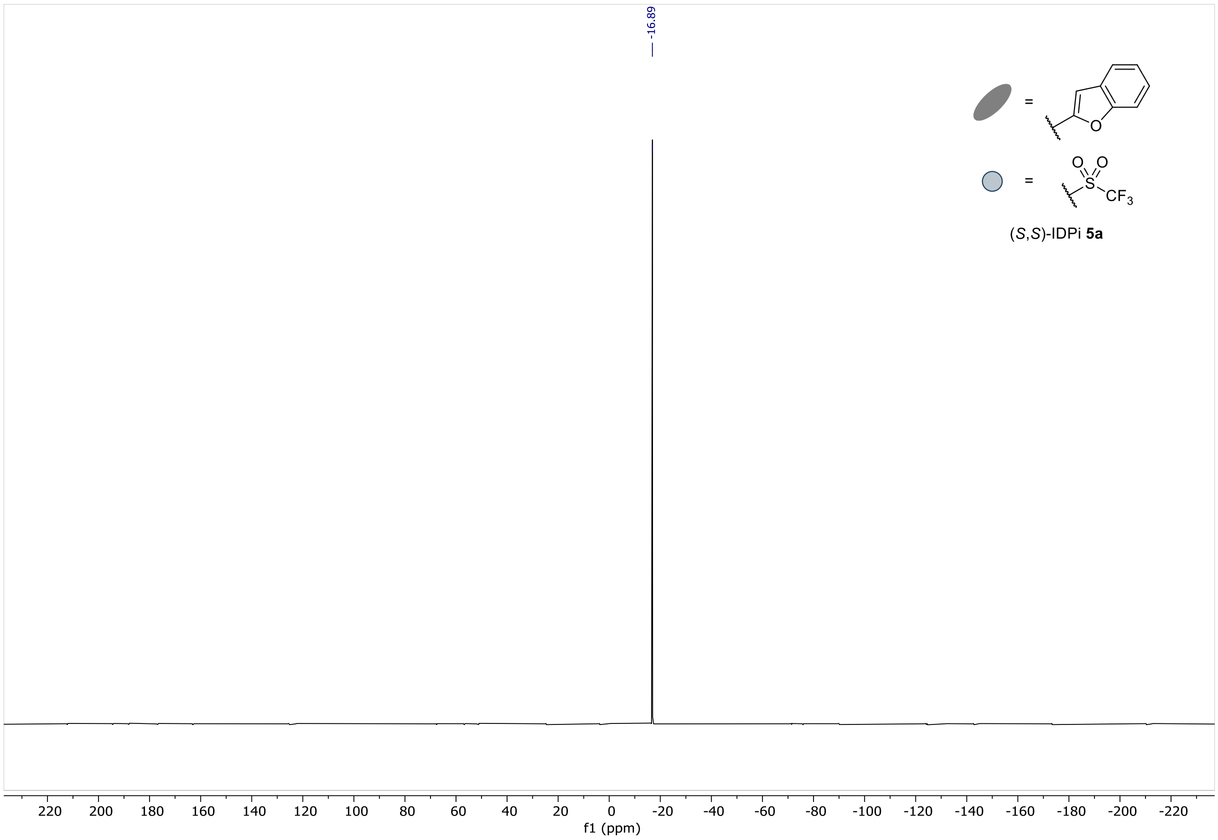

^{31}P-NMR spectrum of IDPi **5a.**

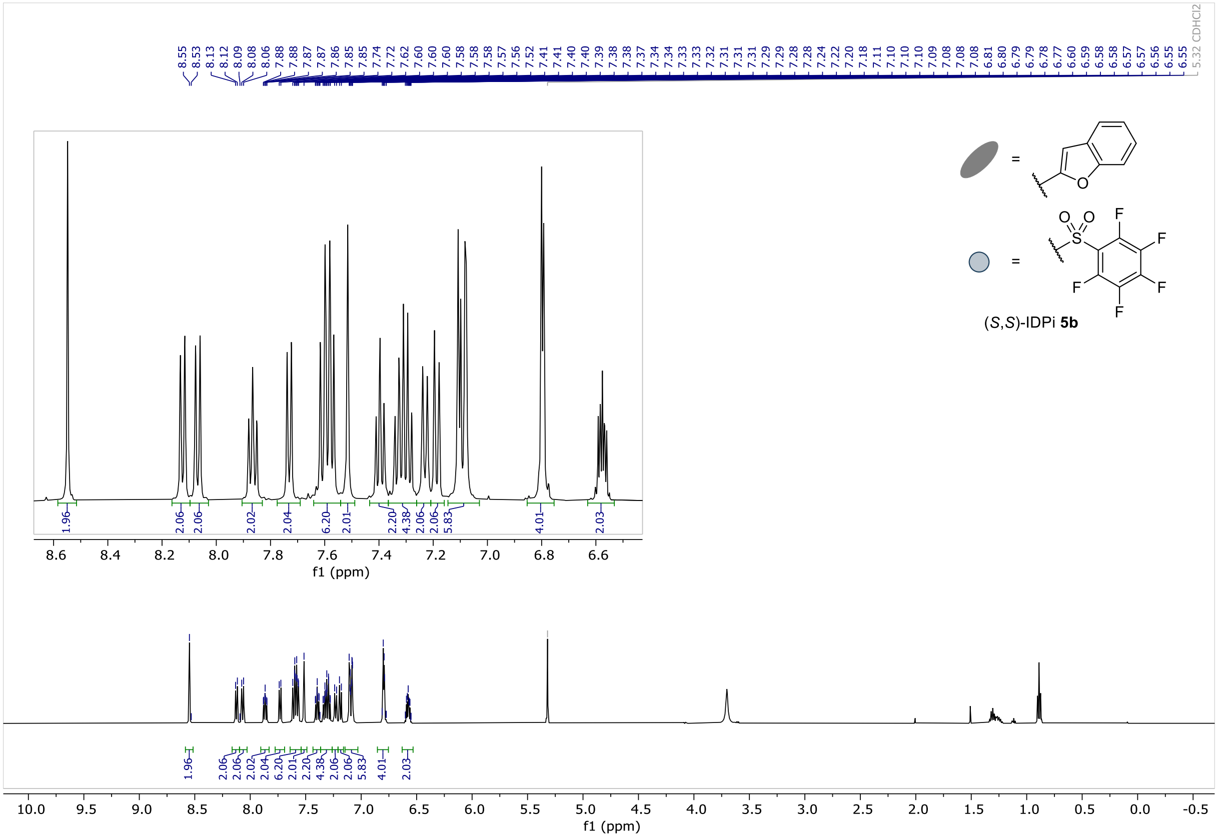

^{1}**H-NMR** spectrum of IDPi **5b.**

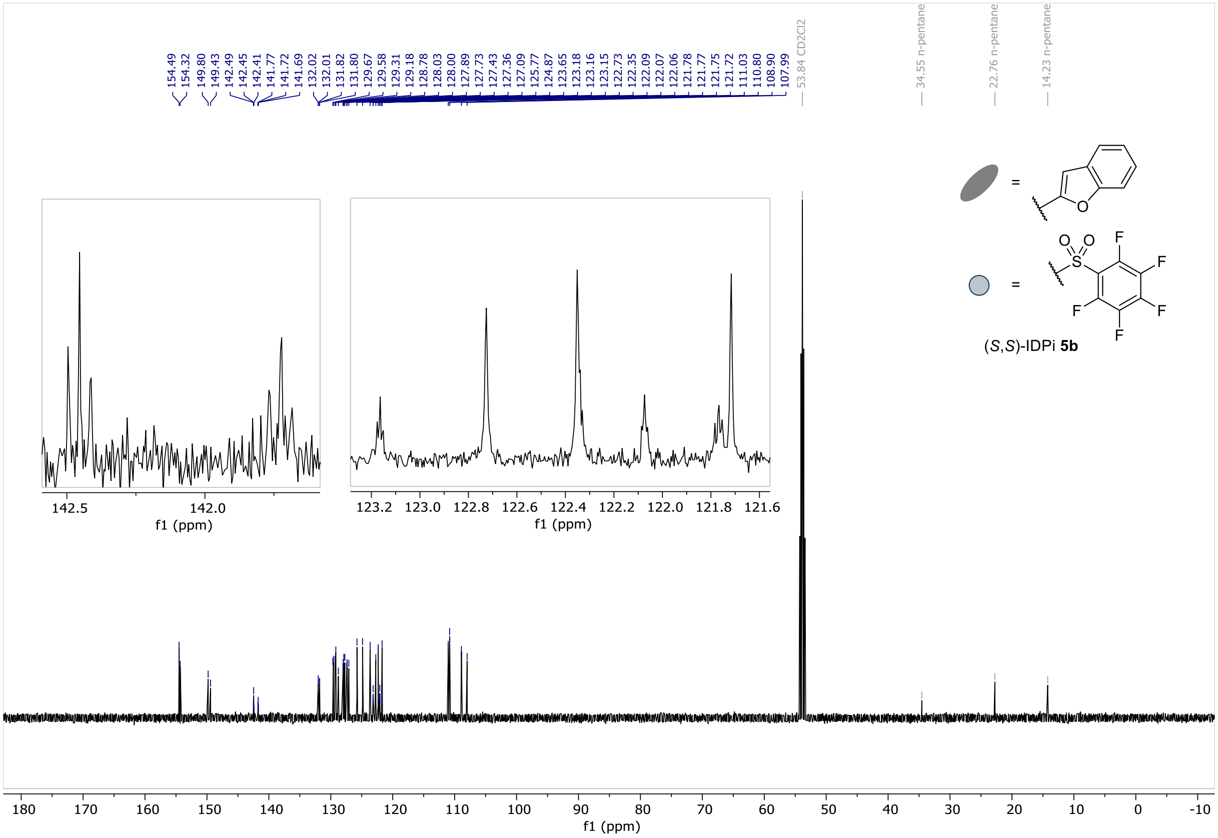

^{13}**C-NMR** spectrum of IDPi **5b.**

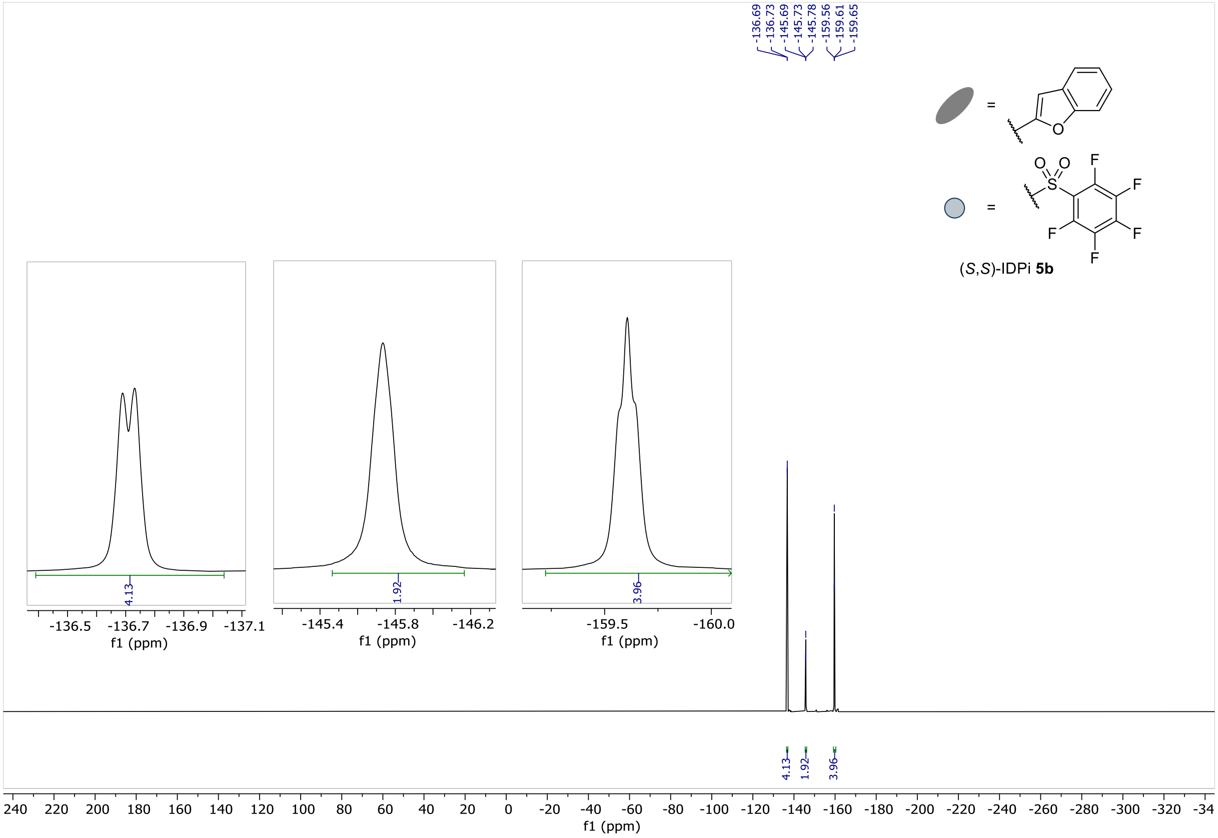

^{19}F-NMR spectrum of IDPi **5b.**

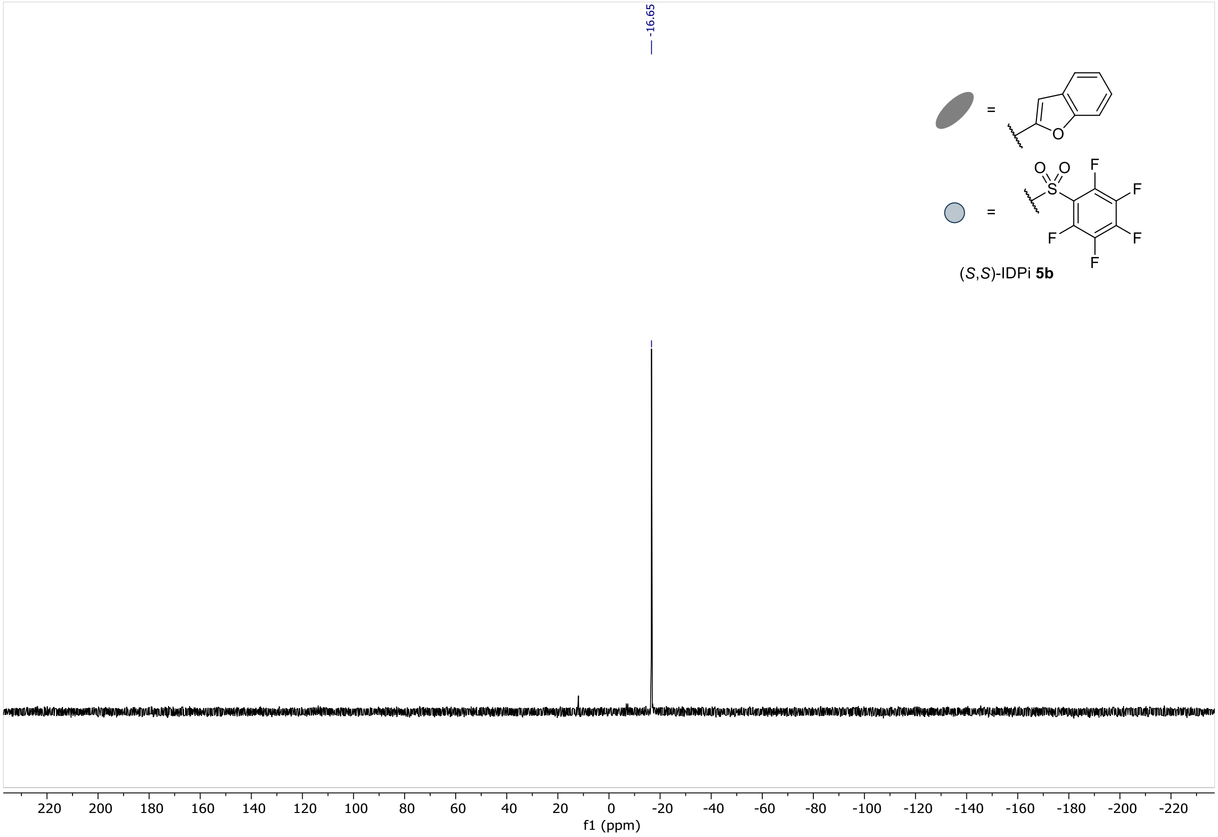

^{31}P-NMR spectrum of IDPi **5b.**

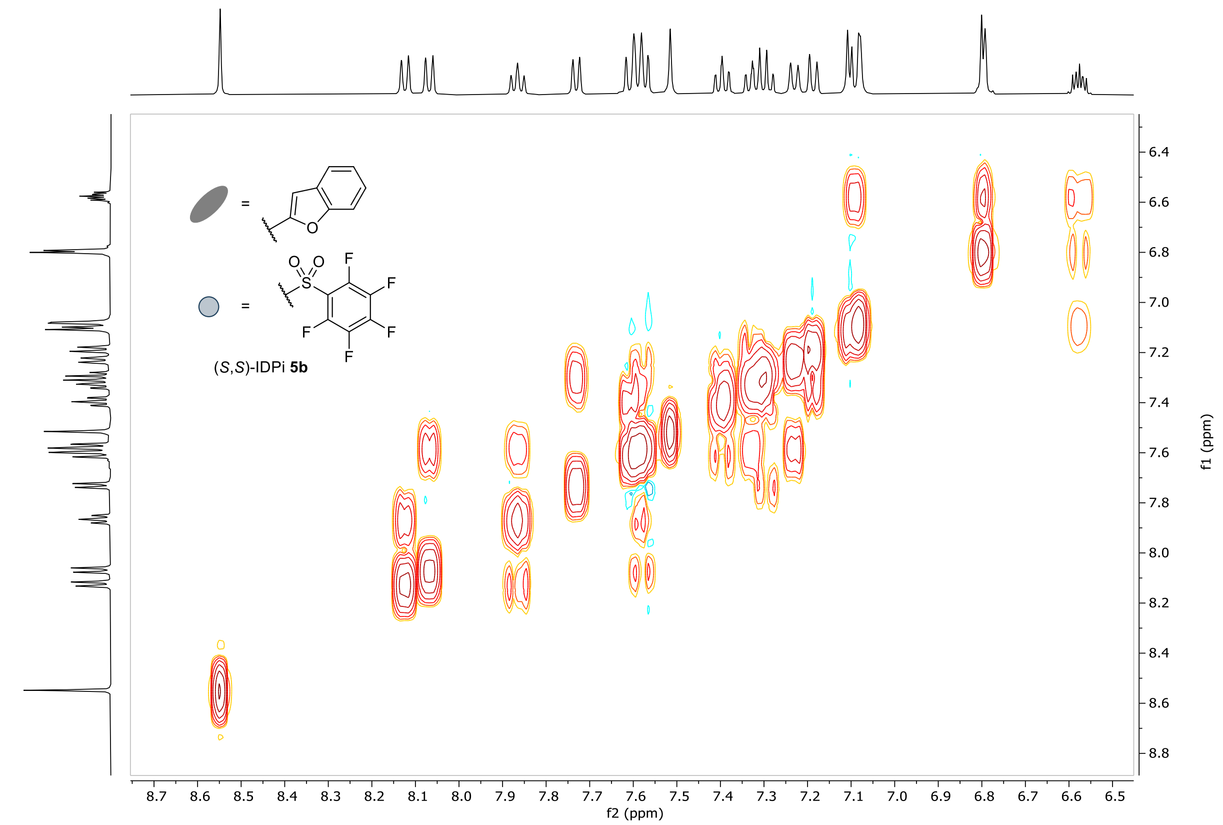

^{1}**H-COSY** NMR-spectrum of IDPi **5b.**

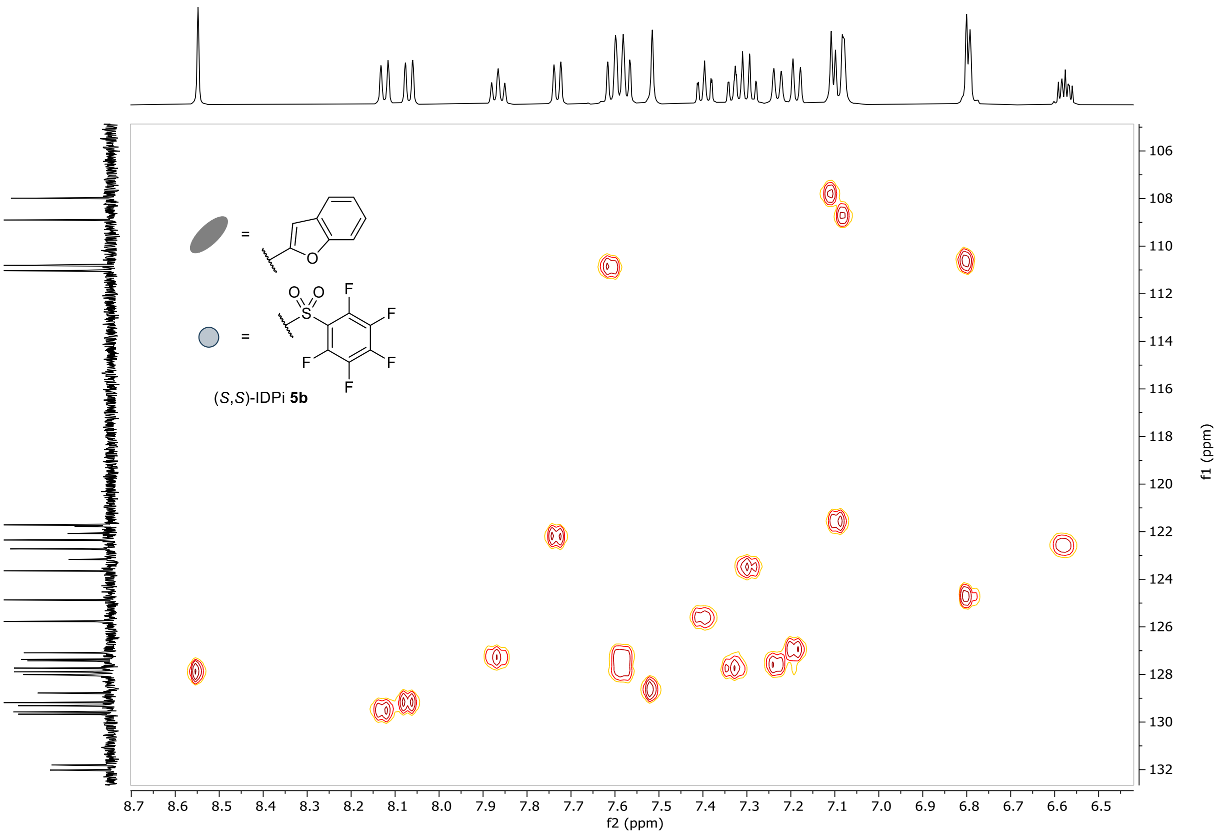

^{1}**H-**^{13}**C-HSQC** NMR-spectrum of IDPi **5b.**

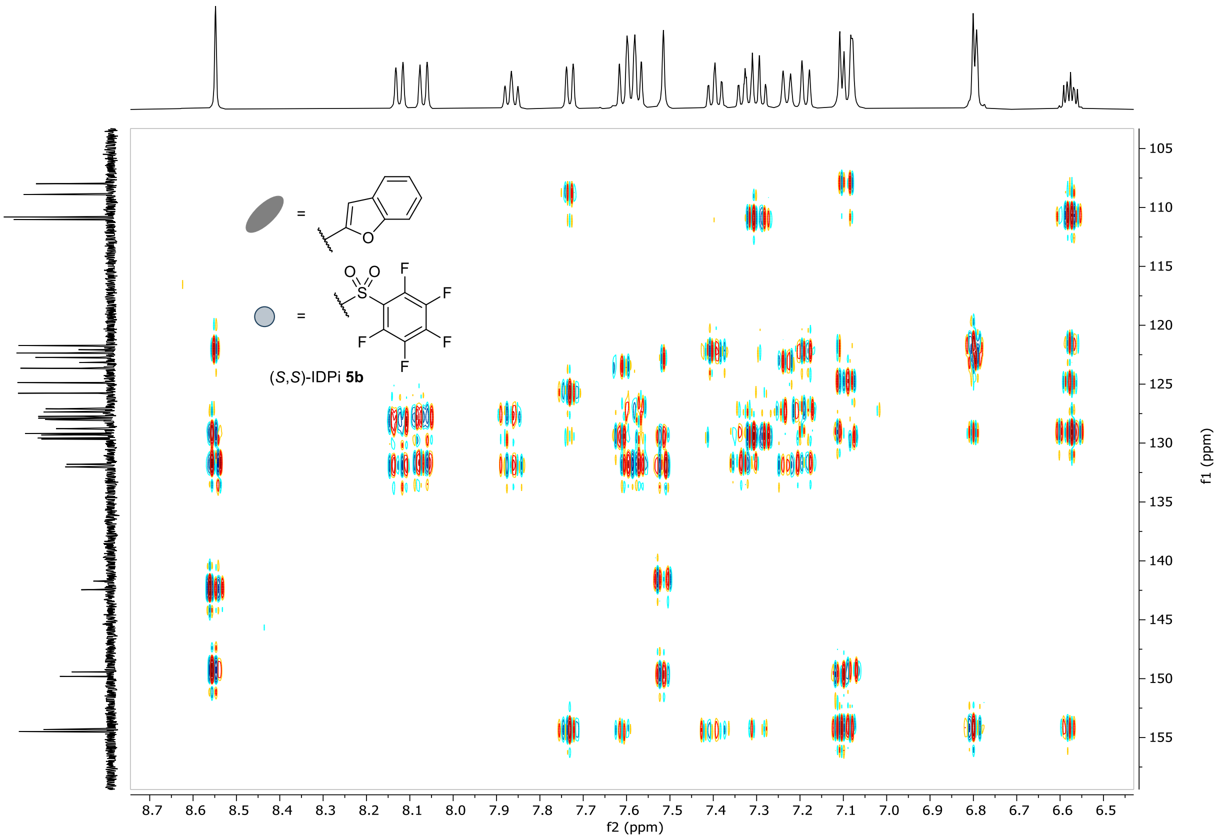

^{1}H-^{13}C-HMBC NMR-spectrum of IDPi **5b.**

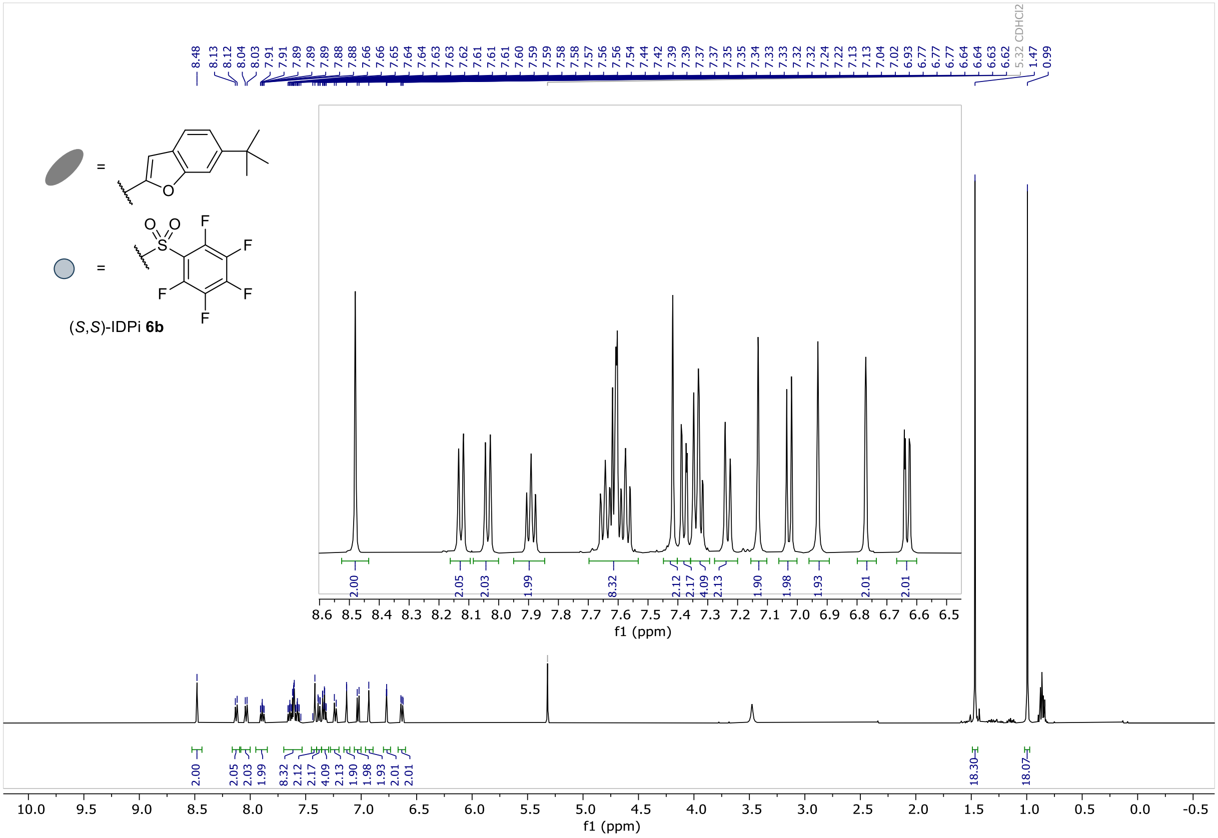

^{1}H-NMR spectrum of IDPi **6b.**

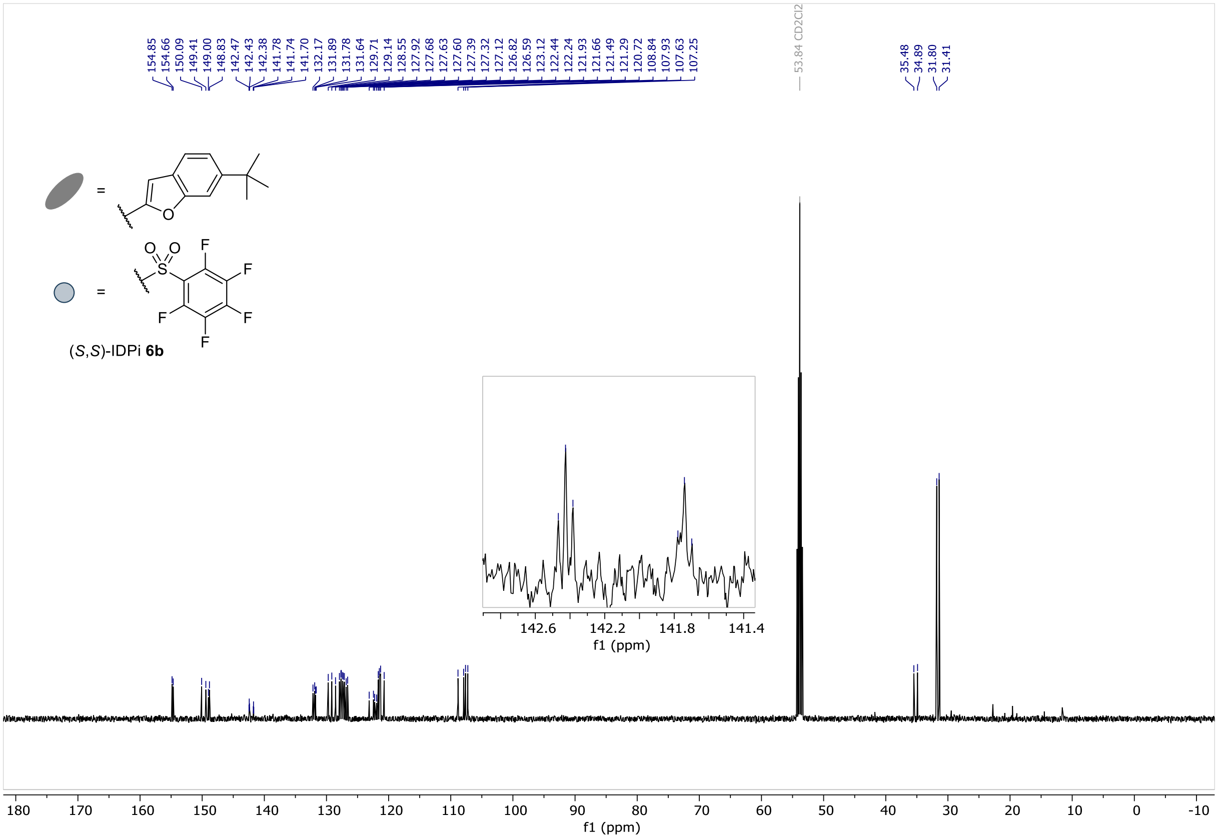

^{13}C-NMR spectrum of IDPi **6b.**

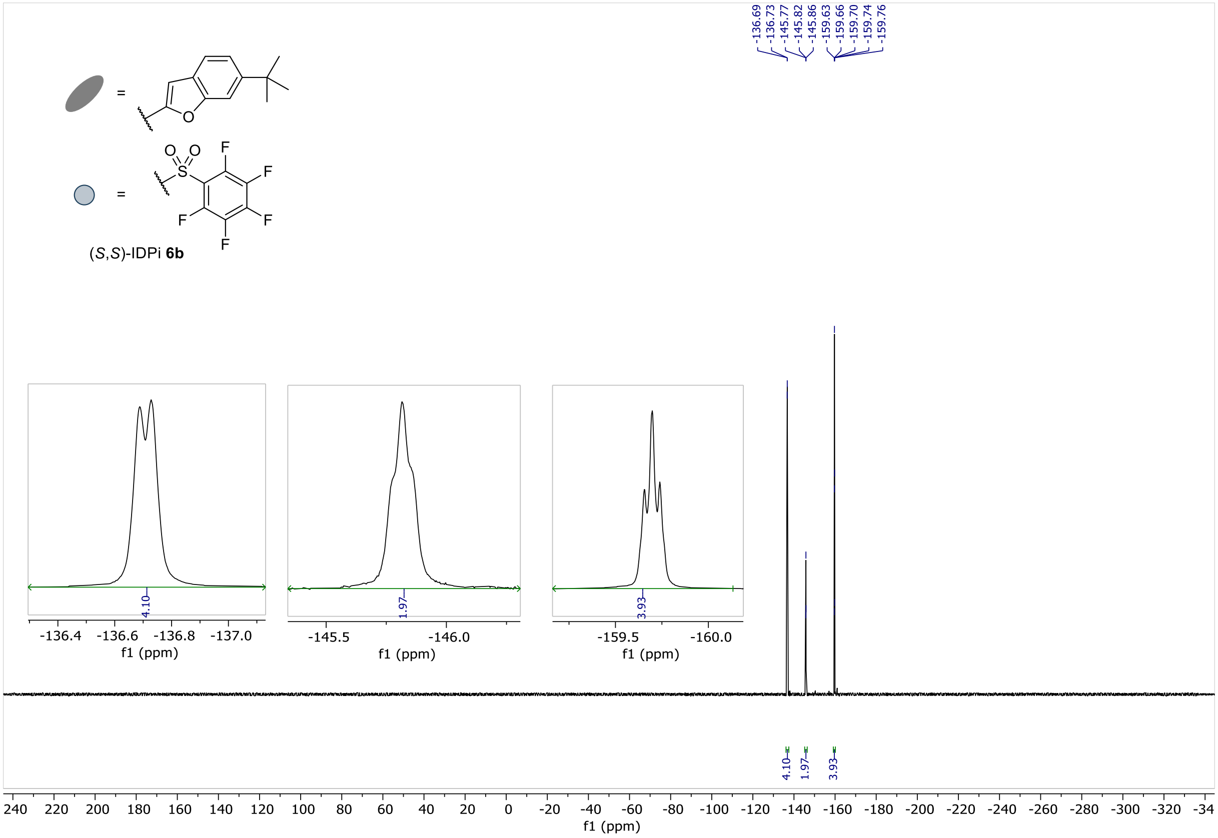

^{19}F-NMR spectrum of IDPi 6**b.**

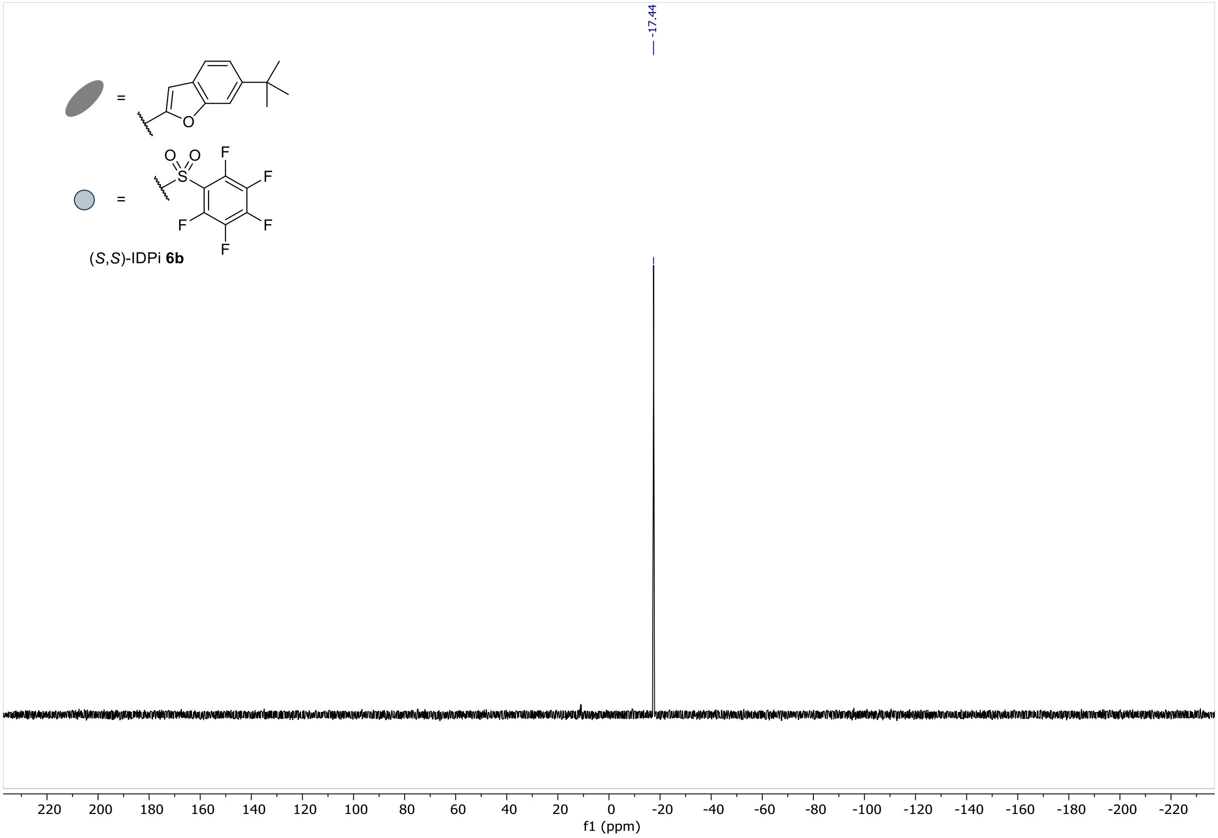

^{31}P-NMR spectrum of IDPi **6b.**

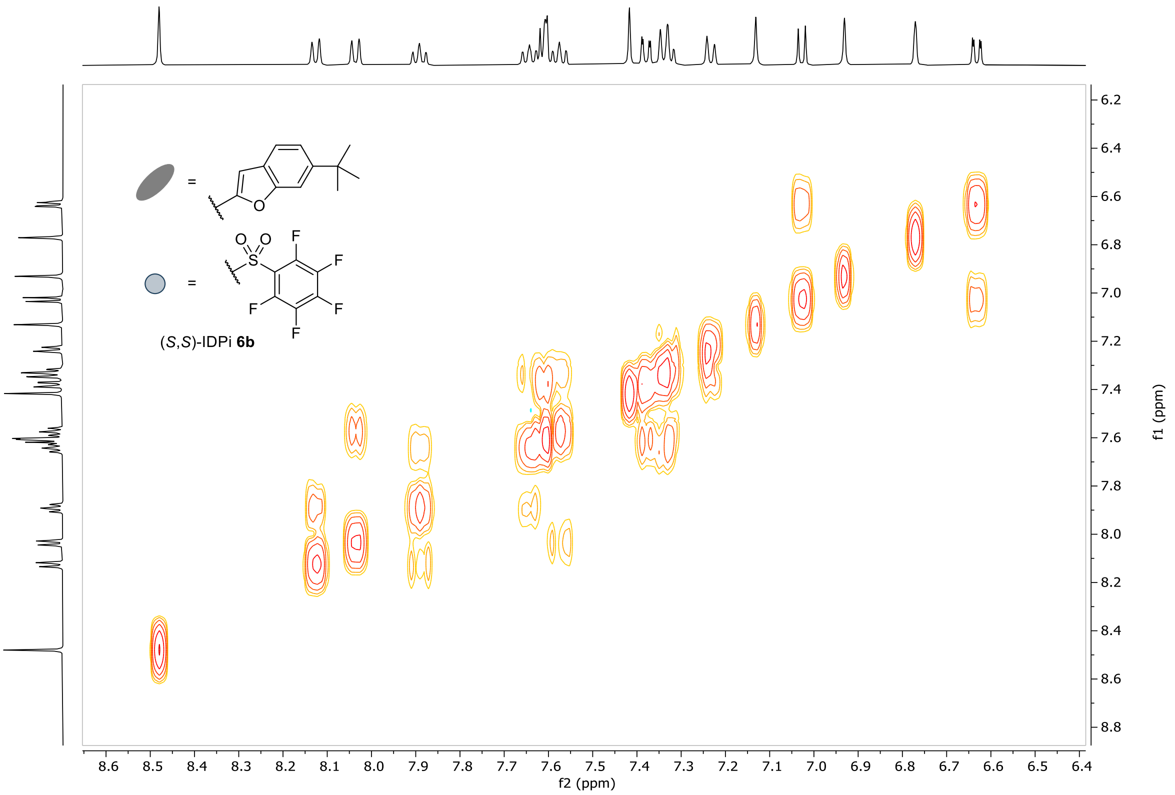

^{1}H-COSY NMR-spectrum of IDPi **6b.**

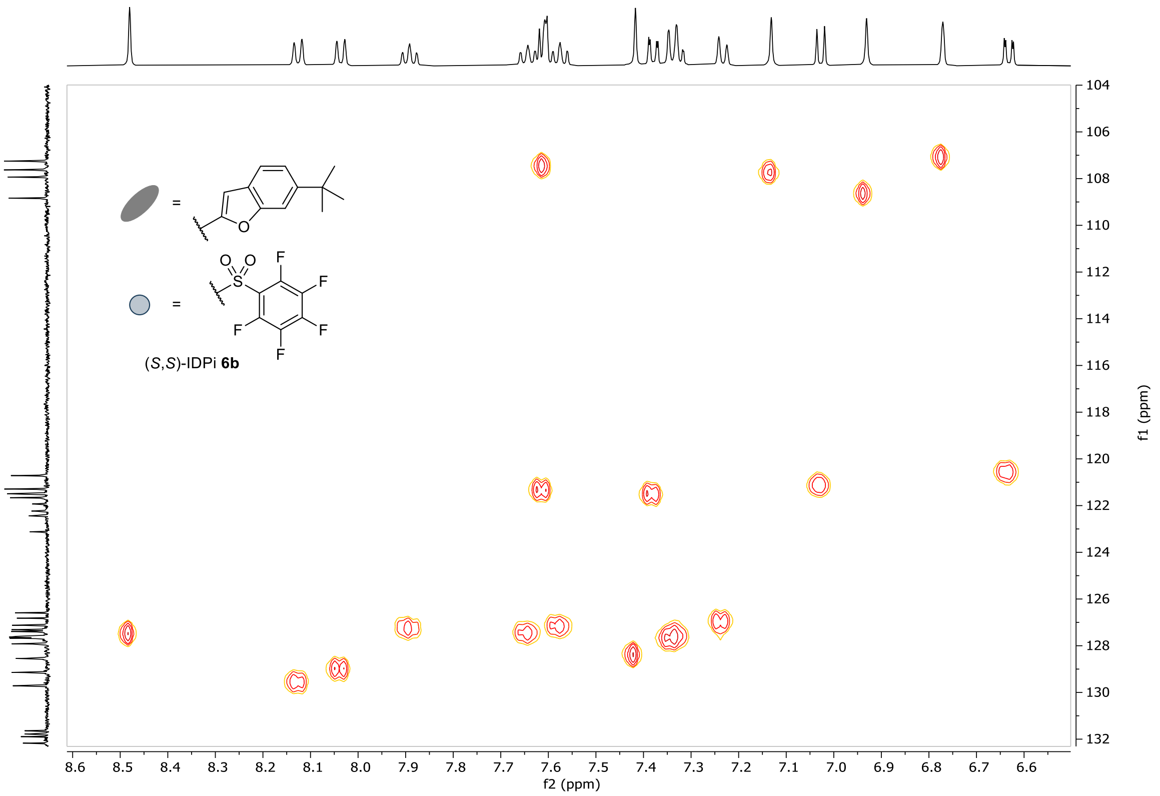

^{1}H-^{13}C-HSQC NMR-spectrum of IDPi **6b.**

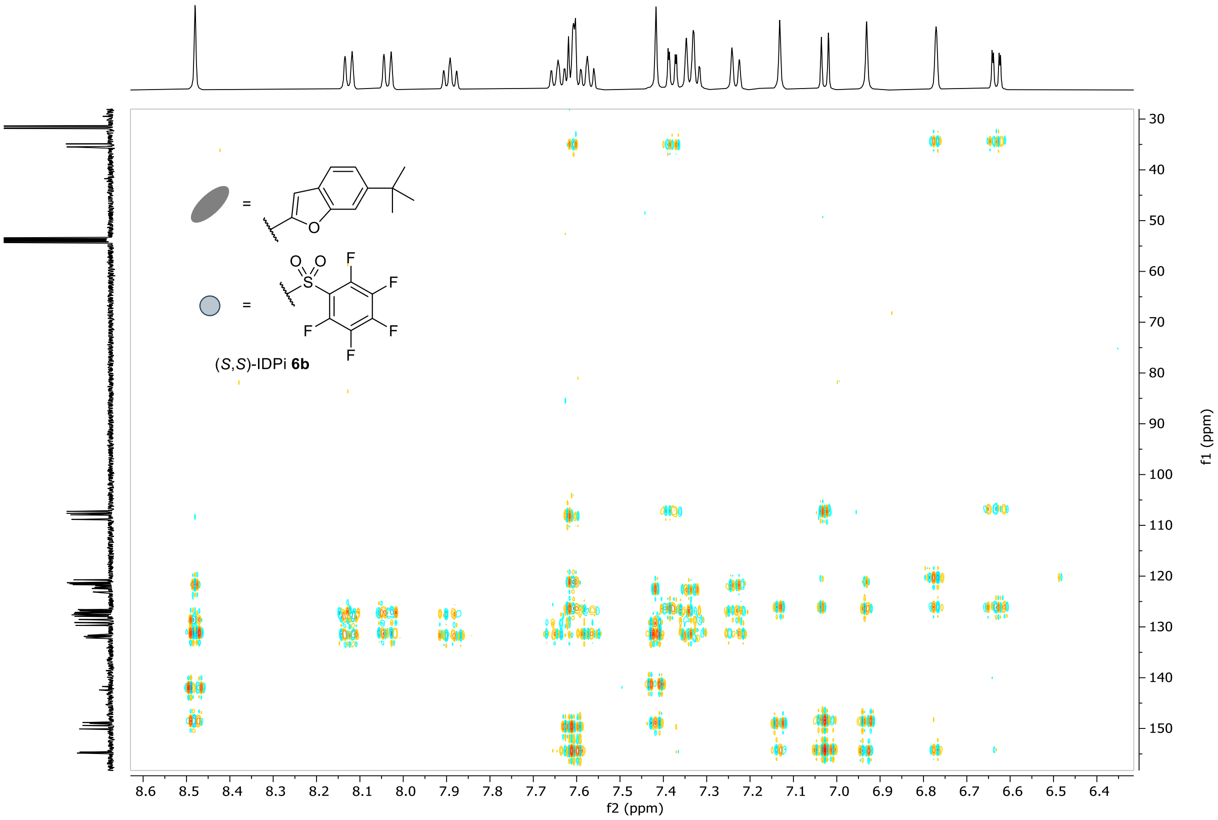

¹H-¹³C-HMBC NMR-spectrum of IDPi **6b.**

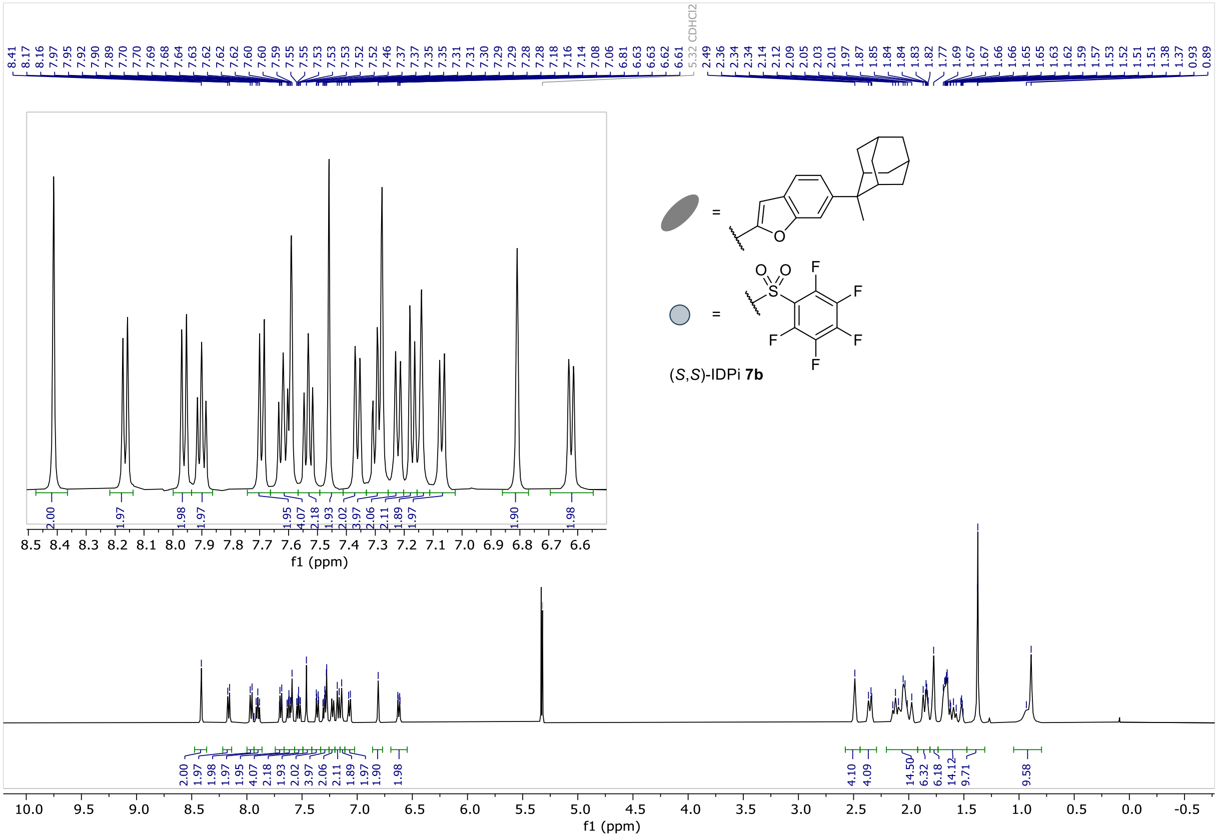

^{1}H-NMR spectrum of IDPi **7b.**

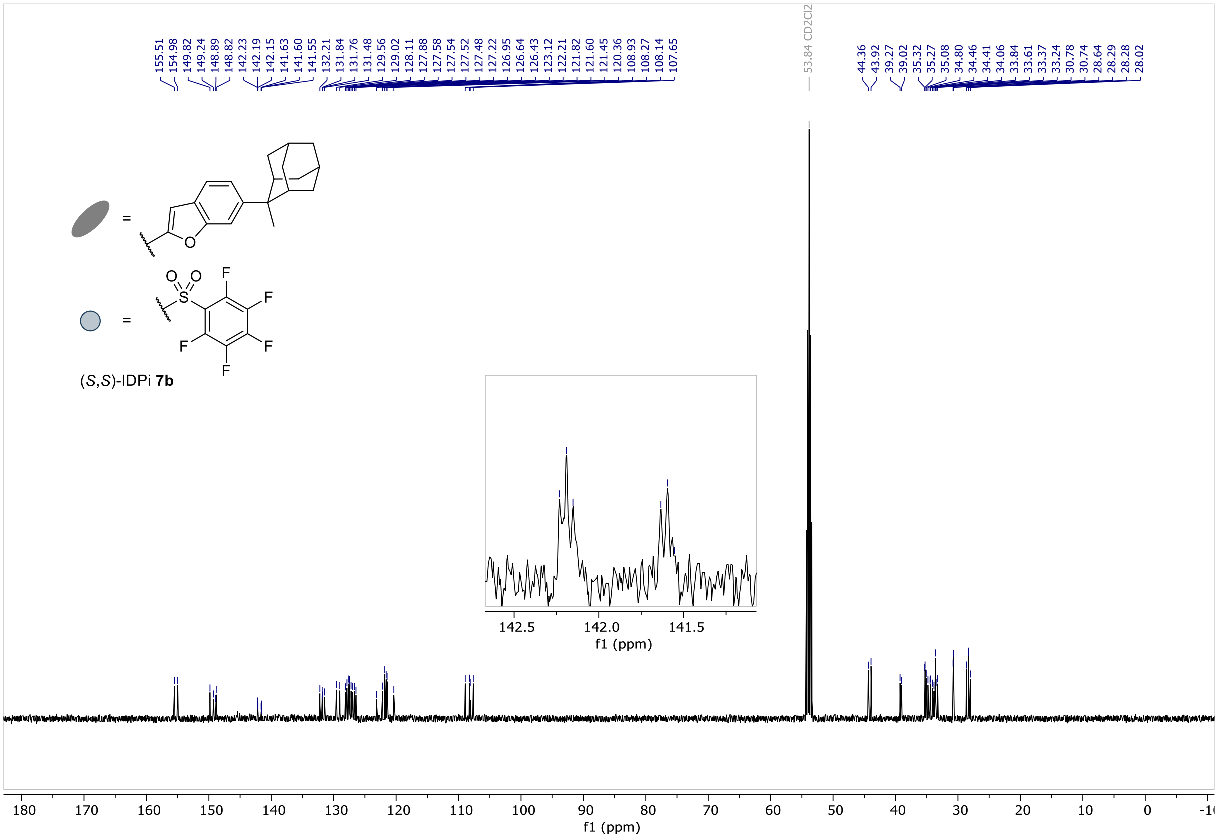

^{13}C-NMR spectrum of IDPi **7b.**

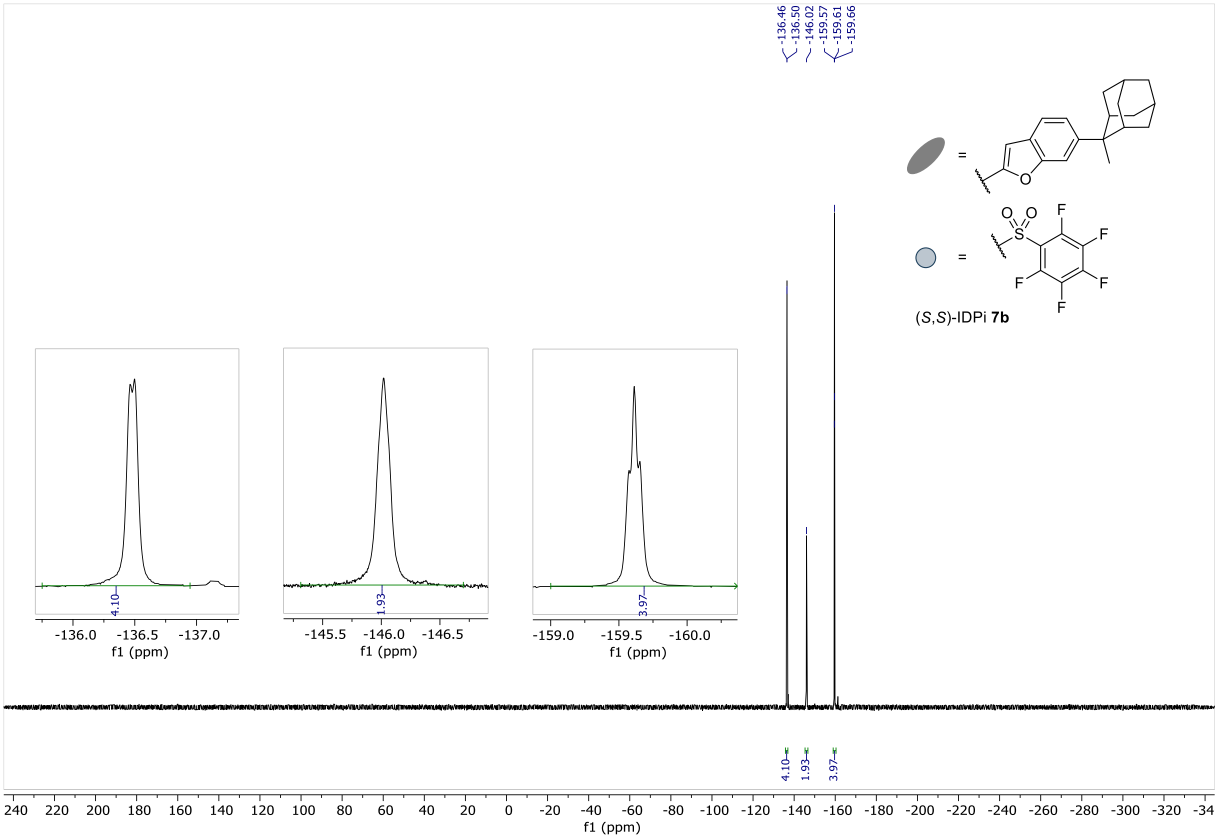

^{19}**F-NMR** spectrum of IDPi **7b.**

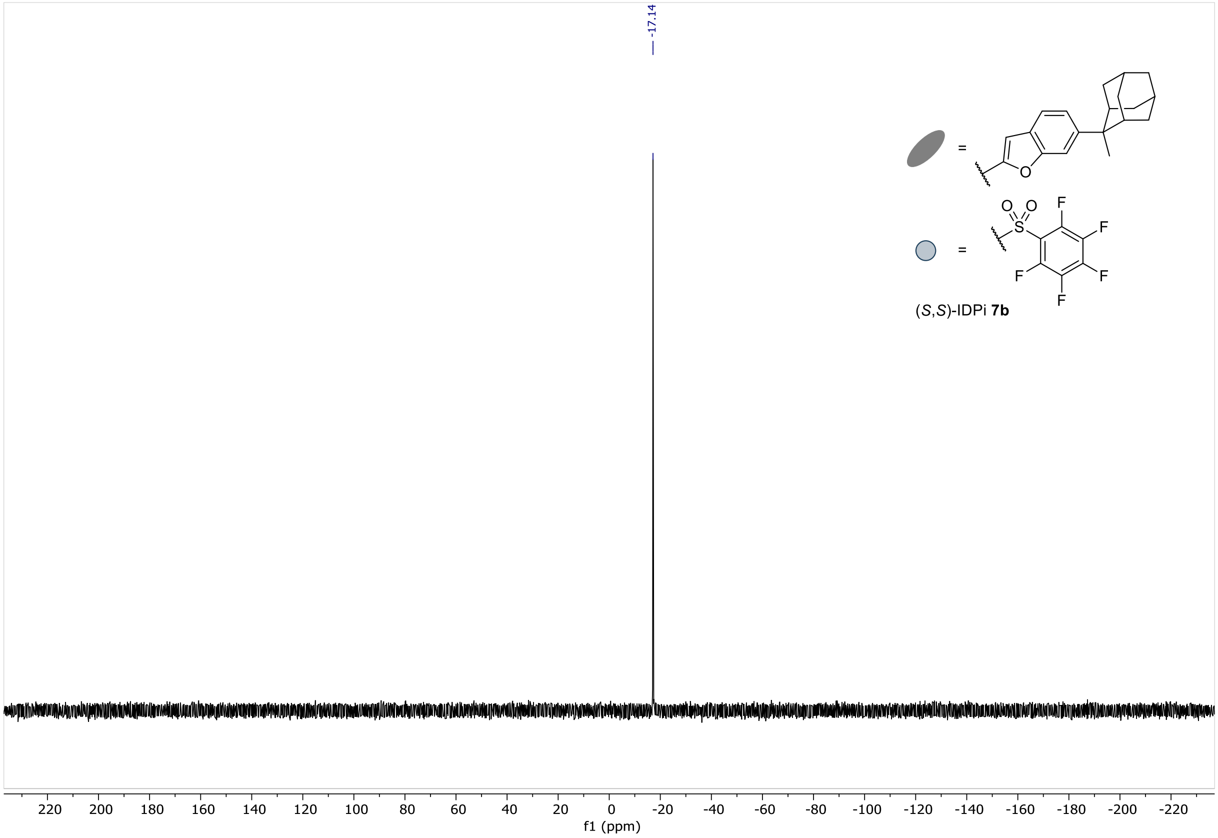

^{31}**P-NMR** spectrum of IDPi **7b.**

9. HPLC Traces

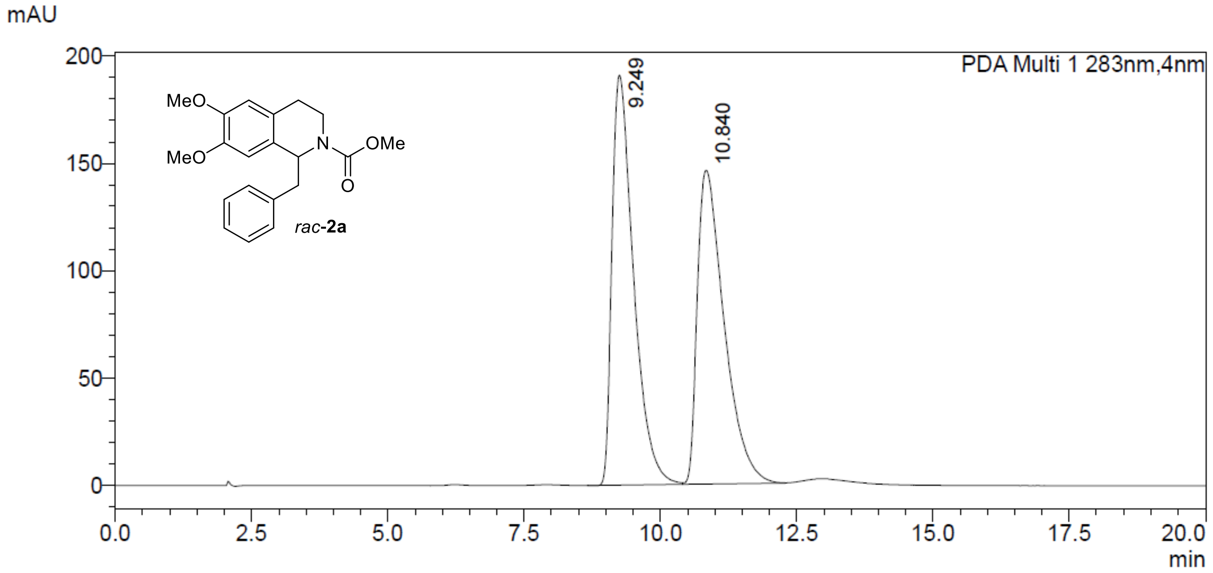

OD-3, *n*-heptane/*i*-PrOH 95:5, 298 K, 283 nm		
**peak #**	**t_R / min**	**area / %**
1	9.249	51.207
2	10.840	48.793

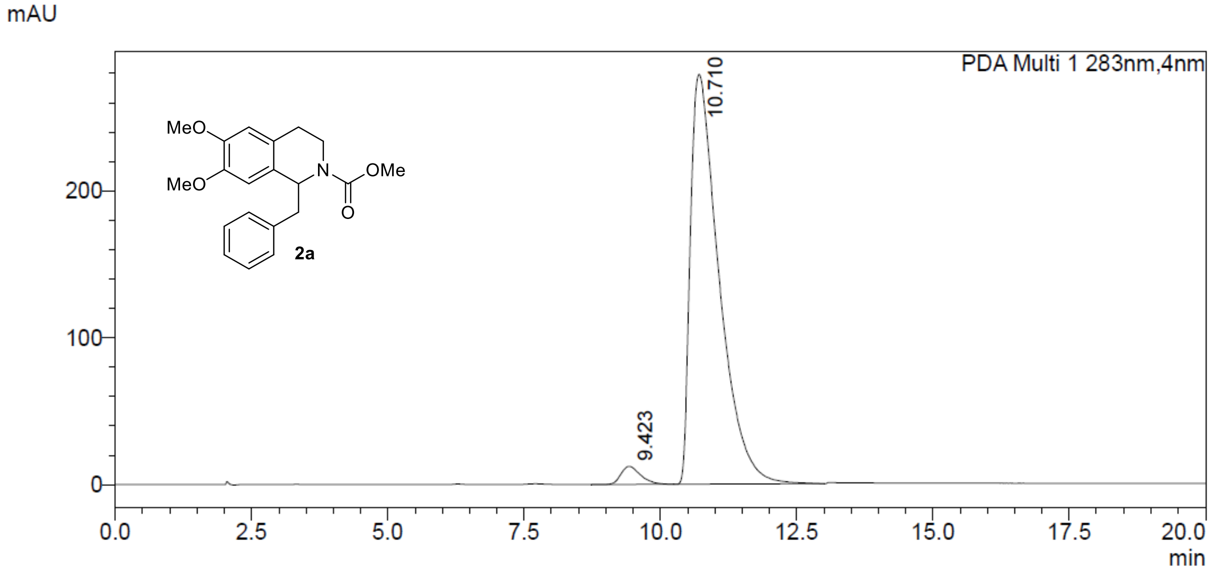

OD-3, *n*-heptane/*i*-PrOH 95:5, 298 K, 283 nm		
**peak #**	**t_R / min**	**area / %**
1	9.423	2.961
2	10.710	97.039

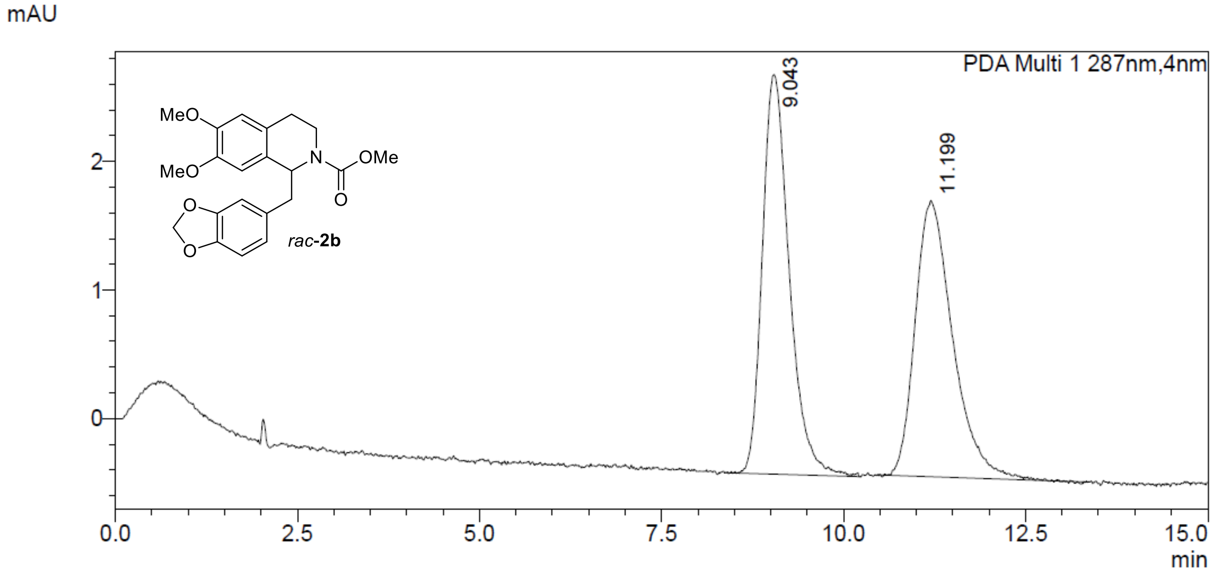

OD-3, *n*-heptane/*i*-PrOH 90:10, 298 K, 287 nm		
**peak #**	t_R **/ min**	**area / %**
1	9.043	50.922
2	11.199	49.078

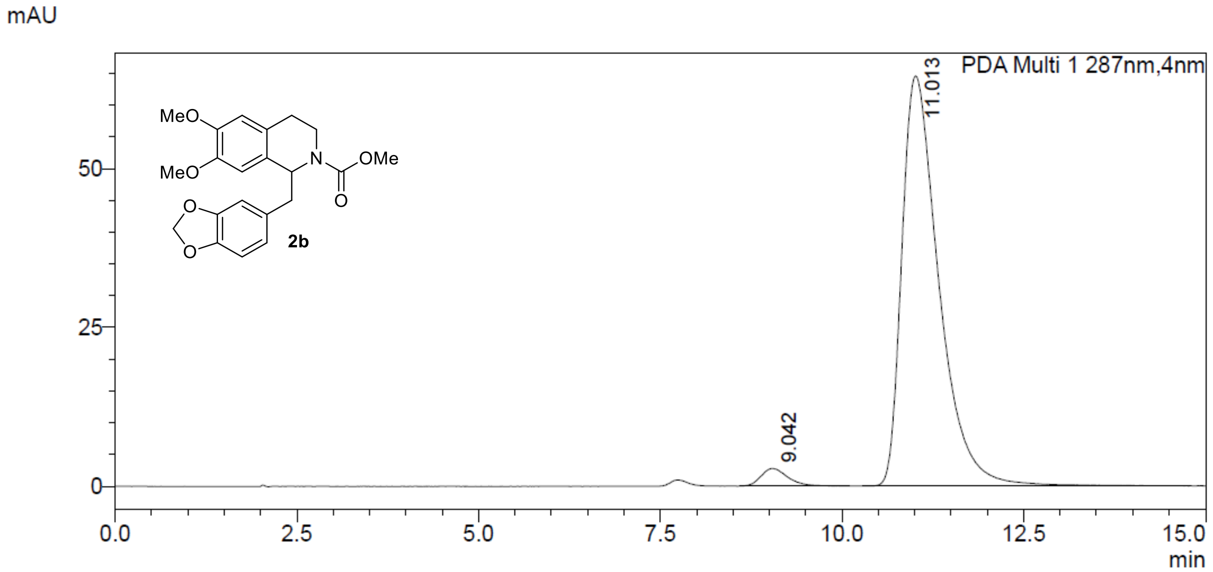

OD-3, *n*-heptane/*i*-PrOH 90:10, 298 K, 287 nm		
**peak #**	t_R **/ min**	**area / %**
1	9.042	2.983
2	11.013	97.017

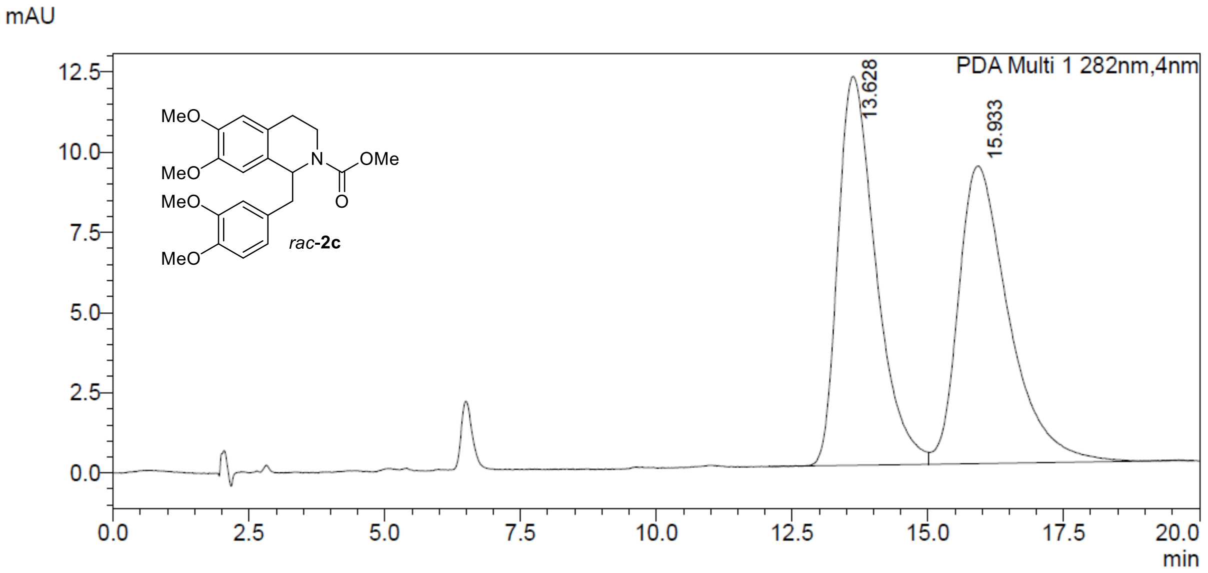

OD-3, *n*-heptane/*i*-PrOH 90:10, 298 K, 282 nm		
**peak #**	t_R **/ min**	**area / %**
1	13.628	49.520
2	15.933	50.480

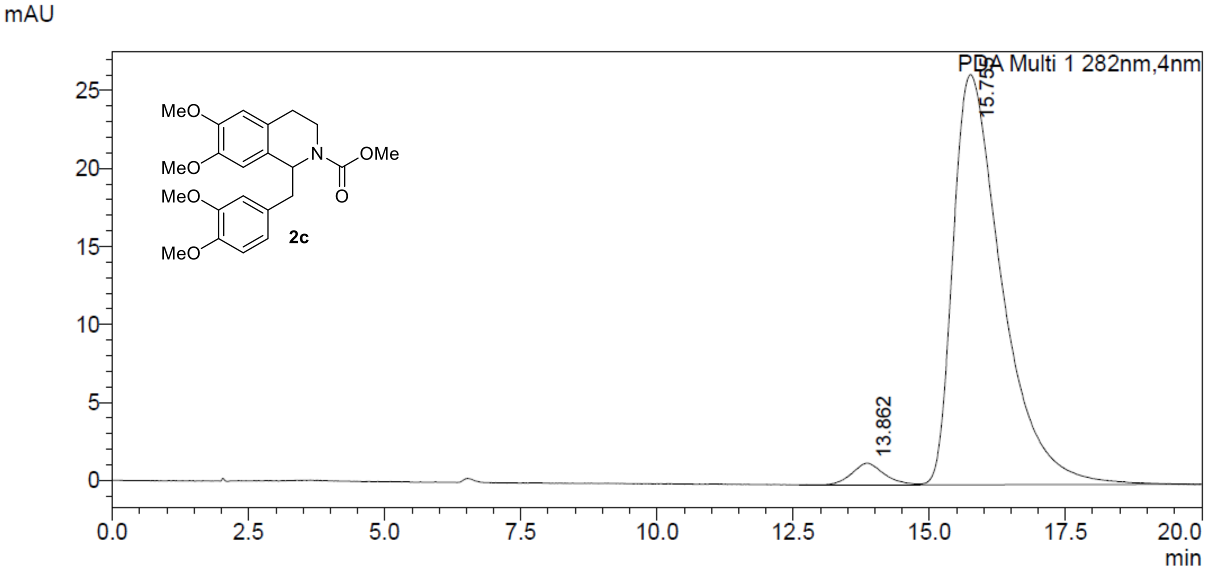

OD-3, *n*-heptane/*i*-PrOH 90:10, 298 K, 282 nm		
**peak #**	t_R **/ min**	**area / %**
1	13.862	3.349
2	15.755	96.651

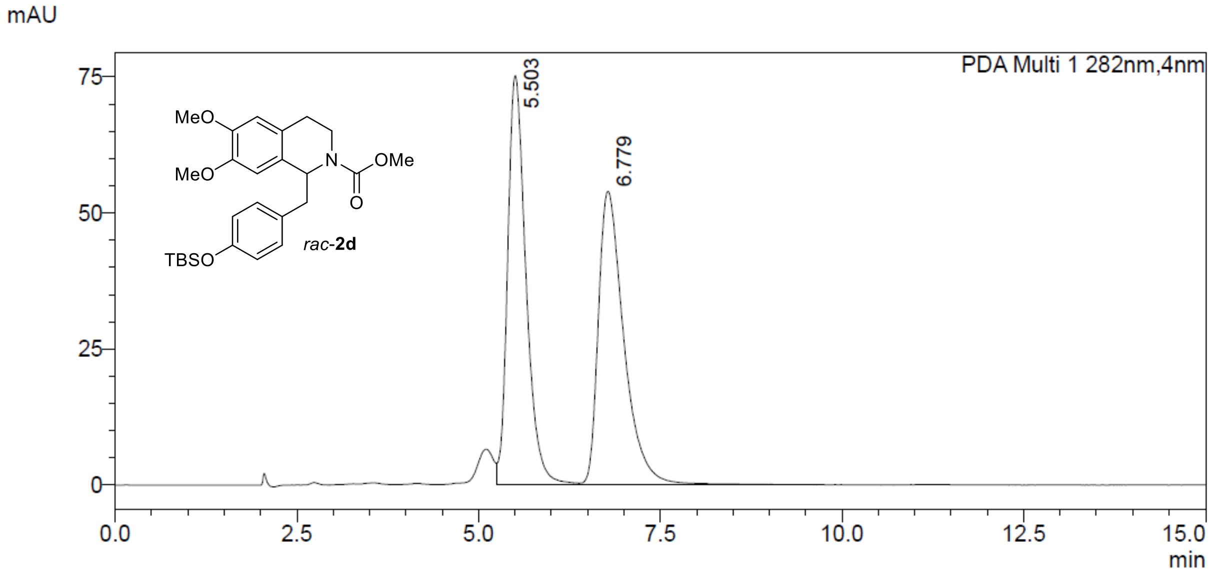

OD-3, *n*-heptane/*i*-PrOH 95:5, 298 K, 282 nm		
**peak #**	t_R **/ min**	**area / %**
1	5.503	50.047
2	6.779	49.953

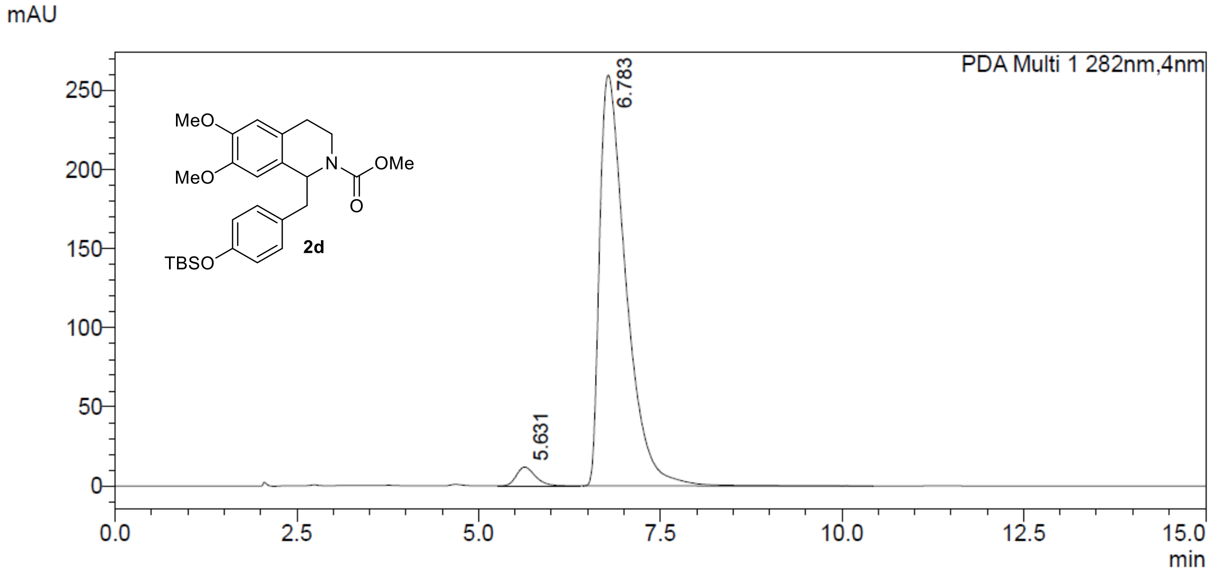

OD-3, *n*-heptane/*i*-PrOH 95:5, 298 K, 282 nm		
**peak #**	t_R **/ min**	**area / %**
1	5.631	3.182
2	6.783	96.818

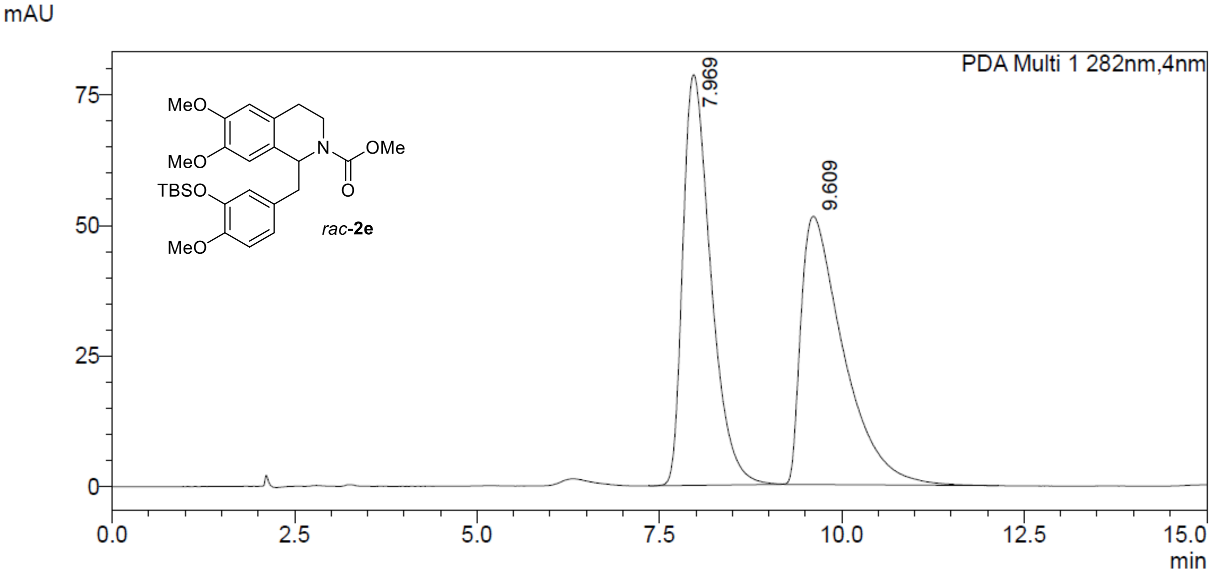

OD-3, *n*-heptane/*i*-PrOH 97:3, 298 K, 282 nm		
**peak #**	t_R **/ min**	**area / %**
1	7.969	50.241
2	9.609	49.759

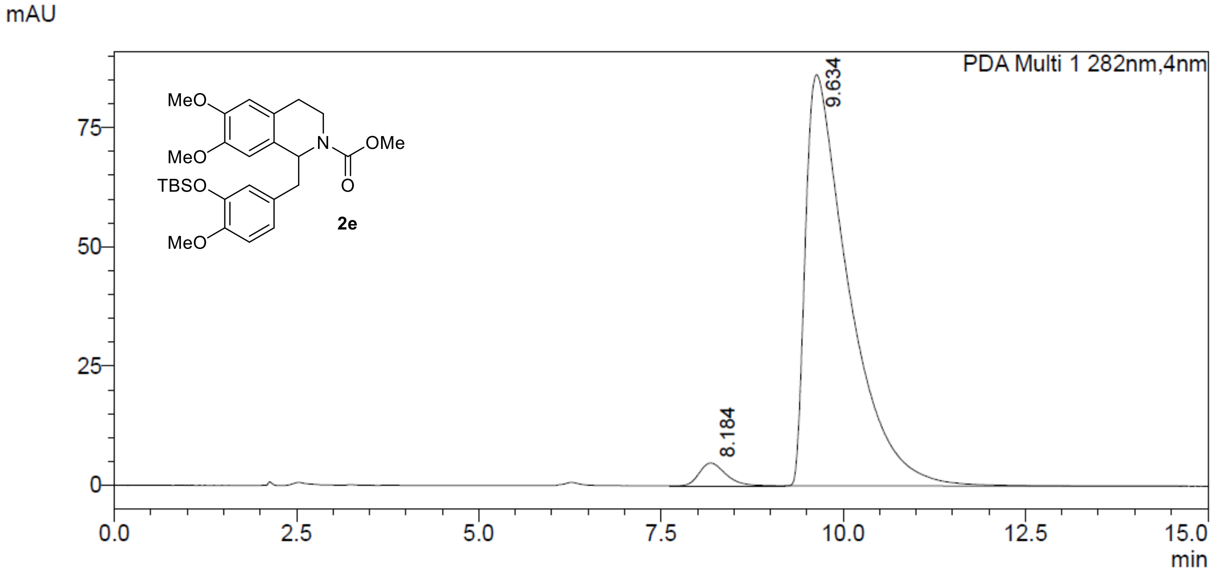

OD-3, *n*-heptane/*i*-PrOH 97:3, 298 K, 282 nm		
**peak #**	t_R **/ min**	**area / %**
1	8.184	3.275
2	9.634	96.725

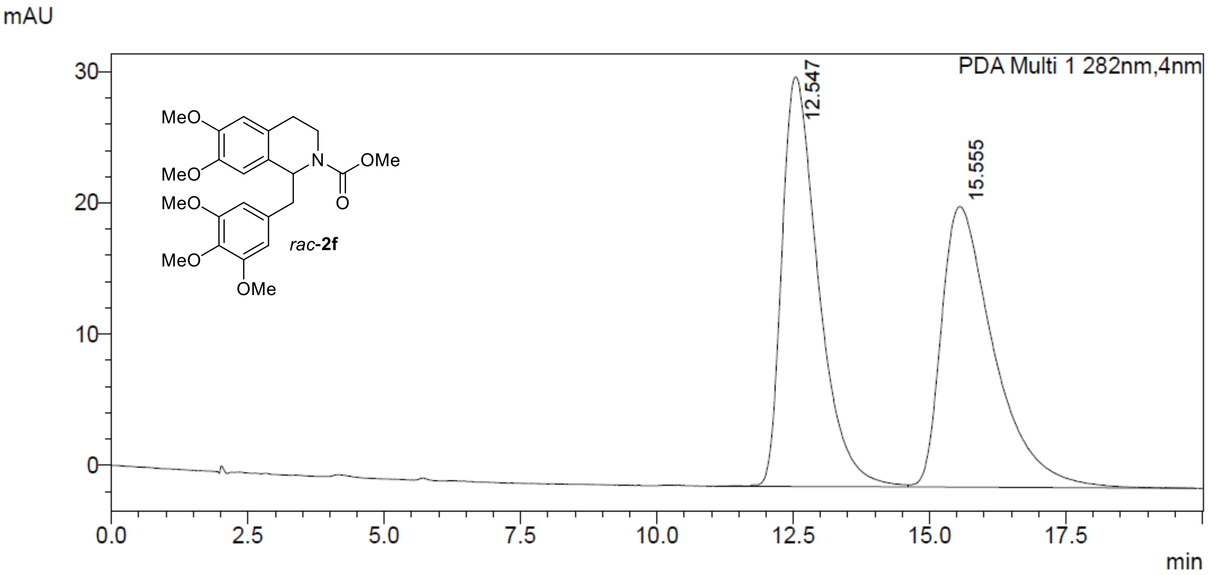

OD-3, *n*-heptane/*i*-PrOH 90:10, 298 K, 282 nm		
**peak #**	**t_R / min**	**area / %**
1	12.547	50.340
2	15.555	49.660

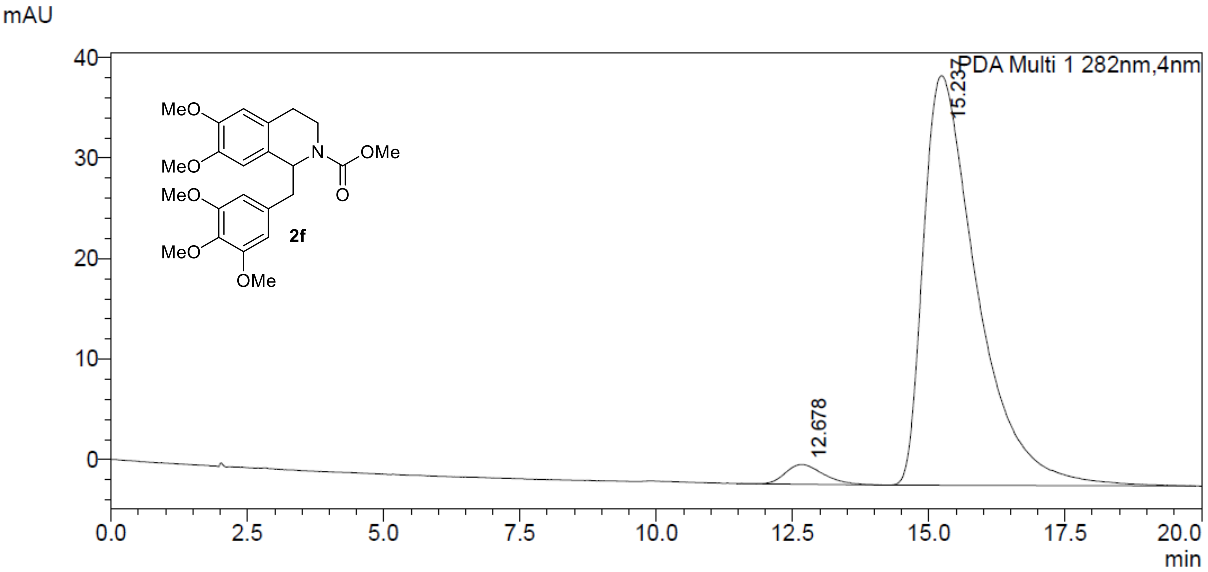

OD-3, *n*-heptane/*i*-PrOH 90:10, 298 K, 282 nm		
**peak #**	**t_R / min**	**area / %**
1	12.678	3.304
2	15.237	96.696

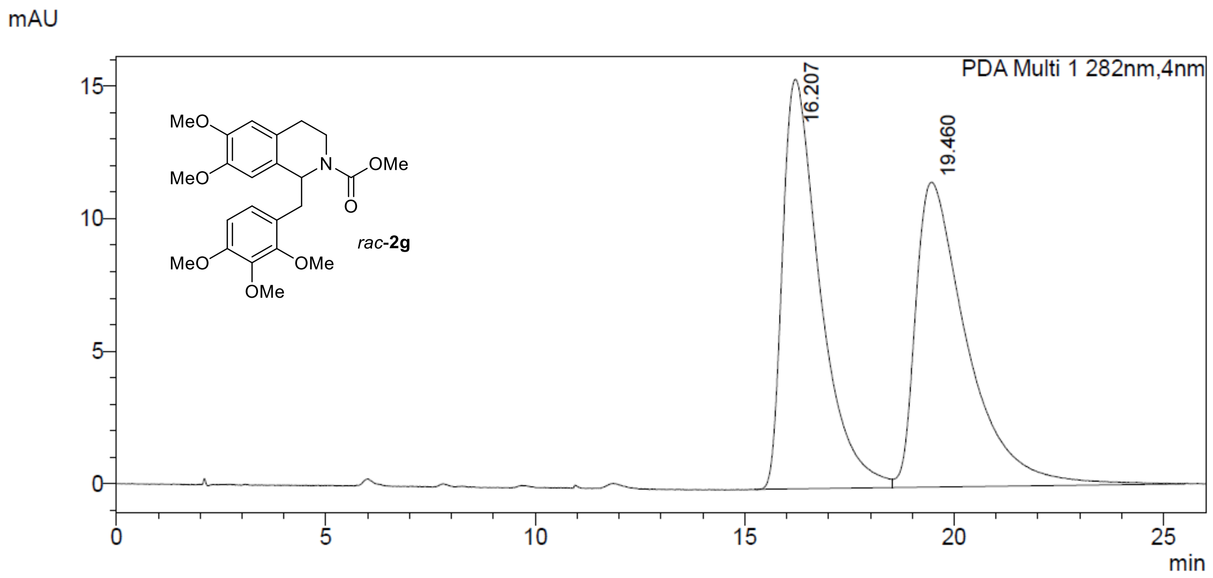

OD-3, *n*-heptane/*i*-PrOH 95:5, 298 K, 282 nm		
**peak #**	t_R **/ min**	**area / %**
1	16.207	49.321
2	19.460	50.679

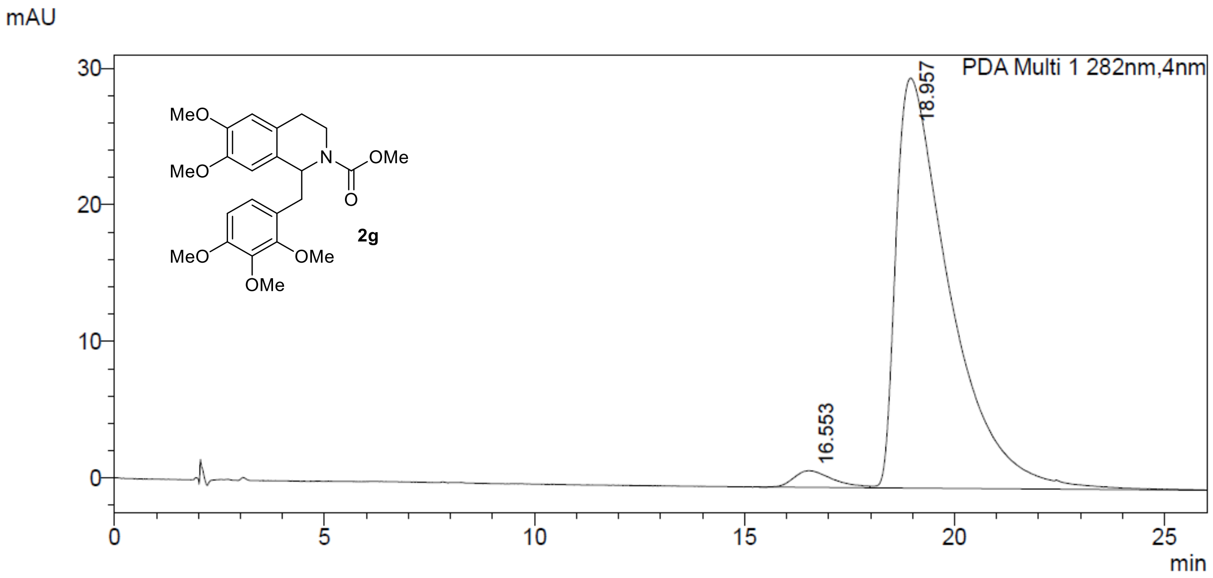

OD-3, *n*-heptane/*i*-PrOH 95:5, 298 K, 282 nm		
**peak #**	t_R **/ min**	**area / %**
1	16.553	2.845
2	18.957	97.155

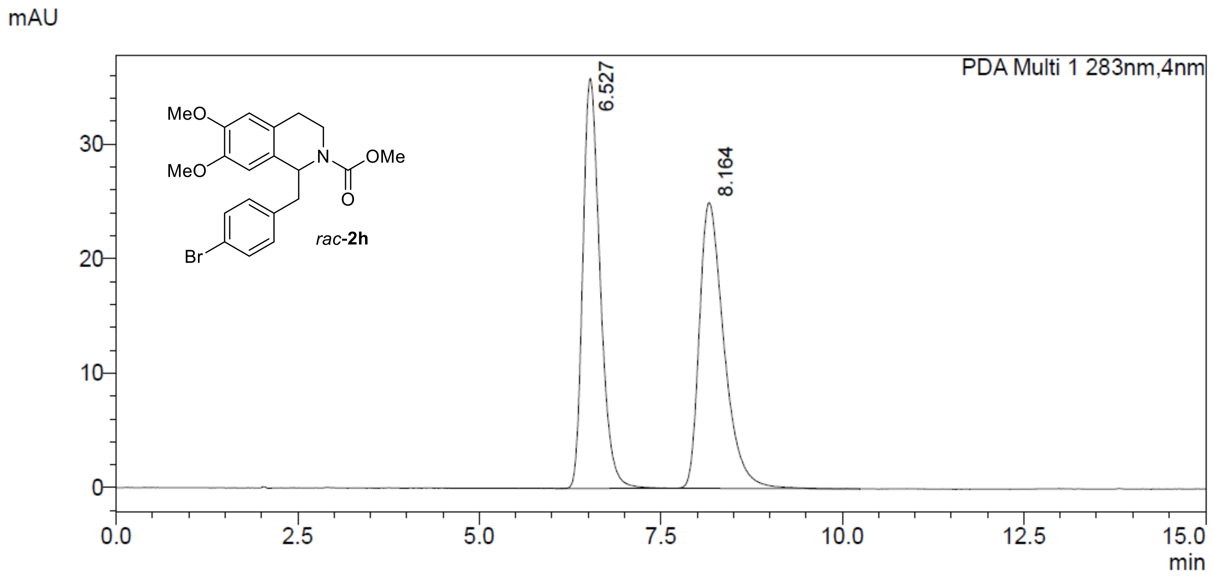

OD-3, *n*-heptane/*i*-PrOH 90:10, 298 K, 283 nm		
**peak #**	t_R **/ min**	**area / %**
1	6.527	50.112
2	8.164	49.888

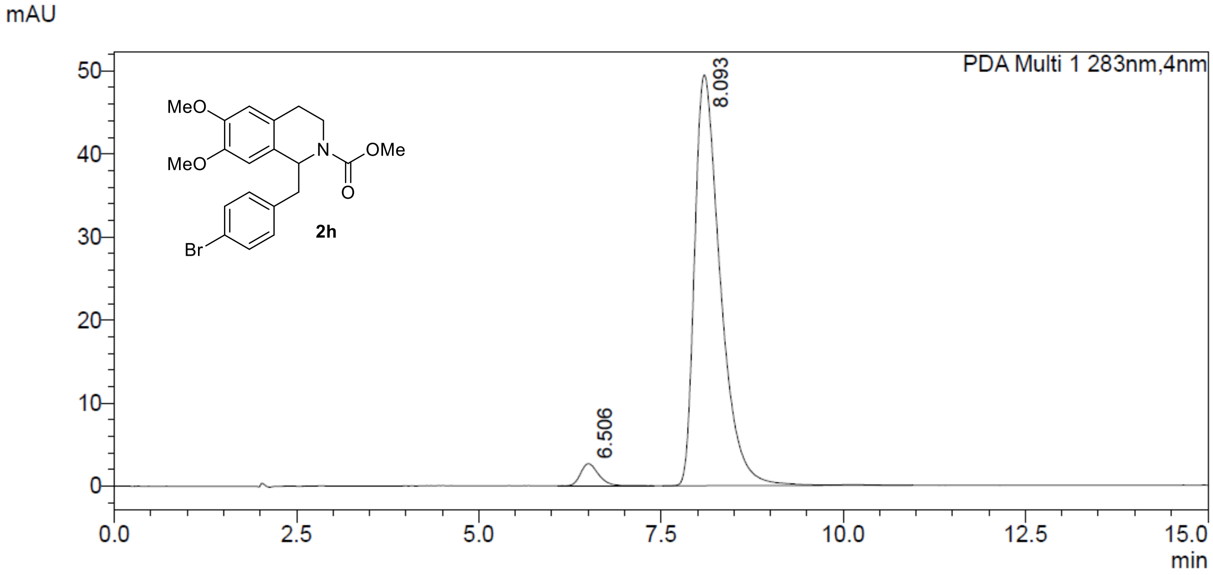

OD-3, *n*-heptane/*i*-PrOH 90:10, 298 K, 283 nm		
**peak #**	t_R **/ min**	**area / %**
1	6.506	3.623
2	8.093	96.377

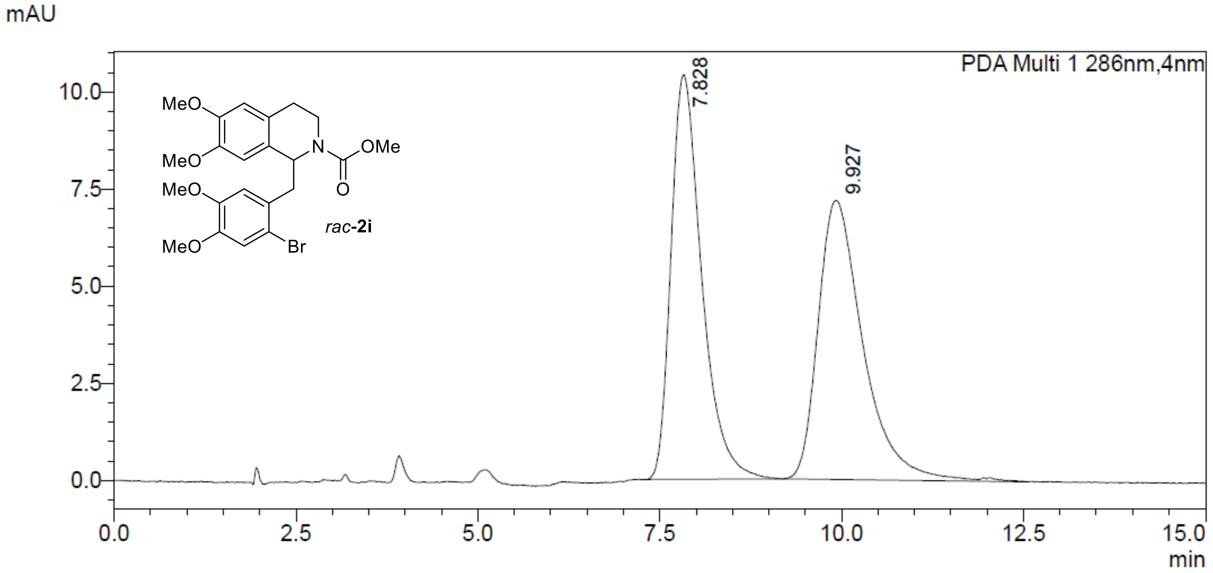

OD-3, *n*-heptane/*i*-PrOH 80:20, 298 K, 286 nm		
**peak #**	t_R **/ min**	**area / %**
1	7.828	49.793
2	9.927	50.207

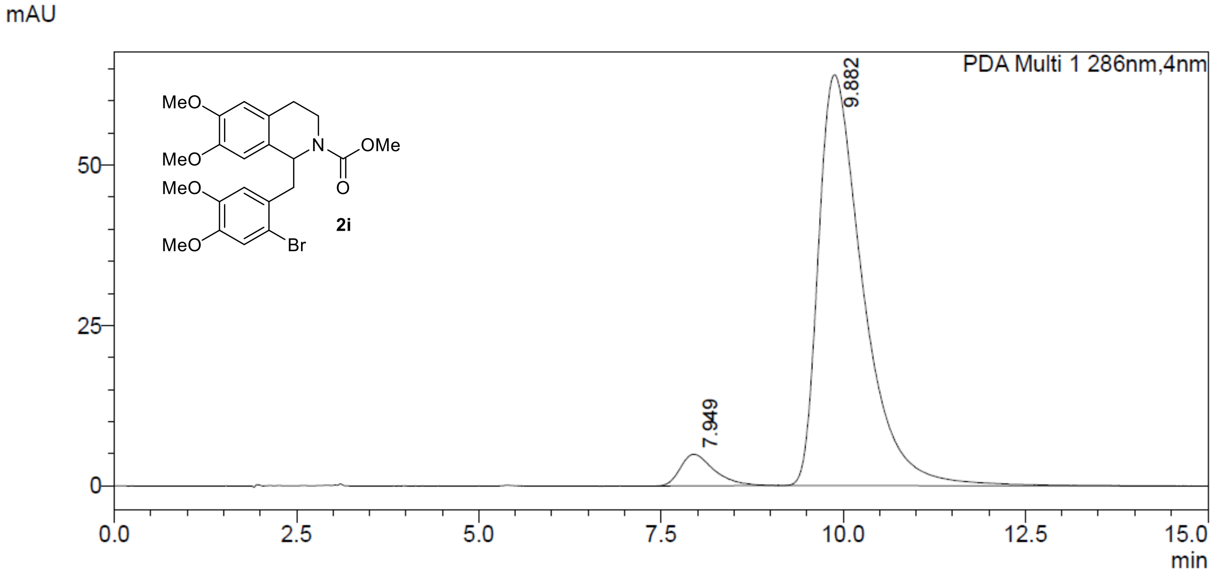

OD-3, *n*-heptane/*i*-PrOH 80:20, 298 K, 286 nm		
**peak #**	t_R **/ min**	**area / %**
1	7.949	5.534
2	9.882	94.466

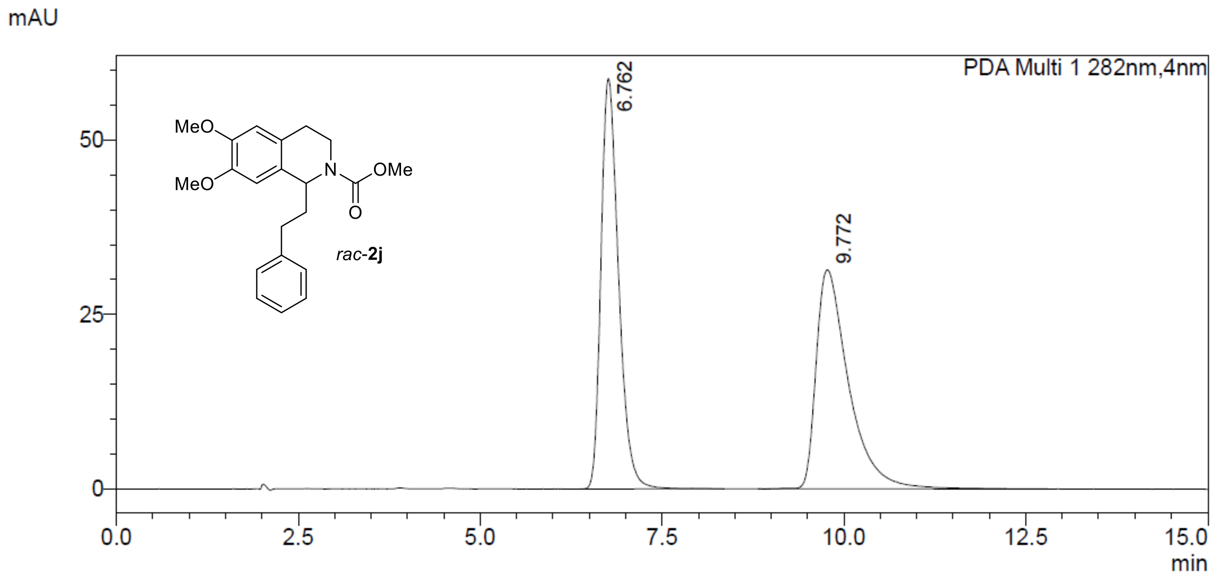

OD-3, *n*-heptane/*i*-PrOH 90:10, 298 K, 282 nm		
**peak #**	t_R **/ min**	**area / %**
1	6.762	50.241
2	9.772	49.759

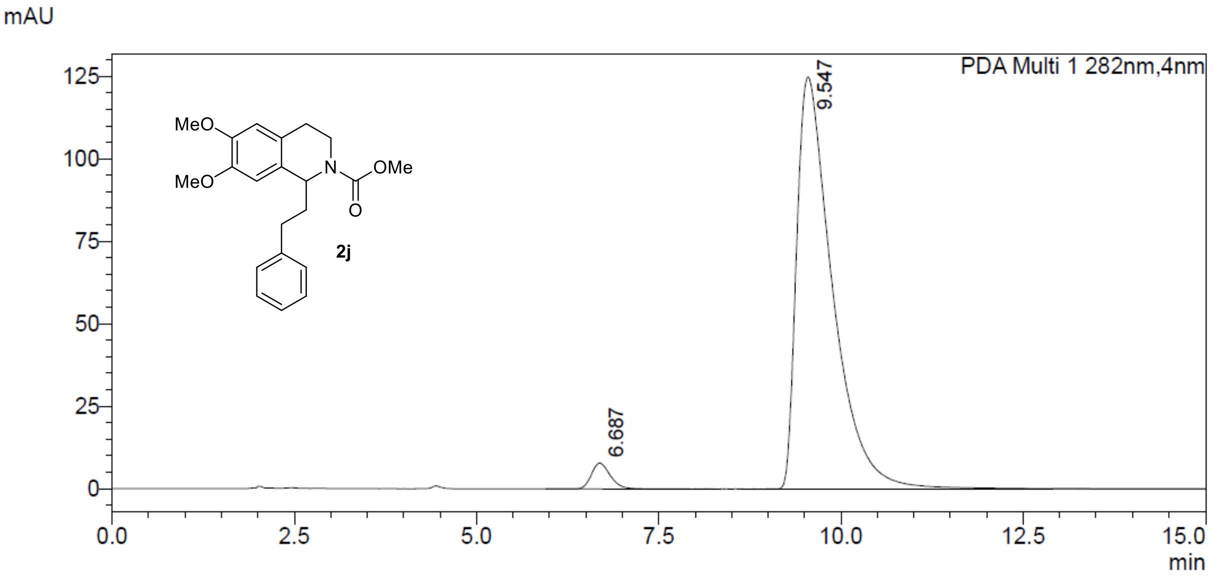

OD-3, *n*-heptane/*i*-PrOH 90:10, 298 K, 282 nm		
**peak #**	t_R **/ min**	**area / %**
1	6.687	3.235
2	9.547	96.765

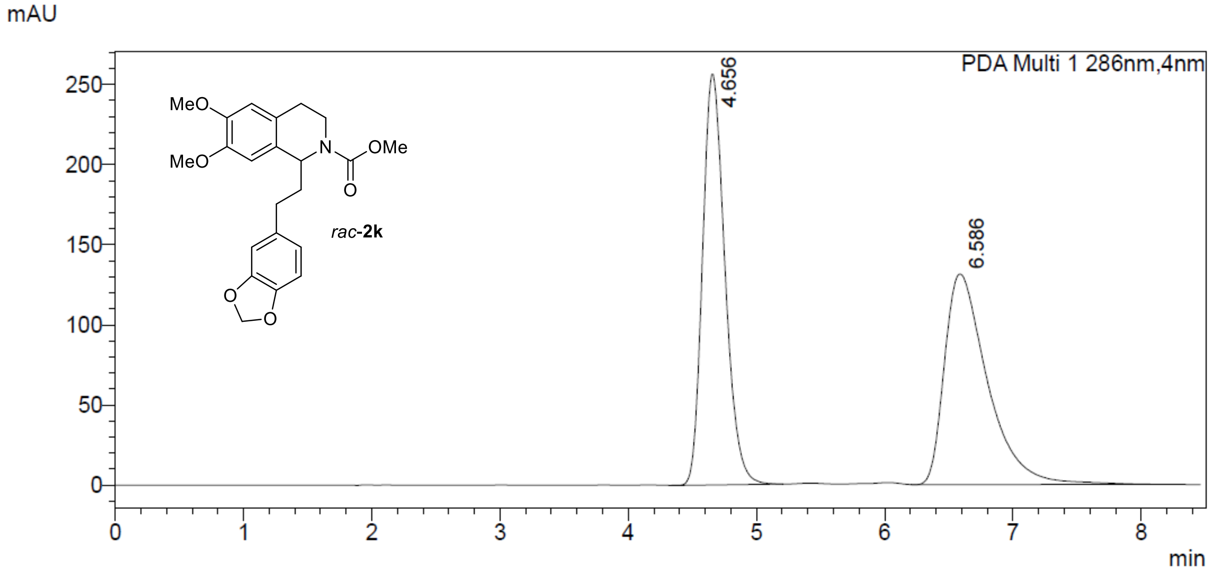

OD-3, *n*-heptane/*i*-PrOH 70:30, 298 K, 286 nm		
**peak #**	t_R **/ min**	**area / %**
1	4.656	50.274
2	6.586	49.726

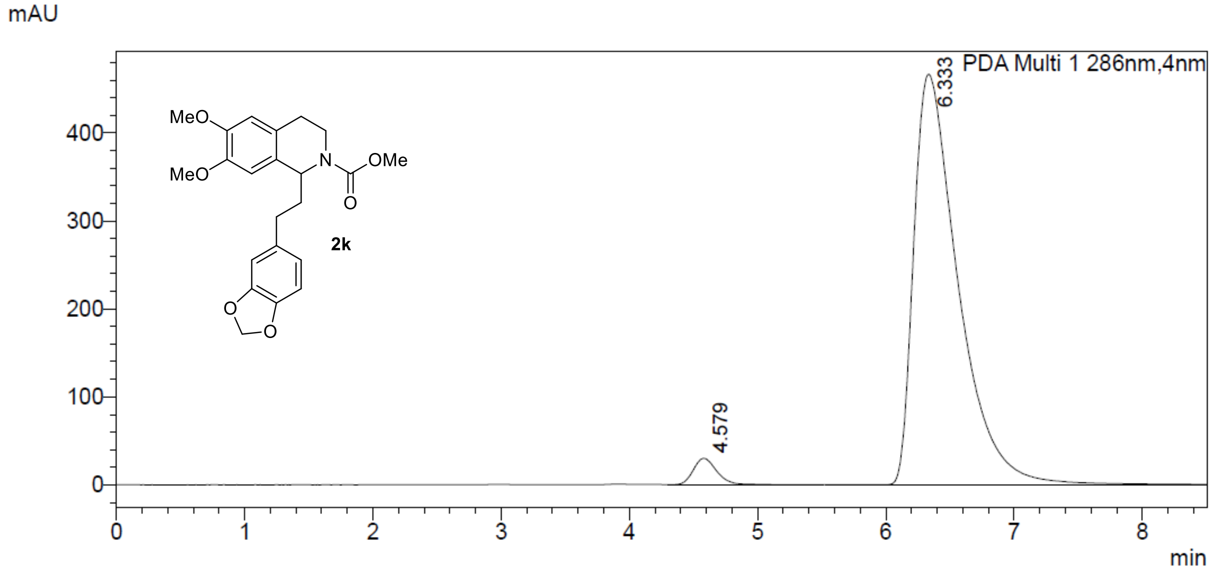

OD-3, *n*-heptane/*i*-PrOH 70:30, 298 K, 286 nm		
**peak #**	t_R **/ min**	**area / %**
1	4.579	3.297
2	6.333	96.703

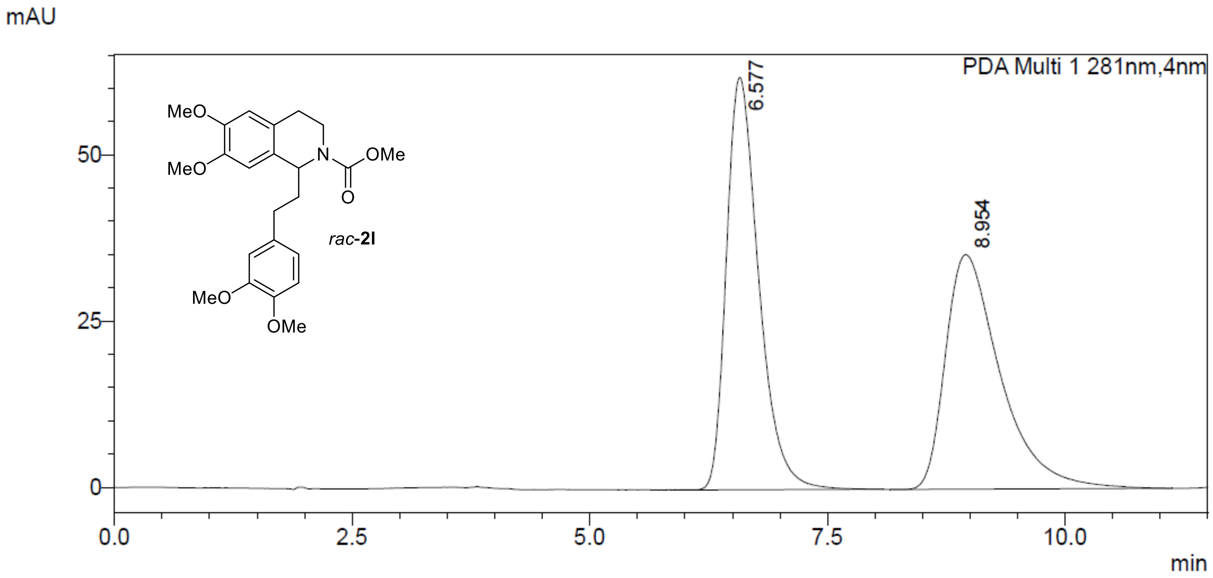

OD-3, *n*-heptane/*i*-PrOH 70:30, 298 K, 281 nm		
**peak #**	t_R **/ min**	**area / %**
1	6.577	50.896
2	8.954	49.104

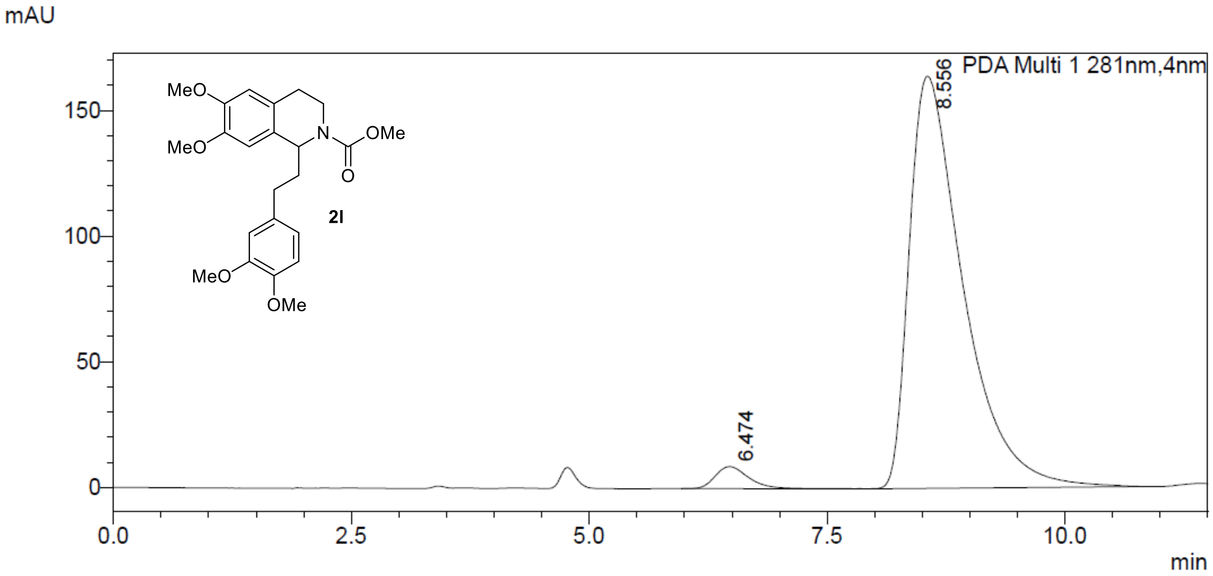

OD-3, *n*-heptane/*i*-PrOH 70:30, 298 K, 281 nm		
**peak #**	t_R **/ min**	**area / %**
1	6.474	3.226
2	8.556	96.774

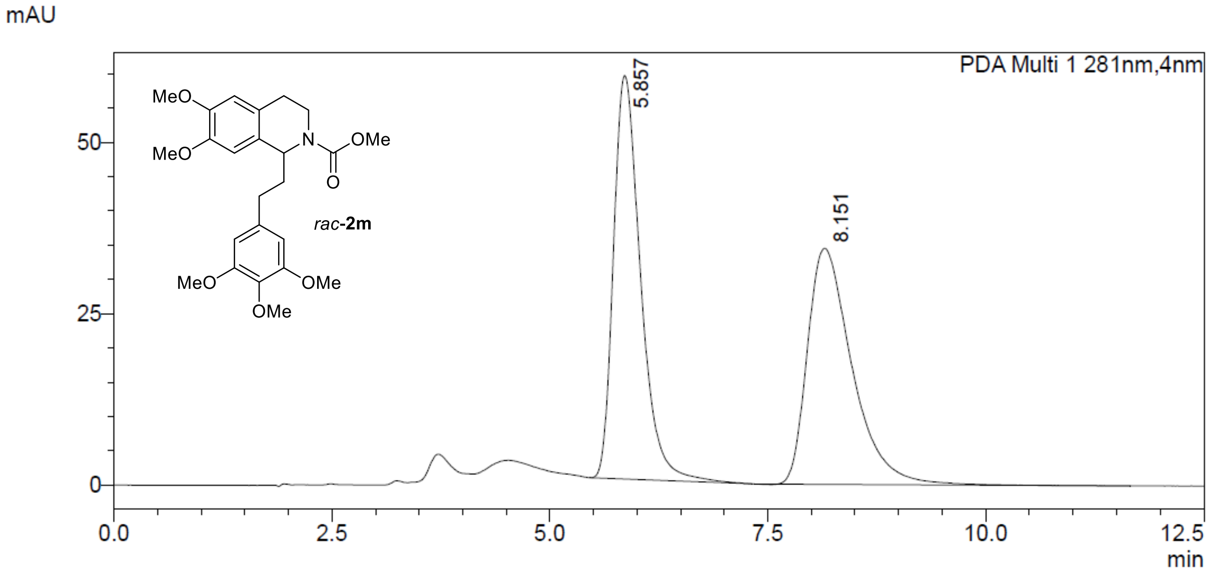

OD-3, *n*-heptane/*i*-PrOH 70:30, 298 K, 281 nm		
**peak #**	t_R **/ min**	**area / %**
1	5.857	50.902
2	8.151	49.098

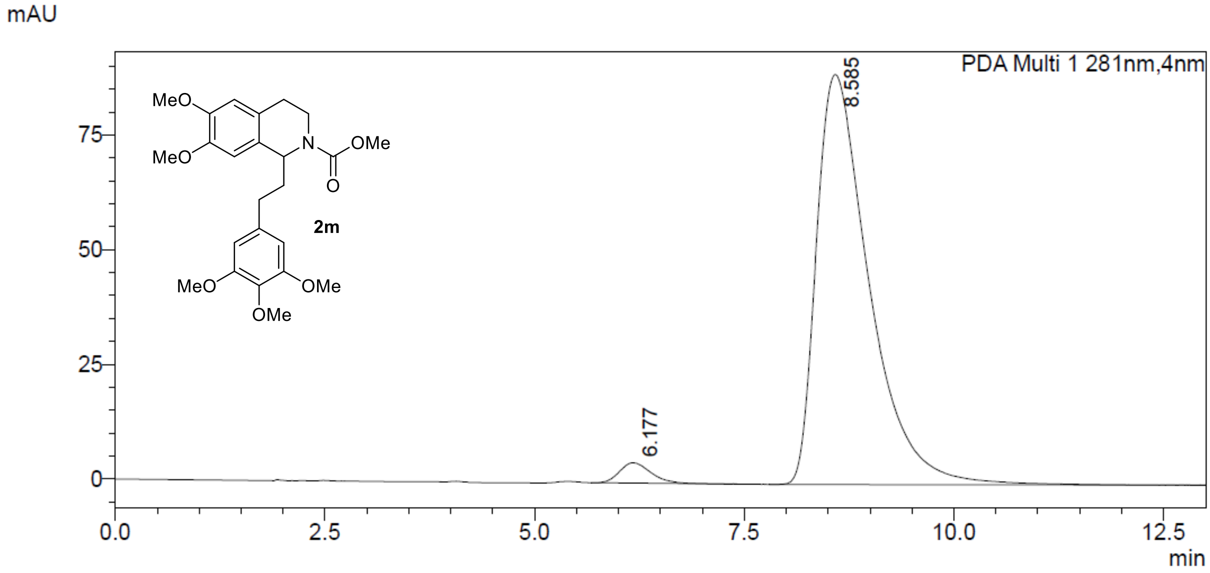

OD-3, *n*-heptane/*i*-PrOH 70:30, 298 K, 281 nm		
**peak #**	t_R **/ min**	**area / %**
1	6.177	2.903
2	8.585	97.097

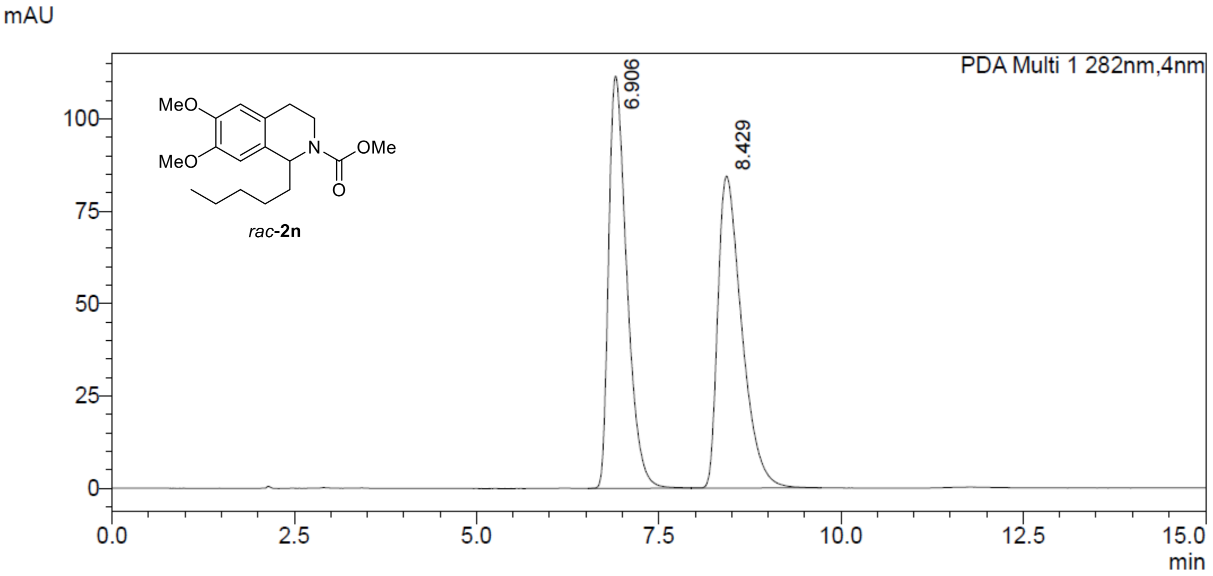

OD-3, *n*-heptane/*i*-PrOH 97:3, 298 K, 282 nm		
**peak #**	***t*$_R$ / min**	**area / %**
1	6.906	50.043
2	8.429	49.957

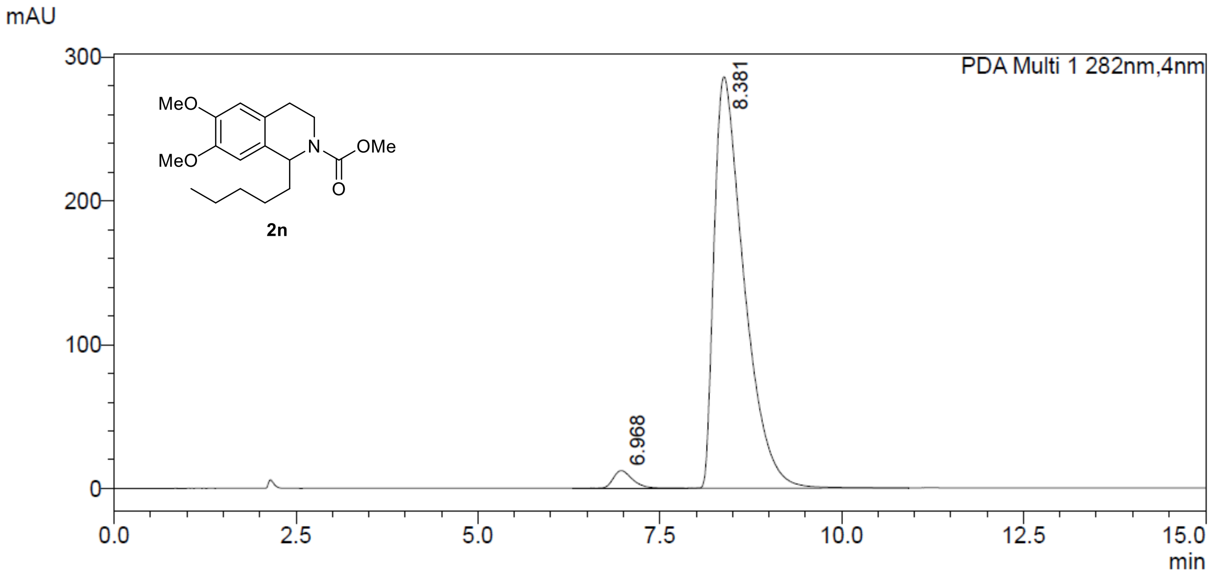

OD-3, *n*-heptane/*i*-PrOH 97:3, 298 K, 282 nm		
**peak #**	***t*$_R$ / min**	**area / %**
1	6.968	2.674
2	8.381	97.326

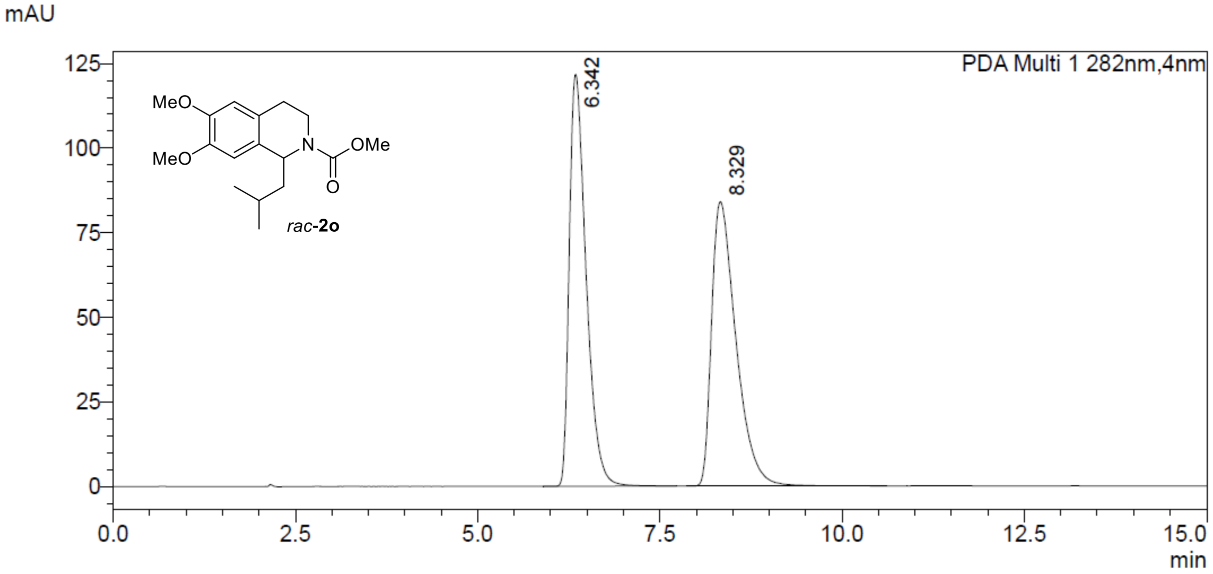

OD-3, *n*-heptane/*i*-PrOH 97:3, 298 K, 282 nm		
**peak #**	t_R **/ min**	**area / %**
1	6.342	49.995
2	8.329	50.005

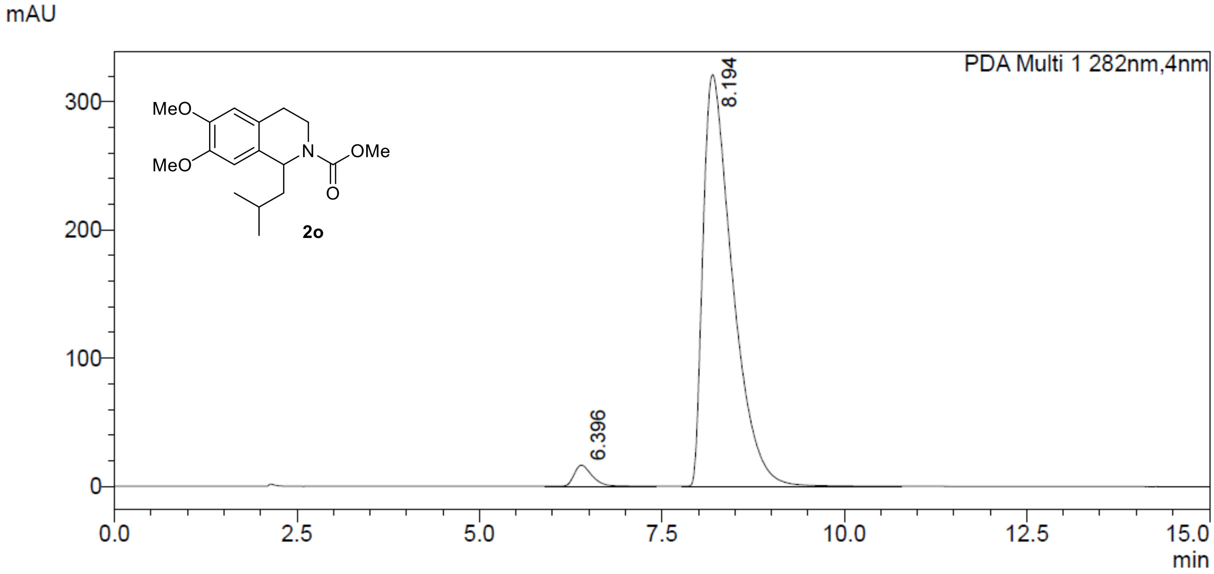

OD-3, *n*-heptane/*i*-PrOH 97:3, 298 K, 282 nm		
**peak #**	t_R **/ min**	**area / %**
1	6.396	2.997
2	8.194	97.003

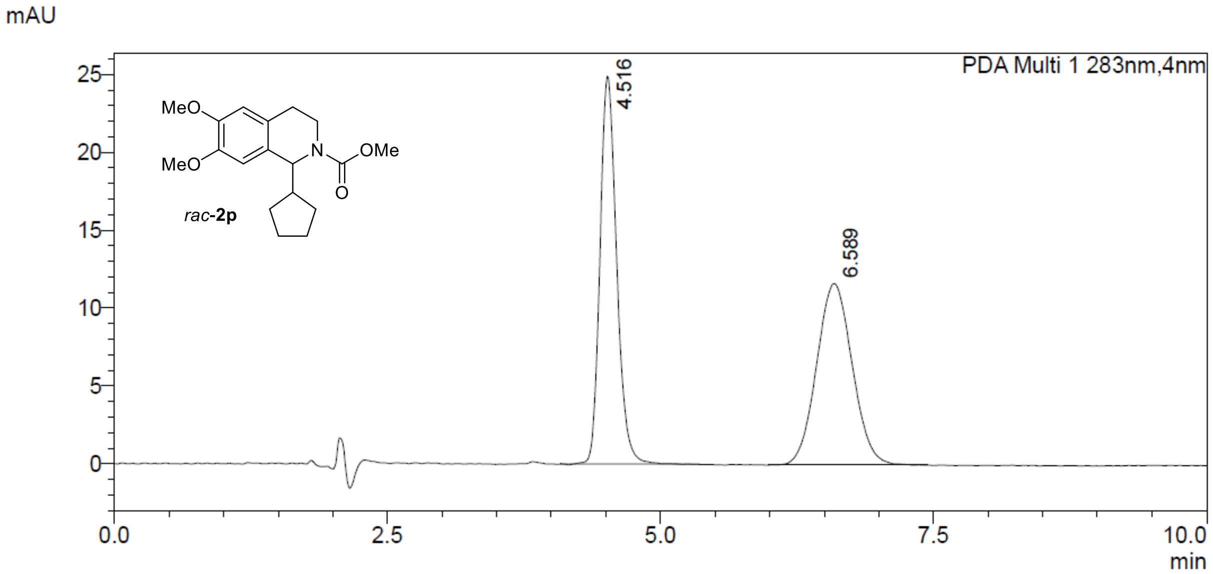

OJ-3R, MeOH/H_2O 90:10, 298 K, 283 nm		
**peak #**	t_R **/ min**	**area / %**
1	4.516	49.954
2	6.589	50.046

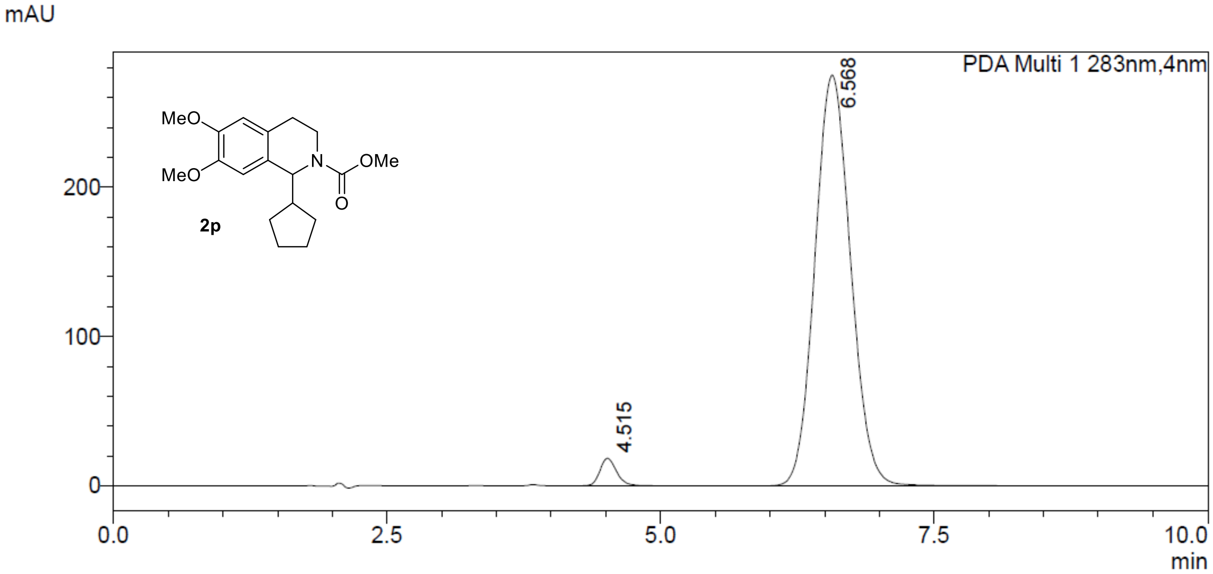

OJ-3R, MeOH/H_2O 90:10, 298 K, 283 nm		
**peak #**	t_R **/ min**	**area / %**
1	4.515	3.020
2	6.568	96.980

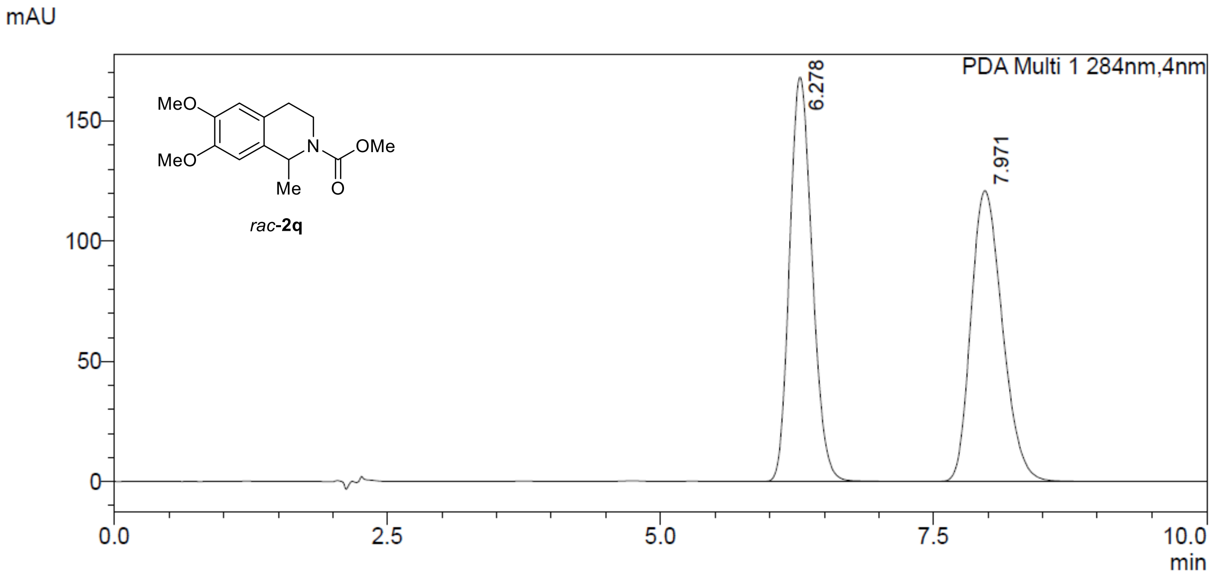

IC-3R, MeOH/H_2O 90:10, 298 K, 284 nm		
**peak #**	t_R **/ min**	**area / %**
1	6.278	50.004
2	7.971	49.996

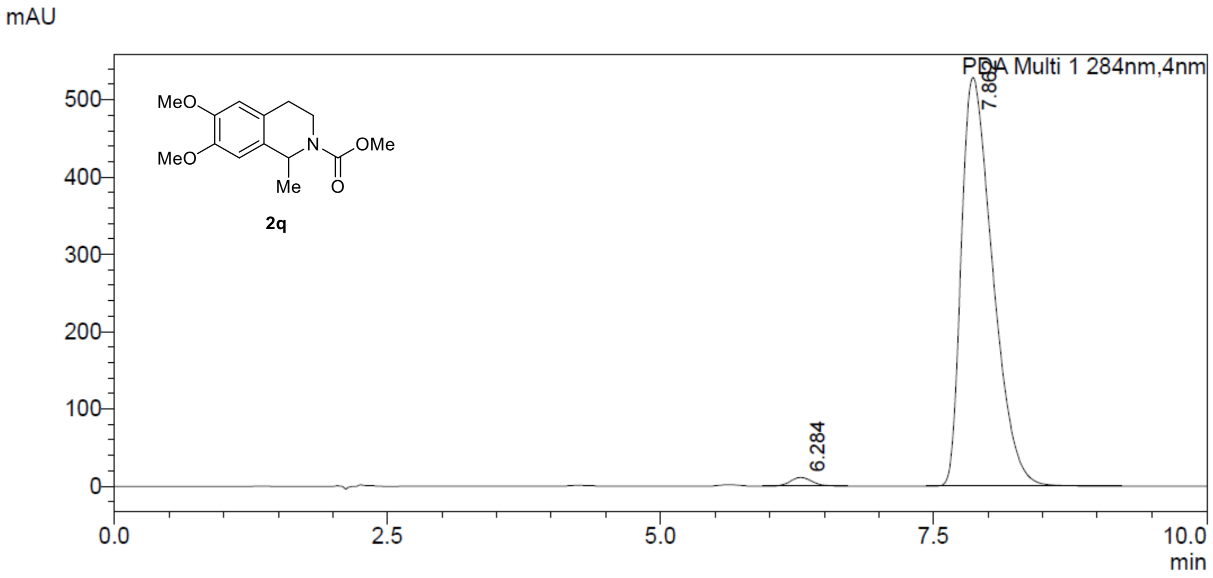

IC-3R, MeOH/H_2O 90:10, 298 K, 284 nm		
**peak #**	t_R **/ min**	**area / %**
1	6.284	1.459
2	7.862	98.541

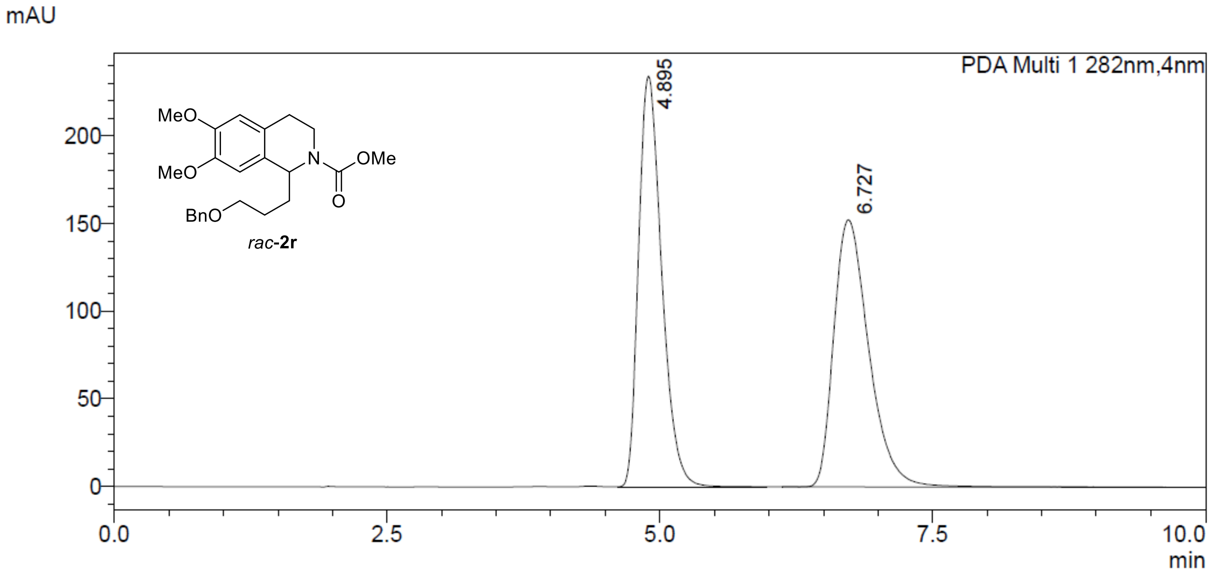

OD-3, *n*-heptane/*i*-PrOH 80:20, 298 K, 282 nm		
**peak #**	t_R **/ min**	**area / %**
1	4.895	50.342
2	6.727	49.658

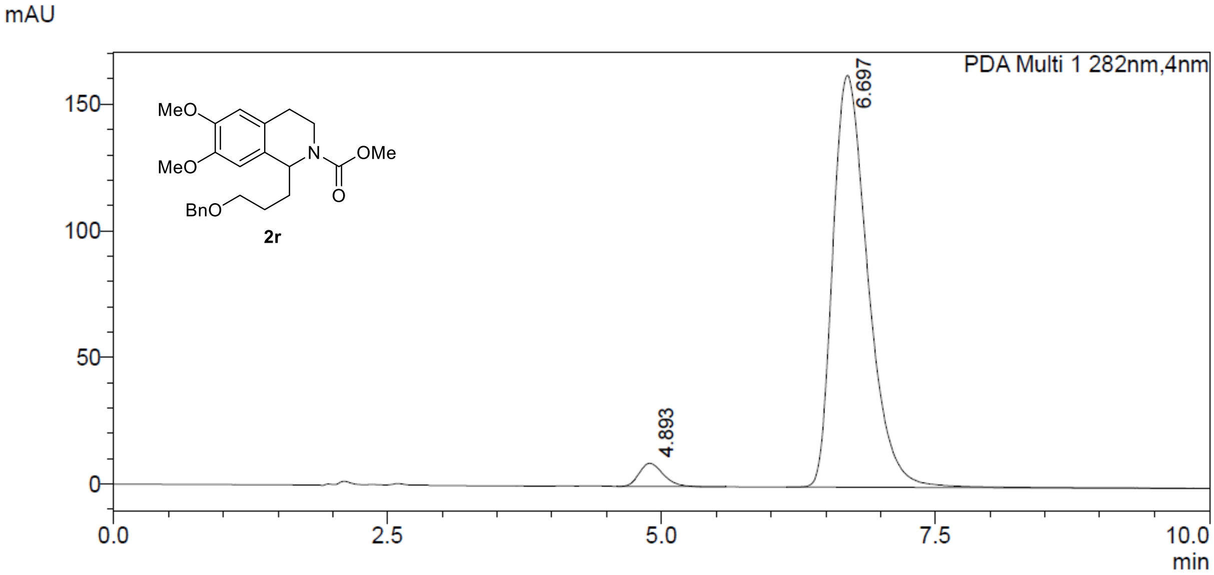

OD-3, *n*-heptane/*i*-PrOH 80:20, 298 K, 282 nm		
**peak #**	t_R **/ min**	**area / %**
1	4.893	3.711
2	6.697	96.289

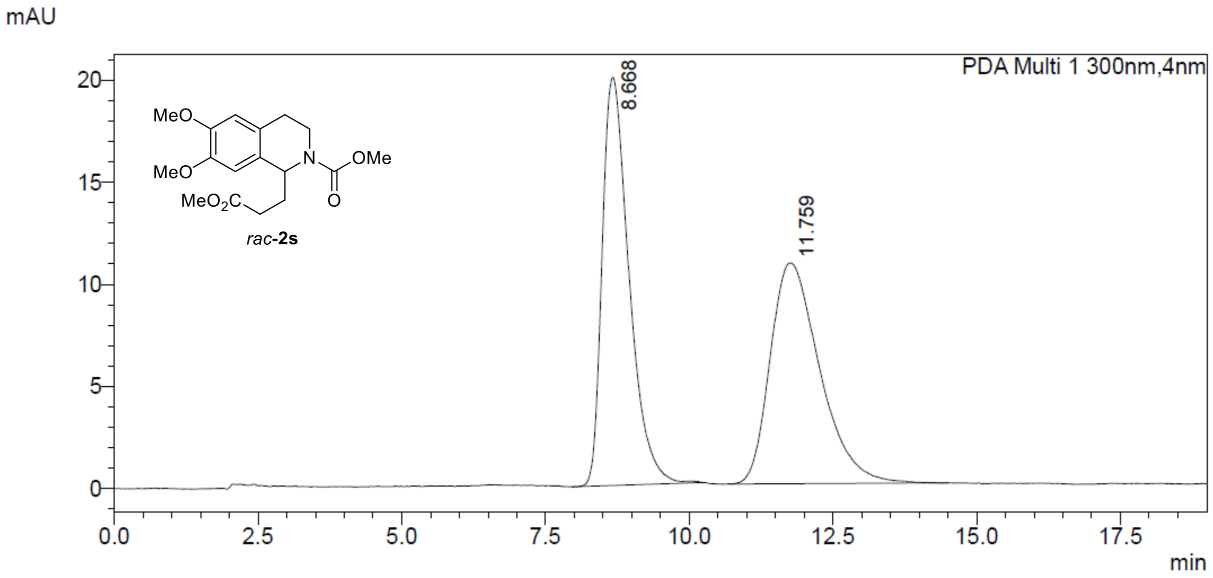

OJ-3, *n*-heptane/*i*-PrOH 80:20, 298 K, 300 nm		
**peak #**	t_R **/ min**	**area / %**
1	8.668	50.097
2	11.759	49.903

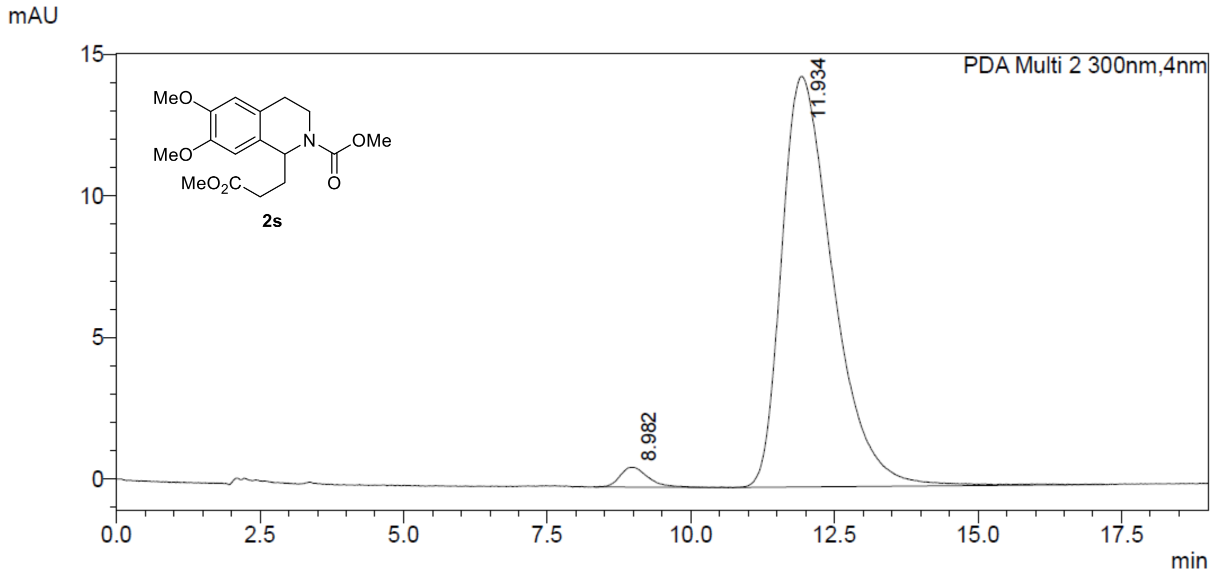

OJ-3, *n*-heptane/*i*-PrOH 80:20, 298 K, 300 nm		
**peak #**	t_R **/ min**	**area / %**
1	8.982	2.600
2	11.934	97.400

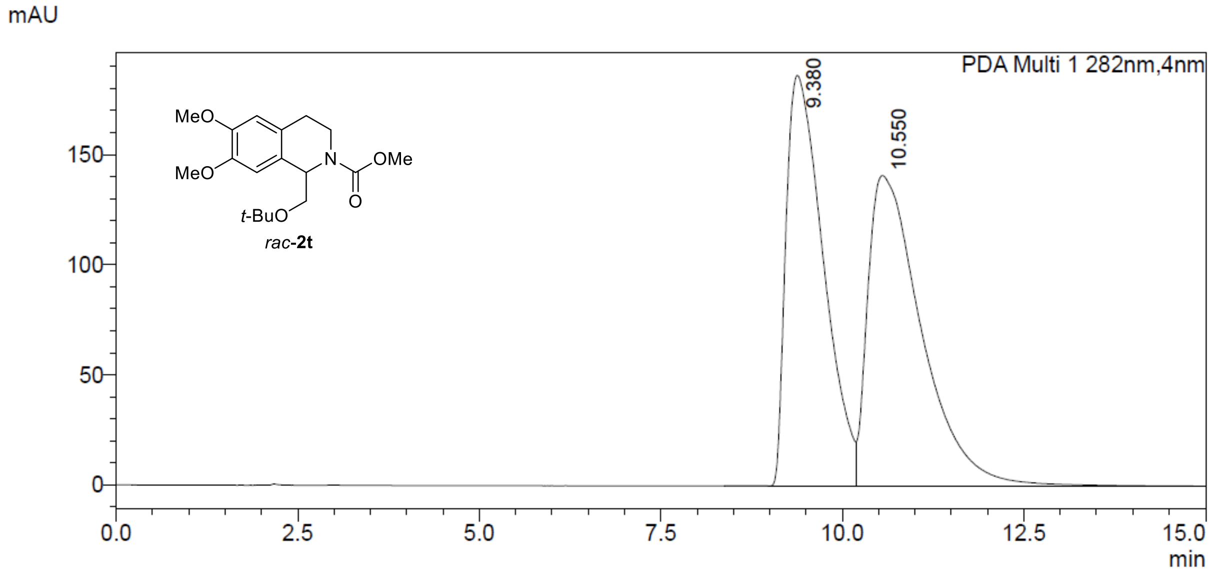

OD-3, *n*-heptane/*i*-PrOH 98:2, 298 K, 282 nm		
**peak #**	t_R **/ min**	**area / %**
1	9.380	47.792
2	10.550	52.208

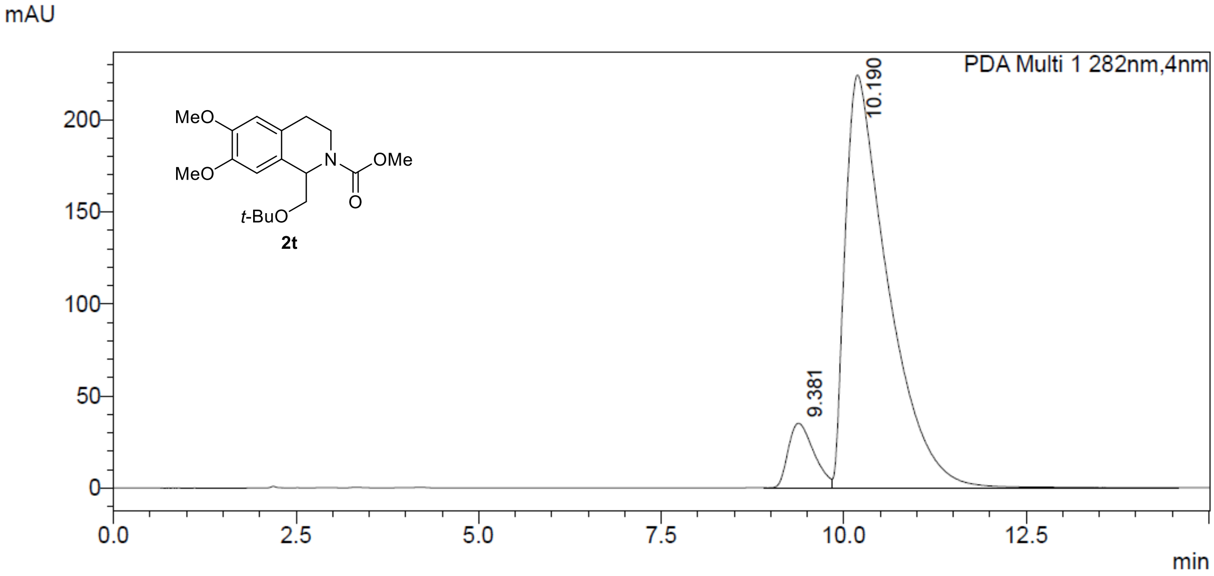

OD-3, *n*-heptane/*i*-PrOH 98:2, 298 K, 282 nm		
**peak #**	t_R **/ min**	**area / %**
1	9.381	8.551
2	10.190	91.449

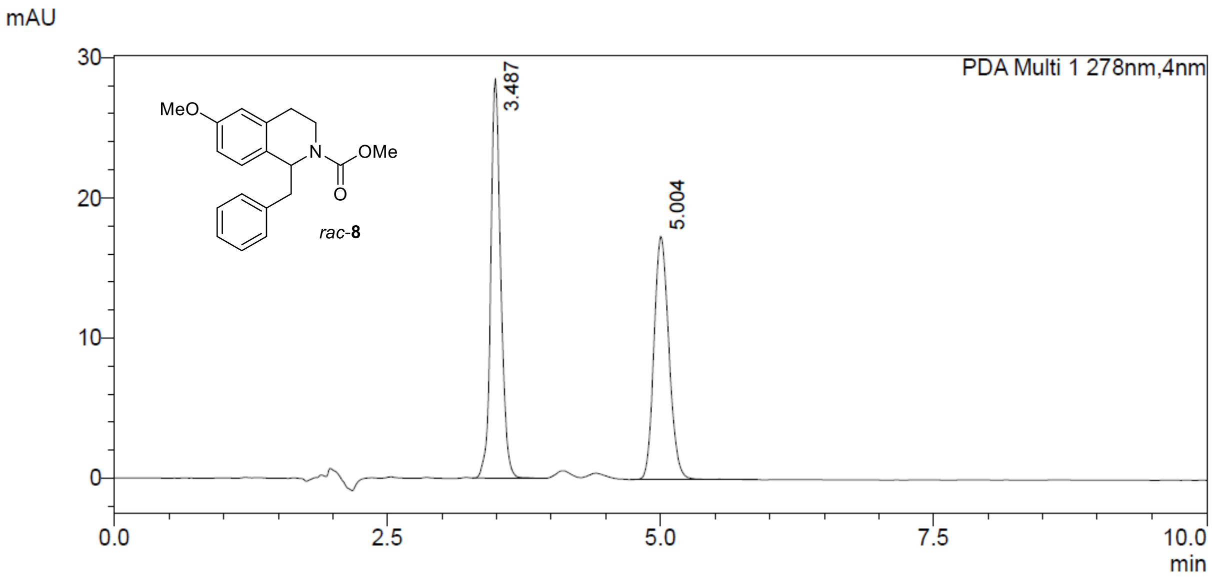

OJ-3R, CH_3CN/H_2O 70:30, 298 K, 278 nm		
**peak #**	***t*$_R$ / min**	**area / %**
1	3.487	52.298
2	5.004	47.702

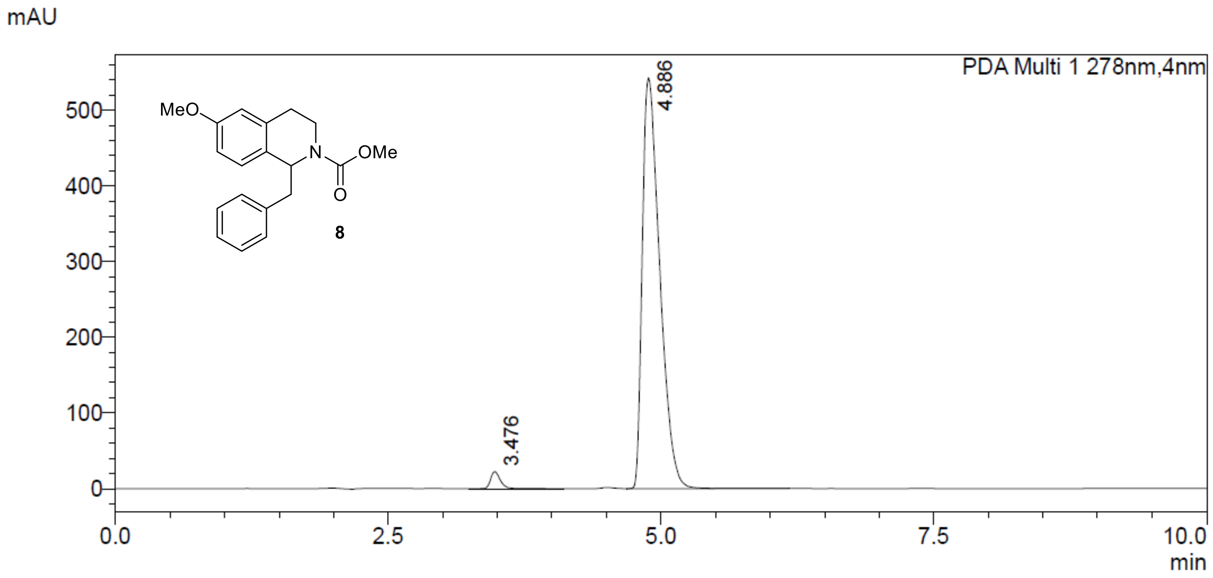

OJ-3R, CH_3CN/H_2O 70:30, 298 K, 278 nm		
**peak #**	***t*$_R$ / min**	**area / %**
1	3.476	2.257
2	4.886	97.743

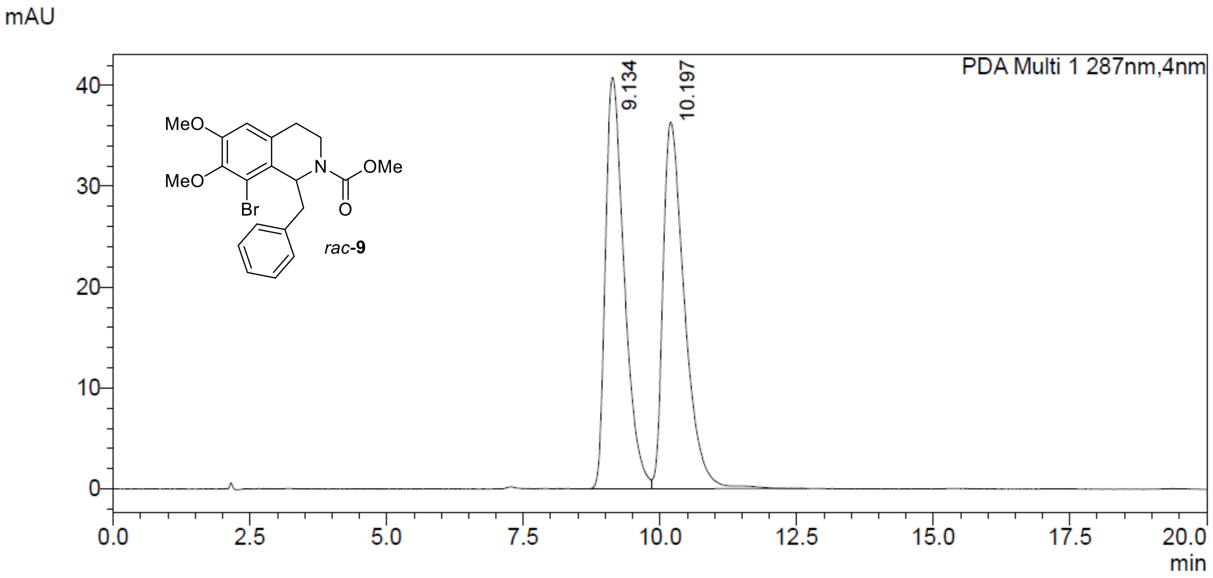

OD-3, *n*-heptane/*i*-PrOH 97:3, 298 K, 287 nm		
**peak #**	t_R **/ min**	**area / %**
1	9.134	49.523
2	10.197	50.477

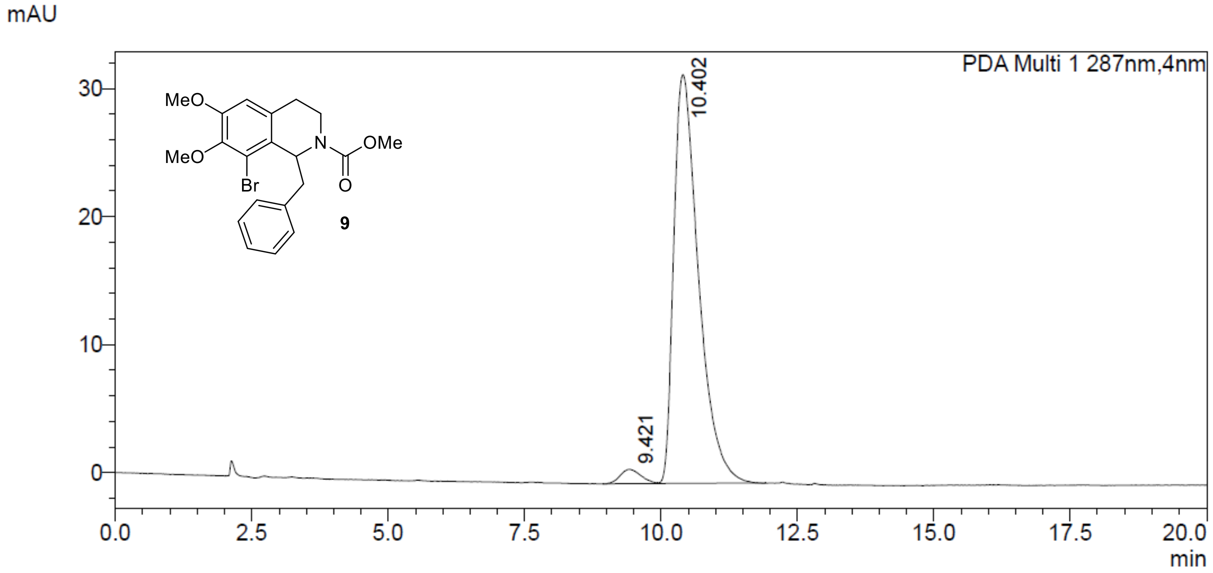

OD-3, *n*-heptane/*i*-PrOH 97:3, 298 K, 287 nm		
**peak #**	t_R **/ min**	**area / %**
1	9.421	2.857
2	10.402	97.143

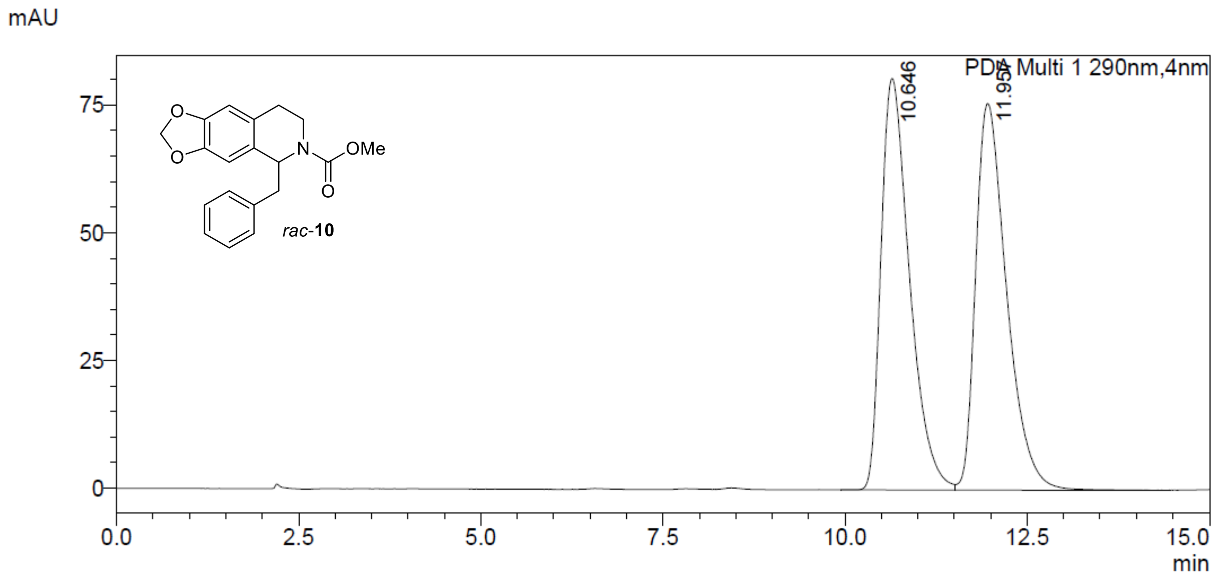

OD-3, *n*-heptane/*i*-PrOH 98:2, 298 K, 290 nm		
**peak #**	**t_R / min**	**area / %**
1	10.646	49.525
2	11.957	50.475

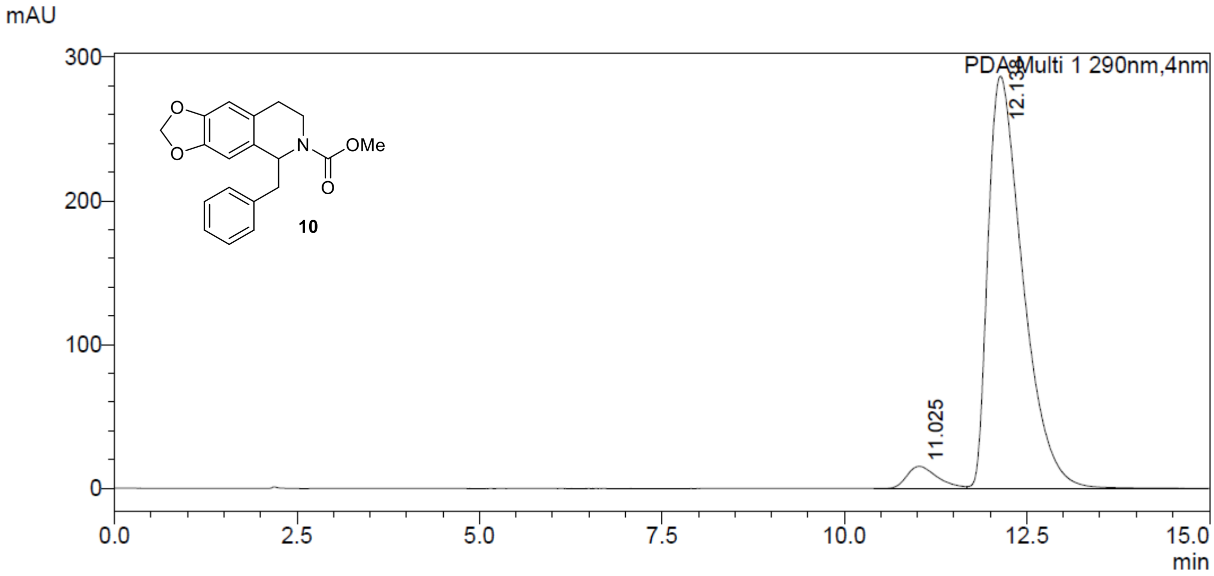

OD-3, *n*-heptane/*i*-PrOH 98:2, 298 K, 290 nm		
**peak #**	**t_R / min**	**area / %**
1	11.025	4.514
2	12.138	95.486

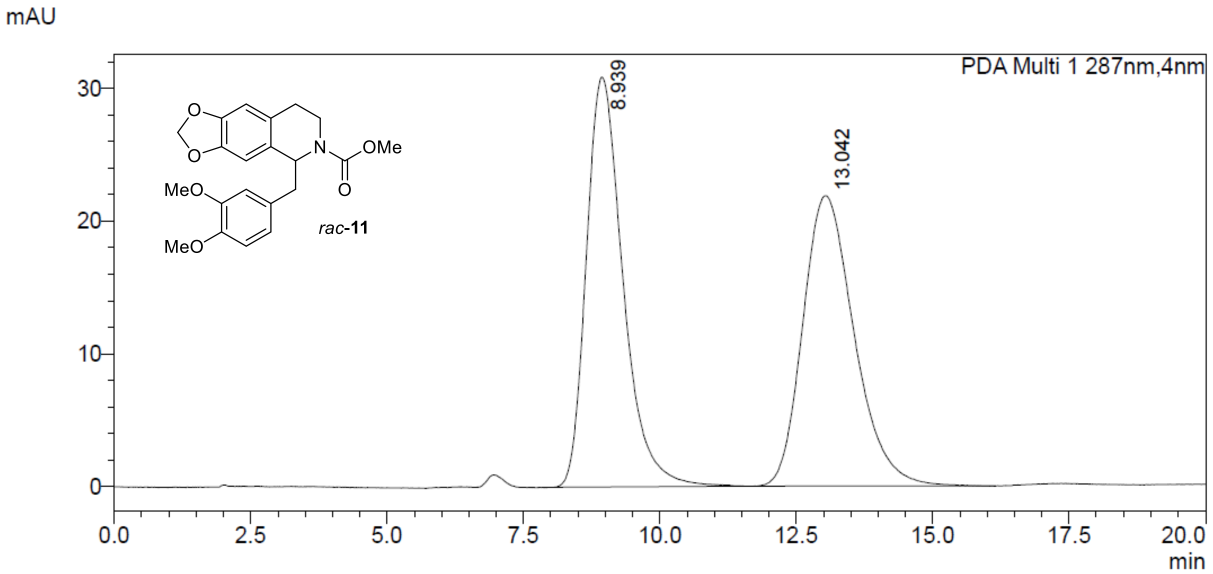

OJ-3, *n*-heptane/*i*-PrOH 70:30, 298 K, 287 nm		
**peak #**	t_R **/ min**	**area / %**
1	8.939	50.205
2	13.042	49.795

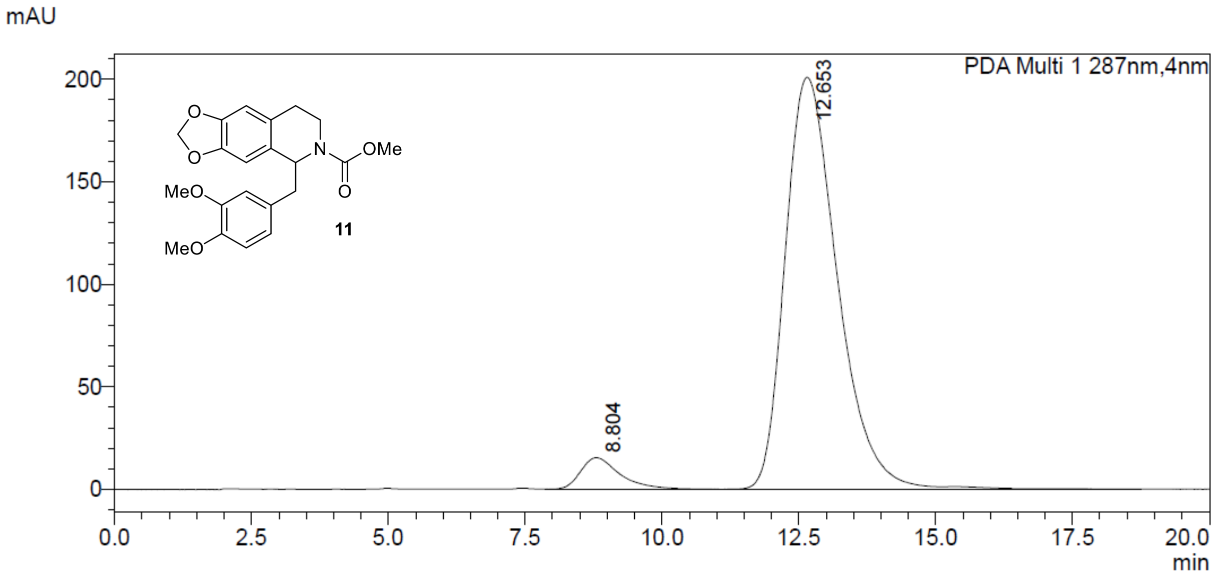

OJ-3, *n*-heptane/*i*-PrOH 70:30, 298 K, 287 nm		
**peak #**	t_R **/ min**	**area / %**
1	8.804	5.428
2	12.653	94.572

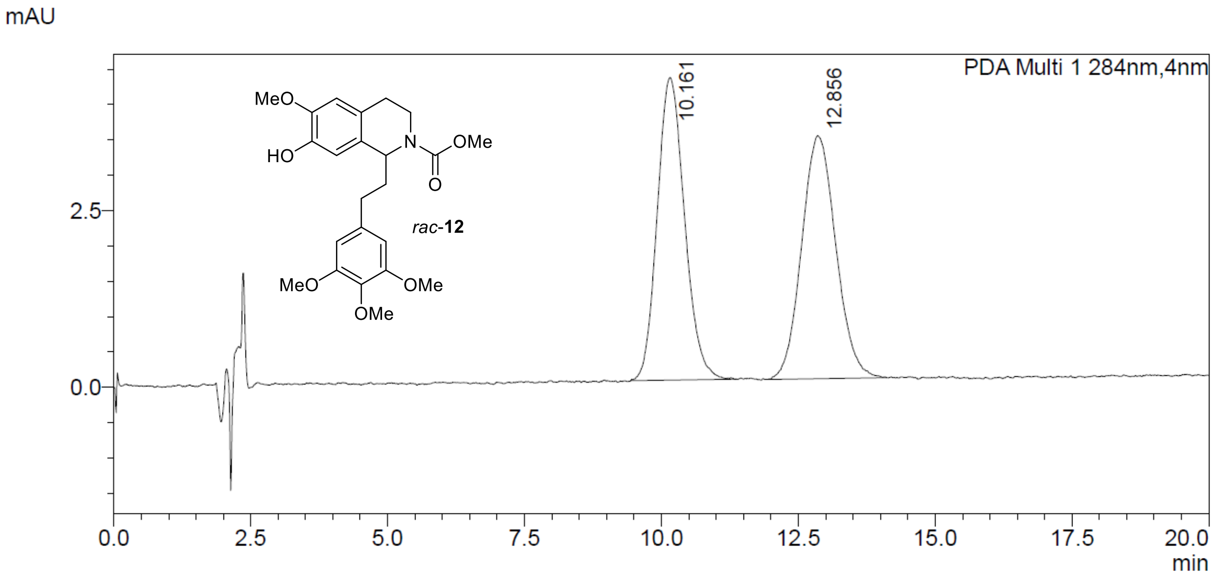

IC-3R, MeOH/H_2O 90:10, 298 K, 284 nm		
**peak #**	t_R **/ min**	**area / %**
1	10.161	49.863
2	12.856	50.137

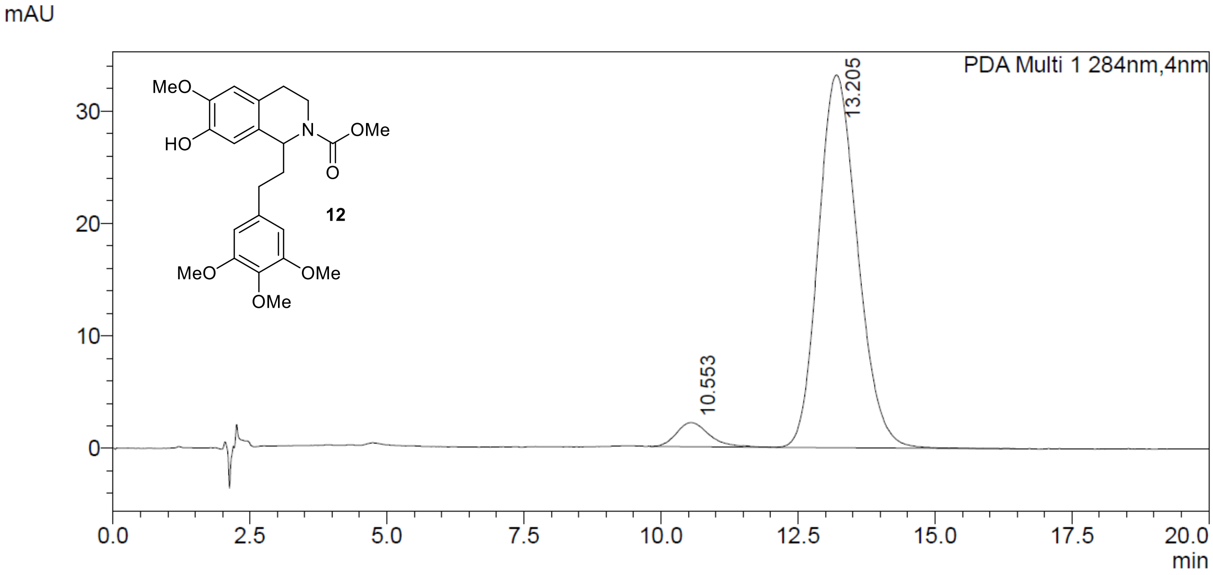

IC-3R, MeOH/H_2O 90:10, 298 K, 284 nm		
**peak #**	t_R **/ min**	**area / %**
1	10.553	5.105
2	13.205	94.895

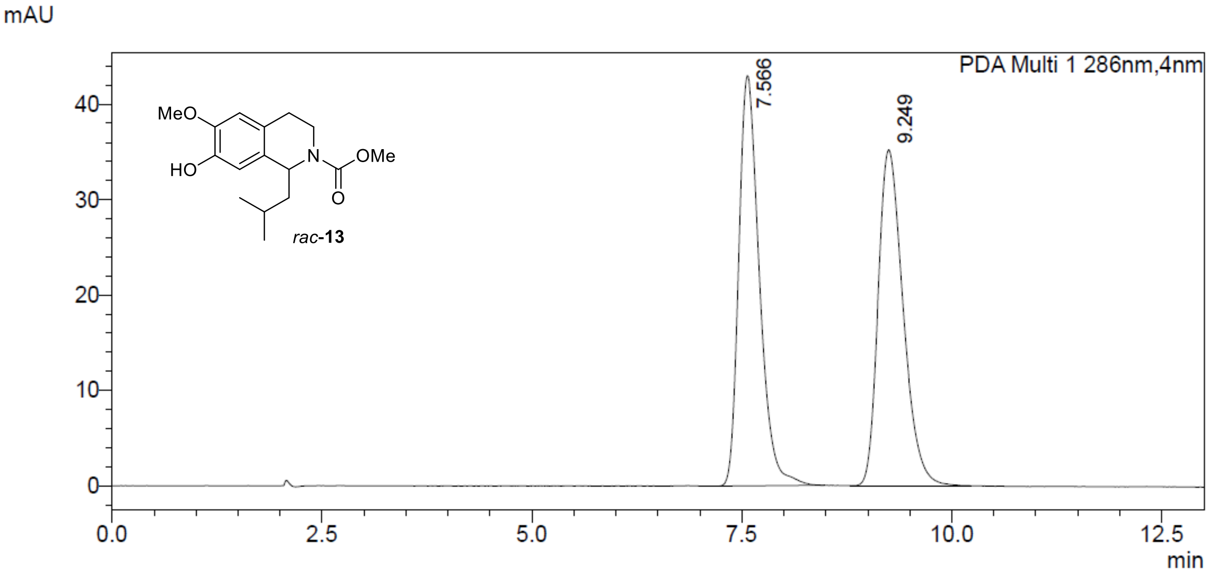

OD-3, *n*-heptane/*i*-PrOH 95:5, 298 K, 286 nm		
**peak #**	***t*$_R$ / min**	**area / %**
1	7.566	50.278
2	9.249	49.722

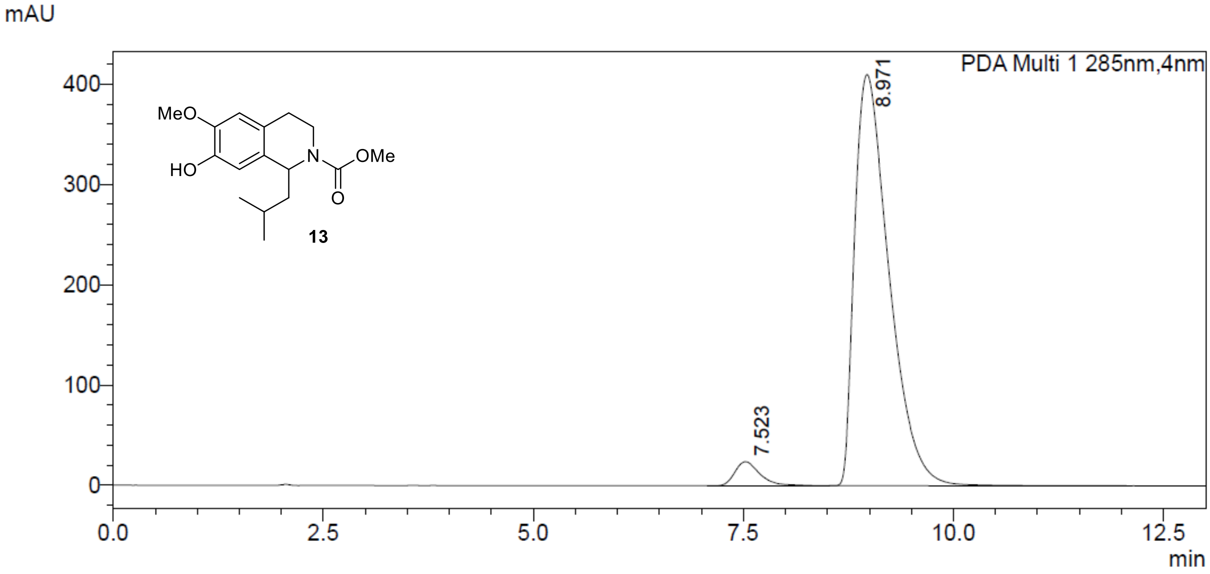

OD-3, *n*-heptane/*i*-PrOH 95:5, 298 K, 285 nm		
**peak #**	***t*$_R$ / min**	**area / %**
1	7.523	4.116
2	8.971	95.884

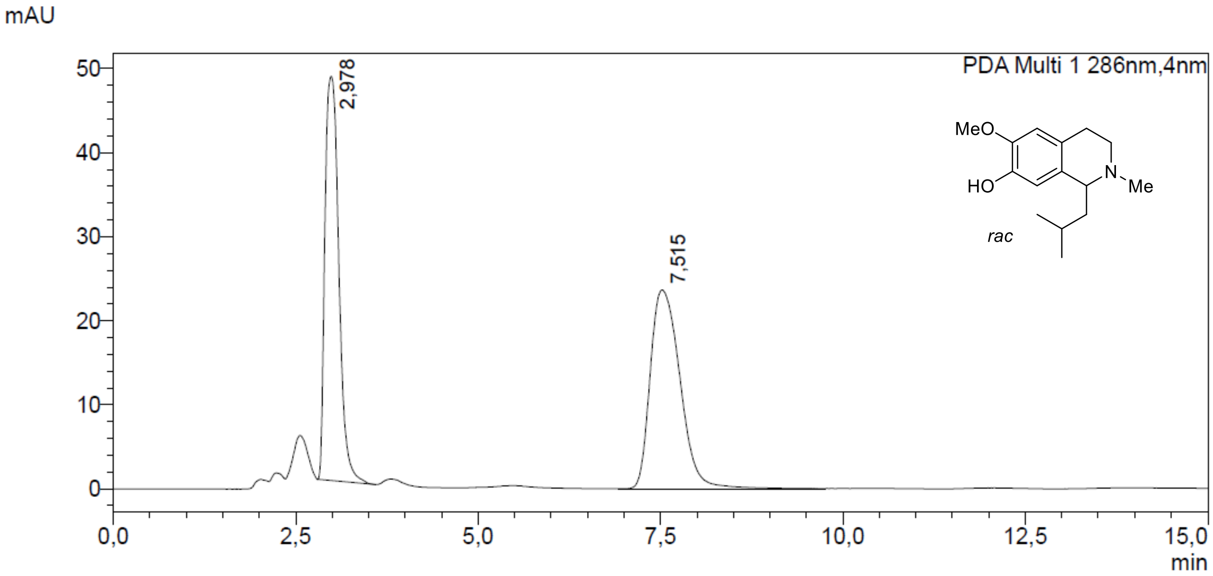

IC-3, (n-heptane + 0.1% Et_3N)/i-PrOH 70:30, 298 K, 286 nm		
**peak #**	$\mathbf{t_R}$ **/ min**	**area / %**
1	2.978	48.573
2	7.515	51.427

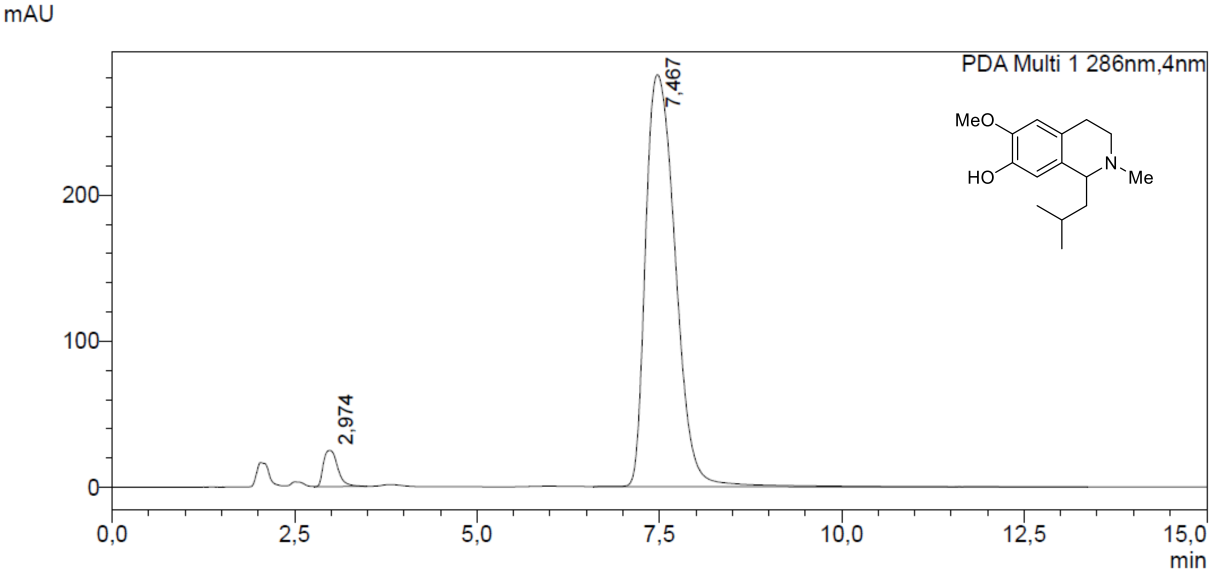

IC-3, (n-heptane + 0.1% Et_3N)/i-PrOH 70:30, 298 K, 286 nm		
**peak #**	$\mathbf{t_R}$ **/ min**	**area / %**
1	2.974	3.997
2	7.467	96.003

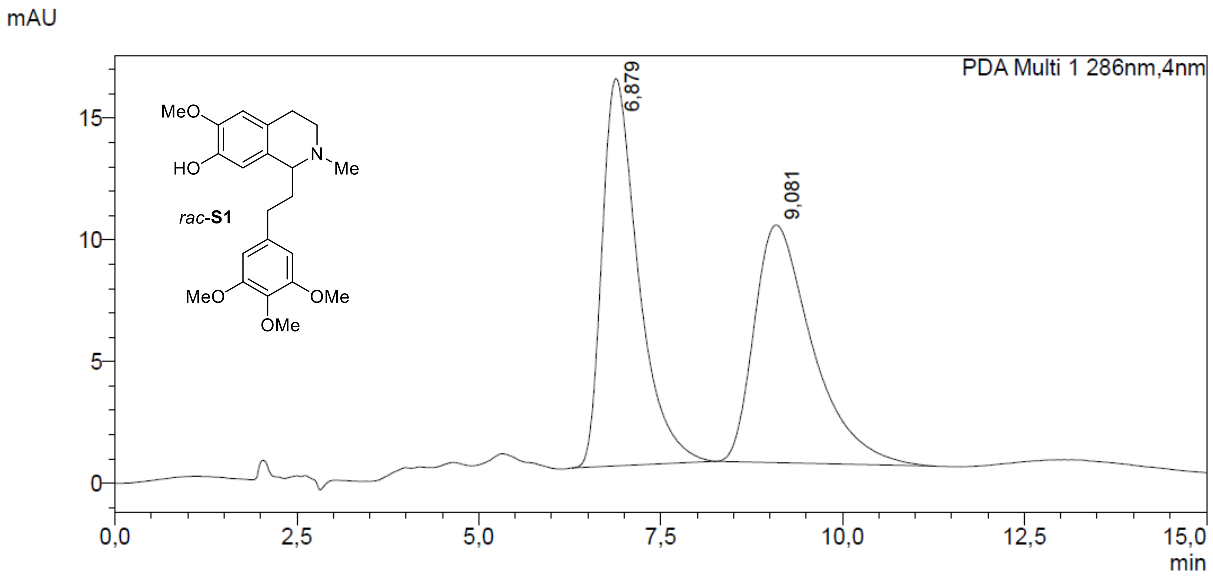

OJ-3, (*n*-heptane + 0.1% Et_3N)/*i*-PrOH 70:30, 298 K, 286 nm		
**peak #**	***t*$_R$ / min**	**area / %**
1	6.879	50.325
2	9.081	49.675

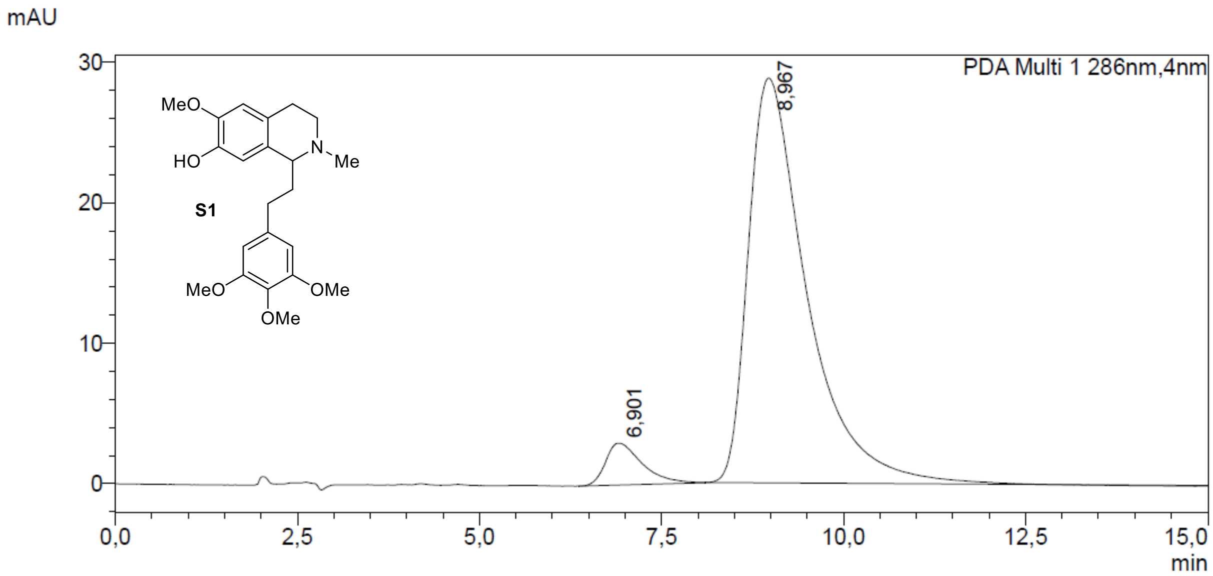

OJ-3, (*n*-heptane + 0.1% Et_3N)/*i*-PrOH 70:30, 298 K, 286 nm		
**peak #**	***t*$_R$ / min**	**area / %**
1	6.901	6.104
2	8.967	93.896

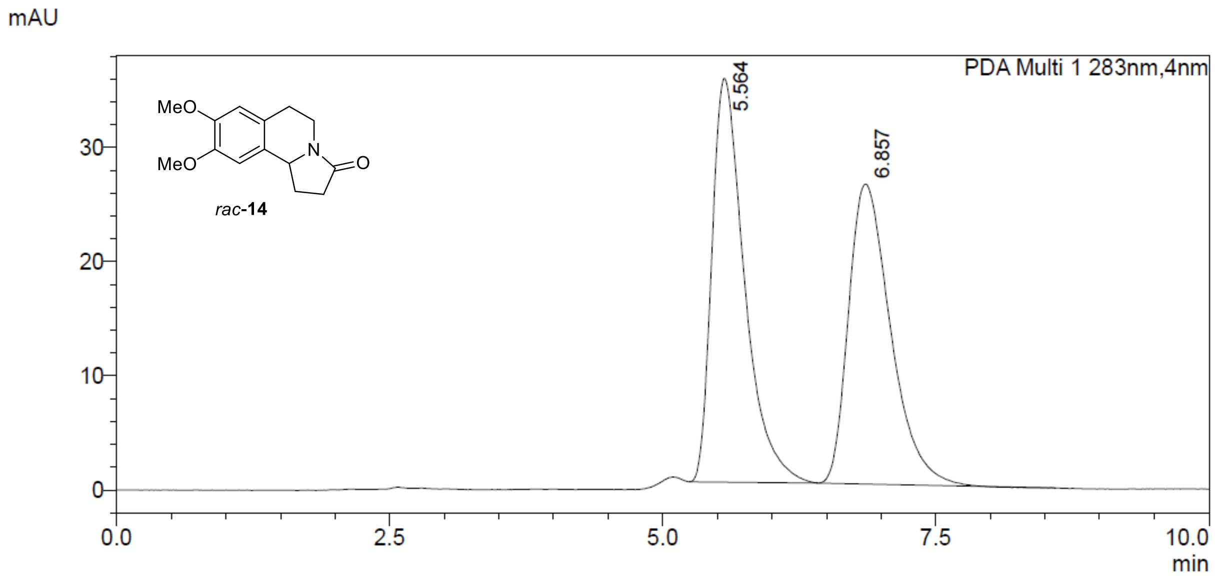

OJ-3, *n*-heptane/*i*-PrOH 60:40, 298 K, 283 nm		
**peak #**	t_R **/ min**	**area / %**
1	5.564	51.120
2	6.857	48.880

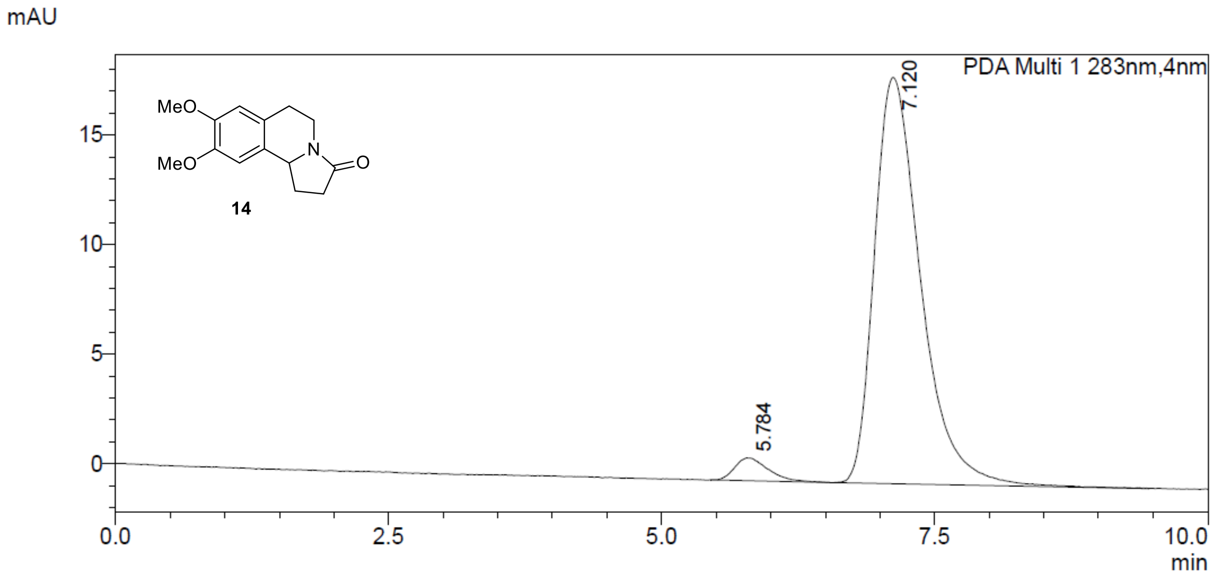

OJ-3, *n*-heptane/*i*-PrOH 60:40, 298 K, 283 nm		
**peak #**	t_R **/ min**	**area / %**
1	5.784	3.922
2	7.120	96.078

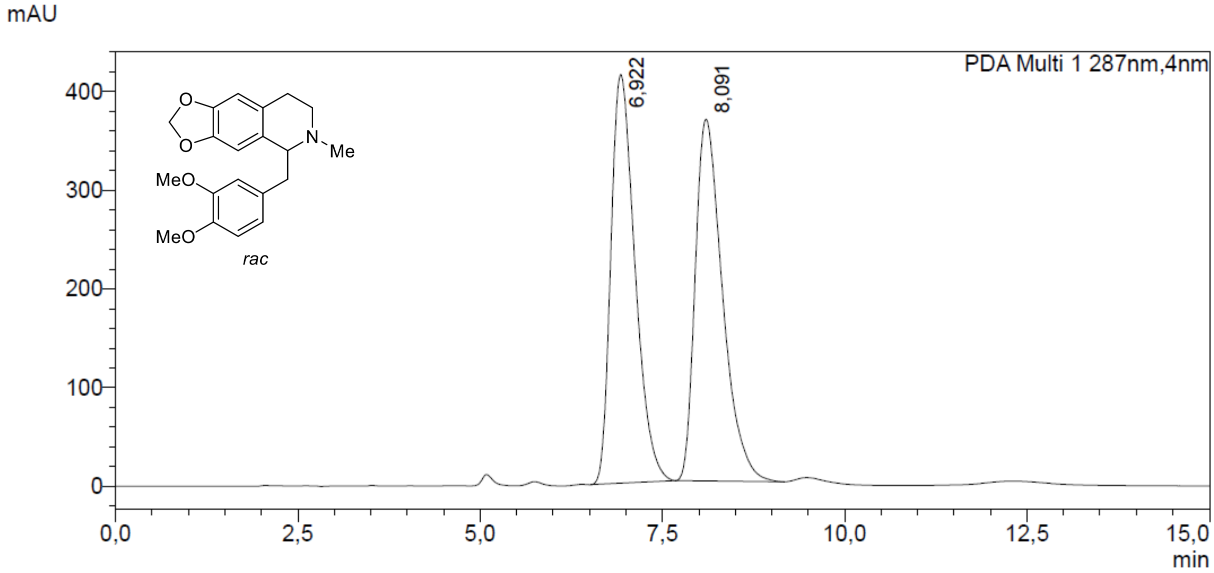

OJ-3, (n-heptane + 0.1% Et_3N)/i-PrOH 70:30, 298 K, 287 nm		
**peak #**	t_R **/ min**	**area / %**
1	6.922	49.881
2	8.091	50.119

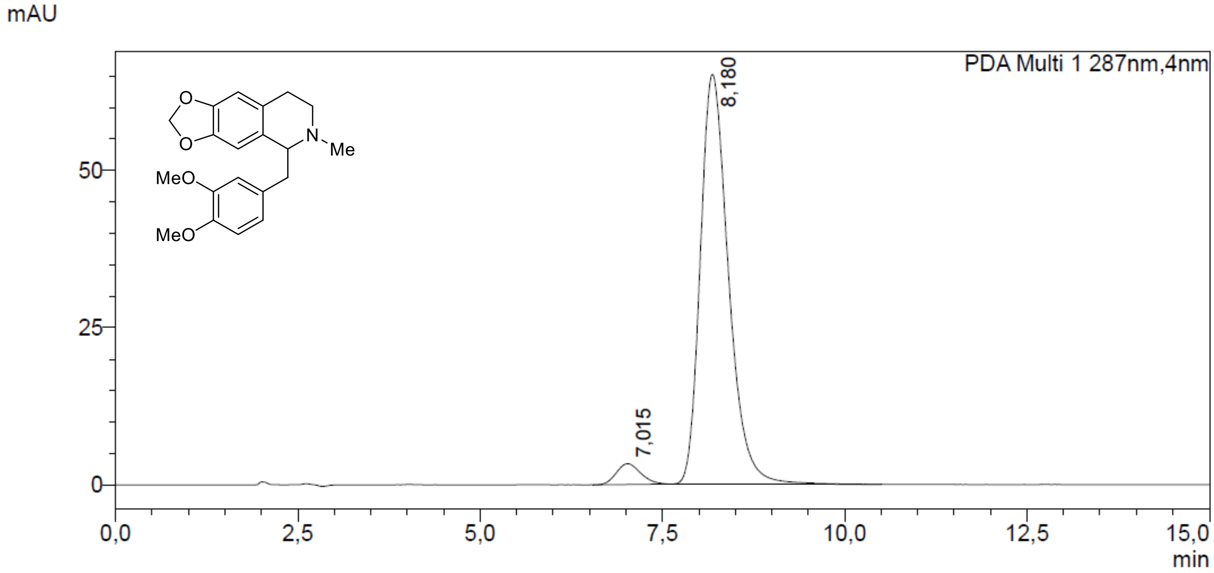

OJ-3, (n-heptane + 0.1% Et_3N)/i-PrOH 70:30, 298 K, 287 nm		
**peak #**	t_R **/ min**	**area / %**
1	7.015	4.220
2	8.180	95.780

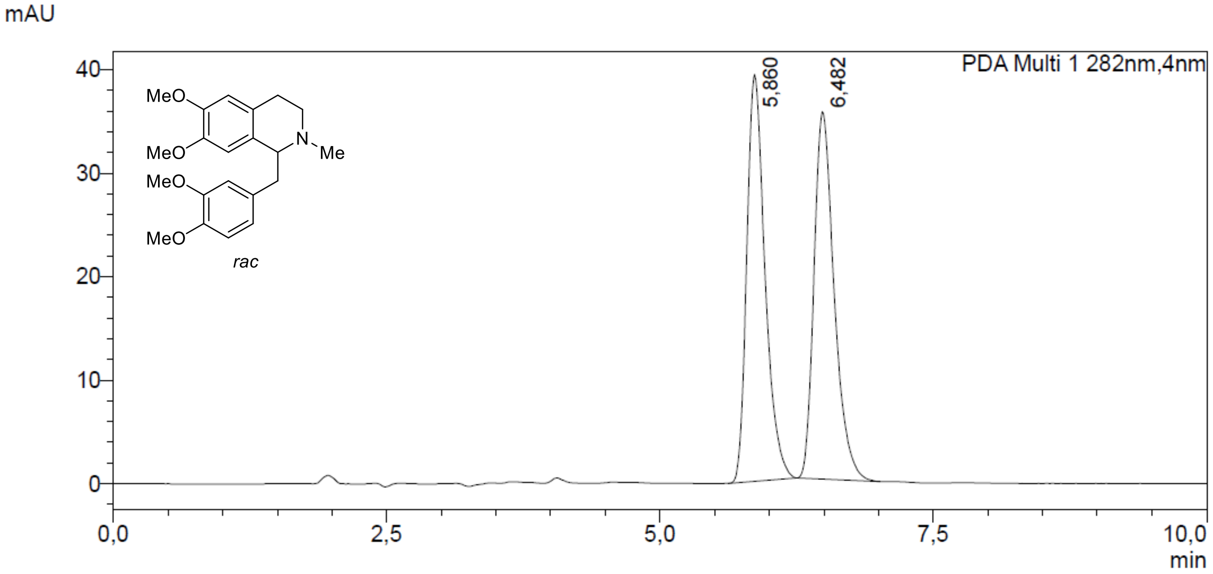

AD-3, (n-heptane + 0.1% Et_3N)/i-PrOH 80:20, 298 K, 282 nm		
**peak #**	$\boldsymbol{t_R}$ **/ min**	**area / %**
1	5.860	50.017
2	6.482	49.983

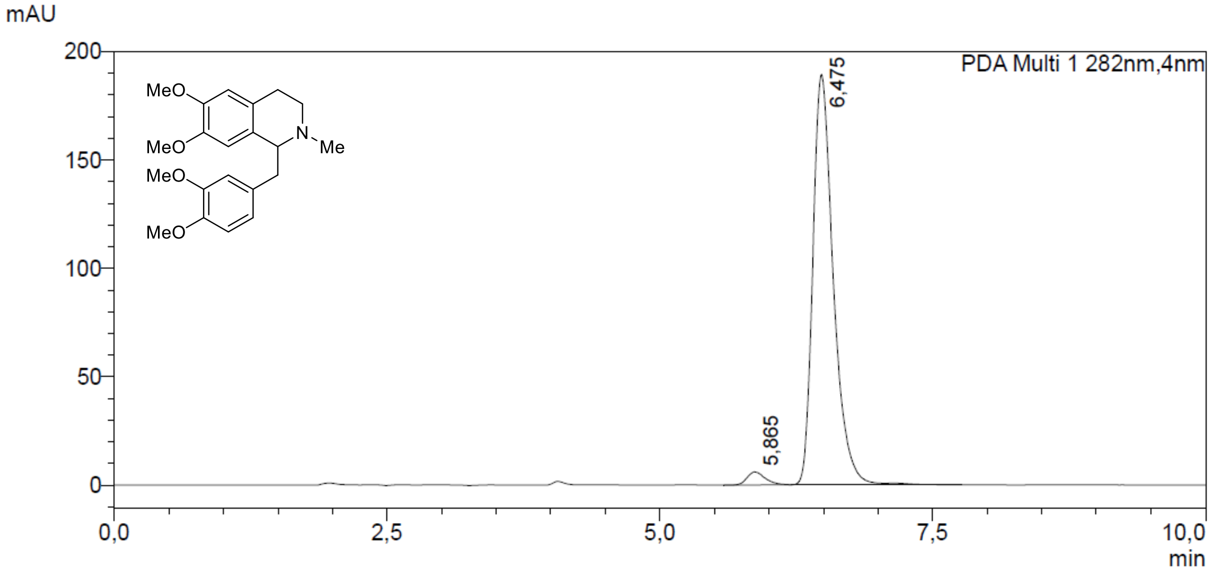

AD-3, (n-heptane + 0.1% Et_3N)/i-PrOH 80:20, 298 K, 282 nm		
**peak #**	$\boldsymbol{t_R}$ **/ min**	**area / %**
1	5.865	2.716
2	6.475	97.284

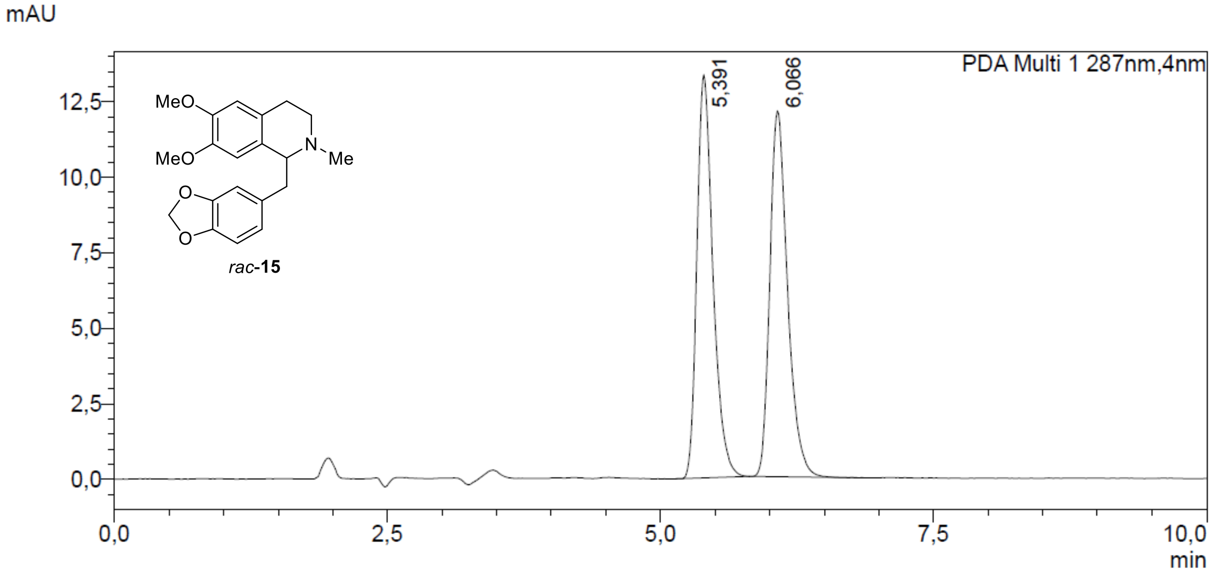

AD-3, (n-heptane + 0.1% Et_3N)/i-PrOH 80:20, 298 K, 282 nm		
**peak #**	$\boldsymbol{t_R}$ **/ min**	**area / %**
1	5.391	49.978
2	6.066	50.022

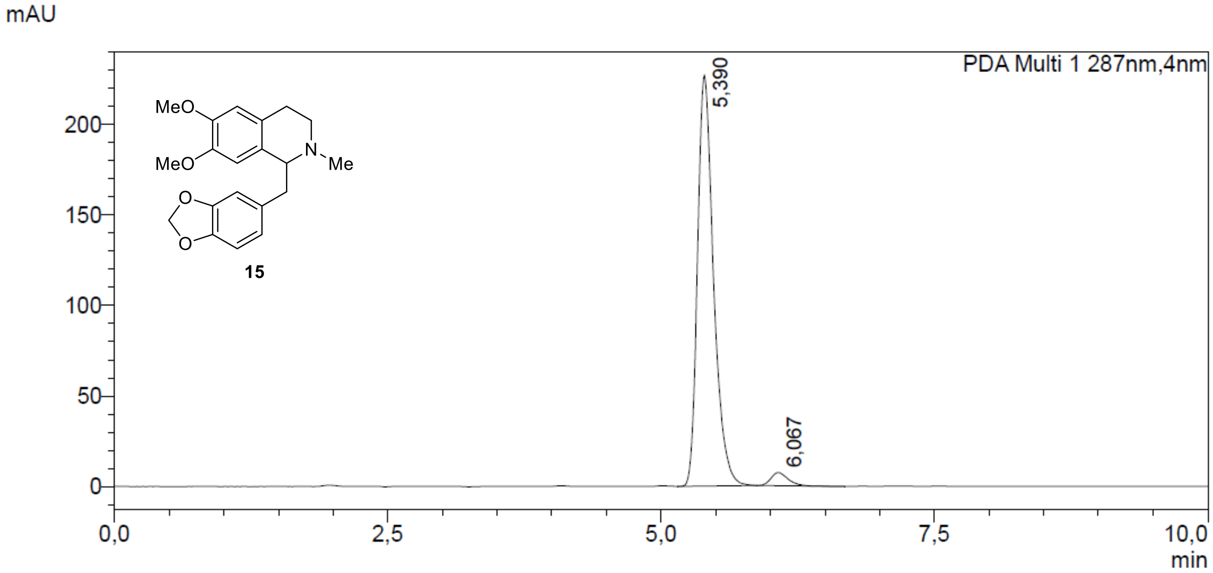

AD-3, (n-heptane + 0.1% Et_3N)/i-PrOH 80:20, 298 K, 282 nm		
**peak #**	$\boldsymbol{t_R}$ **/ min**	**area / %**
1	5.390	96.802
2	6.067	3.198

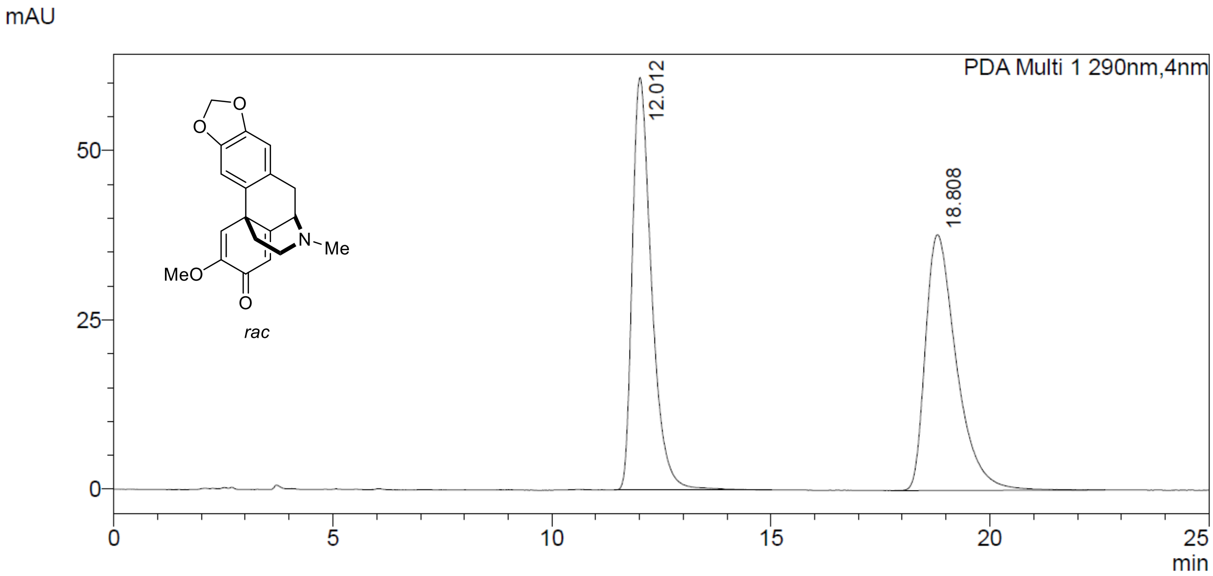

IE-3, (n-heptane + 0.1% Et_3N)/i-PrOH 50:50, 298 K, 290 nm		
**peak #**	**t_R / min**	**area / %**
1	12.012	50.019
2	18.808	49.981

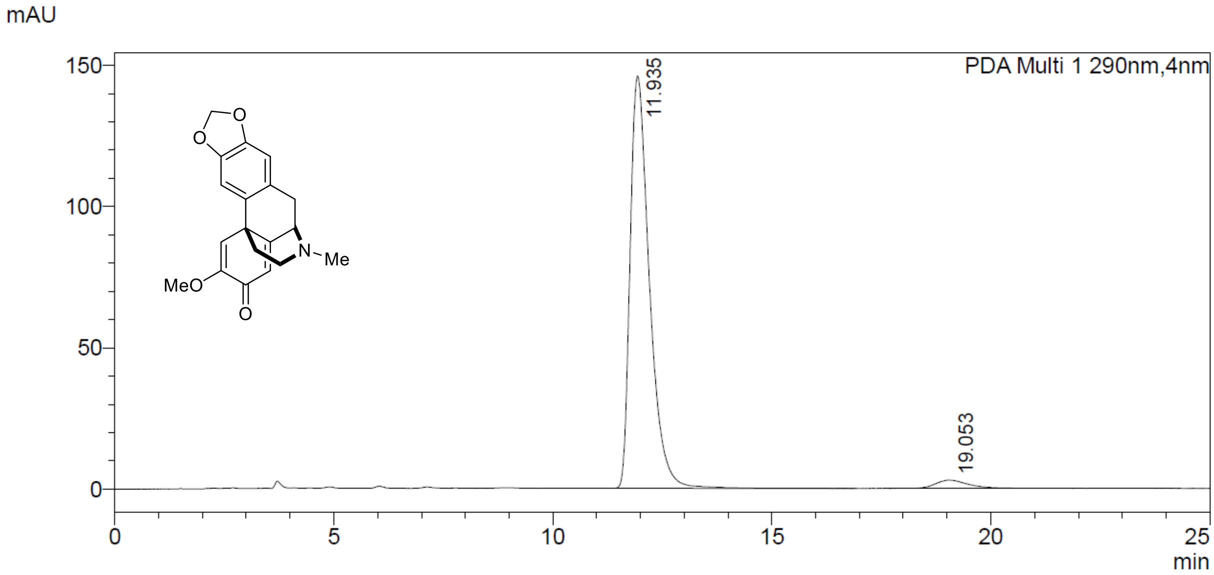

IE-3, (n-heptane + 0.1% Et_3N)/i-PrOH 50:50, 298 K, 290 nm		
**peak #**	**t_R / min**	**area / %**
1	11.935	96.641
2	19.053	3.359

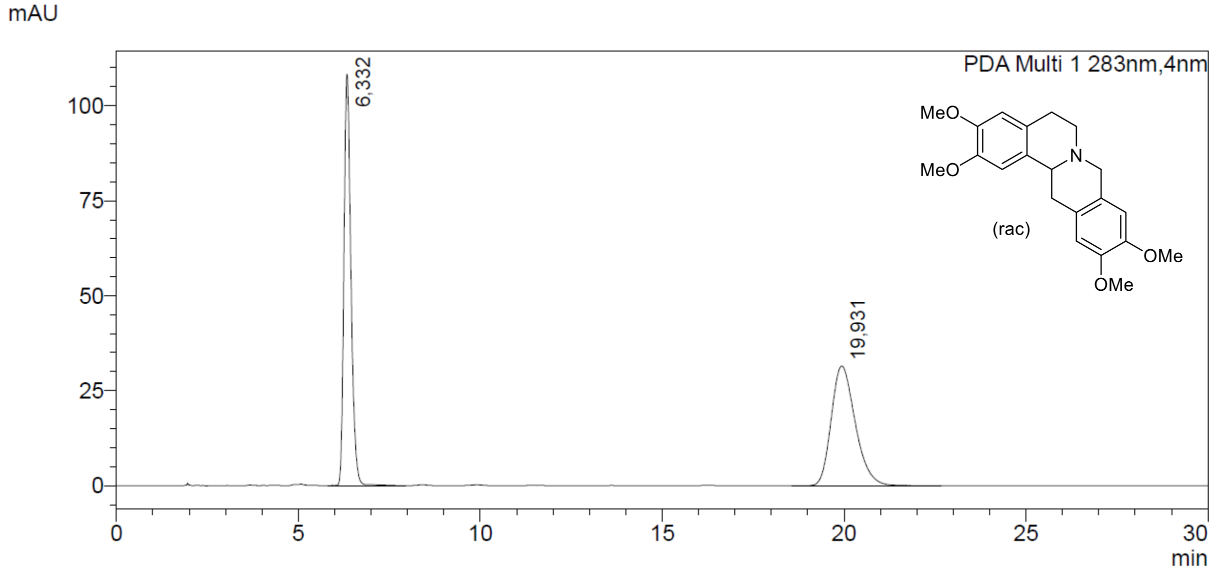

AD-3, (n-heptane + 0.1% Et_3N)/i-PrOH 60:40, 298 K, 283 nm		
**peak #**	t_R **/ min**	**area / %**
1	6.332	50.087
2	19.931	49.913

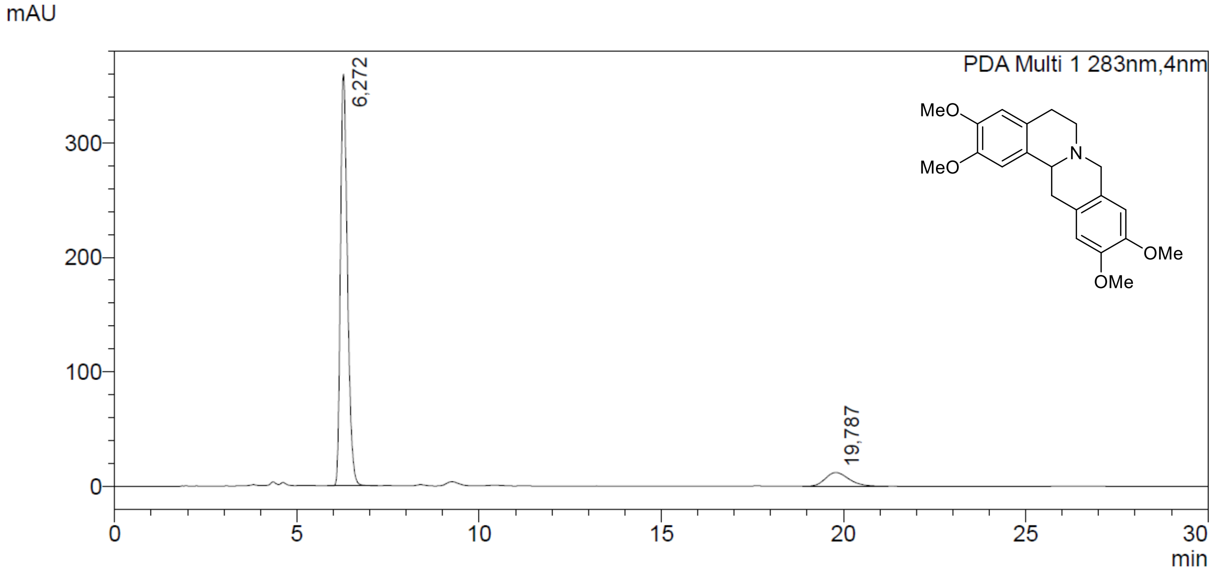

AD-3, (n-heptane + 0.1% Et_3N)/i-PrOH 60:40, 298 K, 283 nm		
**peak #**	t_R **/ min**	**area / %**
1	6.272	89.965
2	19.787	10.035

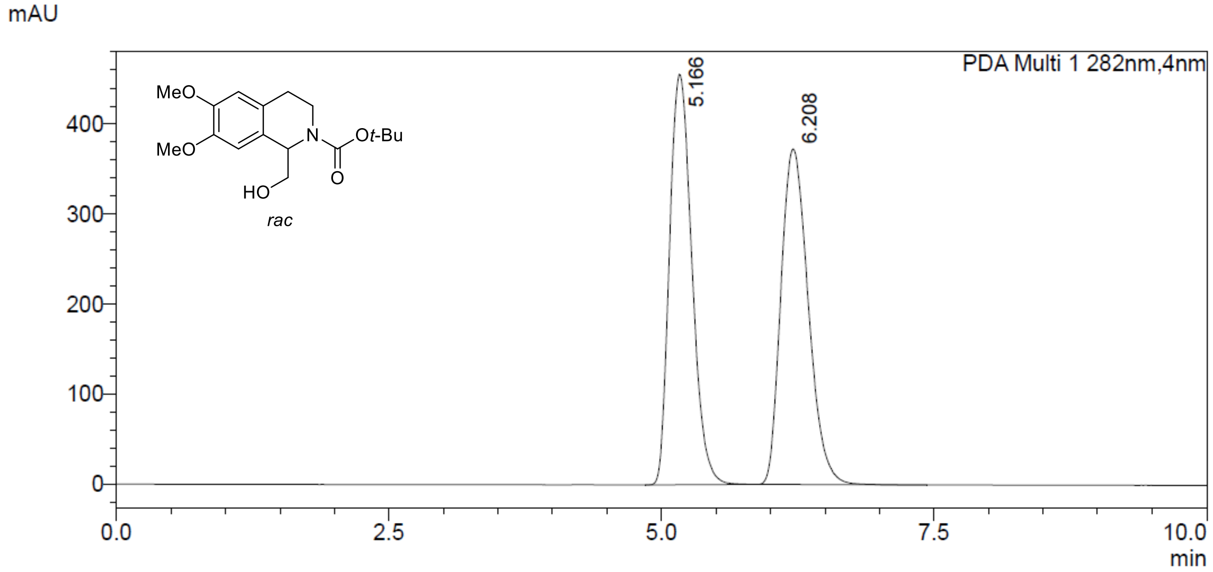

IA-3, *n*-heptane/*i*-PrOH 70:30, 298 K, 282 nm		
**peak #**	t_R **/ min**	**area / %**
1	5.166	49.991
2	6.208	50.009

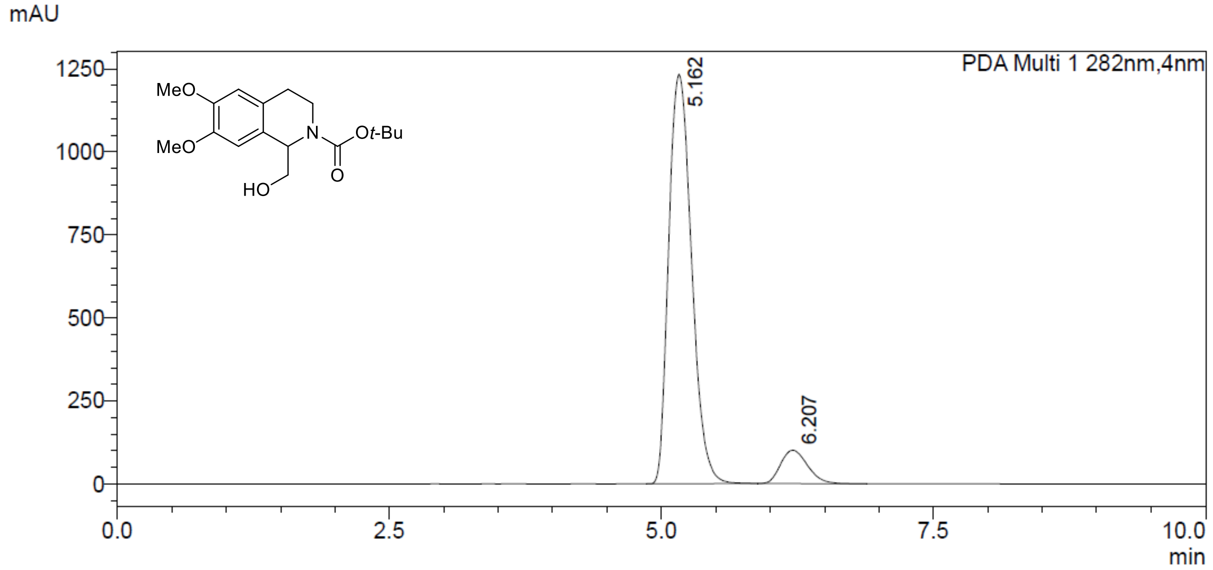

IA-3, *n*-heptane/*i*-PrOH 70:30, 298 K, 282 nm		
**peak #**	t_R **/ min**	**area / %**
1	5.162	91.257
2	6.207	8.743

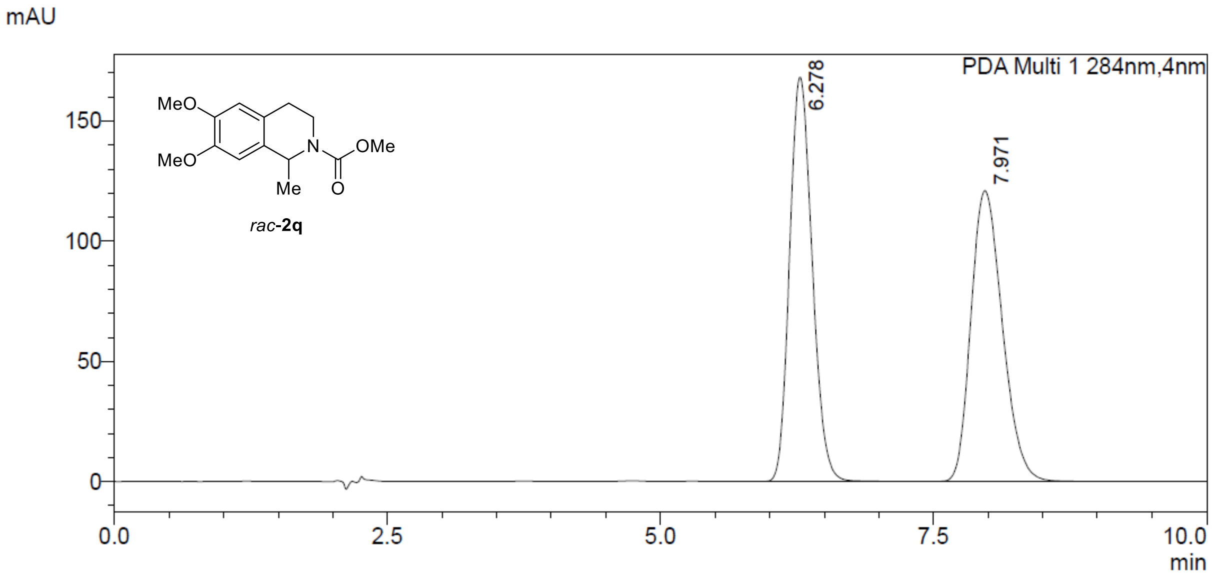

IC-3R, MeOH/H_2O 90:10, 298 K, 284 nm		
**peak #**	***t*R / min**	**area / %**
1	6.278	50.004
2	7.971	49.996

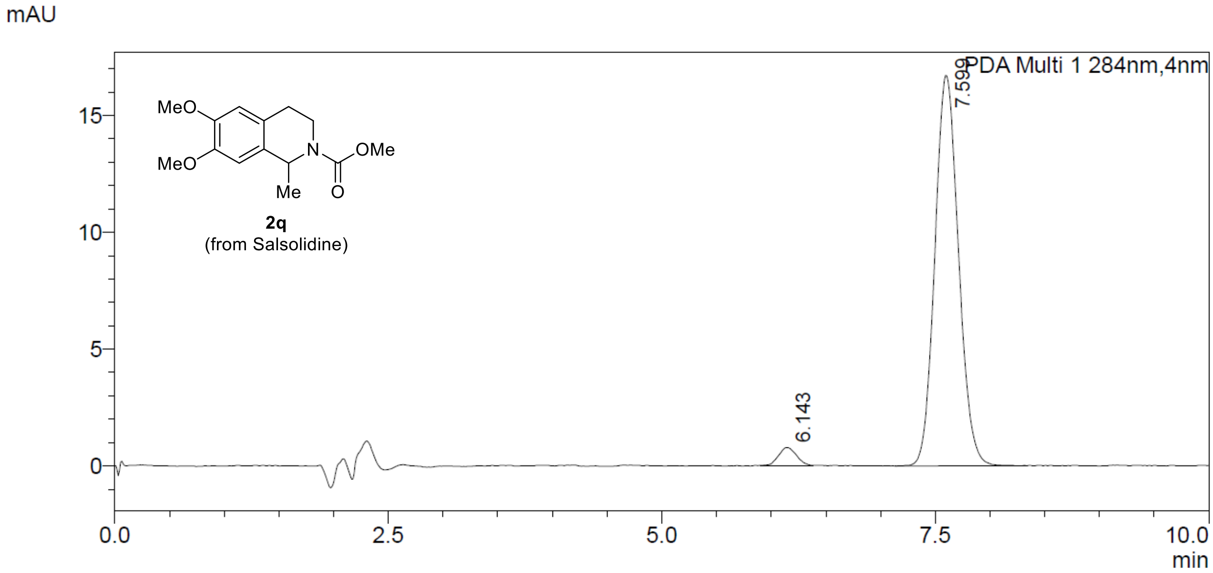

IC-3R, MeOH/H_2O 90:10, 298 K, 284 nm		
**peak #**	***t*R / min**	**area / %**
1	6.143	3.324
2	7.599	96.676

10. Crystallographic Data

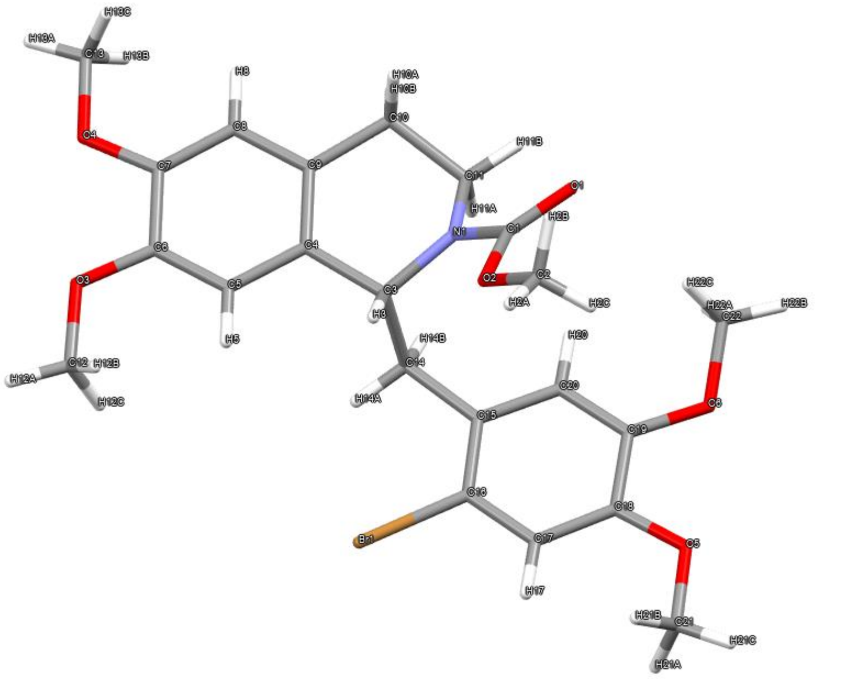

Figure S2. X-Ray structure of **2i**.

Table S4. Crystal data and structure refinement.

Identification code	14467	
Empirical formula	$C_{22}H_{26}BrNO_6$	
Color	colourless	
Formula weight	480.35 g · mol-1	
Temperature	100(2) K	
Wavelength	0.71073 Å	
Crystal system	MONOCLINIC	
Space group	**P2$_1$, (no. 4)**	
Unit cell dimensions	a = 9.7395(4) Å	α = 90°.
	b = 6.1243(2) Å	β = 91.348(2)°.
	c = 17.5601(7) Å	γ = 90°.
Volume	1047.13(7) Å^3	
Z	2	
Density (calculated)	1.523 Mg · m^{-3}	
Absorption coefficient	2.002 mm^{-1}	
F(000)	496 e	

Crystal size	0.121 x 0.051 x 0.032 mm^3
θ range for data collection	1.160 to 34.925°.
Index ranges	-15 ≤ h ≤ 15, -9 ≤ k ≤ 9, -28 ≤ l ≤ 28
Reflections collected	34114
Independent reflections	9085 [R_{int} = 0.0327]
Reflections with I>2σ(I)	7802
Completeness to θ = 25.242°	100.0 %
Absorption correction	Gaussian
Max. and min. transmission	0.95 and 0.85
Refinement method	Full-matrix least-squares on F2
Data/restraints/parameters	9085/1/280
Goodness-of-fit on F^2	1.020
Final R indices [I>2σ(I)]	R_1 = 0.0319 wR^2 = 0.0630
R indices (all data)	R_1 = 0.0439 wR^2 = 0.0667
Absolute structure parameter	-0.011(3)
Largest diff. peak and hole	0.4 and -0.5 e · $Å^{-3}$

Table S5. Bond lengths [Å] and angles [°].

Br1-C16	1.906(2)	C9-C10	1.507(3)
O1-C1	1.216(2)	C10-H10A	0.990
O2-C1	1.346(2)	C10-H10B	0.990
O2-C2	1.448(2)	C10-C11	1.529(3)
O3-C6	1.365(2)	C11-H11A	0.990
O3-C12	1.417(3)	C11-H11B	0.990
O4-C7	1.360(2)	C12-H12A	0.980
O4-C13	1.433(3)	C12-H12B	0.980
O5-C18	1.363(2)	C12-H12C	0.980
O5-C21	1.432(3)	C13-H13A	0.980
O6-C19	1.360(2)	C13-H13B	0.980
O6-C22	1.436(2)	C13-H13C	0.980
N1-C1	1.358(2)	C14-H14A	0.990
N1-C3	1.462(3)	C14-H14B	0.990
N1-C11	1.458(3)	C14-C15	1.510(3)
C2-H2A	0.980	C15-C16	1.384(3)
C2-H2B	0.980	C15-C20	1.404(3)
C2-H2C	0.980	C16-C17	1.399(3)
C3-C4	1.519(3)	C17-H17	0.950
C3-C14	1.551(3)	C17-C18	1.382(3)
C3-H3	1.01(2)	C18-C19	1.406(3)

C4-C5	1.406(3)	C19-C20	1.386(3)
C4-C9	1.382(3)	C20-H20	0.950
C5-H5	0.950	C21-H21A	0.980
C5-C6	1.383(3)	C21-H21B	0.980
C6-C7	1.415(3)	C21-H21C	0.980
C7-C8	1.378(3)	C22-H22A	0.980
C8-H8	0.950	C22-H22B	0.980
C8-C9	1.410(2)	C22-H22C	0.980
C1-O2-C2	114.7(1)	C10-C11-H11B	109.9
C6-O3-C12	117.5(2)	H11A-C11-H11B	108.3
C7-O4-C13	116.1(2)	O3-C12-H12A	109.5
C18-O5-C21	117.0(2)	O3-C12-H12B	109.5
C19-O6-C22	116.8(1)	O3-C12-H12C	109.5
C1-N1-C3	124.5(2)	H12A-C12-H12B	109.5
C1-N1-C11	119.6(2)	H12A-C12-H12C	109.5
C3-N1-C11	115.8(2)	H12B-C12-H12C	109.5
O1-C1-O2	123.5(2)	O4-C13-H13A	109.5
O1-C1-N1	124.1(2)	O4-C13-H13B	109.5
O2-C1-N1	112.3(2)	O4-C13-H13C	109.5
O2-C2-H2A	109.5	H13A-C13-H13B	109.5
O2-C2-H2B	109.5	H13A-C13-H13C	109.5
O2-C2-H2C	109.5	H13B-C13-H13C	109.5
H2A-C2-H2B	109.4	C3-C14-H14A	109.1
H2A-C2-H2C	109.5	C3-C14-H14B	109.1
H2B-C2-H2C	109.5	C3-C14-C15	112.5(2)
N1-C3-C4	109.9(2)	H14A-C14-H14B	107.8
N1-C3-C14	110.7(2)	H14A-C14-C15	109.1
N1-C3-H3	108(1)	H14B-C14-C15	109.1
C4-C3-C14	111.2(2)	C14-C15-C16	123.8(2)
C4-C3-H3	108(1)	C14-C15-C20	119.3(2)
C14-C3-H3	109(1)	C16-C15-C20	116.8(2)
C3-C4-C5	118.2(2)	Br1-C16-C15	120.6(1)
C3-C4-C9	121.8(2)	Br1-C16-C17	116.9(1)
C5-C4-C9	119.9(2)	C15-C16-C17	122.5(2)
C4-C5-H5	119.5	C16-C17-H17	120.2
C4-C5-C6	121.0(2)	C16-C17-C18	119.5(2)
H5-C5-C6	119.5	H17-C17-C18	120.2
O3-C6-C5	125.6(2)	O5-C18-C17	125.4(2)
O3-C6-C7	115.2(2)	O5-C18-C19	115.0(2)
C5-C6-C7	119.2(2)	C17-C18-C19	119.6(2)
O4-C7-C6	115.2(2)	O6-C19-C18	115.8(2)
O4-C7-C8	125.3(2)	O6-C19-C20	124.8(2)
C6-C7-C8	119.5(2)	C18-C19-C20	119.4(2)
C7-C8-H8	119.4	C15-C20-C19	122.2(2)
C7-C8-C9	121.2(2)	C15-C20-H20	118.9
H8-C8-C9	119.4	C19-C20-H20	118.9
C4-C9-C8	119.1(2)	O5-C21-H21A	109.5

C4-C9-C10	122.2(2)	O5-C21-H21B	109.5
C8-C9-C10	118.7(2)	O5-C21-H21C	109.5
C9-C10-H10A	109.3	H21A-C21-H21B	109.5
C9-C10-H10B	109.3	H21A-C21-H21C	109.5
C9-C10-C11	111.6(2)	H21B-C21-H21C	109.5
H10A-C10-H10B	108.0	O6-C22-H22A	109.5
H10A-C10-C11	109.3	O6-C22-H22B	109.5
H10B-C10-C11	109.3	O6-C22-H22C	109.5
N1-C11-C10	108.8(2)	H22A-C22-H22B	109.5
N1-C11-H11A	109.9	H22A-C22-H22C	109.4
N1-C11-H11B	109.9	H22B-C22-H22C	109.5
C10-C11-H11A	109.9		

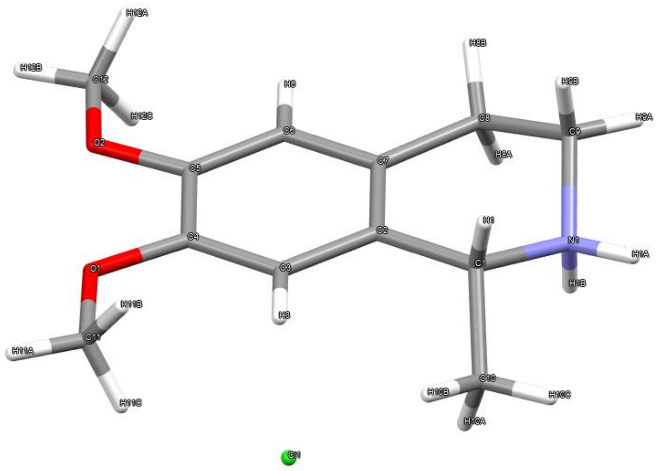

Figure S3. X-Ray structure of salsolidine hydrochloride.

Table S6. Crystal data and structure refinement.

Identification code	14446	
Empirical formula	$C_{12}H_{22}ClNO_4$	
Color	colourless	
Formula weight	279.75 $g \cdot mol^{-1}$	
Temperature	100(2) K	
Wavelength	0.71073 Å	
Crystal system	ORTHORHOMBIC	
Space group	**$P2_12_12_1$, (no. 19)**	
Unit cell dimensions	a = 7.1714(8) Å	α = 90°.
	b = 12.5370(19) Å	β = 90°.
	c = 15.6182(15) Å	γ = 90°.
Volume	1404.2(3) $Å^3$	

Z	4
Density (calculated)	1.323 Mg · m^{-3}
Absorption coefficient	0.279 mm^{-1}
F(000)	600 e
Crystal size	0.25 x 0.09 x 0.07 mm^3
θ range for data collection	3.073 to 33.164°.
Index ranges	-10 ≤ h ≤ 11, -19 ≤ k ≤ 19, -24 ≤ l ≤ 24
Reflections collected	38264
Independent reflections	5356 [R_{int} = 0.0530]
Reflections with I>2σ(I)	4574
Completeness to θ = 25.242°	99.7 %
Absorption correction	Gaussian
Max. and min. transmission	0.98 and 0.95
Refinement method	Full-matrix least-squares on F^2
Data/restraints/parameters	5356/0/194
Goodness-of-fit on F^2	1.042
Final R indices [I>2σ(I)]	R_1 = 0.0306 wR^2 = 0.0713
R indices (all data)	R_1 = 0.0435 wR^2 = 0.0753
Absolute structure parameter	-0.015(19)
Largest diff. peak and hole	0.3 and -0.3 e · $Å^{-3}$

Table S7. Bond lengths [Å] and angles [°].

O1-C4	1.376(2)	C6-H6	0.950
O1-C11	1.428(2)	C6-C7	1.403(2)
O2-C5	1.367(2)	C7-C8	1.515(2)
O2-C12	1.436(2)	C8-H8A	0.990
N1-H1A	0.96(2)	C8-H8B	0.990
N1-H1B	0.86(2)	C8-C9	1.514(2)
N1-C1	1.507(2)	C9-H9A	0.990
N1-C9	1.490(2)	C9-H9B	0.990
C1-H1	0.98(2)	C10-H10A	0.980
C1-C2	1.517(2)	C10-H10B	0.980
C1-C10	1.526(2)	C10-H10C	0.980
C2-C3	1.407(2)	C11-H11A	0.980
C2-C7	1.392(2)	C11-H11B	0.980
C3-H3	0.950	C11-H11C	0.980
C3-C4	1.380(2)	C12-H12A	0.980

C4-C5	1.409(2)	C12-H12B	0.980
C5-C6	1.382(2)	C12-H12C	0.980
C4-O1-C11	116.6(1)	C7-C8-H8A	109.2
C5-O2-C12	116.3(1)	C7-C8-H8B	109.2
H1A-N1-H1B	104(2)	C7-C8-C9	112.2(1)
H1A-N1-C1	111(1)	H8A-C8-H8B	107.9
H1A-N1-C9	110(1)	H8A-C8-C9	109.2
H1B-N1-C1	110(1)	H8B-C8-C9	109.2
H1B-N1-C9	109(1)	N1-C9-C8	108.9(1)
C1-N1-C9	112.6(1)	N1-C9-H9A	109.9
N1-C1-H1	106(1)	N1-C9-H9B	109.9
N1-C1-C2	109.7(1)	C8-C9-H9A	109.9
N1-C1-C10	107.4(1)	C8-C9-H9B	109.9
H1-C1-C2	110(1)	H9A-C9-H9B	108.3
H1-C1-C10	109(1)	C1-C10-H10A	109.5
C2-C1-C10	114.6(1)	C1-C10-H10B	109.5
C1-C2-C3	118.4(1)	C1-C10-H10C	109.5
C1-C2-C7	122.1(1)	H10A-C10-H10B	109.5
C3-C2-C7	119.5(1)	H10A-C10-H10C	109.5
C2-C3-H3	119.7	H10B-C10-H10C	109.5
C2-C3-C4	120.6(1)	O1-C11-H11A	109.5
H3-C3-C4	119.7	O1-C11-H11B	109.5
O1-C4-C3	124.9(1)	O1-C11-H11C	109.5
O1-C4-C5	115.1(1)	H11A-C11-H11B	109.5
C3-C4-C5	120.0(1)	H11A-C11-H11C	109.4
O2-C5-C4	115.1(1)	H11B-C11-H11C	109.5
O2-C5-C6	125.4(1)	O2-C12-H12A	109.5
C4-C5-C6	119.4(1)	O2-C12-H12B	109.5
C5-C6-H6	119.6	O2-C12-H12C	109.5
C5-C6-C7	120.9(1)	H12A-C12-H12B	109.5
H6-C6-C7	119.6	H12A-C12-H12C	109.5
C2-C7-C6	119.6(1)	H12B-C12-H12C	109.4
C2-C7-C8	121.8(1)	H3A-O3-H3B	101(3)
C6-C7-C8	118.6(1)	H4A-O4-H4B	109(3)

References

(1) Nicolaou, K. C.; Valiulin, R. A.; Pokorski, J. K.; Chang, V.; Chen, J. S. Bio-Inspired Synthesis and Biological Evaluation of a Colchicine-Related Compound Library. *Bioorg. Med. Chem. Lett.* **2012**, *22*, 3776–3780.

(2) Selvakumar, J.; Rao, R. S.; Srinivasapriyan, V.; Marutheeswaran, S.; Ramanathan, C. R. Synthesis of Condensed Tetrahydroisoquinoline Class of Alkaloids by Employing TfOH-Mediated Imide Carbonyl Activation. *Eur. J. Org. Chem.* **2015**, *2015*, 2175–2188.

(3) Allin, S. M.; Gaskell, S. N.; Towler, J. M. R.; Page, P. C. B.; Saha, B.; McKenzie, M. J.; Martin, W. P. A New Asymmetric Synthesis of the Anti-Tumor Alkaloid (*R*)-(+)-Crispine A. *J. Org. Chem.* **2007**, *72*, 8972–8975.

(4) Jangir, R.; Argade, N. P. Total Synthesis of Tetrahydroisoquinoline-Based Bioactive Natural Products Laudanosine, Romneine, Glaucine, Dicentrine, and Their Unnatural Analogues Isolaudanosine and Isoromneine. *Synthesis* **2017**, *49*, 1655–1663.

(5) Blank, N.; Opatz, T. Enantioselective Synthesis of Tetrahydroprotoberberines and Bisbenzylisoquinoline Alkaloids from a Deprotonated α-Aminonitrile. *J. Org. Chem.* **2011**, *76*, 9777–9784.

(6) Bali Judicaël Tra, B.; Abollé, A.; Coeffard, V.; Felpin, F.-X. Flow Conditions-Controlled Divergent Oxidative Cyclization of Reticuline-Type Alkaloids to Aporphine and Morphinandienone Natural Products. *Eur. J. Org. Chem.* **2022**, *2022*, e202200301.

(7) Hamamoto, H.; Shiozaki, Y.; Nambu, H.; Hata, K.; Tohma, H.; Kita, Y. The Efficient Synthesis of Morphinandienone Alkaloids by Using a Combination of Hypervalent Iodine(III) Reagent and Heteropoly Acid. *Chem. Eur. J.* **2004**, *10*, 4977–4982.

(8) Li, W.; Jiang, M.; Chen, W.; Chen, Y.; Yang, Z.; Tang, P.; Chen, F. Total Synthesis of (−)-Canadine, (−)-Rotundine, (−)-Sinactine, and (−)-Xylopinine Using a Last-Step Enantioselective Ir-Catalyzed Hydrogenation. *J. Org. Chem.* **2021**, *86*, 8143–8153.

(9) Mastranzo, V. M.; Yuste, F.; Ortiz, B.; Sánchez-Obregón, R.; Toscano, R. A.; García Ruano, J. L. Asymmetric Synthesis of (S)-(−)-Xylopinine. Use of the Sulfinyl Group as an Ipso Director in Aromatic S_E. *J. Org. Chem.* **2011**, *76*, 5036–5041.

(10) Ansari, A.; Gorde, A. B.; Ramapanicker, R. Asymmetric Synthesis of Six Tetrahydroisoquinoline Natural Products through *α*-Amination of an Aldehyde. *Tetrahedron* **2021**, *88*, 132121.

(11) Schönstein, L.; Forró, E.; Fülöp, F. Continuous-Flow Enzymatic Resolution Strategy for the Acylation of Amino Alcohols with a Remote Stereogenic Centre: Synthesis of Calycotomine Enantiomers. *Tetrahedron Asymmetry* **2013**, *24*, 202–206.

(12) Kościołowicz, A.; Rozwadowska, M. D. Diastereoselective Pomeranz-Fritsch-Bobbitt Synthesis of (S)-(-)-Salsolidine Using (R)-N-Tert-Butanesulfinylimine as a Substrate. *Tetrahedron Asymmetry* **2006**, *17*, 1444–1448.

(13) Schönenberger, B.; Brossi, A. Fragmentation of Optically Active (1-Phenylethyl)- and (1-Naphthylethyl)Ureas in Refluxing Alcohols: Easy Preparation of Optically Active Amines of High Optical Purity. *Helv. Chim. Acta* **1986**, *69*, 1486–1497.

(14) Maresh, J. J.; Ralko, A. A.; Speltz, T. E.; Burke, J. L.; Murphy, C. M.; Gaskell, Z.; Girel, J. K.; Terranova, E.; Richtscheidt, C.; Krzeszowiec, M. Chemoselective Zinc/HCl Reduction of Halogenated β-Nitrostyrenes: Synthesis of Halogenated Dopamine Analogues. *Synlett* **2014**, *25*, 2891–2894.

(15) Khunnawutmanotham, N.; Sahakitpichan, P.; Chimnoi, N.; Techasakul, S. Divergent Total Syntheses to Azafluor-anthene and Dehydroaporphine Alkaloids. *Eur. J. Org. Chem.* **2015**, *2015*, 6324–6332.

(16) Milhazes, N.; Calheiros, R.; Marques, M. P. M.; Garrido, J.; Cordeiro, M. N. D. S.; Rodrigues, C.; Quinteira, S.; Novais, C.; Peixe, L.; Borges, F. β-Nitrostyrene Derivatives as Potential Antibacterial Agents: A Structure-Property-Activity Relationship Study. *Bioorg. Med. Chem.* **2006**, *14*, 4078–4088.

(17) Ali, G.; Cuny, G. D. An Efficient Synthesis of an 8-Phenoxy Aporphine Derivative Utilizing Mono-Ligated Palladium Ortho-Phenol Arylation. *Tetrahedron* **2019**, *75*, 4318–4324.

(18) Tian, G.; Fedoseev, P.; Van der Eycken, E. V. Hypervalent Iodine(III)-Mediated Cascade Cyclization of Propargylguanidines and Total Syntheses of Kealiinine B and C. *Chem. Eur. J.* **2017**, *23*, 5224–5227.

(19) Ruiz-Olalla, A.; Würdemann, M. A.; Wanner, M. J.; Ingemann, S.; van Maarseveen, J. H.; Hiemstra, H. Organocatalytic Enantioselective Pictet–Spengler Approach to Biologically Relevant 1-Benzyl-1,2,3,4-Tetrahydroisoquinoline Alkaloids. *J. Org. Chem.* **2015**, *80*, 5125–5132.

(20) Huang, H. M.; Bellotti, P.; Pflüger, P. M.; Schwarz, J. L.; Heidrich, B.; Glorius, F. Three-Component, Interrupted Radical Heck/Allylic Substitution Cascade Involving Unactivated Alkyl Bromides. *J. Am. Chem. Soc.* **2020**, *142*, 10173–10183.

(21) Satyanarayana, G.; Maier, M. E. Synthesis of 1,5-Methano-3-Benzazocines by Intramolecular Buchwald-Hartwig Arylation of 2-Piperidinones. *Tetrahedron* **2008**, *64*, 356–363.

(22) Markad, S. B.; Mane, B. B.; Waghmode, S. B. Asymmetric Total Synthesis of Dihydroisocoumarins: 6-Methoxymellein, Kigelin and Fusarentin 6, 7 Dimethyl Ether by Employing Proline Catalysed Asymmetric α-Aminoxylation. *Tetrahedron* **2020**, *76*, 131524.

(23) Newman, S. G.; Aureggi, V.; Bryan, C. S.; Lautens, M. Intramolecular Cross-Coupling of Gem-Dibromoolefins: A Mild Approach to 2-Bromo Benzofused Heterocycles. *Chem. Commun.* **2009**, No. 35, 5236–5238.
